# SFRP2 induces a mesenchymal subtype transition by suppression of SOX2 in glioblastoma

**DOI:** 10.1038/s41388-021-01825-2

**Published:** 2021-05-21

**Authors:** Min Guo, Kaveh M. Goudarzi, Shiva Abedi, Melanie Pieber, Elin Sjöberg, Jinan Behnan, Xing-Mei Zhang, Robert A. Harris, Jiri Bartek, Mikael S. Lindström, Monica Nistér, Daniel Hägerstrand

**Affiliations:** 1 Department of Oncology-Pathology, Karolinska Institutet, BioClinicum, Solna, Sweden; 2 Department of Radiology, Beijing Tiantan Hospital, Capital Medical University, Beijing, China; 3 Department of Oncology-Pathology, Karolinska Institutet, Science for Life Laboratory, Solna, Sweden; 4 Department of Clinical Neuroscience, Karolinska Institutet, Centre for Molecular Medicine, Solna, Sweden; 5 Department of Immunology, Genetics and Pathology, Rudbeck Laboratory, Uppsala University, Uppsala, Sweden; 6 Division of Molecular Neurobiology, Department of Medical Biochemistry and Biophysics, Karolinska Institutet, Solna, Sweden; 7 Department of Neurosurgery, Albert Einstein College of Medicine, Bronx, NY USA; 8 Department of Medical Biochemistry and Biophysics, Karolinska Institutet, Solna, Sweden; 9 The Danish Cancer Society Research Centre, Copenhagen, Denmark; 10 Department of Molecular Medicine and Surgery, Karolinska Institutet, BioClinicum, Solna, Sweden

**Keywords:** CNS cancer, Tumour heterogeneity

## Abstract

Intratumoral heterogeneity is a characteristic of glioblastomas that contain an intermixture of cell populations displaying different glioblastoma subtype gene expression signatures. Proportions of these populations change during tumor evolution, but the occurrence and regulation of glioblastoma subtype transition is not well described. To identify regulators of glioblastoma subtypes we utilized a combination of in vitro experiments and in silico analyses, using experimentally generated as well as publicly available data. Through this combined approach SOX2 was identified to confer a proneural glioblastoma subtype gene expression signature. SFRP2 was subsequently identified as a *SOX2-*antagonist, able to induce a mesenchymal glioblastoma subtype signature. A subset of patient glioblastoma samples with high *SFRP2* and low *SOX2* expression was particularly enriched with mesenchymal subtype samples. Phenotypically, SFRP2 decreased tumor sphere formation, stemness as assessed by limiting dilution assay, and overall cell proliferation but increased cell motility, whereas SOX2 induced the opposite effects. Furthermore, an SFRP2/non-canonical-WNT/KLF4/PDGFR/phospho-AKT/SOX2 signaling axis was found to be involved in the mesenchymal transition. Analysis of human tumor tissue spatial gene expression patterns showed distinct expression of *SFRP2*- and S*OX2-*correlated genes in vascular and cellular areas, respectively. Finally, conditioned media from SFRP2 overexpressing cells increased CD206 on macrophages. Together, these findings present SFRP2 as a SOX2-antagonist with the capacity to induce a mesenchymal subtype transition in glioma cells located in vascular tumor areas, highlighting its role in glioblastoma tumor evolution and intratumoral heterogeneity.

## Introduction

Glioblastoma is the most aggressive brain tumor type in adults and lacks effective therapeutic options. Glioblastomas are divided into three gene-expression signature-based subtypes denoted proneural, mesenchymal, and classical [1, 2]. Single-cell analyses have revealed that individual glioblastomas contain intermixtures of cells displaying these signatures, suggesting a logical connection between observed intertumoral and intratumoral heterogeneity [3].

The intratumoral glioblastoma heterogeneity is well reported in multiple instances [1, 4–7] and genetically different cell populations have been shown to co-evolve and to be inter-clonally dependent [8]. This is considered to be a major hurdle for successful treatment of glioblastoma, where cells may display differential drug sensitivities, thereby posing an increased risk for developing treatment resistance and the pre-existence of resistant subpopulations, as exemplified in attempts of anti-EGFR treatment and in temozolomide resistance [9–11]. Intratumoral heterogeneity among the malignant cells may arise due to variation of lines and levels of differentiation, co-evolution of populations with different genetic or epigenetic characteristics, local microenvironmental cues or a combination of these that may evoke different phenotypes [12–14]. Frequently occurring genetic alterations in glioblastoma have been connected with different phenotypic states, and cells may also appear in intermediate hybrid states due to plasticity within single tumors [15]. Furthermore, a proneural to mesenchymal transition has been associated with a worse patient outcome and an attraction of microglia cells [2, 3, 16]. The transitions between glioblastoma gene expression subtypes and the spatial organization of different subtype cells in the tumor therefore needs to be further studied for fundamental biological understanding and development of effective treatment against this devastating disease.

We have previously established and classified high-grade glioma cell cultures into two subtypes based on their gene expression patterns, denoted type A and type B cultures [17]. Type A cultures form tumor spheres, grow as intracranial xenografts and express astroglial and stem cell-related genes including *GFAP* (Glial Fibrillary Acidic Protein), *SOX2* (SRY (sex determining region Y)-box 2) and *PDGFRA* (Platelet Derived Growth Factor Receptor α). Type B cultures are defined by a low capacity to grow as tumor spheres and to generate intracranial xenografts, and express mesenchymal-related genes including *FN1* (Fibronectin), *CXCL12,* and *PDGFRB* (Platelet Derived Growth Factor Receptor β). In clinical material, genes highly expressed in type A cultures were correlated with the non-mesenchymal glioblastoma subtype signatures, proneural and classical, whereas genes expressed in type B cultures correlated with the mesenchymal subtype [17]. Since an intermixture of glioblastoma signature displaying cells have been described to occur within individual tumors [3], we herein sought to identify regulators of these signatures and their spatial organization in glioblastomas. To achieve this we used a combined approach of in vitro experiments including overexpression screens, phenotypic profiling, co-immunoprecipitation with subsequent mass spectrometry, and RNA-sequencing, as well as in silico analyses of publicly available data including connectivity map (CMap) and spatial gene expression analyses.

## Results

### Identification of SOX2 and SFRP2 as potential glioblastoma subtype regulators

**Fig. 1:**
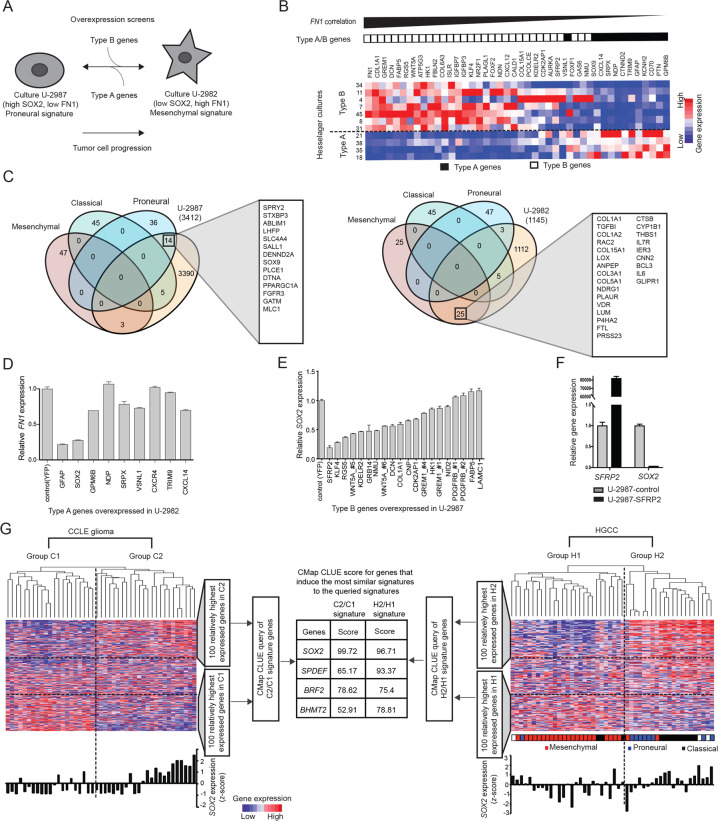
Identification of SOX2 and SFRP2 as antagonistic regulators of gene expression differences between glioblastoma cell lines. **A** Cartoon outlining the setup of the overexpression screens. The arrows indicate the screening of type B genes to identify those that can transition a type A culture into a type B signature, and conversely screening of type A genes to identify those that can transition a type B culture into a type A signature. In this screen, FN1 was used as a proxy for a type B signature and SOX2 as a proxy for a type A signature. **B** Heatmaps illustrating gene expression patterns of type A and type B genes in Hesselager cultures. Cell cultures are arranged from low-to-high expression of *FN1*, followed by a high-to-low arrangement according to *GFAP* expression within the high or low *FN1* expressing groups. In the heatmap, the genes were arranged from left-to-right in descending order according to their overall correlation to *FN1*. The red and blue colors indicate relatively high and low expression levels, respectively. The black and white boxes on top of the heatmap depict type A and type B genes, respectively. **C** Venn diagrams illustrating results from overlap analyses between relatively high (>2-fold) expressed genes in U-2987 versus U-2982 to the left and U-2982 versus U-2987 to the right, and glioblastoma subtype expression signature genes. Number in brackets below each cell line name indicates number of relatively high expressed genes per corresponding cell line. Gene lists indicate overlapping genes for the corresponding highest overlap. **D** Overexpression screen results where *FN1* gene expression levels were monitored by qPCR upon overexpression of mentioned type A genes (Table S1) in culture U-2982. **E** Overexpression screen results where *SOX2* gene expression levels were monitored by qPCR upon overexpression of mentioned type B genes (Table S1) in culture U-2987. The numbering after certain genes, refer to the clone number of used ORFs when several ORFs were cloned. **F** Expression of *SFRP2* and *SOX2* analyzed by qPCR upon SFRP2-overexpression in U-2987. **G** The left and right heatmaps with corresponding dendrograms depict results of hierarchical clustering analysis based on differentially expressed genes of cell lines from CCLE glioma and HGCC, respectively. The highest order cluster branches of the CCLE glioma and HGCC cell lines are denominated C1 and C2, and H1 and H2, respectively. The table in the middle panel lists the top scoring genes from CMap analyses based on differentially expressed genes between cluster branches C1 and C2, and H1 and H2, respectively. The bar graph below each heatmap shows the *SOX2* expression level in each corresponding cell line (*z-*score). For the HGCC cell lines the corresponding predicted glioblastoma gene expression subtype is denoted as reported in the HGCC database.

To experimentally identify potential regulators of glioblastoma gene expression subtype signature we performed two screens by crosswise overexpressing type A genes in a type B culture or type B genes in a type A culture (Fig. 1A). These screens were based on the hypothesis that certain genes in a signature are the ones that have the actual causal effect on the signature. We selected the Hesselager cultures U-2987 (cell culture 18) and U-2982 (cell culture 11) as representative type A and B cultures, respectively (Fig. 1A, B) [17].

To illustrate what glioblastoma subtype these cultures represent their gene expression signatures were compared to other glioblastoma data sets. As previously shown, the type A culture U-2987 expressed high *SOX2* and low *FN1*, whereas the type B culture U-2982 displayed an opposite expression pattern (Fig. 1B) [17]. The type A and B gene expression patterns, arranged by the correlation to *FN1*-expression (Fig. 1B), were also observed among Cancer cell line encyclopedia (CCLE) glioma cell lines (Fig. S1A) and The cancer genome atlas (TCGA) glioblastoma samples (Fig. S1B) [18]. Finally, the U-2987 and U-2982 expression signatures were compared with the recently updated glioblastoma subtype gene expression signatures [2]. Highly expressed genes in U-2987 were enriched in proneural subtype signature genes, 14 out of 50, and in contrast genes highly expressed in U-2982 were enriched in mesenchymal subtype signature genes, 25 out of 50 (Fig. 1C). Thus U-2987 and U-2982 are cultures representative of proneural and mesenchymal glioblastoma subtype signatures, respectively, and will be referred to as such throughout this work.

In our first screen of a mesenchymal-to-proneural transition, type A genes (Table S1) were overexpressed in the mesenchymal culture U-2982, where decreased *FN1*-expression was used as a proxy indicator for a mesenchymal-to-proneural signature change. Here, SOX2 strongly suppressed *FN1*, and was thus considered as a potential mesenchymal-to-proneural transition-inducing gene (Fig. 1D).

Since glioblastoma tumor progression is suggested to proceed from a proneural-like to a mesenchymal subtype [19], our second screen was aimed to identify genes with a capacity to induce a proneural-to-mesenchymal subtype transition. Based on the findings in our first screen, and the low expression of *SOX2* in mesenchymal subtype we sought to identify genes that can suppress *SOX2* in U-2987. Secreted frizzled-related protein 2 (SFRP2), Krüppel-like factor 4 (KLF4), and Regulator of G protein signaling 5 (RGS5) were found to strongly decrease *SOX2* expression, out of 17 screened type B genes (Fig. 1E and Table S1). SFRP2 overexpression and decreased *SOX2* levels were respectively confirmed and recapitulated by qPCR analysis in newly transduced cells (Fig. 1F). Since SFRP2 showed the highest antagonistic effect on *SOX2* expression and had a high novelty value it was selected for further experimentation.

In parallel to the in vitro screens an in silico CMap analysis was performed to identify potential regulators of gene expression signatures. To decrease bias due to cell culture conditions, we extracted gene expression signatures from cell line panels that had been cultured in serum or in neurosphere conditions, CCLE [18] and the Human glioblastoma cell culture resource (HGCC) [20], respectively. Using unsupervised analysis CCLE and HGCC glioma cell lines were hierarchically clustered based on their most differentially expressed genes, yielding two main branches per dataset, denominated C1 and C2, and H1 and H2, respectively (Fig. 1G, Table S2). Subsequently, the 100 highest positively and negatively differentially expressed genes between C2 and C1, or H2 and H1, were used as master signatures for queries in the CLUE (CMap and LINCS Unified Environment) database. A *SOX2* CMap signature was identified to have the highest signature similarity score for both master signatures (Fig. 1G). That is, the top prediction in CLUE, for the signature genes distinguishing the C2 and C1, and the H2 and H1 branches, was that these were contributed to by SOX2 regulation. In support of this, average *SOX2* expression was significantly higher in C2 as compared to C1 (*p* = 0.02), and in H2 versus H1 (*p* = 0.04) (Fig. 1G). Furthermore, the H1 branch was enriched with cell lines of mesenchymal glioblastoma subtype (22/27), and the H2 branch with proneural and classical subtypes (15/21). Together these in vitro and in silico analyses suggested SOX2 as a top regulator of glioma cell culture gene expression signatures irrespective of growth conditions.

### SFRP2 induces a proneural to mesenchymal glioblastoma subtype transition

**Fig. 2:**
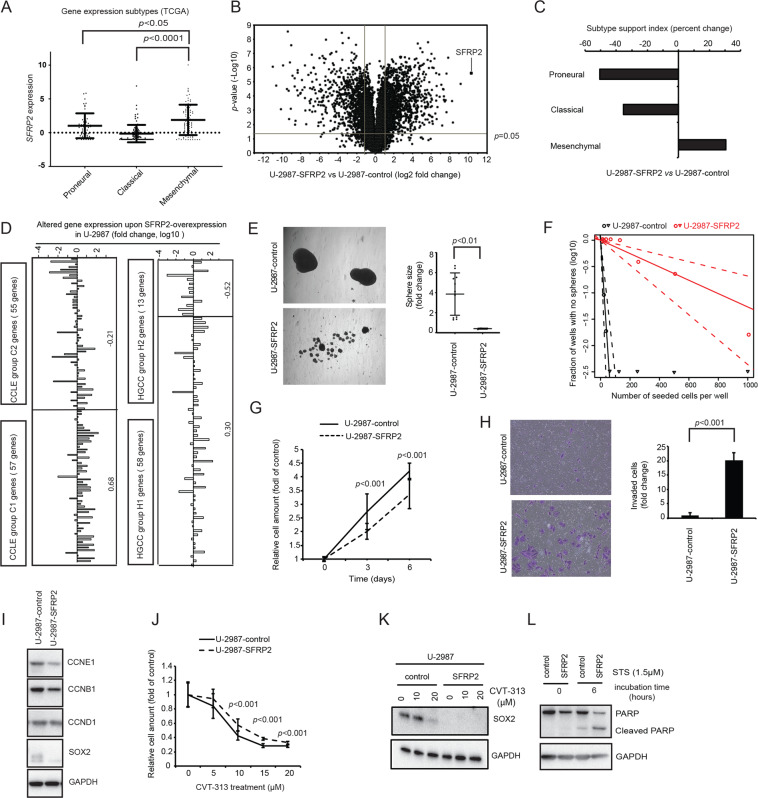
Induction of a mesenchymal gene expression pattern and phenotype upon SFRP2-overexpression in U-2987. **A** Dot-and-whisker graphs for *SFRP2* expression in glioblastoma subtypes among the TCGA samples (unpaired *t*-test). **B** Volcano plots depicting gene expression changes upon SFRP2-overexpression in U-2987. **C** Bar diagram illustrating changes in support index value for glioblastoma subtypes as assessed by RNA-sequencing analysis upon SFRP2-overexpression in U-2987. **D** The gene expression patterns of top 100 C1 and C2 genes in CCLE glioma cell lines (left) and H1 and H2 genes in HGCC (right) upon SFRP2- overexpression in U-2987. The numbers next to the right of the bars indicate the average log10-fold change per group. **E** Tumor sphere formation capacity upon SFRP2-overexpression in U-2987. The bar diagram shows quantification of sphere size (t-test). **F** Line diagram depicts a decrease in self-renewal capacity upon SFRP2-overexpression in U-2987, as analyzed under serum-free conditions using limiting dilution assay **G** Proliferation assessed by differential cell amount upon SFRP2-overexpression and determined by MTT assay after 6 days of growth. **H** Matrigel invasion assay upon SFRP2-overexpression. The bar diagram shows quantification of invaded cells. **I** Western blots showing effects on cyclin proteins CCNE1, CCNB1, CCND1 levels upon SFRP2-overexpression. **J** MTT assay for the proliferation of culture U-2987 upon overexpression of control or SFRP2 and with CVT-313 treatment (0, 5, 10, 15, and 20 μM). **K** Western blot showing SOX2 level upon CVT-313 treatment (0, 10, and 20 μM) for 96 hours in U-2987 with control or SFRP2-overexpression. **L** Western blot showing effects on PARP and cleaved PARP by Staurosporin (STS) treatment after SFRP2-overexpression. For western blot analyses GAPDH levels were added to assess protein input levels in indicated panels.

Upon further analysis of TCGA glioblastomas *SFRP2* was found to be significantly higher expressed in mesenchymal as compared to proneural and classical glioblastoma subtypes (Fig. 2A). To investigate if SFRP2 may induce a mesenchymal subtype signature, the proneural cell culture U-2987 was subjected to RNA-sequencing analysis upon SFRP2-overexpression (Fig. 2B). Indeed, SFRP2-overexpression shifted the subtype support index of this culture from a proneural to a mesenchymal signature (Fig. 2C). In comparison with CCLE and HGCC cluster master signature genes, SFRP2 increased the average expression of C1- and H1-genes by 0.68 and 0.30, and decreased C2- and H2-genes by −0.21 and −0.52, respectively (Fig. 2D). In vitro phenotype analyses after SFRP2-overexpression in U-2987 revealed significantly decreased tumor sphere formation, self-renewal capacity, as well as cell proliferation, and increased Matrigel invasive capacity (Fig. 2E–H).

In the analyses of cell cycle regulator genes upon SFRP2 and SOX2-overexpression in U-2987 and U-2982, respectively, we found an opposite regulation of multiple cell cycle regulator genes, including CCNE1, at both transcript and protein level, and *CDK2* at a transcript level (Fig. 2I and Fig. S2A–C). SFRP2 decreased and increased the ratio of cells in G1- and S-phase, respectively (Fig. S2D), consistent with a de-regulated G1/S cell cycle control. Conversely, SOX2 increased and decreased the ratio of cells in G1- and S-phase, respectively (Fig. S2E). SOX2 has mechanistically been connected to cell cycle regulation by becoming stabilized by G1 cyclins (CCND1 and CCNE1) and their associated cyclin-dependent kinases, including CDK2 [21]. Here we found that SFRP2-overexpression in U-2987 rendered the cells less sensitive to CDK1/2 inhibition by CVT-313 (Fig. 2J). Consistent with a destabilizing effect on SOX2, possibly related to a decrease in G1-S cyclin/CDK proteins, CVT-313 decreased SOX2 protein level in U-2987, whereas SOX2 protein level was already decreased in U-2987 SFRP2-overexpression cells (Fig. 2K). In parallel, SFRP2-overexpression in U-2987 increased the apoptotic response to Staurosporin, as assessed by level of cleaved PARP (Fig. 2L).

In connection to previously reported glioblastoma mesenchymal signature transcription factors, SFRP2 increased and correlated with the expression of *CEBPB*, *RUNX1*, and *FOSL2* in TCGA glioblastomas (Fig. S3A, B) [22].

Collectively, *SFRP2* high expressing glioblastomas were enriched with mesenchymal subtype tumors, and in vitro SFRP2 induced a proneural-to-mesenchymal subtype gene expression signature transition accompanied by decreased stem cell features, de-regulated proliferation, as well as increased Matrigel invasiveness and sensitivity to apoptotic stimuli. This indicated SFRP2 as an inducer of mesenchymal glioblastoma subtype.

### SOX2-overexpression exhibits opposing effects on SFRP2 phenotypes

**Fig. 3:**
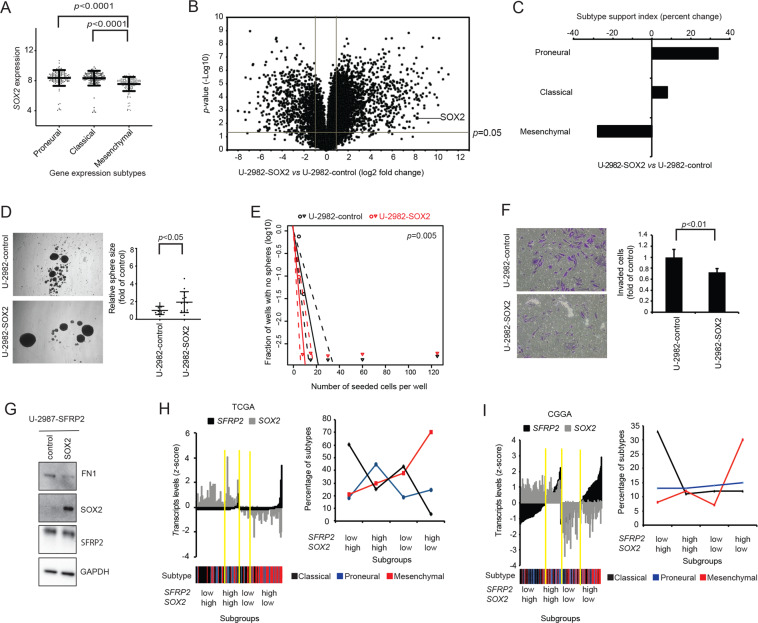
SOX2-overexpression in U-2982 yields opposite effects relative to SFRP2 in U-2987, on gene expression pattern and phenotype. **A** Same as in Fig. 2A but for SOX2 expression. **B**–**F** Same as in Fig. 2B, C, E, F, H, but for SOX2-overexpression in the type B culture U-2982. **G** Western blot experiment to illustrate FN1 and SFRP2 levels in U-2987 with SFRP2-overexpression, with and without re-introduced SOX2. **H** Bar graphs showing the 142 TCGA glioblastoma samples divided into four groups based on their relative gene expression of *SFRP2* and *SOX2*, where the cutoffs are based on mean expression (black bars for SFRP2 with median *z-*score = −0.198, and grey for SOX2 with median *z*-score = 0.076). The heatmap below shows the corresponding described gene expression subtype for each sample in the bar graph. The subtypes are color coded in the heatmap as indicated. The yellow lines separate groups as depicted. The Line plot in the right describes the percent distribution of glioblastoma subtypes within the *SFRP2*- and *SOX2*-based subgroups. **I** Same as Fig. 3H, but for the 180 glioblastoma samples from CGGA database (black bars for SFRP2 with median *z*-score = 1.3, and grey for SOX2 with median *z*-score = 7.01).

To elucidate the connection between SFRP2 and SOX2 we conducted a set of experiments to see if SOX2-overexpression mirrored SFRP2 phenotypes. As opposed to *SFRP2* (Fig. 2A), *SOX2* expression was significantly lower in mesenchymal TCGA glioblastoma as compared to classical and proneural tumors (Fig. 3A). Experimentally, SOX2-overexpression (Fig. 3B) transitioned culture U-2982 from a mesenchymal to a proneural glioblastoma gene expression subtype (Fig. 3C). Furthermore, as opposed to SFRP2 (Fig. 2E, F, H), SOX2-overexpression increased sphere formation and self-renewal capacity, and decreased Matrigel invasion (Fig. 3D–F). In addition, the SFRP2-induced FN1 expression could be reverted by re-introduction of SOX2 in U-2987-SFRP2 cells (Fig. 3G).

Regarding previously reported glioblastoma mesenchymal signature transcription factors, SOX2 as opposed to SFRP2, decreased *CEBPB* and *FOSL2* levels consistent with their negative correlation to SOX2 in TCGA glioblastomas (Fig. S3A, B). In tumor subgroups based on *SOX2* and *SFRP2* transcript levels in the TCGA and The Chinese gliomas genome atlas (CGGA) glioblastoma datasets [23], the respective *SFRP2*^*high*^/*SOX2*^*low*^ subgroups were enriched with mesenchymal subtype tumors (Fig. 3H, I).

In summary, SOX2-overexpression mirrored the SFRP2-overexpression phenotype and a subgroup of glioblastomas with high *SFRP2* and low *SOX2* expression was enriched with mesenchymal subtype tumors.

### SOX2 levels are controlled by an SFRP2/non-canonical-WNT/KLF4/PDGFR/phospho-AKT signaling axis

**Fig. 4:**
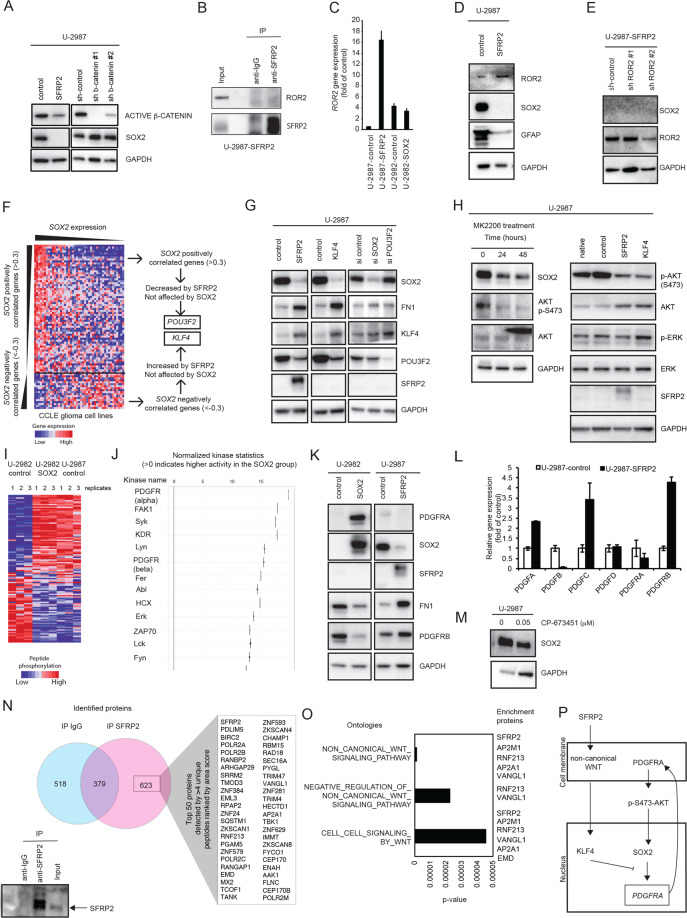
SOX2 levels are controlled by an SFRP2/non-canonical-WNT/KLF4/PDGFR/phospho-AKT signaling axis. **A** Western blots of active β-catenin and SOX2 levels, upon SFRP2-overexpression or suppression of β-catenin in U-2987. **B** Western blots of ROR2 and SFRP2 co-immunoprecipitated by SFRP2 or IgG-control antibodies from U-2987 cells with SFRP2-overexpression. **C** Gene expression levels of *ROR2* determined by RNA-sequencing in U-2987 with or without overexpression of SFRP2, and in U-2982 with or without overexpression of SOX2. **D** Differences in relative protein levels of ROR2, SOX2, and GFAP upon SFRP2-overexpression in U-2987 determined by western blotting. **E** SOX2 levels upon shRNA-suppression of ROR2 in U-2987 cells with SFRP2-overexpression using western blotting. **F** Analysis flowchart for identification of genes as intermediators between SFRP2 and SOX2. **G** Western blot analysis of changes in relative protein levels of SOX2, FN1, and POU3F2, upon overexpression of either SFRP2 or KLF4, and upon siRNA suppression of either SOX2 or POU3F2, as indicated. **H** The left panel shows assessment of SOX2 levels and phosphorylated AKT level at serine residue 473 (S473) in U-2987 after inhibition of AKT activity by addition of the AKT-inhibitor MK2206. The right panel illustrates altered phosphorylated AKT at S473 and total AKT, as well as phosphorylated ERK and total ERK upon SFRP2- or KLF4-overexpression in U-2987 determined by western blotting. **I** Heatmap showing the relative phosphopeptide levels as calculated from a PamGene analysis. The phosphopeptide levels were detected in samples prepared in triplicate from cell culture U-2982-control, U-2982-SOX2, and U-2987-control. **J** Kinase activity prediction plot that shows putative activated kinases upon SOX2-overexpression in U-2982 as compared to control. The predicted activated kinases are ranked according to the normalized kinase statistics value. **K** Analysis of altered PDGFR levels upon SOX2-overexpression in U-2982, and SFRP2-overexpression in U-2987 as determined by western blotting against indicated proteins. Increased FN1 was analyzed as a proxy marker for a mesenchymal transition. **L** Bar graph showing gene expression levels of PDGF-ligands and -receptors upon SFRP2-overexpression in U-2987 as determined by RNA-sequencing. **M** Western blot showing differences in SOX2 levels upon addition of PDGFR inhibitor CP-673451. **N** SFRP2 associated proteins identified by co-immunoprecipitation, control western blot at the bottom left and results from subsequent mass spectrometry analysis to the top right. The blue and red circles indicate number of detected proteins, where the blue circle area denotes the number of proteins detected in the control precipitation, the overlap between the circles the number of proteins detected in both precipitates and the red circle area the number of proteins only detected by the SFRP2 co-immunoprecipitation. The list shows the top 50 proteins according to area score and with more than 4 unique peptide hits. **O** The bar diagram shows selected ontology hits for WNT-related signaling from a query of the proteins listed in panel (**N**). **P** Summary of proposed mechanism of the SFRP2/non-canonical WNT/KLF4/PDGFR/phospho-AKT/SOX2 axis during the mesenchymal transition induced by SFRP2. In all western blots, GAPDH was used to assess protein input levels.

SFRP2 is a secreted protein reported to prevent WNT-ligands from binding to Frizzled receptors and thereby modulate WNT-signaling [24]. Upon investigation of canonical WNT signaling, SFRP2-overexpression indeed decreased level of active β-catenin (Fig. 4A). However, shRNA suppression of β-catenin did not affect SOX2 levels per se. SFRP2 may also act via non-canonical WNT-signaling, including the ROR2 receptor (receptor tyrosine kinase like orphan receptor 2) [25]. No ROR2-SFRP2 interaction could be identified by co-immunoprecipitation (Fig. 4B), but ROR2 expression actually increased upon SFRP2-overexpression in U-2987 (Fig. 4C, D), and subsequent suppression of ROR2 could not restore SOX2 expression to any noticeable degree (Fig. 4E). Together, this did not support a direct involvement of β-catenin or ROR2 in the SFRP2 effect on SOX2.

To identify novel intermediate regulatory factors between SFRP2 and SOX2 we hypothesized that activity of such genes should increase or decrease upon SFRP2-overexpression but not upon SOX2-overexpression, and correlate with *SOX2* expression in the CCLE glioma cell lines. We found that the transcription factors *POU3F2 (OCT7)* and *KLF4* fulfilled these criteria (Fig. 4F). *POU3F2* decreased upon SFRP2-overexpression and correlated positively to *SOX2* expression in the CCLE glioma cell lines. *KLF4* displayed an opposite pattern where it increased upon SFRP2-overexpression and correlated negatively to *SOX2* expression (Figs. 4F and S4). Experimentally, KLF4 indeed increased upon SFRP2-overexpression (Fig. 4G). Direct KLF4-overexpression also decreased SOX2 levels, supporting its involvement (Fig. 4G), and similar to SFRP2, KLF4-overexpression increased FN1 protein levels, suggesting a mesenchymal transition. Conversely, suppression of POU3F2 did not have any effect on SOX2 levels. POU3F2 instead appeared subordinate to both SFRP2 and KLF4, just as SOX2, since both SFRP2- and KLF4-overexpression decreased POU3F2 and SOX2 protein levels (Fig. 4G). In other instances, KLF4 and SOX2 have been connected to PI3K signaling [26]. Consequently, addition of the AKT-inhibitor MK2206, decreasing the phosphorylation of AKT at serine residue 473, resulted in decreased SOX2 levels (Fig. 4H). Finally, both SFRP2- and KLF4-overexpression decreased AKT phosphorylation at serine residue 473 (Fig. 4H). Together, this suggests that the SOX2 protein level is maintained by phosphorylated AKT, and that AKT phosphorylation can be decreased by SFRP2 and KLF4, leading to decreased SOX2 levels. This is in agreement with that SOX2 expression is connected to AKT signaling [27], and that KLF4-overexpression decreases AKT phosphorylation [28].

Upon SOX2-overexpression in culture U-2982 and subsequent phosphoproteomic PamGene analysis, the phosphorylation pattern shifted to resemble the one found in U-2987 cells (Fig. 4I, J, Table S3). The mesenchymal-to-proneural glioblastoma subtype signature transition is thus paralleled by a tyrosine phosphorylation-signature shift. The SOX2-induced phosphorylation pattern was predicted to stem from PDGFR-alpha receptor signaling (Fig. 4J). Indeed, PDGFRA protein appeared upon SOX2-overexpression in U-2982 (Fig. 4K), while SFRP2 in U-2987, in an opposing manner but to a lower extent, decreased PDGFRA levels (Fig. 4K). In addition, *PDGFRA* transcript levels also decreased, and were tracked by decreased *PDGFB* ligand transcript levels (Fig. 4L). Conversely, transcript levels of *PDGFA*, *PDGFC* and *PDGFRB* increased, suggesting a shifted PDGFR-signaling mode. Finally, addition of the PDGFR-inhibitor CP-673451 to U-2987 decreased SOX2 protein levels (Fig. 4M). In summary, SOX2-overexpression increased PDGFRA protein levels and induced a PDGFRA phosphorylation signature, while use of inhibitors of either PDGFR or AKT decreased SOX2 protein levels. On the other hand, SFRP2-overexpression increased KLF4 expression and decreased PDGFRA transcript and protein levels, and both SFRP2- and KLF4-overexpression decreased AKT phosphorylation at serine residue 473.

To identify SFRP2 interaction partners in U-2987 with SFRP2-overexpression we utilized an SFRP2 co-immunoprecipitation and subsequent mass spectrometry analysis approach (Table S4). In the SFRP2 immunoprecipitate we identified SFRP2 itself and 622 further proteins (Fig. 4N and Table S4). Using an ontology enrichment analysis 88 significant ontologies were identified (Table S5). Three of these ontologies were related to non-canonical WNT-signaling (Fig. 4O) with several proteins being in common (Fig. 4O).

In summary, we conclude that the SFRP2-induced SOX2 decrease and mesenchymal transition, occur through a signaling path including non-canonical WNT signaling, KLF4, PDGFRs, and phospho-AKT, comprising both signal transduction events and transcriptional regulation (Fig. 4P).

### SFRP2- expression distributes in vascular areas of glioblastoma tissue

**Fig. 5:**
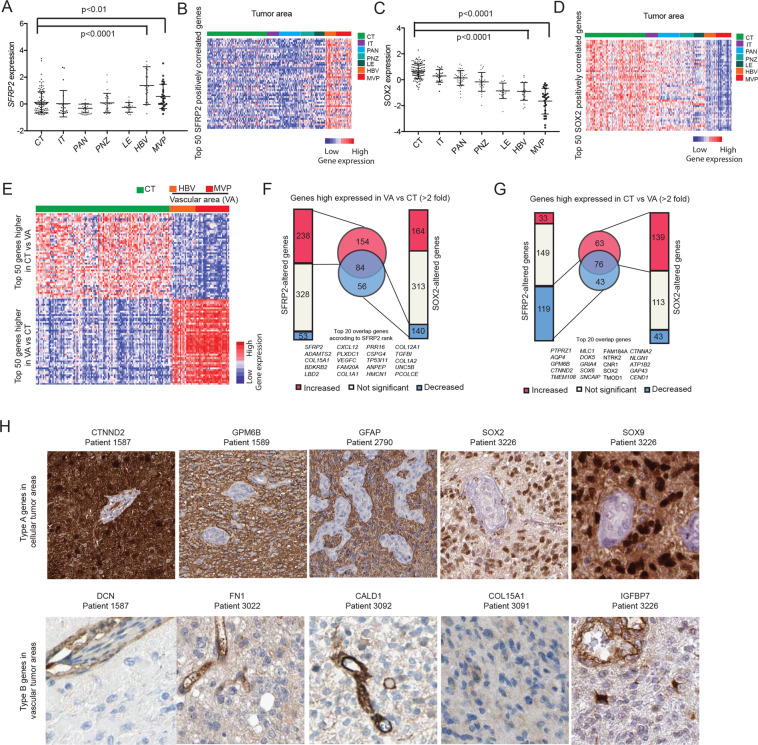
Differential distribution of SFRP2- and SOX2-correlated genes in vascular and cellular tumor areas by spatial gene expression analysis. **A** Dot-and-whisker plots depicting the relative gene expression of *SFRP2* in different areas of laser capture micro dissected glioblastoma samples from the Ivy Glioblastoma Atlas project including cellular tumor (CT), infiltrating tumor (IT), pseudopalisading tumor (PAN), perinecrotic zone (PNZ), leading edge (LE), hyperplastic blood vessel (HBV) and microvascular proliferation (MVP) (unpaired t-test). **B** Heatmap showing distribution of relative gene expression distribution for the top 50 *SFRP2* positively correlated genes in the dataset. (**C**) and (**D**), are as (**A**) and (**B**), respectively, but with regard to *SOX2* expression. **E** Heatmap with the top 50 genes showing the highest differential distribution between CT and VA (Vascular Area, including HBV and MVP). **F** Strip charts showing the number of genes altered by SFRP2- (left) or SOX2- (right) overexpression among the genes higher expressed in VA than CT area (>2-fold). Venn diagram (middle) shows the number of overlapping genes between SFRP2-increased and SOX2-decreased genes. **G** Strip charts showing the number of genes altered by SFRP2- (left) and SOX2- (right) overexpression among the genes higher expressed in CT than VA area (>2-fold). Venn diagram (middle) shows the number of overlapping genes between SFRP2-decreased and SOX2-increased genes. **H** Immunohistochemical staining patterns of selected type A genes in CT areas and type B genes in VA areas in glioma tumor samples from the Human Protein Atlas.

To investigate the spatial distribution of *SFRP2* and *SOX2* cell populations in an intratumoral heterogeneity perspective we analyzed publicly available gene expression data from laser capture-dissected areas in glioblastomas [29] and compared these patterns with the SFRP2- and SOX2-regulated genes identified in our in vitro experiments (Table S6). *SFRP2* was significantly higher expressed in HBV (Hyperplastic blood vessels) and MVP (Microvascular proliferation) areas versus CT (Cellular tumor) areas (Fig. 5A). Furthermore, the top 50 positively *SFRP2*-correlated genes in the tissue were highly expressed in HBV and MVP areas (Fig. 5B). Conversely, *SOX2* displayed an opposite pattern (Fig. 5C). The top 50 *SOX2* positively correlated genes were highly expressed in CT area (Fig. 5D). In a focused analysis of genes differentially expressed between VA (Vascular Areas: composed of HBV and MVP) and CT areas (Fig. 5E, Table S7) we found that 238 out of 619 (38%) VA genes were increased by SFRP2, out of which 84 also overlapped with SOX2-decreased genes from our experiments (Fig. 5F). Conversely, 119 out of 301 (40%) CT genes were decreased by SFRP2, and 139 (46%) increased by SOX2, with an overlap of 76 genes (Fig. 5G). In conclusion, genes highly expressed in VA areas overlap with SFRP2 increased genes, whereas genes highly expressed in CT areas overlap with SOX2 increased genes.

By analyzing glioma tissue stainings from the Human Protein Atlas, a set of type A genes were found expressed in CT areas, whereas type B genes were found expressed in vessel related structures (Fig. 5H). In summary, these tissue analyses indicate that *SFRP2* and its correlated genes and regulation in tissue and in vitro show both RNA and protein distribution in VA areas, whereas RNA and proteins for genes related to *SOX2* are distributed in CT areas. This implies that mesenchymal subtype glioblastoma cells preferentially reside in vascular tumor areas and proneural subtype cells in cellular tumor areas.

### SFRP2 and SOX2 affect pericyte markers and macrophage M2 polarization

**Fig. 6:**
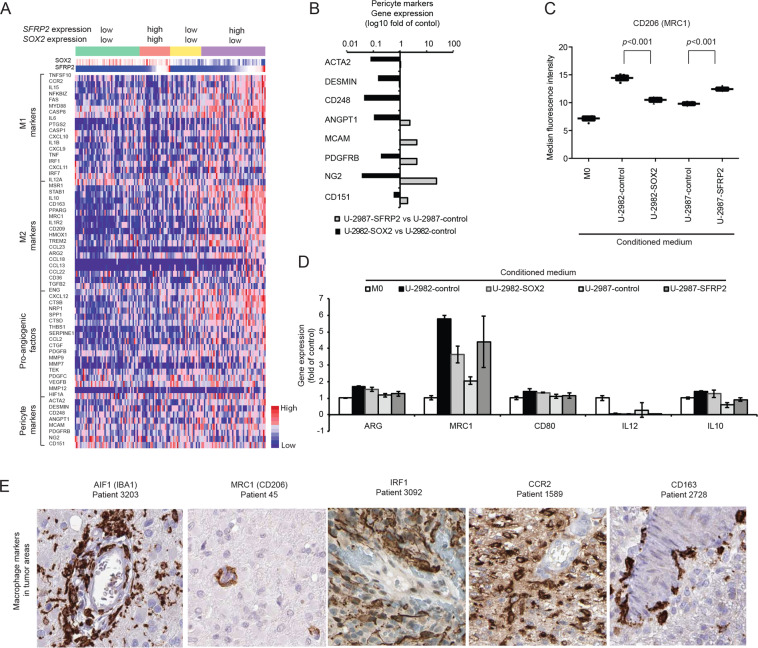
SFRP2 and SOX2 affect pericyte markers and macrophage M2 polarization. **A** Heatmap showing the gene expression of macrophages (M1 and M2), pro-angiogenic factors and pericyte markers in the four gene expression subtypes divided by the median expression level of *SFRP2* and *SOX2* in TCGA samples. **B** Bar graph revealing the regulation of pericyte markers by SFRP2- (gray) or SOX2- (black) overexpression. **C** Monitoring of CD206 median fluorescence intensity by FACS on human monocytes after treatment with conditioned media from U-2982 or U-2987 with/without SOX2- or SFRP2-overexpression, respectively. **D** Same conditions as in 6 C, but showing the effect on *ARG*, *MRC1*, *CD80*, *IL-12*, *IL-10* gene expression in human monocytes analyzed by qPCR. **E** The immunohistochemical staining patterns of selected macrophage markers in glioblastomas from the Human Protein Atlas.

Immune cells such as T cells and tumor-associated macrophages (TAMs) occur more frequent in mesenchymal glioblastomas [30]. We tested if secreted SFRP2 may affect surrounding cells by analyzing genes related to angiogenesis, pericytes and macrophages in *SFRP2*/*SOX2* expression subgroups (Fig. 3H). Elevated expression of macrophage and pericyte markers appeared in the *SFRP2*^high^/*SOX2*^low^ group (Fig. 6A). According to our RNA-sequencing data, SFRP2- overexpression increased the expression of pericyte markers (5/8), whereas SOX2-overexpression decreased such markers (7/8) (Fig. 6B).

Experimentally, conditioned media from SFRP2-overexpressing cells increased CD206 (MRC1) expression on macrophages, whereas SOX2-overexpression conversely diminished CD206 expression (Fig. 6C). Gene expression analysis supported this increase of MRC1, whereas other macrophage markers including ARG, CD80, IL-10 and IL-12 showed minor changes (Fig. 6D). Tissue data from the Human Protein Atlas showed a confinement of macrophage proteins in vascular areas, consistent with the distribution of SFRP2-related gene expression in glioblastoma tissue described above (Fig. 6E). Together this suggests an involvement of SFRP2 in the effect of mesenchymal subtype cells on pericytes and macrophages in glioblastoma.

## Discussion

This work presents SFRP2 and SOX2 as counteracting inducers of gene expression based glioblastoma subtypes, where SFRP2 suppresses SOX2 via non-canonical WNT signaling, KLF4, PDGFRs, and AKT. SFRP2 transitions cells from a proneural into a mesenchymal glioblastoma gene expression signature, suggesting its involvement in glioblastoma tumor cell plasticity and tumor progression.

In this study SOX2 was initially identified as an inducer of glioblastoma subtype transition in an experimental overexpression-screen, and subsequently SOX2 was found through a CMap analysis to be at the apex of gene expression determination in glioblastoma cultures [31, 32]. SOX2 is a well-described stem cell transcription factor that together with KLF4, OCT4 and MYC constitute the Yamanaka factor quartet that together can induce pluripotent stem cells [33]. SOX2 is expressed in neurogenic regions in human brain including the subventricular zone and in glioblastoma SOX2 is essential for maintaining a tumor- and sphere forming cell phenotype [17, 34]. It is also in combination with POU3F2, SALL2, and OLIG2, able to induce glioblastoma tumor growth [35]. Interestingly, we find in this study that both SOX2 and POU3F2 appear subordinate to both SFRP2 and KLF4. SOX2 protein can be stabilized via phosphorylation by a CDK2/CCNE1 complex [21]. We here show that SFRP2-overexpression decreases CCNE1, CCND1 and CCNB1, and at the same time decreases SOX2 protein and overall cell proliferation in glioblastoma cells. We suggest that inactivation of the CDK-inhibitor p27(KIP) may be important here. P27 binds to and prevents activation of CDK2/CCNE1 or CDK4/CCND1 and is involved in blocking the cell cycle in the G0/G1 phase [36]. Additionally, p27 can be inactivated via AKT activation [37].

SFRP2 is a secreted factor reported to antagonize WNT signaling by sequestering of WNT ligands and has been connected with increased tumor growth, metastasis and therapy resistance [38], but has also been described as a tumor suppressor [24]. In this study we found that SFRP2 suppressed β-catenin, consistent with recent findings [39]. However, the decreased SOX2 levels observed here appeared independent of active β-catenin. Nor did we find any experimental support of SFRP2 acting via ROR2 [25] to regulate SOX2 levels. Although, we noted that ROR2 transcript and protein levels actually increased upon SFRP2-overexpression. In our co-immunoprecipitation and mass spectrometry analysis for SFRP2-interactors we instead detected other non-canonical WNT-signaling components including VANGL1, a transmembrane protein associated with non-canonical WNT-signaling recently reported to alter cell motility in glioblastoma [40].

Through a combined analysis approach of gene expression pattern and gene regulation data, KLF4 was identified as an intermediate factor between SFRP2 and SOX2, increased by SFRP2, subsequently leading to decreased SOX2 gene expression and protein levels. As a Yamanaka factor [33] KLF4 has been reported to associate with SOX2 to regulate gene transcription and induce pluripotency [41]. However, in glioblastoma *KLF4* expression appears negatively correlated with *SOX2* expression. KLF4 is higher expressed in mesenchymal, type B, glioblastoma cultures and was here found to act as a SOX2 antagonist.

Based on the data on PDGF ligands and receptors in our RNA-sequencing and PamGene data, confirmed by western blot analyses, the PDGFR/PI3K/AKT signaling axis caught our attention. With regard to previous observations of an active PDGFRA signaling loop in glioblastoma [42] we here experimentally show this can be maintained by SOX2, and subsequently turned off by SFRP2, potentially acting via KLF4. Furthermore, phosphorylation of AKT at serine 473 has been reported to regulate SOX2 protein levels and self-renewal activity [43, 44]. Here AKT phosphorylation decreased upon SFRP2- or KLF4-overexpression, and treatment by AKT inhibitor MK2206 or PDGF inhibitor CP-673451 both decreased SOX2 protein levels. This adds the inhibition of the PDGFR-AKT pathway as key for the antagonistic effect of SFRP2 on SOX2. Furthermore, we find it notable that PDGFRA and GFAP proteins were highly expressed in the proneural culture U-2987 and decreased upon the SFRP2 induced mesenchymal transition. *Pdgfra*^*+*^*/Gfap*^*+*^ neural stem cells in the subventricular zone of the adult mouse brain have been identified as the origin of PDGF-induced gliomas [45]. The proneural culture U-2987 also expressed high levels of PTPRZ1, which together with PDGFRA is a marker of oligodendrocyte precursors [46], indicating a mixture of astrocyte and oligodendrocyte progenitor characteristics.

Our study also highlights the significance of spatial tumor cell distribution. The analysis of SFRP2- and SOX2-regulated and correlated genes revealed their distinct allocation in vascular or cellular areas, respectively, providing a clue to how intratumoral heterogeneity may evolve. Our experimental findings of SFRP2 and SOX2 signaling events represent a scenario whereby SFRP2 counteracts SOX2 function allowing glioma cell progression from a proneural towards a mesenchymal phenotype. In concert with our observed SFRP2-induced pericyte profile, SOX2 expressing glioblastoma cells have been shown in xenograft models to be able to lose SOX2 expression to generate tumor vessel cell structures that resemble pericytes [47]. Furthermore, we found higher levels of macrophage, pericyte, and pro-angiogenic factors in *SFRP2*^*high*^ areas, consistent with the vascular distribution of expressed SFRP2 and correlating genes, suggesting an effect of SFRP2 on the surrounding microenvironment. Other secreted factors from SFRP2-overexpressing cells, including CXCL12 have been shown important for the recruitment of immune cells in glioblastoma [48]. However, what triggers the increased SFRP2 levels remains to be identified.

In conclusion, this work suggests an important role of SFRP2 in the transition from a proneural to a mesenchymal glioblastoma subtype via suppression of SOX2 during tumor progression. A role in intratumoral heterogeneity is suggested by the spatial distribution or SOX2 and SFRP2 expressing cells in glioblastoma tumors. In extension, we suggest that combined targeting of SFRP2 and SOX2 signaling should be investigated to achieve combined anti-angiogenic, anti-immunogenic and anti-proliferative treatment effects.

## Materials and methods

### Cell culture

The Hesselager glioma cultures 11 and 18 have respectively been referred to as U-2982 and U-2987 in other studies [16, 49] and are here referred to as such for coherence. Cells were maintained in Minimum Essential Medium (MEM, Gibco) containing 10% FBS and 1% penicillin-streptomycin at 37 °C with 5% CO_2_, except in sphere formation assay and limiting dilution assay, where cells were cultured in neurosphere medium (#05751 Neurocult NA-S proliferation human kit; StemCell Technologies), supplemented with 20 ng/ml EGF (Invitrogen), 20 ng/ml bFGF (Invitrogen), and 2 μg/ml heparin (Sigma). The identities of the cell cultures were confirmed by STR profiling at NGI-Uppsala, SciLifeLab, Uppsala University, using the AmpFISTR Identifiler PCR Amplification kit (Thermo Fisher) (Table S8). For further information please see supplemental material and methods.

### Publicly available data

Gene expression datasets for glioblastoma samples were downloaded from the following resources: Hesselager cultures from EMBL-EBI ArrayExpress (accession number E-MEXP-1063) [50]; CCLE at www.broadinstitute.org/ccle [18] from which 45 glioma cell lines were selected based on literature and information provided at the CCLE site (Table S9); HGCC from https://www.hgcc.se/ [20]; TCGA (Firehose Legacy, previously known as TCGA provisional) for 116 glioblastomas from http://cbioportal.org (G-CIMP, neural, and non-subtype categorized samples excluded); CGGA data for 180 glioblastomas from http://gliovis.bioinfo.cnio.es/; spatial gene expression data from Ivy Glioblastoma Atlas Project (http://glioblastoma.alleninstitute.org) [29]. Differential gene expression analyses, hierarchical clustering, Gene Set Enrichment Analysis (GSEA), and heatmap generation were performed at the online GenePattern server at https://genepattern.broadinstitute.org [51]. Immunohistochemical staining data was retrieved from The Human Protein Atlas (HPA, https://www.proteinatlas.org/) [52]. Clustering of genes in CCLE and HGCC is shown in supplemental methods.

### Generation of stable cell cultures overexpressing either type A or type B genes

Fifteen type A genes and thirty type B genes were selected based on their differential expression between 23 high-grade glioma cultures according to methods previously described [50] (Table S1). For further information about overexpression screens, establishment of gene overexpressing or knockdown cell cultures, please see supplemental material and methods.

### RNA sequencing, qPCR, and Western blot

RNA-sequencing was performed as previously described [16] and data is deposited at EBI ArrayExpress (E-MTAB-7591). Detailed information on qPCR and Westernblot is found in supplemental material and methods. Primers and antibodies are shown in Table S10.

### Glioblastoma gene expression subtype signature support index

SFRP2- or SOX2-regulated genes were compared with subtype-defining centroid genes as previously described [1, 16]. For further information see supplemental material and methods.

### Phenotype assays

Cell phenotype assays, including tumor sphere formation, limiting dilution assay, Matrigel invasion, cell proliferation assay, and cell cycle analysis were performed. For detailed information please see supplemental material and methods.

### Global protein tyrosine kinase (PTK) assay

The protein tyrosine kinase assay with cell lysates performed on PamChip arrays on the PamStation®12 is a flow-through microarray assay to determine the activity of tyrosine kinases. For further information see supplemental material and methods.

### Co-immunoprecipitation and subsequent mass spectrometry analysis

Co-immunoprecipitations were performed by using Dynabeads protein G (10003D, Invitrogen) according to the manufacturer’s instruction in combination with IgG mouse (CS200621, Millipore) or anti-SFRP2 (sc-365524, Santa Cruz) antibodies. Further information about the co-immunoprecipitation conditions and subsequent mass spectrometry analysis can be found in supplemental material and methods.

### Statistical Analysis

Data in bar graphs depict mean ± standard deviation. The laboratory experiments were repeated independently three times. Comparisons between the two groups were assessed using Student’s *t*-tests (two-tailed) and χ^2^ tests. *p* < 0.05 was considered to be statistically significant. Correlation rates were assessed by Pearson’s correlation test.

## Supplementary information

Supplemental material and methods

Supplemental tables
