# Correction to: SFRP2 induces a mesenchymal subtype transition by suppression of SOX2 in glioblastoma

**DOI:** 10.1038/s41388-021-01929-9

**Published:** 2021-07-13

**Authors:** Min Guo, Kaveh M. Goudarzi, Shiva Abedi, Melanie Pieber, Elin Sjöberg, Jinan Behnan, Xing-Mei Zhang, Robert A. Harris, Jiri Bartek, Mikael S. Lindström, Monica Nistér, Daniel Hägerstrand

**Affiliations:** 1 Department of Oncology-Pathology, Karolinska Institutet, BioClinicum, Solna, Sweden; 2 Department of Radiology, Beijing Tiantan Hospital, Capital Medical University, Beijing, China; 3 Department of Oncology-Pathology, Karolinska Institutet, Science for Life Laboratory, Solna, Sweden; 4 Department of Clinical Neuroscience, Karolinska Institutet, Centre for Molecular Medicine, Solna, Sweden; 5 Department of Immunology, Genetics and Pathology, Rudbeck Laboratory, Uppsala University, Uppsala, Sweden; 6 Division of Molecular Neurobiology, Department of Medical Biochemistry and Biophysics, Karolinska Institutet, Solna, Sweden; 7 Department of Neurosurgery, Albert Einstein College of Medicine, Bronx, NY USA; 8 Department of Medical Biochemistry and Biophysics, Karolinska Institutet, Solna, Sweden; 9 The Danish Cancer Society Research Centre, Copenhagen, Denmark; 10 Department of Molecular Medicine and Surgery, Karolinska Institutet, BioClinicum, Solna, Sweden

**Keywords:** CNS cancer, Tumour heterogeneity

Correction to: *Oncogene* 10.1038/s41388-021-01825-2, published online 21 May 2021

Unfortunately, the second corresponding author was not given in the Original article.

The second corresponding author is:

Daniel Hägerstrand, Email: daniel.hagerstrand@ki.se

The original article has been corrected.

