# Supplementary material for: SFRP2 induces a mesenchymal subtype transition by suppression of SOX2 in glioblastoma
Source: Oncogene. 2021 May 21;40(32):5066–80. doi: 10.1038/s41388-021-01825-2 (PMC8363098; doi:10.1038/s41388-021-01825-2)
Supplement: Supplementary file 1 — Supplemental material and methods [file 41388_2021_1825_MOESM1_ESM.docx]

**SUPPLEMENTARY DATA**

# **SFRP2 induces a mesenchymal subtype transition by suppression of SOX2 in glioblastoma**

Min Guo^1,2*^, Kaveh M. Goudarzi^3^, Shiva Abedi^1^, Melanie Pieber^4^, Elin Sjöberg^5^, Jinan Behnan^6,7^, Xing-Mei Zhang^4^, Robert A. Harris^4^, Jiri Bartek^8,9^, Mikael S. Lindström^8^, Monica Nistér^1^, Daniel Hägerstrand^10*^

Author affiliations: ^1^Department of Oncology-Pathology, Karolinska Institutet, BioClinicum, SE-171 64 Solna, Sweden. ^2^Department of Radiology, Beijing Tiantan Hospital, Capital Medical University, Beijing, China. ^3^Department of Oncology-Pathology, Karolinska Institutet, Science for Life Laboratory, SE-171 65 Solna, Sweden. ^4^Department of Clinical Neuroscience, Karolinska Institutet, Centre for Molecular Medicine, Karolinska University Hospital at Solna, SE-171 76 Stockholm, Sweden. ^5^Department of Immunology, Genetics and Pathology, Rudbeck Laboratory, Uppsala University, SE-751 85 Uppsala, Sweden. ^6^Division of Molecular Neurobiology, Department of Medical Biochemistry and Biophysics, Karolinska Institutet, SE-171 65 Solna, Sweden. ^7^Department of Neurosurgery, Albert Einstein College of Medicine, Bronx, 104 67 NY, USA. ^8^Department of Medical Biochemistry and Biophysics, Karolinska Institutet, SE-171 21 Solna, Sweden. ^9^The Danish Cancer Society Research Centre, DK-2100, Copenhagen, Denmark. ^10^Department of Molecular Medicine and Surgery, Karolinska Institutet, BioClinicum, SE-171 64 Solna, Sweden.

## Supplemental materials and methods

### Cell culture and quality control

U2982 and U2987 were confirmed to not express R132H-mutated IDH1 protein. HEK293T cells overexpressing R132H-mutated IDH1 was used as a positive control in western blot assay (data not shown). The medium from cultured cells (MEM with 10%FBS and 1%PEST) were checked for Mycoplasma using the MycoAlert**™** Mycoplasma Detection Kit (LT07-218) (Lonza).

### CMap analysis

All genes in CCLE or HGCC were arranged according to high to low standard deviation among samples. The top 300 genes were clustered by hierarchical clustering with pairwise average linkage as clustering methods and pearson correlation as distance measure into two groups in the online GenePattern server, and named group C1 and C2 in CCLE, H1 and H2 in HGCC. The 100 highest expressed genes in Group 1 (C1 or H1) and Group 2 (C2 or H2) were subsequently subjected to a CMap query in the CLUE data base (<https://clue.io/query>) [1, 2]. Genes in the CLUE database that generated expression signatures that resembled the C2/C1 or H2/H1 signatures were retrieved and ranked according to a high to low CLUE score. The CMap CLUE score denotes the similarity of the higher and lower expressed genes between the C1 and C2, and H1 and H2 branches and the overexpression induced gene expression signatures in the CMap database. The scores based on the CCLE glioma cell lines and HGCC database analyses were summarized and used to identify genes with the overall highest score. Score ranges from -100 to 100, where 100 denotes a 100% similarity, 0 no similarity, and -100 a 100% opposing signature.

### Overexpression screen for identification of glioblastoma subtype transition genes

The type B culture U-2982 was used to screen for type A genes that can transition a type B culture into a type A signature. A decreased *FN1* expression level, determined by qPCR, was used as a proxy for the transition from a type B into type A signature. Conversely, the type A culture U-2987 was used to screen type B genes that can transition a type A culture into a type B signature. A decreased *SOX2* expression level, determined by qPCR, was used as a proxy for the transition from a type A into type B signature.

### Generation of gene-overexpressing cell cultures by lentiviral transduction

cDNA constructs for 9/15 type A genes and 17/30 type B genes available in the Human ORFeome [3] were retrieved from the High Throughput Screening center at the Department of Medicine, Karolinska Institutet, Sweden. The ORFs were transferred by Gateway cloning into the lentiviral overexpression vector pLEX-BLAST-V5-DEST and lentiviral particles were produced as previously described [4], and subsequently used to generate overexpressing cell lines of cell cultures U-2982 and U-2987. An YFP expressing lentiviral vector was used in parallel as negative control. Transduced cells were selected by growth in 2 μg/ml blasticidin.

### Generation of gene knock-down cell cultures by shRNA and siRNA transfection

Gene knockdown by transducing shRNA plasmids was performed by using lentiviral particles as previously described [4]. shRNA plasmids for targeting β-catenin (TRCN0000314921, TRCN0000314990) and controls were purchased from Sigma, and the transduced cells were selected in the presence of 1 μg/ml puromycin. For siRNA experiments, SMARTpool: ON-TARGETplus siRNAs were purchased from GE Healthcare Dharmacon, and they were transfected into cells with Lipofectamine™ RNAiMax (Invitrogen) according to the manufacturer's instructions. The siRNAs used in this work were: SOX2 (L-011778-00-0005), POU3F2 (L-020029-00-0005) and non-targeting control (D-001810-00-0005).

### RNA sequencing and gene expression

Total RNA was purified using the PureLink® kit and RNA sequencing analysis upon SFRP2 or SOX2 overexpression was performed at the National Genomics Infrastructure (NGI) at Science for Life Laboratory (SciLifelab), Stockholm as previously described [15]. To compare the generated RNA sequencing data with glioma gene expression data from CCLE database, expression values for the corresponding genes found at the CCLE and HGCC data sources (Affymetrix platform generated data) were extracted. Subsequently the average values per gene derived from 3 replicates per group were compared between groups: a) glioblastoma culture U-2982-control versus U-2982-SOX2; b) glioblastoma culture U-2987-control versus U-2987-SFRP2; and c) U-2982-control versus U-2987-control. Genes were filtered away if the average values in both groups were less than 0.5. Venn diagrams were generated using GeneVenn (<http://genevenn.sourceforge.net)> to identify the overlapping genes between different compared gene lists. qPCR was performed with SYBR green master mix (Applied Biosystems). Gene expression levels were assessed using a 7500 Real-Time PCR system, with *GAPDH* as a control for cDNA input, where each sample was measured in triplicates.

### Western blot

For western blots analyses, cells were harvested in RIPA buffer supplemented with protease and phosphatase inhibitors (Roche) for 20 min on ice. Lysate was then mixed with Nupage LDS sample buffer and sample reduction agent (Invitrogen) and boiled for 5 min. Western blots were performed by SDS-PAGE and transferred to nitrocellulose filters by using Transblot® Turbo™ Transfer system (BioRad).

***Glioblastoma gene expression subtype signature support index***

Each glioblastoma gene expression subtype is defined by a set of 210 centroid genes where a support index value of 100 means all the genes are altered in the positive direction, 0 means no changes, and -100 means a full change in the negative direction. Where mentioned the updated subtype signatures according to Wang et al. [5], with 50 genes for each subtype, were used to assess subset signature resemblance by assessment of overlap between these defined standard signature genes and observed highly expressed genes as illustrated by Venn diagrams.

### Tumor sphere formation assay and limiting dilution assay

Cells were plated in non-adherent 6-well plates at 20,000 cells/well in neurosphere medium (#05751 Neurocult NA-S proliferation human kit; StemCell Technologies), supplemented with 20 ng/ml EGF (Invitrogen), 20 ng/ml bFGF (Invitrogen), and 2 μg/ml heparin (Sigma).  Spheres were split and re-plated after 7 days and culturing continued for another 7 days, then images were taken. The number of spheres and their size were determined using Image J. In limiting dilution assays cells were plated in non-adherent 96-well plates with diluted cell number ranging from 1000 cells/well to 2 cells/well (1000, 500, 250, 125, 63, 32, 16, 8, 4, 2) with 6 replicates per condition in neurosphere medium. The number of wells with spheres was recorded after 3 weeks. The results were analyzed using Extreme Limiting Dilution Analysis (ELDA) (n=6) [6]. The experiments were repeated independently for three times.

### Matrigel invasion, cell proliferation assay, and cell cycle analysis

Matrigel invasion capacity of glioma culture cells was determined by Corning´s invasion chamber system as previously described [4] (Corning, cat. #354480). In short, for the Matrigel invasion assay 50,000 cells were plated per top-chamber-well in low-serum medium (MEM with 1% FBS), and put into bottom-chamber with regular medium (MEM with 10% FBS and 1% PEST). After 48 hours’ incubation, the invaded cells were stained by crystal violet staining solution and counted. Cell proliferation was measured by MTT assay. For MTT assays, 5,000 cells/well were seeded in 96-well plates and cultured for one week. At the end of experiment staining with 500 μg /ml MTT was performed and the absorbance was measured at 540nm. For cell cycle analysis, U-2982-control, U-2982-SOX2, U-2987-control, and U-2987-SFRP2 were seeded at 200,000 cells/well in 6-well plates in triplicate. After 48 hours culture, cells were collected and fixed with ice-cold 70% ethanol, then washed and stained with DAPI for 30 minutes at room temperature and finally subjected to cell cycle analysis by FACS. The experiments were repeated independently three times.

### Identification of genes acting as intermediators between SFRP2 and its effect on SOX2 transcription levels

The glioma cell lines from CCLE were arranged from high to low *SOX2* expression. The genes with the highest positive or negative correlation with *SOX2* (-0.3< r >0.3) were arranged in a falling order. Of the genes positively correlated to *SOX2* in the CCLE analysis, only the ones that were decreased by SFRP2-overexpression and did not change significantly upon SOX2-overexpression were considered. Of the *SOX2* negatively correlated genes from the CCLE analysis, only those that were increased by SFRP2-overexpression and did not change significantly upon SOX2-overexpression were considered. In the end, transcription factors among these genes were considered as potential intermediators between SFRP2 and its effect on *SOX2* transcript levels.

### Global protein tyrosine kinase (PTK) assay

The phosphorylation activity is determined by using peptides immobilized on the PamChip. The activity is detected with a fluorescently labeled antibody and recorded by CCD-camera in the PamStation ^®^12. 2x10^6^ cells were seeded in a 10 cm dish and incubated for 2 days. Then the culture medium was removed, and cells were washed with cold PBS twice. Finally, 200 μl lysis buffer was added (Halt Phosphatase Inhibitor Cocktail and Halt Protease Inhibitor Cocktail EDTA free, 1:100 in M-PER Mammalian Extraction Buffer) and the lysate incubated on ice for 15 min. The protein concentration was measured by BCA assay after removal of the cell debris by centrifugation. Then the lysate was added to the PamChip arrays and the tyrosine kinase assay was performed according to the manufacturer’s protocol (n=3).

### Detection of macrophage marker CD206

Glioblastoma cells were seeded at 75% confluence, media was changed on the second day and conditioned media was collected after incubating for 48 hours. Human monocytes were isolated from buffy coats [7] and conditioned media was added with fresh media at a ratio of 1:1 on day 2 and 4 during 6 days of monocyte culture. Cells were collected and stained with CD206 antibody for FACS analysis. The experiments were repeated independently for three times.

### Co-immunoprecipitation and subsequent mass spectrometry analysis

The dynabeads^TM^ magnetic beads (10003D, Invitrogen) were incubated with 10 μg normal mouse IgG (CS200621, Millipore) or anti-SFRP2 (sc-365524, Santa Cruz) at room temperature for 30 minutes and washed twice in PBS with Tween™ 20. Then the magnetic bead-Ab complexes were incubated with cell lysates at 4°C overnight. In the end, the magnetic bead-Ab-Ag complexes were washed and eluted in Elution buffer (Glycine HCL elution buffer). A fraction was separated by SDS-page and analyzed by western blot, and the remainder was subsequently subjected to mass-spectrometry analysis. Each sample was separated using a Thermo Scientific Dionex nano LC-system in a 3 hour 5-40% ACN gradient coupled to Thermo Scientific High Field QExactive. The software Proteome Discoverer vs. 1.4 including Sequest-Percolator for improved identification was used to search the human Uniprot database for protein identification, limited to a false discovery rate of 1%. To identify the potential SFRP2 interacting partners (Table S4) all proteins that were identified in the IgG-background fraction were removed, subsequently proteins with less than 5 unique peptide hits were filtered out, and the remaining identified proteins were arranged according to their area score. The top-50 of these proteins were subjected to gene set enrichment analysis for ontology gene sets at MSigDB (www.gsea-msigdb.org/gsea/msigdb/) [8, 9] (Table S5).

## Supplemental References

1 Subramanian A, Narayan R, Corsello SM, Peck DD, Natoli TE, Lu X *et al*. A Next Generation Connectivity Map: L1000 Platform and the First 1,000,000 Profiles. *Cell* 2017; 171: 1437-1452 e1417.

2 Lamb J, Crawford ED, Peck D, Modell JW, Blat IC, Wrobel MJ *et al*. The Connectivity Map: using gene-expression signatures to connect small molecules, genes, and disease. *Science* 2006; 313: 1929-1935.

3 Rual JF, Venkatesan K, Hao T, Hirozane-Kishikawa T, Dricot A, Li N *et al*. Towards a proteome-scale map of the human protein-protein interaction network. *Nature* 2005; 437: 1173-1178.

4 Hagerstrand D, Tong A, Schumacher SE, Ilic N, Shen RR, Cheung HW *et al*. Systematic interrogation of 3q26 identifies TLOC1 and SKIL as cancer drivers. *Cancer Discov* 2013; 3: 1044-1057.

5 Wang Q, Hu B, Hu X, Kim H, Squatrito M, Scarpace L *et al*. Tumor Evolution of Glioma-Intrinsic Gene Expression Subtypes Associates with Immunological Changes in the Microenvironment. *Cancer Cell* 2017; 32: 42-56 e46.

6 Hu Y, Smyth GK. ELDA: extreme limiting dilution analysis for comparing depleted and enriched populations in stem cell and other assays. *J Immunol Methods* 2009; 347: 70-78.

7 Mia S, Warnecke A, Zhang XM, Malmstrom V, Harris RA. An optimized protocol for human M2 macrophages using M-CSF and IL-4/IL-10/TGF-beta yields a dominant immunosuppressive phenotype. *Scand J Immunol* 2014; 79: 305-314.

8 Subramanian A, Tamayo P, Mootha VK, Mukherjee S, Ebert BL, Gillette MA *et al*. Gene set enrichment analysis: a knowledge-based approach for interpreting genome-wide expression profiles. *Proc Natl Acad Sci U S A* 2005; 102: 15545-15550.

9 Liberzon A, Subramanian A, Pinchback R, Thorvaldsdottir H, Tamayo P, Mesirov JP. Molecular signatures database (MSigDB) 3.0. *Bioinformatics* 2011; 27: 1739-1740.

10 Parsons DW, Jones S, Zhang X, Lin JC, Leary RJ, Angenendt P *et al*. An integrated genomic analysis of human glioblastoma multiforme. *Science* 2008; 321: 1807-1812.

## Supplemental Figure legends

### Supplemental Figure 1. The distribution of type A and type B genes in CCLE glioma cell lines and TCGA glioblastoma datasets

**A** and **B**. Data is displayed in a similar fashion as in Fig.1B where the heatmaps illustrate gene expression patterns of type A and type B genes in CCLE glioma cell lines (**A**) and TCGA glioblastoma samples (**B**). The cell lines were firstly divided into low and high *FN1* expression groups. Secondly, the *FN1* low or high expression groups were arranged according to high to low *GFAP* expression. The genes were ranked according by their correlation to the *FN1* expression signature. The red to blue colors indicate relatively high to low expression levels. The black and white boxes indicate type A and type B genes.

### Supplemental Figure 2. Effects of SFRP2 and SOX2 on expression levels of cell cycle related genes and on cell cycle phase

**A**. Changes in cyclin, cyclin-dependent kinase, and cyclin-dependent kinase inhibitor gene expression levels determined by RNA-sequencing upon SFRP2- or SOX2-overexpression in U-2987 and U-2982, respectively. The genes are listed according to their observed higher-to-lower gene expression in U-2987 (black columns). The CDKN2A (p16/p14ARF locus) is strongly up-regulated by SFRP2 in U-2987, whereas it is not expressed at all in U-2982 and not induced by overexpressed SOX2, indicating that U-2982 lacks this key glioma susceptibility locus [10]. Whereas CDKN1A (p21Cip) and CDKN2B (p15) are similarly up-regulated, by SFRP2 in U-2987 and by SOX2 in U-2982, the cyclin-dependent kinase inhibitor 1B (CDKN1B; p27(KIP)) is reversely regulated. P27 is relatively decreased by SFRP2 in U-2987 and increased by SOX2 in U-2982 and is therefore interesting as potentially involved in the proneural-to-mesenchymal transition. **B**. Heatmap illustrating relative gene expression levels of selected cyclins, cyclin-dependent kinases, and cyclin-dependent kinase inhibitors determined by RNA-sequencing upon SFRP2- or SOX2-overexpression in U-2987 and U-2982, respectively. **C**. Western blot analysis of protein levels of SOX2, and cyclins A, B, D, and E upon SFRP2- or SOX2-overexpression in U-2987 and U-2982, respectively. GAPDH was added to assess protein input levels. **D**. Cell cycle phase analysis upon SFRP2-overexpression in U-2987 as determined by nuclear DAPI staining and FACS analysis. **E**. The same as in panel D, but for U-2982 upon SOX2-overexpression.

###
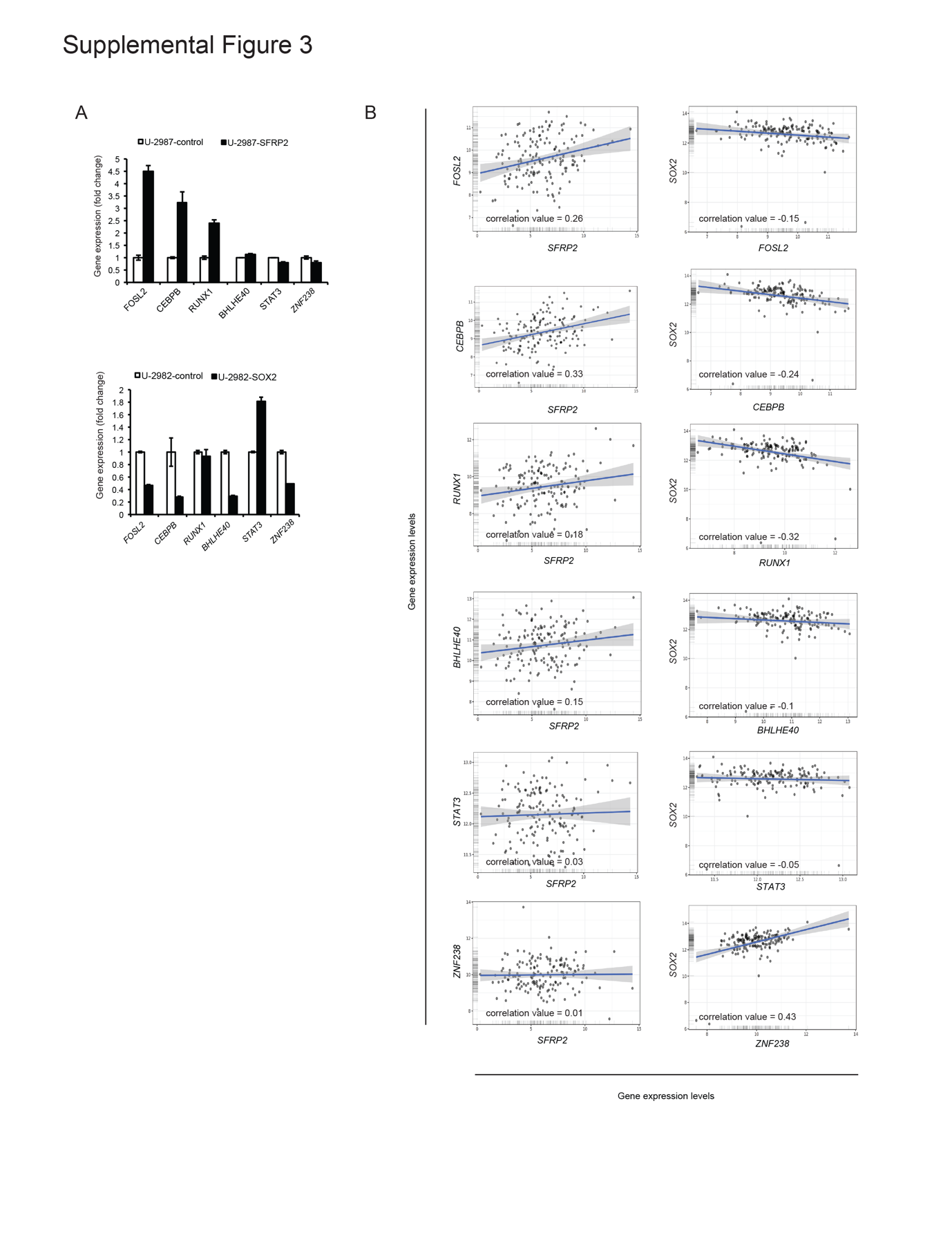


### Supplemental Figure 3. The correlation of SFRP2 or SOX2 with other genes in TCGA glioblastomas

**A**. Relative expression levels (fold of control) of *STAT3*, *CEBPB*, *BHLHE40*, *RUNX1*, *FOSL2* and *ZNF238* upon SFRP2-overexpression in U-2987 (upper panel) and SOX2-overexpression in U-2982 (lower panel). **B**. The correlation rate of *SFRP2* and *SOX2* with *STAT3*, *BHLHE40*, *RUNX1*, *FOSL2*, and *ZNF238* according to RNA-sequencing expression data in TCGA_GBM. These analyses were performed by GlioVis analysis.

### Supplemental Figure 4. Identification of transcription factors potentially involved between SFRP2 and SOX2 signaling events

Heatmap showing 65 *SOX2* positively (Pearson’s correlation, r > 0.3) and 18 negatively (r < -0.3) correlated genes to the *SOX2* expression pattern in 45 CCLE glioma cell lines. The bar graph in the right depicts fold-changes of gene expression upon SFRP2- (gray bars) or SOX2- (black bars) overexpression. *POU3F2* and *KLF4* are marked with star as the two transcription factors that fit the requirements of respectively being positively correlated to *SOX2* expression but do not increase upon SOX2 overexpression but decrease upon SFRP2 overexpression, or being negatively correlated to *SOX2* expression but do not decrease upon SOX2 overexpression but increase upon SFRP2 overexpression.

## Supplemental Table legends (See more information in the attached Excel)

Supplemental Table 1. Open reading frame (ORF) cDNA constructs retrieved from the Orfeome library. All genes in Table 1 were queried in the Orfeome database and 9 out of 15 type A genes and 17 of the 30 type B genes were available. The Orfeome clones were retrieved from the Karolinska High Throughput Center in Huddinge. Genes that were possible to retrive and subsequently clone into a viral vector are labeled with blue text and denoted in column 4.

Supplemental Table 2. Two unbiased classifications in CCLE and HGCC databases. The genes in CCLE or HGCC cell cultures were arranged according to STDEV from high to low among all of the samples and the first 300 genes were picked up and used to divide all samples into two groups. The top 100 genes in CCLE Group1 (C1) and Group 2 (C2) were named C1 and C2 genes. The top 100 genes in HGCC Group1 (H1) and Group 2 (H2) were named H1 and H2 genes.

Supplemental Table 3. Identification of altered phosphopeptide levels by PamGene analysis. PamGene data was retrieved from the PamGene station. The first column indicates the spotted peptides for corresponding phospho-sites in the denominated proteins. The value in columns 2 to 10 show normalized relative peptide phosphorylation levels for triplicate measurements of cell lysates derived from U-2982-control, U-2982-SOX2 and U-2987-control. High value indicates detection of high levels of phosphorylated tyrosine residue for the corresponding peptides.

### Supplemental Table 4. Proteins identified by mass spectrometry analysis of SFRP2 co-immunoprecipitated proteins. SFRP2 is highlighted in orange.

### Supplemental Table 5. Ontology enrichment analysis of top 50 identified proteins. Enrichment analysis was performed at the msigDB portal among ontology gene sets ([www.gsea-msigdb.org/gsea/msigdb](http://www.gsea-msigdb.org/gsea/msigdb)).

Supplemental Table 6. List of genes that increased or decreased upon SOX2 overexpression in U-2982 or SFRP2 overexpression in U-2987 or were differentially expressed between U-2982 and U-2987. The first and second column sets show 2444 and 761 genes that were significantly increased or decreased by SOX2 overexpression in U-2982 (>2-fold, p<0.05), respectively. The third and fourth column sets show 1212 and 970 genes significantly increased or decreased by SFRP2 overexpression in U-2987 respectively (>2-fold, p<0.05). The fifth and sixth column sets show 3412 and 1145 genes with significantly different expression levels in U-2987 and U-2982 respectively (>2-fold, p<0.05). The fold change in expression was calculated by comparing the average value from triplicates, and p-values were calculated by t-test of triplicates.

Supplemental Table 7. Comparison of SFRP2 and SOX2 regulated genes with spatial gene expression profiles from glioblastoma tissue. A. The first column lists genes from the spatial gene expression data that positively correlated with SFRP2 expression with r>0.5. The second column describes the correlation to SFRP2 (r value). The third and fourth columns show the fold change value of the respective genes upon SFRP2 overexpression in U-2987 or SOX2 overexpression in U-2982. B. The same as for A, but with regard to SOX2. C. The first column lists genes from the spatial gene expression data higher expressed in VA areas than CT areas. Other two columns are as in column set A. D. The first column lists genes from the spatial gene expression data higher expressed in CT areas than in VA areas. Other two columns are as in column set A.

Supplemental Table 8. STR profiling of U-2982, U-2987, and their SOX2 or SFRP2 overexpressing derivatives by AmpFISTR Identifiler PCR amplification Kit (Thermo Fisher). The analysis confirms that U-2982-control and U-2982-SOX2, and U-2987-control and U-2987-SFRP2 are more than 96% related, thus confirming no mixing up of cells has occurred during generation of the cultures. Inconsistencies were found for 3 alleles denoted with * of the 16 analyzed markers in U-2982 and 1 allele denoted with ** for U-2987.

Supplemental Table 9. Table of included cell lines from CCLE***.*** First column denotes full name used in CCLE gene expression data matrix. Second column denotes cell line name according to CCLE. Column 3 denotes if cell line was selected and included in figure 1 and subsequent analyses. The two following columns indicated the order of appearance of the cell lines in figure 1 from left to right and if they were clustered into cluster C1 or C2. The two last columns indicate the mentioned histological origin of each cell lines and subsequent histological subtype.

### Supplemental Table 10. List of antibodies and qPCR primers.
