# Supplementary material for: SFRP2 induces a mesenchymal subtype transition by suppression of SOX2 in glioblastoma
Source: Oncogene. 2021 May 21;40(32):5066–80. doi: 10.1038/s41388-021-01825-2 (PMC8363098; doi:10.1038/s41388-021-01825-2)
Supplement: Supplementary file 2 — Supplemental tables [file 41388_2021_1825_MOESM2_ESM.pdf]

Supplemental Table 1. Open reading frame (ORF) cDNA constructs retrieved from the Orfeome library. All genes in Table 1 were queried in the Orfeome database and 9 out of 15 type A genes and 17 of the 30 type B genes were available. The Orfeome clones were retrieved from the Karolinska High Throughput Center in Huddinge. Genes that were possible to retrive and subsequently clone into a viral vector are labeled with blue text and denoted in column 4.

GBM culture subtypes	Gene symbol	Name	Present in Orfeome
Type A genes	NDP	Norrie disease (pseudoglioma)	Yes
	GFAP	glial fibrillary acidic protein	Yes
	VANL1	visinin-like 1 (VSNL1)	Yes
	CXCR4	chemokine (C-X-C motif) receptor 4	Yes
	CXCL14	chemokine (C-X-C motif) ligand 14	Yes
	GPM6B	glycoprotein M6B	Yes
	SRPX	sushi-repeat containing protein, X-linked	Yes
	TRIM9	tripartite motif containing 9	Yes
	SOX2	SRY (sex determining region Y)-box 2	Yes
	SOX9	SRY-Box 9	No
	SLC1A3	Solute Carrier Family 1 Member 3	No
	KCNH2	Potassium Voltage-Gated Channel Subfamily H Member 2	No
	CD70	CD70 Molecule	No
	CTNND2	Catenin Delta 2	No
	PTN	Pleiotrophin	No
Type B genes	DCN	Decorin	Yes
	CNP	2',3'-Cyclic-nucleotide 3'-phosphodiesterase	Yes
	NID2	nidogen 2 (osteonidogen)	Yes
	HK1	hexokinase 1	Yes
	KDELR2	KDEL Endoplasmic Reticulum Protein Retention Receptor 2	Yes
	CDK2AP1	Cyclin-dependent kinase 2 associated protein 1	Yes
	NMU	Neuromedin U	Yes
	SFRP2	Secreted frizzled-related protein 2	Yes
	KLF4	Kruppel-like factor 4 (gut)	Yes
	COL1A1	collagen, type I, alpha 1	Yes
	PDGFRB	platelet-derived growth factor receptor, beta polypeptide	Yes
	LAMC1	laminin, gamma 1 (formerly LAMB2) (LAMC1)	Yes
	FABP5	fatty acid binding protein 5 (psoriasis-associated)	Yes
	WNT5A	wingless-type MMTV integration site family, member 5A	Yes
	RGS5	regulator of G-protein signaling 5	Yes
	GREM1	gremlin 1, DAN family BMP antagonist	Yes
	GRB14	growth factor receptor-bound protein 14	Yes
	PCOLCE	procollagen C-endopeptidase enhancer	No
	ISLR	Immunoglobulin superfamily containing leucine-rich repeat	No
	IGFBP7	Insulin Like Growth Factor Binding Protein 7	No
	NR2F1	Nuclear Receptor Subfamily 2 Group F Member 1	No
	ATP5G3	ATP Synthase Membrane Subunit C Locus 3	No
	PLAGL1	PLAG1 Like Zinc Finger 1	No
	FOXF2	Forkhead Box F2	No
	FBLN2	Fibulin 2	No
	FN1	Fibronectin 1	No
	IGFBP3	Insulin Like Growth Factor Binding Protein 3	No
	COL6A3	Collagen Type VI Alpha 3 Chain	No
	CXCL12	C-X-C Motif Chemokine Ligand 12, Stromal Derived Factor 1 (SDF1)	No
	NDN	Necdin, MAGE Family Member	No

Supplemental Table 2. Two unbiased classifications in CCLE and HGCC databases. The genes in CCLE or HGCC cell cultures were arranged according to STDEV from high to low among all of the samples and the first 300 genes were picked up and used to divide all samples into two groups. The top 100 genes in CCLE Group1 (C1) and Group 2 were named C1 and C2 genes. The top 100 genes in HGCC Group1 (H1) and Group 2 were named H1 and H2 genes.

	CCLE Group 1 (C1)																					CCLE Group 2 (C2)																							
Description	U87MG	AM38	SNU489	SNU1105	YH13	DKMG	42MGBA	LN443	U178	U138MG	U118MG	BECKER	KG1C	CAS1	YKG1	SF295	U343	LN18	CFSTTG	LN382	T98G	A172	LN428	SF172	8MGBA	GMS10	LN215	M059K	KNS60	LNZ308	KS1	LN464	SNU466	LN229	LN235	LN340	STRG05M	GB1	TM31	SNU201	SNB19	U251MG	KNS81	SNU626	KNS42
COL11A	3,8	3,9	5,6	8,0	3,8	12,1	3,8	3,6	4,0	3,9	4,1	10,7	3,7	10,0	7,2	6,7	3,7	6,9	3,6	7,8	3,7	6,7	6,1	3,7	4,0	9,9	9,6	4,6	3,6	12,2	11,6	10,3	13,0	11,6	12,4	11,4	11,6	11,8	11,4	11,0	10,6	11,3	12,6	3,6	11,6
LIMCH1	5,1	4,1	4,0	7,7	3,7	5,1	4,4	3,9	3,7	4,1	5,9	5,3	7,4	8,0	8,8	6,6	8,9	9,1	9,5	8,7	10,6	9,0	10,5	8,0	7,4	8,5	9,7	9,4	9,0	11,2	8,6	7,2	11,9	10,1	8,9	8,4	9,3	9,7	8,5	10,5	10,8	10,7	10,0	10,4	7,5
MYO5C	4,8	4,4	6,1	4,7	5,0	4,7	5,4	3,9	4,5	4,7	4,8	4,9	4,2	4,9	7,3	4,5	4,9	6,3	4,8	4,7	4,7	5,3	5,5	9,1	9,7	10,3	10,0	6,5	5,3	4,7	8,3	4,8	6,0	5,3	4,7	4,0	8,5	6,2	8,7	8,1	6,9	8,5	10,4	10,7	10,4
GPM6B	4,1	4,2	3,9	5,8	4,7	7,4	4,2	4,4	3,9	4,1	3,8	4,5	4,2	5,6	4,8	4,0	3,9	4,0	4,2	3,9	3,9	4,0	4,1	4,0	5,0	4,2	4,1	4,3	4,2	8,8	4,0	8,4	11,5	7,2	5,9	4,8	9,6	10,5	11,6	9,6	9,8	12,7	10,0	10,2	12,4
ELOVL7	8,3	8,2	7,9	7,2	4,8	4,3	4,2	3,9	9,4	4,1	4,9	3,5	7,3	4,3	5,6	4,0	4,3	4,3	3,9	3,9	4,0	5,7	8,9	9,3	7,7	8,9	9,6	9,1	8,7	5,7	9,3	6,3	4,7	6,7	7,1	4,0	9,1	3,6	5,9	5,7	8,3	7,5	8,6	5,3	6,1
EFEMP1	7,7	6,7	10,3	10,7	7,5	10,6	5,1	4,3	4,5	4,5	4,0	8,3	12,0	7,5	4,8	5,6	11,5	6,9	11,2	9,5	8,4	4,7	10,7	11,9	10,2	10,0	11,4	7,3	13,0	11,2	11,2	4,2	11,1	12,4	12,4	9,5	13,1	12,5	12,7	12,2	12,8	12,3	12,5	11,8	7,3
CENPV	3,7	4,0	5,0	4,0	6,0	6,5	5,4	4,5	3,8	3,9	4,8	3,9	4,0	8,7	9,8	7,6	7,0	10,2	7,0	4,0	7,4	8,5	9,1	10,0	8,3	9,9	9,4	8,7	8,3	8,3	10,0	4,6	5,6	4,0	9,3	6,3	8,3	3,8	3,8	7,1	4,1	4,0	9,6	8,2	9,6
CRYAB	4,9	5,0	5,0	9,5	6,7	11,7	4,6	7,0	6,5	10,2	6,9	5,5	4,8	6,0	4,9	5,1	5,0	4,6	9,6	5,0	5,0	8,7	4,6	4,5	4,5	7,2	10,9	9,6	6,1	4,8	7,7	8,6	11,9	6,1	13,2	9,9	9,0	8,5	6,7	12,6	11,5	12,0	11,1	12,1	12,1
SOX2	4,1	4,4	3,9	7,1	4,3	5,4	4,1	4,1	3,9	3,9	3,9	5,7	7,7	4,6	4,7	7,0	4,6	4,0	8,4	4,1	4,1	4,7	4,0	4,0	4,2	3,9	4,7	3,8	5,3	8,0	4,0	4,1	6,5	8,6	7,1	9,1	8,2	7,5	8,3	10,0	10,7	10,7	9,6	9,5	11,8
PTPRZ1	3,8	4,1	3,7	4,0	4,0	6,3	3,9	3,8	3,7	3,7	3,6	4,3	7,9	3,9	3,7	3,9	4,5	3,6	4,0	3,8	3,8	3,8	3,7	3,6	3,8	3,9	3,7	3,6	3,7	3,6	3,9	8,1	12,3	10,8	7,6	8,4	7,7	3,5	6,1	7,4	7,3	11,9	8,4	7,9	10,6
CPVL	4,3	4,4	7,4	5,7	4,1	4,2	10,5	5,9	4,3	3,9	3,9	6,8	4,0	8,8	9,0	3,8	6,1	8,4	4,3	5,2	3,7	6,6	9,6	5,9	10,1	7,3	11,1	9,4	9,7	9,7	3,8	10,0	3,9	4,4	6,6	4,3	5,2	8,3	4,0	7,1	7,3	5,6	9,1	8,2	4,2
GPC4	4,4	4,5	9,2	9,2	4,5	10,0	6,4	5,6	4,3	6,3	7,4	6,5	7,5	6,3	4,6	4,3	9,2	5,5	5,6	6,4	5,7	6,0	6,5	4,2	7,2	8,7	8,5	6,7	9,4	9,9	4,6	4,3	6,4	5,4	8,9	7,7	10,2	11,9	10,7	9,5	9,4	9,6	10,1	10,4	10,6
MOXD1	9,0	7,4	10,4	10,2	10,6	8,0	4,6	5,1	5,0	10,1	7,2	6,2	4,5	9,7	9,1	5,1	5,8	6,0	5,3	5,3	4,9	9,2	10,2	4,2	7,5	8,5	7,4	10,4	8,8	4,6	4,4	10,9	9,5	8,1	8,3	6,8	10,5	8,7	7,3	9,3	10,5	10,8	11,8	9,6	8,9
FLRT3	4,7	7,5	5,1	7,0	4,3	4,5	4,5	5,8	4,6	4,3	4,6	6,6	9,6	5,7	7,4	4,4	4,6	4,7	4,7	4,0	4,4	6,6	4,3	4,4	7,4	5,2	4,1	4,3	5,5	6,2	6,4	9,6	12,1	12,1	9,8	8,5	7,5	9,3	10,2	8,9	8,8	7,9	5,7	4,5	4,6
ANKRD	4,1	10,0	3,6	5,1	3,8	4,0	3,7	3,6	4,6	3,6	3,6	3,7	4,1	3,8	4,0	3,8	3,9	3,8	4,1	3,6	3,7	7,7	3,9	11,0	4,4	7,8	5,6	9,1	7,0	4,2	8,5	3,8	4,1	4,0	3,9	3,7	8,4	6,5	10,5	5,5	9,5	4,8	6,3	4,9	6,3
NES	6,3	9,0	4,6	6,4	5,7	7,6	5,9	4,7	4,4	4,8	5,5	9,5	10,1	8,7	9,2	8,4	4,5	4,4	8,1	7,1	4,2	10,9	5,2	5,2	5,4	7,0	5,3	4,4	4,7	9,6	8,9	4,6	11,0	11,1	9,2	8,5	8,7	9,3	11,4	9,5	9,3	10,1	8,7	7,8	7,0
CHRDL1	5,9	10,3	8,4	7,6	4,2	7,0	4,3	8,8	4,2	10,9	5,8	8,1	4,1	4,7	3,9	4,3	4,8	4,2	4,4	5,4	4,6	4,1	10,5	4,7	8,5	11,2	10,6	9,2	9,6	4,5	4,2	4,2	4,4	9,2	5,6	7,3	9,3	10,1	4,1	10,9	4,4	4,6	12,4	8,1	4,6
C5orf46	5,0	5,3	5,3	12,1	5,3	5,6	5,1	5,1	6,0	4,8	4,7	9,0	6,8	5,3	6,3	5,6	12,0	4,9	5,3	5,3	5,4	6,7	7,4	5,1	4,7	7,1	6,5	10,8	5,6	11,1	9,4	5,1	5,8	8,0	10,8	9,1	10,5	8,8	11,0	10,2	10,1	9,8	6,7	7,0	6,5
OCIAD2	11,4	7,3	10,4	9,5	9,8	6,7	4,9	5,2	5,0	4,7	4,5	8,8	4,5	10,1	8,9	11,0	9,8	4,7	7,5	8,0	7,7	11,3	9,9	10,7	9,2	4,6	11,1	9,6	11,1	10,7	10,0	5,2	4,9	4,6	10,1	10,5	12,1	4,7	12,0	10,9	11,9	12,1	11,4	4,5	11,1
SNX10	3,6	5,4	6,8	6,2	3,5	3,5	9,0	4,2	3,2	3,5	3,3	8,6	9,7	4,2	7,3	7,8	8,3	8,3	5,8	3,6	3,8	8,1	7,7	9,6	7,9	6,7	9,8	5,5	9,9	3,5	3,3	10,9	4,4	9,9	7,6	6,4	8,8	3,2	9,2	3,3	9,5	10,1	9,8	9,1	5,4
DHRS3	8,9	7,8	6,2	5,8	3,9	4,8	5,6	4,4	4,3	4,5	3,9	7,7	7,5	6,5	4,5	10,0	8,4	4,2	5,6	4,6	6,3	4,8	10,5	5,6	5,8	7,6	5,7	4,8	6,9	10,3	7,9	10,6	9,9	11,3	8,3	4,9	9,7	8,7	9,7	7,6	8,4	10,9	6,4	5,2	6,7
POU3F2	4,0	5,6	4,3	5,2	3,9	4,8	4,0	4,5	4,4	4,2	3,8	4,5	5,6	4,2	9,8	4,0	4,2	4,0	5,7	3,9	4,1	4,8	9,2	4,1	3,9	4,2	4,2	5,9	5,2	4,1	4,8	7,7	7,3	6,0	3,9	5,7	7,1	7,5	10,6	6,2	9,1	9,4	8,5	9,3	10,0
TUBB2B	3,9	4,3	5,9	8,6	4,8	5,5	8,8	3,9	4,8	3,7	3,7	10,3	7,8	9,9	11,5	7,1	5,2	5,3	10,4	4,2	6,4	5,8	5,6	8,3	9,5	9,1	4,2	7,1	6,2	6,4	5,8	6,2	7,6	10,7	9,0	7,2	7,7	10,4	11,6	9,4	8,6	10,2	11,7	12,1	5,9
RPL39L	7,8	3,6	3,4	4,2	7,8	7,1	3,7	3,4	6,3	3,4	4,3	7,9	9,1	3,8	7,5	7,3	8,0	3,7	9,2	4,2	5,0	9,8	5,7	10,0	4,1	7,7	8,9	8,2	9,5	8,1	9,2	9,9	8,1	3,7	5,4	8,1	7,1	8,0	3,6	4,1	3,7	5,1	3,9	4,2	3,9
FBN2	7,3	9,6	7,9	4,4	9,3	9,9	3,9	3,8	4,1	8,8	5,6	3,7	6,6	4,1	4,3	3,7	3,7	4,0	3,9	4,1	6,7	8,2	3,8	3,4	6,6	8,2	9,9	8,8	8,1	11,6	10,5	7,1	7,4	6,6	3,9	3,9	4,1	3,9	3,7	4,8	7,4	5,9	7,0	12,1	3,9
CTSZ	4,2	4,3	6,8	4,2	7,6	7,5	6,6	4,8	3,9	3,8	3,7	6,8	3,7	4,1	4,3	3,8	6,3	3,9	3,6	4,3	8,6	8,2	8,9	8,8	6,5	6,1	10,3	5,7	8,0	3,4	3,9	9,2	4,0	4,5	8,3	4,1	10,6	8,2	9,7	6,1	4,1	4,1	5,9	7,7	3,9
IRX1	4,0	3,5	3,7	5,6	3,7	3,3	3,7	3,6	3,7	4,3	5,6	4,0	3,5	3,6	5,7	7,1	6,3	3,5	3,7	3,5	9,7	5,5	6,6	3,7	3,4	3,5	6,0	3,9	6,3	9,8	3,6	3,7	3,7	6,9	5,2	9,0	7,4	5,4	3,6	8,0	10,0	10,5	6,4	5,8	3,5
MGP	5,9	3,8	7,6	4,1	8,6	10,2	3,7	4,3	3,8	3,7	3,7	8,2	5,9	10,2	8,6	8,7	12,1	5,4	4,5	10,2	9,2	4,2	4,1	6,3	12,4	10,1	11,0	5,2	4,4	3,8	5,4	8,8	9,9	8,9	13,0	11,9	9,8	5,4	4,0	11,8	4,6	9,2	9,8	10,0	3,7
HOXB2	10,7	9,8	10,9	10,6	6,1	8,9	9,8	6,5	8,8	6,1	6,1	6,5	6,0	6,8	12,8	11,1	10,6	5,8	6,3	6,1	6,7	11,4	9,1	10,6	5,6	8,5	7,3	9,8	10,9	5,6	7,2	10,9	6,5	6,8	10,9	10,0	11,0	6,3	7,3	8,7	5,7	6,5	12,0	9,3	10,5
BCHE	7,0	8,3	6,9	6,5	8,5	9,9	3,8	5,1	9,5	8,6	9,7	8,1	9,5	4,5	9,5	4,1	4,3	5,3	3,9	3,6	3,8	8,7	9,8	3,7	4,2	7,0	7,1	10,6	3,9	9,4	10,9	11,0	7,8	9,5	9,5	6,1	7,7	6,2	10,0	9,4	9,5	8,9	9,5	5,3	9,5
ODZ2	6,9	11,9	13,1	12,9	8,0	9,3	12,4	12,4	12,8	8,8	4,5	9,4	8,4	7,8	8,5	5,1	4,7	9,5	5,1	4,1	7,4	7,5	11,2	8,1	9,9	11,7	11,3	12,7	12,6	11,9	4,1	4,0	4,4	6,7	9,7	8,0	11,6	11,4	12,6	12,2	12,3	12,4	13,5	11,8	11,3
EPSTI1	5,0	4,6	7,0	6,1	6,6	4,6	8,2	4,6	5,1	4,4	4,4	5,4	5,0	6,8	4,6	5,6	5,4	8,2	7,8	8,7	8,0	5,0	5,3	9,2	10,9	10,7	9,1	8,4	5,6	7,1	4,1	4,3	4,5	5,0	7,4	4,6	10,0	11,5	6,8	7,7	4,4	7,3	8,2	9,4	4,7
PODXL	8,9	10,3	5,6	6,6	4,2	5,9	6,6	10,7	9,7	10,2	10,9	8,3	8,4	5,2	7,8	9,3	7,3	8,9	4,3	9,7	6,0	13,2	10,2	12,0	9,0	10,2	12,3	8,9	9,6	11,1	4,4	5,7	10,4	10,7	7,1	7,5	7,2	12,8	11,8	10,1	9,7	10,3	10,0	11,6	11,9
IGFBP2	5,9	5,2	4,9	10,3	10,5	10,3	5,2	5,2	5,0	5,0	4,7	7,2	11,0	10,1	11,7	8,5	4,9	8,4	5,7	7,9	10,9	8,7	4,8	4,7	10,1	5,4	7,2	4,6	5,0	11,5	11,4	7,2	12,9	9,1	6,9	4,8	12,3	11,0	12,4	11,7	10,7	11,9	12,2	12,0	8,1
COL21A	4,5	4,4	5,2	4,1	8,5	11,5	4,5	3,9	4,0	5,9	4,0	8,4	4,4	6,8	11,2	5,3	4,8	5,9	4,9	5,9	4,2	4,9	7,3	4,2	8,0	4,1	4,0	4,5	4,0	8,0	4,1	3,9	4,3	4,5	8,2	11,3	7,1	7,5	4,1	8,4	7,7	9,4	8,2	10,5	6,4
CXCR7	4,2	6,1	9,7	9,0	8,7	4,6	6,8	4,4	10,7	6,9	5,1	7,1	4,2	6,1	4,2	4,3	8,7	4,9	7,8	10,7	8,4	4,2	8,4	3,8	9,0	5,1	9,4	6,8	9,8	10,9	9,8	3,7	10,7	6,7	10,2	10,3	4,1	6,1	4,3	9,9	9,2	5,9	10,0	10,3	5,3

100 highest genes in C1

[illegible]

Description	HGCC Group 1 (H1)																											HGCC Group 2 (H2)																			
Description	U3008	U3121	U3033	U3051	U3088	U3053	U3110	U3027	U3020	U3071	U3067	U3062	U3101	U3078	U3029	U3035	U3024	U3037	U3073	U3034	U3039	U3028	U3031	U3068	U3046	U3060	U3123	U3054	U3019	U3016	U3013	U3117	U3118	U3005	U3065	U3002	U3056	U3017	U3009	U3084	U3004	U3048	U3086	U3042	U3021	U3082	U3085
BCAN	10,6181	7,70924	8,07723	7,95496	9,15723	8,42939	8,09303	9,67736	9,71965	7,73463	7,47218	8,26734	7,65912	7,54963	8,04284	8,10405	7,46729	7,54533	7,62602	7,2147	8,13011	8,90368	7,82094	7,45638	7,45181	7,57758	9,25456	7,62867	11,0829	11,9187	10,6347	10,5872	10,668	9,73522	10,1974	11,4945	11,2423	10,934	11,4106	11,5868	11,2894	11,5995	11,9612	12,2445	11,1851	11,2777	12,2895
KCND2	7,2	6,2	5,8	5,2	8,0	7,4	5,4	6,7	5,7	5,3	6,3	6,9	5,3	5,1	6,3	5,6	4,9	4,9	6,1	4,9	6,3	5,6	5,7	4,9	5,1	5,5	7,8	5,1	6,7	6,4	6,0	10,6	10,6	6,7	6,6	6,5	8,3	9,6	10,9	9,0	9,8	8,1	10,2	11,1	9,3	11,1	11,3
LPAR4	8,1	7,1	9,9	5,7	8,7	7,4	7,4	9,5	6,2	8,5	6,9	5,9	7,5	8,8	7,7	6,2	6,4	6,4	7,4	5,7	7,0	6,1	7,0	9,1	6,0	7,1	8,5	10,4	9,2	9,9	10,9	9,9	10,0	10,0	9,8	7,9	8,3	10,7	6,1	10,4	10,1	9,3	8,4	10,7	11,0	10,5	9,7
GPR19	10,2	8,0	8,4	7,8	8,9	7,8	7,1	8,7	6,4	8,6	5,2	7,3	6,3	8,1	5,3	7,6	7,2	6,9	6,6	7,3	7,6	8,9	8,2	8,7	8,5	9,2	8,6	7,7	10,1	10,2	10,8	9,8	10,3	10,3	9,6	9,7	10,2	9,6	11,1	10,2	10,1	8,1	10,8	9,9	9,9	10,8	10,8
SCG3	6,7	5,7	4,4	5,5	5,7	6,6	4,5	7,5	5,4	4,3	3,9	5,5	4,1	4,7	5,0	4,1	4,1	3,8	4,5	4,0	5,0	5,5	7,3	4,1	5,2	4,4	6,0	4,0	6,8	6,9	7,4	7,7	8,1	7,3	5,6	8,2	8,4	6,9	6,7	8,5	8,1	7,8	8,7	7,7	6,1	9,6	8,5
DCX	8,5	8,8	9,4	6,5	7,3	10,1	8,1	9,4	7,9	8,0	6,1	7,4	6,1	7,5	7,2	8,1	6,3	6,5	6,3	6,3	9,9	10,7	8,7	6,1	6,4	6,4	8,1	6,4	11,3	12,0	11,6	10,2	10,3	9,3	9,6	9,1	9,5	10,0	9,2	10,0	11,3	11,9	9,8	10,4	10,3	11,5	11,1
NCAM2	9,3	6,5	4,9	5,9	6,8	6,8	7,1	7,7	6,2	6,3	5,3	6,1	5,6	5,3	5,5	7,5	7,7	5,6	5,3	6,6	6,8	6,0	6,1	5,6	5,9	6,3	8,4	6,4	7,1	7,4	7,7	8,6	8,9	6,7	8,2	8,4	9,5	8,5	5,4	9,3	8,0	7,9	9,7	10,1	9,0	9,0	10,4
GRIK3	10,0	8,7	8,6	8,2	8,1	10,0	7,8	8,9	8,1	7,5	6,5	8,7	7,3	6,3	6,8	6,6	6,5	7,0	7,3	6,8	8,9	9,2	10,0	8,4	8,4	7,0	9,3	7,5	9,1	9,8	10,1	9,9	10,2	9,9	10,3	11,3	11,0	11,8	10,6	11,3	10,9	8,7	11,2	11,3	9,5	10,3	9,9
LHFPL3	10,8	5,9	8,9	10,0	7,7	10,9	8,7	8,6	8,7	6,6	6,5	8,0	6,7	6,1	7,2	8,8	6,8	7,4	7,3	6,3	10,4	9,9	9,7	7,4	6,8	8,2	6,3	6,9	8,9	9,1	9,0	10,5	10,7	10,0	11,3	9,4	10,6	12,1	10,4	11,9	11,5	9,8	11,9	12,2	9,7	12,3	11,9
KCNJ10	10,3	8,9	8,4	9,5	10,2	9,9	9,7	9,7	7,7	6,7	6,2	8,3	7,9	6,4	6,6	7,4	7,0	6,2	7,4	7,2	8,6	9,3	7,1	6,7	8,8	8,0	11,0	6,5	9,6	10,7	10,5	10,5	10,5	9,8	9,4	11,2	11,4	10,2	10,5	11,3	10,9	8,8	11,2	11,1	11,5	10,4	11,5
NNAT	6,6	11,6	5,6	6,1	5,5	6,2	9,9	6,1	6,2	6,3	6,5	11,6	6,0	9,3	6,4	6,2	9,3	8,3	7,7	8,3	6,3	11,3	8,9	6,5	5,8	11,4	12,1	6,7	11,5	11,4	12,0	11,9	11,6	6,4	6,0	10,8	6,1	10,1	11,3	8,1	12,0	6,1	6,6	11,5	13,0	12,5	12,3
HAPLN1	8,9	5,5	5,6	6,6	7,1	6,3	5,3	7,1	8,4	8,9	7,8	6,4	5,4	5,5	5,8	6,7	6,0	5,1	6,9	5,1	5,8	6,0	5,8	5,3	6,4	9,5	9,1	5,7	10,9	11,3	11,7	5,8	6,3	11,0	7,2	11,8	11,2	9,1	12,3	9,8	6,1	9,2	7,8	10,0	6,3	10,9	6,5
TSPAN7	10,5	9,2	7,8	11,2	10,7	9,7	8,7	10,2	11,4	11,4	6,4	9,2	5,0	9,3	8,8	10,6	10,0	10,4	9,3	8,6	7,8	9,8	9,2	6,7	9,0	8,6	11,6	9,2	10,3	10,8	9,9	11,9	12,0	11,7	11,7	11,3	11,4	11,0	12,0	12,3	10,3	11,9	12,4	12,4	10,8	12,6	12,7
C1orf61	11,5	9,7	8,4	5,8	6,9	8,3	7,4	8,8	8,4	8,2	5,3	9,9	7,0	4,9	6,3	5,6	6,3	4,9	5,7	4,8	9,6	10,7	8,0	5,4	8,0	7,8	8,2	5,2	8,4	7,9	8,6	10,3	10,0	9,5	8,7	11,6	11,7	11,3	10,8	11,5	11,9	11,2	11,8	12,2	10,1	11,2	11,4
GRIA2	9,3	5,9	8,9	9,2	6,9	8,8	7,0	8,9	6,8	5,6	4,1	6,9	4,6	4,5	6,6	5,5	4,3	6,2	5,2	7,5	8,3	5,2	9,1	6,1	8,1	5,8	8,1	5,5	6,9	7,0	8,4	9,0	9,2	7,3	9,9	9,8	9,9	10,5	7,4	10,4	10,5	9,5	10,6	10,8	9,1	11,2	10,1
PHYHIP	10,3	9,0	9,6	7,7	9,2	8,7	8,6	9,6	8,4	8,5	6,2	8,3	6,6	6,6	5,8	9,0	6,0	7,2	6,6	8,3	8,4	9,3	7,2	7,2	8,0	8,9	9,8	6,3	9,3	10,7	10,2	10,0	10,5	9,5	10,5	10,5	10,7	10,1	9,3	10,9	11,5	9,5	11,0	11,0	10,4	10,2	11,0
ADCYA	8,6	6,9	8,6	7,6	9,4	7,7	9,4	9,2	10,4	7,3	7,4	7,3	7,2	7,1	7,8	7,1	6,8	7,1	7,0	7,4	10,3	9,1	8,0	7,1	7,6	8,6	8,8	7,1	7,6	7,4	7,1	9,4	9,5	8,9	7,9	11,1	10,3	9,8	9,3	11,3	11,7	12,2	12,1	11,3	11,1	9,9	11,2
LGR5	5,9	6,3	9,1	5,1	9,0	6,8	5,9	6,5	5,0	5,7	11,0	10,5	7,3	6,1	5,7	6,1	5,7	5,4	5,2	5,2	6,2	6,1	5,8	5,8	4,9	5,1	6,6	5,3	7,4	8,2	8,3	9,1	9,5	10,3	7,4	7,9	8,4	8,4	7,9	7,7	7,6	6,4	9,1	6,1	9,3	9,0	10,0
MPPED2	7,4	6,3	5,3	5,5	6,3	7,0	5,5	6,0	6,6	6,2	5,8	7,0	5,6	5,2	5,6	5,6	5,2	5,2	5,3	5,2	9,8	7,9	6,8	5,9	7,5	6,9	7,3	5,7	5,6	5,6	5,4	7,8	8,4	10,3	7,3	6,7	7,6	8,3	10,4	8,8	8,9	10,8	11,2	10,0	9,2	8,2	8,0
TUBB4 /	9,5	9,1	6,7	9,3	7,3	9,9	7,5	7,6	7,9	8,5	7,0	8,2	6,8	10,8	7,1	9,3	10,8	9,5	9,2	7,6	6,4	10,1	9,4	10,6	7,3	7,5	8,1	11,1	12,0	12,6	11,7	11,2	11,0	10,0	10,7	9,0	6,9	9,5	6,1	9,4	10,5	10,1	9,3	8,6	9,6	12,0	11,5
CHRDL1	9,0	8,5	6,2	8,4	8,7	7,5	8,5	7,4	9,5	7,5	9,3	7,0	5,5	6,3	7,7	10,1	7,7	8,4	7,0	5,5	6,5	7,5	7,7	7,6	5,6	10,1	6,9	11,1	9,9	10,5	10,3	9,6	9,5	9,4	10,6	7,8	8,4	8,1	9,1	8,9	11,3	8,1	8,9	8,1	9,3	8,7	10,4
DPP6	10,3	8,1	7,6	10,1	10,9	6,7	9,4	9,8	8,4	7,1	6,3	8,4	8,6	6,9	9,8	7,9	10,8	7,0	7,8	9,1	9,4	8,1	8,9	6,5	9,3	9,2	10,5	7,8	8,3	7,1	7,5	9,7	9,9	10,6	10,5	10,7	11,4	11,2	10,0	10,9	10,7	9,8	11,0	11,5	11,4	10,9	10,6
ASCL1	10,5	9,1	11,4	7,8	10,1	10,3	8,4	9,9	7,0	7,1	5,8	9,1	9,2	5,9	8,4	6,9	6,6	5,7	6,1	9,6	10,6	10,0	9,1	5,4	6,4	8,9	10,0	5,9	10,3	9,7	10,1	10,2	10,6	10,1	9,7	11,2	11,4	11,1	9,3	11,2	11,8	10,7	11,1	12,0	11,9	11,5	11,3
CHST9	8,7	5,4	6,2	5,6	6,7	6,7	7,6	9,5	7,4	5,9	5,2	5,5	5,2	5,7	5,3	6,9	5,1	5,5	5,2	5,5	5,6	6,1	5,4	5,6	5,8	8,1	9,2	8,7	6,0	6,2	5,6	6,3	6,5	5,3	7,2	10,1	9,3	9,4	8,5	8,7	7,2	7,5	7,7	8,6	9,7	6,4	9,0
DNER	8,9	7,4	7,3	8,2	9,6	9,1	8,6	9,7	8,6	6,9	6,2	7,8	9,4	8,2	7,2	6,6	8,5	6,6	9,1	8,2	7,4	7,5	8,9	7,6	6,5	6,7	10,3	7,8	7,9	7,5	8,0	9,9	10,0	9,9	9,3	8,9	11,2	10,9	8,3	9,3	10,7	9,0	8,4	11,1	12,0	11,3	11,6
FIBIN	6,1	6,1	6,4	9,2	10,2	10,1	10,7	9,4	10,0	7,1	5,1	6,5	8,4	8,2	8,9	9,3	8,3	7,5	7,8	7,7	11,1	8,9	10,2	7,7	6,5	10,7	8,9	7,7	7,1	7,9	8,2	10,7	10,9	9,2	9,2	10,3	10,4	10,7	8,4	10,4	9,4	10,1	9,5	11,0	10,4	11,4	10,9
NCAN	10,3	8,3	7,6	10,4	9,7	11,4	7,3	11,4	10,9	9,4	6,9	9,2	7,1	7,2	7,9	6,9	7,1	7,1	7,0	6,8	10,4	10,9	9,4	8,9	7,6	10,2	12,0	7,1	11,1	9,6	10,2	11,1	10,8	8,9	9,5	11,0	8,6	9,4	7,3	11,8	10,7	12,1	11,8	11,9	9,9	12,1	12,0
CENPV	8,4	8,5	8,4	6,8	6,7	7,2	7,5	7,0	5,9	6,7	7,0	6,8	8,6	9,0	6,4	6,9	5,7	7,7	7,9	7,6	8,7	6,1	6,0	8,8	8,8	10,1	9,6	5,6	9,1	9,9	10,3	8,8	8,7	10,3	9,9	8,1	9,6	9,6	6,0	7,4	9,4	8,5	7,1	8,6	8,1	10,3	10,0
ERBB3	5,9	5,8	5,6	6,0	6,1	6,1	5,5	5,9	5,6	6,2	5,7	5,6	5,7	5,9	6,2	6,3	6,6	6,7	6,2	6,1	5,8	6,2	6,4	5,7	6,1	5,7	6,1	11,1	11,1	12,0	11,7	6,8	8,1	7,4	8,3	8,7	6,9	6,3	7,2	6,1	7,0	5,8	5,9	6,2	6,8	9,6	9,4
ADAMT	6,5	5,9	5,8	5,7	5,7	6,5	6,5	6,6	6,1	6,5	6,1	5,9	5,9	6,9	6,4	6,4	5,7	7,7	5,6	8,4	8,3	6,8	9,1	7,9	10,1	7,7	6,5	5,4	8,2	8,4	9,6	6,4	6,7	9,9	10,7	7,3	10,2	11,4	10,6	6,7	10,8	6,0	9,3	7,7	7,4	7,4	7,7
ELOVL2	9,6	10,2	8,7	5,8	8,6	10,9	9,4	9,4	8,4	8,6	6,3	9,9	8,0	6,8	6,8	7,8	5,9	5,6	5,3	5,4	10,9	10,1	11,0	9,0	8,4	8,8	9,5	8,0	10,8	12,2	11,8	8,2	8,5	8,9	8,8	11,7	11,2	12,2	11,0	11,9	11,8	11,0	12,3	12,4	7,3	6,4	6,1
TNFAIP(	6,6	6,4	7,0	6,5	8,1	6,6	7,3	7,8	7,9	10,2	8,2	8,1	7,6	5,9	8,6	7,5	7,8	6,5	7,5	9,5	8,3	8,0	9,5	7,4	9,1	6,1	8,9	12,1	8,6	10,7	10,1	8,8	9,4	10,6	10,8	9,5	10,6	11,0	10,7	6,9	5,8	7,5	10,4	7,7	7,3	8,8	7,9
MEOX2	10,2	8,5	8,3	7,5	11,2	9,7	9,5	10,0	9,8	9,7	8,8	7,8	7,9	7,8	8,8	8,5	9,2	8,3	7,3	7,7	12,1	9,6	10,7	7,8	7,7	9,9	11,2	12,5	7,9	8,0	7,8	7,5	8,1	10,8	10,8	8,1	10,4	12,2	10,8	10,8	12,0	11,2	11,0	11,5	7,4	7,6	10,6
FAM5C	8,0	6,1	4,9	5,0	4,9	5,9	7,1	9,3	5,0	5,3	4,8	5,0	5,0	4,5	5,2	7,9	5,3	5,3	6,0	7,4	7,5	4,9	7,3	4,6	5,5	4,5	8,0	4,7	5,3	5,0	4,7	8,2	8,8	4,8	7,1	4,7	6,7	10,4	4,8	6,2	8,1	8,6	6,1	10,3	7,8	9,0	9,7
LUZP2	7,6	4,0	4,2	3,9	4,4	4,4	5,0	5,8	4,3	4,1	4,1	6,0	4,2	4,1	4,0	4,0	3,9	4,1	3,9	4,1	4,1	4,1	5,5	3,5	4,4	4,3	7,1	3,6	4,2	3,8	3,6	6,4	5,7	4,5	5,5	3,8	8,2	8,7	4,1	6,4	3,8	5,3	6,3	4,9	7,3	7,0	9,5
HIST1H1	11,5	12,2	12,9	7,4	8,1	7,4	10,9	12,3	7,3	10,4	10,3	10,2	7,8	12,6	8,0	10,2	10,8	9,2	7,8	12,7	12,4	12,3	12,3	11,7	11,7	12,4	10,9	12,9	8,2	8,9	9,4	13,0	12,7	13,0	13,0	10,7	13,0	13,0	12,1	11,2	8,9	13,0	11,5	7,9	13,1	13,2	10,2
[illegible]																																															

100 highest genes in H2

[illegible]

	CXCL11	8,4	6,0	8,5	5,4	7,9	8,5	10,4	8,5	8,9	5,9	5,3	7,7	6,9	6,1	8,1	10,2	11,5	5,0	8,3	5,1	6,5	9,3	9,6	6,9	4,9	8,6	7,1	6,1	7,2	7,1	7,2	9,0	9,7	5,4	6,2	5,0	5,5	7,6	7,0	7,8	9,3	7,3	8,7	5,9	6,5	4,5	6,0
	RPS6KA	4,0	3,7	4,7	4,9	3,7	6,3	4,5	6,8	4,7	8,5	4,7	4,1	8,1	4,7	4,9	8,4	5,7	7,8	4,7	7,6	6,3	6,0	7,4	4,0	3,8	5,9	5,6	3,8	3,8	3,9	3,6	6,0	6,9	4,0	5,1	3,9	4,1	6,5	3,8	5,7	5,1	3,8	3,8	4,3	5,5	3,8	6,0
	EFEMP1	10,4	8,8	9,6	12,9	12,0	12,7	11,3	12,7	12,4	12,6	9,0	9,7	10,3	8,9	10,3	11,9	11,8	11,8	10,8	11,3	11,0	9,1	12,0	12,6	11,7	11,4	11,3	12,6	10,4	10,8	11,2	11,9	11,9	8,4	11,8	9,8	9,7	9,6	7,1	11,8	7,0	8,0	8,9	10,5	11,6	11,0	6,2
	TACR1	6,6	5,7	7,7	9,1	8,1	11,1	9,7	7,9	7,1	6,7	6,5	6,6	11,1	6,6	6,9	8,6	8,9	8,7	11,2	10,3	6,2	7,0	8,1	8,2	7,4	7,6	8,5	6,3	7,0	6,2	6,3	8,9	9,7	8,5	7,0	6,5	7,2	6,4	6,3	7,1	7,7	7,6	5,7	6,1	10,1	7,7	8,2
	RPL41	10,4	8,4	7,7	9,3	10,0	7,0	6,3	8,4	9,1	9,1	8,6	7,4	8,7	5,1	7,3	9,0	9,2	8,4	9,0	7,7	5,9	7,4	6,2	9,0	6,1	8,9	9,6	7,8	8,2	9,1	9,1	8,3	8,7	7,6	9,6	6,9	4,9	7,4	4,8	6,4	6,6	5,0	8,1	8,6	6,6	4,7	8,4
	HSPA1A	11,0	10,3	10,0	10,6	10,9	11,6	10,8	11,4	10,7	9,9	10,5	10,9	9,0	9,9	8,5	10,9	11,3	11,8	7,5	11,5	10,4	11,0	7,0	9,3	10,9	7,0	7,9	10,2	9,1	10,8	10,9	7,2	7,8	10,1	7,7	11,0	9,7	10,7	11,3	10,5	7,2	7,0	10,5	8,5	10,7	8,3	7,3
	ANPEP	7,3	7,2	7,1	6,8	7,2	7,7	10,7	7,8	7,9	6,8	12,3	11,1	9,1	10,7	10,0	8,1	6,7	10,5	8,3	11,4	9,2	7,2	7,0	6,8	6,7	6,8	6,8	6,9	7,1	6,7	6,6	9,7	10,2	7,6	6,8	6,8	6,9	6,7	6,8	6,8	6,6	6,8	6,6	6,6	6,7	6,9	6,8
	CLEC2B	7,8	7,1	4,5	4,0	5,8	4,4	5,8	8,1	8,4	7,8	5,5	3,8	4,9	4,9	3,8	7,2	7,6	3,8	8,4	4,9	5,5	4,4	4,9	4,6	4,2	8,0	4,9	4,2	4,6	5,1	4,8	4,5	4,7	3,9	4,0	4,3	4,2	4,2	4,1	4,4	3,9	4,0	4,0	3,8	7,5	3,8	4,4
	PRSS12	6,5	6,4	7,4	6,7	10,3	6,8	6,9	9,5	11,7	8,2	10,3	7,9	9,3	6,6	6,6	8,2	11,8	8,0	12,5	9,7	7,8	8,3	7,2	10,6	8,7	8,6	9,8	6,8	7,9	7,9	8,0	8,1	8,2	6,6	7,0	6,7	8,2	5,9	10,1	7,4	7,7	7,1	6,9	6,3	7,4	6,9	6,6
	CACNA	6,4	7,1	9,9	8,2	9,8	9,3	8,5	8,4	8,3	9,8	11,0	8,7	10,2	9,9	6,0	10,0	9,8	10,3	10,4	9,9	9,4	8,2	10,6	9,2	5,0	9,9	9,2	8,5	7,5	8,8	9,3	8,6	9,3	8,6	9,4	8,0	8,6	8,7	7,3	8,4	6,6	5,3	5,1	5,7	8,3	8,2	8,6
	BVES	11,7	11,5	12,7	13,0	12,8	12,5	13,0	12,3	5,6	11,6	9,9	11,4	12,5	12,7	11,6	12,2	12,7	13,2	12,0	12,1	12,5	10,2	5,6	12,6	5,4	13,1	12,0	11,5	11,1	11,8	12,2	5,4	5,7	12,1	12,4	9,7	9,7	10,2	10,8	6,4	11,1	8,6	5,5	10,7	11,4	10,7	11,4
	PRSS35	9,0	8,4	6,0	10,5	10,5	8,1	8,2	7,5	9,8	7,8	7,0	5,9	5,9	6,0	9,9	7,0	8,2	11,3	8,8	7,6	5,8	5,7	9,1	9,6	7,1	6,9	10,9	5,4	6,4	6,4	7,0	9,9	9,4	6,0	8,8	6,0	5,4	7,1	5,6	7,9	5,0	6,8	5,8	7,2	9,8	8,3	7,3
	HOXB9	7,4	10,5	9,6	6,6	6,8	7,1	6,1	7,5	6,2	8,4	6,3	7,0	11,5	10,9	7,5	6,5	9,7	6,7	7,2	10,8	6,6	7,8	6,3	8,0	8,5	9,1	6,5	6,6	6,7	6,3	6,4	6,8	7,1	6,4	10,1	6,7	6,3	6,4	8,9	7,4	9,7	6,3	6,1	6,0	6,3	6,5	9,0
	CCL2	12,5	11,5	12,9	12,1	10,6	12,8	12,8	13,2	11,4	12,3	12,2	12,9	12,9	10,2	12,1	12,3	13,0	10,9	13,0	12,7	12,1	11,7	12,2	11,8	7,6	11,0	11,3	8,4	7,6	7,2	7,5	13,0	13,1	9,4	11,9	11,5	8,5	12,5	11,0	12,5	12,9	10,5	10,2	11,6	11,5	11,6	10,5
	ST8SIA5	10,0	9,2	11,1	10,7	8,3	9,8	8,8	10,4	9,3	6,1	6,1	10,2	9,8	10,1	10,8	8,7	10,6	7,8	7,9	10,8	11,5	9,1	7,1	8,9	8,4	7,8	7,5	8,2	8,0	7,5	7,5	6,6	6,4	11,4	11,8	10,7	6,8	11,6	7,3	9,3	11,0	6,5	8,2	11,5	7,9	7,2	7,8
	LPHN2	9,9	7,4	11,1	5,1	4,9	8,4	10,2	10,3	10,2	8,2	8,3	7,1	6,7	5,7	5,9	9,3	7,3	8,3	9,5	8,1	10,0	9,6	10,3	6,8	9,8	9,1	10,1	6,8	7,0	6,2	7,8	6,7	7,2	5,2	5,1	5,2	8,6	7,3	11,1	9,3	11,3	10,7	9,3	9,5	6,5	5,3	6,0
	IL13RA2	8,1	10,1	5,4	4,7	10,6	3,6	9,4	10,9	10,1	10,2	3,6	6,1	10,9	10,2	9,9	9,2	10,6	5,2	3,8	10,1	3,9	8,8	7,1	7,6	7,5	10,6	11,1	8,3	7,3	8,5	8,1	7,4	9,4	4,6	9,4	3,6	3,7	3,9	9,3	6,2	4,4	3,6	4,0	3,6	11,2	3,6	9,5
	PCDHB2	11,6	11,5	11,9	7,6	6,8	11,3	8,1	6,7	5,5	11,0	7,4	10,3	8,7	11,6	10,0	10,5	10,5	11,6	7,8	8,5	9,6	9,3	11,6	6,1	5,9	10,9	11,0	9,5	5,9	5,7	6,1	7,5	9,5	11,4	10,9	5,8	8,9	10,0	10,6	11,4	7,1	7,4	11,0	7,1	8,5	6,6	6,4
	LRIG3	9,1	5,5	10,2	9,3	9,8	8,7	9,6	8,7	9,9	12,6	8,9	8,2	10,2	8,5	7,9	9,9	9,3	11,9	9,3	8,5	8,9	7,4	8,2	9,1	9,4	9,2	8,1	7,1	7,6	8,5	8,4	9,3	10,3	6,3	8,6	8,2	8,4	8,3	7,8	9,0	8,4	6,8	6,9	6,0	9,9	5,1	8,0
	CALB1	6,7	10,5	8,2	5,5	10,6	9,0	9,5	8,8	9,9	5,5	5,4	6,8	7,6	12,9	5,4	8,8	12,0	5,0	6,0	7,6	11,6	8,2	12,2	5,1	7,9	9,9	6,6	5,6	5,6	5,3	6,2	7,2	7,8	7,3	6,5	7,8	5,4	6,4	9,6	8,9	7,8	6,9	5,7	6,6	8,4	6,0	7,7
	FAM198	11,8	9,2	9,6	11,8	10,7	11,7	11,3	10,5	10,6	11,4	9,4	9,0	10,3	9,2	9,6	11,3	8,7	11,7	8,8	8,8	10,0	9,1	11,0	10,7	6,4	11,7	10,5	10,7	10,6	11,9	12,1	8,7	9,9	3,9	8,2	7,9	6,4	9,1	7,2	10,7	10,3	8,1	9,2	11,6	10,3	7,2	10,2
	HGF	3,0	3,5	4,1	3,0	4,6	5,6	3,4	3,5	3,5	8,4	9,5	6,7	6,1	3,6	3,5	7,6	3,6	3,3	7,1	3,3	5,3	6,5	5,9	4,6	2,9	4,8	4,3	4,2	3,9	3,8	3,4	4,1	4,5	3,0	4,7	3,1	4,0	3,3	3,2	3,3	2,9	3,0	3,1	3,2	4,7	3,2	3,8
	F13A1	5,7	6,2	6,8	6,4	5,6	9,6	7,1	6,6	6,2	9,8	10,8	7,0	6,9	8,8	10,1	9,6	7,5	7,9	6,2	8,0	6,2	6,3	10,4	7,4	5,2	4,7	5,8	5,5	6,2	5,4	5,8	7,5	7,7	5,8	5,7	5,8	5,9	5,7	5,9	9,4	5,4	6,5	6,1	5,8	5,7	5,5	5,8
	MGP	9,6	7,9	6,7	5,2	7,8	10,3	9,2	10,8	7,6	10,6	8,5	6,9	6,7	5,4	10,1	9,8	6,7	9,1	7,8	9,1	7,5	7,9	9,7	8,9	8,1	8,6	8,2	8,4	7,7	9,5	7,5	7,2	7,0	4,9	7,0	4,4	4,4	4,5	7,7	6,7	9,0	7,7	6,7	4,9	6,0	4,3	7,1
	VANGL	7,5	7,3	3,3	5,8	7,3	5,6	6,5	7,1	7,4	5,8	6,8	6,1	7,1	6,5	5,7	5,6	7,7	7,3	8,3	8,3	5,3	3,8	3,1	6,5	2,9	6,4	3,3	5,2	4,6	6,6	6,7	4,4	6,1	5,5	3,1	5,6	2,9	5,8	3,1	3,4	5,3	3,0	2,5	3,3	7,6	3,3	5,8
	SLN	6,4	5,8	6,5	11,7	12,3	7,1	7,4	10,4	8,5	9,7	4,8	8,0	8,5	6,4	11,3	9,8	7,1	10,1	7,8	10,9	6,4	9,1	11,0	11,5	11,4	9,9	7,2	6,4	7,0	5,4	6,9	8,8	8,5	9,3	10,4	6,0	8,6	10,4	6,0	7,5	6,1	6,8	5,5	5,7	9,6	9,4	5,6
	CHI3L1	9,0	10,1	11,8	6,7	9,0	10,9	9,7	12,6	10,9	10,3	5,8	11,6	8,1	7,6	8,5	13,2	10,2	12,2	10,1	8,5	7,7	12,0	12,0	9,7	8,4	8,2	9,1	8,5	8,2	6,2	7,0	7,0	8,3	7,4	7,7	8,1	10,1	12,6	8,1	11,3	6,7	6,2	8,2	8,1	8,7	8,5	12,0
	VAT1L	6,9	9,7	7,3	9,1	10,4	9,9	9,2	9,1	6,6	8,7	6,9	7,2	10,9	10,8	7,2	7,9	10,9	10,7	12,0	10,9	8,9	9,0	10,7	12,1	11,4	11,2	7,5	8,1	6,9	6,6	7,2	8,9	10,5	8,5	8,0	6,4	5,8	6,1	6,3	8,6	7,0	7,3	7,7	9,1	7,9	9,6	9,3
	CAV2	8,8	8,1	9,1	8,8	8,4	6,4	9,4	7,9	11,3	10,6	11,7	10,8	11,2	8,2	9,1	10,9	8,4	8,8	10,0	11,5	9,2	8,0	9,2	7,1	9,5	10,1	7,8	8,3	5,8	5,3	5,0	9,6	10,2	8,0	8,2	8,7	7,8	8,1	5,1	5,5	8,6	5,3	4,8	5,3	8,2	5,2	5,0
	SERPIN	7,6	7,3	6,7	10,8	9,3	9,9	9,6	10,7	8,5	9,0	6,4	8,3	9,2	6,0	6,8	11,8	8,0	12,6	8,1	6,9	5,8	6,7	8,4	8,0	6,3	6,6	7,3	8,5	6,8	6,2	7,1	8,5	10,1	5,9	7,7	5,9	6,7	8,2	6,1	8,2	5,9	5,9	6,4	6,6	7,4	7,3	7,5
	CD74	11,4	9,0	8,6	13,0	11,7	11,9	11,7	12,5	7,6	8,3	6,5	7,0	8,5	7,7	10,9	11,7	10,7	10,9	12,4	11,4	10,2	7,1	7,9	10,1	6,7	6,5	6,9	9,6	8,4	7,2	8,3	9,7	9,9	8,7	11,2	10,0	6,5	7,2	6,1	9,0	6,5	7,0	6,6	7,1	12,3	11,6	7,0
	SLC14A	6,4	5,6	7,7	5,1	7,1	5,7	4,9	6,3	7,5	9,1	5,8	10,4	9,3	8,7	9,2	4,9	10,3	5,9	7,4	10,5	7,3	5,9	5,2	8,6	6,6	5,5	5,3	5,3	6,8	7,1	7,0	5,7	5,8	5,1	7,4	5,4	5,1	4,7	7,6	5,8	6,2	5,1	4,6	6,1	5,1	5,0	5,2
	ARHGA	8,8	9,9	7,5	9,4	9,3	6,2	8,3	8,3	9,2	9,5	11,3	9,8	9,4	7,6	7,0	9,2	10,0	7,9	10,3	10,3	6,5	6,3	8,3	8,4	9,7	9,2	8,6	8,3	5,6	6,1	7,8	8,2	8,9	5,0	7,7	7,4	8,8	8,1	9,2	9,1	8,5	4,7	8,6	7,9	7,9	4,4	5,8
	HHIP	9,1	11,4	11,7	7,4	8,0	6,4	6,9	6,4	5,9	11,8	7,0	8,9	8,1	6,4	6,1	6,2	5,9	6,3	5,8	8,9	10,9	6,2	7,9	8,7	10,1	10,0	8,4	6,1	6,1	5,7	5,8	6,4	7,3	5,9	6,9	6,8	8,1	8,5	7,4	7,7	7,2	7,0	5,7	7,8	6,5	5,9	6,1
	LUM	7,9	6,7	3,9	3,7	3,7	4,7	4,6	7,0	7,0	10,5	11,4	9,5	3,7	3,9	6,8	9,0	5,7	4,4	3,5	9,5	7,7	4,0	6,3	4,4	3,9	6,8	4,9	5,8	4,0	3,6	4,0	8,1	9,1	3,7	3,9	3,8	3,7	3,6	3,9	5,4	3,9	3,8	3,9	4,1	5,2	3,6	3,8
	ME1	5,8	9,6	4,7	10,5	10,0	4,7	9,6	7,0	9,3	6,4	8,8	6,2	4,7	8,7	9,4	6,7	10,7	11,5	10,9	10,3	5,5	4,4	9,1	4,8	4,5	5,1	6,2	4,6	5,1	6,0	7,5	9,2	10,1	4,5	4,5	6,8	4,5	5,7	4,6	6,0	4,5	4,6	4,0	6,1	9,0	4,6	4,7
100 highest genes in H1	TFPI2	7,8	7,9	5,5	8,8	10,0	6,4	8,8	5,9	7,2	8,7	11,9	10,6	11,7	9,2	6,7	7,4	10,3	5,8	10,3	10,6	8,8	7,5	8,4	6,7	11,8	7,8	9,6	6,1	6,3	6,5	6,7	9,3	9,9	9,4	10,7	8,7	11,1	10,6	6,6	6,9	8,7	5,8	7,1	5,8	7,6	5,3	5,7
	FGF5	5,7	7,2	5,7	5,9	6,8	5,7	6,3	6,7	6,7	10,9	12,0	9,9	7,6	6,9	5,4	7,1	8,8	7,8	6,3	7,1	5,9	5,7	9,7	9,2	6,3	10,3	6,3	7,0	5,7	5,5	5,3	5,4	5,7	6,0	6,0	5,4	5,7	4,9	5,9	6,7	6,8	6,2	5,4	5,3	5,4	6,1	6,2
	MXRA5	6,2	6,6	7,5	10,7	8,5	10,5	9,6	7,5	5,5	6,0	10,2	8,2	8,7	5,7	9,2	8,3	6,3	9,8	6,2	10,9	7,1	6,1	6,9	6,1	6,0	5,3	5,6	5,7	6,8	6,0	6,1	7,4	7,5	5,8	5,8	5,9	5,7	5,5	5,7	8,3	5,6	5,8	5,5	5,6	6,4	6,2	5,8
	GULP1	8,3	5,9	7,5	7,6	8,9	8,0	6,7	8,5	7,5	9,1	8,1	7,5	5,7	8,1	4,2	8,3	8,3	8,0	7,4	7,0	6,6	4,7	7,7	7,9	8,6	9,0	8,7	10,3	5,3	6,4	6,6	5,9	7,0	4,7	8,1	4,5	6,5	4,6	4,0	6,5	8,3	7,3	4,7	8,4	8,5	7,2	7,1
	HLA-DP	11,5	9,9	8,6	13,4	12,7	12,3	11,8	12,7	6,2	8,9	6,4	7,1	7,5	6,4	11,2	11,6	11,3	11,1	12,7	12,2	10,7	7,0	8,7	10,7	6,2	6,1	6,3	7,7	8,5	8,3	9,4	10,9	10,7	9,2	12,4	10,2	5,6	6,8	6,5	9,9	5,4	7,1	6,1	6,6	11,9	12,6	6,4
	EDNRA	5,9	6,5	6,1	5,2	7,8	8,2	6,0	5,5	6,3	9,8	11,8	10,9	5,6	6,5	6,1	7,7	7,9	8,6	10,4	9,5	8,8	5,5	7,4	6,5	5,3	5,8	5,3	5,7	5,7	5,0	4,9	5,6	6,0	5,1	5,1	5,1	5,4	7,4	5,1	5,2	5,5	5,5	4,6	5,0	5,3	5,1	5,7
	AHR	10,5	10,6	10,8	11,8	10,9	10,5	10,6	11,7	11,9	9,1	11,8	11,2	11,3	9,9	11,0	12,1	12,4	12,2	10,9	11,7	10,4	8,6	11,7	9,3	5,0	6,0	5,1	10,9	5,4	5,1	4,5	10,7	10,8	10,0	10,3	8,4	8,5	10,2	4,9	8,8	9,1	6,8	8,2	9,2	9,0	10,1	4,8
	COL12A	7,3	11,4	6,9	6,0	7,6	7,6	7,4	6,8	5,7	9,6	11,5	10,0	9,2	9,7	8,7	10,0	6,7	7,0	6,7	6,7	7,5	5,9	8,8	9,8	6,7	7,5	7,6	9,8	6,8	6,6	7,9	8,2	8,7	5,9	6,4	5,4	5,7	8,1	5,5	7,9	5,4	8,6	5,5	6,0	5,8	5,5	5,7
	PCDHB5	10,2	10,7	7,9	9,1	7,6	11,4	6,8	9,4	10,2	6,1	6,8	7,0	11,6	9,1	9,6	10,5	11,3	11,9	9,9	5,0	7,3	11,5	12,1	11,3	11,3	7,2	6,9	6,6	10,9	11,4	11,9	11,4	11,7	7,2	6,4	6,4	7,0	7,9	6,7	12,1	7,8	7,3	11,9	12,4	6,2	7,1	6,1
	SPP1	10,1	11,5	12,7	12,1	12,2	12,5	12,3	12,4	9,5	12,5	5,5	7,3	13,0	13,0	11,7	10,9	11,6	12,4	13,1	13,4	11,6	11,3	12,7	12,5	12,3	12,3	11,1	11,5	9,0	9,1	10,1	11,7	11,8	11,5	11,7	9,4	9,7	10,7	11,2	12,2	11,6	7,4	10,3	8,3	8,9	11,9	10,6
	HLA-DM	9,7	7,7	7,8	12,2	10,5	10,7	10,0	10,8	6,9	7,7	8,3	7,8	8,4	6,8	9,1	10,3	9,0	9,6	11,1	10,1	8,9	8,4	7,6	10,0	7,5	8,8	8,3	9,8	7,6	7,0	7,5	9,6	9,6	7,5	10,4	8,7	6,9	8,4	6,6	7,8	6,7	7,5	6,4	7,0	10,3	10,5	7,0
	ENPP1	7,6	8,8	8,6	7,0	9,1	6,6	8,0	7,0	7,6	9,0	10,8	8,5	8,0	9,2	5,9	7,9	9,8	7,5	9,1	8,2	7,9	6,4	9,2	6,6	7,4	9,4	7,3	7,4	6,6	8,0	8,0	7,0	8,0	5,3	7,2	4,7	6,7	7,4	4,6	7,5	7,1	4,4	6,2	4,9	6,4	4,2	6,7
	ACTA2	11,1	10,6	9,6	9,6	11,5	11,0	9,7	10,6	9,8	12,9	12,0	12,3	12,1	9,4	10,7	10,2	9,9	12,1	9,6	8,7	8,0	7,6	10,0	10,0	9,0	9,5	9,6	5,9	8,3	8,2	8,5	11,6	11,7	7,5	9,9	6,7	9,6	8,4	9,7	10,9	9,3	7,5	7,1	7,9	7,3	9,5	8,5
	PLXDC2	9,2	8,6	9,8	11,1	10,7	10,9	6,0	5,4	5,2	10,7	9,7	8,6	10,3	5,5	9,1	10,4	9,1	6,7	5,8	9,4	6,2	7,8	9,2	9,4	6,2	8,0	7,5	5,8	5,6	7,5	6,7	10,2	10,2	8,2	8,4	9,6	5,1	6,3	8,0	7,1	5,1	8,3	5,1	5,3	10,0	7,2	6,0
	MSTN	5,2	4,4	6,0	9,4	8,8	5,4	7,8	9,0	11,7	6,8	9,1	4,6	7,5	4,6	6,7	9,2	10,8	6,8	7,7	8,5	4,3	7,5	5,3	6,9	4,4	5,4	7,4	5,1	4,7	4,7	4,8	6,1	6,5	4,9	5,4	5,9	6,2	5,3	3,9	5,6	4,2	4,6	4,3	4,0	9,9	5,5	5,1
	C1S	6,6	6,3	7,6	11,5	8,7	11,0	10,3	10,8	10,0	10,0	8,9	7,1	7,0	7,0	7,9	11,0	8,1	9,3	9,9	8,6	9,2	8,3	8,6	9,3	7,0	7,8	6,7	8,5	7,2	6,2	7,1	7,2	8,3	6,0	8,7	6,6	6,2	8,3	6,5	8,9	6,1	6,5	7,3	7,8	8,3	7,4	8,0
	SLC44A	6,7	7,8	9,8	7,7	7,5	7,9	8,7	8,8	7,7	7,5	5,1	7,6	7,1	7,6	8,1	8,6	9,4	9,9	9,3	7,3	6,5	7,6	9,5	6,6	5,7	8,4	9,5	5,1	5,6	5,2	5,8	8,6	9,2	6,8	8,3	5,7	5,1	5,6	5,8	7,1	5,3	6,5	5,2	6,2	9,4	8,9	7,3
	TPPP3	12,6	9,7	10,9	11,9	12,4	12,2	12,8	12,5	11,6	9,3	10,7	9,4	11,4	12,3	11,0	11,0	12,6	11,3	11,5	11,5	9,7	11,0	11,5	9,4	10,5	11,4	11,8	9,6	8,6	9,0	9,4	11,8	12,3	10,9	11,3	10,4	10,7	10,4	9,8	8,5	8,8	7,4	8,1	7,2	13,9	9,0	11,8
	CFH	7,3	5,9	5,8	4,4	4,0	7,0	6,9	8,4	4,9	10,5	10,5	7,4	4,3	4,2	5,4	7,6	9,3	5,4	4,4	6,5	6,0	4,8	7,7	4,2	4,2	5,7	5,3	4,8	4,4	4,1	4,2	6,1	6,9	4,1	4,9	4,0	4,1	4,1	4,1	5,2	4,0	4,4	4,3	4,1	5,3	4,1	4,3
	COLEC1	10,3	6,7	10,2	11,0	11,1	10,7	11,4	11,1	8,1	10,8	9,6	9,6	6,8	6,1	9,1	10,1	6,3	7,7	7,3	9,5	10,0	7,3	7,6	8,7	6,0	6,2	5,8	9,7	7,4	7,1	6,9	9,3	9,1	7,5	9,2	8,5	6,0	6,5	7,5	8,2	6,2	6,4	6,3	6,3	9,0	7,3	6,5
	SERPINI	7,2	6,4	9,1	4,5	8,0	6,6	5,9	10,7	6,4	5,6	7,7	5,8	4,7	9,1	8,0	6,2	4,5	6,3	6,6	8,1	7,8	5,4	10,4	5,6	9,8	6,5	6,5	6,3	4,5	4,7	4,9	5,4	5,9	4,5	4,7	4,4	4,2	4,2	7,0	6,0	6,7	6,0	6,1	5,6	5,4	5,0	5,0
	ENPP5	8,3	6,0	4,8	7,9	6,8	7,1	4,3	8,8	4,1	8,1	6,5	6,7	5,7	4,9	7,8	9,7	8,1	6,4	4,3	7,9	4,4	5,0	4,7	6,9	6,0	6,6	4,7	4,4	5,6	6,1	6,9	7,3	8,2	4,4	4,5	4,4	4,5	4,5	4,4	6,1	4,6	4,6	3,9	4,8	9,4	4,1	8,2
	HSPB8	8,8	8,2	6,4	10,1	7,9	9,8	10,7	10,7	7,1	9,5	10,9	9,1	10,3	8,8	9,9	8,6	6,1	10,4	7,1	8,5	6,1	8,3	9,9	10,0	7,6	6,4	7,3	7,0	6,8	6,2	7,1	9,0	10,3	6,8	8,1	7,4	7,3	6,7	6,2	9,0	5,6	8,0	6,8	5,8	9,0	8,3	8,6
	CXCL14	9,9	10,2	11,7	10,2	10,8	11,4	13,0	12,9	12,4	11,2	7,2	8,9	11,4	11,6	9,1	12,1	13,1	12,3	12,7	11,7	11,8	10,6	11,2	11,4	8,5	12,7	8,2	10,0	7,3	6,9	8,5	10,7	12,1	7,7	9,2	7,2	9,0	10,5	9,5	12,2	9,9	7,2	7,4	10,6	10,2	9,4	10,8
	DKK1	8,0	6,8	5,9	12,4	11,9	11,6	10,4	9,4	12,4	11,1	11,1	9,3	12,2	12,9	11,5	10,6	12,0	12,8	13,1	12,8	7,9	8,3	11,5	12,4	12,1	12,6	6,7	10,0	11,5	11,5	12,1	11,2	11,9	11,1	10,1	10,4	4,6	4,8	5,6	8,4	8,6	5,4	5,2	5,0	6,3	7,8	5,2
	PDGFD	6,8	7,6	8,6	10,2	9,2	8,6	7,7	9,0	10,2	7,5	5,4	7,2	8,7	7,8	7,5	7,3	7,8	9,9	8,6	8,1	8,7	6,7	8,5	8,0	6,0	8,6	7,4	5,8	4,9	5,9	5,1	7,8	8,7	5,1	6,8	5,1	5,1	7,8	4,9	8,5	7,0	6,8	7,3	8,5	7,2	6,4	5,6
	TDO2	6,5	5,6	6,4	7,0	8,7	11,0	7,4	10,3	7,6	7,5	4,3	5,0	6,1	4,8	7,6	9,2	8,6	7,0	9,3	7,3	5,0	5,7	7,2	8,2	6,3	6,4	7,0	5,9	5,1	4,6	4,2	6,1	7,6	4,6	6,2	5,1	6,9	6,1	4,9	5,1	4,0	4,9	4,2	4,6	9,7	4,7	5,7
	TSHZ2	8,9	10,7	10,5	9,4	7,8	9,0	9,4	9,4	9,6	9,4	5,5	6,3	9,1	8,8	8,4	10,5	8,4	10,0	9,1	9,3	9,0	5,9	9,7	8,0	7,4	9,9	5,2	8,2	6,3	6,5	6,5	8,6	9,4	8,2	8,4	7,4	9,4	6,7	8,0	9,0	9,4	6,9	5,9	5,3	8,0	7,0	6,7
	OMD	5,9	4,8	5,7	8,5	5,7	5,5	4,4	7,5	3,7	9,0	6,1	5,1	7,4	5,0	6,5	8,5	5,2	6,2	4,3	6,5	6,3	4,0	3,9	7,3	4,3	4,0	4,0	7,5	4,0	3,8	3,6	7,3	6,8	3,7	4,3	3,6	3,9	3,9	4,1	4,8	4,2	4,2	3,4	4,1	3,7	3,9	3,8
	PCDHB1	10,7	10,8	10,8	11,1	11,1	9,1	9,7	7,6	5,9	10,4	7,8	10,2	10,1	10,8	10,0	9,6	9,2	10,8	5,2	7,6	9,7	9,8	9,9	10,3	7,9	10,7	6,6	8,1	7,7	9,0	9,2	9,1	8,1	7,2	7,6	7,4	8,4	7,5	8,0	10,9	6,5	6,9	10,3	10,3	10,5	7,5	8,4
	HLA-DR	11,6	8,8	8,6	12,8	11,8	11,8	11,5	12,2	6,0	8,6	5,6	6,2	9,5	6,4	11,2	11,3	11,1	10,9	12,5	11,4	10,6	7,1	8,6	11,1	5,6	5,5	6,2	10,0	7,6	5,9	7,4	10,8	10,5	9,8	11,7	10,7	5,7	7,3	5,6	9,0	5,9	7,1	5,3	6,4	11,7	11,8	6,0
	FN1	10,8	11,0	10,6	11,3	11,9	10,8	10,4	10,0	12,1	12,8	13,5	13,2	10,9	9,1	12,6	12,0	11,2	12,3	11,3	11,9	11,6	9,7	11,8	10,7	10,7	9,1	10,9	12,7	9,6	10,5	10,7	11,1	11,1	9,2	10,0	9,7	9,5	10,2	9,8	9,5	10,0	9,7	8,6	9,4	9,8	7,2	7,4
	GPX8	8,9	9,9	8,7	5,3	9,0	7,9	7,8	8,5	7,8	9,1	10,8	10,0	9,1	9,1	8,7	9,2	8,8	8,6	9,2	8,8	7,1	7,5	9,3	9,5	8,8	9,3	7,2	9,4	5,8	7,6	8,6	6,9	7,7	4,2	8,8	4,7	7,1	8,7	7,1	8,4	7,4	6,4	8,2	5,2	7,2	4,0	6,1
	ACTG2	11,0	9,3	9,3	8,8	12,3	9,4	7,8	11,6	8,1	12,8	12,8	12,5	12,1	6,3	12,5	12,6	11,7	12,9	8,1	10,0	7,9	5,8	10,4	8,0	8,4	9,1	5,9	11,1	6,7	6,4	9,0	11,8	11,9	6,5	8,5	5,6	5,5	5,9	5,9	11,7	6,0	7,4	5,6	5,9	7,8	7,5	5,5
	PCDH20	8,8	5,6	7,9	9,2	9,3	10,0	7,1	9,5	6,2	8,0	4,8	5,9	6,2	5,6	6,3	7,8	7,8	10,5	5,9	8,7	7,1	6,7	9,9	6,4	8,2	10,0	8,3	7,4	7,6	9,7	8,4	6,3	7,8	4,8	5,1	6,8	6,2	5,0	5,1	5,5	5,8	7,1	4,9	4,9	5,9	5,9	5,5
	SYT1	5,6	7,7	6,5	4,7	7,7	9,6	6,0	10,1	9,4	11,5	10,0	8,5	7,3	8,1	9,8	8,7	9,6	6,6	5,4	10,6	8,3	8,4	9,3	6,4	5,0	10,0	8,6	5,3	6,2	7,1	7,2	7,8	6,9	4,9	6,0	5,0	5,3	5,0	5,2	8,0	4,9	4,9	5,3	7,7	9,1	6,0	10,5
	PI15	9,8	9,6	9,6	11,8	12,8	9,8	11,3	12,1	12,3	7,5	5,1	6,3	11,8	5,3	7,9	10,7	11,7	10,8	12,0	8,8	6,4	5,3	7,7	8,0	6,8	11,5	7,8	9,0	6,1	6,7	7,2	10,5	10,1	5,6	7,8	6,9	8,1	6,7	5,1	9,7	6,1	5,9	5,8	5,2	11,6	6,3	8,2
	CP	8,2	7,1	8,4	10,2	10,0	7,8	9,2	9,4	8,7	4,8	4,5	5,1	7,3	4,5	4,8	9,1	5,7	6,4	8,4	4,7	5,4	4,6	4,4	5,1	4,7	8,9	7,7	5,7	5,0	4,5	4,8	5,4	5,7	4,5	6,1	4,5	4,4	5,4	4,6	5,7	4,4	4,7	4,5	4,4	10,7	4,7	5,9
	TRIM22	10,3	9,3	10,9	10,3	10,8	10,4	10,5	10,7	10,8	9,9	10,5	9,7	10,1	10,9	8,8	9,9	11,0	10,1	9,8	9,3	9,6	9,4	8,2	9,6	7,1	9,4	7,2	6,4	7,3	6,9	8,0	9,7	10,4	5,3	9,3	7,5	8,8	8,4	8,0	10,2	9,0	8,9	7,5	10,4	8,2	6,9	8,3
	ADAM1	10,0	11,8	10,7	11,3	11,0	10,5	10,7	10,3	9,8	10,8	11,2	11,0	11,1	10,8	10,5	10,6	10,7	11,7	9,5	10,7	10,3	9,0	11,2	11,5	10,9	10,2	9,5	11,1	8,0	7,7	9,1	11,4	11,8	11,0	10,7	9,4	9,5	10,7	8,9	9,6	9,0	6,0	7,7	10,1	6,8	7,6	7,3
	POSTN	11,6	11,8	11,2	12,4	12,1	10,7	11,4	11,7	12,4	12,0	10,0	11,8	7,7	12,5	11,6	12,2	12,5	11,8	11,4	10,4	9,2	9,7	12,2	11,7	12,4	8,5	9,7	9,7	6,7	10,0	6,9	11,1	11,8	11,2	11,1	6,8	5,5	9,1	6,0	10,7	6,5	11,5	11,6	7,9	5,9	10,6	6,2
	COL14A	6,9	9,5	7,3	11,1	10,5	10,9	10,6	6,5	7,7	9,2	6,9	8,2	10,7	8,4	8,4	11,1	8,2	10,8	10,5	9,1	6,3	6,3	7,6	8,0	8,3	8,4	7,0	7,1	7,0	6,5	8,6	9,1	10,0	6,1	8,7	6,1	5,9	5,7	6,2	7,6	6,5	6,2	5,9	6,1	6,9	6,4	6,3
	CTGF	11,8	12,0	11,1	12,3	11,2	11,0	11,0	10,6	10,8	12,9	13,2	12,8	12,6	11,4	11,6	10,5	10,9	10,6	10,8	11,1	10,3	9,5	11,6	11,2	10,8	11,7	11,7	10,7	8,4	9,2	10,9	11,8	12,0	7,8	12,1	6,9	11,1	11,0	8,6	11,9	11,4	6,8	8,5	6,9	9,2	9,7	8,3
	KCNJ6	7,5	7,7	9,5	10,0	9,4	8,1	8,5	8,9	6,7	5,7	9,5	9,0	9,9	5,6	6,5	8,6	7,3	8,2	8,8	10,1	6,8	6,5	7,7	8,8	6,7	8,7	7,8	5,4	5,6	5,4	5,5	8,7	9,4	5,7	7,3	5,9	7,1	8,5	6,1	8,8	8,5	8,8	8,9	8,3	6,2	5,6	5,7
	LMO3	11,4	8,4	5,3	5,1	5,1	8,5	11,4	10,9	12,6	11,4	6,7	10,1	6,7	7,3	5,5	11,6	7,6	8,0	6,1	6,5	10,9	10,0	10,5	10,6	5,3	10,7	6,2	5,3	5,9	6,8	7,3	6,2	5,1	5,1	5,2	5,4	5,3	5,4	5,7	7,2	9,6	9,0	6,8	8,0	5,9	5,3	5,4
	TMEFF2	7,8	11,2	10,6	10,0	11,5	10,7	10,5	9,7	9,0	10,5	8,8	9,3	11,0	12,5	10,8	9,8	11,6	10,6	10,4	10,5	8,8	8,8	11,9	6,4	8,3	10,5	8,0	6,4	7,5	7,9	8,8	10,9	11,3	9,6	10,1	7,0	6,2	6,6	5,0	10,6	5,1	6,4	6,3	5,4	8,8	11,3	10,0
	SLC7A1	10,8	9,2	10,8	8,8	10,8	9,1	10,2	10,8	11,7	10,3	9,5	10,3	11,7	10,1	11,0	11,4	10,2	11,2	12,3	11,0	10,6	9,1	10,3	11,3	9,0	10,3	9,4	9,7	6,3	7,0	8,0	8,7	9,4	6,4	8,3	8,8	8,1	8,5	9,1	9,9	9,6	7,0	9,6	11,2	9,9	7,0	9,9
	SEMA3C	8,2	8,4	9,6	8,2	10,1	10,1	9,8	9,0	10,5	9,3	11,9	8,9	9,7	8,9	7,2	10,7	8,6	10,4	7,9	5,8	7,0	6,5	10,1	5,6	4,3	9,9	4,7	8,5	6,7	8,3	7,9	6,9	8,0	6,1	9,3	4,7	5,0	4,8	5,1	9,4	5,4	5,0	4,5	4,6	7,6	4,9	6,5
	MYO1B	8,2	10,4	6,7	5,6	7,6	9,7	5,9	9,5	9,6	8,7	11,8	10,2	9,5	10,1	10,4	9,4	6,8	11,1	5,8	9,4	7,5	5,3	9,6	6,6	6,5	8,6	4,8	10,8	5,5	5,6	6,4	7,7	7,3	6,6	6,1	7,2	5,1	8,6	7,8	6,2	5,1	5,0	4,7	4,8	4,8	5,7	7,0
	RAB27B	7,2	6,1	7,7	9,4	10,6	9,0	9,6	9,4	12,5	7,6	11,2	9,2	9,4	8,5	9,5	8,8	10,9	8,3	12,1	11,1	7,9	7,5	9,0	10,5	10,4	9,0	8,5	7,3	6,7	7,4	6,5	8,7	8,9	8,8	10,0	8,6	9,3	8,1	6,0	7,1	6,4	6,2	6,0	6,3	8,8	7,3	9,2
	IFITM1	6,2	6,9	9,2	8,9	8,5	9,8	9,2	8,4	8,8	8,3	10,3	7,1	6,0	6,1	7,1	8,8	10,9	10,5	8,4	7,4	7,5	11,5	8,1	7,8	8,9	7,3	6,7	8,1	9,4	8,8	9,4	7,0	7,6	6,3	6,7	6,5	6,4	6,0	6,2	6,2	6,0	6,5	6,1	6,3	7,1	6,1	6,5
	HOXB2	10,9	12,0	11,9	10,0	7,4	9,2	9,5	11,6	10,4	8,1	7,6	11,0	12,0	11,9	10,0	10,1	7,3	10,6	11,4	10,8	10,9	10,5	7,6	7,9	8,0	11,7	7,6	7,9	7,7	7,5	7,5	11,5	11,8	7,2	11,8	7,7	7,5	8,7	7,6	8,7	12,1	8,1	7,8	8,7	9,3	7,2	10,1
	VRK2	3,9	8,0	3,9	4,0	3,9	3,9	6,0	7,6	4,0	7,7	8,1	6,1	3,9	6,3	4,1	6,5	3,9	5,5	8,2	8,1	7,1	6,0	7,2	6,8	5,5	3,9	4,0	7,8	4,1	3,8	3,8	3,7	3,8	3,8	6,8	3,8	4,0	3,7	4,0	3,8	3,9	4,3	3,8	3,8	5,3	4,0	3,9
	CFI	7,4	6,0	7,6	9,0	8,5	9,3	9,2	9,0	5,2	8,7	4,7	6,8	6,8	5,7	6,3	9,4	7,6	10,1	8,1	6,8	8,4	6,2	9,4	8,0	5,8	9,3	5,2	5,3	5,1	4,8	4,6	7,4	8,0	4,6	8,8	5,8	5,4	7,2	5,0	8,1	4,6	5,2	5,5	5,6	6,7	6,0	5,4
	SERPINI	11,2	12,0	11,6	12,5	12,0	12,0	11,7	11,0	9,6	12,1	13,5	12,8	13,3	11,8	12,7	10,5	11,6	12,1	11,9	12,3	11,8	10,2	12,4	11,7	11,9	12,5	12,1	10,3	9,4	8,6	10,5	12,4	12,3	10,1	11,1	9,9	10,6	10,5	10,2	11,6	11,9	7,8	8,7	8,7	11,1	9,4	9,3
	CAV1	12,2	12,4	12,2	12,2	12,4	12,3	12,6	12,0	13,1	12,5	13,2	12,9	12,7	12,6	11,9	12,6	12,7	12,0	12,2	13,0	12,0	11,5	12,2	11,9	11,7	12,3	11,8	12,8	11,1	11,5	11,4	12,2	12,1	11,0	11,0	10,4	10,7	9,8	9,1	9,8	11,7	6,9	6,9	8,0	10,2	8,0	10,7
	MOXD1	9,3	10,9	10,3	9,8	11,7	11,0	12,2	10,7	9,4	9,9	8,0	7,9	11,3	11,1	8,1	10,8	11,3	11,7	10,7	10,9	11,5	9,6	11,7	10,4	9,2	11,8	10,4	9,0	6,3	7,9	7,3	9,2	10,7	6,7	8,5	9,0	10,1	11,3	6,1	9,5	7,9	8,2	6,2	8,8	9,3	5,4	8,6
	FAM26E	4,9	6,8	5,2	5,5	8,3	5,3	5,7	4,8	5,4	8,7	11,0	10,7	6,6	8,6	7,9	8,0	9,1	8,0	7,4	7,5	6,1	4,9	6,2	7,7	4,6	6,9	5,5	4,9	4,8	5,1	4,9	6,5	6,7	4,8	5,6	4,6	4,7	4,7	5,1	4,8	4,5	4,9	4,5	4,6	5,3	4,9	4,8
	GLIPR1	12,2	12,9	11,7	12,7	12,9	12,7	12,7	12,5	12,4	13,1	13,1	12,5	13,0	13,4	12,7	12,5	12,9	13,2	12,1	12,1	10,6	10,5	12,6	11,9	10,1	12,1	11,9	10,4	11,6	11,4	12,5	12,5	12,6	11,3	12,3	9,8	7,8	9,1	11,4	11,7	9,9	9,0	7,9	8,1	11,1	10,1	10,6
	MFAP5	9,3	11,2	9,5	8,2	8,4	8,5	9,4	7,8	9,2	11,4	6,8	7,9	11,0	7,5	6,8	8,5	8,2	11,8	11,8	12,8	6,0	5,9	7,5	11,0	10,1	9,9	6,8	6,5	6,0	6,0	5,6	9,7	9,7	5,9	7,6	5,9	5,6	5,9	5,5	7,0	5,2	6,0	5,5	5,3	6,2	6,0	5,5
	F2RL2	6,6	7,7	8,3	6,4	8,8	6,0	7,8	5,2	5,6	10,8	10,9	6,2	8,0	9,0	7,0	7,6	7,7	6,7	7,7	8,8	9,5	6,1	8,9	6,7	5,9	8,1	5,1	8,9	5,7	5,1	5,9	6,4	6,2	6,7	6,3	5,6	5,6	5,6	7,1	6,2	5,5	5,0	5,2	5,6	6,0	5,4	6,2
	LIPH	5,4	5,0	5,1	8,5	8,5	8,2	7,2	8,7	8,1	4,9	9,6	6,2	6,7	5,9	7,2	7,8	7,9	7,3	8,8	6,2	5,7	6,1	8,2	8,1	4,6	7,2	6,4	6,1	5,4	5,3	6,3	6,1	6,4	4,5	6,1	4,4	4,7	4,6	4,5	6,5	4,3	4,8	4,6	4,4	7,8	5,2	6,5
	PAWR	9,1	8,4	9,4	7,8	10,0	8,8	8,0	9,1	8,5	10,1	9,4	9,2	10,1	8,6	8,5	9,2	9,9	9,3	10,8	9,8	8,3	7,4	8,5	8,9	8,4	9,9	9,2	6,8	8,2	8,9	9,3	9,1	9,9	4,9	8,9	5,0	5,9	5,3	4,4	8,5	4,8	4,7	5,1	6,8	8,6	4,7	8,3
	RGS4	7,8	10,7	7,5	7,2	8,6	9,3	7,3	9,2	6,3	10,1	12,0	10,9	10,2	9,2	8,7	6,6	9,8	8,1	8,7	9,6	8,7	9,2	11,0	8,9	7,9	10,2	7,7	5,7	6,0	5,6	6,0	9,2	9,7	7,6	7,6	5,6	6,0	6,4	5,9	8,2	7,2	6,6	7,3	6,1	7,2	8,5	8,4
	COL1A2	7,5	9,8	8,4	9,8	9,6	11,5	11,0	11,3	7,6	12,0	12,6	11,8	8,9	8,6	10,4	11,6	11,3	10,8	8,3	10,7	11,7	11,5	9,5	10,6	9,7	7,9	8,3	11,5	9,4	10,1	10,7	10,9	10,7	7,8	8,5	7,1	7,0	8,0	7,1	6,9	7,0	7,6	6,8	8,1	7,9	7,2	7,3
	ERAP2	9,6	8,7	8,4	8,0	9,7	8,7	8,4	9,5	6,1	5,9	9,4	7,9	9,2	8,7	5,8	10,0	9,4	6,0	6,3	6,1	8,6	7,3	9,1	6,2	6,0	9,8	5,7	6,2	5,5	5,5	6,3	5,5	5,9	7,2	5,9	7,7	7,3	6,2	8,8	8,2	7,7	5,3	8,1	7,0	5,8	5,9	7,5
	TFAP2C	8,2	7,4	6,5	9,2	8,8	8,5	8,2	8,7	8,5	6,2	10,0	9,1	7,0	10,1	8,5	8,2	10,5	9,1	10,4	10,2	7,5	6,6	6,6	5,9	5,4	6,9	5,8	9,3	6,7	7,2	7,8	7,1	7,6	7,2	7,6	6,1	6,0	5,9	6,1	6,0	6,0	5,8	5,6	5,6	6,5	5,6	6,6
	SLIT2	10,0	9,0	10,6	5,4	7,4	8,4	8,6	10,6	9,6	8,3	8,0	8,0	8,1	5,2	5,5	7,8	9,4	6,8	8,3	6,7	9,2	5,7	7,1	8,5	5,1	9,2	6,8	7,0	5,5	5,4	5,8	5,3	5,5	5,4	5,6	5,1	5,7	7,1	5,0	8,0	10,3	6,5	5,0	5,1	5,6	5,4	7,0
	ABCC3	9,9	9,8	9,5	11,5	10,3	10,8	11,6	11,3	9,6	7,7	7,5	9,8	11,4	10,3	9,9	10,8	10,2	9,9	11,5	11,2	10,2	9,4	9,8	10,1	10,6	8,4	9,9	7,4	7,2	6,7	7,2	10,9	11,1	9,0	8,7	8,1	7,4	8,9	6,9	8,9	8,0	6,9	6,6	6,8	8,7	8,4	8,6
	MFAP4	10,8	10,7	11,1	10,2	9,1	9,3	11,3	12,1	10,3	11,4	9,7	11,9	11,3	9,6	11,1	12,6	10,4	10,4	8,3	11,0	11,9	9,3	10,2	11,3	11,1	10,8	8,3	6,6	7,7	7,8	7,2	11,1	10,5	6,9	7,9	8,4	8,6	9,1	8,7	10,9	9,7	11,9	9,2	7,8	10,0	6,4	7,1
	GCNT1	8,2	8,5	7,0	7,1	8,9	8,5	10,0	9,8	9,7	6,4	8,9	8,6	10,1	7,4	7,5	11,2	10,4	10,0	9,1	9,4	8,1	6,7	8,5	9,1	7,0	10,0	7,8	8,3	7,1	7,4	7,3	9,0	10,1	5,8	7,8	6,1	5,7	5,9	5,9	8,3	6,8	6,2	6,1	5,6	7,8	5,9	6,4
	CD109	7,6	8,6	6,9	8,5	9,6	6,7	9,5	7,7	8,7	7,5	8,6	8,0	9,8	9,5	8,2	8,8	9,7	9,4	9,7	10,1	7,9	6,1	8,7	9,5	9,5	9,2	8,8	9,4	7,2	7,8	9,1	7,6	8,7	9,4	7,8	5,9	6,2	7,5	4,9	5,7	4,7	4,9	4,9	4,8	8,3	4,7	6,3
	COL1A1	9,5	10,6	9,5	8,1	10,9	9,9	8,9	11,1	8,0	12,5	11,9	12,8	12,3	8,1	12,4	11,2	9,7	11,6	8,4	10,6	11,3	9,1	10,2	11,3	10,0	9,6	8,0	10,2	8,4	8,0	9,1	11,2	10,9	7,9	8,0	7,8	7,8	7,9	7,9	9,1	7,6	8,0	7,4	7,7	8,0	7,8	7,8
	NNMT	9,2	10,9	7,8	8,4	10,0	10,0	10,9	11,1	9,5	10,9	9,4	11,2	12,1	6,6	10,3	11,0	9,7	11,8	10,5	9,8	8,2	7,1	8,0	10,6	7,6	10,0	11,3	6,9	7,6	6,8	7,7	9,5	9,7	7,6	10,8	6,7	7,5	8,3	6,7	9,9	6,7	6,8	6,9	7,2	9,2	7,7	7,5
	CALCRL	6,9	8,8	9,9	11,8	11,9	10,6	11,6	10,4	11,3	8,8	7,6	9,2	9,0	11,1	9,5	10,7	11,9	11,8	12,0	11,3	9,9	6,4	10,9	9,4	9,2	11,0	9,0	7,0	5,1	5,4	6,0	11,1	11,4	6,7	9,6	7,6	7,1	6,7	6,4	8,9	5,1	6,8	7,7	6,3	11,1	10,0	10,2
	VCAM1	8,1	6,7	7,2	6,5	9,6	9,6	8,1	10,7	8,4	8,9	9,8	6,5	6,4	6,0	9,1	10,6	10,7	9,1	9,9	9,9	9,5	7,6	9,6	7,0	5,5	8,4	8,8	5,3	5,7	5,8	5,6	7,7	7,7	5,2	5,3	7,5	6,4	7,9	5,2	6,2	7,5	6,9	6,1	9,0	10,0	5,3	8,1
	GALNT5	8,3	7,9	7,8	11,2	10,1	6,7	6,7	8,6	10,1	8,3	10,7	7,9	8,5	6,7	8,0	9,3	10,9	9,3	8,4	8,6	7,7	7,4	6,2	8,9	6,5	5,3	8,4	8,1	5,9	5,7	6,7	7,4	7,5	7,0	8,1	6,9	5,7	6,1	5,5	7,7	5,7	5,4	5,1	5,2	7,3	6,1	5,7
	ABI3BP	6,6	5,9	7,1	11,2	11,1	10,1	9,4	9,0	11,1	10,6	12,1	11,7	7,9	7,1	8,2	9,8	9,6	10,8	8,9	10,5	6,3	7,3	8,8	10,3	8,2	8,5	8,5	10,1	6,5	7,7	8,2	7,3	7,7	5,7	7,5	5,7	6,0	6,2	5,7	7,8	5,5	6,0	5,7	5,4	9,0	5,8	6,3
	DOCK5	8,4	9,3	5,5	5,6	6,0	7,5	9,1	9,3	9,0	5,4	9,7	8,8	10,2	10,9	8,3	9,3	5,8	5,3	10,8	10,5	5,5	5,4	5,6	5,6	5,3	9,9	5,5	5,4	5,6	5,4	5,5	6,8	7,3	5,4	5,4	5,4	5,4	5,3	5,4	7,9	5,3	5,5	5,2	5,7	8,6	5,4	7,7
	ALPK2	9,1	8,1	8,7	9,4	9,0	10,5	10,6	10,3	10,0	8,2	8,2	9,2	10,2	8,0	8,0	8,2	10,2	9,9	10,1	8,7	8,4	6,6	7,9	8,7	6,1	10,6	6,4	5,3	6,1	5,6	7,1	10,5	10,8	6,0	8,8	5,4	5,3	6,1	5,4	8,5	6,9	5,7	5,7	5,6	6,1	6,8	6,0
	THBS1	11,4	11,8	10,4	12,0	11,8	11,8	12,8	12,5	13,2	12,0	13,1	13,0	12,6	12,7	12,8	11,9	11,9	12,5	12,0	11,9	10,6	9,2	12,2	11,8	10,5	11,3	10,6	12,0	9,1	9,3	10,7	12,4	12,6	8,1	10,0	9,7	6,4	9,4	7,4	12,3	11,3	6,6	8,9	8,0	9,5	8,4	8,1
	MYOF	9,5	10,5	10,4	10,8	11,1	10,4	11,0	10,8	11,6	11,2	12,5	11,4	11,3	11,7	10,4	10,4	10,6	11,1	11,5	11,6	9,8	8,9	10,5	11,0	10,7	10,5	10,2	9,9	7,4	7,2	8,7	10,9	11,5	9,9	10,2	8,3	8,6	8,7	6,3	9,1	8,3	6,3	7,1	6,9	9,0	8,6	8,5
	TNFSF1	6,4	6,7	6,9	6,0	8,2	5,8	5,7	7,7	6,4	10,0	5,5	5,8	8,0	6,4	9,6	8,8	10,3	7,1	9,2	8,2	7,4	8,5	8,7	9,5	6,1	9,0	7,1	6,2	6,1	6,4	6,2	5,9	6,1	5,2	6,0	5,2	5,5	5,2	5,8	5,6	5,0	5,1	5,6	5,2	6,3	5,2	5,4
	LAYN	8,3	8,1	8,3	9,0	9,0	8,5	9,5	8,8	10,0	10,0	7,2	6,3	10,8	8,6	7,5	9,6	9,8	9,7	9,9	9,9	7,9	5,7	8,2	9,4	8,6	9,8	7,8	7,5	6,5	5,4	5,9	8,6	9,6	5,7	7,9	6,6	5,8	6,8	6,9	7,1	8,0	5,9	6,0	5,8	7,7	5,9	7,4
	VSNL1	7,3	7,9	10,4	11,8	11,0	11,3	11,0	11,7	12,1	9,2	7,3	9,5	11,5	9,7	9,2	11,7	11,7	12,0	13,1	12,9	11,7	11,2	12,0	12,3	11,3	11,7	10,6	6,4	8,1	7,6	8,4	9,8	9,6	9,1	6,1	7,4	5,6	8,3	7,1	11,0	9,3	8,3	10,9	8,0	9,0	9,2	8,1
	IL7R	7,1	8,8	4,8	4,8	7,1	7,5	4,8	5,5	7,0	7,6	5,0	6,8	8,9	5,6	7,1	5,9	10,2	9,9	8,9	5,8	5,0	4,6	7,0	7,9	4,8	7,5	4,7	5,0	4,9	4,9	7,3	5,8	6,1	4,4	4,6	4,6	4,3	4,3	4,5	4,6	4,5	4,7	4,4	4,5	4,7	4,5	4,6
	FBXO32	8,4	7,9	8,5	10,1	10,2	10,3	11,0	10,8	9,4	10,0	8,9	8,3	10,8	10,6	9,5	10,2	10,6	11,1	10,1	10,0	8,4	8,3	9,6	9,0	9,7	11,0	7,6	7,2	6,6	8,1	8,2	10,1	10,8	6,6	9,3	6,5	8,6	7,7	5,7	9,2	7,9	6,2	6,9	6,8	7,6	7,4	7,9
	LOX	8,1	9,9	8,7	10,6	10,6	8,0	12,0	10,7	11,2	9,3	12,2	10,3	10,8	10,9	8,5	11,5	10,2	8,3	8,0	9,1	10,1	8,6	9,9	10,0	9,1	9,7	8,2	11,2	6,1	5,8	6,3	9,9	9,9	5,6	8,0	7,0	8,5	9,2	5,5	7,9	9,0	6,8	6,1	6,1	10,1	5,8	5,8
	CYP1B1	8,8	8,4	8,2	8,6	8,8	7,9	7,7	9,6	11,8	6,6	8,9	7,9	9,3	7,1	8,0	9,0	9,5	10,3	8,5	8,9	8,2	7,1	8,9	6,5	5,9	7,0	6,3	8,7	6,3	5,8	5,7	7,6	7,8	6,7	7,4	6,1	6,1	6,8	5,9	6,2	6,3	6,4	5,5	6,0	6,2	6,0	5,9
	MET	8,2	8,7	9,0	7,0	9,9	8,0	8,8	7,3	9,8	11,0	9,6	7,9	10,9	10,2	6,6	10,3	11,3	9,5	10,6	10,8	8,7	8,6	10,1	10,0	8,9	9,9	9,0	7,9	6,5	7,4	7,4	8,9	9,7	10,6	7,6	5,4	5,8	5,4	5,6	7,8	7,1	5,5	5,3	5,4	5,8	5,7	7,3
	AGPAT	8,3	7,6	8,3	9,3	9,6	7,6	8,1	8,9	8,6	8,1	5,9	6,2	8,9	9,0	8,7	8,2	9,8	10,2	11,1	10,4	9,3	6,5	10,1	9,2	5,9	8,7	6,0	8,2	6,2	6,1	6,0	7,1	8,3	8,3	8,3	6,1	6,0	6,1	5,7	6,3	7,2	5,7	5,6	5,8	6,3	5,8	6,3
	SLC2A1	8,4	9,3	9,2	9,4	11,6	7,3	10,1	9,9	10,9	8,2	9,1	7,2	10,7	10,2	8,1	10,1	11,7	9,9	11,7	9,6	8,2	7,0	8,1	9,5	9,4	11,7	7,9	7,3	5,9	5,9	6,2	8,8	9,5	6,6	8,0	6,7	8,0	7,7	6,2	8,0	6,6	6,2	6,3	6,6	8,6	6,1	6,9
	DAB2	7,7	7,4	6,2	9,1	9,1	10,0	10,0	8,6	4,9	10,7	12,8	11,3	10,3	8,6	8,8	10,5	9,6	9,5	9,4	10,1	8,6	5,9	9,0	8,3	8,4	10,4	8,5	9,8	5,6	6,6	7,4	8,4	9,2	4,7	5,3	4,8	4,6	4,5	4,9	7,7	4,8	5,2	4,6	5,1	7,6	5,4	6,3
	TGFBI	11,5	11,4	7,8	8,6	10,5	9,8	11,1	11,5	9,6	11,9	10,7	11,6	11,6	12,5	10,8	11,0	7,9	12,4	10,9	12,0	12,3	7,9	12,4	11,2	11,2	12,3	6,2	9,0	8,8	9,8	10,1	9,0	9,5	7,5	9,3	5,7	5,8	5,8	7,1	9,1	7,8	5,9	5,6	7,4	6,4	6,1	8,6
	STEAP2	8,5	8,3	7,6	8,4	9,4	8,7	8,9	9,6	9,9	8,5	6,9	6,0	9,1	9,6	7,8	10,4	10,2	9,7	9,4	9,6	9,3	6,8	9,8	8,3	7,0	10,2	7,9	6,0	5,6	5,5	5,6	7,1	7,7	6,3	5,8	5,7	5,5	7,2	7,8	7,6	6,1	6,2	6,5	6,0	7,3	5,9	9,3
	MAN1A	7,7	8,7	9,0	9,6	10,0	8,4	9,4	8,6	9,3	9,2	11,3	9,7	10,2	11,0	8,3	10,0	11,0	8,0	11,1	10,8	10,4	7,9	10,7	9,7	9,9	10,6	7,5	7,5	6,5	7,0	7,0	8,5	8,5	7,7	7,1	7,7	7,1	9,1	5,9	7,5	7,2	5,9	6,2	8,0	9,4	6,1	7,0
	POPDC3	9,3	10,5	8,2	10,1	8,1	9,1	9,2	8,9	5,4	9,0	8,1	6,8	9,8	10,4	9,6	10,1	10,8	11,1	10,5	10,5	5,5	6,3	7,8	5,2	4,8	11,3	7,4	5,1	5,2	5,6	4,3	6,8	7,1	4,6	8,9	5,1	4,9	4,1	4,4	7,4	5,1	4,9	5,3	4,5	4,5	4,5	4,4
	EDIL3	8,5	9,1	10,3	4,7	11,9	12,0	9,7	9,4	12,5	12,7	12,5	12,0	12,4	12,1	8,8	12,6	12,3	11,3	12,3	11,3	8,5	8,0	11,8	9,4	6,1	10,2	8,2	4,8	6,2	6,3	8,1	11,3	11,2	4,8	5,8	5,0	4,7	4,9	4,9	10,1	5,1	4,7	4,9	5,6	10,8	5,0	10,3
	AHNAK	9,9	9,4	10,2	10,4	10,1	7,9	10,8	9,7	12,0	9,2	11,2	9,0	11,3	10,7	8,9	10,0	10,6	10,4	9,7	10,3	10,2	7,5	9,4	8,8	10,7	10,4	6,2	8,3	7,1	6,5	7,5	9,7	10,3	7,8	7,4	6,9	6,9	6,6	5,8	8,3	8,2	5,5	6,4	5,7	7,9	6,1	6,5
	GFPT2	9,7	10,6	9,3	10,0	10,3	9,4	10,6	9,8	10,5	10,5	8,2	8,3	11,0	10,3	8,4	11,2	11,0	11,5	11,0	11,5	10,0	8,5	11,1	10,6	9,1	11,0	9,7	9,3	7,8	8,2	8,8	8,8	9,8	7,4	8,6	7,4	8,7	7,7	6,0	7,7	7,7	7,1	6,3	6,7	8,9	7,7	8,3
	MGST1	8,0	4,9	5,1	7,6	8,9	9,3	10,2	7,7	10,4	9,9	9,2	9,0	8,4	4,7	6,0	10,8	8,5	9,6	10,3	8,8	7,7	8,6	9,1	9,3	7,9	9,9	7,3	6,1	5,0	6,5	6,1	5,9	5,0	4,4	4,4	4,5	3,6	4,3	3,4	4,5	3,7	4,5	4,1	4,2	7,1	3,6	6,9

Supplemental Table 3. List of genes that increased or decreased upon SOX2 overexpression in U-2982 or SFRP2 overexpression in U-2987 or were differentially expressed between U-2982 and U-2987. The first and second column sets show 2444 and 761 genes that were significantly increased or decreased by SOX2 overexpression in U-2982 (>2-fold, p<0.05), respectively. The third and fourth column sets show 1212 and 970 genes significantly increased or decreased by SFRP2 overexpression in U-2987 respectively (>2-fold, p<0.05). The fifth and sixth column sets show 3412 and 1145 genes with significantly different expression levels in U-2987 and U-2982 respectively (>2-fold, p<0.05). The fold change in expression was calculated by comparing the average value from triplicates, and p-values were calculated by t-test of triplicates.

Order	Description	U-2987 vs U-2982 (>2-fold)	*p-value*	Order	Description	U-2982 vs U-2987 (>2-fold)	*p-value*	Order	Description	U-2982 (SOX2 vs control) (>2-fold)	*p-value*	Order	Description	U-2982 (control vs SOX2) (>2-fold)	*p-value*	Order	Description	U-2987 (SFRP2 vs control) (>2-fold)	*p-value*	Order	Description	U-2987 (control vs SFRP2) (>2-fold)	*p-value*
1	COL4A6	4405,0	4,440E-06	1	ADAMTS2	21253,8	1,598E-05	1	FLT1	2234,5	1,985E-06	1	MTSS1	256,8	0,001	1	SFRP2	1254,9	2,429E-06	1	CELF2	2067,8	8,607E-07
2	TYRP1	4384,9	3,721E-06	2	PSG5	1969,5	8,254E-08	2	PTPRZ1	1591,0	4,575E-06	2	BZRAP1	167,2	0,001	2	FLT1	573,9	1,397E-05	2	SGCD	1616,0	1,873E-05
3	VWA5B2	2367,4	1,033E-03	3	SEMA7A	833,1	3,301E-06	3	SUSD5	1439,3	6,620E-07	3	MMP3	159,0	0,000	3	ADAMTS2	325,8	7,352E-03	3	SLC4A4	1124,6	8,328E-04
4	GFAP	2217,2	7,324E-06	4	ITGA11	822,7	3,706E-08	4	SORL1	1230,5	3,693E-09	4	SLC22A15	158,1	0,005	4	COL15A1	224,5	1,926E-07	4	GRIN3A	968,2	6,816E-07
5	CTNND2	1952,0	2,601E-08	5	POSTN	801,9	9,088E-06	5	LAMA2	1199,0	1,357E-06	5	CALB2	127,2	0,001	5	PSG1	197,5	2,645E-06	5	PTPRZ1	716,5	3,153E-05
6	ZNF518B	1892,8	1,459E-05	6	DMKN	800,9	2,017E-04	6	COL4A6	1164,5	4,124E-04	6	NFASC	124,8	0,000	6	F2RL2	176,6	2,364E-04	6	AQP4	634,4	4,316E-05
7	AQP4	1763,8	4,299E-05	7	SPOCD1	786,8	1,078E-04	7	ACSS3	1054,5	1,073E-04	7	LRRN1	121,4	0,000	7	UBE2QL1	165,3	1,821E-04	7	SNTB1	577,0	4,267E-07
8	CNR1	1633,2	1,764E-03	8	INHBA	756,4	1,682E-03	8	ARHGEF4	927,9	6,273E-05	8	DPP4	117,8	0,000	8	GABRE	165,0	1,043E-04	8	GPM6B	545,4	7,901E-04
9	ATRNL1	1374,0	1,143E-06	9	GABRE	597,8	9,077E-05	9	NTRK2	850,7	3,616E-09	9	CHI3L1	101,1	0,000	9	TNFRSF9	158,9	3,490E-05	9	CTNND2	527,6	2,673E-08
10	GPM6B	1300,0	7,869E-04	10	DAAM2	572,6	4,698E-09	10	SYT1	711,8	1,226E-05	10	MFAP4	98,5	0,000	10	MME	157,1	3,185E-05	10	TYRP1	470,9	3,750E-06
11	SERPINB1	1237,0	6,230E-09	11	PTPRM	570,5	5,940E-06	11	ZNF518B	686,0	4,600E-05	11	SUSD4	73,9	0,000	11	INHBA	138,2	1,627E-05	11	A2M	403,9	5,703E-06
12	EHHADH	1175,1	1,928E-05	12	APCDD1L	529,5	2,813E-05	12	GCA	673,2	9,490E-06	12	CH25H	73,7	0,000	12	PSG5	119,3	1,139E-04	12	PRSS35	398,0	2,128E-05
13	VANGL2	958,0	2,159E-06	13	SLC16A3	500,8	3,291E-04	13	PTPRO	630,2	8,397E-03	13	C10orf11	72,1	0,000	13	MEG3	117,0	3,029E-06	13	TMEM108	396,0	1,043E-02
14	ACSS3	923,4	8,685E-05	14	TP53I11	466,1	1,651E-08	14	CD200	563,6	8,535E-05	14	CPA3	66,5	0,008	14	MKX	102,2	6,165E-04	14	ZFP82	381,1	5,417E-03
15	MLC1	785,2	3,970E-05	15	PSG4	461,3	3,806E-05	15	EHF	490,3	4,401E-04	15	LAMA1	64,0	0,000	15	XAF1	96,2	4,262E-03	15	MLC1	336,8	4,000E-05
16	TMOD1	706,3	1,687E-07	16	MEG3	446,0	4,499E-06	16	GRIA4	479,4	7,289E-04	16	IGSF1	58,1	0,002	16	SLC6A6	95,8	8,836E-05	16	DOK5	321,1	1,449E-06
17	SPARCL1	675,8	2,060E-06	17	CXCL12	427,7	7,712E-08	17	ALDH1A1	449,8	5,574E-05	17	GATA4	55,3	0,002	17	LGALS9	94,5	1,998E-05	17	ITIH5	308,0	9,257E-05
18	GATM	659,5	2,781E-04	18	COL1A1	426,0	4,407E-05	18	PREX1	422,2	1,034E-04	18	PTPRN	52,6	0,001	18	SEMA7A	91,4	2,029E-04	18	GRIA4	270,0	6,703E-05
19	ST6GALNAC2	621,4	1,511E-05	19	TNS1	402,0	9,278E-07	19	HLA-DPA1	420,2	1,630E-08	19	UBE2QL1	52,4	0,000	19	PLEKHN1	86,1	1,746E-03	19	PI15	257,7	5,839E-05
20	GCA	612,0	4,227E-05	20	COL6A3	397,4	2,525E-10	20	PDGFRL	412,1	3,772E-06	20	EMR1	50,8	0,000	20	BDKRB1	82,6	2,350E-07	20	GFAP	233,8	7,438E-06
21	CXADR	596,5	1,925E-04	21	UBE2QL1	363,5	9,068E-05	21	COL4A4	391,3	7,783E-05	21	TRIM59	50,8	0,000	21	LYPD6B	76,6	1,055E-06	21	KCNJ2	228,3	3,673E-05
22	NGEF	566,9	3,882E-04	22	TNFRSF9	357,2	3,431E-04	22	IQGAP2	357,0	1,992E-04	22	PTPRC	48,9	0,018	22	CPA3	71,3	8,729E-03	22	MAP6	188,4	6,931E-07
23	TP73	559,0	2,107E-02	23	TGFBI	353,0	5,417E-04	23	MERTK	337,4	1,376E-04	23	HSPB3	47,1	0,000	23	H19	70,5	6,008E-03	23	SOX6	187,9	1,345E-04
24	PTPRZ1	556,1	3,166E-05	24	GYPC	346,5	1,298E-05	24	CD74	335,1	2,853E-06	24	STON2	46,6	0,000	24	IFITM1	70,5	3,014E-03	24	VWA5B2	178,9	1,054E-03
25	GRIA4	515,5	6,617E-05	25	LDB2	329,3	2,163E-05	25	IDO1	328,3	3,926E-04	25	FIBCD1	45,4	0,000	25	IL8	69,6	9,884E-07	25	GRIA3	169,4	9,872E-03
26	FAM184A	504,7	1,708E-04	26	H19	326,4	7,323E-04	26	LY75	323,7	1,048E-05	26	FNDC1	44,7	0,000	26	PARP8	68,3	2,903E-03	26	NALCN	146,1	3,147E-06
27	TPPP3	467,3	2,241E-04	27	PLXDC1	324,3	1,002E-04	27	CD34	317,2	1,783E-03	27	CDH3	42,3	0,002	27	RAC2	67,9	3,705E-05	27	TPPP3	134,0	2,289E-04
28	WDR63	440,5	1,229E-05	28	MMP3	312,6	3,378E-06	28	SERPINB1	309,6	8,010E-05	28	NOG	42,1	0,007	28	CADPS2	67,2	1,868E-04	28	CHRDL1	128,7	3,218E-04
29	ELN	423,8	2,412E-03	29	AMZ1	269,2	7,521E-04	29	CACHD1	309,4	1,586E-06	29	SNAP25	41,4	0,000	29	GYPC	66,7	5,199E-04	29	SNCAIP	127,7	2,711E-05
30	C7orf57	410,7	9,726E-07	30	MLPH	258,4	3,072E-06	30	RTN1	308,3	1,668E-04	30	ADORA1	41,2	0,000	30	SEMA3D	65,8	1,047E-07	30	FAM184A	121,7	1,754E-04
31	C12orf68	386,6	8,274E-04	31	C5orf46	231,2	3,222E-04	31	SOX2	306,4	3,929E-03	31	SORCS2	40,6	0,000	31	CYP24A1	65,6	1,476E-04	31	RBP1	113,2	1,182E-07
32	CYP27A1	361,9	8,510E-08	32	TIMP3	226,6	2,159E-06	32	SLC7A2	295,6	6,637E-05	32	METTL7B	40,0	0,001	32	MPP7	62,2	3,362E-07	32	LRRC2	110,1	9,978E-06
33	SLC40A1	358,8	4,545E-05	33	COL1A2	199,9	4,185E-11	33	CYP27A1	294,4	1,131E-04	33	SYNDIG1	39,7	0,000	33	CA12	61,4	2,610E-04	33	RTN1	107,8	1,307E-03
34	PDE8B	329,4	5,612E-05	34	ARSI	190,4	5,949E-06	34	PLEKHG1	292,1	1,146E-07	34	PTPRE	39,5	0,005	34	LDB2	61,2	8,904E-05	34	PLA2G4A	100,2	5,076E-04
35	HERC5	328,3	7,923E-05	35	SEMA3D	167,2	3,116E-06	35	TMOD1	276,5	1,140E-05	35	DUSP15	39,5	0,029	35	EYA2	60,7	6,039E-04	35	FNDC1	99,4	9,590E-07
36	TIGD7	321,0	6,161E-08	36	OLFML3	165,4	1,300E-03	36	FAM169A	262,8	2,563E-03	36	P2RX5	38,7	0,000	36	PSG4	59,2	2,317E-03	36	SOX11	98,0	2,480E-05
37	FAM169A	312,4	1,983E-05	37	PSG1	164,9	5,329E-05	37	NRCAM	255,0	3,228E-07	37	DCN	38,2	0,000	37	DAAM2	56,3	7,247E-03	37	C7orf57	94,5	1,051E-06
38	SOX2	298,4	2,391E-04	38	RAC2	152,4	1,701E-05	38	NTN1	248,9	4,434E-05	38	ALOX5AP	37,4	0,000	38	HLA-DPB1	55,9	7,201E-05	38	CDH6	88,8	3,631E-05
39	PRSS35	285,8	2,137E-05	39	MN1	148,1	2,127E-04	39	GAP43	247,9	1,161E-06	39	PODNL1	36,7	0,002	39	FPR1	55,3	1,063E-05	39	KCNJ2-AS1	86,7	2,440E-04
40	FGFR3	285,6	1,862E-03	40	LARGE	143,0	1,690E-04	40	TBC1D30	244,0	2,228E-02	40	LHX9	35,7	0,011	40	SLC38A4	53,4	1,068E-06	40	RASSF2	84,4	2,824E-06
41	COL9A3	283,9	3,566E-02	41	SLC6A6	139,8	2,048E-06	41	PRRG4	235,3	6,961E-04	41	ZNF311	35,5	0,006	41	TNFAIP6	52,2	6,111E-05	41	P2RY1	80,5	6,870E-06
42	PPP1R14C	260,2	2,871E-07	42	SH3BP1	137,6	1,018E-03	42	PDE8B	220,7	2,262E-05	42	ATP10A	35,1	0,000	42	PTPRM	51,4	1,625E-08	42	ASPN	74,1	1,478E-04
43	SYT12	258,8	1,534E-03	43	TSPAN18	133,8	1,667E-05	43	CX3CL1	216,8	6,566E-06	43	BGN	34,9	0,000	43	CIITA	49,9	3,327E-04	43	FBLN5	70,8	4,139E-04
44	ABCA3	238,3	2,105E-05	44	TRIM7	133,4	7,696E-04	44	PPP1R9A	212,6	1,888E-05	44	BASP1	32,8	0,000	44	IL1B	49,8	4,344E-03	44	CAPS	68,8	1,292E-02
45	NEBL	234,3	2,938E-05	45	INHBE	133,3	5,062E-05	45	MSTN	212,4	8,071E-07	45	KLC3	32,4	0,001	45	ADAM28	49,8	6,023E-04	45	SALL1	64,5	4,679E-05
46	FAM65B	229,4	2,449E-05	46	MME	123,1	1,785E-06	46	SERPINA5	194,9	3,137E-05	46	ASS1	30,9	0,000	46	HLA-DPA1	49,0	2,724E-07	46	NTRK2	63,7	2,088E-07
47	BCO2	217,2	3,341E-04	47	COL15A1	112,6	1,282E-03	47	EHHADH	191,8	7,523E-03	47	ITIH5	30,4	0,000	47	CXCL12	47,1	3,635E-05	47	WDR63	61,6	1,623E-05
48	ZNF711	213,3	1,816E-08	48	CA12	106,3	7,524E-06	48	GATM	188,8	3,155E-04	48	AGT	30,0	0,000	48	SLC7A2	44,6	1,299E-04	48	NEBL	58,2	3,095E-05
49	SPAG17	208,8	9,394E-06	49	COL4A1	106,1	4,259E-11	49	CXADR	188,2	1,803E-02	49	AMZ1	29,7	0,001	49	PDGFRL	44,3	7,756E-05	49	APC2	57,9	6,991E-03
50	PLEKHG1	207,4	1,036E-05	50	TAGLN	105,8	4,292E-05	50	SCUBE3	186,4	1,185E-04	50	CNTFR	28,5	0,000	50	TMEM171	44,1	5,381E-04	50	ALS2CL	57,4	4,768E-05
51	CLDN1	206,5	2,695E-05	51	CPA3	104,8	7,715E-03	51	TMEM100	182,6	1,514E-09	51	CLIC3	28,4	0,001	51	MLPH	43,5	2,924E-06	51	TPPP	55,7	2,596E-04
52	NTN1	202,9	4,826E-05	52	ALOX5AP	102,3	7,033E-06	52	TCEAL7	171,9	1,983E-06	52	CSPG4	28,2	0,000	52	DMKN	42,0	4,422E-03	52	KLHL26	52,9	3,873E-05
53	LONRF2	200,3	6,569E-06	53	MMP17	101,0	1,236E-02	53	IL8	167,1	7,448E-07	53	RASD2	27,7	0,000	53	IFI27	41,4	7,420E-04	53	CNR1	52,6	1,893E-03
54	CX3CL1	199,8	3,329E-02	54	NID2	94,4	1,425E-06	54	SPARCL1	165,1	1,309E-05	54	CHRDL1	26,9	0,000	54	STAC2	40,5	5,564E-04	54	SOX2	52,5	2,545E-04
55	NTRK2	196,4	1,953E-07	55	SSC5D	93,2	1,550E-04	55	KIF21A	161,3	5,014E-05	55	MAP6	26,9	0,000	55	TSPAN18	40,3	6,987E-05	55	SESN3	52,2	1,294E-04
56	C1orf226	195,3	9,377E-09	56	SORCS2	88,5	4,795E-05	56	GRAMD1C	160,3	9,324E-07	56	FHAD1	26,5	0,005	56	NOD2	39,9	3,853E-05	56	TSHZ3	50,4	1,525E-07
57	ZC2HC1C	193,4	9,409E-04	57	IL1R1	86,9	4,815E-03	57	C8orf4	158,5	5,146E-05	57	CA9	25,5	0,000	57	ITGA11	39,8	4,876E-06	57	FHAD1	49,0	3,907E-03
58	PIEZO2	191,6	3,183E-05	58	TPM2	80,2	1,348E-06	58	ARHGEF26	157,7	9,637E-06	58	SHROOM2	25,2	0,011	58	CCDC85A	39,3	1,258E-04	58	CALCRL	48,9	4,443E-04
59	SLC1A1	183,3	7,728E-04	59	LSP1	78,0	3,200E-04	59	ST6GALNAC2	153,5	3,626E-05	59	FAP	24,8	0,000	59	L1CAM	39,1	2,820E-04	59	TMOD1	46,4	2,112E-07
60	PRRG4	182,2	7,261E-05	60	ARHGAP28	71,9	7,147E-05	60	NES	149,7	1,486E-05	60	RIMS1	24,4	0,007	60	HMHA1	38,5	1,678E-03	60	EHF	46,1	3,777E-02
61	TRIM36	178,0	1,542E-04	61	MATN3	71,4	6,491E-06	61	ZDHHC23	144,5	2,727E-04	61	PLXDC1	24,1	0,000	61	GALNT12	38,5	7,517E-06	61	CADPS	45,8	1,189E-02
62	PPARG	173,4	1,361E-03	62	RCN3	70,9	1,737E-03	62	COL9A3	143,1	2,266E-02	62	SNED1	24,1	0,032	62	AMZ1	37,6	1,046E-02	62	SORBS1	45,5	9,014E-05
63	SYT1	167,0	2,467E-04	63	LOX	69,1	5,557E-06	63	KCNJ8	141,9	1,944E-04	63	ENPP2	23,9	0,000	63	PLXDC1	37,6	1,949E-03	63	CTNNA2	45,0	4,585E-07
64	PPP1R9A	164,9	2,418E-04	64	ZNF521	68,4	4,830E-05	64	ELN	141,3	3,988E-05	64	CREB3L1	23,9	0,000	64	KCNH1	37,2	1,940E-06	64	DCLK1	41,6	8,706E-06
65	KIF21A	161,0	5,213E-05	65	COL13A1	68,0	7,257E-03	65	SOX5	132,8	1,201E-04	65	SEMA7A	23,6	0,000	65	SERPINA1	36,9	1,819E-04	65	NOVA2	41,2	4,319E-06
66	DNALI1	156,7	9,965E-09	66	ADM	67,6	2,090E-06	66	GPM6B	132,3	2,004E-08	66	CORO2B	23,5	0,000	66	SIRPA	36,4	5,368E-07	66	PELI2	41,0	1,721E-05
67	DSC2	154,5	3,231E-08	67	SIRPA	67,2	9,243E-07	67	DSC2	131,7	2,436E-03	67	ADAMTS2	23,2	0,000	67	PTGER4	34,7	2,352E-03	67	C12orf68	41,0	9,031E-04
68	ATP2A1	153,2	1,278E-02	68	PARP8	66,9	1,368E-03	68	SYT12	131,0	2,122E-02	68	TAGLN	23,1	0,000	68	VEGFC	34,5	2,756E-06	68	LIPG	41,0	2,494E-07
69	PRRT1	152,5	1,803E-03	69	CRISPLD2	64,9	4,536E-05	69	SLC40A1	128,7	6,858E-06	69	OXTR	23,1	0,000	69	RARRES2	34,3	1,014E-04	69	MTSS1	40,5	1,297E-03
70	ARHGEF26	151,8	4,179E-04	70	FPR1	64,7	1,503E-06	70	C7orf57	125,4	6,220E-05	70	SOX18	22,7	0,014	70	CPE	33,7	6,649E-07	70	NTN1	39,1	5,243E-05
71	PTPRO	147,4	3,172E-02	71	PTGDS	64,7	1,485E-04	71	PCYT1B	124,7	5,156E-06	71	MICAL2	22,6	0,000	71	ZNF385B	33,6	8,190E-08	71	DNALI1	39,1	1,073E-08
72	CLU	147,4	4,707E-04	72	HMCN1	64,4	1,795E-05	72	FCHO1	122,6	3,682E-03	72	SGCD	22,6	0,000	72	FAM20A	32,5	2,306E-03	72	ARHGAP20	39,1	6,217E-04
73	SOX5	142,6	9,942E-03	73	RTN4RL1	62,5	2,691E-04	73	SLC16A9	120,7	1,292E-05	73	PHLDA3	22,4	0,000	73	CD74	31,8	2,181E-04	73	RPRM	36,6	1,238E-04
74	PCYT1B	141,4	2,754E-05	74	CSPG4	61,7	7,072E-05	74	PRSS35	120,6	3,620E-07	74	SH3RF2	22,3	0,002	74	OAF	31,7	5,128E-04	74	NLGN1	36,3	1,617E-03
75	TUSC3	140,6	2,010E-03	75	MICAL2	61,0	4,823E-05	75	CIITA	119,4	3,368E-03	75	VDR	22,3	0,000	75	ROR2	31,7	7,701E-05	75	PLEKHA7	33,4	1,042E-04
76	KCNJ8	132,1	3,359E-05	76	AMPD3	60,0	4,579E-06	76	FAM65B	118,6	1,459E-04	76	CADPS	22,1	0,000	76	PLAC8	31,4	9,120E-06	76	PCYT1B	32,6	3,104E-05
77	RTN1	131,6	1,295E-03	77	ITGBL1	59,8	3,354E-04	77	SYTL5	116,2	2,009E-05	77	CADM3	21,2	0,000	77	CD163L1	30,3	1,195E-03	77	COCH	32,4	2,308E-04
78	PLEKHA7	131,0	9,453E-05	78	FAP	58,9	2,140E-06	78	GCNT2	111,0	5,199E-06	78	GPRC5C	20,6	0,003	78	LRRC17	29,7	1,904E-05	78	RAB39B	31,1	1,271E-06
79	FAM213A	130,6	5,784E-06	79	CPE	57,7	1,239E-05	79	MCOLN2	110,8	1,340E-06	79	TRIM29	20,4	0,011	79	ARMC4	29,1	7,212E-07	79	NEFL	29,8	2,772E-05
80	MCOLN3	129,4	3,502E-04	80	NREP	56,8	1,029E-03	80	CLDN1	106,3	3,903E-05	80	PLXNA4	20,4	0,000	80	TMC6	29,1	1,296E-04	80	ATP1B2	29,5	1,019E-05
81	DYRK1B	127,7	2,696E-03	81	BDKRB2	56,5	5,207E-05	81	PRRT1	105,2	8,102E-04	81	LARGE	20,1	0,000	81	SCN9A	28,6	1,252E-02	81	PDE8B	29,4	6,554E-05
82	CEBPA	123,9	7,354E-05	82	ANPEP	55,3	8,684E-07	82	JAKMIP2	104,0	8,522E-06	82	GPNMB	19,5	0,000	82	ANKRD30B	28,2	4,208E-08	82	GAP43	28,6	1,137E-07
83	LAMA2	120,3	1,471E-03	83	IGFBP5	52,7	5,689E-08	83	SLC1A1	103,4	5,664E-04	83	DARC	19,4	0,001	83	PPAP2B	27,7	7,204E-07	83	WT1	28,5	1,011E-05
84	FCHO1	116,1	1,576E-02	84	SLC38A4	52,4	1,005E-04	84	CYP24A1	100,8	1,243E-02	84	NPNT	19,4	0,000	84	EDNRA	27,5	4,701E-05	84	CEND1	28,2	3,005E-02
85	GCHFR	115,6	5,948E-04	85	NPR3	51,0	6,229E-04	85	SIX2	99,7	9,352E-05	85	COL10A1	19,1	0,000	85	ISG20	27,0	1,652E-02	85	PTPRD	27,3	5,469E-03
86	HSPA4L	115,5	1,213E-06	86	TMEM171	50,9	9,808E-03	86	TRIM36	99,3	1,492E-04	86	ZNF385D	19,0	0,000	86	C3	26,9	1,303E-06	86	GPC4	26,8	2,288E-06
87	FAM131A	112,8	2,506E-04	87	PHLDA2	49,7	3,411E-03	87	SAMD5	98,4	2,093E-08	87	FAM46B	18,9	0,000	87	PTGFR	26,8	6,051E-06	87	CEBPA	24,3	8,505E-05
88	LY75	109,7	4,869E-06	88	CSTA	49,3	2,264E-03	88	CNR1	97,7	3,902E-04	88	TSPAN18	18,8	0,000	88	TRIM7	26,6	6,993E-03	88	NRCAM	24,2	1,588E-05
89	IQCD	107,1	3,385E-07	89	P2RX5	48,0	3,201E-04	89	CTNND2	95,5	1,542E-03	89	PLAC1	18,6	0,005	89	FAM20C	26,4	3,928E-05	89	PPP4R4	24,1	3,895E-04
90	WNK3	106,5	4,686E-04	90	KCNK6	48,0	2,001E-03	90	FOXF1	90,5	2,114E-05	90	NCALD	18,6	0,036	90	ZNF521	26,2	5,024E-05	90	BAALC	23,8	2,449E-02
91	COL4A4	103,1	1,059E-05	91	PDGFA	47,9	7,857E-06	91	DHRS3	87,5	8,816E-06	91	TPM2	18,0	0,000	91	ADAMTS5	25,9	1,300E-05	91	PPP1R14C	23,4	4,943E-07
92	LIN7A	101,6	4,002E-05	92	ADA	47,2	7,858E-04	92	BBOX1	87,5	7,681E-06	92	NPTXR	18,0	0,000	92	DUSP2	25,7	8,112E-05	92	NLRP1	23,1	5,380E-04
93	CTXN1	100,2	2,690E-02	93	HSPB3	46,6	4,172E-07	93	PPARG	87,3	1,798E-04	93	DMKN	17,9	0,000	93	COL1A1	25,7	6,794E-05	93	LRRC46	22,3	8,193E-06
94	OAS1	98,3	1,199E-06	94	CREB3L1	46,3	1,755E-04	94	SOX6	86,2	1,116E-04	94	ANXA3	17,9	0,002	94	SERPINE1	25,6	9,663E-07	94	BMP6	22,0	4,664E-05
95	THBS4	98,0	5,573E-04	95	THBS2	46,1	5,079E-08	95	CALCRL	84,9	1,940E-03	95	STEAP1	17,9	0,000	95	PRR16	25,2	2,670E-06	95	ANKRD6	21,4	2,835E-03
96	NRCAM	98,0	1,305E-05	96	OXTR	46,0	3,656E-10	96	HAND2	84,1	5,317E-04	96	SEMA3F	17,8	0,000	96	BST2	24,6	1,004E-05	96	LPL	21,3	3,269E-04
97	SLC16A9	93,3	3,459E-04	97	ANGPTL2	45,0	2,567E-05	97	GARNL3	80,6	7,338E-04	97	EPHB2	17,7	0,000	97	OAS2	24,3	4,651E-07	97	PIPOX	21,3	9,914E-06
98	PURG	92,4	1,116E-02	98	ENG	44,0	1,215E-05	98	TP73	79,3	9,856E-04	98	GJB2	17,5	0,008	98	MXRA5	24,2	3,733E-06	98	ANK2	21,3	1,332E-06
99	CD9	90,7	1,144E-06	99	UNC5B	43,7	4,400E-05	99	LONRF2	78,9	1,063E-04	99	PPAPDC1A	17,3	0,000	99	PPM1H	24,0	2,471E-05	99	WNT10B	21,2	6,366E-04
100	CALCRL	90,6	4,283E-04	100	RASD2	43,4	3,877E-05	100	LURAP1L	78,7	2,813E-05	100	SPOCK1	17,3	0,000	100	MMP3	24,0	4,630E-05	100	CYFIP2	20,8	8,185E-04
101	LPHN3	85,6	6,407E-06	101	PLEKHN1	42,1	7,242E-03	101	FAM65C	74,7	2,001E-05	101	NRG1	17,1	0,000	101	AMPD3	23,9	6,081E-04	101	FCHO1	20,5	1,844E-02
102	MERTK	85,0	2,403E-03	102	LMOD1	40,1	5,436E-10	102	HUNK	74,6	5,973E-04	102	APCDD1L	17,0	0,000	102	CSPG4	23,8	3,792E-05	102	EFR3B	20,2	4,703E-04
103	COL21A1	84,7	9,890E-04	103	TRPM4	39,6	6,767E-07	103	TIGD7	74,4	1,918E-02	103	COL3A1	16,9	0,000	103	SCG2	23,8	4,722E-05	103	ZC2HC1C	20,1	1,173E-03
104	RRAGD	84,0	6,076E-05	104	BGN	39,3	5,576E-06	104	VANGL2	73,5	1,655E-05	104	RPRM	16,8	0,000	104	TP53I11	23,6	2,083E-04	104	PLCB1	20,0	6,854E-03
105	TTC25	84,0	5,389E-05	105	CCDC85A	38,9	2,504E-06	105	SDK1	73,0	2,058E-05	105	ANKRD30B	16,6	0,002	105	FIBCD1	23,5	5,512E-04	105	FGFR3	19,6	2,268E-03
106	JAKMIP2	82,9	9,762E-04	106	STMN3	38,6	4,574E-05	106	PLAT	72,7	6,975E-06	106	SALL1	16,5	0,000	106	ADAMTS1	23,2	1,966E-08	106	SPARCL1	19,6	2,723E-06
107	CAPS2	82,8	5,991E-05	107	ADAMTS5	38,5	1,199E-07	107	ST6GALNAC3	71,5	1,377E-05	107	CDH8	16,4	0,000	107	PLAC9	22,9	1,092E-04	107	SEPP1	19,2	3,633E-04
108	C11orf70	81,2	3,064E-05	108	DRD1	38,3	2,998E-05	108	SPINT1	71,4	3,076E-03	108	PTP4A3	16,3	0,012	108	PPAPDC1A	22,8	1,511E-05	108	CD34	18,8	1,246E-05
109	PDE6B	80,8	2,194E-02	109	OAF	36,7	2,866E-04	109	HLA-DMB	70,9	1,647E-03	109	TNS1	16,2	0,000	109	FAM50B	22,6	2,864E-03	109	TMEM231	18,6	2,859E-03
110	C1orf228	80,2	1,271E-03	110	MSC	36,3	3,586E-06	110	MTUS1	70,4	2,082E-04	110	ELAVL2	15,9	0,000	110	MX2	22,6	7,235E-04	110	CDO1	18,2	7,297E-08
111	RNFT2	78,9	3,627E-04	111	RTN2	35,6	1,627E-04	111	CTSH	68,9	5,891E-06	111	RTN4RL1	15,7	0,000	111	CHI3L1	22,6	7,931E-05	111	GATM	18,0	3,470E-04
112	DACH1	78,9	4,243E-06	112	PPAPDC1A	35,4	1,243E-05	112	EDNRA	67,9	4,729E-03	112	L1CAM	15,6	0,000	112	KRT81	22,5	1,319E-03	112	KIRREL3	17,9	1,587E-04
113	C5	77,8	1,987E-05	113	NPTXR	34,5	1,388E-06	113	DENND2D	67,8	1,481E-04	113	MME	15,5	0,000	113	ISG15	22,2	9,704E-04	113	PNMA2	17,8	1,851E-06
114	SNCAIP	77,5	2,761E-05	114	PCOLCE	34,4	6,138E-06	114	C12orf68	67,2	1,260E-05	114	CRLF1	15,3	0,007	114	IER3	21,9	2,622E-03	114	ITGB8	17,6	4,848E-05
115	ODF3B	75,9	2,595E-03	115	COL3A1	34,3	2,462E-08	115	IL1A	65,3	7,191E-04	115	IGDCC4	15,3	0,003	115	ARSI	21,9	2,799E-03	115	GLDC	17,4	1,771E-05
116	RASSF2	75,5	2,866E-06	116	SPOCK1	34,0	1,954E-04	116	SUSD2	65,3	3,217E-04	116	ABI3BP	15,0	0,000	116	IDO1	21,7	7,012E-04	116	FAM107B	17,1	2,508E-04
117	CDO1	75,0	5,465E-08	117	STAC2	33,3	2,529E-05	117	NGEF	65,0	2,599E-04	117	PKDCC	14,9	0,001	117	CPA4	21,4	3,163E-03	117	ECM2	17,1	5,143E-05
118	PIPOX	73,9	8,086E-06	118	KIF26B	33,1	1,354E-06	118	C1orf228	64,4	6,081E-03	118	LOXL2	14,9	0,000	118	KCNE4	21,4	2,226E-07	118	MUC12	17,0	1,452E-04
119	PIH1D2	73,0	3,027E-05	119	IRX3	33,1	9,469E-04	119	WDR63	63,7	7,697E-03	119	FMNL1	14,9	0,002	119	APCDD1L	21,4	1,513E-03	119	CNIH2	16,5	9,843E-05
120	CLDN11	72,9	7,342E-03	120	FIBCD1	32,6	3,252E-06	120	FRMPD4	63,3	9,505E-06	120	EVI2A	14,4	0,007	120	EBF1	21,3	4,126E-03	120	SLC27A6	16,4	1,884E-02
121	CSPG5	72,2	2,893E-04	121	MGC4294	32,5	4,926E-04	121	DYRK1B	62,7	4,181E-04	121	NUDT11	14,4	0,000	121	ADM	21,3	5,762E-06	121	SAMD5	16,2	8,798E-05
122	FRMPD4	70,7	5,017E-06	122	KCNH1	32,2	2,507E-05	122	SLC16A4	62,6	3,726E-09	122	PTPRR	14,4	0,000	122	ANPEP	21,0	7,902E-04	122	SH3BGRL2	16,1	9,664E-05
123	SLITRK5	70,3	2,059E-04	123	SERPINE1	32,0	6,390E-09	123	FGFR3	62,5	8,099E-05	123	OLFML3	14,3	0,002	123	HMCN1	21,0	2,689E-05	123	JAM2	15,7	1,403E-02
124	LOXL4	69,8	3,475E-05	124	PODNL1	31,7	1,996E-03	124	SPAG17	59,6	1,323E-02	124	SFRP2	14,1	0,000	124	IRX3	20,8	2,375E-05	124	PDGFB	15,4	3,732E-04
125	SLC5A3	69,4	7,338E-03	125	EVI2A	31,3	5,883E-03	125	CEBPA	59,2	2,519E-05	125	EFNA5	14,1	0,002	125	CA9	20,5	2,714E-04	125	PDZRN3	15,4	4,436E-06
126	SORL1	68,8	3,957E-02	126	EPHA5	31,0	1,242E-04	126	FAM131A	59,2	1,061E-03	126	RSPO3	14,1	0,000	126	PHLDA2	20,1	1,837E-04	126	UBD	15,0	1,556E-03
127	FZD3	67,4	6,356E-03	127	MYLK	30,4	2,651E-05	127	GALNT12	57,8	1,491E-03	127	KCTD15	14,1	0,000	127	SH3BP1	19,6	2,101E-02	127	ADAMTS14	15,0	2,204E-03
128	MLLT3	66,6	1,339E-05	128	GPRC5C	30,1	2,882E-03	128	RASEF	57,8	6,732E-06	128	PPFIA4	14,1	0,009	128	DRD1	19,5	6,961E-07	128	TSPAN13	14,9	5,661E-05
129	DDO	65,9	6,265E-04	129	SNAI2	30,0	3,260E-09	129	FAM213A	57,7	2,191E-06	129	MYL9	13,9	0,000	129	ACE	19,5	3,807E-03	129	CCDC113	14,5	4,242E-04
130	PHYHIP	64,7	6,545E-03	130	LFNG	29,6	3,734E-05	130	KCNJ2	57,4	5,501E-04	130	RAMP1	13,9	0,000	130	UNC5C	19,5	2,104E-05	130	MAPK8IP1	14,5	2,813E-03
131	ID4	64,7	4,800E-07	131	LOXL2	29,5	5,733E-06	131	NUDT7	56,8	7,728E-06	131	SLC39A4	13,9	0,042	131	DIO2	19,2	6,809E-06	131	METRN	14,4	8,847E-03
132	ZNF528	64,1	1,055E-07	132	COL4A2	29,4	2,415E-06	132	LIN7A	55,3	5,869E-04	132	FAM20A	13,7	0,004	132	NGF	19,2	2,380E-04	132	SYNDIG1	14,2	9,283E-03
133	GLDC	63,9	5,932E-06	133	KCTD15	29,2	7,632E-06	133	OLFML2A	55,1	1,519E-05	133	MAP3K8	13,6	0,001	133	ZNF385D	19,2	1,515E-03	133	TTC25	14,2	7,080E-05
134	KCNJ2	63,8	3,890E-05	134	MMP2	28,8	1,747E-04	134	FAM184A	55,1	8,222E-03	134	LSP1	13,6	0,000	134	CSGALNACT1	19,2	1,130E-04	134	PKNOX2	14,0	3,017E-04
135	TMEM108	63,3	1,094E-02	135	SERPINH1	28,7	1,376E-02	135	METTL7A	54,3	3,667E-02	135	NFE2	13,6	0,002	135	IL1R1	18,9	8,812E-05	135	SFRP4	14,0	1,517E-04
136	CAPS	62,2	1,299E-02	136	SCN9A	28,6	2,652E-03	136	P2RY1	54,0	1,374E-05	136	CXCL12	13,2	0,000	136	AKR1B10	18,7	5,164E-04	136	SLCO2A1	14,0	3,750E-05
137	IQGAP2	62,0	7,648E-04	137	ADORA1	28,6	1,777E-05	137	BMP6	53,7	4,453E-07	137	PRKG1	13,0	0,000	137	LRP5L	18,7	1,545E-02	137	COLEC12	14,0	4,682E-06
138	SOX6	61,1	1,403E-04	138	CD163L1	28,5	6,582E-03	138	ZNF711	53,4	1,207E-03	138	NID2	12,8	0,000	138	LFNG	18,7	2,077E-04	138	NPAS3	13,9	1,606E-06
139	ASRGL1	61,1	1,038E-03	139	KLC3	27,9	9,486E-04	139	PDZD2	53,3	2,696E-03	139	ACTA2	12,8	0,000	139	INPP5J	18,7	6,695E-06	139	MAP1A	13,9	8,169E-05
140	SDK1	60,9	1,094E-05	140	HAPLN3	27,9	6,050E-05	140	RAVER2	53,2	6,758E-07	140	BDKRB1	12,6	0,000	140	IGFBP5	18,7	2,662E-07	140	SVEP1	13,4	9,665E-05
141	ZDHHC23	60,8	3,568E-03	141	GPR124	27,8	6,622E-05	141	CECR1	52,9	3,013E-06	141	KALRN	12,6	0,000	141	MSC	18,5	1,707E-04	141	FNDC4	13,3	1,159E-06
142	RAVER2	59,8	7,104E-05	142	CA9	27,8	1,587E-06	142	ATP2A1	52,1	1,907E-02	142	LUM	12,6	0,000	142	COL12A1	18,4	4,284E-05	142	SLC40A1	13,1	7,437E-05
143	RAB36	59,2	5,946E-06	143	TGM2	27,7	9,171E-05	143	ZC2HC1C	51,9	4,646E-02	143	GPR63	12,5	0,001	143	PDE3A	18,3	9,423E-07	143	CALB2	12,8	4,608E-03
144	PPP2R2B	58,9	6,096E-05	144	COL5A1	27,6	2,723E-04	144	HERC5	51,5	6,995E-04	144	IGFBP3	12,4	0,000	144	TGFBI	18,2	3,674E-04	144	USP2	12,8	1,334E-04
145	PI15	58,4	6,164E-05	145	IFITM2	27,6	1,053E-04	145	PLEKHA7	51,3	2,300E-05	145	MAP1A	12,3	0,000	145	C3AR1	18,1	5,443E-05	145	CDH13	12,5	2,226E-06
146	GRAMD1C	57,6	2,720E-05	146	COL12A1	26,1	2,718E-04	146	RTP4	50,8	4,143E-06	146	COL1A1	12,0	0,000	146	CYP26B1	17,9	1,205E-04	146	HEATR5A	12,4	1,121E-05
147	P2RY1	56,8	8,245E-06	147	CDH8	25,8	1,340E-06	147	CACNG4	49,2	1,266E-05	147	NREP	12,0	0,001	147	EPS8L2	17,7	5,344E-04	147	NEK11	12,4	5,577E-04
148	PRTFDC1	56,5	9,767E-06	148	SYNDIG1	25,8	2,395E-06	148	PDE6B	49,0	5,886E-04	148	STEAP2	11,9	0,000	148	PCDH10	17,4	4,462E-05	148	NACC2	12,2	2,474E-04
149	UCP2	56,4	3,196E-03	149	DYSF	25,8	2,915E-06	149	PLEKHH2	48,9	5,527E-04	149	DRD1	11,9	0,000	149	WNT2	17,2	4,524E-05	149	FAM65C	12,2	4,369E-02
150	CD200	56,2	3,829E-06	150	DACT1	25,4	4,459E-04	150	SPATA13	48,7	3,118E-03	150	LMOD1	11,9	0,000	150	MMP17	17,1	6,988E-03	150	POU3F2	11,9	3,575E-09
151	ANKRD6	56,2	2,529E-03	151	STAT6	25,3	2,229E-06	151	ATL1	48,5	1,370E-06	151	CAMK4	11,9	0,006	151	COL13A1	17,0	2,348E-02	151	PLEKHG1	11,9	1,477E-05
152	GLB1L	55,9	5,628E-07	152	IL1B	25,2	1,606E-02	152	AKAP12	48,4	3,779E-05	152	TBC1D2	11,8	0,002	152	SORCS2	16,9	1,422E-05	152	SMPDL3A	11,9	7,616E-05
153	EFHD1	54,5	1,905E-05	153	PDE5A	24,6	2,252E-03	153	VCAM1	47,7	3,381E-06	153	VSTM2L	11,6	0,006	153	COL1A2	16,8	8,215E-06	153	RALGAPA2	11,6	7,706E-05
154	ZNF75A	54,4	1,424E-04	154	IFITM10	24,5	1,737E-03	154	DKK1	47,7	3,529E-06	154	DGCR5	11,6	0,001	154	GATA2	16,8	1,260E-04	154	LRRC4	11,3	2,922E-05
155	RHPN1	54,1	2,921E-02	155	TNNT1	24,4	7,542E-07	155	ARAP2	47,1	9,879E-06	155	SERPINB7	11,6	0,005	155	PDE5A	16,6	1,532E-03	155	STAT6	11,3	4,629E-03
156	SAMD5	53,5	7,533E-05	156	COL7A1	23,8	5,061E-05	156	CLDN11	46,9	9,426E-03	156	SSC5D	11,5	0,000	156	PDE7B	16,5	8,102E-05	156	EFNB2	11,2	1,816E-08
157	PLEKHH2	53,4	2,173E-05	157	DPP4	23,8	7,946E-06	157	KCNJ2-AS1	46,5	6,532E-03	157	WNT5B	11,5	0,000	157	CREB3L1	16,0	4,562E-05	157	ADAMTS15	10,8	2,775E-05
158	GARNL3	53,2	4,831E-04	158	HTATSF1P2	23,8	1,160E-04	158	VWA5B2	45,9	7,485E-04	158	GPR124	11,5	0,000	158	RTN4RL1	16,0	5,098E-05	158	MOV10L1	10,7	7,364E-03
159	FANK1	53,1	1,970E-05	159	ANGPT1	23,6	7,243E-05	159	XAF1	45,5	3,841E-04	159	ARHGEF5	11,5	0,000	159	IL11	15,6	1,323E-04	159	WDR78	10,6	2,982E-04
160	WDR17	52,4	3,272E-03	160	ZNF385D	23,6	3,872E-04	160	KCNAB3	44,9	4,519E-06	160	NAP1L3	11,5	0,000	160	TSKU	15,5	3,365E-06	160	TFAP2C	10,5	4,684E-06
161	KCNJ2-AS1	52,2	2,698E-04	161	AOX1	22,7	9,815E-07	161	EFNA1	44,9	1,412E-05	161	STARD8	11,4	0,000	161	IL6	15,3	4,564E-05	161	ILDR2	10,5	5,775E-06
162	MCOLN2	51,4	2,150E-04	162	LAPTM5	22,6	1,594E-04	162	TPPP3	44,8	1,244E-03	162	LIMS2	11,3	0,000	162	DPP4	15,3	1,169E-04	162	TMCC2	10,5	3,931E-04
163	MX1	51,3	7,751E-04	163	CAMKK1	22,5	1,220E-04	163	MASP1	44,7	1,228E-02	163	MDFI	11,3	0,000	163	ARHGAP28	15,2	1,345E-03	163	HRCT1	10,4	7,759E-05
164	SLC16A14	50,6	9,591E-06	164	PWWP2B	22,4	4,882E-03	164	CSPG5	44,6	1,098E-02	164	C1orf233	11,3	0,000	164	HMGA2	15,1	8,111E-03	164	ADAMTS16	10,3	3,586E-04
165	CACHD1	48,7	3,827E-05	165	NGF	22,2	7,094E-07	165	WNK3	44,6	1,777E-05	165	JPH2	11,3	0,000	165	UNC5B	15,1	1,320E-05	165	ST6GALNAC2	10,3	2,326E-05
166	HOXD13	48,4	3,895E-06	166	KIAA1199	21,9	5,648E-05	166	AKR1B10	44,3	1,025E-05	166	AFF3	11,2	0,002	166	S1PR5	15,0	2,277E-04	166	CCDC151	10,3	6,627E-04
167	SVIP	47,5	3,036E-05	167	GATA2	21,9	6,774E-04	167	GCHFR	44,0	3,775E-04	167	BACE2	11,2	0,000	167	SNED1	14,9	1,188E-02	167	AP3B2	10,3	2,049E-04
168	GRIN3A	47,1	8,400E-07	168	SCG2	21,8	6,508E-09	168	PRKAR2B	43,1	4,169E-09	168	TPD52L1	11,1	0,001	168	GATA3	14,9	1,484E-04	168	ENHO	10,2	6,774E-06
169	ATL1	46,9	1,735E-05	169	BDKRB1	21,8	9,689E-05	169	EML6	43,0	2,162E-03	169	CTHRC1	11,1	0,000	169	PNPLA3	14,8	9,310E-04	169	PHYHIP	10,0	9,090E-03
170	PNMA2	45,9	1,538E-06	170	PLAC9	21,7	4,149E-02	170	LRP1B	42,8	1,162E-04	170	ALX4	11,1	0,000	170	FOXF1	14,8	1,847E-03	170	FANK1	10,0	6,720E-05
171	CCDC146	44,5	8,613E-06	171	PRSS12	21,2	2,158E-06	171	HEY1	42,5	5,511E-08	171	FAM89A	11,0	0,000	171	LAPTM5	14,7	2,468E-03	171	CLSTN2	9,9	5,982E-04
172	SLC37A1	44,1	2,408E-04	172	SCG5	21,2	3,921E-06	172	ALS2CL	41,9	8,069E-05	172	POSTN	11,0	0,000	172	IGSF1	14,6	3,234E-03	172	RRAD	9,9	1,157E-02
173	FAM84B	43,9	1,718E-07	173	NDRG1	21,0	1,185E-02	173	KLHL13	41,7	3,265E-04	173	PRR16	10,9	0,000	173	PCOLCE	14,3	1,697E-05	173	MMP7	9,7	3,392E-05
174	LRCH2	43,9	5,867E-04	174	CADPS2	20,8	2,772E-05	174	SLC12A2	41,6	2,123E-07	174	SLC7A7	10,9	0,009	174	TNFSF10	14,2	1,266E-06	174	FBXL13	9,6	1,578E-03
175	ITGB4	42,6	2,273E-03	175	PTGER4	20,7	3,675E-05	175	RHOU	41,5	4,940E-06	175	KIF26B	10,8	0,000	175	HAPLN3	14,1	1,422E-03	175	HEY1	9,6	1,940E-05
176	PREX1	41,7	7,234E-06	176	PLAUR	20,7	3,221E-06	176	LRCH2	41,3	2,257E-05	176	PMAIP1	10,5	0,001	176	SLC16A7	14,1	1,756E-05	176	NCALD	9,5	7,509E-05
177	ANK2	41,6	1,086E-06	177	KCNE4	20,5	3,317E-07	177	MT1M	41,2	8,733E-06	177	MN1	10,5	0,000	177	MN1	14,1	8,759E-05	177	SORBS2	9,4	3,663E-05
178	EFR3B	41,6	4,185E-04	178	ADAM12	20,5	1,729E-05	178	AFAP1L2	40,6	6,912E-05	178	SEMA3D	10,4	0,000	178	TNS1	13,9	1,888E-02	178	SH3PXD2A	9,3	5,457E-05
179	KLHL13	41,4	2,241E-05	179	COL5A3	20,4	2,911E-06	179	IL13RA2	40,5	2,705E-06	179	DYSF	10,4	0,000	179	SPINT1	13,8	1,201E-05	179	ST3GAL1	9,3	6,841E-05
180	ATP1B2	41,4	8,651E-06	180	PPFIA4	20,2	8,203E-03	180	DDO	40,0	7,822E-05	180	IRX3	10,3	0,000	180	MTMR9LP	13,8	1,784E-04	180	ZNF436	9,3	9,610E-06
181	IL13RA2	41,3	9,809E-06	181	HMHA1	20,2	4,090E-03	181	PLA2G4A	39,9	4,371E-06	181	ZC4H2	10,2	0,000	181	BARX1	13,7	5,661E-03	181	C22orf23	9,3	4,764E-05
182	FBN2	41,2	1,699E-05	182	GATA3	20,1	4,847E-04	182	COCH	39,1	5,213E-08	182	ARHGAP24	10,2	0,031	182	IL15RA	13,7	1,120E-03	182	RAB6B	9,1	1,278E-05
183	ARAP2	41,1	5,563E-04	183	GLI2	19,9	2,980E-05	183	PDGFB	39,0	8,445E-06	183	IFITM10	10,2	0,000	183	DYSF	13,6	5,453E-04	183	MAN1C1	9,0	8,854E-06
184	SERPINA5	40,4	1,471E-03	184	L1CAM	19,8	1,199E-04	184	CD9	38,5	5,631E-07	184	KIAA1199	10,1	0,000	184	IFI6	13,6	1,454E-02	184	CLU	8,9	7,274E-04

	A	B	C	D	E	F	G	H	I	J	K	L	M	N	O	P	Q	R	S	T	U	V	W	X
187	185	DENND2D	40,0	3,267E-03	185	ADAP1	19,5	6,077E-04	185	PRKCDBP	38,3	2,700E-03	185	MMP24	10,1	0,000	185	EFNA5	13,5	1,014E-02	185	MMP15	8,9	8,435E-04
188	186	SPINT1	39,2	6,065E-03	186	CPS1	19,5	2,353E-04	186	SLC5A3	38,1	5,026E-04	186	USP2	9,9	0,005	186	AKR1C1	13,5	3,000E-02	186	RND2	8,9	3,095E-04
189	187	TEX9	38,9	9,495E-04	187	EBF1	19,5	1,465E-05	187	EFHD1	38,1	2,250E-04	187	SIRPA	9,8	0,000	187	TMEM130	13,5	4,112E-05	187	CKB	8,7	3,830E-03
190	188	KIAA1407	38,6	1,424E-06	188	SYTL2	19,1	4,724E-05	188	PRKAA2	37,9	3,043E-06	188	SCIN	9,8	0,008	188	SERPINB7	13,4	7,009E-05	188	ITGB4	8,7	3,284E-03
191	189	HAND2	38,1	3,964E-03	189	LCP1	19,1	3,899E-06	189	AIF1L	37,8	3,482E-07	189	LPPR3	9,8	0,043	189	ELAVL2	13,4	7,937E-07	189	CDNF	8,5	2,445E-02
192	190	CTSS	37,9	2,272E-06	190	PARVB	19,1	3,122E-05	190	PRSS12	37,6	1,070E-07	190	CXXC5	9,7	0,000	190	MT1M	13,4	2,244E-03	190	REEP1	8,5	7,348E-05
193	191	ITIH5	37,7	1,018E-04	191	ECM1	19,0	1,106E-06	191	PANX2	37,1	2,580E-03	191	PLCB4	9,7	0,000	191	ADA	13,3	7,267E-04	191	GAB2	8,5	1,947E-05
194	192	GDPD1	37,3	6,617E-04	192	KALRN	18,8	2,074E-04	192	MSLN	35,5	2,857E-03	192	KCNH1	9,6	0,000	192	NPTXR	13,3	1,604E-06	192	FGF1	8,4	6,245E-04
195	193	DHRS3	37,0	6,522E-08	193	MIR210HG	18,8	1,516E-03	193	ARHGEF37	35,2	2,681E-08	193	ANGPT1	9,6	0,000	193	HUNK	13,3	3,919E-03	193	TMTC2	8,4	1,030E-04
196	194	GAP43	36,8	6,261E-08	194	LOXL1	18,7	1,765E-03	194	HSPA4L	34,5	3,938E-05	194	SYTL2	9,6	0,000	194	DISC1	13,2	6,553E-04	194	RNF19A	8,2	1,661E-05
197	195	B3GNT5	36,6	6,928E-03	195	RSPO3	18,6	4,000E-05	195	ALDOC	34,4	9,188E-07	195	COL5A3	9,6	0,000	195	IL15	13,1	1,474E-04	195	RPGR	8,2	2,592E-05
198	196	MASP1	35,7	1,681E-02	196	ADRA1D	18,6	4,718E-04	196	FGF12	33,9	5,054E-04	196	CPE	9,5	0,000	196	DUSP4	13,1	2,208E-05	196	BCL2	8,2	8,196E-06
199	197	TMTC2	35,5	5,001E-05	197	ITGA5	18,6	1,998E-06	197	RGL3	33,8	5,676E-03	197	CCDC85A	9,5	0,000	197	P2RX5	13,0	2,104E-04	197	CHN2	8,2	5,590E-04
200	198	SAMD15	35,2	8,016E-05	198	C1QTNF1	18,5	2,429E-03	198	RAB38	33,5	8,572E-05	198	APLN	9,5	0,007	198	SLC15A3	12,9	1,406E-04	198	GJA1	8,2	1,013E-05
201	199	GPR162	34,9	8,515E-03	199	FRMD6	18,2	2,976E-08	199	SLITRK5	33,5	2,784E-04	199	PRICKLE1	9,4	0,003	199	PRDM11	12,8	7,471E-04	199	RFX3	8,0	1,390E-05
202	200	WNT2B	34,6	2,871E-05	200	HLA-DPB1	18,0	7,603E-03	200	PPP2R2B	33,2	1,151E-03	200	EFNB1	9,4	0,000	200	PWWP2B	12,8	4,353E-04	200	SCUBE3	8,0	1,327E-03
203	201	OAS2	34,4	4,926E-04	201	KDELR3	17,4	2,401E-06	201	GPRC5B	32,9	7,272E-06	201	FAS	9,2	0,003	201	JUP	12,7	4,552E-04	201	LONRF2	7,9	1,239E-05
204	202	FAM69B	34,3	1,473E-03	202	HCN2	17,0	2,271E-05	202	PDGFD	32,8	5,443E-07	202	ADM	9,2	0,000	202	ADRA1D	12,6	1,258E-03	202	CCDC146	7,8	9,983E-06
205	203	MSTN	34,3	7,123E-05	203	ADAMTSL5	16,8	2,117E-05	203	LRRC8B	32,8	4,793E-04	203	P2RX6	9,2	0,006	203	OXTR	12,5	2,295E-07	203	ITPKB	7,7	2,891E-06
206	204	VCAM1	34,2	7,673E-07	204	VDR	16,7	3,865E-09	204	MCOLN3	32,5	2,826E-04	204	AHNAK2	9,0	0,000	204	CLDN7	12,4	5,319E-04	204	IQCG	7,7	7,688E-05
207	205	DKK1	33,6	7,478E-07	205	ACTG2	16,5	6,360E-05	205	IL20RA	32,4	5,378E-05	205	THRB	8,9	0,027	205	IFIT1	12,4	4,685E-06	205	PMP22	7,7	4,839E-06
208	206	WDR78	33,1	2,117E-04	206	CYP26B1	16,5	4,485E-05	206	LRRC2	31,6	9,467E-07	206	TNFRSF21	8,9	0,000	206	ANGPTL2	12,3	1,256E-04	206	DUSP18	7,6	1,911E-05
209	207	AMDHD1	33,1	1,721E-03	207	PCDH7	16,3	2,818E-04	207	MED12L	31,5	2,105E-04	207	MEGF6	8,8	0,002	207	ALOX5AP	12,3	4,939E-03	207	ITGBL1	7,5	3,121E-03
210	208	KCNAB3	32,8	1,296E-03	208	FRAS1	16,0	1,174E-04	208	ACE	31,4	1,352E-07	208	PCDHGC5	8,8	0,001	208	KCNK6	12,3	3,503E-04	208	TRAC	7,5	1,118E-05
211	209	METTL7A	32,8	6,608E-07	209	DOCK6	15,9	1,062E-05	209	FKBP11	31,2	6,045E-05	209	RTN4R	8,7	0,023	209	NFKBIZ	12,3	1,012E-06	209	SLC5A3	7,4	1,187E-02
212	210	LRRC46	32,5	1,241E-05	210	PGF	15,8	7,818E-04	210	ATP1B2	31,0	1,563E-04	210	BDKRB2	8,6	0,000	210	ARHGAP6	12,3	8,629E-04	210	EML6	7,4	6,498E-03
213	211	MYEF2	32,4	2,880E-05	211	IL32	15,7	1,400E-08	211	MLLT3	30,5	2,197E-05	211	LOXL1	8,5	0,002	211	GPR85	12,2	3,537E-03	211	KIF9	7,4	3,585E-05
214	212	ZNF620	32,2	1,698E-02	212	PDLIM7	15,6	5,951E-04	212	RASD1	30,4	5,029E-05	212	HS3ST3A1	8,4	0,005	212	PODNL1	12,2	9,699E-04	212	CRYAB	7,4	1,822E-03
215	213	SELENBP1	32,0	1,027E-06	213	NXPH4	15,4	3,564E-04	213	CCDC148	29,9	5,207E-03	213	STMN3	8,4	0,000	213	PLAUR	12,2	2,185E-04	213	CCDC40	7,3	1,625E-05
216	214	MYCBPAP	31,1	1,106E-02	214	CADPS	15,3	4,163E-05	214	PURG	29,9	3,274E-03	214	PRELID2	8,4	0,000	214	PPFIBP2	12,1	3,173E-03	214	KCNB1	7,2	6,443E-05
217	215	NHSL1	31,1	2,080E-06	215	ACTA2	15,2	1,263E-07	215	THBS4	29,7	1,905E-03	215	SPOCD1	8,4	0,000	215	COL5A1	12,0	5,707E-05	215	ITGB3	7,2	8,304E-09
218	216	ZNF542	30,8	1,952E-02	216	LRP5L	15,0	2,085E-03	216	NOD2	29,5	5,505E-03	216	TMEM56	8,4	0,004	216	HLA-DMB	11,8	2,320E-04	216	IGSF3	7,2	1,085E-06
219	217	C9orf116	30,7	7,607E-05	217	MYL9	15,0	2,411E-06	217	CXCL14	27,9	1,151E-05	217	SLC22A4	8,3	0,000	217	TIMP3	11,7	8,849E-07	217	SPAG1	7,2	2,430E-05
220	218	ILDR2	30,4	4,414E-06	218	MEIS3	14,7	3,038E-05	218	PIPOX	27,9	5,917E-08	218	DGAT2	8,3	0,001	218	TRIM38	11,6	1,632E-06	218	PDE4B	7,1	1,404E-05
221	219	CKB	30,4	2,683E-03	219	MEX3B	14,5	3,676E-04	219	AP1S3	27,5	3,143E-04	219	SERPINA1	8,3	0,004	219	GAS1	11,6	1,948E-05	219	CELSR2	7,1	5,974E-05
222	220	RFX3	29,9	1,134E-05	220	AKR1C3	14,4	1,333E-06	220	IL22RA1	27,2	2,198E-03	220	TUSC3	8,3	0,002	220	RAB20	11,6	1,414E-06	220	EFNB1	7,1	3,882E-04
223	221	SVIL	29,7	9,589E-05	221	FAM20C	14,3	6,702E-05	221	C1orf226	26,7	8,736E-04	221	STS	8,3	0,000	221	IFITM2	11,5	2,924E-03	221	DAB2IP	7,1	2,082E-03
224	222	SLC12A7	29,6	5,677E-04	222	FZD2	14,2	1,142E-03	222	SNCAIP	26,6	1,847E-04	222	HMOX1	8,3	0,000	222	HTATSF1P2	11,5	2,427E-02	222	REEP2	7,0	1,645E-03
225	223	CECR1	28,9	4,479E-06	223	PDGFRB	14,2	1,200E-04	223	TMEM220	26,5	2,824E-02	223	FPR1	8,2	0,000	223	ACTG2	11,5	5,388E-04	223	SLC16A9	6,8	6,130E-04
226	224	ADAMTS15	28,4	2,154E-05	224	B4GALNT4	13,9	6,985E-04	224	NGFR	26,3	3,080E-06	224	DUSP2	8,2	0,001	224	RASD2	11,4	3,091E-03	224	ARRDC4	6,8	6,596E-05
227	225	TMEM170B	28,2	9,706E-05	225	ATP10A	13,8	9,600E-06	225	SVIL	26,1	6,677E-07	225	INHBE	8,2	0,000	225	TSPAN15	11,4	6,411E-06	225	FAM171B	6,8	9,800E-06
228	226	POU3F2	28,2	4,009E-09	226	DIRAS1	13,7	1,657E-04	226	RNF157	26,1	2,778E-05	226	A2M	8,2	0,000	226	BAIAP2L1	11,4	6,553E-06	226	NTRK3	6,7	5,711E-03
229	227	KCNB1	28,1	3,881E-05	227	CDKN1C	13,6	1,186E-03	227	ZDHHC11	26,0	2,745E-03	227	STXBP6	8,2	0,000	227	NT5E	11,4	8,436E-06	227	APLP1	6,7	7,451E-06
230	228	LRRC34	28,0	4,613E-06	228	BARX1	13,5	6,255E-03	228	B3GNT5	25,8	1,500E-04	228	HSPB8	8,1	0,000	228	IFIT3	11,4	2,413E-06	228	AXIN2	6,7	1,333E-04
231	229	CCDC40	27,9	1,228E-05	229	FBN1	13,4	3,741E-07	229	SCG2	25,2	1,831E-07	229	DMD	8,1	0,001	229	RGS16	11,3	8,191E-04	229	HOXD13	6,7	7,745E-06
232	230	C16orf93	27,5	1,788E-03	230	UNC5C	13,4	3,947E-06	230	SLC12A7	25,1	5,086E-05	230	CLSTN2	8,0	0,000	230	LIF	11,3	2,161E-05	230	BCO2	6,7	1,465E-03
233	231	CCDC148	27,4	1,162E-02	231	EGR2	13,4	6,424E-06	231	ADAMTS15	25,0	1,379E-06	231	FGF1	7,9	0,005	231	SAMD11	11,2	2,493E-04	231	RRAGD	6,7	1,073E-04
234	232	CPVL	27,2	1,231E-05	232	WNT2	13,1	1,188E-02	232	WDR66	24,7	1,829E-05	232	FZD2	7,8	0,002	232	MLKL	11,2	1,663E-05	232	AK5	6,7	1,045E-04
235	233	ITGB8	27,1	4,342E-05	233	CLMP	13,0	7,160E-06	233	LOXL4	24,4	2,592E-04	233	C4orf48	7,8	0,037	233	CDK18	11,2	1,254E-03	233	GPR162	6,7	1,368E-02
236	234	PHLPP1	27,1	2,804E-07	234	ANKDD1A	13,0	1,121E-03	234	CACNA2D1	24,4	9,403E-05	234	TNNT1	7,7	0,000	234	TPD52L1	11,2	6,005E-05	234	TSNAXIP1	6,7	1,362E-04
237	235	TP53	27,0	1,538E-05	235	STC2	13,0	6,125E-07	235	ABCA3	23,8	7,795E-03	235	ITGA7	7,7	0,001	235	CASP10	11,1	9,082E-04	235	LRRC48	6,7	2,100E-03
238	236	ZNF677	26,5	9,013E-05	236	GJB2	12,9	8,921E-03	236	IQCD	23,7	5,944E-04	236	NGF	7,7	0,000	236	PTGER2	11,1	6,784E-06	236	CCDC153	6,6	1,213E-02
239	237	SYNE2	26,4	8,988E-04	237	MTMR9LP	12,8	8,009E-04	237	ZNF620	23,3	5,478E-03	237	SRPX	7,7	0,000	237	TGM2	10,9	4,559E-04	237	WWC1	6,5	6,358E-04
240	238	BATF3	26,3	5,074E-03	238	SNED1	12,8	3,563E-02	238	NHSL1	23,3	6,063E-04	238	CPM	7,6	0,000	238	IL12A	10,9	2,042E-02	238	ARHGEF4	6,5	9,727E-06
241	239	IFI44L	26,3	7,433E-05	239	MFAP4	12,7	2,241E-06	239	HPSE	23,1	3,526E-06	239	SIK1	7,5	0,000	239	COL3A1	10,8	1,940E-06	239	DPYSL2	6,5	3,051E-06
242	240	HES6	26,1	1,451E-03	240	ANKRD30B	12,6	2,464E-03	240	CCDC102B	22,5	3,296E-07	240	PPP1R13L	7,5	0,001	240	TMEM200A	10,7	4,887E-05	240	LRP1B	6,5	2,650E-03
243	241	XK	26,1	1,341E-04	241	CORO2B	12,5	1,896E-08	241	JUP	22,3	3,592E-05	241	MEST	7,5	0,000	241	PDCD1LG2	10,6	1,155E-05	241	CXADR	6,5	5,522E-04
244	242	ID1	26,1	1,315E-02	242	LIMS2	12,4	3,682E-05	242	EEF1A2	21,8	9,886E-06	242	NPDC1	7,5	0,003	242	MATN3	10,6	4,146E-04	242	ABCA5	6,5	2,699E-03
245	243	C7orf31	25,8	5,629E-05	243	MFAP5	12,4	1,930E-05	243	PEG10	21,8	2,446E-07	243	CSF1R	7,5	0,002	243	AKR1C3	10,5	9,048E-04	243	C9orf116	6,5	1,408E-04
246	244	CACNG4	25,3	3,610E-05	244	SERPINA1	12,4	2,984E-03	244	LPHN3	21,6	1,242E-03	244	DEPTOR	7,5	0,000	244	NOTUM	10,5	2,100E-03	244	HES6	6,4	2,364E-03
247	245	NLRP1	25,2	5,293E-04	245	FSCN1	12,3	7,017E-04	245	C7orf31	21,6	3,628E-05	245	RHCE	7,4	0,035	245	SUSD5	10,4	1,957E-05	245	ENDOD1	6,4	9,254E-05
248	246	SALL2	25,2	3,773E-07	246	MDFI	12,3	4,710E-07	246	CGN	21,3	1,012E-03	246	GAD1	7,3	0,000	246	FGF5	10,4	2,024E-04	246	CSPG5	6,4	6,534E-04
249	247	SPATA13	25,1	1,339E-05	247	PKDCC	12,2	5,793E-04	247	CXCL3	21,2	1,282E-02	247	C3	7,3	0,000	247	EPHA5	10,4	3,277E-04	247	LIN7A	6,4	3,289E-04
250	248	SPTBN2	25,0	1,852E-03	248	FAM212A	12,1	1,466E-05	248	RNFT2	21,0	1,434E-02	248	MOXD1	7,3	0,000	248	IL4R	10,3	4,104E-06	248	FAM182B	6,3	3,940E-03
251	249	AKAP12	24,9	4,080E-05	249	GPR85	12,1	4,167E-02	249	VWA5A	21,0	4,689E-05	249	CPVL	7,3	0,000	249	KCTD15	10,3	5,817E-04	249	CPVL	6,2	2,162E-05
252	250	ACHE	24,7	6,091E-03	250	KCNMA1	12,1	9,004E-04	250	CITED4	20,9	7,624E-04	250	TRIM9	7,2	0,000	250	CSF1R	10,3	6,774E-06	250	DNAH1	6,2	6,791E-03
253	251	RND2	24,5	1,610E-04	251	HMOX1	12,1	4,695E-06	251	MT1F	20,9	9,283E-03	251	SPARC	7,1	0,003	251	DUSP5	10,3	2,881E-04	251	LETM2	6,1	1,649E-03
254	252	SRGAP3	24,3	7,357E-05	252	FXYD5	12,0	1,739E-05	252	ALDH1A3	20,6	2,210E-04	252	KCNN4	7,1	0,000	252	RNF217	10,2	2,766E-03	252	ARHGEF26	6,1	8,146E-04
255	253	PRKAR2B	24,2	5,994E-05	253	CSF1R	12,0	1,155E-03	253	WNT2B	20,6	1,430E-05	253	TYMP	7,1	0,037	253	GPR124	10,2	3,626E-05	253	RHOJ	6,1	4,609E-06
256	254	SCUBE3	24,1	8,633E-04	254	NAV3	11,9	8,826E-06	254	CLU	20,5	6,585E-03	254	PRUNE2	7,1	0,000	254	LOXL1	10,2	3,080E-05	254	CNTFR	6,1	1,392E-02
257	255	DEPDC4	23,9	2,883E-04	255	EDIL3	11,9	8,760E-06	255	DNAJC12	20,4	8,369E-05	255	THBS2	7,0	0,000	255	F2R	10,1	4,135E-07	255	JAKMIP2	6,0	2,212E-03
258	256	AGBL3	23,8	2,190E-05	256	COL5A2	11,8	2,515E-07	256	GSTM3	20,4	3,283E-04	256	PLAC8	7,0	0,001	256	CXCL14	10,1	4,129E-06	256	SOX5	6,0	1,810E-02
259	257	RAB26	23,7	3,560E-03	257	PXDN	11,8	5,296E-09	257	FAM167A	20,4	2,526E-07	257	DHRS2	7,0	0,034	257	ACSL5	10,1	3,333E-05	257	FAM84B	6,0	3,270E-07
260	258	CCDC113	23,7	3,697E-04	258	ADAMTS6	11,7	1,945E-03	258	PPP1R14C	20,3	1,190E-05	258	GPAM	6,9	0,000	258	MMP24	10,1	7,942E-07	258	ATRNL1	6,0	2,473E-04
261	259	STXBP2	23,6	1,427E-05	259	GPC6	11,6	2,415E-06	259	SLC2A13	20,3	1,867E-06	259	FAM198B	6,9	0,000	259	CDH8	10,0	7,620E-04	259	LPHN3	6,0	1,364E-05
262	260	EGF	23,6	1,341E-03	260	TBKBP1	11,5	1,706E-02	260	CAPS2	20,2	1,016E-04	260	AEBP1	6,9	0,008	260	WNK4	10,0	2,051E-03	260	EPHA4	5,9	2,584E-04
263	261	EHF	23,4	4,108E-02	261	DNAJC15	11,4	5,643E-07	261	DOK5	19,9	2,954E-05	261	VGLL3	6,8	0,007	261	MDFI	10,0	7,820E-04	261	MLF1	5,9	3,506E-04
264	262	SYBU	23,3	5,903E-03	262	LUM	11,2	3,305E-05	262	MGP	19,9	3,686E-02	262	TSPAN10	6,7	0,001	262	PTGDS	9,9	3,780E-03	262	TRNP1	5,9	1,021E-04
265	263	JAM2	23,3	1,299E-02	263	MFAP2	11,2	1,715E-04	263	SEMA3A	19,8	1,091E-05	263	SLC7A8	6,7	0,000	263	TMEM140	9,9	2,561E-06	263	LRTOMT	5,9	2,338E-03
266	264	APOM	23,2	3,206E-04	264	FMNL1	11,2	1,725E-03	264	CDO1	19,7	2,107E-05	264	ATP1B1	6,7	0,000	264	PPARG	9,7	4,016E-07	264	SAMD15	5,9	1,612E-04
267	265	STK33	23,2	4,777E-04	265	AXL	11,1	2,748E-07	265	BATF3	19,7	6,534E-04	265	IL4I1	6,7	0,001	265	PLEKHF1	9,7	2,696E-03	265	MIR600HG	5,9	1,469E-02
268	266	NUDT7	23,2	2,335E-04	266	P4HA2	11,1	1,777E-06	266	GMPR	19,6	2,092E-06	266	CERS4	6,7	0,000	266	DAPK1	9,7	1,031E-06	266	IL20RA	5,9	6,216E-03
269	267	LRRC8D	23,0	2,607E-04	267	SFRP2	11,0	2,337E-04	267	TBX3	19,4	3,720E-07	267	C5orf46	6,6	0,001	267	IL7R	9,7	1,660E-05	267	HAND2	5,8	7,459E-03
270	268	TSNAXIP1	22,9	4,707E-05	268	KCNS3	10,9	2,140E-04	268	MOV10L1	19,2	1,598E-03	268	RIN1	6,6	0,002	268	FOSL1	9,7	5,810E-05	268	DSC2	5,8	8,882E-08
271	269	GATS	22,7	4,517E-08	269	INPP5J	10,9	2,457E-03	269	XK	19,1	9,340E-06	269	ZNF503	6,6	0,002	269	SCN8A	9,6	2,634E-03	269	MNS1	5,8	8,667E-05
272	270	SHBG	22,7	3,332E-03	270	SNHG5	10,8	9,518E-06	270	TNFRSF1B	18,9	1,525E-05	270	SAMD11	6,6	0,006	270	CSTA	9,6	5,499E-03	270	IFT57	5,7	2,242E-05
273	271	TCEAL7	22,3	8,914E-04	271	PDE7B	10,8	2,186E-03	271	SELL	18,9	4,510E-02	271	TRIB3	6,5	0,000	271	PGF	9,6	4,939E-05	271	KIF17	5,6	9,139E-05
274	272	E2F5	22,3	2,191E-03	272	PPAP2B	10,7	1,244E-04	272	OAS1	18,5	1,807E-04	272	SLC38A4	6,4	0,000	272	FZD2	9,5	1,705E-04	272	SALL2	5,6	7,005E-06
275	273	CNIH2	22,2	9,463E-05	273	EPS8L2	10,7	2,152E-02	273	RHOJ	18,4	1,523E-06	273	COL4A1	6,4	0,000	273	KALRN	9,5	4,061E-04	273	B9D2	5,6	1,161E-04
276	274	MUC12	21,9	1,266E-04	274	FBLIM1	10,7	3,159E-04	274	RAB26	18,4	1,442E-02	274	PDLIM7	6,4	0,001	274	WNT5B	9,5	1,659E-03	274	PLTP	5,6	1,277E-03
277	275	RIMKLB	21,8	1,674E-06	275	PLEKHO2	10,7	2,130E-04	275	TLR1	18,2	3,153E-05	275	COL8A2	6,3	0,000	275	LOXL2	9,4	1,820E-05	275	SLIT3	5,6	1,888E-04
278	276	RHPN2	21,8	4,223E-05	276	KCNN4	10,7	4,834E-05	276	LRP4	18,1	5,891E-07	276	ZNF571	6,3	0,031	276	KCNIP3	9,4	1,875E-03	276	CORO2B	5,6	7,221E-04
279	277	MED12L	21,7	2,346E-03	277	RAB33A	10,7	6,382E-04	277	GRIN3A	17,6	1,941E-05	277	DAAM2	6,3	0,000	277	SSC5D	9,2	1,705E-03	277	ENPP2	5,5	3,667E-05
280	278	SEMA3A	21,5	1,867E-03	278	C3AR1	10,7	1,626E-02	278	FBLN1	17,5	7,337E-05	278	MAGI1	6,3	0,002	278	GLI2	9,2	1,706E-02	278	ATP1B1	5,5	5,556E-05
281	279	C2orf81	21,1	7,335E-03	279	CALB2	10,6	2,035E-03	279	CD83	17,5	1,161E-05	279	SERPINH1	6,3	0,022	279	ELL2	9,2	1,902E-07	279	BEX1	5,5	2,006E-06
282	280	CCDC96	21,0	2,044E-04	280	TSKU	10,6	1,381E-05	280	KRT7	17,2	4,732E-02	280	RAB33A	6,2	0,002	280	SNAP25	9,2	4,242E-05	280	DARC	5,4	3,289E-05
283	281	TPPP	21,0	2,964E-04	281	MMP11	10,4	5,265E-04	281	NEFL	17,1	2,958E-07	281	FAM84B	6,2	0,000	281	SNAI2	9,1	1,861E-05	281	S100A4	5,4	4,388E-05
284	282	SIX2	20,9	3,454E-03	282	NEO1	10,4	6,465E-06	282	LRRC8D	16,8	1,632E-03	282	FRAS1	6,2	0,000	282	FST	9,1	7,798E-04	282	SYNE1	5,4	1,097E-03
285	283	ARMC2	20,7	3,448E-05	283	ACE	10,2	1,865E-02	283	CDH6	16,8	4,684E-06	283	ALPK2	6,1	0,000	283	LARGE	9,1	2,530E-05	283	SYNC	5,4	1,889E-04
286	284	RRAD	20,3	9,515E-03	284	EFEMP2	10,0	1,143E-03	284	PPL	16,8	7,628E-07	284	ARHGAP22	6,0	0,003	284	EVI2B	9,1	4,859E-05	284	KIAA1407	5,3	8,215E-06
287	285	EML6	20,3	4,753E-03	285	PDCD1LG2	10,0	3,108E-06	285	TIMP4	16,6	5,133E-06	285	GPC1	6,0	0,001	285	IFIH1	9,0	2,929E-06	285	FAM65B	5,3	5,549E-05
288	286	FBLN1	20,3	2,530E-04	286	SLC7A7	9,9	1,034E-02	286	ODF3B	16,5	2,383E-02	286	MSC	6,0	0,000	286	ZC3H12A	9,0	1,963E-05	286	CRYGS	5,3	2,570E-03
289	287	PPARGC1A	20,1	1,218E-07	287	NOTCH3	9,9	2,171E-04	287	RND2	16,5	3,591E-05	287	TRIM7	5,9	0,002	287	MICAL2	9,0	2,606E-05	287	SLC16A14	5,3	1,047E-04
290	288	SUSD5	20,0	1,596E-02	288	SPARC	9,9	2,575E-03	288	ADAM28	16,4	1,692E-04	288	IER5L	5,9	0,042	288	C11orf45	9,0	3,589E-02	288	STXBP6	5,3	3,657E-04
291	289	SPAG1	20,0	1,631E-05	289	EFNA5	9,8	3,227E-03	289	SCD5	16,3	1,613E-07	289	ADAMTSL1	5,8	0,008	289	ST3GAL6	9,0	1,972E-06	289	S1PR3	5,3	2,936E-06
292	290	C17orf97	19,8	9,190E-04	290	PDE4A	9,8	3,218E-04	290	CTNNA2	16,3	9,235E-05	290	RRAD	5,7	0,000	290	OLFML3	8,9	1,250E-04	290	PURG	5,3	2,179E-02
293	291	CGN	19,8	3,037E-05	291	F2RL2	9,8	3,154E-04	291	GPR137C	16,2	1,831E-05	291	CHN2	5,7	0,001	291	TRIP6	8,9	2,327E-04	291	FAM167A	5,2	4,700E-07
294	292	NES	19,7	4,000E-05	292	GLS	9,8	3,860E-06	292	SERPINI1	16,2	4,726E-04	292	GLS	5,7	0,000	292	GMPR	8,9	5,145E-04	292	SPATA6	5,2	2,924E-04
295	293	ALDH5A1	19,6	3,371E-06	293	AGT	9,7	6,027E-06	293	GPR126	15,9	5,815E-05	293	SPEG	5,6	0,000	293	TBC1D2	8,8	3,056E-05	293	TUBA1A	5,2	3,686E-02
296	294	A2M	19,6	7,005E-06	294	HTRA1	9,7	9,672E-07	294	OAS2	15,9	1,001E-06	294	FGFR2	5,6	0,000	294	ITGA5	8,7	1,245E-04	294	IGDCC4	5,1	4,431E-03
297	295	DMGDH	19,5	1,425E-05	295	RIMS1	9,7	9,383E-03	295	MLC1	15,8	9,314E-04	295	PIWIL4	5,6	0,006	295	PPFIA4	8,7	6,995E-05	295	LPHN2	5,1	5,173E-04
298	296	GPRC5B	19,4	2,639E-05	296	ADAMTS12	9,6	4,285E-05	296	FAM124A	15,8	6,634E-06	296	ARSI	5,6	0,000	296	CILP2	8,7	1,023E-03	296	TCP11L2	5,1	9,887E-07
299	297	SORBS1	19,3	1,227E-04	297	FKBP7	9,6	2,788E-05	297	CTSS	15,8	1,493E-04	297	GAS1	5,6	0,000	297	GIMAP2	8,6	1,208E-04	297	CCDC96	5,1	4,363E-04
300	298	IFT74	19,3	5,691E-05	298	C1orf233	9,5	9,724E-05	298	COL4A5	15,7	1,952E-04	298	FAT3	5,6	0,001	298	MTUS1	8,6	2,240E-04	298	IQCD	5,1	1,803E-06
301	299	KIAA1107	19,2	1,985E-04	299	NAV1	9,5	7,459E-06	299	LYPD6	15,6	2,582E-05	299	CACNA1C	5,5	0,001	299	CDKN1C	8,5	1,465E-05	299	CAMK2N1	5,1	1,560E-04
302	300	GRTP1	19,1	3,410E-03	300	SRRM3	9,5	3,698E-03	300	TNFSF13B	15,3	3,008E-02	300	FXYD5	5,5	0,000	300	FIBIN	8,5	4,423E-06	300	PIH1D2	5,1	8,607E-05
303	301	SLC16A4	18,8	5,744E-04	301	FN1	9,5	1,781E-06	301	LRRC8C	15,3	1,372E-04	301	ITGA11	5,4	0,000	301	OAS1	8,5	2,179E-04	301	KLHL13	5,1	7,728E-05
304	302	CD83	18,7	1,859E-04	302	MCF2L	9,3	2,402E-02	302	IRF5	15,3	1,262E-06	302	HSPB6	5,4	0,000	302	STC2	8,5	2,052E-06	302	AGBL3	5,0	8,010E-05
305	303	PARD6A	18,7	2,513E-03	303	DEPTOR	9,2	9,079E-06	303	MAST4	15,2	1,231E-03	303	G0S2	5,4	0,000	303	ESYT3	8,5	6,926E-04	303	DPYD	5,0	1,394E-05
306	304	TMEM220	18,5	1,527E-02	304	FAM20A	9,1	4,819E-03	304	GLDC	15,2	4,160E-03	304	LOX	5,4	0,000	304	IL1A	8,5	3,796E-05	304	MORN2	5,0	1,094E-05
307	305	SH3BGR	18,4	1,514E-05	305	HSD3B7	9,1	1,564E-06	305	RBP1	15,0	3,202E-06	305	HSD3B7	5,3	0,000	305	OBSCN	8,5	6,698E-03	305	PTPRN2	5,0	4,398E-04
308	306	RMI2	18,4	1,968E-08	306	TPM1	9,0	1,478E-04	306	WDR17	15,0	1,572E-02	306	PTPLA	5,3	0,000	306	HTR7	8,4	8,138E-06	306	STK33	5,0	1,125E-03
309	307	ENPP4	18,3	2,011E-05	307	NBEAL2	9,0	5,509E-03	307	ANK1	14,9	5,894E-03	307	CHST15	5,2	0,003	307	TMEFF2	8,4	7,306E-05	307	WRB	5,0	1,377E-04
310	308	IFI44	18,2	2,312E-05	308	SLC22A15	9,0	8,491E-03	308	FZD3	14,7	3,461E-02	308	MOK	5,2	0,000	308	HLA-G	8,4	7,570E-04	308	LRRC23	4,9	3,360E-05
311	309	GCNT2	18,1	5,182E-03	309	MACROD1	8,9	1,057E-02	309	NIPA1	14,7	6,893E-05	309	TXNIP	5,2	0,000	309	MYLK	8,3	1,738E-02	309	AHCYL2	4,9	3,323E-03
312	310	RGS5	18,0	2,508E-04	310	LPHN2	8,9	7,667E-05	310	TGFA	14,6	4,646E-03	310	PDGFRB	5,2	0,000	310	CXCL3	8,3	7,489E-03	310	LZTS1	4,9	1,572E-03
313	311	CFI	18,0	6,494E-06	311	ADAM19	8,9	8,044E-09	311	ZNF385B	14,6	1,528E-03	311	TGFBI	5,1	0,001	311	ZNF467	8,3	2,499E-03	311	PROS1	4,9	4,616E-04
314	312	ZNF214	18,0	2,749E-03	312	COL27A1	8,8	1,750E-04	312	SP140	14,6	3,481E-02	312	COL5A1	5,1	0,001	312	ENG	8,2	6,869E-05	312	LEF1	4,8	5,110E-03
315	313	EFHC1	18,0	9,370E-05	313	GRIA3	8,7	6,281E-06	313	CADPS2	14,5	6,161E-05	313	ZNF204P	5,1	0,001	313	HERC6	8,2	3,534E-06	313	MAP2	4,8	1,202E-03
316	314	SLC27A6	18,0	1,965E-02	314	CRYAB	8,7	6,033E-06	314	REM1	14,4	1,240E-02	314	VEGFC	5,1	0,000	314	AEBP1	8,1	7,755E-03	314	KLF5	4,8	1,078E-07
317	315	MBOAT1	18,0	2,417E-04	315	SIK1	8,6	1,594E-05	315	PRTFDC1	14,3	1,010E-02	315	COL1A2	5,1	0,000	315	DOCK2	8,1	1,069E-03	315	RFX2	4,8	4,470E-04
318	316	SERPINI1	17,9	6,344E-04	316	ZNF503	8,6	1,728E-03	316	RAB36	14,3	1,188E-04	316	TDO2	5,1	0,000	316	SH2D5	8,0	8,535E-04	316	DHRS3	4,8	2,628E-07
319	317	TMEM231	17,8	2,881E-03	317	HOXC10	8,4	3,486E-04	317	TSPAN33	14,2	2,671E-07	317	CSGALNACT1	5,1	0,016	317	HS3ST3B1	8,0	9,090E-04	317	LRRC8B	4,7	6,138E-05
320	318	S1PR3	17,8	1,700E-06	318	PRDM11	8,4	2,880E-04	318	LRRC4	14,1	1,837E-04	318	TP53I11	5,1	0,000	318	KLF4	8,0	8,977E-06	318	GATS	4,7	4,425E-05
321	319	RGL3	17,7	4,500E-04	319	TRIP6	8,4	1,404E-04	319	GDPD1	14,1	6,201E-03	319	PAX6	5,0	0,004	319	FAM46A	8,0	7,467E-07	319	FEZ1	4,7	6,981E-05
322	320	RPRM	17,6	1,386E-04	320	WNT5B	8,3	3,658E-04	320	SVIP	14,0	3,053E-05	320	ABLIM3	5,0	0,000	320	SYTL5	8,0	5,553E-05	320	GNG7	4,7	2,519E-03
323	321	WDR31	17,5	1,533E-06	321	ROR2	8,2	3,187E-04	321	RARRES1	13,7	1,864E-04	321	VWA1	5,0	0,010	321	GPRC5C	7,9	2,852E-06	321	SLC1A1	4,7	1,907E-03
324	322	KRT80	17,5	2,114E-04	322	LYPD6B	8,2	1,143E-03	322	RPS6KA5	13,4	4,991E-06	322	RAC2	5,0	0,000	322	EPHB2	7,9	6,938E-04	322	ALDH3A2	4,7	7,661E-03
325	323	ZNF43	17,5	1,124E-04	323	SLC16A7	8,2	7,890E-05	323	PI15	13,3	3,100E-05	323	FAM182B	4,9	0,008	323	RAP1GAP2	7,9	1,475E-04	323	LYNX1	4,7	3,304E-03
326	324	FZD5	17,5	4,354E-06	324	C3	8,1	7,970E-06	324	MREG	13,1	1,147E-05	324	RGS7	4,9	0,002	324	PTPRC	7,8	7,050E-05	324	APBA2	4,6	1,588E-03
327	325	GFRA1	17,5	1,269E-07	325	VEGFC	8,1	2,670E-05	325	NEBL	12,9	4,306E-05	325	ZNF391	4,9	0,001	325	CNN1	7,8	1,639E-04	325	SDK1	4,6	4,305E-05
328	326	CDH3	17,4	1,282E-06	326	FAM89A	8,1	6,540E-04	326	LRRC34	12,7	1,139E-03	326	KCNG1	4,9	0,008	326	EEF1A2	7,7	7,255E-06	326	BFSP1	4,6	5,536E-03
329	327	BTG2	17,4	2,930E-04	327	STARD10	8,0	2,960E-02	327	KIAA1107	12,6	4,133E-05	327	CD68	4,8	0,000	327	CD274	7,7	6,142E-06	327	FAM69B	4,5	3,535E-03
330	328	HOTAIR	17,3	4,390E-05	328	SNPH	8,0	7,333E-04	328	CCDC146	12,5	4,570E-03	328	SLC16A2	4,8	0,007	328	SHISA5	7,6	3,247E-02	328	WDR91	4,5	4,831E-05
331	329	C12orf60	17,1	1,513E-03	329	SLC12A5	8,0	6,674E-03	329	MRAP2	12,4	2,273E-04	329	SPHK1	4,8	0,000	329	AXL	7,5	2,176E-06	329	SPA17	4,5	1,549E-03
332	330	CELSR2	17,0	3,593E-05	330	PARP3	7,9	2,269E-04	330	SPRY2	12,3	5,396E-06	330	CDC42EP2	4,8	0,000	330	FMNL1	7,4	5,546E-06	330	TEX9	4,5	2,266E-03
333	331	LRP1B	16,9	1,796E-03	331	RASSF5	7,9	4,888E-04	331	DACH1	12,1	8,130E-04	331	MYO1B	4,7	0,008	331	REM1	7,4	4,532E-04	331	CCDC121	4,5	1,974E-03
334	332	BBOX1	16,9	6,996E-05	332	C14orf132	7,9	4,858E-04	332	ST3GAL5	12,1	3,712E-05	332	PLP2	4,7	0,000	332	QPRT	7,4	2,665E-03	332	ZDHHC11	4,5	4,755E-02
335	333	MAP2	16,8	6,095E-04	333	SH2D5	7,9	5,202E-04	333	AFP	12,0	3,875E-03	333	FGF5	4,7	0,001	333	LY6E	7,4	1,097E-04	333	EML1	4,5	1,256E-03
336	334	C6orf52	16,8	2,261E-04	334	ABTB2	7,8	4,283E-07	334	C11orf70	11,8	9,615E-03	334	SCN1B	4,7	0,003	334	TRAF3IP2	7,3	5,670E-04	334	LGALS3	4,5	1,654E-04
337	335	HEY1	16,7	2,809E-06	335	MMP19	7,8	8,731E-06	335	SOCS2	11,8	2,540E-02	335	IRX5	4,7	0,018	335	MEX3B	7,3	5,772E-05	335	KLHL29	4,5	5,003E-04
338	336	LRRC48	16,7	1,067E-03	336	TNC	7,8	1,894E-04	336	ARSE	11,8	2,687E-04	336	ARHGEF6	4,7	0,000	336	ST6GALNAC5	7,1	2,852E-06	336	PKIA	4,5	2,684E-06
339	337	CARD16	16,6	1,527E-06	337	LPP	7,8	4,197E-05	337	CCL26	11,7	5,940E-05	337	GDPD5	4,6	0,003	337	SLC16A3	7,1	2,799E-02	337	C17orf97	4,5	1,942E-03
340	338	VWA5A	16,6	1,493E-05	338	PDLIM2	7,8	5,829E-03	338	TFPI2	11,6	2,464E-08	338	NEK7	4,6	0,000	338	PMAIP1	7,1	1,506E-04	338	CSPP1	4,5	1,127E-06
341	339	DTNA	16,3	9,227E-05	339	MYH9	7,8	3,399E-06	339	TGFB2	11,6	9,833E-06	339	JUNB	4,6	0,010	339	COL7A1	7,1	9,734E-04	339	PDLIM3	4,5	1,046E-03
342	340	SUSD2	16,0	4,537E-02	340	TPM4	7,8	3,004E-07	340	EFR3B	11,6	3,408E-03	340	CFH	4,5	0,000	340	CLIC1	7,0	5,694E-07	340	RAMP1	4,5	1,102E-02
343	341	CCDC151	16,0	4,229E-04	341	OLFM2	7,7	6,391E-05	341	SH3D21	11,6	2,975E-07	341	ROR1	4,5	0,000	341	NAV3	7,0	2,374E-04	341	SHROOM3	4,4	6,570E-06
344	342	TSPAN33	15,8	7,727E-07	342	AKR1C1	7,7	4,531E-02	342	MT1G	11,4	3,506E-04	342	PCK2	4,5	0,000	342	DUSP10	7,0	1,957E-05	342	LINC00475	4,4	1,189E-02
345	343	RNF157	15,8	2,406E-04	343	EFS	7,7	4,875E-06	343	SOD3	11,3	1,756E-03	343	PSG1	4,5	0,000	343	STMN3	7,0	2,245E-04	343	RAB36	4,4	2,164E-05
346	344	LHX2	15,7	1,848E-02	344	EVI2B	7,6	5,950E-03	344	FAXC	11,2	4,356E-04	344	H19	4,4	0,002	344	VDR	7,0	3,897E-05	344	ZNF285	4,4	9,665E-04
347	345	RTP4	15,7	2,422E-03	345	RNASE4	7,6	6,902E-05	345	C12orf60	11,2	3,027E-03	345	AMPD3	4,4	0,000	345	OAS3	6,9	1,428E-06	345	LRRC8D	4,4	3,812E-03
348	346	HERC6	15,6	1,052E-04	346	PTPRN	7,6	2,027E-03	346	MAP2K6	11,2	5,912E-05	346	ADAM12	4,4	0,000	346	IFI44L	6,9	9,844E-05	346	C2orf81	4,4	1,525E-02
349	347	PLEKHB1	15,6	4,685E-02	347	DIO2	7,6	1,270E-04	347	RASL11A	11,1	3,615E-04	347	CILP2	4,4	0,009	347	MX1	6,9	4,170E-04	347	EFCAB6	4,4	2,115E-03
350	348	EFNA1	15,6	8,416E-03	348	GRIK2	7,6	1,132E-02	348	DEPDC4	11,1	6,480E-03	348	HDHD1	4,4	0,000	348	JUNB	6,9	1,991E-03	348	EFHC1	4,4	2,124E-04
351	349	IL20RA	15,4	1,674E-03	349	CH25H	7,6	7,821E-05	349	ZNF140	11,0	1,940E-02	349	ST6GALNAC5	4,4	0,000	349	HLA-J	6,9	3,069E-03	349	CPNE2	4,4	2,000E-07
352	350	CCDC121	15,3	8,966E-04	350	TLN2	7,6	2,573E-05	350	ASRGL1	11,0	4,638E-03	350	WNT10B	4,3	0,017	350	DRAM1	6,8	2,212E-05	350	MAF	4,4	6,287E-04
353	351	CDH6	15,2	4,809E-05	351	STXBP5	7,6	6,826E-05	351	TLR4	10,9	8,680E-07	351	PTGDS	4,3	0,000	351	EGFL7	6,8	4,555E-04	351	VANGL2	4,3	7,105E-06
354	352	LETM2	15,2	9,259E-04	352	WNK4	7,4	1,028E-04	352	GLIS3	10,8	2,496E-06	352	EGLN3	4,3	0,009	352	RGS4	6,7	7,768E-06	352	WDR66	4,3	1,558E-03
355	353	GK	15,0	5,832E-05	353	NRXN3	7,4	9,971E-03	353	EXOC6	10,8	6,938E-05	353	PXK	4,3	0,000	353	TMEM63C	6,7	2,327E-03	353	C6orf52	4,3	4,962E-04
356	354	IGFBP2	15,0	1,079E-03	354	FOSL2	7,4	1,519E-07	354	MYCBPAP	10,8	3,011E-02	354	GPR1	4,3	0,004	354	IL27RA	6,7	7,102E-05	354	KIAA1377	4,3	5,242E-04
357	355	RHOJ	14,9	5,138E-06	355	ICAM5	7,4	8,955E-03	355	BFSP1	10,8	2,448E-04	355	KCNQ5	4,3	0,009	355	PAPPA	6,7	1,783E-07	355	ASIC1	4,3	3,662E-04
358	356	DHRS11	14,8	6,239E-05	356	F3	7,3	1,250E-04	356	PLEKHB1	10,8	6,344E-04	356	SLC29A3	4,3	0,000	356	MBP	6,7	4,769E-03	356	EFCAB2	4,3	1,896E-03
359	357	MYLK-AS1	14,8	5,072E-03	357	ADAMTS1	7,3	3,719E-07	357	TPPP	10,7	3,143E-04	357	SCML2	4,3	0,002	357	PDZD2	6,7	1,475E-02	357	SDCBP	4,3	2,388E-04
360	358	GLIS3	14,8	2,540E-06	358	SOD3	7,3	9,396E-06	358	GALNT1	10,6	1,116E-06	358	STARD10	4,2	0,046	358	SERINC2	6,6	1,190E-04	358	HTR1D	4,3	2,050E-03
361	359	MSX1	14,8	2,402E-03	359	EYA2	7,3	1,113E-02	359	TTC25	10,5	1,071E-02	359	ABTB2	4,2	0,000	359	TRANK1	6,6	1,791E-04	359	LRRC8C	4,3	3,987E-04
362	360	LRRCC1	14,7	4,024E-06	360	TNFAIP6	7,3	5,837E-03	360	AGBL3	10,5	1,655E-05	360	COL6A2	4,2	0,002	360	NREP	6,6	3,323E-04	360	ZNF546	4,3	1,290E-02
363	361	COL4A5	14,7	1,370E-04	361	ITGA4	7,2	7,728E-05	361	NRGN	10,5	2,536E-04	361	TLN2	4,2	0,000	361	AFAP1L2	6,6	1,317E-02	361	CETN2	4,3	5,408E-05
364	362	PRKAA2	14,7	8,220E-04	362	GPNMB	7,2	9,315E-05	362	FAM69B	10,4	2,302E-03	362	COL6A1	4,2	0,001	362	TYMP	6,6	1,755E-03	362	C1orf226	4,3	2,379E-08
365	363	ZNF140	14,6	1,584E-05	363	PICK1	7,2	1,880E-04	363	NAPEPLD	10,4	2,141E-02	363	HOMER3	4,2	0,005	363	INHBE	6,6	8,839E-04	363	RHPN2	4,2	8,963E-05
366	364	PEG10	14,6	6,399E-07	364	SLC29A3	7,2	2,464E-04	364	FMOD	10,4	1,190E-04	364	AHDC1	4,1	0,002	364	PICK1	6,6	1,175E-02	364	RARB	4,2	4,102E-04
367	365	ITGA7	14,5	5,143E-04	365	EPHB2	7,1	6,751E-05	365	TMEM25	10,4	4,750E-06	365	GRIK2	4,1	0,018	365	FSCN1	6,6	1,144E-04	365	ICA1L	4,2	1,024E-03
368	366	MTUS1	14,5	1,753E-02	366	HSPG2	7,1	1,159E-03	366	TMTC4	10,4	3,230E-07	366	FLNB	4,1	0,000	366	BDNF	6,6	1,369E-06	366	PDZD4	4,2	1,272E-02
369	367	TTC30B	14,4	2,163E-05	367	SUSD4	7,1	6,309E-04	367	KIAA1407	10,3	1,452E-04	367	HAPLN3	4,1	0,000	367	MEST	6,6	9,203E-07	367	PELI1	4,2	3,065E-05
370	368	SYTL5	14,4	1,607E-05	368	IL27RA	7,0	1,424E-05	368	CDCA7	10,3	3,504E-07	368	EPHA5	4,1	0,000	368	EHD3	6,6	3,291E-05	368	LCA5	4,2	3,560E-04
371	369	YPEL1	14,3	9,047E-05	369	SPHK1	7,0	2,551E-05	369	DTNA	10,3	3,422E-05	369	MIR210HG	4,1	0,004	369	HLA-B	6,5	1,302E-03	369	ABAT	4,2	2,596E-03
372	370	ZNF880	14,2	1,493E-06	370	CAMK4	7,0	8,208E-03	370	PPFIBP2	10,3	7,766E-06	370	SLC12A8	4,0	0,001	370	DGCR5	6,5	4,919E-04	370	FAT3	4,2	1,593E-03
373	371	RAB38	14,1	1,265E-02	371	FIBIN	7,0	5,899E-06	371	EMP1	10,2	1,529E-03	371	ENHO	4,0	0,003	371	GRAMD3	6,5	2,029E-07	371	AKAP6	4,2	4,822E-03
374	372	CHRNA5	14,0	2,547E-03	372	RGS16	7,0	8,725E-04	372	CD55	10,2	8,725E-07	372	GLI2	4,0	0,000	372	PTPRR	6,5	1,431E-04	372	DICER1-AS1	4,2	9,157E-03
375	373	EYA4	13,9	9,371E-05	373	IL15	6,9	4,429E-04	373	TNFSF4	10,2	1,132E-05	373	DOPEY2	4,0	0,004	373	ADAMTS6	6,4	3,105E-03	373	ZNF235	4,2	9,116E-04
376	374	ITPKB	13,9	2,723E-06	374	TSPO	6,9	3,147E-03	374	RIMKLB	10,1	4,073E-04	374	ADAP1	4,0	0,002	374	MARVELD1	6,4	1,721E-07	374	TMEM47	4,2	1,571E-04
377	375	SH3BGRL2	13,8	8,850E-05	375	TMEM54	6,9	4,732E-05	375	HSBP1L1	10,1	3,780E-04	375	FADS3	4,0	0,028	375	MSTN	6,4	1,413E-04	375	CADM1	4,2	1,066E-06
378	376	NEURL1B	13,7	2,938E-03	376	HS3ST3B1	6,8	1,031E-05	376	MBOAT1	10,1	4,183E-04	376	DACT1	4,0	0,001	376	EDN1	6,4	1,591E-05	376	LHFP	4,1	1,473E-07
379	377	MYH10	13,7	3,511E-04	377	TPST2	6,8	2,022E-03	377	WLS	10,1	9,227E-06	377	ZNF442	4,0	0,011	377	ACTC1	6,4	5,364E-04	377	FZD3	4,1	2,521E-02
380	378	DPY19L2	13,7	1,545E-03	378	TSPAN10	6,8	6,661E-04	378	SYNE2	10,1	5,927E-04	378	STXBP5	4,0	0,000	378	STEAP3	6,3	8,667E-05	378	TRIM36	4,1	4,776E-04
381	379	MANSC1	13,6	2,114E-02	379	DGCR5	6,7	1,754E-03	379	RHBDL2	10,0	7,964E-04	379	TOX	3,9	0,000	379	RTN4R	6,3	7,892E-04	379	UNC119B	4,1	3,278E-05
382	380	IQCH	13,5	1,239E-04	380	LRP1	6,7	6,168E-05	380	RHPN2	10,0	4,503E-05	380	LIME1	3,9	0,008	380	PYCARD	6,3	5,103E-04	380	WDR17	4,1	9,087E-03
383	381	NACC2	13,5	2,403E-04	381	CGREF1	6,7	1,237E-05	381	GBP5	10,0	2,238E-05	381	IL32	3,9	0,000	381	SLC29A1	6,3	1,817E-05	381	SCUBE2	4,1	1,288E-02
384	382	SELL	13,4	2,012E-03	382	TGFB1I1	6,7	7,212E-05	382	DUSP16	10,0	4,878E-05	382	SLC12A5	3,9	0,044	382	MRC2	6,3	3,117E-04	382	OSCP1	4,1	4,312E-05
385	383	FBXO16	13,4	7,716E-04	383	ROBO3	6,7	2,039E-03	383	INTU	9,9	6,802E-03	383	DIRAS1	3,9	0,000	383	TNNT1	6,3	4,157E-04	383	HSPA12A	4,0	1,778E-03
386	384	LURAP1L	13,4	7,080E-04	384	LMCD1	6,7	5,055E-06	384	GRTP1	9,9	4,366E-03	384	UCK2	3,9	0,000	384	TSLP	6,3	3,697E-04	384	ALDH2	4,0	1,470E-06
387	385	FLT1	13,3	1,570E-02	385	RAB3IL1	6,7	6,191E-04	385	MAP3K5	9,8	5,949E-07	385	EEPD1	3,9	0,001	385	OSR1	6,3	1,598E-03	385	FOXO1	4,0	2,223E-05
388	386	MFSD2A	13,3	4,600E-09	386	RGS11	6,6	7,433E-03	386	CAMK2N1	9,8	1,133E-06	386	CD70	3,9	0,002	386	GPR126	6,3	1,172E-03	386	DTNA	4,0	2,854E-04

	A	B	C	D	E	F	G	H	I	J	K	L	M	N	O	P	Q	R	S	T	U	V	W	X
389	387	CACNA2D1	13,1	7,709E-04	387	STEAP3	6,6	5,117E-05	387	GLB1L	9,8	7,286E-06	387	COL4A2	3,9	0,000	387	CCDC80	6,3	2,754E-05	387	C11orf63	4,0	1,715E-05
390	388	ZNF320	13,1	1,176E-03	388	ADAMTS10	6,6	1,099E-04	388	MANSC1	9,8	4,048E-03	388	CDHR3	3,9	0,001	388	MB21D2	6,2	2,251E-06	388	WNT2B	3,9	7,443E-04
391	389	TNFSF13B	13,1	7,656E-03	389	IL11	6,6	8,444E-05	389	LINC00475	9,7	2,688E-04	389	RGS4	3,8	0,049	389	LSP1	6,2	1,488E-02	389	GLB1L	3,9	5,396E-06
392	390	PCDHB15	12,9	2,458E-05	390	RB1	6,6	2,971E-06	390	SLC27A6	9,7	4,646E-02	390	IFITM2	3,8	0,000	390	RELB	6,2	5,032E-04	390	C21orf49	3,9	4,335E-02
393	391	GPR137C	12,9	6,086E-05	391	VGLL3	6,5	7,674E-03	391	TMEFF2	9,7	1,679E-03	391	IL15RA	3,8	0,005	391	RASEF	6,2	1,322E-06	391	LINC00094	3,9	8,695E-04
394	392	ZNF396	12,9	1,560E-05	392	BZRAP1	6,5	2,665E-03	392	E2F5	9,7	2,449E-05	392	PRDX4	3,8	0,000	392	GDF15	6,2	3,015E-03	392	HSPB6	3,9	1,311E-03
395	393	TGFA	12,8	2,482E-03	393	LGALS1	6,5	2,603E-04	393	CLCNKB	9,6	2,830E-03	393	ALDH1B1	3,8	0,000	393	RHBDF2	6,2	2,989E-05	393	AMOTL1	3,9	1,548E-06
396	394	KIAA0895	12,8	1,470E-03	394	EEF1A2	6,5	2,136E-04	394	MMP15	9,6	1,129E-04	394	ZNF879	3,8	0,004	394	NTN4	6,2	8,688E-05	394	KIF5A	3,9	2,956E-04
397	395	CAND2	12,7	1,520E-03	395	CALD1	6,5	3,304E-06	395	HERC6	9,6	1,113E-02	395	AHNAK	3,7	0,000	395	ZFYVE28	6,2	3,207E-02	395	CCDC41	3,9	3,131E-03
398	396	ZNF436	12,6	8,352E-06	396	HOMER3	6,5	3,511E-03	396	EFS	9,6	1,465E-03	396	AVEN	3,7	0,000	396	CD68	6,1	1,158E-02	396	KIAA1683	3,9	1,329E-05
399	397	NFIB	12,5	3,886E-05	397	PMEPA1	6,5	3,459E-08	397	MAP3K1	9,5	2,346E-06	397	IL4R	3,7	0,023	397	CHRM3	6,1	2,436E-04	397	MFAP3L	3,9	2,117E-02
400	398	PLA2G16	12,5	2,525E-06	398	TGFB1	6,4	7,169E-04	398	FAM43A	9,5	2,501E-03	398	PTPRG	3,7	0,000	398	TSHZ2	6,1	1,322E-04	398	NPHP1	3,9	1,308E-03
401	399	BTBD8	12,4	8,117E-03	399	C3orf18	6,4	5,322E-03	399	PODXL2	9,4	1,338E-04	399	TMEM158	3,7	0,000	399	SLFN11	6,1	7,222E-04	399	COL14A1	3,8	3,522E-04
402	400	CDS1	12,4	1,869E-04	400	NPAS2	6,4	1,778E-03	400	CMTM8	9,4	6,584E-05	400	FBLN5	3,7	0,002	400	CCDC85B	6,0	8,115E-03	400	TSPAN33	3,8	7,539E-06
403	401	RPS6KA5	12,3	5,177E-05	401	FTL	6,4	1,499E-07	401	SALL2	9,4	5,326E-04	401	RTN2	3,7	0,001	401	PML	6,0	3,799E-04	401	TMEM64	3,8	1,936E-05
404	402	RBP1	12,3	3,958E-07	402	RHBDF2	6,4	4,871E-05	402	A4GALT	9,4	1,094E-02	402	TACC2	3,7	0,004	402	FAM89A	6,0	2,151E-03	402	PTGFRN	3,8	1,419E-06
405	403	TM7SF2	12,3	3,662E-06	403	PARVA	6,4	4,149E-06	403	MARC1	9,4	5,031E-05	403	DAB2	3,7	0,000	403	C1orf94	6,0	2,402E-05	403	NES	3,8	1,082E-04
406	404	IFT57	12,2	1,452E-05	404	OBSCN	6,3	6,860E-04	404	MFAP5	9,3	1,127E-05	404	PLEKHO1	3,7	0,006	404	FKBP7	6,0	1,145E-03	404	XPR1	3,8	4,104E-05
407	405	PM20D2	12,2	1,584E-06	405	GATSL3	6,3	3,783E-02	405	CTGF	9,3	1,799E-06	405	ARMC9	3,7	0,000	405	HERC5	6,0	1,267E-05	405	COL4A6	3,8	2,240E-05
408	406	FBLN5	12,1	5,534E-04	406	TRIP10	6,3	7,355E-07	406	APOBEC3G	9,3	2,063E-04	406	RNF112	3,7	0,000	406	HS3ST3A1	6,0	5,492E-03	406	NFIB	3,8	9,544E-05
409	407	VAMP1	12,1	3,297E-03	407	SHROOM2	6,3	1,773E-02	407	TMEM140	9,2	6,804E-06	407	SLC2A3	3,7	0,006	407	ITPR3	5,9	4,106E-06	407	MED12L	3,8	7,811E-03
410	408	SPA17	12,1	6,888E-04	408	RRAS	6,3	2,131E-05	408	NLGN1	9,2	1,102E-03	408	CNN1	3,7	0,000	408	IFIT2	5,9	9,439E-05	408	GPRC5B	3,8	7,868E-05
411	409	DPYD	12,1	7,443E-06	409	SNAI1	6,2	2,644E-05	409	ARMCX4	9,2	1,138E-04	409	P4HA2	3,7	0,000	409	NCOA7	5,9	1,531E-04	409	ZNF396	3,7	8,781E-05
412	410	GBP5	12,0	1,459E-04	410	DAB2	6,2	4,345E-06	410	CLDN7	9,2	2,253E-03	410	CCDC107	3,7	0,001	410	TNFRSF1B	5,9	2,580E-03	410	CHN1	3,7	3,396E-04
413	411	PDGFRL	11,9	2,528E-03	411	C11orf45	6,2	2,791E-02	411	BTBD8	9,1	1,496E-03	411	CEBPB	3,7	0,006	411	ARHGAP25	5,9	7,998E-05	411	HSD17B14	3,7	9,939E-03
414	412	HES1	11,9	7,196E-05	412	SCN1B	6,2	1,999E-03	412	CCL2	9,1	1,112E-05	412	NMB	3,6	0,001	412	STARD5	5,9	3,799E-05	412	FAM46B	3,7	1,435E-03
415	413	LRRC8B	11,8	3,401E-05	413	FLRT2	6,2	1,006E-03	413	FAM107B	9,0	2,773E-05	413	GAS6	3,6	0,000	413	PXDN	5,8	1,047E-06	413	CHST15	3,7	1,530E-03
416	414	PSIP1	11,8	1,646E-08	414	VASP	6,2	3,780E-05	414	MUC12	9,0	6,830E-05	414	ARL4D	3,6	0,000	414	AK4	5,8	1,923E-05	414	CPEB2	3,7	1,860E-03
417	415	HOXC13	11,8	2,119E-04	415	SCN8A	6,1	1,984E-02	415	PTN	9,0	2,066E-07	415	CAMKK1	3,6	0,001	415	CLMP	5,8	1,050E-04	415	ALDH5A1	3,7	3,996E-05
418	416	TTC39B	11,8	5,054E-06	416	MANBA	6,1	2,905E-06	416	SPRY1	8,9	2,837E-02	416	ARHGAP28	3,6	0,000	416	PIM3	5,8	1,579E-03	416	AK1	3,7	1,638E-03
419	417	EZR	11,8	1,716E-05	417	IGFBP6	6,1	8,458E-05	417	STAMBPL1	8,9	4,731E-04	417	SLC9A7	3,6	0,002	417	ADAP1	5,8	1,241E-03	417	TASP1	3,7	4,334E-04
420	418	ZFP3	11,7	7,407E-07	418	GLT8D2	6,1	1,443E-03	418	CKB	8,9	3,676E-04	418	SPSB1	3,6	0,000	418	PDLIM7	5,8	5,511E-04	418	DOCK7	3,7	9,132E-04
421	419	PDGFB	11,6	7,504E-04	419	SULF1	6,1	6,043E-05	419	SMAD9	8,8	2,830E-04	419	TMEM160	3,6	0,021	419	SHROOM2	5,7	4,776E-03	419	SLC4A8	3,6	1,493E-04
422	420	SLC25A29	11,6	2,549E-04	420	TPD52L1	6,0	9,726E-04	420	STK33	8,8	2,399E-05	420	FBXL16	3,6	0,001	420	C14orf132	5,7	1,168E-04	420	C16orf93	3,6	5,251E-03
423	421	ALDH1A1	11,6	5,041E-06	421	TMEM200A	6,0	1,255E-04	421	PIH1D2	8,8	3,325E-04	421	NDST1	3,6	0,000	421	FRMD6	5,7	1,085E-06	421	RNF20	3,6	2,111E-04
424	422	PASK	11,5	8,265E-05	422	DUSP2	6,0	8,059E-04	422	SYBU	8,8	2,593E-04	422	SETD6	3,6	0,000	422	BMF	5,7	8,635E-06	422	RGS2	3,6	2,422E-04
425	423	PELI1	11,5	1,468E-05	423	OLFML2B	6,0	7,624E-04	423	FBXL2	8,8	7,936E-03	423	IGSF10	3,6	0,000	423	DOK4	5,7	2,103E-04	423	NDRG4	3,6	4,014E-02
426	424	CCP110	11,3	2,626E-05	424	NFE2	5,9	3,197E-03	424	TCN2	8,8	6,364E-04	424	PLXNA2	3,6	0,001	424	KCNN4	5,7	4,524E-02	424	TTC30B	3,6	9,218E-05
427	425	CCDC136	11,3	1,368E-04	425	GAS6	5,9	1,110E-04	425	PCDH9	8,8	1,292E-06	425	SH3KBP1	3,6	0,001	425	CPS1	5,6	7,768E-03	425	NGEF	3,6	1,493E-03
428	426	NIPA1	11,3	1,575E-06	426	ADM2	5,9	2,265E-05	426	ANK2	8,7	2,738E-05	426	GATA3	3,5	0,003	426	STC1	5,6	2,929E-05	426	CRIP1	3,6	3,170E-02
429	427	TYMS	11,2	5,249E-06	427	DLC1	5,8	6,142E-05	427	MYEF2	8,7	1,441E-03	427	S100A3	3,5	0,000	427	ADORA1	5,6	2,639E-03	427	SERINC5	3,6	1,333E-04
430	428	FKBP11	11,2	1,662E-04	428	ACTN1	5,8	2,457E-04	428	CDON	8,7	2,001E-04	428	ZBTB47	3,5	0,001	428	HPSE	5,6	1,314E-04	428	NTNG1	3,6	9,564E-05
431	429	TCTN2	11,2	2,304E-05	429	PTGS1	5,8	2,095E-04	429	KLF5	8,7	3,365E-05	429	TRIM46	3,5	0,000	429	KIAA1199	5,6	4,102E-05	429	C8orf37	3,6	3,019E-03
432	430	NIPSNAP3B	11,1	3,297E-04	430	IL12A	5,8	3,029E-02	430	CAND2	8,6	2,192E-04	430	ADAMTS10	3,5	0,000	430	ADAMTSL1	5,5	2,644E-03	430	PLXNC1	3,6	2,170E-05
433	431	CCDC153	11,1	1,307E-02	431	FER1L4	5,8	3,769E-03	431	GPR162	8,6	3,581E-03	431	ACTC1	3,5	0,016	431	C1orf233	5,5	1,800E-03	431	LRRCC1	3,6	3,369E-05
434	432	ZNF883	11,1	6,606E-05	432	PRSS23	5,8	9,429E-03	432	SESN3	8,6	3,517E-06	432	SLC1A5	3,5	0,005	432	DACT1	5,5	1,444E-05	432	ARMC2	3,6	2,296E-04
435	433	KIAA1377	11,1	2,567E-04	433	KCNIP3	5,7	7,822E-03	433	ACYP1	8,5	1,052E-07	433	CALHM2	3,5	0,001	433	HLA-F	5,5	3,813E-04	433	C11orf70	3,6	3,939E-04
436	434	SMAD9	11,1	5,949E-05	434	C10orf10	5,7	8,467E-05	434	KCTD1	8,5	1,482E-05	434	LDB2	3,5	0,000	434	ICAM1	5,5	5,744E-06	434	AMZ2P1	3,6	1,225E-03
437	435	COLEC12	11,1	4,749E-06	435	ACSL5	5,7	1,922E-03	435	LYPD6B	8,4	1,544E-06	435	UNC5B	3,4	0,000	435	DDX60	5,5	2,915E-05	435	SLC2A13	3,6	7,294E-04
438	436	ETV4	11,0	1,241E-05	436	FAT4	5,7	1,027E-04	436	RGS2	8,4	3,181E-06	436	BHLHE40	3,4	0,000	436	HLA-C	5,4	2,291E-05	436	ZNF542	3,5	4,981E-02
439	437	ZNF713	11,0	2,308E-05	437	MAFB	5,7	1,827E-04	437	ITGA2	8,4	2,627E-06	437	TRAF3IP2	3,4	0,002	437	DOK3	5,4	2,237E-04	437	PLCE1	3,5	2,602E-05
440	438	RBM26-AS1	10,9	2,516E-03	438	ETS1	5,7	2,446E-05	438	NR0B1	8,4	6,948E-03	438	ARRDC4	3,4	0,000	438	COL4A1	5,4	9,380E-05	438	PSENEN	3,5	2,424E-05
441	439	FAM211A	10,8	5,196E-04	439	NMB	5,6	3,701E-04	439	MMD	8,4	2,890E-06	439	MATN3	3,4	0,000	439	IGFBP6	5,4	1,198E-05	439	FRMD5	3,5	5,394E-05
442	440	FANCL	10,8	7,889E-06	440	HS3ST3A1	5,6	6,868E-03	440	TMEM217	8,4	9,003E-04	440	HHIPL2	3,4	0,001	440	CAMKK1	5,4	1,211E-03	440	KLHL25	3,5	2,303E-03
443	441	MMD	10,8	5,078E-06	441	IGFBP3	5,6	1,078E-05	441	FBN2	8,3	7,966E-03	441	S100A11	3,4	0,000	441	MIR210HG	5,4	2,600E-05	441	DPY19L2	3,5	5,387E-03
444	442	ADAMTS16	10,8	3,545E-04	442	BDNF	5,6	2,561E-05	442	CADM1	8,2	1,488E-02	442	RASSF9	3,4	0,004	442	HES4	5,4	1,060E-02	442	SEMA3A	3,5	6,213E-03
445	443	CRIP2	10,8	3,803E-02	443	VCAN	5,6	3,749E-06	443	IFI44L	8,2	8,361E-04	443	BBC3	3,4	0,005	443	PITX1	5,4	3,166E-02	443	TMEM67	3,5	5,262E-03
446	444	ZNF367	10,7	7,425E-08	444	LEPRE1	5,6	6,125E-07	444	TM7SF2	8,2	1,173E-04	444	HSPA12A	3,3	0,005	444	FAIM3	5,4	2,251E-02	444	TRIM9	3,5	1,141E-05
447	445	IL22RA1	10,7	1,545E-03	445	RABAC1	5,5	1,953E-03	445	PLA2G16	8,2	7,148E-05	445	CSTA	3,3	0,030	445	PRSS12	5,4	9,125E-03	445	ZMAT3	3,5	1,299E-02
448	446	ZNF665	10,7	5,609E-05	446	MAPK12	5,5	2,218E-04	446	GSTM4	8,2	8,152E-05	446	PPDPF	3,3	0,007	446	ASPHD1	5,4	1,858E-05	446	ZNF214	3,5	1,389E-02
449	447	METRN	10,7	9,132E-03	447	AFAP1L1	5,5	4,596E-04	447	ABAT	8,2	3,365E-04	447	NAV1	3,3	0,000	447	MT1F	5,4	3,314E-03	447	TMEM17	3,5	1,816E-05
450	448	MFI2-AS1	10,7	2,044E-03	448	GSTT1	5,5	2,221E-04	448	CHST2	8,1	2,821E-05	448	MGST3	3,3	0,000	448	G0S2	5,4	1,002E-04	448	THBS4	3,5	2,005E-03
451	449	HLA-DPA1	10,6	1,450E-03	449	EHBP1L1	5,5	6,498E-04	449	FEZ1	8,1	1,909E-05	449	RABAC1	3,3	0,003	449	CSMD2	5,3	1,704E-04	449	BBOX1	3,5	2,450E-03
452	450	CYP4V2	10,5	4,278E-05	450	CLIC1	5,5	4,877E-06	450	RRAGD	8,0	6,557E-03	450	MKNK2	3,3	0,000	450	BBC3	5,3	8,436E-03	450	COL11A1	3,5	6,374E-05
453	451	CNTRL	10,4	4,583E-05	451	CTSB	5,4	4,313E-03	451	SLC4A11	8,0	3,373E-04	451	SLMO1	3,3	0,040	451	GADD45A	5,3	4,214E-05	451	AIF1L	3,5	3,404E-02
454	452	PTPRN2	10,4	2,640E-04	452	NDST1	5,4	2,263E-05	452	IL1B	8,0	2,290E-04	452	FAM46A	3,3	0,000	452	DDIT4L	5,3	6,497E-04	452	ZNF302	3,5	1,741E-02
455	453	GINS3	10,3	3,703E-06	453	EHD2	5,4	5,050E-03	453	MGST2	7,9	5,020E-04	453	TSPAN4	3,3	0,000	453	CYP27B1	5,3	8,279E-03	453	CEP19	3,4	1,035E-04
456	454	C22orf23	10,3	1,669E-04	454	FGFR2	5,4	1,517E-04	454	S1PR3	7,9	6,740E-05	454	HSF2BP	3,3	0,010	454	HHIPL2	5,3	1,069E-04	454	PHLDB1	3,4	8,465E-03
457	455	MAPK10	10,3	7,720E-04	455	CYP27B1	5,4	1,982E-02	455	ARHGAP18	7,8	2,533E-06	455	LPCAT2	3,3	0,004	455	ANO7	5,3	3,692E-03	455	EPB41L1	3,4	3,536E-04
458	456	NDRG4	10,2	1,963E-02	456	ABCC4	5,3	1,928E-04	456	HES1	7,8	3,840E-06	456	VEGFA	3,3	0,001	456	F3	5,3	3,341E-06	456	RGL1	3,4	1,945E-03
459	457	IMPA2	10,2	3,277E-03	457	CCDC80	5,3	1,075E-03	457	IFI44	7,8	6,128E-05	457	ARHGEF17	3,2	0,000	457	BGN	5,2	3,017E-04	457	RFTN1	3,4	2,933E-04
460	458	RALGPS1	10,2	4,902E-04	458	SULF2	5,3	6,795E-07	458	TSGA10	7,8	6,203E-03	458	F3	3,2	0,000	458	PLSCR1	5,2	7,972E-06	458	C9orf72	3,4	1,107E-04
461	459	ATF3	10,2	3,002E-05	459	ATHL1	5,3	3,994E-05	459	DNAJC22	7,7	2,671E-04	459	CSMD2	3,2	0,000	459	C5orf46	5,2	1,511E-03	459	SYT11	3,4	2,700E-05
462	460	NRGN	10,2	3,986E-04	460	FSTL3	5,3	3,966E-04	460	SYTL4	7,7	2,364E-06	460	DDIT4	3,2	0,000	460	TNFAIP3	5,2	3,468E-04	460	ARNT2	3,4	8,889E-07
463	461	EMP1	10,2	2,614E-05	461	RIN1	5,3	1,496E-03	461	AK3	7,7	1,600E-06	461	NDUFS7	3,2	0,017	461	ADRA1B	5,2	1,778E-04	461	PTPRU	3,4	1,627E-02
464	462	COL9A2	10,2	8,419E-03	462	PPFIBP2	5,3	2,347E-03	462	MEGF9	7,7	1,049E-05	462	ANGPTL2	3,2	0,000	462	LPPR4	5,2	8,739E-05	462	STK36	3,4	2,884E-05
465	463	CCDC102B	10,2	5,545E-04	463	SGK1	5,3	1,346E-04	463	OSGIN1	7,7	1,122E-03	463	OBSCN	3,2	0,002	463	NRXN3	5,1	7,807E-04	463	FRAS1	3,4	1,342E-02
466	464	CEP19	10,2	3,507E-05	464	LTBP2	5,2	1,727E-06	464	MAP2	7,7	1,648E-03	464	PCOLCE	3,2	0,000	464	TNFRSF14	5,1	3,749E-03	464	SYNM	3,3	7,135E-05
467	465	BCL2	10,1	2,732E-06	465	FRMD4A	5,2	1,128E-05	465	CADM4	7,6	4,353E-05	465	NDRG1	3,2	0,035	465	STXBP5	5,1	2,075E-04	465	ERBB2	3,3	8,504E-05
468	466	METTL20	10,1	1,950E-02	466	BASP1	5,2	3,077E-05	466	RBM26-AS1	7,6	1,476E-02	466	HTRA1	3,1	0,000	466	RTP4	5,1	1,535E-05	466	CAPN5	3,3	9,572E-05
469	467	LCA5	10,1	1,841E-04	467	PLXNA4	5,2	6,435E-05	467	LRRCC1	7,6	3,232E-05	467	CD82	3,1	0,001	467	IRF7	5,1	3,042E-03	467	TYRO3	3,3	5,638E-06
470	468	ZSCAN23	10,1	4,244E-02	468	CILP2	5,2	8,473E-03	468	HIST2H2BE	7,5	6,634E-04	468	SNAI2	3,1	0,000	468	BTN3A3	5,1	5,261E-04	468	C9orf9	3,3	7,324E-05
471	469	GMPR	10,0	1,708E-04	469	RELB	5,2	1,356E-03	469	ID1	7,5	1,983E-03	469	SYTL3	3,1	0,021	469	UBE2L6	5,1	2,131E-06	469	EMP1	3,3	1,395E-04
472	470	PAQR6	9,9	7,390E-04	470	TUBA4A	5,2	2,317E-03	470	SEMA3C	7,5	2,846E-07	470	JOSD2	3,1	0,012	470	FXYD5	5,0	1,299E-05	470	TTLL7	3,3	1,465E-03
473	471	ACYP1	9,8	3,736E-08	471	MKX	5,2	4,491E-04	471	GXYLT1	7,5	3,597E-04	471	CMAHP	3,1	0,018	471	ATP8B1	5,0	1,808E-05	471	IFT81	3,3	1,004E-04
474	472	SYNE1	9,8	7,307E-04	472	FKBP10	5,2	1,552E-06	472	KIAA1671	7,4	1,353E-03	472	SLCO2A1	3,1	0,001	472	FOXL2	5,0	6,647E-04	472	ASS1	3,3	1,162E-06
475	473	HRCT1	9,8	7,990E-05	473	NSUN5	5,1	2,835E-04	473	COL21A1	7,4	9,471E-05	473	CCIN	3,1	0,000	473	SEMA3C	5,0	7,068E-06	473	NCAM1	3,3	8,167E-04
476	474	ZNF415	9,7	1,040E-05	474	PHLDB2	5,1	5,223E-06	474	TMTC2	7,4	1,330E-03	474	KCNIP3	3,1	0,019	474	IRAK2	5,0	1,690E-04	474	MOB1B	3,3	2,783E-03
477	475	KLHL29	9,7	2,754E-04	475	EPHB3	5,1	6,022E-05	475	TMEM194A	7,4	4,085E-07	475	NALCN	3,1	0,002	475	PROCR	5,0	7,887E-06	475	TCF7L1	3,3	4,430E-04
478	476	KIAA1324	9,6	3,512E-03	476	GNAO1	5,1	3,350E-03	476	PM20D2	7,4	9,402E-05	476	RCN3	3,1	0,008	476	GAS6	4,9	3,875E-04	476	NAP1L3	3,3	3,378E-02
479	477	RPGR	9,6	1,886E-05	477	IL11RA	5,1	1,207E-04	477	TBC1D8	7,3	2,053E-05	477	PRDM1	3,1	0,001	477	RASSF5	4,9	6,266E-04	477	SPATA13	3,3	1,104E-04
480	478	LYPD1	9,6	8,231E-06	478	VSTM2L	5,1	8,891E-03	478	PTPRN2	7,3	5,966E-05	478	SLC2A4RG	3,1	0,005	478	SRRM3	4,9	7,908E-03	478	MLLT3	3,2	5,726E-05
481	479	ARL6IP1	9,6	2,308E-05	479	CNTNAP1	5,1	4,547E-06	479	APCDD1	7,3	6,528E-03	479	MYBL1	3,1	0,001	479	IL4I1	4,9	4,732E-05	479	MEGF9	3,2	1,967E-04
482	480	MPP2	9,5	3,721E-05	480	FOXF1	5,1	1,352E-02	480	TRIM2	7,2	4,651E-06	480	TPM1	3,0	0,001	480	NEK7	4,9	2,212E-05	480	ZNF491	3,2	3,935E-02
483	481	ST6GALNAC3	9,5	1,899E-03	481	STARD5	5,0	8,078E-04	481	ITGA6	7,2	2,185E-03	481	PWWP2B	3,0	0,017	481	SIK1	4,9	1,062E-04	481	PFKFB2	3,2	9,236E-04
484	482	MNS1	9,4	6,257E-05	482	PTEN	5,0	1,999E-02	482	PLS1	7,2	1,264E-04	482	ARHGAP24	3,0	0,001	482	UBA7	4,9	1,379E-06	482	ADSSL1	3,2	5,110E-04
485	483	NUAK2	9,4	2,956E-05	483	DUSP7	5,0	1,047E-03	483	C5	7,2	1,152E-02	483	SLC38A1	3,0	0,000	483	MACROD1	4,9	3,760E-03	483	CDKL1	3,2	1,698E-03
486	484	ASMTL-AS1	9,3	2,674E-03	484	SELM	5,0	8,153E-03	484	SLC2A12	7,2	1,285E-06	484	FLRT2	3,0	0,002	484	PDE4A	4,8	5,964E-05	484	FAM212B	3,2	1,214E-05
487	485	HIST1H2AE	9,3	3,633E-04	485	NT5DC2	5,0	6,210E-06	485	DHX58	7,2	2,233E-04	485	RGMB	3,0	0,000	485	ETS2	4,8	3,386E-06	485	SELENBP1	3,2	8,674E-06
488	486	ATP6V1G2	9,3	1,384E-05	486	CSGALNACT1	5,0	1,533E-02	486	ZNF75A	7,1	5,375E-04	486	LRFN4	3,0	0,044	486	TAP1	4,8	1,109E-03	486	PRKAR2B	3,2	2,489E-04
489	487	ATP6V0E2	9,3	6,934E-04	487	ILK	5,0	6,016E-07	487	TOM1L2	7,1	1,577E-05	487	PLXNC1	3,0	0,003	487	SH2B3	4,8	5,191E-06	487	HIPK1	3,2	1,436E-02
490	488	DYX1C1	9,2	6,307E-03	488	SLC39A13	5,0	2,299E-04	488	COL4A3BP	7,1	2,426E-05	488	PDE4DIP	3,0	0,000	488	CLIC3	4,8	2,764E-03	488	BBS9	3,2	1,220E-03
491	489	RNF19A	9,1	1,570E-05	489	MGAT5B	5,0	5,892E-04	489	ANKRD6	7,1	1,694E-02	489	PLEKHN1	3,0	0,029	489	SCARB1	4,8	1,385E-06	489	GLIS3	3,2	3,865E-06
492	490	LINC00467	9,1	3,463E-07	490	BCAR3	4,9	6,816E-07	490	LGALSL	7,1	1,030E-04	490	RAB3IL1	3,0	0,002	490	PTPRB	4,8	2,982E-05	490	RABL5	3,2	5,106E-05
493	491	MAPK8IP1	9,1	3,411E-03	491	LEPR	4,9	3,424E-03	491	MFI2	7,1	2,754E-04	491	SMAD7	3,0	0,004	491	CRISPLD2	4,8	2,179E-02	491	ASNS	3,2	1,544E-03
494	492	UBE2T	9,1	2,174E-05	492	MARVELD1	4,9	2,236E-05	492	CSRP2	7,1	1,910E-03	492	KIRREL3	3,0	0,000	492	CSF1	4,8	2,469E-04	492	RASSF9	3,2	1,760E-04
495	493	C1orf204	9,1	2,997E-02	493	MFGE8	4,9	2,154E-07	493	KRT80	7,0	8,212E-05	493	STC2	3,0	0,000	493	NAV1	4,8	3,273E-05	493	MYO5B	3,2	7,658E-03
496	494	MGST2	9,1	1,592E-06	494	RAB3B	4,9	1,031E-06	494	PPP4R4	7,0	1,466E-02	494	BRE	3,0	0,000	494	TPCN1	4,8	6,189E-04	494	YPEL1	3,2	4,462E-04
497	495	ABHD3	9,0	1,300E-03	495	S100A16	4,9	2,571E-05	495	CHRNA5	7,0	2,854E-02	495	C19orf60	2,9	0,037	495	HLX	4,7	1,537E-03	495	MAPRE3	3,2	6,202E-06
498	496	IFI6	9,0	1,528E-02	496	TMEM198	4,9	8,222E-04	496	ZNF528	7,0	5,678E-04	496	TENC1	2,9	0,000	496	PFKFB4	4,7	7,944E-06	496	SLC7A11	3,1	1,927E-03
499	497	EFCAB2	9,0	1,053E-03	497	PDE3A	4,9	8,576E-04	497	YPEL1	7,0	1,453E-03	497	LMCD1	2,9	0,000	497	MMP11	4,7	1,474E-02	497	ZNF425	3,1	1,209E-04
500	498	WLS	9,0	1,141E-04	498	CYP1B1	4,9	1,843E-04	498	N4BP2L1	7,0	4,495E-03	498	PLEKHO2	2,9	0,001	498	CHPF	4,7	2,663E-03	498	PRADC1	3,1	2,795E-05
501	499	GAB2	8,9	8,528E-05	499	ATP8B1	4,9	2,590E-05	499	FCGRT	7,0	6,257E-03	499	HK2	2,9	0,000	499	NHS	4,7	9,429E-05	499	PIR	3,1	8,724E-06
502	500	MTSS1L	8,8	2,132E-03	500	TSLP	4,9	1,490E-04	500	PLEKHG4	6,9	2,679E-02	500	PARVA	2,9	0,000	500	IL32	4,7	4,277E-06	500	AMOT	3,1	2,935E-04
503	501	ADPRHL1	8,8	1,997E-05	501	ICAM1	4,9	2,390E-08	501	AKAP7	6,9	8,436E-05	501	SCN4B	2,9	0,019	501	TRPM4	4,7	3,406E-03	501	FBLN7	3,1	1,089E-06
504	502	HEATR5A	8,8	1,420E-05	502	JUNB	4,9	1,115E-02	502	ARHGAP26	6,9	7,345E-07	502	ZNF358	2,9	0,006	502	PARP12	4,7	3,402E-04	502	PHLPP1	3,1	2,669E-06
505	503	RASL11A	8,8	3,546E-03	503	HLX	4,8	6,634E-04	503	FGFR4	6,9	3,280E-02	503	GCNT1	2,9	0,005	503	SPHK1	4,7	2,829E-04	503	GUCY1B3	3,1	1,033E-05
506	504	OAS3	8,7	8,944E-05	504	THBS1	4,8	2,448E-06	504	PPARGC1A	6,9	6,880E-03	504	SLC25A6	2,9	0,003	504	APOE	4,7	3,418E-03	504	CLIP3	3,1	5,867E-05
507	505	CDON	8,7	2,853E-04	505	EHD3	4,8	5,546E-05	505	JAM3	6,8	1,053E-04	505	NR2F2	2,9	0,001	505	C2	4,7	1,008E-03	505	ACADS	3,1	7,859E-04
508	506	UNC119B	8,7	1,742E-05	506	ACVR1	4,8	2,591E-05	506	NDRG4	6,8	2,714E-04	506	KLF2	2,9	0,017	506	TNFRSF21	4,7	5,273E-06	506	BCL9	3,1	1,826E-06
509	507	SLC4A8	8,7	1,314E-04	507	FADS3	4,7	2,264E-02	507	ADORA2B	6,8	4,931E-05	507	STEAP3	2,9	0,000	507	CITED2	4,7	1,506E-04	507	KATNAL2	3,1	7,907E-04
510	508	HIST1H1C	8,7	6,335E-06	508	AHNAK	4,7	1,865E-05	508	DUSP4	6,8	2,924E-06	508	COL13A1	2,9	0,031	508	RGS7	4,7	6,275E-06	508	PGRMC1	3,1	1,202E-06
511	509	DHX58	8,7	4,370E-04	509	CRLF1	4,7	1,357E-02	509	STAP2	6,7	1,071E-05	509	ALDH1L2	2,9	0,000	509	IL22RA1	4,6	8,829E-05	509	LYPD1	3,1	3,187E-05
512	510	MAP2K6	8,7	5,594E-05	510	RBP4	4,7	3,231E-02	510	NCEH1	6,7	7,622E-04	510	CCDC22	2,9	0,000	510	SAMHD1	4,6	5,235E-07	510	TTC21A	3,1	1,498E-02
513	511	SSX2IP	8,6	2,451E-05	511	RGS4	4,7	3,813E-02	511	SSX2IP	6,6	1,844E-04	511	SLC36A1	2,9	0,004	511	SLC7A7	4,6	1,728E-03	511	FER	3,1	1,597E-02
514	512	PLCE1	8,6	1,004E-05	512	PNPLA3	4,7	1,625E-02	512	RAB42	6,6	2,475E-04	512	ZYX	2,9	0,000	512	IFI35	4,6	2,768E-05	512	KLHL5	3,1	7,704E-06
515	513	FGF12	8,5	1,115E-08	513	ETS2	4,6	6,778E-06	513	IGFBP2	6,6	1,196E-03	513	AIM1	2,9	0,003	513	CABLES1	4,6	1,388E-03	513	EAF2	3,1	7,820E-03
516	514	BEX2	8,5	4,881E-04	514	IL7R	4,6	1,197E-05	514	NR4A2	6,6	4,819E-04	514	ITGA5	2,9	0,000	514	MOB3C	4,5	6,967E-03	514	CC2D2A	3,1	1,058E-02
517	515	TMEM217	8,5	4,824E-02	515	ZNF358	4,6	1,349E-03	515	DBNDD1	6,6	4,176E-04	515	FAM212A	2,8	0,001	515	DHX58	4,5	1,372E-04	515	MIEN1	3,1	1,071E-04
518	516	SESN3	8,5	1,973E-04	516	SRD5A1	4,6	1,616E-02	516	EBF4	6,5	1,982E-05	516	FRMD4A	2,8	0,000	516	IFI30	4,5	2,008E-06	516	PSRC1	3,0	1,020E-03
519	517	ENHO	8,5	7,687E-06	517	IL15RA	4,6	3,394E-03	517	NOTCH4	6,5	1,817E-03	517	MUC1	2,8	0,000	517	FOSL2	4,5	2,715E-05	517	EGLN3	3,0	1,244E-03
520	518	ALG10B	8,5	2,940E-03	518	RRBP1	4,6	2,005E-03	518	SFRP4	6,5	6,764E-07	518	COL12A1	2,8	0,002	518	PIK3IP1	4,5	3,420E-03	518	HAS2	3,0	2,139E-05
521	519	HTR1D	8,5	1,430E-03	519	PTGFR	4,6	4,879E-07	519	GNG12	6,5	1,041E-07	519	TRAC	2,8	0,002	519	NR0B1	4,5	1,391E-02	519	CCDC136	3,0	6,044E-04
522	520	NEK2	8,5	5,601E-06	520	LHX9	4,6	2,421E-02	520	SYT11	6,5	1,428E-06	520	REEP1	2,8	0,002	520	TBKBP1	4,5	1,661E-04	520	MAGI2	3,0	1,258E-03
523	521	ZDHHC11	8,5	2,824E-02	521	TRIO	4,6	2,342E-04	521	DHFRL1	6,5	5,077E-05	521	LAMA5	2,8	0,006	521	TUBA4A	4,5	1,902E-05	521	DHX40	3,0	1,248E-03
524	522	CADM1	8,4	1,290E-06	522	SLC29A4	4,5	6,425E-03	522	TTC39B	6,5	6,310E-04	522	RBPMS	2,8	0,000	522	PARP10	4,5	2,359E-02	522	PGAP1	3,0	7,909E-03
525	523	ARSE	8,4	1,070E-04	523	P4HA1	4,5	3,931E-03	523	GALC	6,4	3,449E-05	523	UBE2D4	2,8	0,029	523	PSMB9	4,4	3,502E-05	523	ZNF385A	3,0	8,542E-04
526	524	NBR2	8,4	8,012E-04	524	TRIM46	4,5	2,642E-04	524	SAMD15	6,4	5,827E-03	524	HIF1A	2,8	0,000	524	TBC1D4	4,4	2,072E-05	524	ATP6V0E2	3,0	2,133E-03
527	525	GFM2	8,4	4,631E-06	525	PLEKHO1	4,5	3,885E-03	525	CDS1	6,4	5,974E-04	525	BAMBI	2,8	0,000	525	KDM6B	4,4	2,990E-03	525	MEGF8	3,0	7,343E-04
528	526	SGK494	8,4	2,624E-04	526	LPXN	4,5	8,071E-04	526	DDR1	6,4	1,542E-05	526	FLNC	2,8	0,000	526	RASGRP3	4,4	3,834E-04	526	MOK	3,0	2,550E-04
529	527	PMP22	8,3	4,656E-06	527	FAM26E	4,5	9,263E-05	527	SLC16A14	6,4	6,795E-03	527	METRN	2,8	0,040	527	IFITM10	4,4	9,050E-04	527	MYO6	3,0	1,798E-05
530	528	APOLD1	8,3	2,682E-04	528	SLC4A3	4,5	1,349E-04	528	ADRA1B	6,4	9,279E-06	528	METRNL	2,8	0,009	528	PTGS1	4,4	5,073E-04	528	TCTN2	3,0	9,193E-05
531	529	CXCL3	8,3	2,202E-02	529	SLC22A4	4,5	6,563E-06	529	ADD3	6,4	3,768E-04	529	DOCK11	2,8	0,000	529	DCN	4,4	4,649E-04	529	LMF1	3,0	1,565E-02
532	530	FGFR4	8,3	4,665E-02	530	ICAM3	4,5	3,686E-05	530	ITGB4	6,4	2,377E-04	530	GPR176	2,8	0,000	530	MCAM	4,4	6,273E-04	530	PLA2G16	3,0	8,870E-06
533	531	PIM2	8,3	2,016E-05	531	MIF	4,4	3,334E-02	531	FAM111B	6,3	1,007E-04	531	ACTN1	2,8	0,001	531	TMEM154	4,3	3,845E-04	531	TMEM91	3,0	3,118E-03
534	532	KRT7	8,3	4,368E-02	532	PPM1H	4,4	4,513E-03	532	FYCO1	6,3	4,720E-05	532	AMPH	2,7	0,000	532	SGK1	4,3	1,859E-05	532	TSPAN2	3,0	7,492E-05
535	533	IFI27	8,2	9,488E-03	533	SLC12A8	4,4	4,583E-04	533	MFHAS1	6,3	7,779E-06	533	KHDRBS3	2,7	0,000	533	TRAF1	4,3	9,492E-04	533	ADCK4	3,0	1,293E-02
536	534	TRIM22	8,2	3,944E-06	534	SH3PXD2A	4,4	1,541E-05	534	CYFIP2	6,3	4,102E-05	534	LANCL2	2,7	0,000	534	WFS1	4,3	1,706E-02	534	LTBP4	3,0	1,063E-02
537	535	AP1S3	8,2	2,253E-03	535	SLC25A32	4,4	4,006E-06	535	FAM117B	6,3	5,720E-04	535	PSG5	2,7	0,000	535	ECM1	4,3	9,024E-04	535	SORT1	3,0	1,583E-05
538	536	CEND1	8,2	4,002E-02	536	TRIB1	4,4	1,403E-06	536	KIAA0922	6,3	4,141E-05	536	RGS11	2,7	0,023	536	HLA-DMA	4,3	4,879E-04	536	GPR161	3,0	1,563E-05
539	537	HUNK	8,2	1,116E-02	537	SGCD	4,4	4,189E-07	537	ABLIM1	6,2	3,374E-05	537	THBS3	2,7	0,000	537	COL4A2	4,3	1,111E-04	537	DZIP3	3,0	2,957E-05
540	538	HCG27	8,2	3,026E-02	538	MAPK11	4,4	3,385E-07	538	PRKAR1B	6,2	1,651E-04	538	EXT1	2,7	0,000	538	TRIB1	4,3	1,410E-06	538	PPAP2A	3,0	7,297E-05
541	539	EAF2	8,1	3,168E-03	539	H1F0	4,4	8,813E-06	539	TMEM2	6,2	3,913E-04	539	TMEM47	2,7	0,000	539	EFS	4,3	3,367E-03	539	BAIAP2	3,0	6,033E-03
542	540	HELLS	8,1	2,027E-05	540	SNAP25	4,3	5,169E-04	540	TRANK1	6,1	1,228E-02	540	LFNG	2,7	0,000	540	ARRDC2	4,3	2,597E-04	540	RRAGB	2,9	1,633E-04
543	541	MORN2	8,1	4,730E-06	541	MRC2	4,3	2,126E-05	541	IL7R	6,1	4,625E-05	541	OGT	2,7	0,000	541	PDGFRB	4,3	4,107E-05	541	EFEMP1	2,9	2,299E-04
544	542	KATNAL2	8,1	2,317E-04	542	GPAM	4,3	2,332E-05	542	UCP2	6,1	8,164E-03	542	NT5M	2,7	0,006	542	SLC43A2	4,3	2,162E-02	542	TP53INP1	2,9	3,554E-04
545	543	PIR	8,1	3,050E-06	543	DPYSL4	4,3	2,020E-03	543	ECE1	6,1	5,696E-07	543	FHL2	2,7	0,000	543	ELK3	4,2	7,458E-05	543	ANKRD54	2,9	5,059E-04
546	544	IRF5	8,1	5,156E-04	544	RNF217	4,3	6,307E-03	544	H2AFJ	6,1	7,422E-04	544	CNN2	2,7	0,001	544	CGREF1	4,2	7,649E-04	544	DNAH11	2,9	4,571E-02
547	545	MAP6	8,1	1,600E-06	545	LAMB1	4,3	6,411E-04	545	KRBA2	6,1	2,496E-03	545	FAM129A	2,7	0,000	545	MPST	4,2	1,371E-03	545	ELMOD2	2,9	1,148E-03
548	546	TMEM17	8,1	1,184E-05	546	S1PR5	4,3	8,275E-03	546	FAM211A	6,0	1,808E-03	546	PPP1R3B	2,7	0,000	546	CACNB1	4,2	4,649E-04	546	LAMA1	2,9	1,417E-04
549	547	EXO1	8,1	1,658E-06	547	DCN	4,3	2,121E-05	547	SFXN2	6,0	6,212E-04	547	LRP1	2,7	0,000	547	AVPI1	4,2	7,805E-05	547	WLS	2,9	3,891E-04
550	548	RAB9B	8,0	6,005E-05	548	MXRA7	4,3	1,281E-03	548	FBXO16	6,0	8,894E-03	548	AJUBA	2,7	0,001	548	NOV	4,2	1,920E-05	548	BCHE	2,9	1,970E-04
551	549	STON1	8,0	4,088E-02	549	JPH2	4,3	8,656E-05	549	ZKSCAN2	6,0	2,996E-05	549	ALKBH7	2,7	0,019	549	SOCS1	4,2	1,545E-04	549	PPP1R9A	2,9	1,210E-03
552	550	DARC	8,0	3,002E-05	550	TENC1	4,3	3,660E-05	550	TMEM170B	6,0	1,865E-05	550	LYNX1	2,7	0,000	550	ADARB1	4,2	4,417E-03	550	STON2	2,9	1,635E-04
553	551	ZNF587	8,0	7,550E-05	551	IMPDH1	4,3	3,369E-06	551	VSIG10	6,0	1,503E-05	551	NHS	2,7	0,008	551	DDX60L	4,1	6,276E-05	551	CAPS2	2,9	5,783E-04
554	552	ZNF502	8,0	2,123E-04	552	IGSF1	4,2	6,480E-03	552	SLC37A1	6,0	3,890E-02	552	RUSC2	2,7	0,000	552	CAMK4	4,1	3,110E-04	552	FBN2	2,9	1,498E-04
555	553	HAUS6	8,0	1,707E-05	553	PTGER2	4,2	1,402E-02	553	ASB9	5,9	1,003E-04	553	PPIC	2,7	0,000	553	MILR1	4,1	4,601E-03	553	KIAA1161	2,9	1,674E-04
556	554	C9orf9	8,0	1,042E-05	554	ROR1	4,2	3,788E-07	554	CHIC2	5,9	1,140E-03	554	HNMT	2,7	0,006	554	DUSP7	4,1	5,132E-05	554	TOM1L2	2,9	2,681E-04
557	555	MARC1	8,0	1,553E-04	555	TMEM117	4,2	2,591E-04	555	PELI2	5,9	1,171E-04	555	FLNA	2,7	0,000	555	GBP4	4,1	1,207E-05	555	C2orf76	2,9	4,244E-03
558	556	MMP15	8,0	9,007E-04	556	LPPR4	4,2	1,234E-03	556	TNFRSF25	5,9	3,327E-04	556	INPP5J	2,7	0,009	556	NGFR	4,1	1,948E-03	556	SPTAN1	2,9	1,254E-05
559	557	HOXD10	7,9	5,372E-05	557	RAP1GAP2	4,2	8,562E-03	557	CCP110	5,9	3,057E-06	557	CCDC80	2,7	0,006	557	SLC22A23	4,1	1,808E-04	557	C11orf71	2,9	1,718E-03
560	558	CERS4	7,9	3,131E-04	558	TMEM59L	4,2	8,850E-05	558	C8orf46	5,9	5,503E-04	558	PLEC	2,7	0,027	558	MSLN	4,1	2,228E-03	558	MTSS1L	2,9	6,524E-03
561	559	NEFL	7,9	4,211E-05	559	FHL2	4,2	7,090E-07	559	HOTAIR	5,9	3,990E-03	559	BMP1	2,6	0,001	559	GPSM3	4,1	6,458E-03	559	GNAZ	2,9	7,483E-05
562	560	FAM135A	7,9	6,959E-05	560	FAM198B	4,1	1,393E-05	560	IL1RAP	5,9	1,464E-05	560	PAFAH1B3	2,6	0,002	560	KLC3	4,1	2,991E-03	560	CIRBP	2,9	1,519E-04
563	561	KIFC1	7,8	8,775E-04	561	OPLAH	4,1	1,509E-02	561	ASPN	5,9	2,276E-03	561	LONP1	2,6	0,001	561	DLX1	4,1	2,197E-04	561	TMEM19	2,9	1,977E-03
564	562	SVEP1	7,8	1,343E-04	562	ARRDC2	4,1	2,096E-02	562	UST	5,9	2,851E-06	562	VASP	2,6	0,000	562	C5orf56	4,1	5,258E-06	562	EIF4E3	2,9	3,273E-05
565	563	FAM122C	7,8	3,569E-04	563	CFH	4,1	1,284E-04	563	DLK2	5,8	6,099E-03	563	EHD2	2,6	0,013	563	MIF	4,0	1,094E-02	563	SLC1A3	2,9	2,737E-03
566	564	RNF182	7,8	1,339E-03	564	LPL	4,1	3,472E-05	564	ZNF43	5,8	2,453E-03	564	GTPBP6	2,6	0,007	564	CSRNP1	4,0	1,323E-04	564	ZFP3	2,9	9,444E-06
567	565	F11R	7,8	5,639E-06	565	TGFBR2	4,1	8,930E-07	565	CHD7	5,8	1,847E-04	565	ATP8B1	2,6	0,000	565	HDAC9	4,0	3,851E-05	565	PODXL	2,9	1,809E-04
568	566	HMGN5	7,8	1,256E-03	566	REEP6	4,1	8,734E-03	566	TNFAIP6	5,7	8,518E-05	566	ZFP36L1	2,6	0,000	566	KCNQ5	4,0	2,007E-03	566	KATNB1	2,9	8,992E-04
569	567	IFIT1	7,8	1,590E-02	567	C15orf39	4,1	8,228E-05	567	HLA-DPB1	5,7	1,018E-03	567	WTIP	2,6	0,004	567	STAT1	4,0	1,594E-06	567	MSI2	2,9	3,364E-04
570	568	ARHGEF4	7,7	5,676E-05	568	COL6A2	4,0	2,480E-03	568	PARP9	5,7	2,983E-06	568	TGFB1I1	2,6	0,000	568	ZNF503	4,0	2,254E-02	568	LRRC34	2,8	9,940E-04
571	569	TMEM194A	7,7	3,614E-06	569	SERPINB7	4,0	1,136E-02	569	DGCR11	5,7	7,324E-04	569	NEK3	2,6	0,011	569	MT2A	4,0	4,505E-04	569	ZBTB10	2,8	2,488E-03
572	570	MLF1	7,7	3,032E-04	570	SDC3	4,0	2,915E-06	570	KLHL5	5,7	2,793E-05	570	SLC6A9	2,6	0,000	570	PLA2R1	4,0	1,144E-06	570	VWA5A	2,8	9,352E-05
573	571	FAM161A	7,7	2,560E-04	571	FAM211B	4,0	7,068E-05	571	APOBEC3D	5,7	1,544E-04	571	MALT1	2,6	0,001	571	KRT15	4,0	6,139E-05	571	CENPF	2,8	4,816E-04
574	572	ZNF814	7,7	2,753E-04	572	KHDRBS3	4,0	8,437E-06	572	FGF13	5,7	4,745E-03	572	STK10	2,6	0,000	572	SPOCK1	4,0	1,145E-02	572	TMEM170B	2,8	4,958E-04
575	573	IFT81	7,6	3,913E-05	573	RTN4R	4,0	4,004E-02	573	EML2	5,6	1,506E-05	573	DPYSL3	2,6	0,000	573	A4GALT	3,9	4,451E-03	573	SSX2IP	2,8	5,131E-04
576	574	C1QL1	7,6	5,944E-03	574	ALDH1B1	4,0	5,568E-07	574	AP1S2	5,6	1,202E-06	574	TFEB	2,6	0,002	574	ADAM12	3,9	3,205E-04	574	MPND	2,8	1,254E-02
577	575	PLXNC1	7,6	4,524E-06	575	TSSC4	4,0	2,777E-04	575	ZNF214	5,6	2,962E-02	575	MFAP2	2,6	0,001	575	HLA-E	3,9	7,265E-06	575	ATP6V1B2	2,8	2,562E-06
578	576	IQCK	7,6	4,845E-03	576	SLC26A6	4,0	6,670E-04	576	PPM1H	5,6	1,154E-04	576	DISP2	2,6	0,023	576	MGAT5B	3,9	1,112E-03	576	KCTD17	2,8	9,129E-04
579	577	FAM107B	7,6	3,590E-04	577	NEK7	4,0	9,898E-07	577	GNG4	5,6	3,150E-02	577	ENG	2,6	0,000	577	TRIM8	3,9	9,005E-05	577	DNER	2,8	4,869E-03
580	578	ATP6AP1L	7,6	1,684E-04	578	ALKBH7	4,0	8,477E-03	578	ZNF542	5,6	3,293E-03	578	COL6A3	2,5	0,000	578	MFAP2	3,9	9,007E-03	578	BTG2	2,8	1,383E-03
581	579	CDCA3	7,6	3,313E-05	579	JAG1	4,0	2,578E-07	579	E2F8	5,5	8,406E-03	579	RNASE4	2,5	0,000	579	CMTM7	3,9	2,722E-03	579	SNAPIN	2,8	2,457E-04
582	580	ASB9	7,6	3,770E-05	580	CDC42EP2	4,0	2,535E-05	580	DAPK2	5,5	8,025E-06	580	ACTG2	2,5	0,001	580	CD55	3,9	1,203E-03	580	MYLK-AS1	2,8	1,910E-02
583	581	TLR3	7,6	6,057E-07	581	TSPAN4	4,0	5,528E-05	581	PGF	5,5	6,498E-05	581	RUNX2	2,5	0,000	581	SLC26A6	3,8	1,837E-06	581	IFT140	2,8	3,636E-05
584	582	SHROOM3	7,6	4,408E-06	582	LTBP1	4,0	9,325E-03	582	SPIRE2	5,5	2,807E-05	582	CLTCL1	2,5	0,001	582	CELSR3	3,8	3,367E-02	582	NIPA1	2,8	1,400E-05
585	583	ZNF385A	7,5	1,102E-04	583	ST6GALNAC5	3,9	2,343E-04	583	SERINC5	5,5	2,697E-04	583	SH3BP2	2,5	0,002	583	RAB42	3,8	1,045E-03	583	UBE2H	2,8	3,774E-06
586	584	MAGI2	7,5	4,266E-04	584	YIF1A	3,9	8,991E-05	584	DNMBP	5,5	9,024E-05	584	MID1IP1	2,5	0,003	584	FAT4	3,8	4,614E-04	584	AVIL	2,8	2,972E-02
587	585	MAST4	7,5	1,952E-05	585	APOE	3,9	2,806E-02	585	PHLPP1	5,5	2,451E-06	585	KLHL4	2,5	0,000	585	SP100	3,8	1,296E-04	585	CEBPG	2,8	4,232E-04
588	586	TMCC2	7,4	5,177E-04	586	LIMS1	3,9	6,195E-06	586	C22orf23	5,5	5,911E-03	586	MAN1C1	2,5	0,000	586	MET	3,8	1,397E-03	586	TM7SF2	2,8	1,276E-05
589	587	ATF6B	7,4	7,567E-05	587	PFKFB4	3,9	1,176E-03	587	SORBS2	5,5	4,844E-04	587	LCP1	2,5	0,000	587	MOCOS	3,8	3,557E-04	587	APOA1BP	2,8	4,768E-06
590	588	FAM43A	7,4	3,409E-03	588	GNB1L	3,9	1,518E-02	588	SVEP1	5,5	1,027E-02	588	PDGFC	2,5	0,013	588	EGR2	3,8	5,764E-04	588	IFT46	2,8	3,017E-04

	A	B	C	D	E	F	G	H	I	J	K	L	M	N	O	P	Q	R	S	T	U	V	W	X
591	589	PER2	7,4	1,200E-05	589	SLC45A3	3,9	9,110E-04	589	PROS1	5,5	5,317E-05	589	RELB	2,5	0,004	589	SAMD12	3,8	3,975E-02	589	MAP3K14	2,8	2,513E-03
592	590	PERP	7,4	6,218E-06	590	IER3	3,9	1,538E-02	590	CREB5	5,4	2,912E-04	590	TUBA4A	2,5	0,010	590	THSD4	3,8	5,548E-05	590	SLC41A1	2,8	3,195E-04
593	591	FAM182B	7,4	3,618E-03	591	ITPR3	3,9	1,704E-06	591	AASS	5,4	3,332E-06	591	C21orf2	2,5	0,048	591	TRIM14	3,8	9,824E-05	591	FAM102B	2,8	1,501E-04
594	592	CETN2	7,3	3,286E-05	592	FAM46A	3,9	9,319E-07	592	PIR	5,4	2,764E-05	592	GARS	2,5	0,000	592	PGBD3	3,8	1,336E-04	592	EVI5	2,8	3,710E-04
595	593	RALGAPA2	7,3	9,397E-05	593	PPP1R13L	3,9	3,323E-03	593	CTXN1	5,4	3,624E-03	593	GALNT5	2,5	0,000	593	ZNF697	3,8	9,517E-05	593	KIAA1324	2,8	2,146E-02
596	594	TFAP2C	7,3	7,302E-06	594	SNORD104	3,9	4,193E-02	594	SLC45A4	5,4	8,923E-04	594	PTPRU	2,5	0,009	594	ARHGAP18	3,8	3,854E-07	594	YJEFN3	2,8	8,184E-05
597	595	LGALSL	7,3	4,151E-04	595	LIMA1	3,9	1,666E-04	595	C3orf58	5,4	4,871E-04	595	BCL9L	2,5	0,001	595	SYT12	3,8	2,252E-04	595	FAM131A	2,8	1,601E-03
598	596	APOBEC3D	7,3	1,418E-02	596	PCDHB13	3,8	6,965E-04	596	GBP4	5,4	7,562E-04	596	ZNF44	2,5	0,001	596	RAB32	3,8	2,393E-06	596	ANKRD46	2,8	6,372E-03
599	597	STRBP	7,2	1,776E-05	597	PCDHB14	3,8	7,281E-05	597	DNALI1	5,4	5,688E-03	597	HRAS	2,5	0,000	597	TEAD3	3,8	9,854E-05	597	GK	2,8	8,940E-04
600	598	PTN	7,2	1,676E-07	598	KLHL17	3,8	4,236E-02	598	TMEM206	5,3	1,026E-05	598	VASH1	2,5	0,002	598	ADCY7	3,8	2,997E-05	598	NIPSNAP3B	2,8	1,440E-03
601	599	CKAP2	7,2	6,845E-05	599	DDIT4L	3,8	1,820E-04	599	CRYAB	5,3	1,918E-04	599	FBXO32	2,5	0,001	599	LSR	3,8	1,375E-02	599	PARD6A	2,8	1,139E-02
602	600	BEX4	7,2	4,726E-06	600	OSR1	3,8	1,995E-02	600	SPTBN2	5,3	1,124E-02	600	SESN2	2,5	0,000	600	PCDH7	3,7	4,726E-05	600	COL10A1	2,8	3,162E-02
603	601	PODXL2	7,2	6,893E-04	601	AVEN	3,8	3,947E-04	601	DZIP3	5,3	6,672E-04	601	C1S	2,5	0,000	601	NRN1	3,7	1,764E-05	601	NEK2	2,8	2,031E-05
604	602	NTNG1	7,2	1,845E-05	602	FCHSD2	3,8	1,047E-05	602	FOS	5,3	3,165E-02	602	RRBP1	2,5	0,005	602	MRAP2	3,7	3,869E-03	602	PPP1R12B	2,7	1,572E-04
605	603	KIF14	7,1	7,364E-05	603	PLA2G4C	3,8	1,164E-03	603	MYLK-AS1	5,3	1,586E-02	603	GBP1	2,5	0,000	603	SLC7A8	3,7	3,544E-03	603	SOX13	2,7	2,205E-03
606	604	AMOTL1	7,1	4,038E-07	604	PLA2R1	3,8	1,517E-03	604	SLC22A23	5,3	3,527E-05	604	C1orf198	2,5	0,000	604	LEPR	3,7	3,351E-03	604	CYB5D2	2,7	1,168E-02
607	605	E2F8	7,1	1,358E-05	605	ZYX	3,8	5,117E-05	605	F2RL2	5,2	1,632E-04	605	PLEKHG3	2,5	0,028	605	MMP2	3,7	3,449E-07	605	ZCCHC18	2,7	1,776E-03
608	606	ERO1LB	7,1	9,911E-04	606	SRD5A3	3,8	5,043E-04	606	LHX2	5,2	2,394E-04	606	TNRC18	2,5	0,007	606	COL27A1	3,7	5,540E-04	606	CTSH	2,7	1,128E-04
609	607	FAM120C	7,1	6,397E-06	607	OSBPL5	3,8	2,075E-02	607	CNKSR3	5,2	7,163E-05	607	TSNARE1	2,5	0,001	607	TNFAIP8	3,7	1,247E-03	607	SYNPO	2,7	2,612E-03
610	608	VTN	7,1	1,763E-03	608	CHI3L1	3,8	1,235E-06	608	MSI2	5,2	7,843E-04	608	CSRP1	2,5	0,006	608	APOBEC3G	3,7	8,554E-04	608	CNN3	2,7	6,858E-05
611	609	TMPO	7,1	2,620E-06	609	ADRA1B	3,8	7,437E-05	609	HMGA1	5,2	1,565E-03	609	GEMIN8	2,5	0,011	609	PARVB	3,7	1,612E-04	609	ABCA3	2,7	3,261E-04
612	610	B9D1	7,1	3,578E-06	610	PQLC3	3,8	1,661E-03	610	GPR155	5,2	7,292E-04	610	DSE	2,4	0,000	610	TPM2	3,7	1,052E-04	610	SPEF2	2,7	1,719E-03
613	611	CD47	7,1	1,332E-04	611	ATP10D	3,8	6,477E-07	611	PLCE1	5,2	7,293E-06	611	SDC2	2,4	0,022	611	ERCC6	3,7	1,949E-04	611	CLUAP1	2,7	6,032E-03
614	612	CDC7	7,1	1,948E-04	612	GSN	3,8	6,648E-05	612	ITGA4	5,2	1,029E-03	612	CHAC1	2,4	0,001	612	BICC1	3,7	5,496E-05	612	FAM109A	2,7	5,179E-03
615	613	TCP11L2	7,1	1,350E-06	613	ANKH	3,7	9,483E-05	613	ENO1-AS1	5,1	1,531E-03	613	PLXDC2	2,4	0,000	613	PPL	3,7	2,885E-04	613	CKAP2	2,7	2,627E-04
616	614	RBM24	7,1	9,610E-05	614	PCBP4	3,7	4,564E-05	614	ANKRD10	5,1	5,312E-06	614	TIMP1	2,4	0,000	614	TNFSF13B	3,7	1,785E-02	614	LOH12CR2	2,7	1,606E-02
617	615	MEGF9	7,1	8,620E-05	615	CMAHP	3,7	1,269E-02	615	GOLIM4	5,1	1,291E-06	615	ZNF285	2,4	0,005	615	TRIL	3,7	3,192E-04	615	ALDH9A1	2,7	5,464E-06
618	616	CTNNA2	7,0	1,881E-05	616	FGF5	3,7	8,489E-04	616	SYNJ2	5,1	1,086E-05	616	PDLIM2	2,4	0,020	616	LHX9	3,7	3,041E-03	616	NRBP2	2,7	5,136E-03
619	617	NEK11	7,0	7,454E-04	617	PLCB4	3,7	1,263E-04	617	SLC25A4	5,1	3,839E-05	617	ZMIZ2	2,4	0,001	617	RCN3	3,7	2,515E-03	617	NR2F1	2,7	1,832E-03
620	618	ZNF19	7,0	2,286E-03	618	PML	3,7	2,990E-03	618	TNFSF10	5,1	3,240E-04	618	YIF1A	2,4	0,000	618	EMR1	3,7	3,251E-03	618	CYP27A1	2,7	4,176E-06
621	619	ATAD5	7,0	4,796E-04	619	FZD7	3,7	6,667E-05	619	TRIM22	5,1	9,874E-05	619	HMCN1	2,4	0,000	619	SAMD9	3,7	9,311E-05	619	RNF112	2,7	6,088E-04
622	620	LRRC8C	7,0	2,385E-04	620	PFKP	3,7	2,812E-05	620	SLC6A6	5,1	4,892E-02	620	GPR156	2,4	0,025	620	TAGLN	3,7	5,208E-04	620	HES1	2,7	3,638E-04
623	621	RAB3IP	6,9	3,738E-05	621	C19orf60	3,7	3,267E-02	621	P2RX7	5,1	2,034E-04	621	SSBP4	2,4	0,009	621	PLCD4	3,6	8,682E-03	621	GPR137B	2,7	3,592E-04
624	622	HOXB3	6,9	1,684E-03	622	LGALS9	3,7	1,103E-04	622	MPP2	5,1	3,535E-04	622	TCIRG1	2,4	0,005	622	KCNS3	3,6	1,542E-06	622	C22orf29	2,7	6,701E-06
625	623	DHX40	6,9	4,944E-04	623	IL4R	3,7	2,459E-02	623	MCF2L	5,0	3,692E-02	623	PCDH7	2,4	0,002	623	DOCK6	3,6	1,993E-03	623	TCTN1	2,7	6,732E-04
626	624	ALG10	6,9	7,929E-05	624	CDR2L	3,7	3,686E-04	624	PLCB1	5,0	3,557E-06	624	TRIP10	2,4	0,000	624	BCAR3	3,6	1,636E-05	624	NUAK2	2,7	1,370E-04
627	625	KIAA1683	6,9	1,354E-05	625	EMR1	3,7	8,310E-04	625	HCG27	5,0	2,303E-02	625	MYH9	2,4	0,000	625	LURAP1L	3,6	7,393E-04	625	CHST2	2,7	1,900E-03
628	626	POLQ	6,9	1,671E-05	626	PLAC1	3,7	1,242E-02	626	ACP6	5,0	4,412E-03	626	COL7A1	2,4	0,001	626	PAPSS2	3,6	1,583E-05	626	COX20	2,7	6,210E-03
629	627	NUF2	6,9	7,409E-06	627	GALNT5	3,7	7,427E-06	627	ADAMTS9	5,0	9,394E-05	627	EDIL3	2,4	0,000	627	PTK7	3,6	1,119E-04	627	SLC9B2	2,7	1,150E-03
630	628	PSPH	6,9	2,401E-07	628	SIM2	3,7	2,133E-02	628	ACBD4	5,0	7,554E-04	628	CAPN2	2,4	0,000	628	NFKBIE	3,6	6,359E-04	628	ZNF221	2,7	4,735E-02
631	629	APOBEC3B	6,9	6,267E-07	629	C15orf52	3,7	3,246E-03	629	C3orf33	5,0	1,530E-04	629	CPA4	2,4	0,000	629	MICB	3,6	1,424E-05	629	CHKA	2,7	1,004E-05
632	630	AK3	6,9	3,107E-05	630	CNN1	3,7	9,132E-05	630	TMEM200B	5,0	1,766E-06	630	C7orf50	2,4	0,005	630	STX1A	3,6	7,956E-03	630	ZIC2	2,7	1,452E-02
633	631	CDCA7	6,9	3,011E-04	631	PLAC8	3,7	7,702E-04	631	FOXO1	5,0	2,801E-07	631	TPST2	2,4	0,009	631	EVI2A	3,6	1,974E-02	631	SEMA6C	2,7	5,854E-04
634	632	WWOX	6,9	3,868E-04	632	BRE	3,6	6,859E-08	632	UACA	5,0	8,957E-04	632	TBC1D4	2,4	0,000	632	HLA-A	3,6	1,016E-04	632	RAB9B	2,7	2,687E-04
635	633	COQ10A	6,9	1,290E-04	633	FHOD1	3,6	1,281E-04	633	SMAD3	5,0	1,814E-06	633	PDGFA	2,4	0,000	633	ZCCHC2	3,6	3,713E-04	633	MYCBPAP	2,7	4,889E-02
636	634	TMEM67	6,9	2,552E-03	634	STK10	3,6	3,669E-05	634	FZD4	5,0	1,326E-04	634	WWC1	2,4	0,001	634	FGFR2	3,6	9,258E-05	634	FAM161A	2,7	5,496E-04
637	635	LRRC23	6,9	4,292E-05	635	MAGI1	3,6	3,742E-03	635	DAG1	5,0	2,658E-03	635	FN1	2,4	0,000	635	IRF1	3,6	7,614E-03	635	STRBP	2,7	1,491E-03
638	636	CCNE2	6,9	1,368E-05	636	CNN2	3,6	7,500E-04	636	PLTP	5,0	1,347E-05	636	TCP11L1	2,4	0,000	636	SLC2A6	3,6	5,472E-03	636	ZNF527	2,6	2,825E-03
639	637	RFC5	6,9	9,867E-05	637	BRSK1	3,6	2,327E-02	637	SPRY4	5,0	1,095E-04	637	COL27A1	2,4	0,001	637	FBLIM1	3,6	4,953E-03	637	FAM63A	2,6	3,066E-03
640	638	LYRM5	6,8	4,668E-02	638	BCL3	3,6	1,970E-03	638	ATF3	5,0	2,190E-04	638	SLC52A2	2,4	0,014	638	NRG1	3,6	3,493E-06	638	HSBP1	2,6	3,733E-05
641	639	HIST2H2BE	6,8	2,028E-06	639	TBC1D4	3,6	1,135E-04	639	TMEM198B	5,0	2,707E-05	639	TRAF5	2,4	0,000	639	SIX4	3,6	4,408E-02	639	NEURL1B	2,6	1,318E-02
642	640	EFCAB6	6,8	1,973E-04	640	IKBKE	3,6	1,204E-03	640	SAT1	5,0	1,539E-06	640	SLC39A13	2,3	0,001	640	NID2	3,6	4,389E-04	640	SPAG17	2,6	1,977E-04
643	641	CHAF1B	6,8	1,671E-06	641	MPST	3,6	3,933E-02	641	KIAA1467	5,0	4,185E-06	641	CGNL1	2,3	0,004	641	SQRDL	3,6	5,291E-05	641	TMX4	2,6	9,552E-05
644	642	CRIP1	6,8	1,860E-02	642	ZC3H12A	3,6	1,584E-04	642	DENND2A	5,0	4,889E-05	642	ERF	2,3	0,005	642	HIST1H2BK	3,6	2,601E-05	642	KHK	2,6	2,411E-02
645	643	FBXL16	6,8	1,212E-03	643	EMILIN2	3,6	1,561E-03	643	PCDH10	5,0	2,662E-06	643	FARP1	2,3	0,000	643	COL5A2	3,6	1,668E-05	643	DYRK1B	2,6	1,583E-02
646	644	NAPRT1	6,7	4,484E-02	644	GPR176	3,6	2,243E-05	644	GPRC5A	4,9	2,544E-04	644	SLC4A3	2,3	0,001	644	CMAHP	3,5	4,134E-03	644	PARK2	2,6	4,824E-02
647	645	SYNM	6,7	4,049E-05	645	CTSD	3,5	1,652E-04	645	FAM117A	4,9	1,095E-02	645	NEXN	2,3	0,006	645	PSORS1C1	3,5	3,817E-03	645	DPF1	2,6	1,650E-03
648	646	RNF20	6,7	1,115E-04	646	RNF122	3,5	1,362E-04	646	METTL20	4,9	1,876E-02	646	BCAS4	2,3	0,013	646	IFI44	3,5	3,836E-05	646	GDPD1	2,6	5,518E-03
649	647	TCTN1	6,7	2,040E-04	647	ADAMTSL1	3,5	1,072E-02	647	CASP10	4,9	2,236E-02	647	EIF4EBP1	2,3	0,000	647	CDC42EP3	3,5	5,371E-05	647	GLIPR2	2,6	1,841E-02
650	648	C21orf91	6,7	2,153E-03	648	ZDHHC8	3,5	1,932E-02	648	RGS5	4,9	4,272E-02	648	ERCC2	2,3	0,000	648	SLIT2	3,5	2,712E-05	648	ATP9A	2,6	1,717E-05
651	649	FDXR	6,7	4,504E-03	649	RCAN2	3,5	4,048E-04	649	CCDC113	4,9	5,075E-03	649	CREG1	2,3	0,001	649	TSPAN4	3,5	6,215E-03	649	LRRC49	2,6	2,643E-04
652	650	KATNB1	6,7	2,349E-04	650	ALCAM	3,5	1,059E-04	650	MFSD2A	4,9	9,310E-04	650	SMYD3	2,3	0,000	650	BTN3A2	3,5	2,414E-05	650	PREPL	2,6	1,280E-04
653	651	LMBR1	6,7	5,110E-04	651	LRP3	3,5	8,463E-04	651	OSTM1	4,9	1,116E-05	651	SSSCA1	2,3	0,001	651	SHOX2	3,5	7,202E-05	651	FAM161B	2,6	2,738E-04
654	652	RHBDL2	6,7	1,709E-02	652	KIAA0930	3,5	7,423E-03	652	PDIA5	4,9	4,322E-05	652	HOXB9	2,3	0,001	652	KIAA0922	3,5	3,910E-03	652	TCTEX1D2	2,6	1,690E-03
655	653	SLC9A3R1	6,7	3,554E-03	653	PCDHB10	3,5	7,493E-04	653	ARVCF	4,9	1,172E-02	653	IL15	2,3	0,008	653	C15orf39	3,5	2,613E-02	653	METTL7A	2,6	1,052E-05
656	654	HPSE	6,7	1,981E-04	654	RGS19	3,5	1,085E-03	654	TSC22D1	4,9	2,510E-06	654	CFL1	2,3	0,021	654	NLRC5	3,5	2,222E-04	654	KIF21A	2,6	1,226E-03
657	655	ABAT	6,7	1,588E-03	655	RAMP2	3,5	2,450E-02	655	IQCH	4,9	4,464E-02	655	TLN1	2,3	0,000	655	FOXP1	3,5	1,558E-04	655	APOLD1	2,6	4,726E-04
658	656	CDC6	6,6	9,678E-05	656	TWF2	3,5	2,168E-04	656	CD44	4,9	3,094E-04	656	GADD45B	2,3	0,019	656	EHBP1L1	3,5	4,800E-03	656	C21orf59	2,6	1,292E-03
659	657	NR4A2	6,6	4,806E-05	657	ACTC1	3,4	2,258E-02	657	CORO1A	4,9	9,445E-04	657	GNB1L	2,3	0,033	657	FLRT2	3,5	2,614E-04	657	TMEM59L	2,6	2,186E-02
660	658	TOP2A	6,6	9,555E-05	658	SEC23A	3,4	4,063E-05	658	LRRC7	4,8	6,093E-05	658	ANPEP	2,3	0,000	658	SULF1	3,5	3,404E-03	658	NFATC1	2,6	3,482E-03
661	659	PYROXD2	6,6	2,443E-04	659	PANX1	3,4	1,761E-05	659	KCTD12	4,8	6,929E-06	659	POLM	2,3	0,003	659	SNPH	3,5	4,836E-04	659	FIGN	2,6	2,531E-03
662	660	RHOU	6,6	5,290E-04	660	DOCK9	3,4	1,343E-03	660	SEPSECS	4,8	5,432E-04	660	NDUFB7	2,3	0,009	660	POSTN	3,5	2,664E-03	660	ENPP4	2,6	1,413E-04
663	661	RBM38	6,6	3,770E-03	661	PLCB3	3,4	1,754E-04	661	CNTRL	4,8	2,836E-03	661	TFAP2A	2,3	0,000	661	FOXP4	3,5	1,985E-04	661	PRKG1	2,6	2,509E-03
664	662	TDRKH	6,6	8,813E-06	662	C1S	3,4	1,219E-05	662	GPR125	4,8	8,625E-06	662	ACTN4	2,3	0,033	662	SH3RF2	3,5	2,949E-04	662	WDR47	2,6	6,833E-05
665	663	SCUBE2	6,6	4,300E-03	663	EN1	3,4	6,099E-05	663	FBXO27	4,8	6,071E-04	663	COL15A1	2,2	0,015	663	ALDH1A1	3,4	7,713E-05	663	DZANK1	2,6	1,145E-03
666	664	PDE4B	6,6	5,238E-06	664	SPINT2	3,4	2,761E-02	664	APOBEC3F	4,8	1,911E-03	664	EPB41L4B	2,2	0,016	664	GJB2	3,4	4,525E-03	664	OSBPL11	2,6	2,450E-04
667	665	FHL1	6,5	5,843E-06	665	GPSM3	3,4	2,738E-02	665	ARL6	4,8	1,297E-03	665	PSG4	2,2	0,000	665	RPL27A	3,4	2,685E-07	665	MFSD2A	2,5	1,030E-05
668	666	NEIL3	6,5	5,454E-05	666	C2	3,4	1,714E-03	666	CYB5R2	4,8	3,973E-05	666	TMEM159	2,2	0,002	666	SLC35F2	3,4	2,390E-04	666	PSPH	2,5	2,472E-06
669	667	GINS2	6,5	2,101E-06	667	C5orf30	3,4	6,385E-04	667	ZFYVE28	4,8	8,545E-04	667	ASNS	2,2	0,000	667	PCOLCE2	3,4	4,147E-04	667	TMEM198B	2,5	4,191E-04
670	668	LYN	6,5	2,887E-05	668	ESYT3	3,4	4,514E-02	668	HIPK2	4,8	2,527E-05	668	TMEM161A	2,2	0,032	668	TRIM21	3,4	5,094E-05	668	MGP	2,5	2,988E-04
671	669	CTHRC1	6,5	1,239E-05	669	PPFIA3	3,4	1,225E-02	669	CNIH2	4,8	3,103E-04	669	KIAA1217	2,2	0,002	669	PDGFC	3,4	7,716E-03	669	CRADD	2,5	2,874E-04
672	670	CNTLN	6,5	2,085E-05	670	C10orf54	3,4	1,210E-04	670	ADSSL1	4,8	7,259E-05	670	GPR39	2,2	0,016	670	FBXL8	3,4	4,109E-03	670	SAP30	2,5	3,394E-04
673	671	DZANK1	6,5	3,128E-05	671	PHLDA3	3,4	2,251E-04	671	DTX4	4,8	6,307E-03	671	EPAS1	2,2	0,014	671	CALHM2	3,4	1,089E-04	671	SLC25A29	2,5	1,952E-03
674	672	SAMHD1	6,5	1,098E-07	672	HOOK2	3,4	8,128E-04	672	HDAC9	4,7	1,080E-03	672	NXPH4	2,2	0,003	672	LIMA1	3,4	1,175E-04	672	IQCK	2,5	2,030E-02
675	673	ID3	6,5	2,337E-03	673	AHNAK2	3,4	2,136E-05	673	ABCC1	4,7	4,011E-05	673	FMNL2	2,2	0,000	673	SCN1B	3,4	1,227E-02	673	ACHE	2,5	3,410E-02
676	674	ECM2	6,5	7,703E-05	674	ARHGEF17	3,4	2,218E-05	674	CSF1	4,7	6,815E-05	674	KIFC2	2,2	0,005	674	RPS6KA1	3,4	3,633E-04	674	MSX1	2,5	1,252E-02
677	675	PKN3	6,5	3,610E-03	675	TMED3	3,4	5,240E-07	675	STXBP2	4,7	8,183E-03	675	MZT2A	2,2	0,050	675	TRIP10	3,4	2,098E-06	675	PSPN	2,5	1,897E-03
678	676	GBP4	6,5	6,111E-04	676	PKNOX2	3,4	3,078E-05	676	STON1	4,7	5,146E-03	676	PLOD2	2,2	0,000	676	TGFBR2	3,4	7,098E-05	676	SAT2	2,5	2,779E-05
679	677	CD34	6,5	2,982E-05	677	PCDH18	3,4	1,949E-03	677	FAM171B	4,7	8,603E-06	677	FHOD3	2,2	0,009	677	MICA	3,4	3,208E-06	677	ATP2B4	2,5	1,278E-04
680	678	EZH2	6,4	9,208E-05	678	GADD45A	3,4	4,694E-04	678	EPB41L2	4,7	1,633E-05	678	NRIP1	2,2	0,001	678	LUM	3,4	5,374E-05	678	LCP1	2,5	2,281E-03
681	679	TRIM45	6,4	1,402E-05	679	PDGFC	3,4	8,152E-03	679	LIF	4,7	5,502E-04	679	UXT	2,2	0,001	679	GPR63	3,4	2,307E-04	679	DEPDC4	2,5	9,257E-03
682	680	DLK2	6,4	2,149E-03	680	ITGAV	3,3	2,686E-05	680	C3orf52	4,7	7,934E-03	680	LGALS1	2,2	0,002	680	SEZ6L2	3,3	1,068E-03	680	VLDLR	2,5	3,553E-04
683	681	RIC8B	6,4	4,039E-06	681	RPS6KA4	3,3	1,976E-04	681	ELOVL6	4,7	2,891E-03	681	FRMD6	2,2	0,000	681	IL20RB	3,3	1,463E-02	681	ZC2HC1A	2,5	3,539E-03
684	682	RMI1	6,4	1,166E-04	682	SNX33	3,3	6,340E-06	682	ABHD3	4,7	1,227E-03	682	RAB27A	2,2	0,002	682	MESDC1	3,3	4,299E-03	682	SIAE	2,5	3,797E-05
685	683	NLGN1	6,4	3,431E-03	683	UBASH3B	3,3	2,288E-03	683	POLQ	4,7	9,623E-06	683	ANG	2,2	0,035	683	LIMCH1	3,3	1,332E-02	683	IPP	2,5	7,998E-04
686	684	FUK	6,4	3,745E-03	684	LMO7	3,3	2,384E-05	684	MGST1	4,7	2,251E-04	684	TNFRSF9	2,2	0,004	684	SLC22A15	3,3	4,849E-03	684	GLDN	2,5	4,285E-02
687	685	SOX9	6,4	4,912E-05	685	DLX1	3,3	4,888E-03	685	TMEM86A	4,7	1,470E-05	685	ZNF668	2,2	0,015	685	MOXD1	3,3	1,842E-06	685	HEXIM1	2,5	6,026E-04
688	686	TRIM14	6,4	2,144E-05	686	SUGT1	3,3	1,894E-05	686	SEL1L3	4,6	2,669E-04	686	MSN	2,2	0,000	686	STAP2	3,3	2,874E-04	686	PAQR3	2,5	1,372E-02
689	687	HMGB2	6,4	6,149E-05	687	NHS	3,3	5,544E-03	687	EDN1	4,6	7,535E-05	687	UBTD1	2,2	0,003	687	SAMD9L	3,3	5,693E-05	687	CDC7	2,5	7,245E-04
690	688	CEP85	6,4	2,971E-06	688	DCAF4	3,3	9,812E-04	688	WDR52	4,6	4,699E-03	688	PJA1	2,2	0,000	688	IL11RA	3,3	1,968E-03	688	SOCS3	2,5	2,407E-04
691	689	PLTP	6,4	1,139E-03	689	GPC1	3,3	3,155E-03	689	LRRC1	4,6	1,256E-04	689	RRAS2	2,2	0,000	689	TLN2	3,3	8,346E-04	689	TTC30A	2,5	2,785E-04
692	690	CETN3	6,4	3,423E-03	690	NKX3-1	3,3	2,283E-05	690	IPMK	4,5	7,483E-05	690	RNH1	2,2	0,002	690	TRAM2	3,3	3,156E-06	690	FBXO36	2,5	2,768E-03
693	691	IZUMO4	6,4	2,453E-03	691	CIB2	3,3	6,989E-04	691	TMEM200A	4,5	2,880E-06	691	LGR4	2,2	0,001	691	RHOBTB3	3,3	5,513E-05	691	SYBU	2,5	3,238E-02
694	692	FEZ1	6,4	5,552E-05	692	DISC1	3,3	9,073E-03	692	BTN2A2	4,5	4,427E-05	692	ITGA3	2,2	0,000	692	CASP4	3,3	1,698E-02	692	NDUFS2	2,5	5,061E-04
695	693	TRIM2	6,3	1,346E-04	693	LOXL3	3,3	7,790E-05	693	TMEM154	4,5	1,190E-02	693	EPB41L4A-AS1	2,2	0,002	693	NEDD9	3,3	1,554E-05	693	ATP11C	2,5	4,897E-03
696	694	DENND2A	6,3	2,229E-05	694	SERINC2	3,3	2,751E-03	694	RBM38	4,5	3,855E-04	694	RFTN1	2,2	0,000	694	MYD88	3,3	2,491E-02	694	PUS10	2,5	6,140E-04
697	695	KLHL9	6,3	2,974E-05	695	PPP2R5B	3,3	2,416E-05	695	MFSD6	4,5	6,984E-05	695	FBXL13	2,1	0,025	695	P4HA1	3,3	4,934E-03	695	SRR	2,5	7,965E-05
698	696	TCTEX1D2	6,3	6,298E-04	696	TCP11L1	3,3	9,615E-06	696	SH3BGR	4,5	7,109E-04	696	SLC1A4	2,1	0,000	696	TMEM132A	3,3	1,666E-03	696	MPDZ	2,5	2,957E-04
699	697	PRIM1	6,3	2,617E-05	697	LAMA5	3,3	6,771E-03	697	ECM2	4,5	8,442E-08	697	NUAK1	2,1	0,001	697	C17orf96	3,3	1,075E-03	697	SGK494	2,5	2,900E-03
700	698	SH3D21	6,3	4,022E-03	698	MAPK13	3,2	1,170E-02	698	HAS2	4,5	7,273E-06	698	MAP3K6	2,1	0,005	698	QSOX1	3,3	1,971E-05	698	PFN4	2,5	4,376E-04
701	699	CHAC2	6,3	3,947E-05	699	SSSCA1	3,2	6,815E-04	699	ATP2C1	4,5	7,529E-06	699	CAMTA2	2,1	0,002	699	CD70	3,3	1,379E-03	699	LMBRD1	2,5	1,007E-03
702	700	SPEF2	6,3	6,891E-04	700	FOXP1	3,2	1,966E-03	700	DCBLD2	4,4	2,630E-05	700	HSD17B6	2,1	0,025	700	LAMB1	3,3	6,444E-04	700	WDR31	2,5	1,279E-05
703	701	WDR90	6,3	4,935E-03	701	PYCR1	3,2	3,652E-04	701	NIPSNAP3B	4,4	1,797E-02	701	CD3EAP	2,1	0,004	701	GBP2	3,3	3,044E-05	701	SETBP1	2,5	3,175E-04
704	702	WDR34	6,3	5,325E-03	702	SBNO2	3,2	1,704E-02	702	L3MBTL3	4,4	2,874E-04	702	RAB39B	2,1	0,001	702	JAG1	3,2	1,043E-06	702	SVIP	2,5	1,806E-03
705	703	IQGAP3	6,3	6,328E-07	703	BST1	3,2	1,815E-06	703	PIEZO2	4,4	1,623E-02	703	ZBTB45	2,1	0,018	703	ARHGEF19	3,2	9,349E-03	703	SV2A	2,5	8,104E-06
706	704	CENPF	6,3	1,724E-04	704	SRC	3,2	1,970E-06	704	LPP-AS2	4,4	1,503E-05	704	GXYLT2	2,1	0,006	704	LIMK1	3,2	3,321E-04	704	FMNL2	2,4	2,250E-04
707	705	MSH2	6,3	1,552E-05	705	PRICKLE2	3,2	1,914E-03	705	WWTR1	4,4	3,798E-06	705	C8orf58	2,1	0,001	705	CEBPB	3,2	1,098E-03	705	CDKN2D	2,4	6,471E-03
708	706	PSD4	6,2	1,269E-02	706	CAMTA2	3,2	1,197E-03	706	AMOTL1	4,4	1,536E-07	706	PRICKLE2	2,1	0,007	706	STX1B	3,2	1,538E-02	706	EML2	2,4	1,705E-03
709	707	FBXL2	6,2	1,934E-03	707	COMT	3,2	6,649E-03	707	PCDHB15	4,4	4,035E-04	707	FOSL2	2,1	0,000	707	FSTL3	3,2	6,020E-03	707	TOP2A	2,4	4,166E-04
710	708	DOK5	6,2	2,845E-06	708	FBXO41	3,2	2,570E-03	708	CDCA2	4,4	2,147E-04	708	MKX	2,1	0,002	708	DEPDC7	3,2	2,247E-04	708	SNHG9	2,4	2,325E-04
711	709	TRIM9	6,2	4,027E-06	709	POLD4	3,2	8,389E-03	709	RAPGEF2	4,4	8,645E-07	709	POLD2	2,1	0,001	709	AFAP1L1	3,2	9,755E-03	709	ID4	2,4	8,572E-06
712	710	IFIT2	6,2	2,410E-02	710	SAMD11	3,2	1,571E-02	710	BCL2	4,4	8,896E-04	710	APLF	2,1	0,010	710	BCAR1	3,2	5,771E-03	710	IFT172	2,4	6,727E-05
713	711	ARL6	6,2	1,447E-06	711	QSOX1	3,2	1,525E-05	711	RMI2	4,4	2,138E-04	711	GFPT2	2,1	0,000	711	LOX	3,2	1,589E-05	711	C7orf63	2,4	1,850E-03
714	712	ADAT2	6,2	4,081E-05	712	LEPREL2	3,2	2,929E-04	712	TFAP2C	4,4	1,605E-05	712	DLC1	2,1	0,000	712	PKDCC	3,2	2,602E-03	712	FZD5	2,4	3,249E-05
715	713	LRRC27	6,2	3,971E-03	713	HIF1A	3,2	1,271E-07	713	ABCA5	4,4	9,889E-04	713	SLC2A1	2,1	0,000	713	NPAS2	3,2	1,386E-03	713	ZNF19	2,4	1,594E-02
716	714	TMEM91	6,2	1,252E-03	714	SLC22A17	3,2	4,320E-03	714	RFX3	4,4	1,594E-03	714	LTBP2	2,1	0,000	714	MITF	3,2	1,857E-03	714	FAM211A	2,4	2,244E-03
717	715	HSDL2	6,2	6,503E-03	715	IL20RB	3,2	6,013E-03	715	SAMD12	4,3	9,260E-03	715	PLAUR	2,1	0,000	715	HMOX1	3,2	1,698E-06	715	TXNIP	2,4	6,150E-05
718	716	HYLS1	6,2	7,509E-06	716	BBC3	3,2	6,683E-03	716	HRH1	4,3	5,594E-03	716	MAGEH1	2,1	0,000	716	PRDM1	3,2	3,111E-04	716	HS1BP3	2,4	3,649E-04
719	717	MCM3	6,2	2,659E-06	717	ARHGAP24	3,2	5,471E-04	717	KIAA0513	4,3	1,692E-04	717	HOXD-AS1	2,1	0,009	717	TAPBPL	3,2	4,195E-04	717	URB1	2,4	2,755E-05
720	718	LINC00475	6,2	7,997E-03	718	IL6	3,2	1,311E-03	718	TYMS	4,3	4,224E-05	718	C11orf83	2,1	0,031	718	DUSP8	3,2	1,401E-02	718	PLXNB3	2,4	1,700E-02
721	719	SYTL4	6,2	3,988E-04	719	LACTB	3,2	1,050E-03	719	PYCARD	4,3	1,369E-03	719	ECM1	2,1	0,000	719	GTPBP2	3,2	1,563E-06	719	C21orf91	2,4	8,175E-03
722	720	CCDC19	6,2	7,743E-03	720	RGS10	3,1	9,158E-04	720	MXD1	4,3	1,432E-04	720	CYP1B1	2,1	0,001	720	SERPINH1	3,2	4,817E-03	720	ITM2B	2,4	6,650E-05
723	721	SALL1	6,2	8,880E-05	721	MB21D2	3,1	1,751E-03	721	MFSD11	4,3	1,056E-03	721	TSKU	2,1	0,000	721	SUSD2	3,2	1,932E-02	721	C14orf37	2,4	9,283E-03
724	722	ITM2B	6,2	1,255E-05	722	ARHGEF2	3,1	1,606E-05	722	TTC28	4,3	1,774E-05	722	FJX1	2,1	0,005	722	TESK2	3,2	1,037E-02	722	YBEY	2,4	9,105E-04
725	723	UNG	6,2	2,991E-07	723	DNER	3,1	1,891E-04	723	APOM	4,3	5,094E-03	723	C16orf45	2,1	0,002	723	FAM198B	3,1	7,589E-05	723	PRRX1	2,4	1,753E-04
726	724	TMEM56	6,2	5,357E-04	724	PAPPA	3,1	1,447E-05	724	ALDH6A1	4,3	1,044E-04	724	PHLDB1	2,1	0,008	724	GLT8D2	3,1	2,482E-03	724	FAM105A	2,4	2,154E-03
727	725	C16orf80	6,1	5,646E-06	725	HDAC5	3,1	9,372E-04	725	ANKLE1	4,3	7,497E-03	725	JDP2	2,1	0,049	725	TENC1	3,1	3,381E-03	725	BEX4	2,4	1,867E-05
728	726	ZNF738	6,1	8,856E-04	726	EMP3	3,1	4,379E-05	726	EFNB2	4,3	2,372E-07	726	CBR3	2,1	0,000	726	TSPO	3,1	4,838E-03	726	IDH2	2,4	2,721E-04
729	727	BBS2	6,1	1,267E-03	727	PTPLA	3,1	1,407E-04	727	HBEGF	4,3	2,497E-03	727	C14orf132	2,1	0,005	727	SIX1	3,1	1,623E-03	727	SRI	2,4	9,955E-06
730	728	ADSSL1	6,1	1,199E-04	728	PLOD2	3,1	1,885E-05	728	IFIT2	4,3	6,802E-05	728	RBM3	2,1	0,001	728	C1S	3,1	4,710E-06	728	HDDC3	2,4	9,848E-03
731	729	SLC25A33	6,1	1,114E-04	729	NTN4	3,1	3,501E-04	729	ANK3	4,3	1,432E-02	729	CTIF	2,1	0,003	729	LTBP2	3,1	3,062E-05	729	CBY1	2,4	2,415E-04
732	730	KIF9	6,1	4,902E-04	730	IDUA	3,1	1,712E-02	730	N4BP2	4,2	2,519E-04	730	LPXN	2,1	0,007	730	GLS	3,1	4,348E-05	730	FAM172A	2,4	4,008E-03
733	731	WDR91	6,1	3,060E-05	731	SLC35C1	3,1	8,247E-05	731	SLIT2	4,2	3,658E-05	731	NDUFA4L2	2,1	0,019	731	IQSEC2	3,1	4,042E-04	731	INPP4A	2,4	1,553E-03
734	732	TMEM116	6,1	6,140E-06	732	B4GALNT1	3,1	6,370E-03	732	RNF149	4,2	2,137E-05	732	YIPF2	2,1	0,048	732	KLHL17	3,1	6,907E-03	732	ATL1	2,3	3,961E-04
735	733	ZNF418	6,1	1,662E-04	733	XBP1	3,1	3,343E-03	733	MFSD9	4,2	4,529E-03	733	WDR18	2,1	0,019	733	DCBLD2	3,1	7,899E-05	733	NR4A2	2,3	3,178E-04
736	734	ASIC1	6,1	2,641E-04	734	MPP7	3,1	1,223E-02	734	MCCC1	4,2	4,962E-04	734	RAB6B	2,1	0,002	734	RAB27B	3,1	2,191E-04	734	WFDC3	2,3	4,903E-02
737	735	RHEBL1	6,1	1,602E-03	735	RARRES2	3,1	1,781E-03	735	KCNB1	4,2	3,434E-04	735	FCHSD2	2,1	0,001	735	COL8A2	3,1	1,802E-03	735	PTHLH	2,3	3,606E-04
738	736	CHAC1	6,1	3,160E-02	736	KIAA1462	3,1	5,585E-06	736	ZNF100	4,2	1,629E-03	736	ATP6AP2	2,1	0,000	736	CTSC	3,1	7,325E-04	736	CEP85	2,3	1,941E-04
739	737	BFSP1	6,1	2,822E-03	737	BAIAP2L1	3,1	5,681E-06	737	MARCKSL1	4,2	3,342E-04	737	C20orf112	2,0	0,006	737	ABTB2	3,1	5,411E-04	737	SPAG16	2,3	4,928E-04
740	738	HSBP1L1	6,1	3,840E-05	738	KIAA1614	3,1	4,455E-02	738	ETV1	4,2	2,665E-04	738	DKK3	2,0	0,000	738	MAPKAPK3	3,1	1,443E-05	738	C7orf31	2,3	4,530E-04
741	739	CCDC169	6,0	1,254E-03	739	GAS1	3,1	3,210E-06	739	FAM212B	4,2	6,447E-05	739	DHX34	2,0	0,006	739	SMTN	3,1	7,082E-04	739	MFHAS1	2,3	1,178E-05
742	740	KIF22	6,0	4,945E-08	740	RBFOX2	3,1	5,780E-07	740	WDR31	4,2	5,001E-03	740	REEP6	2,0	0,031	740	SYNJ2	3,1	3,258E-05	740	GRAMD1C	2,3	6,550E-03
743	741	C3orf58	6,0	7,475E-04	741	MESDC1	3,1	2,544E-02	741	MICA	4,2	5,866E-05	741	TNKS1BP1	2,0	0,001	741	PLEKHO2	3,1	1,981E-05	741	KIAA1841	2,3	4,384E-02
744	742	PLEKHG4	6,0	2,615E-03	742	HSD17B14	3,1	2,806E-03	742	LRP6	4,2	2,293E-05	742	AFAP1	2,0	0,000	742	IL17RC	3,1	2,354E-06	742	C19orf55	2,3	2,459E-04
745	743	REEP1	6,0	3,421E-05	743	NRP1	3,1	2,959E-04	743	TCTN2	4,2	1,065E-06	743	LPP	2,0	0,001	743	ADAMTSL5	3,1	2,234E-03	743	SLC37A1	2,3	2,022E-03
746	744	SEPSECS	6,0	2,888E-03	744	FARP1	3,1	4,768E-05	744	TFB1M	4,2	1,563E-05	744	RRAS	2,0	0,000	744	CRABP2	3,1	5,127E-04	744	SEPW1	2,3	2,323E-04
747	745	B9D2	6,0	1,166E-04	745	CHD3	3,1	9,798E-04	745	TSPAN3	4,2	8,218E-03	745	TCF7	2,0	0,023	745	C1R	3,1	1,833E-06	745	SFT2D2	2,3	2,058E-04
748	746	CDK20	6,0	7,431E-05	746	GCAT	3,1	4,250E-04	746	KIF11	4,2	1,653E-05	746	AOX1	2,0	0,005	746	CD276	3,1	1,958E-03	746	HNRNPU-AS1	2,3	7,401E-03
749	747	KDM4D	6,0	5,808E-05	747	FURIN	3,0	7,541E-04	747	MT1X	4,2	1,297E-05	747	ECHDC2	2,0	0,008	747	MAP2K3	3,1	2,316E-05	747	ACADVL	2,3	1,775E-04
750	748	ZWINT	6,0	5,840E-08	748	CARS2	3,0	3,013E-04	748	SSH2	4,2	9,509E-05	748	BCL3	2,0	0,008	748	RIN3	3,1	8,968E-04	748	INTS6-AS1	2,3	7,813E-04
751	749	MYO5B	6,0	2,696E-04	749	CYBRD1	3,0	1,198E-05	749	NAV2	4,2	5,411E-03	749	PTRF	2,0	0,000	749	PPP1R18	3,1	8,631E-03	749	SDC2	2,3	1,538E-06
752	750	ZCCHC18	6,0	9,799E-04	750	FBXO17	3,0	2,042E-05	750	LOH12CR2	4,1	3,751E-02	750	UGCG	2,0	0,000	750	NMI	3,1	6,597E-04	750	SFXN5	2,3	5,660E-04
753	751	ACACB	6,0	2,556E-03	751	NFASC	3,0	4,317E-03	751	RAP2B	4,1	1,998E-07	751	ANKH	2,0	0,000	751	TMEM158	3,0	5,783E-03	751	ARV1	2,3	4,263E-05
754	752	PLS1	5,9	2,908E-03	752	DUSP10	3,0	1,243E-03	752	IMPA2	4,1	3,372E-03	752	CUX1	2,0	0,001	752	FILIP1L	3,0	3,299E-05	752	CCDC167	2,3	6,502E-05
755	753	SIRT4	5,9	5,679E-03	753	DBP	3,0	3,661E-04	753	CASD1	4,1	1,157E-05	753	INSR	2,0	0,019	753	GXYLT2	3,0	1,510E-03	753	CD47	2,3	1,848E-04
756	754	UBXN11	5,9	5,375E-05	754	AFF3	3,0	1,216E-02	754	ARL13B	4,1	7,353E-04	754	CST3	2,0	0,000	754	PTPRJ	3,0	1,005E-04	754	ZNF25	2,3	1,098E-03
757	755	C16orf46	5,9	3,298E-03	755	ARMC9	3,0	1,474E-05	755	FOSL1	4,1	9,672E-03	755	PMP22	2,0	0,000	755	ZYX	3,0	6,462E-04	755	LRRC27	2,3	1,855E-02
758	756	WDR76	5,9	4,502E-03	756	EPG5	3,0	2,351E-02	756	E2F2	4,1	1,031E-03	756	PLXNB3	2,0	0,006	756	SLC19A1	3,0	2,573E-02	756	LCLAT1	2,3	2,596E-03
759	757	GDF15	5,9	2,615E-03	757	ACVR2A	3,0	5,254E-04	757	NFE2L3	4,1	2,176E-05	757	GLI4	2,0	0,003	757	PNPT1	3,0	4,678E-06	757	DTD1	2,3	2,661E-05
760	758	STAMBPL1	5,9	4,856E-05	758	CEBPB	3,0	7,752E-03	758	GDF15	4,1	2,169E-03	758	TMEM51	2,0	0,026	758	IER5L	3,0	2,537E-02	758	TATDN3	2,3	1,584E-04
761	759	FICD	5,9	1,822E-04	759	SLC25A37	3,0	2,115E-03	759	KREMEN1	4,1	2,543E-05	759	PTPN14	2,0	0,000	759	AMIGO2	3,0	1,220E-02	759	C1orf228	2,3	1,441E-02
762	760	MND1	5,9	1,286E-04	760	CD151	3,0	2,256E-05	760	EZR	4,1	4,802E-07	760	TNC	2,0	0,002	760	P4HA2	3,0	4,878E-06	760	C8orf58	2,3	2,321E-03
763	761	PSRC1	5,9	4,756E-04	761	BCAR1	3,0	2,145E-02	761	SH3BP4	4,1	8,118E-05	761	SIX5	2,0	0,017	761	FMOD	3,0	2,074E-03	761	CBX5	2,3	5,147E-04
764	762	SFXN2	5,9	3,102E-03	762	SLC7A8	3,0	6,867E-04	762	CLSTN3	4,1	6,118E-05					762	DLC1	3,0	2,235E-04	762	BAG1	2,3	1,423E-02
765	763	KLF5	5,8	6,127E-07	763	TRIM8	3,0	7,585E-04	763	MEX3A	4,1	4,445E-05					763	ITPR1	3,0	6,307E-03	763	DACH1	2,3	3,976E-05
766	764	MIR600HG	5,8	1,453E-02	764	STXBP6	2,9	8,834E-04	764	TERF2IP	4,1	2,511E-03					764	SERTAD3	3,0	2,590E-03	764	TMCO1	2,3	1,090E-04
767	765	FBXL14	5,8	3,677E-03	765	CHSY1	2,9	9,364E-05	765	TMEM144	4,1	3,057E-03					765	SLC43A3	3,0	2,042E-05	765	S100A10	2,3	3,522E-06
768	766	C21orf49	5,8	3,210E-02	766	PPP3CC	2,9	2,587E-06	766	TPST1	4,1	3,664E-06					766	FJX1	3,0	1,006E-03	766	RDX	2,3	1,579E-03
769	767	HIST1H4H	5,8	1,661E-03	767	DISP2	2,9	1,476E-02	767	ZNF853	4,1	4,889E-04					767	APOL3	3,0	4,005E-04	767	CGN	2,3	7,496E-04
770	768	COQ4	5,8	6,725E-04	768	DLGAP4	2,9	2,913E-03	768	CEP19	4,0	8,546E-06					768	LGALS3BP	3,0	3,035E-03	768	RIC8B	2,3	4,820E-04
771	769	HEXIM1	5,8	1,530E-04	769	LAMC1	2,9	1,205E-05	769	SYNGR2	4,0	1,482E-02					769	ERRFI1	3,0	2,184E-04	769	PHTF1	2,3	4,148E-05
772	770	MREG	5,8	3,132E-04	770	CLSTN2	2,9	3,542E-05	770	GAS8	4,0	8,663E-03					770	TMEM51	3,0	7,968E-03	770	C3orf58	2,3	1,013E-03
773	771	IFI35	5,8	3,972E-05	771	LEPREL4	2,9	6,176E-04	771	CPZ	4,0	2,090E-02					771	TFPI	3,0	1,444E-04	771	KIAA1715	2,3	2,090E-04
774	772	COBLL1	5,8	1,813E-02	772	MXRA5	2,9	1,382E-05	772	ATF6	4,0	3,428E-07					772	TGFB1	3,0	2,532E-03	772	DYNC2H1	2,3	6,579E-04
775	773	PELI2	5,8	3,461E-05	773	GLTSCR2	2,9	1,599E-03	773	ADAM17	4,0	6,270E-07					773	PDLIM4	3,0	5,317E-03	773	MKL2	2,3	5,631E-03
776	774	ZNF274	5,8	1,180E-04	774	EEPD1	2,9	5,271E-04	774	PELI1	4,0	3,806E-05					774	FBXO41	3,0	7,907E-04	774	MORC4	2,3	9,548E-03
777	775	PSMC3IP	5,8	1,987E-05	775	PAPSS2	2,9	1,478E-04	775	IQCK	4,0	5,046E-03					775	INSIG1	3,0	9,463E-03	775	CDKN2C	2,3	2,517E-04
778	776	ZNF470	5,8	2,407E-03	776	KCNG1	2,9	1,637E-02	776	PARK2	4,0	5,503E-03					776	C1QTNF1	2,9	4,058E-02	776	FAM71E1	2,3	3,423E-02
779	777	FGF2	5,8	6,260E-05	777	COL6A1	2,9	2,336E-03	777	DCHS1	4,0	1,858E-04					777	RGS19	2,9	9,945E-04	777	SLC27A3	2,3	2,757E-04
780	778	MDM1	5,7	4,067E-05	778	PPIC	2,9	2,630E-06	778	NFE2L2	4,0	4,145E-02					778	NXPH4	2,9	6,095E-03	778	INSIG2	2,3	4,781E-04
781	779	DDR1	5,7	2,987E-04	779	RRAS2	2,9	3,284E-05	779	LYST	4,0	2,771E-04					779	IFIT5	2,9	2,275E-04	779	B9D1	2,3	4,633E-05
782	780	LOH12CR2	5,7	1,798E-03	780	MAP1A	2,9	4,855E-05	780	ALDH5A1	4,0	1,279E-04					780	SOD2	2,9	2,649E-05	780	KCNJ8	2,3	1,227E-03
783	781	BTG3	5,7	6,110E-05	781	DKK3	2,9	1,186E-04	781	ADAT2	4,0	3,293E-03					781	LEPREL2	2,9	5,785E-03	781	SDC3	2,3	8,997E-04
784	782	TPBG	5,7	4,443E-06	782	NT5E	2,9	1,462E-04	782	GNG11	4,0	9,637E-09					782	RGS10	2,9	8,366E-05	782	DUSP14	2,3	2,150E-06
785	783	TMEM107	5,7	1,102E-04	783	PLXNA2	2,9	3,417E-03	783	FGFR1	4,0	6,518E-03					783	B2M	2,9	2,865E-04	783	VIM	2,3	1,401E-04
786	784	MYO1B	5,6	1,475E-04	784	UAP1L1	2,9	5,761E-03	784	FANCL	4,0	7,232E-03					784	MASP1	2,9	2,308E-03	784	TMEM8B	2,3	6,757E-03
787	785	MCM2	5,6	2,707E-05	785	SH3BGRL3	2,9	4,269E-06	785	WDR78	4,0	8,045E-04					785	PLAT	2,9	7,838E-03	785	HOXD10	2,2	3,095E-04
788	786	FBXO36	5,6	7,377E-04	786	CAPN2	2,9	3,552E-08	786	KIAA1598	4,0	5,465E-05					786	UBE2B	2,9	8,065E-04	786	C5	2,2	4,682E-04
789	787	FNDC4	5,6	2,705E-06	787	SLC22A23	2,8	2,316E-04	787	IGSF3	4,0	4,582E-06					787	JAK2	2,9	7,569E-04	787	PRDX6	2,2	4,138E-06
790	788	C21orf58	5,6	6,869E-04	788	ACSF2	2,8	1,132E-04	788	C16orf52	4,0	7,117E-03					788	KDELR3	2,9	1,919E-03	788	UBXN11	2,2	5,142E-04
791	789	REPS2	5,6	2,043E-03	789	PLOD1	2,8	5,902E-07	789	BPGM	4,0	5,271E-05					789	NR1D2	2,9	5,421E-03	789	GSTA4	2,2	3,593E-03
792	790	BTN2A2	5,6	4,423E-06	790	FLT3LG	2,8	1,939E-03	790	MINPP1	4,0	3,857E-05					790	TBX3	2,9	1,291E-04	790	POLE4	2,2	2,198E-02

	A	B	C	D	E	F	G	H	I	J	K	L	M	N	O	P	Q	R	S	T	U	V	W	X
793	791	MFSD6	5,6	6,810E-04	791	ADCY7	2,8	6,699E-03	791	RBMXL1	4,0	1,064E-05					791	BTN3A1	2,9	4,946E-05	791	CFI	2,2	2,080E-04
794	792	CDC14A	5,6	1,372E-03	792	DAPK1	2,8	4,865E-03	792	NT5DC3	4,0	2,401E-05					792	NR4A1	2,9	1,184E-02	792	PCDH9	2,2	2,488E-04
795	793	PARP9	5,6	5,046E-05	793	P2RX6	2,8	2,026E-02	793	ZNF652	4,0	3,549E-02					793	MEIS3	2,9	3,010E-04	793	SLC35D1	2,2	5,403E-05
796	794	APITD1	5,6	1,774E-06	794	ANKRD30BL	2,8	1,022E-02	794	APOBEC3B	4,0	1,022E-04					794	VGLL3	2,9	2,467E-04	794	DYNC2LI1	2,2	1,646E-03
797	795	BTBD9	5,6	1,033E-05	795	FLNA	2,8	2,626E-04	795	USP54	4,0	1,349E-03					795	HRH1	2,9	6,750E-05	795	RAB4A	2,2	7,342E-05
798	796	SPRY1	5,6	1,283E-03	796	TMSB10	2,8	2,699E-06	796	SIRT4	3,9	2,311E-02					796	TMEM198	2,9	4,971E-03	796	ITGAE	2,2	5,460E-03
799	797	ODC1	5,6	1,795E-04	797	NRP2	2,8	4,021E-03	797	NFIB	3,9	5,439E-03					797	SLC29A3	2,9	4,290E-02	797	CLCN4	2,2	7,187E-06
800	798	C7orf63	5,6	7,904E-04	798	PIK3IP1	2,8	3,944E-02	798	IMMP2L	3,9	2,449E-03					798	JARID2	2,9	4,443E-05	798	TP53TG1	2,2	1,345E-03
801	799	TDRD7	5,6	1,270E-06	799	NME4	2,8	3,976E-03	799	SGK494	3,9	6,197E-03					799	ECE1	2,9	2,044E-05	799	CLCN2	2,2	3,503E-04
802	800	XRCC2	5,6	3,600E-05	800	SLC2A4RG	2,8	3,563E-03	800	ARMC4	3,9	2,087E-05					800	TFAP2A	2,9	7,068E-04	800	MORN4	2,2	7,777E-04
803	801	MAP3K14	5,5	7,121E-05	801	CHST7	2,8	4,103E-02	801	RBM47	3,9	1,684E-03					801	FTL	2,9	6,454E-07	801	LCAT	2,2	8,586E-03
804	802	CC2D2A	5,5	4,649E-03	802	PRDM1	2,8	2,915E-03	802	ATP6V1A	3,9	4,635E-07					802	TLE4	2,9	1,052E-04	802	CCDC85C	2,2	8,187E-04
805	803	AQP3	5,5	4,382E-03	803	ASPHD1	2,8	4,449E-03	803	FANK1	3,9	1,018E-02					803	MOCS1	2,9	2,580E-04	803	TARSL2	2,2	4,237E-03
806	804	RTTN	5,5	4,763E-06	804	NFKBIZ	2,8	1,783E-03	804	LHFPL2	3,9	7,335E-08					804	GSN	2,9	2,570E-03	804	LMBRD2	2,2	1,289E-03
807	805	PRICKLE1	5,5	1,140E-03	805	SEC24D	2,8	3,184E-07	805	NR6A1	3,9	2,592E-04					805	REC8	2,9	5,785E-04	805	WWOX	2,2	4,056E-03
808	806	SLC37A4	5,5	1,163E-04	806	GAD1	2,8	4,456E-05	806	SIPA1L2	3,9	3,834E-05					806	HNMT	2,8	1,983E-02	806	TTC5	2,2	9,694E-05
809	807	AIM1	5,5	8,683E-05	807	HOXA1	2,8	1,058E-03	807	RBM24	3,9	1,643E-03					807	SIM2	2,8	4,026E-03	807	APITD1	2,2	5,887E-05
810	808	LRRC4	5,5	9,115E-05	808	FAM89B	2,8	3,259E-02	808	LYPD1	3,9	3,192E-05					808	DISP2	2,8	1,473E-03	808	ZNF69	2,2	1,837E-02
811	809	ZNF680	5,5	5,714E-04	809	DBN1	2,8	3,310E-04	809	NEK6	3,9	8,599E-07					809	IKBKE	2,8	1,391E-04	809	GCLM	2,2	2,482E-03
812	810	BARD1	5,5	3,028E-04	810	MKNK2	2,8	2,784E-05	810	PER2	3,9	8,484E-04					810	RSPO3	2,8	6,057E-03	810	PLD2	2,2	3,742E-04
813	811	E2F2	5,5	1,033E-04	811	TACC2	2,8	5,646E-03	811	NFATC1	3,9	6,471E-03					811	TNK2	2,8	1,627E-02	811	HIP1	2,2	3,340E-05
814	812	EFCAB7	5,5	7,065E-05	812	PTPRG	2,8	7,938E-04	812	PDP2	3,9	1,189E-06					812	LEPRE1	2,8	1,371E-05	812	CCDC159	2,2	1,852E-03
815	813	C1orf56	5,5	2,504E-03	813	AMPH	2,8	1,051E-04	813	CCDC28A	3,9	7,415E-06					813	TNC	2,8	3,325E-04	813	WDR54	2,2	2,491E-05
816	814	CDNF	5,5	3,329E-02	814	SLC2A6	2,8	6,003E-03	814	CCDC169	3,9	2,451E-02					814	TMEM87B	2,8	2,199E-04	814	NCEH1	2,2	2,277E-03
817	815	PDZD2	5,5	1,493E-02	815	SPATA20	2,8	3,461E-04	815	FNDC3A	3,8	1,391E-05					815	HOXB9	2,8	2,511E-06	815	ZNF664	2,2	3,222E-04
818	816	OSBP2	5,5	3,625E-04	816	PPIB	2,8	2,577E-06	816	TTLL7	3,8	2,301E-03					816	REV3L	2,8	1,279E-04	816	C21orf119	2,2	1,902E-02
819	817	COQ3	5,5	5,023E-05	817	SAMD4A	2,8	6,210E-05	817	SAV1	3,8	1,676E-04					817	CD164	2,8	4,178E-03	817	NAP1L5	2,2	1,188E-03
820	818	SLC25A27	5,5	1,123E-02	818	CABLES1	2,8	1,868E-02	818	OSGIN2	3,8	6,745E-03					818	EMILIN2	2,8	5,164E-03	818	GOLIM4	2,2	4,643E-05
821	819	RAMP1	5,5	9,181E-03	819	S1PR2	2,8	6,470E-03	819	HLA-DMA	3,8	9,872E-04					819	SLC25A28	2,8	6,545E-05	819	RAP2A	2,2	4,303E-05
822	820	SHCBP1	5,5	5,442E-07	820	PIM3	2,7	1,033E-02	820	PARP8	3,8	2,832E-03					820	LOXL3	2,8	1,440E-04	820	MTM1	2,2	9,154E-03
823	821	KCTD1	5,4	8,927E-06	821	LZTS2	2,7	3,654E-02	821	SOX13	3,8	6,790E-05					821	TRIM22	2,8	4,319E-02	821	OSBP2	2,2	1,465E-03
824	822	CADM4	5,4	3,406E-04	822	PCDHB2	2,7	2,370E-05	822	CHCHD10	3,8	7,255E-04					822	AGMAT	2,8	5,193E-03	822	NSUN5	2,2	3,189E-02
825	823	LRTOMT	5,4	2,535E-03	823	P4HB	2,7	1,067E-03	823	SLC9A9	3,8	2,572E-03					823	IFI16	2,8	5,143E-04	823	ANXA1	2,2	3,418E-04
826	824	REM1	5,4	1,146E-02	824	BCAM	2,7	1,219E-03	824	CNNM4	3,8	2,600E-05					824	RASSF3	2,8	3,047E-05	824	AGTPBP1	2,2	2,745E-05
827	825	FLVCR1	5,4	9,077E-05	825	TMEM45A	2,7	2,716E-03	825	USP53	3,8	3,405E-03					825	ST3GAL5	2,8	1,929E-05	825	TMEM99	2,2	2,238E-04
828	826	PARK2	5,4	2,240E-02	826	BICC1	2,7	9,102E-05	826	TASP1	3,8	1,209E-03					826	FAP	2,8	6,253E-03	826	NUF2	2,2	1,695E-04
829	827	LYPD6	5,4	2,122E-04	827	GPR63	2,7	3,195E-03	827	ATP6V1B2	3,8	1,348E-05					827	STARD8	2,8	3,307E-04	827	ZNF275	2,2	2,221E-02
830	828	PBX1	5,4	5,249E-06	828	THSD4	2,7	2,445E-04	828	DOK4	3,8	1,556E-04					828	KCNMA1	2,8	7,364E-04	828	ESCO2	2,2	2,413E-03
831	829	CENPQ	5,4	4,207E-04	829	RGMB	2,7	1,404E-04	829	CLSPN	3,8	7,042E-05					829	MFGE8	2,8	2,004E-05	829	CHMP2A	2,2	1,374E-03
832	830	ABCA5	5,4	2,646E-03	830	NCK2	2,7	2,138E-04	830	PAQR8	3,8	2,655E-05					830	ETS1	2,8	8,777E-07	830	GPR1	2,2	6,468E-03
833	831	DCAF12	5,4	9,385E-06	831	SNX18	2,7	2,463E-05	831	TTYH2	3,8	1,204E-02					831	KAZALD1	2,8	2,393E-05	831	IFT122	2,2	3,461E-05
834	832	RABL5	5,4	6,493E-06	832	RUNX2	2,7	2,077E-04	832	MET	3,8	1,074E-03					832	FAM124A	2,8	9,627E-04	832	DSTYK	2,2	4,350E-04
835	833	ZBTB10	5,4	9,482E-04	833	PILRB	2,7	4,878E-02	833	PLA2G15	3,8	3,594E-02					833	SP110	2,7	2,516E-05	833	PCDHB7	2,2	4,977E-04
836	834	SFR1	5,4	2,529E-03	834	ANXA6	2,7	3,112E-07	834	TRERF1	3,8	4,633E-04					834	DDO	2,7	1,680E-04	834	TMEM66	2,2	3,076E-03
837	835	SHMT1	5,4	1,655E-04	835	CACNB1	2,7	5,450E-03	835	TRIM45	3,8	5,294E-05					835	ZNF395	2,7	1,589E-03	835	FAM213A	2,2	1,312E-04
838	836	C8orf37	5,4	1,915E-03	836	GAS2L1	2,7	4,853E-02	836	UBD	3,8	6,668E-04					836	ZNRF1	2,7	1,144E-02	836	FAS	2,2	4,593E-02
839	837	ASF1B	5,4	2,641E-07	837	TPCN1	2,7	6,683E-03	837	STK32B	3,8	2,161E-03					837	BID	2,7	6,434E-03	837	FBXW9	2,2	6,110E-03
840	838	TMEM38B	5,4	2,053E-03	838	ERO1L	2,7	1,267E-03	838	MAFF	3,8	4,497E-05					838	RHEBL1	2,7	1,287E-04	838	CDC42EP4	2,2	1,431E-03
841	839	LINC00094	5,4	2,429E-05	839	RBPMS	2,7	4,769E-04	839	ASAH1	3,8	2,096E-04					839	IL17RD	2,7	1,910E-04	839	KIF14	2,2	5,367E-04
842	840	PLK1	5,4	6,672E-03	840	PPP3CA	2,7	3,888E-04	840	GAL3ST4	3,8	1,065E-05					840	TNFAIP2	2,7	1,630E-03	840	ZDHHC1	2,2	2,959E-02
843	841	DNASE1	5,4	5,185E-04	841	PRUNE2	2,7	3,885E-05	841	PRPSAP2	3,8	1,764E-04					841	GTF2IRD1	2,7	6,549E-05	841	ZNF462	2,2	1,635E-02
844	842	CDK1	5,4	1,338E-03	842	RPL10	2,7	5,133E-04	842	EEA1	3,8	1,063E-05					842	PPIC	2,7	1,144E-06	842	CMTM4	2,2	9,375E-03
845	843	ZNF84	5,4	1,094E-03	843	RPS6KA2	2,7	5,868E-06	843	IRF2	3,8	1,778E-04					843	N4BP1	2,7	4,033E-06	843	BTBD9	2,2	1,785E-04
846	844	OSCP1	5,3	1,975E-05	844	PLIN3	2,7	1,068E-03	844	MAGI3	3,8	2,601E-06					844	KIF7	2,7	4,673E-03	844	BBS5	2,2	1,818E-03
847	845	APLP1	5,3	9,342E-06	845	MGAT4B	2,6	1,244E-03	845	CRELD1	3,7	2,773E-03					845	B4GALNT4	2,7	3,109E-02	845	IQCC	2,2	8,595E-04
848	846	ZNF137P	5,3	1,771E-02	846	SEMA3F	2,6	1,560E-03	846	SPINT2	3,7	3,314E-04					846	BNIP3	2,7	8,282E-05	846	FBXO10	2,2	8,700E-03
849	847	NDE1	5,3	1,787E-04	847	PIWIL4	2,6	1,455E-02	847	ISOC1	3,7	5,920E-06					847	TICAM1	2,7	1,028E-03	847	FAM132B	2,2	2,730E-03
850	848	METTL7B	5,3	8,115E-05	848	WDR18	2,6	1,530E-02	848	CCNE2	3,7	7,091E-05					848	DSE	2,7	2,013E-06	848	CETN3	2,2	2,384E-02
851	849	N4BP2L1	5,3	1,567E-02	849	GALNT10	2,6	2,048E-06	849	HELQ	3,7	8,922E-03					849	LTBP1	2,7	7,190E-03	849	DSTN	2,2	2,581E-05
852	850	ACADS	5,3	2,921E-04	850	PDLIM4	2,6	2,099E-02	850	DUSP8	3,7	3,108E-04					850	DRAP1	2,7	1,105E-03	850	KCTD12	2,2	4,891E-05
853	851	ACTR6	5,3	1,857E-02	851	FERMT2	2,6	2,274E-04	851	NAB1	3,7	6,251E-03					851	LZTS2	2,7	1,046E-02	851	SLC35B4	2,2	2,011E-04
854	852	HINT2	5,3	2,344E-05	852	VWA1	2,6	3,243E-02	852	PARP14	3,7	5,301E-05					852	CNKSR3	2,7	1,118E-05	852	DHX57	2,2	1,147E-03
855	853	PAQR8	5,3	3,630E-04	853	WIPI1	2,6	4,676E-05	853	BLM	3,7	2,967E-02					853	HCN2	2,7	7,053E-03	853	SKA2	2,2	7,577E-04
856	854	RRM2	5,3	3,057E-04	854	PIK3CD	2,6	2,834E-02	854	INO80C	3,7	3,526E-05					854	FAM211B	2,7	1,142E-02	854	TMEM107	2,1	8,701E-04
857	855	TTC30A	5,3	5,370E-05	855	FAH	2,6	4,491E-04	855	FUT8	3,7	2,616E-03					855	PLXNA2	2,7	1,403E-03	855	SPIRE2	2,1	8,932E-04
858	856	SLC4A11	5,3	5,312E-03	856	PPP1R3B	2,6	8,242E-04	856	ACSS1	3,7	1,010E-02					856	EHD4	2,7	3,300E-05	856	KIFAP3	2,1	1,778E-04
859	857	C11orf71	5,3	6,273E-04	857	BCAS4	2,6	1,594E-02	857	BDH1	3,7	2,546E-04					857	ULBP2	2,7	2,450E-05	857	MAN2A1	2,1	1,934E-04
860	858	FAM46B	5,3	7,600E-04	858	SHOX2	2,6	5,099E-03	858	ACHE	3,7	2,957E-02					858	AOX1	2,7	4,430E-03	858	GNG11	2,1	5,308E-05
861	859	LAMA1	5,3	6,783E-05	859	CBLB	2,6	1,299E-03	859	C3AR1	3,7	6,784E-03					859	GPR176	2,7	5,760E-04	859	CES2	2,1	1,337E-03
862	860	HSF2	5,3	1,044E-04	860	ANO10	2,6	1,737E-03	860	S1PR5	3,7	1,380E-02					860	DUSP23	2,7	3,658E-03	860	PEX11G	2,1	1,171E-02
863	861	CENPI	5,3	4,623E-04	861	SLC2A1	2,6	1,449E-05	861	CEP85	3,7	4,407E-03					861	SPSB1	2,7	2,134E-04	861	TOR3A	2,1	5,764E-04
864	862	RFX2	5,3	4,106E-04	862	TIMP1	2,6	1,102E-05	862	EPN2	3,7	2,749E-03					862	TMEM217	2,7	6,917E-03	862	PARP1	2,1	3,163E-06
865	863	EXOC6	5,3	9,285E-04	863	MVP	2,6	1,305E-04	863	ID3	3,7	4,146E-05					863	BMP4	2,7	4,730E-02	863	HSDL2	2,1	3,067E-02
866	864	ZNF169	5,3	1,881E-05	864	TMEM109	2,6	2,841E-03	864	AP1G1	3,7	3,954E-05					864	FASN	2,7	5,458E-03	864	PDIK1L	2,1	3,575E-04
867	865	GSTM4	5,3	1,541E-04	865	R3HDM4	2,6	5,936E-04	865	CRISPLD2	3,7	4,388E-06					865	EIF2AK2	2,7	6,801E-05	865	MAP3K13	2,1	2,540E-04
868	866	SPAG5	5,3	2,188E-05	866	TBC1D8B	2,6	1,811E-05	866	MAGEF1	3,7	1,742E-07					866	UGCG	2,7	1,505E-04	866	PDK3	2,1	1,281E-05
869	867	ZNF48	5,2	1,478E-06	867	IGSF10	2,6	3,793E-04	867	ZSCAN23	3,7	3,083E-02					867	CARD10	2,7	6,260E-04	867	FAM135A	2,1	4,982E-04
870	868	RAD51AP1	5,2	9,275E-04	868	GXYLT2	2,6	2,168E-03	868	RCN2	3,7	9,253E-05					868	RARG	2,7	1,262E-03	868	ZNF577	2,1	5,568E-04
871	869	IFIT3	5,2	1,130E-02	869	C1orf198	2,6	1,336E-05	869	TMX4	3,7	1,278E-06					869	TCOF1	2,7	7,236E-04	869	HK1	2,1	3,523E-05
872	870	SPIRE2	5,2	8,456E-05	870	TNK2	2,6	1,400E-02	870	DPF1	3,7	2,511E-03					870	NF2	2,7	9,821E-03	870	ENSA	2,1	1,818E-04
873	871	FAM111B	5,2	9,045E-03	871	C19orf12	2,6	1,585E-03	871	AKAP9	3,7	1,133E-04					871	MAP3K11	2,7	8,289E-03	871	TP53INP2	2,1	1,119E-04
874	872	DYNC2LI1	5,2	2,985E-04	872	ANO7	2,6	1,445E-02	872	KIAA1147	3,7	1,863E-06					872	OGFR	2,7	5,141E-03	872	C20orf96	2,1	4,417E-04
875	873	USP54	5,2	2,750E-03	873	ADARB1	2,6	3,130E-04	873	PLAU	3,7	7,263E-05					873	RARA	2,7	1,008E-03	873	WDR92	2,1	9,600E-04
876	874	FANCD2	5,2	3,950E-05	874	CPA4	2,6	1,910E-04	874	PLCD4	3,7	2,020E-03					874	NPDC1	2,6	2,448E-02	874	CGNL1	2,1	1,075E-04
877	875	TMEM86B	5,2	1,133E-02	875	CLTCL1	2,6	7,580E-04	875	TANC1	3,7	1,725E-04					875	DFNB31	2,6	1,767E-03	875	DYX1C1	2,1	3,613E-02
878	876	FANCC	5,2	6,240E-07	876	SECISBP2L	2,6	1,527E-02	876	BEX4	3,7	2,233E-05					876	PFKP	2,6	5,267E-04	876	OGDH	2,1	3,245E-04
879	877	ALDH3A2	5,2	7,027E-03	877	JDP2	2,6	2,292E-02	877	NDFIP2	3,7	4,250E-09					877	OLFML2A	2,6	3,897E-04	877	KIAA0556	2,1	2,897E-04
880	878	CDC42EP4	5,2	2,762E-04	878	MYOF	2,5	3,646E-03	878	SAMD9	3,7	6,204E-06					878	NKX3-1	2,6	1,159E-04	878	RHOU	2,1	5,915E-03
881	879	ZNF100	5,2	4,035E-04	879	ZNF697	2,5	4,191E-05	879	LINC00338	3,7	1,787E-02					879	TRIM69	2,6	2,251E-04	879	DNAL1	2,1	5,088E-03
882	880	TIMELESS	5,2	2,088E-06	880	C16orf45	2,5	1,385E-03	880	ZNF771	3,7	2,502E-03					880	TRERF1	2,6	2,804E-06	880	MAGEH1	2,1	2,043E-04
883	881	GPRC5A	5,2	4,490E-04	881	PDK1	2,5	1,997E-02	881	ID4	3,7	2,613E-03					881	CYB5R2	2,6	1,976E-04	881	ZNF566	2,1	4,109E-02
884	882	ZNF273	5,2	2,459E-04	882	FILIP1L	2,5	3,069E-06	882	NID1	3,7	3,131E-06					882	IER5	2,6	2,853E-05	882	MZF1	2,1	1,519E-02
885	883	COQ7	5,2	6,893E-04	883	LRIG1	2,5	1,414E-05	883	CKAP4	3,6	1,399E-06					883	EGFR	2,6	1,416E-06	883	ZNF780A	2,1	2,860E-02
886	884	TMEFF2	5,2	5,895E-03	884	FBXL7	2,5	2,534E-04	884	SLC15A4	3,6	9,524E-05					884	ARHGEF2	2,6	4,857E-04	884	HAGHL	2,1	3,376E-02
887	885	RASSF8	5,2	6,882E-05	885	CLIC3	2,5	1,517E-02	885	NECAP1	3,6	1,187E-04					885	TFEB	2,6	5,422E-04	885	DPCD	2,1	3,001E-04
888	886	ZNF891	5,2	4,052E-04	886	TRAF3IP2	2,5	5,271E-03	886	SDF2	3,6	4,927E-03					886	MDK	2,6	2,038E-03	886	E2F5	2,1	2,156E-02
889	887	AASS	5,2	2,001E-04	887	SLC52A2	2,5	1,991E-02	887	ITM2B	3,6	1,497E-05					887	CLSTN3	2,6	6,703E-04	887	RIMKLB	2,1	8,188E-05
890	888	AMN1	5,2	3,693E-04	888	KIAA1522	2,5	2,964E-04	888	LRP8	3,6	7,650E-03					888	HOXC13	2,6	1,606E-04	888	IFT80	2,1	1,367E-03
891	889	ADCK3	5,2	3,736E-04	889	TBC1D17	2,5	1,848E-03	889	FAM105A	3,6	5,579E-04					889	ALDH1B1	2,6	6,230E-07	889	AGAP1	2,1	3,934E-03
892	890	HIPK1	5,2	6,170E-03	890	SSH3	2,5	1,123E-03	890	SLC25A40	3,6	3,474E-06					890	PYCR1	2,6	2,913E-02	890	MYEF2	2,1	3,833E-04
893	891	EPHA3	5,1	1,157E-04	891	LRRFIP1	2,5	1,349E-05	891	SCAMP5	3,6	3,572E-02					891	MAP3K1	2,6	7,011E-05	891	CCDC169	2,1	2,351E-02
894	892	ZDHHC21	5,1	9,583E-04	892	NDEL1	2,5	1,550E-03	892	SNX4	3,6	3,852E-06					892	ARHGAP4	2,6	1,322E-02	892	PIGF	2,1	1,242E-03
895	893	CPZ	5,1	6,171E-04	893	SH2D4A	2,5	7,502E-04	893	ANKRD28	3,6	2,623E-06					893	MYO1B	2,6	1,166E-04	893	CEP112	2,1	2,940E-03
896	894	SLC25A4	5,1	1,194E-04	894	CCIN	2,5	4,098E-04	894	TNFAIP3	3,6	2,979E-04					894	LRP5	2,6	3,444E-03	894	MREG	2,1	2,846E-04
897	895	RACGAP1	5,1	1,873E-05	895	TRIM38	2,5	2,967E-05	895	SEC14L1	3,6	3,972E-06					895	RAB23	2,6	1,320E-05	895	ERLIN2	2,1	1,283E-04
898	896	TMEM106C	5,1	1,716E-05	896	KSR1	2,5	2,131E-02	896	C16orf93	3,6	1,173E-02					896	CH25H	2,6	2,793E-04	896	IRAK1BP1	2,1	1,086E-03
899	897	HNRNPF	5,1	9,717E-07	897	PDZRN3	2,5	3,015E-04	897	CCDC121	3,6	2,579E-03					897	PHLDB2	2,6	7,687E-05	897	TDO2	2,1	1,356E-03
900	898	TASP1	5,1	2,599E-04	898	TAF1D	2,5	1,336E-06	898	C16orf46	3,6	8,516E-03					898	LDOC1	2,6	4,770E-04	898	GNPAT	2,1	8,138E-04
901	899	CDKN1A	5,1	1,823E-05	899	BMF	2,5	3,135E-03	899	PSTPIP2	3,6	3,575E-03					899	DDT	2,6	1,075E-02	899	UBE2T	2,1	5,041E-04
902	900	DEPDC1B	5,1	1,296E-04	900	STX3	2,5	1,027E-02	900	GMDS	3,6	1,991E-04					900	SERTAD1	2,6	1,478E-05	900	PITPNM2	2,1	2,158E-03
903	901	LRRC20	5,1	2,495E-02	901	ALPK2	2,5	2,550E-04	901	OSBP2	3,6	4,671E-03					901	RASSF1	2,6	4,041E-04	901	TMEM38B	2,1	1,249E-02
904	902	YEATS4	5,1	1,603E-03	902	ADRBK2	2,5	5,983E-04	902	TMEM133	3,6	3,118E-06					902	L3MBTL3	2,6	1,811E-04	902	KIAA0895	2,1	1,312E-02
905	903	SYT11	5,1	1,763E-05	903	TMEM87B	2,5	1,587E-04	903	SMAGP	3,6	2,687E-03					903	ASPHD2	2,6	7,071E-04	903	ALG6	2,1	3,577E-02
906	904	MGST1	5,1	1,626E-04	904	BID	2,5	8,219E-03	904	GPD2	3,6	3,256E-04					904	AFF3	2,6	2,404E-02	904	TAF9B	2,1	5,790E-05
907	905	CPNE2	5,1	3,955E-07	905	SEMA3B	2,5	6,691E-03	905	TNRC6C	3,6	1,207E-02					905	ADAMTS12	2,6	2,196E-04	905	HOXA7	2,1	3,789E-02
908	906	STAG3L4	5,1	2,553E-04	906	PIP5K1C	2,5	5,346E-03	906	HOXC13	3,6	6,450E-04					906	LIMD2	2,6	2,253E-02	906	LIG1	2,1	5,758E-04
909	907	WWC1	5,1	7,954E-04	907	RAB11FIP5	2,5	1,569E-02	907	SORBS1	3,6	1,215E-02					907	MEX3A	2,6	1,101E-03	907	CLDN1	2,1	3,757E-04
910	908	ISYNA1	5,1	3,163E-03	908	TBC1D2B	2,5	3,205E-05	908	ACADM	3,6	1,348E-03					908	FBXO6	2,6	1,711E-05	908	SLC38A6	2,1	1,210E-02
911	909	LIPG	5,1	9,704E-07	909	DLEU1	2,5	5,021E-03	909	DYNC2LI1	3,6	3,606E-05					909	MKNK2	2,6	6,820E-04	909	ID2	2,1	3,942E-02
912	910	NEDD1	5,1	1,127E-04	910	HSPB6	2,5	9,740E-05	910	CLN5	3,6	6,111E-06					910	SERPINE2	2,5	6,088E-04	910	PARP11	2,1	3,800E-03
913	911	IQCC	5,1	3,272E-04	911	ELL	2,5	3,387E-02	911	HLA-F-AS1	3,5	6,766E-03					911	BIRC3	2,5	6,799E-04	911	ZCCHC24	2,1	5,912E-04
914	912	NUSAP1	5,1	2,109E-04	912	B4GALT2	2,4	1,896E-04	912	EPB41L5	3,5	3,622E-03					912	MAFF	2,5	1,987E-04	912	ARL6IP1	2,1	2,624E-04
915	913	DNAJC22	5,1	1,192E-03	913	TBC1D2	2,4	1,136E-02	913	SMO	3,5	2,806E-05					913	ZFP36L1	2,5	1,660E-05	913	CDK20	2,1	3,292E-04
916	914	CLSPN	5,1	4,412E-06	914	TMEM51	2,4	1,163E-02	914	DRAM1	3,5	1,171E-05					914	PPM1K	2,5	3,741E-03	914	DYRK3	2,1	5,472E-05
917	915	PHF19	5,1	1,526E-05	915	DRAP1	2,4	2,053E-04	915	ZDHHC21	3,5	1,331E-04					915	FZD4	2,5	1,987E-06	915	PPP1CB	2,1	1,865E-04
918	916	TRANK1	5,1	4,190E-04	916	CDH11	2,4	1,423E-03	916	SUSD1	3,5	5,899E-04					916	SSSCA1	2,5	5,476E-03	916	GDAP1	2,1	4,317E-04
919	917	DUSP6	5,0	4,332E-04	917	ETHE1	2,4	3,034E-04	917	SYNGR1	3,5	7,426E-06					917	KIAA1462	2,5	2,968E-05	917	SRGAP1	2,1	2,454E-02
920	918	SPC25	5,0	1,955E-04	918	CAPZB	2,4	5,342E-03	918	HOXD13	3,5	4,923E-02					918	AGPAT2	2,5	4,278E-03	918	TTC26	2,1	1,727E-03
921	919	ANXA3	5,0	1,309E-04	919	KLF13	2,4	2,068E-06	919	KAZALD1	3,5	8,171E-03					919	CRLF1	2,5	1,186E-02	919	EPB41L5	2,1	5,084E-03
922	920	ARHGEF37	5,0	3,441E-03	920	CD68	2,4	6,615E-04	920	ADC	3,5	1,110E-03					920	TRIO	2,5	3,486E-04	920	NDUFA4	2,1	4,645E-06
923	921	HMGB3	5,0	1,001E-06	921	CASP4	2,4	9,012E-03	921	PDE5A	3,5	1,057E-04					921	ARHGEF17	2,5	2,862E-04	921	SLC25A14	2,1	4,515E-03
924	922	KLHL25	5,0	1,462E-03	922	EFR3A	2,4	8,642E-04	922	PAIP2	3,5	4,618E-05					922	HDAC7	2,5	1,647E-02	922	CRYL1	2,1	6,564E-04
925	923	ANKRD32	5,0	2,006E-03	923	MAP3K6	2,4	1,113E-03	923	AMN1	3,5	1,851E-03					923	SIX2	2,5	3,416E-02	923	MMD	2,1	2,179E-04
926	924	MTHFD2L	5,0	2,430E-03	924	MEF2A	2,4	1,182E-04	924	ETV5	3,5	3,095E-06					924	SNHG5	2,5	8,401E-03	924	ADD3	2,1	3,749E-04
927	925	GCLC	5,0	1,081E-04	925	S100A3	2,4	8,167E-04	925	MTMR9LP	3,5	1,881E-02					925	NCKIPSD	2,5	8,494E-04	925	ATP6V1E2	2,1	3,987E-03
928	926	USP6NL	5,0	1,973E-04	926	TSHZ1	2,4	3,707E-04	926	FIBIN	3,5	5,918E-05					926	SDF2L1	2,5	1,471E-02	926	NUCKS1	2,1	1,926E-04
929	927	GLCCI1	5,0	2,811E-03	927	ZNF395	2,4	1,157E-02	927	AMOT	3,5	2,343E-05					927	GPR39	2,5	1,479E-03	927	ITGA2	2,1	1,767E-03
930	928	PARP14	5,0	1,096E-05	928	G0S2	2,4	6,044E-03	928	CDC6	3,5	2,821E-05					928	TRIM25	2,5	1,099E-04	928	AK3	2,1	2,543E-04
931	929	OSBPL11	5,0	7,035E-05	929	CAPN1	2,4	2,825E-04	929	GNAO1	3,5	9,448E-05					929	FLOT1	2,5	3,820E-04	929	CCDC104	2,1	2,112E-05
932	930	DUSP18	5,0	1,930E-05	930	ATP6V1B1	2,4	1,090E-02	930	GTF2E1	3,5	5,558E-05					930	IL7	2,5	2,083E-02	930	EFCAB7	2,1	5,877E-04
933	931	DEK	5,0	1,057E-02	931	RAB42	2,4	5,956E-03	931	TP53INP1	3,5	1,162E-03					931	VSTM2L	2,5	1,386E-02	931	SELL	2,1	4,165E-02
934	932	EEF1E1	5,0	3,397E-03	932	RPS27L	2,4	2,379E-03	932	GLT8D2	3,5	1,821E-05					932	RRAS2	2,5	6,175E-04	932	SOX9	2,1	3,282E-04
935	933	MPDZ	5,0	1,053E-04	933	IL4I1	2,4	1,968E-03	933	FOPNL	3,5	1,698E-04					933	PSMB8	2,5	1,031E-06	933	HMGN3	2,1	3,968E-05
936	934	DENND5B	5,0	3,180E-04	934	PACS1	2,4	8,547E-06	934	GFM2	3,5	2,718E-05					934	EFEMP2	2,5	5,627E-03	934	PER2	2,1	1,443E-04
937	935	HLA-F-AS1	5,0	1,037E-03	935	CAP1	2,4	3,427E-05	935	MBP	3,5	1,109E-02					935	MT1X	2,5	3,794E-04	935	ZNF334	2,1	3,601E-03
938	936	ECT2	5,0	1,822E-04	936	SUN2	2,4	1,877E-04	936	SOWAHC	3,5	1,278E-03					936	NR1D1	2,5	3,043E-04	936	KIF1B	2,0	5,319E-04
939	937	GAS2L3	5,0	1,625E-04	937	LZTR1	2,4	2,436E-03	937	MAPK8IP1	3,5	1,612E-04					937	BYSL	2,5	3,145E-02	937	FAM46C	2,0	1,930E-03
940	938	USP2	5,0	2,782E-04	938	RCOR2	2,4	1,484E-02	938	MR1	3,5	3,464E-05					938	ZNF618	2,5	3,703E-05	938	JKAMP	2,0	6,897E-04
941	939	UCK1	5,0	9,540E-04	939	SNAPC2	2,4	9,578E-04	939	MAP1B	3,5	2,467E-05					939	EGR1	2,5	8,462E-04	939	NAGK	2,0	3,040E-03
942	940	SPAG16	4,9	1,168E-04	940	CFL1	2,4	1,684E-02	940	HFE	3,5	3,639E-05					940	OSBPL10	2,5	2,766E-03	940	COQ7	2,0	5,172E-03
943	941	URB1	4,9	7,630E-06	941	CCDC107	2,4	2,144E-03	941	CEP70	3,5	6,769E-05					941	TOX2	2,5	3,194E-04	941	KIDINS220	2,0	4,857E-04
944	942	GSG2	4,9	6,331E-05	942	MTFP1	2,4	1,345E-03	942	PSIP1	3,5	2,112E-06					942	GOPC	2,5	4,680E-05	942	COQ10A	2,0	2,510E-03
945	943	C19orf57	4,9	8,710E-04	943	CXXC5	2,4	5,585E-03	943	DIABLO	3,5	1,610E-02					943	RTN2	2,5	4,946E-02	943	SLC39A8	2,0	4,575E-03
946	944	CDC45	4,9	3,139E-06	944	NR1D1	2,4	1,423E-05	944	SGSH	3,5	1,700E-02					944	GPC1	2,5	5,593E-03	944	EZR	2,0	1,750E-04
947	945	ZNF311	4,9	1,173E-04	945	SRPX	2,4	1,768E-05	945	CAST	3,5	1,510E-05					945	B4GALT1	2,5	6,340E-06	945	NSL1	2,0	8,984E-04
948	946	ASPM	4,9	2,812E-04	946	ERGIC1	2,4	6,305E-06	946	NF2	3,5	5,785E-04					946	BCL9L	2,5	4,979E-03	946	ALG14	2,0	7,095E-03
949	947	NDC80	4,9	5,516E-05	947	GPX7	2,4	9,689E-04	947	TSPAN14	3,5	7,978E-05					947	NDST1	2,4	7,899E-04	947	FANCF	2,0	3,734E-04
950	948	FOXO1	4,9	8,239E-06	948	ACSL1	2,4	5,390E-06	948	SCARA3	3,5	3,246E-06					948	C1GALT1	2,4	1,146E-03	948	SMO	2,0	4,157E-04
951	949	DZIP3	4,9	2,232E-05	949	STOML1	2,4	6,193E-03	949	CDH24	3,5	3,301E-03					949	ZNFX1	2,4	1,942E-05	949	CD109	2,0	6,693E-04
952	950	SFMBT1	4,9	6,782E-05	950	CDK18	2,4	2,978E-03	950	STAU2	3,5	3,656E-06					950	RASSF7	2,4	2,845E-02	950	MFI2-AS1	2,0	1,772E-02
953	951	CAB39L	4,9	2,819E-03	951	NSMAF	2,4	6,776E-03	951	CHEK1	3,5	4,561E-05					951	ACVR1	2,4	1,112E-04	951	GPC6	2,0	5,401E-05
954	952	SLC4A4	4,9	2,516E-03	952	C14orf28	2,4	2,261E-02	952	HOXA7	3,4	9,608E-04					952	BCL2L11	2,4	9,090E-06	952	TMTC4	2,0	9,535E-04
955	953	IGSF3	4,9	1,566E-06	953	FAIM2	2,4	1,109E-03	953	EHBP1	3,4	4,832E-04					953	SLC29A4	2,4	4,775E-02	953	ITGAV	2,0	7,851E-04
956	954	PHTF1	4,9	2,605E-07	954	POLG	2,4	1,372E-02	954	CREBL2	3,4	4,629E-06					954	NAGS	2,4	2,283E-03	954	BTG3	2,0	5,339E-04
957	955	MMP7	4,9	7,122E-05	955	PLEC	2,4	4,390E-02	955	USP12	3,4	1,748E-06					955	CTSD	2,4	3,255E-04	955	C16orf46	2,0	1,790E-02
958	956	ZNF649	4,9	1,248E-03	956	TBX3	2,4	2,674E-02	956	RERE	3,4	2,764E-04					956	CHST11	2,4	1,496E-04	956	MRPS28	2,0	2,249E-03
959	957	SIAE	4,9	8,140E-06	957	DOK1	2,4	3,797E-04	957	ORAI2	3,4	8,920E-03					957	ZNF852	2,4	1,292E-02	957	IQGAP3	2,0	1,291E-05
960	958	C8orf46	4,9	3,102E-04	958	BCL9L	2,4	1,871E-03	958	PASK	3,4	9,701E-03					958	PDE4D	2,4	2,578E-04	958	RRM2B	2,0	2,391E-04
961	959	KAZALD1	4,9	5,276E-04	959	ASAP2	2,3	3,817E-04	959	PCTP	3,4	6,443E-05					959	ZDHHC8	2,4	2,141E-02	959	MCC	2,0	3,757E-03
962	960	GSTM3	4,9	3,710E-02	960	LGR4	2,3	3,449E-04	960	SLC25A33	3,4	4,689E-04					960	MIIP	2,4	4,057E-03	960	ARL6	2,0	7,223E-04
963	961	CSAD	4,9	5,700E-05	961	MGAT1	2,3	9,512E-04	961	DUSP6	3,4	1,693E-04					961	DBN1	2,4	2,143E-03	961	SEC23B	2,0	7,025E-06
964	962	DUSP16	4,9	1,288E-04	962	WNT5A	2,3	8,667E-05	962	AKIRIN1	3,4	7,491E-05					962	RND3	2,4	1,329E-04	962	FAM104B	2,0	2,601E-03
965	963	ZNF671	4,9	1,080E-04	963	ASPH	2,3	2,292E-04	963	B3GNT2	3,4	1,386E-05					963	IGF2BP2	2,4	2,635E-06	963	ACACB	2,0	1,812E-02
966	964	LZTFL1	4,9	6,623E-03	964	NUDT11	2,3	1,770E-03	964	FAM210B	3,4	4,302E-07					964	VASP	2,4	1,535E-03	964	APOO	2,0	5,145E-05
967	965	HMGN3	4,9	9,106E-06	965	SLC16A2	2,3	2,486E-02	965	NFKBIZ	3,4	3,813E-05					965	REPS2	2,4	1,148E-03	965	CADM3	2,0	2,953E-02
968	966	ENSA	4,8	1,184E-04	966	TICAM1	2,3	4,655E-03	966	CMTM3	3,4	2,073E-02					966	LEPREL4	2,4	1,723E-04	966	SLITRK5	2,0	2,695E-03
969	967	MELK	4,8	1,597E-05	967	PORCN	2,3	9,653E-05	967	LIN52	3,4	7,381E-06					967	LPPR3	2,4	2,867E-02	967	WDR35	2,0	1,588E-03
970	968	FANCG	4,8	7,426E-05	968	SPSB1	2,3	5,854E-05	968	DST	3,4	8,066E-05					968	ATP10A	2,4	4,055E-02	968	MAN1A1	2,0	1,641E-05
971	969	AMOT	4,8	1,411E-04	969	GABBR1	2,3	3,820E-03	969	CTTNBP2NL	3,4	8,308E-05					969	PRDX4	2,4	2,952E-03	969	TRIM45	2,0	1,830E-04
972	970	FANCA	4,8	7,451E-05	970	STEAP1	2,3	4,736E-04	970	ZNF436	3,4	6,880E-05					970	MAN2B2	2,4	4,385E-03	970	BRMS1L	2,0	3,786E-03
973	971	RAB39B	4,8	3,920E-06	971	GNG7	2,3	2,596E-03	971	ZNF75D	3,4	7,853E-04					971	CNPY3	2,4	9,655E-05				
974	972	MAN1C1	4,8	1,422E-05	972	SLCO2A1	2,3	6,474E-04	972	PPM1J	3,4	1,164E-02					972	FDXACB1	2,4	1,079E-03				
975	973	C21orf59	4,8	4,109E-04	973	TMEM44	2,3	3,943E-03	973	PARD6A	3,4	3,852E-03					973	LRFN4	2,4	4,776E-02				
976	974	NCAPD2	4,8	8,862E-04	974	GBE1	2,3	1,253E-05	974	SLC17A5	3,4	2,906E-05					974	GLIPR1	2,4	2,968E-03				
977	975	LCAT	4,8	1,106E-03	975	PRR15	2,3	4,468E-02	975	PFKFB3	3,4	4,833E-04					975	HSPA1A	2,4	2,603E-02				
978	976	PXMP2	4,8	1,728E-05	976	THAP8	2,3	4,771E-03	976	XRN1	3,4	2,689E-04					976	CHSY1	2,4	4,840E-04				
979	977	METTL18	4,8	3,795E-04	977	LYNX1	2,3	6,645E-04	977	IRS2	3,4	1,568E-05					977	RUNX1	2,4	1,311E-04				
980	978	TJP2	4,8	7,581E-06	978	USP35	2,3	5,088E-04	978	HELLS	3,4	1,119E-03					978	SCARF2	2,4	3,483E-02				
981	979	SMC2	4,8	1,584E-05	979	FAT1	2,3	3,310E-03	979	TMEM64	3,4	2,019E-05					979	STX3	2,4	4,075E-03				
982	980	CENPH	4,8	5,927E-04	980	CTTN	2,3	3,456E-03	980	GLRB	3,4	5,038E-04					980	LPAR1	2,4	2,441E-04				
983	981	KIF4A	4,8	3,246E-05	981	DAPK3	2,3	4,937E-05	981	NLK	3,4	5,537E-06					981	ZBTB42	2,4	1,254E-03				
984	982	GPT2	4,8	2,583E-04	982	TMEM102	2,3	1,378E-03	982	NDE1	3,4	8,742E-03					982	STARD10	2,4	1,409E-02				
985	983	C1orf74	4,8	8,079E-05	983	KCTD10	2,3	1,353E-04	983	APOL3	3,4	1,711E-03					983	GPR3	2,4	4,213E-04				
986	984	DTL	4,8	8,933E-08	984	ANXA11	2,3	2,776E-05	984	FUK	3,4	6,782E-04					984	EPHA2	2,4	1,039E-03				
987	985	ITGA2	4,8	3,047E-04	985	PABPC3	2,3	1,212E-04	985	ZMYM2	3,4	5,225E-04					985	RBPMS2	2,4	7,491E-04				
988	986	RP2	4,8	1,418E-04	986	PPDPF	2,3	2,348E-02	986	GNRH1	3,4	1,738E-03					986	ATAD3B	2,4	1,606E-02				
989	987	GPR137B	4,8	1,753E-04	987	RAI14	2,3	6,680E-04	987	EAF2	3,4	6,422E-03					987	RNF24	2,4	2,490E-03				
990	988	DICER1-AS1	4,8	7,800E-03	988	MMP24	2,3	1,787E-04	988	NSUN3	3,4	5,738E-04					988	ARPC1B	2,4	1,447E-03				
991	989	PSTPIP2	4,8	1,463E-04	989	ITGB1BP1	2,3	3,496E-02	989	MLEC	3,4	1,539E-02					989	MVP	2,4	3,891E-04				
992	990	DNAJC12	4,8	4,465E-04	990	SIPA1	2,3	2,850E-02	990	HTR7P1	3,3	9,575E-03					990	GNL3	2,3	1,112E-05				
993	991	SAP30	4,8	1,229E-04	991	METRNL	2,3	2,427E-02	991	FAM200B	3,3	3,637E-04					991	ARRDC3	2,3	1,058E-04				
994	992	MOV10L1	4,8	1,509E-02	992	MEF2D	2,3	9,763E-04	992	RANGRF	3,3	9,845E-04					992	LGR4	2,3	7,038E-04				

	A	B	C	D	E	F	G	H	I	J	K	L	M	N	O	P	Q	R	S	T	U	V	W	X
995	993	DPCD	4,8	8,540E-05	993	JMJD7	2,3	2,443E-04	993	CHAC2	3,3	2,784E-04					993	NFE2L3	2,3	4,282E-05				
996	994	CCDC15	4,7	4,278E-04	994	PLOD3	2,3	1,283E-04	994	WIPF2	3,3	3,469E-02					994	C19orf66	2,3	3,457E-03				
997	995	FAM122B	4,7	3,314E-05	995	CMTM7	2,3	1,377E-02	995	FST	3,3	4,554E-05					995	ANGPT1	2,3	2,833E-03				
998	996	AIFM2	4,7	6,474E-06	996	FAM58A	2,2	2,219E-03	996	ITGB8	3,3	4,086E-03					996	RAB3IL1	2,3	2,635E-02				
999	997	MCM9	4,7	2,836E-03	997	ACTB	2,2	3,236E-03	997	PPAPDC2	3,3	8,013E-05					997	PPIB	2,3	1,395E-06				
1000	998	NSL1	4,7	5,217E-05	998	OSBPL10	2,2	2,948E-03	998	CBX7	3,3	8,320E-05					998	KCNAB2	2,3	9,028E-03				
1001	999	FAM71E1	4,7	1,034E-02	999	IGFBP4	2,2	6,685E-04	999	PDE4A	3,3	2,435E-04					999	LAP3	2,3	7,473E-05				
1002	1000	CEP192	4,7	2,104E-04	1000	BET1L	2,2	8,709E-04	1000	FZD5	3,3	1,783E-05					1000	COL9A2	2,3	1,946E-02				
1003	1001	ZKSCAN3	4,7	9,529E-03	1001	LSM7	2,2	6,818E-03	1001	SS18	3,3	9,042E-05					1001	SCO2	2,3	7,247E-03				
1004	1002	SPATA6	4,7	4,910E-04	1002	C15orf61	2,2	9,435E-03	1002	ZMAT3	3,3	3,216E-05					1002	GNAS	2,3	2,449E-02				
1005	1003	CDT1	4,7	8,998E-03	1003	TRIB3	2,2	7,849E-05	1003	AQP11	3,3	4,086E-03					1003	COMT	2,3	1,580E-03				
1006	1004	TAF9B	4,7	5,396E-06	1004	BAMBI	2,2	1,264E-04	1004	TFDP2	3,3	1,242E-03					1004	PDGFA	2,3	2,291E-05				
1007	1005	CKS2	4,7	6,884E-05	1005	ZNF668	2,2	2,171E-02	1005	RAB20	3,3	1,186E-03					1005	PMEPA1	2,3	1,216E-02				
1008	1006	C2orf74	4,7	1,642E-03	1006	PPP1R18	2,2	9,332E-05	1006	ATAD5	3,3	2,035E-04					1006	PLCL2	2,3	1,924E-04				
1009	1007	BMP6	4,7	3,710E-04	1007	CALHM2	2,2	5,066E-03	1007	TTF2	3,3	7,115E-04					1007	SKIL	2,3	5,461E-03				
1010	1008	HELQ	4,7	1,430E-02	1008	RBMS1	2,2	3,735E-04	1008	ETV4	3,3	2,401E-03					1008	TNIP1	2,3	6,290E-04				
1011	1009	BCL9	4,7	1,371E-06	1009	DNMT3B	2,2	9,482E-03	1009	CLDND1	3,3	1,332E-03					1009	EMP2	2,3	1,214E-04				
1012	1010	TLR1	4,7	3,736E-04	1010	APBB3	2,2	2,110E-03	1010	MB21D1	3,3	4,812E-04					1010	PTPLA	2,3	7,804E-03				
1013	1011	TRAF3IP2-AS1	4,7	3,709E-03	1011	WIPF1	2,2	9,865E-04	1011	TMEM106C	3,3	4,678E-04					1011	ELF1	2,3	1,447E-04				
1014	1012	LETMD1	4,7	6,694E-05	1012	YIPF2	2,2	4,463E-02	1012	CAT	3,3	6,485E-06					1012	PLAGL1	2,3	3,415E-06				
1015	1013	HJURP	4,7	3,638E-06	1013	SMTN	2,2	1,410E-03	1013	ARHGEF12	3,3	5,548E-05					1013	SRC	2,3	6,336E-05				
1016	1014	FAM117A	4,7	3,795E-05	1014	TNFAIP8L1	2,2	2,871E-03	1014	ECT2	3,3	3,061E-03					1014	ARHGAP24	2,3	1,319E-03				
1017	1015	C21orf119	4,7	5,272E-03	1015	CYB5R3	2,2	3,282E-04	1015	PLEKHA5	3,3	1,983E-04					1015	TIAM2	2,3	2,215E-03				
1018	1016	RPS6KA6	4,7	5,181E-03	1016	KDM4B	2,2	1,838E-03	1016	KBTBD6	3,3	5,926E-04					1016	PSMD5	2,3	2,126E-06				
1019	1017	GPR161	4,6	2,422E-06	1017	PITX1	2,2	3,431E-02	1017	ULBP3	3,3	6,400E-03					1017	ATOH8	2,3	4,846E-04				
1020	1018	IRAK1BP1	4,6	2,988E-04	1018	TRIM3	2,2	1,686E-02	1018	FAM135A	3,3	1,366E-03					1018	SLC9A7	2,3	1,311E-02				
1021	1019	COPG2	4,6	3,535E-02	1019	HOXD-AS1	2,2	1,397E-03	1019	TMEM164	3,3	4,986E-04					1019	EFHD2	2,3	3,211E-03				
1022	1020	GAS8	4,6	2,472E-05	1020	RNH1	2,2	3,622E-03	1020	LZTS1	3,3	6,134E-06					1020	C11orf95	2,3	3,066E-04				
1023	1021	TTC26	4,6	6,482E-06	1021	CD276	2,2	7,243E-03	1021	ARNTL2	3,3	8,601E-05					1021	C9orf89	2,3	3,194E-03				
1024	1022	USP37	4,6	7,495E-05	1022	CHPF	2,2	3,478E-02	1022	DHX40	3,3	1,820E-03					1022	GPR146	2,3	1,529E-03				
1025	1023	RTKN2	4,6	1,095E-05	1023	MYD88	2,2	1,250E-02	1023	SLC35A5	3,3	9,880E-06					1023	MARCH3	2,3	9,688E-04				
1026	1024	L3MBTL1	4,6	4,407E-05	1024	CDK5R1	2,2	2,511E-03	1024	MTHFD2L	3,3	4,034E-03					1024	MGAT4B	2,3	2,983E-02				
1027	1025	CTSK	4,6	5,110E-04	1025	FHL3	2,2	1,335E-03	1025	MSRA	3,3	3,514E-04					1025	HSPG2	2,3	1,715E-03				
1028	1026	JAM3	4,6	4,349E-04	1026	SRM	2,2	1,258E-03	1026	RAB9B	3,3	1,479E-03					1026	ARL5B	2,3	3,001E-04				
1029	1027	SGOL1	4,6	2,394E-03	1027	GRK5	2,2	1,135E-05	1027	PPT1	3,3	1,447E-03					1027	PLS1	2,3	2,229E-02				
1030	1028	KRBA2	4,6	7,554E-03	1028	LIME1	2,2	5,097E-03	1028	DIP2B	3,3	9,900E-08					1028	ARID1B	2,3	2,121E-04				
1031	1029	HDHD3	4,6	1,501E-05	1029	SRPX2	2,2	1,368E-08	1029	EFHC1	3,3	2,807E-04					1029	FAM110B	2,3	4,431E-04				
1032	1030	TP53INP1	4,6	1,776E-04	1030	CCDC125	2,2	2,045E-03	1030	CDKN2B	3,3	3,574E-05					1030	PTPRE	2,3	3,665E-03				
1033	1031	BDH1	4,6	1,591E-03	1031	BLVRB	2,2	2,401E-03	1031	EGR1	3,3	6,791E-04					1031	MYL9	2,3	1,412E-04				
1034	1032	CDKAL1	4,6	8,234E-05	1032	PKN1	2,2	1,201E-03	1032	CRYL1	3,3	9,730E-05					1032	SCARB2	2,3	1,681E-03				
1035	1033	TMEM25	4,6	3,659E-03	1033	SEZ6L2	2,2	6,201E-03	1033	SLC29A4	3,3	4,701E-03					1033	FHOD1	2,3	1,699E-03				
1036	1034	BST2	4,6	3,455E-03	1034	RALB	2,2	3,468E-02	1034	ARL5B	3,3	3,457E-06					1034	HSPB3	2,3	6,645E-03				
1037	1035	L2HGDH	4,6	1,167E-04	1035	AGMAT	2,2	1,087E-02	1035	XPO6	3,3	1,046E-04					1035	ICAM5	2,3	4,247E-02				
1038	1036	LRRC2	4,6	3,510E-05	1036	RHOG	2,2	1,371E-03	1036	NCBP2	3,3	3,477E-03					1036	C14orf159	2,3	4,749E-04				
1039	1037	CCBL1	4,6	3,397E-04	1037	C20orf27	2,2	1,225E-02	1037	HMBOX1	3,2	8,950E-04					1037	BAG2	2,3	9,801E-05				
1040	1038	SAT1	4,6	1,028E-05	1038	BCAT1	2,2	2,285E-04	1038	RPS6KA1	3,2	1,012E-04					1038	IFNGR2	2,3	1,999E-03				
1041	1039	ZNF606	4,6	1,679E-02	1039	PAX6	2,2	1,746E-02	1039	PLA2R1	3,2	1,766E-04					1039	BOK	2,3	5,082E-04				
1042	1040	IQCB1	4,6	8,741E-06	1040	BMP1	2,1	1,447E-03	1040	MON1B	3,2	1,940E-05					1040	IGSF8	2,3	3,258E-03				
1043	1041	ELOVL6	4,6	6,269E-03	1041	SLC4A7	2,1	8,649E-04	1041	DNAJC3-AS1	3,2	2,704E-04					1041	ERO1L	2,3	3,487E-04				
1044	1042	RPA2	4,6	1,899E-04	1042	RPL23	2,1	3,576E-03	1042	TLE3	3,2	1,028E-04					1042	MAMLD1	2,3	8,156E-03				
1045	1043	PARP1	4,6	7,942E-07	1043	BMPR2	2,1	1,201E-04	1043	FOXRED2	3,2	1,446E-04					1043	SLC38A10	2,3	7,638E-03				
1046	1044	C18orf25	4,6	2,790E-05	1044	MOCOS	2,1	5,817E-03	1044	RUVBL1	3,2	2,281E-05					1044	ROBO3	2,3	2,380E-02				
1047	1045	KNTC1	4,6	6,400E-05	1045	ZHX3	2,1	1,103E-03	1045	NIPAL3	3,2	2,258E-04					1045	UGDH	2,3	2,889E-04				
1048	1046	TTC21A	4,6	6,534E-03	1046	MIIP	2,1	3,930E-03	1046	TLR3	3,2	9,146E-04					1046	MTFP1	2,2	3,124E-03				
1049	1047	GTF3C4	4,6	1,219E-05	1047	PVR	2,1	2,582E-02	1047	RCAN2	3,2	7,453E-05					1047	RAB3B	2,2	7,074E-04				
1050	1048	TMEM198B	4,6	9,905E-05	1048	CACNA1C	2,1	1,363E-03	1048	ME3	3,2	3,894E-04					1048	FKBP11	2,2	4,464E-03				
1051	1049	CENPM	4,6	1,623E-03	1049	ARL4C	2,1	1,560E-05	1049	HOMER2	3,2	1,191E-03					1049	DTX3L	2,2	1,278E-05				
1052	1050	KIF11	4,6	6,112E-05	1050	MEIS1	2,1	1,608E-02	1050	GBP2	3,2	2,310E-04					1050	SNX9	2,2	2,748E-06				
1053	1051	ZNF789	4,5	1,218E-03	1051	SERPINE2	2,1	1,904E-03	1051	MACF1	3,2	1,517E-03					1051	C3orf18	2,2	5,849E-03				
1054	1052	CDK5RAP2	4,5	1,176E-03	1052	PRKCD	2,1	2,231E-04	1052	C8orf37	3,2	1,145E-03					1052	FHL2	2,2	1,333E-04				
1055	1053	BRIP1	4,5	1,878E-04	1053	FAM160B2	2,1	2,617E-03	1053	KIAA1432	3,2	5,922E-05					1053	ELF4	2,2	4,736E-05				
1056	1054	SETBP1	4,5	1,029E-04	1054	CCDC149	2,1	2,366E-02	1054	TMEM130	3,2	1,273E-02					1054	TEL1-TNFRSF6	2,2	1,954E-02				
1057	1055	GMNN	4,5	1,057E-02	1055	ATXN3	2,1	2,657E-03	1055	PPP1CC	3,2	2,564E-04					1055	SUGT1	2,2	6,695E-04				
1058	1056	BBS12	4,5	8,289E-04	1056	LIMCH1	2,1	5,446E-03	1056	SUZ12	3,2	7,502E-05					1056	PPP3CA	2,2	5,576E-04				
1059	1057	LRRC29	4,5	1,649E-02	1057	RASSF7	2,1	4,484E-02	1057	ADPGK	3,2	1,451E-02					1057	NCDN	2,2	9,585E-03				
1060	1058	KIAA1467	4,5	4,032E-05	1058	RAB2A	2,1	1,008E-04	1058	CDK14	3,2	2,719E-05					1058	PHLDA3	2,2	1,037E-04				
1061	1059	TROAP	4,5	1,984E-05	1059	DVL1	2,1	1,830E-02	1059	KDM4D	3,2	1,058E-03					1059	NFE2	2,2	3,058E-02				
1062	1060	FBXL19-AS1	4,5	2,347E-03	1060	TEAD3	2,1	7,507E-05	1060	ARL6IP1	3,2	9,761E-06					1060	DNAJB5	2,2	1,990E-02				
1063	1061	H2AFJ	4,5	1,980E-03	1061	CORO1B	2,1	2,989E-03	1061	TMEM39A	3,2	1,837E-03					1061	HSD3B7	2,2	6,458E-04				
1064	1062	CAHM	4,5	3,656E-03	1062	GLIPR1	2,1	5,575E-04	1062	FNDC4	3,2	1,848E-04					1062	NDEL1	2,2	2,037E-04				
1065	1063	FER	4,5	9,839E-03	1063	ARHGAP23	2,1	1,357E-03	1063	CPT1C	3,2	3,383E-04					1063	MANF	2,2	1,757E-03				
1066	1064	SAMD9	4,5	7,875E-03	1064	KIF7	2,1	2,337E-02	1064	DTX3L	3,2	4,642E-06					1064	NOP14-AS1	2,2	8,368E-03				
1067	1065	FAM109A	4,5	1,894E-03	1065	LIMK1	2,1	2,002E-03	1065	QPRT	3,2	5,878E-03					1065	ST3GAL4	2,2	3,604E-04				
1068	1066	MEF2C	4,5	1,766E-03	1066	MAPKAPK3	2,1	4,447E-04	1066	TMX1	3,2	1,468E-05					1066	PRR5L	2,2	1,769E-02				
1069	1067	IFT46	4,5	1,252E-04	1067	FKBP2	2,1	9,072E-04	1067	PARP11	3,2	9,245E-04					1067	FKBP10	2,2	3,695E-03				
1070	1068	HIST1H2BD	4,5	5,302E-04	1068	SLC9A7	2,1	1,435E-03	1068	NAA38	3,1	1,258E-04					1068	ZNF358	2,2	2,752E-02				
1071	1069	DIABLO	4,5	2,980E-02	1069	RASA3	2,1	2,855E-05	1069	MICB	3,1	6,074E-03					1069	CAPG	2,2	1,139E-03				
1072	1070	GPR56	4,5	9,172E-05	1070	C1GALT1	2,1	6,711E-03	1070	NCAPD2	3,1	1,515E-08					1070	RAB33A	2,2	6,123E-03				
1073	1071	HIST1H2BK	4,5	3,995E-04	1071	CDC42EP3	2,1	2,712E-04	1071	FBXO21	3,1	1,739E-04					1071	IRF9	2,2	1,813E-04				
1074	1072	C16orf59	4,5	3,957E-03	1072	FAM195B	2,1	8,613E-03	1072	STXBP4	3,1	7,173E-03					1072	PTPRK	2,2	2,109E-04				
1075	1073	TMEM209	4,5	9,401E-05	1073	MXD4	2,1	2,607E-03	1073	CDC42EP4	3,1	2,969E-05					1073	SLC20A1	2,2	1,200E-05				
1076	1074	FAM46C	4,5	4,602E-04	1074	PSMD5	2,1	1,765E-06	1074	PHLPP2	3,1	3,070E-02					1074	DCAF4	2,2	8,649E-04				
1077	1075	ORC1	4,4	2,596E-05	1075	PTPRB	2,1	6,740E-03	1075	PPP2R3A	3,1	2,451E-05					1075	SBNO2	2,2	1,197E-02				
1078	1076	STXBP4	4,4	1,970E-03	1076	C11orf95	2,1	1,462E-03	1076	PMEPA1	3,1	7,943E-08					1076	MFSD10	2,2	1,536E-02				
1079	1077	FAM66C	4,4	1,790E-05	1077	PICALM	2,1	2,215E-05	1077	AGPS	3,1	1,213E-06					1077	HMGCR	2,2	1,479E-04				
1080	1078	ITGB3BP	4,4	8,168E-05	1078	RAB34	2,1	1,029E-04	1078	RSRC1	3,1	1,721E-04					1078	THBS1	2,2	1,811E-06				
1081	1079	WDR54	4,4	1,735E-06	1079	PDE8A	2,1	6,151E-04	1079	EYA4	3,1	2,522E-02					1079	PDIA5	2,2	1,575E-05				
1082	1080	ADD3	4,4	4,731E-05	1080	TSPAN17	2,1	7,778E-03	1080	CDC45	3,1	1,621E-04					1080	SYT1	2,2	3,425E-04				
1083	1081	RBBP8	4,4	5,876E-04	1081	CCND1	2,1	4,253E-04	1081	PFKM	3,1	1,151E-05					1081	LDLR	2,2	2,145E-03				
1084	1082	POLE4	4,4	6,957E-03	1082	RELL2	2,1	1,213E-02	1082	IST1	3,1	3,811E-04					1082	EPS8L1	2,2	1,538E-02				
1085	1083	DDX60L	4,4	2,841E-03	1083	LPCAT4	2,1	5,986E-03	1083	FBXO36	3,1	3,118E-04					1083	BAMBI	2,2	2,390E-04				
1086	1084	VSIG10	4,4	6,595E-04	1084	TGFB3	2,1	4,004E-03	1084	SELT	3,1	4,353E-06					1084	PHF11	2,2	3,435E-02				
1087	1085	KIAA1841	4,4	1,528E-02	1085	LIN7B	2,1	2,918E-02	1085	ZNF48	3,1	1,914E-05					1085	GLYCTK	2,2	7,411E-03				
1088	1086	FBXO5	4,4	4,383E-06	1086	AEBP1	2,1	4,944E-02	1086	TPMT	3,1	2,396E-05					1086	SLC4A7	2,2	9,508E-04				
1089	1087	TTTY15	4,4	1,392E-02	1087	FMNL3	2,1	6,041E-03	1087	LEPREL1	3,1	9,297E-05					1087	TDRD7	2,2	1,300E-05				
1090	1088	ZNF420	4,4	8,180E-04	1088	BIN1	2,1	1,071E-03	1088	SH3BGRL2	3,1	1,997E-04					1088	SEC24A	2,2	2,603E-05				
1091	1089	TTK	4,4	9,293E-05	1089	ARHGEF1	2,1	4,893E-03	1089	ORC1	3,1	3,221E-04					1089	KCND1	2,2	1,948E-04				
1092	1090	CCNE1	4,4	9,330E-06	1090	PTPRJ	2,1	4,559E-04	1090	ITFG1	3,1	1,887E-07					1090	ROR1	2,2	7,739E-04				
1093	1091	SDCBP	4,4	2,486E-04	1091	SFXN3	2,1	1,098E-06	1091	ARHGAP17	3,1	1,722E-02					1091	ATP5J2	2,2	2,069E-04				
1094	1092	ASF1A	4,4	2,225E-06	1092	CLN8	2,1	6,724E-03	1092	CMTM4	3,1	3,307E-03					1092	CD200	2,2	3,678E-03				
1095	1093	SENP1	4,4	1,341E-05	1093	ZFPL1	2,1	1,102E-03	1093	CDCA3	3,1	1,314E-03					1093	TMSB10	2,2	7,936E-06				
1096	1094	ERBB2	4,4	1,600E-05	1094	SMYD3	2,1	7,220E-04	1094	SPEF2	3,1	3,593E-03					1094	P2RX6	2,2	1,213E-02				
1097	1095	DYRK3	4,4	2,758E-05	1095	HSF2BP	2,1	4,023E-02	1095	COQ10A	3,1	8,799E-04					1095	PLOD1	2,2	4,746E-04				
1098	1096	UHMK1	4,4	8,695E-04	1096	GADD45GIP1	2,1	5,782E-03	1096	EML1	3,1	3,721E-03					1096	BCL3	2,2	1,315E-02				
1099	1097	BUB1	4,4	1,092E-04	1097	ACTR3	2,1	3,279E-04	1097	SYNRG	3,1	1,124E-03					1097	LMAN2	2,2	2,600E-03				
1100	1098	UBE2H	4,4	1,163E-06	1098	INSIG1	2,1	3,357E-03	1098	GALNT7	3,1	2,361E-06					1098	LMO7	2,2	4,967E-04				
1101	1099	MKI67	4,4	7,292E-03	1099	FTH1	2,1	1,222E-02	1099	RNF182	3,1	4,220E-03					1099	ZC3HAV1	2,2	4,925E-05				
1102	1100	CDKN2C	4,4	6,980E-05	1100	SLC9A1	2,1	1,783E-04	1100	PTPN1	3,1	1,092E-07					1100	CCRN4L	2,2	5,986E-03				
1103	1101	TXNL4B	4,4	8,825E-04	1101	TIMP2	2,1	1,002E-02	1101	SPA17	3,1	6,398E-04					1101	LMF2	2,2	4,489E-02				
1104	1102	CDCA8	4,4	9,861E-07	1102	PRDX4	2,1	2,565E-05	1102	BCL2L13	3,1	1,045E-07					1102	ADORA2B	2,2	1,108E-03				
1105	1103	KIAA1598	4,4	3,516E-06	1103	RPS6KA3	2,1	1,101E-03	1103	IFNGR2	3,1	1,328E-03					1103	BASP1	2,2	7,832E-05				
1106	1104	SMC5	4,4	1,760E-04	1104	DZIP1	2,1	3,683E-03	1104	LYN	3,1	6,241E-04					1104	NCKAP5L	2,2	1,094E-02				
1107	1105	ERI2	4,4	5,619E-03	1105	MLLT11	2,1	5,194E-05	1105	TRIM14	3,1	1,349E-04					1105	SEC24D	2,2	2,090E-06				
1108	1106	AKNA	4,4	3,939E-03	1106	KDM6B	2,0	2,390E-02	1106	DZANK1	3,1	5,863E-04					1106	RXRA	2,2	2,227E-03				
1109	1107	RUVBL1	4,4	1,863E-05	1107	SYNPO	2,0	8,518E-04	1107	GPR56	3,1	2,504E-04					1107	C15orf61	2,2	5,848E-03				
1110	1108	MCM6	4,3	1,211E-05	1108	RAB20	2,0	1,792E-02	1108	AEBP2	3,1	6,360E-05					1108	SNX33	2,2	2,496E-04				
1111	1109	RSPH3	4,3	2,886E-02	1109	ANKRD37	2,0	3,357E-03	1109	GINS3	3,1	1,682E-04					1109	LHFPL2	2,2	2,511E-04				
1112	1110	SGCE	4,3	1,828E-05	1110	KCNQ5	2,0	3,275E-02	1110	DCAF16	3,1	1,128E-03					1110	ANKLE1	2,1	1,940E-03				
1113	1111	FAM83D	4,3	4,725E-06	1111	THY1	2,0	6,782E-06	1111	AHCYL2	3,1	2,542E-03					1111	TGFBR1	2,1	3,294E-04				
1114	1112	RHOF	4,3	3,127E-02	1112	BTBD2	2,0	1,874E-02	1112	FBXL19-AS1	3,1	1,849E-02					1112	PDIA6	2,1	6,736E-06				
1115	1113	CSPP1	4,3	1,950E-05	1113	ABLIM3	2,0	1,862E-04	1113	CHD9	3,1	2,911E-02					1113	UACA	2,1	4,694E-04				
1116	1114	TNFSF10	4,3	9,532E-03	1114	SMARCC1	2,0	1,090E-05	1114	LY6G5C	3,1	3,396E-02					1114	TBC1D22B	2,1	1,009E-04				
1117	1115	MSRA	4,3	2,510E-03	1115	GOLGA4	2,0	3,504E-02	1115	PISD	3,1	2,739E-03					1115	GLI3	2,1	1,847E-05				
1118	1116	ZNF347	4,3	1,183E-04	1116	MIR22HG	2,0	3,267E-03	1116	DHCR24	3,1	3,519E-06					1116	SLC12A9	2,1	1,012E-02				
1119	1117	ANP32E	4,3	3,074E-04	1117	RPLP2	2,0	1,516E-03	1117	IL18BP	3,1	1,348E-02					1117	ST5	2,1	2,345E-03				
1120	1118	CDKN1B	4,3	3,194E-06	1118	COMMD7	2,0	1,489E-04	1118	ITPR1	3,1	2,858E-03					1118	HIF1A	2,1	1,851E-05				
1121	1119	DPF1	4,3	5,105E-04	1119	GIPC1	2,0	3,502E-03	1119	TMEM108	3,1	7,841E-03					1119	SAPCD2	2,1	1,222E-02				
1122	1120	CXCL16	4,3	1,416E-04	1120	AVPI1	2,0	1,361E-02	1120	ABCB9	3,1	8,019E-03					1120	C6orf48	2,1	5,180E-06				
1123	1121	SORT1	4,3	8,234E-06	1121	TOP1MT	2,0	1,820E-03	1121	B3GALNT1	3,1	4,523E-04					1121	CD2AP	2,1	1,082E-04				
1124	1122	RANBP10	4,3	2,609E-05	1122	COL10A1	2,0	6,550E-04	1122	PARD3	3,1	1,987E-03					1122	ME3	2,1	5,164E-04				
1125	1123	EIF1AY	4,3	3,007E-04	1123	KANK2	2,0	2,121E-04	1123	PPAP2C	3,1	9,049E-04					1123	C6orf120	2,1	1,016E-04				
1126	1124	SPTAN1	4,3	6,605E-06	1124	UBE2E2	2,0	4,784E-03	1124	IPO8	3,1	2,293E-05					1124	PPAP2C	2,1	4,107E-04				
1127	1125	ZNF605	4,3	4,457E-04	1125	PNPLA2	2,0	1,426E-02	1125	PYROXD2	3,1	2,454E-02					1125	PRKY	2,1	1,704E-03				
1128	1126	SETD8	4,3	7,272E-03	1126	RIN3	2,0	8,892E-03	1126	TNRC6B	3,0	1,897E-02					1126	CCND1	2,1	5,366E-05				
1129	1127	KIF20B	4,3	6,631E-03	1127	GMPPA	2,0	1,519E-04	1127	FAM104A	3,0	4,261E-06					1127	NFIL3	2,1	2,899E-04				
1130	1128	KIF15	4,3	3,611E-05	1128	DSEL	2,0	6,395E-04	1128	RRN3P3	3,0	2,809E-02					1128	CREBZF	2,1	6,682E-04				
1131	1129	ZNF184	4,3	1,184E-03	1129	EEF2	2,0	3,373E-04	1129	ARHGAP29	3,0	2,464E-06					1129	ICK	2,1	3,316E-05				
1132	1130	ZNF117	4,3	4,191E-04	1130	FAM114A1	2,0	1,099E-05	1130	OTUD3	3,0	1,367E-02					1130	PPDPF	2,1	3,222E-02				
1133	1131	SNX27	4,3	2,545E-04	1131	PFN1	2,0	1,525E-04	1131	CCDC134	3,0	1,216E-03					1131	CXCL16	2,1	3,903E-04				
1134	1132	PBK	4,3	1,152E-03	1132	TCF3	2,0	2,695E-02	1132	LTBP4	3,0	6,642E-03					1132	SLC22A18	2,1	1,400E-02				
1135	1133	DCLRE1A	4,3	1,027E-04	1133	MED16	2,0	1,490E-02	1133	NIPSNAP1	3,0	6,611E-05					1133	NR2F2	2,1	3,495E-03				
1136	1134	LEPREL1	4,3	1,386E-04	1134	LAMP1	2,0	2,500E-03	1134	G2E3	3,0	1,598E-03					1134	DVL1	2,1	1,191E-02				
1137	1135	SRBD1	4,3	9,309E-05	1135	ZNF251	2,0	1,139E-03	1135	GIMAP2	3,0	9,541E-03					1135	LRRC1	2,1	1,514E-05				
1138	1136	FLVCR1-AS1	4,3	6,021E-04	1136	PLXNA3	2,0	3,178E-03	1136	ZNF396	3,0	3,070E-03					1136	TGFB1I1	2,1	2,035E-03				
1139	1137	CCDC18	4,3	1,037E-02	1137	ZGPAT	2,0	3,434E-02	1137	ARHGAP27	3,0	6,050E-04					1137	HSPA1B	2,1	5,867E-03				
1140	1138	CDK17	4,3	9,208E-04	1138	RPL28	2,0	3,590E-03	1138	CDKN1B	3,0	2,032E-05					1138	CRTAP	2,1	2,371E-02				
1141	1139	LINC00476	4,3	7,158E-04	1139	TTLL12	2,0	6,545E-03	1139	LCA5	3,0	7,711E-04					1139	TULP4	2,1	4,011E-03				
1142	1140	SEPHS1	4,3	5,628E-06	1140	SIX5	2,0	3,562E-02	1140	FKBP1B	3,0	7,464E-03					1140	SLC38A2	2,1	4,954E-03				
1143	1141	MASTL	4,2	1,490E-04	1141	CLTB	2,0	3,237E-02	1141	ZNF747	3,0	3,482E-03					1141	SCD	2,1	1,260E-04				
1144	1142	MAPRE3	4,2	1,996E-06	1142	CUX1	2,0	1,015E-03	1142	NUDT21	3,0	1,975E-05					1142	SH3D19	2,1	6,483E-04				
1145	1143	TRIM24	4,2	1,575E-04	1143	BNIP3	2,0	1,333E-04	1143	PALB2	3,0	4,634E-05					1143	B4GALNT1	2,1	4,211E-03				
1146	1144	IFT140	4,2	3,166E-05	1144	SERTAD1	2,0	1,860E-03	1144	RAC3	3,0	1,030E-02					1144	PLK2	2,1	1,022E-04				
1147	1145	EFNB2	4,2	1,181E-07	1145	NUCB1	2,0	2,259E-03	1145	PTBP2	3,0	3,029E-03					1145	PARP14	2,1	7,637E-06				
1148	1146	NPNT	4,2	3,149E-03					1146	ATXN1L	3,0	5,691E-05					1146	ARHGEF5	2,1	2,738E-03				
1149	1147	LRRC49	4,2	5,842E-04					1147	ZNF385A	3,0	5,179E-04					1147	RHOG	2,1	2,210E-03				
1150	1148	CREBL2	4,2	2,814E-06					1148	LCOR	3,0	2,464E-03					1148	NSMAF	2,1	2,021E-02				
1151	1149	BORA	4,2	8,769E-04					1149	OCIAD2	3,0	6,493E-03					1149	SPRY4	2,1	3,289E-04				
1152	1150	ZIK1	4,2	7,179E-05					1150	RRM2	3,0	1,183E-03					1150	DNMT3B	2,1	4,209E-03				
1153	1151	RNF38	4,2	2,411E-05					1151	RAD54L	3,0	2,091E-04					1151	PLCB3	2,1	2,024E-04				
1154	1152	CENPK	4,2	2,867E-03					1152	TBC1D9	3,0	2,106E-05					1152	AIM1	2,1	7,580E-05				
1155	1153	HMMR	4,2	3,085E-04					1153	TTC14	3,0	2,008E-03					1153	NFKB1	2,1	3,207E-05				
1156	1154	TMEM170A	4,2	2,084E-03					1154	B4GALT4	3,0	5,136E-04					1154	SMOX	2,1	2,870E-03				
1157	1155	GXYLT1	4,2	1,073E-04					1155	RBBP4	3,0	1,264E-02					1155	CDR2	2,1	2,644E-04				
1158	1156	PFN4	4,2	5,718E-05					1156	LRRC49	3,0	1,410E-03					1156	EDEM1	2,1	7,892E-06				
1159	1157	CENPE	4,2	5,656E-03					1157	RARB	3,0	2,642E-03					1157	INTS1	2,1	4,941E-02				
1160	1158	PPL	4,2	2,004E-03					1158	VPS13D	3,0	6,387E-03					1158	SYTL2	2,1	2,094E-03				
1161	1159	PHOSPHO2	4,2	3,883E-03					1159	SGCE	3,0	1,059E-04					1159	PANX1	2,1	3,098E-05				
1162	1160	SOCS3	4,2	8,685E-05					1160	FBXO33	3,0	6,853E-05					1160	RAMP2	2,1	1,504E-02				
1163	1161	SCARA3	4,2	2,949E-05					1161	ITM2C	3,0	1,538E-05					1161	ZNF354A	2,1	8,687E-05				
1164	1162	ZKSCAN2	4,2	3,772E-05					1162	LY96	3,0	2,495E-04					1162	GPR180	2,1	2,361E-03				
1165	1163	LIN52	4,2	2,914E-04					1163	NEK8	3,0	1,313E-03					1163	PRKCE	2,1	1,898E-03				
1166	1164	SLC2A12	4,2	1,253E-07					1164	CENPN	3,0	2,786E-06					1164	SLC39A13	2,1	6,984E-03				
1167	1165	ZNF664	4,2	4,961E-05					1165	SPRYD4	3,0	6,358E-05					1165	SNRK	2,1	9,064E-03				
1168	1166	RPAIN	4,2	9,744E-05					1166	WDR19	3,0	1,069E-03					1166	FLT3LG	2,1	5,383E-03				
1169	1167	CIT	4,2	1,629E-04					1167	SH3PXD2B	3,0	5,133E-05					1167	DOCK9	2,1	5,350E-03				
1170	1168	UHRF2	4,2	4,362E-07					1168	AGL	3,0	1,802E-05					1168	TSPYL2	2,1	4,270E-04				
1171	1169	C9orf37	4,2	2,592E-03					1169	MCC	3,0	5,363E-03					1169	ZNF620	2,0	2,436E-02				
1172	1170	PPP1R12B	4,2	8,047E-04					1170	TGOLN2	3,0	4,203E-06					1170	EDIL3	2,0	1,929E-04				
1173	1171	ZBTB24	4,1	1,675E-05					1171	MCM9	3,0	6,132E-03					1171	CLK3	2,0	3,977E-04				
1174	1172	PALB2	4,1	1,490E-04					1172	C17orf80	3,0	1,303E-03					1172	ATXN3	2,0	4,350E-02				
1175	1173	ARL8A	4,1	8,798E-06					1173	ZNF92	3,0	8,716E-04					1173	RDH10	2,0	3,603E-05				
1176	1174	PIGM	4,1	9,616E-06					1174	PERP	3,0	1,793E-05					1174	C19orf24	2,0	3,363E-02				
1177	1175	CLCN2	4,1	1,621E-05					1175	MICAL3	3,0	1,103E-03					1175	SLC22A4	2,0	4,695E-03				
1178	1176	DPYSL2	4,1	6,080E-06					1176	RNF168	3,0	1,144E-04					1176	RGMB	2,0	1,779E-04				
1179	1177	LIN9	4,1	6,729E-03					1177	RPS6KA2	3,0	1,421E-06					1177	TAF4B	2,0	6,257E-04				
1180	1178	FAM117B	4,1	1,353E-02					1178	C17orf97	3,0	5,239E-03					1178	PIEZO1	2,0	6,052E-03				
1181	1179	TOB1	4,1	2,421E-02					1179	CETN3	3,0	7,899E-03					1179	ITGA3	2,0	1,474E-04				
1182	1180	KIAA1009	4,1	1,045E-03					1180	CARD16	3,0	1,247E-02					1180	PVR	2,0	2,967E-02				
1183	1181	USP21	4,1	2,149E-04					1181	DCXR	3,0	8,353E-03					1181	C6orf106	2,0	3,007E-06				
1184	1182	WRB	4,1	1,637E-04					1182	SDC4	3,0	4,510E-06					1182	FGFR1OP	2,0	1,670E-04				
1185	1183	PPP1R26	4,1	1,119E-03					1183	MPDZ	3,0	7,233E-05					1183	OXSR1	2,0	1,273E-04				
1186	1184	TLCD1	4,1	9,288E-05					1184	SIAE	3,0	1,043E-04					1184	RBCK1	2,0	1,481E-03				
1187	1185	USP33	4,1	6,844E-05					1185	IFI35	3,0	1,850E-03					1185	SIPA1	2,0	1,214E-02				
1188	1186	WDR47	4,1	7,076E-06					1186	MAN2A1	3,0	5,335E-06					1186	SATB1	2,0	4,555E-03				
1189	1187	AFP	4,1	1,234E-03					1187	MID1	3,0	1,902E-04					1187	ITGA4	2,0	8,209E-03				
1190	1188	NIPSNAP3A	4,1	1,607E-06					1188	SH3D19	3,0	8,669E-04					1188	RPS6KA5	2,0	6,855E-05				
1191	1189	ESCO2	4,1	9,462E-04					1189	GLCCI1	3,0	4,514E-03					1189	EFNA4	2,0	5,192E-03				
1192	1190	RILPL2	4,1	8,098E-04					1190	ZBTB10	3,0	1,338E-02					1190	DENND2D	2,0	2,856E-03				
1193	1191	VMA21	4,1	1,462E-04					1191	STARD13	3,0	9,211E-04					1191	PRELID2	2,0	3,157E-03				
1194	1192	PRMT6	4,1	8,445E-05					1192	NLRP1	3,0	2,089E-04					1192	FADS2	2,0	2,479E-02				
1195	1193	TFPI2	4,1	2,811E-06					1193	UHMK1	3,0	6,184E-03					1193	LARS2	2,0	8,871E-06				
1196	1194	OIP5	4,1	1,735E-04					1194	EEF2K	3,0	1,779E-03					1194	BIN1	2,0	8,274E-04				

	A	B	C	D	E	F	G	H	I	J	K	L	M	N	O	P	Q	R	S	T	U	V	W	X
1197	1195	NPAS3	4,1	2,712E-05					1195	PRMT10	3,0	1,110E-03					1195	OPTN	2,0	2,287E-05				
1198	1196	CCND3	4,1	5,439E-03					1196	RPL32P3	3,0	5,092E-04					1196	ZCCHC7	2,0	4,964E-03				
1199	1197	PCDH9	4,1	5,116E-05					1197	APAF1	2,9	6,432E-03					1197	PRKAR1B	2,0	2,259E-03				
1200	1198	ICA1L	4,1	8,902E-04					1198	CBL	2,9	2,556E-05					1198	LYSMD2	2,0	1,211E-03				
1201	1199	LMNB1	4,1	1,243E-05					1199	CYLD	2,9	6,295E-03					1199	RNF149	2,0	9,657E-05				
1202	1200	MB21D1	4,1	4,589E-07					1200	KIAA0586	2,9	1,846E-03					1200	LYAR	2,0	2,100E-04				
1203	1201	NOTCH4	4,1	2,466E-02					1201	MAP9	2,9	6,533E-03					1201	ZNF485	2,0	3,059E-03				
1204	1202	ZNF442	4,1	2,969E-04					1202	SV2A	2,9	3,812E-05					1202	DHCR7	2,0	8,520E-05				
1205	1203	MTERFD3	4,1	1,253E-03					1203	VPS8	2,9	5,629E-06					1203	MYO1E	2,0	5,964E-06				
1206	1204	NUP188	4,1	1,443E-06					1204	PI4K2B	2,9	5,347E-04					1204	ANKIB1	2,0	6,818E-05				
1207	1205	FANCE	4,1	4,539E-08					1205	EFCAB7	2,9	1,214E-03					1205	VGLL4	2,0	2,121E-04				
1208	1206	ORC6	4,1	2,341E-02					1206	PCMTD1	2,9	4,989E-03					1206	YDJC	2,0	8,154E-03				
1209	1207	BHLHB9	4,1	2,265E-03					1207	LMLN	2,9	6,709E-06					1207	MFSD12	2,0	4,960E-03				
1210	1208	ANKRD26	4,1	4,579E-04					1208	SETD7	2,9	3,344E-06					1208	ITPRIP	2,0	1,737E-03				
1211	1209	SOX13	4,1	1,058E-03					1209	CCND3	2,9	8,693E-05					1209	DPYSL4	2,0	4,535E-02				
1212	1210	APOBEC3G	4,1	5,238E-05					1210	BORA	2,9	7,110E-05					1210	APOBEC3F	2,0	8,751E-04				
1213	1211	AK5	4,1	1,863E-04					1211	KIAA1524	2,9	3,390E-03					1211	SRPK1	2,0	9,813E-04				
1214	1212	ZNF227	4,1	3,401E-04					1212	BBS2	2,9	6,019E-05					1212	TIFA	2,0	2,523E-03				
1215	1213	CNN3	4,1	3,401E-05					1213	BHLHB9	2,9	1,060E-02												
1216	1214	CBX5	4,1	1,514E-04					1214	ASTE1	2,9	4,996E-05												
1217	1215	LNP1	4,1	2,517E-03					1215	SIK3	2,9	1,383E-04												
1218	1216	TXLNG	4,0	3,118E-04					1216	SCPEP1	2,9	1,730E-06												
1219	1217	MORN1	4,0	5,338E-03					1217	ATP11A	2,9	2,066E-02												
1220	1218	WDR19	4,0	3,799E-04					1218	RNF114	2,9	7,371E-07												
1221	1219	USP30	4,0	3,710E-04					1219	RFC3	2,9	2,432E-04												
1222	1220	ATG10	4,0	1,503E-06					1220	NUDT4	2,9	3,461E-02												
1223	1221	CYFIP2	4,0	2,259E-03					1221	SETD9	2,9	1,353E-04												
1224	1222	CCNF	4,0	9,201E-05					1222	DPY19L2	2,9	2,459E-02												
1225	1223	TRIM32	4,0	3,084E-02					1223	HYAL2	2,9	1,531E-03												
1226	1224	INTU	4,0	4,282E-02					1224	GATS	2,9	2,322E-03												
1227	1225	POLA1	4,0	9,623E-05					1225	SLC16A1	2,9	1,903E-05												
1228	1226	CCDC77	4,0	4,923E-04					1226	CUL9	2,9	2,020E-03												
1229	1227	MLYCD	4,0	1,193E-03					1227	CD274	2,9	8,026E-04												
1230	1228	MSI2	4,0	1,774E-04					1228	ZC3H12C	2,9	8,404E-05												
1231	1229	CENPV	4,0	1,692E-05					1229	PLEKHF2	2,9	5,368E-04												
1232	1230	LCORL	4,0	4,407E-03					1230	BCL9	2,9	3,268E-05												
1233	1231	HAGH	4,0	3,248E-03					1231	GCLC	2,9	3,272E-03												
1234	1232	PHF10	4,0	1,541E-05					1232	EXTL2	2,9	1,419E-05												
1235	1233	TRAF4	4,0	5,360E-05					1233	UNC119B	2,9	2,967E-06												
1236	1234	MR1	4,0	2,238E-06					1234	SH3BP1	2,9	3,644E-04												
1237	1235	DONSON	4,0	5,780E-05					1235	IFT57	2,9	1,751E-04												
1238	1236	XRCC3	4,0	1,342E-03					1236	TFPI	2,9	1,393E-03												
1239	1237	DNAJC28	4,0	7,008E-03					1237	ZSCAN5A	2,9	1,106E-03												
1240	1238	MPHOSPH9	4,0	8,099E-03					1238	MLYCD	2,9	6,795E-04												
1241	1239	PPAPDC2	4,0	1,345E-03					1239	SIRT1	2,9	1,042E-04												
1242	1240	IST1	4,0	1,930E-05					1240	TRIM13	2,9	2,788E-02												
1243	1241	P4HTM	4,0	2,477E-03					1241	HIST1H2BD	2,9	2,516E-03												
1244	1242	ZBED3	4,0	3,549E-05					1242	ALDH3A2	2,9	2,154E-02												
1245	1243	RPP38	4,0	1,400E-03					1243	LCORL	2,9	1,156E-03												
1246	1244	HIBADH	4,0	3,410E-07					1244	PSD3	2,9	3,329E-04												
1247	1245	ZNF546	4,0	1,483E-02					1245	SLC12A6	2,9	9,662E-03												
1248	1246	POP5	4,0	7,222E-05					1246	DIO2	2,9	5,805E-05												
1249	1247	HEBP2	3,9	3,099E-02					1247	DHTKD1	2,9	4,252E-05												
1250	1248	NPHP1	3,9	3,316E-04					1248	DNASE1	2,9	1,010E-03												
1251	1249	DST	3,9	4,621E-04					1249	RPAIN	2,9	1,244E-04												
1252	1250	PDIK1L	3,9	1,382E-04					1250	TACC1	2,9	2,449E-04												
1253	1251	IFIH1	3,9	7,875E-03					1251	IL17D	2,9	2,689E-05												
1254	1252	RFWD3	3,9	2,477E-05					1252	TDRD7	2,9	6,821E-05												
1255	1253	FANCI	3,9	7,059E-05					1253	AP3M2	2,9	6,117E-05												
1256	1254	ZFP28	3,9	2,416E-04					1254	TRPC1	2,9	2,951E-04												
1257	1255	APOO	3,9	2,789E-06					1255	BUD13	2,9	8,079E-05												
1258	1256	ERCC6L	3,9	6,047E-04					1256	ODC1	2,8	4,335E-06												
1259	1257	CEP41	3,9	1,876E-02					1257	KLF9	2,8	2,488E-05												
1260	1258	BRCA1	3,9	5,608E-04					1258	CENPQ	2,8	1,324E-04												
1261	1259	DTX3L	3,9	6,444E-05					1259	ZNF185	2,8	7,856E-04												
1262	1260	TOPORS	3,9	1,333E-04					1260	CHN1	2,8	1,198E-03												
1263	1261	FBXO10	3,9	1,833E-03					1261	RAB40B	2,8	1,017E-03												
1264	1262	CCDC126	3,9	3,987E-03					1262	COX15	2,8	1,044E-04												
1265	1263	TSPYL1	3,9	5,239E-08					1263	SLC25A17	2,8	2,615E-02												
1266	1264	C1orf112	3,9	5,112E-03					1264	CEP290	2,8	1,441E-03												
1267	1265	SKA1	3,9	8,474E-04					1265	PARP12	2,8	1,508E-05												
1268	1266	MAP9	3,9	1,560E-03					1266	TTC28-AS1	2,8	9,252E-04												
1269	1267	SKA2	3,9	2,211E-04					1267	ITPKB	2,8	3,038E-04												
1270	1268	ZNF107	3,9	6,985E-05					1268	RNGTT	2,8	4,284E-04												
1271	1269	SNAPIN	3,9	1,487E-04					1269	DLG5	2,8	1,528E-03												
1272	1270	MCM10	3,9	1,027E-05					1270	DNAJC14	2,8	2,093E-03												
1273	1271	CDCA2	3,9	1,181E-05					1271	USP30	2,8	8,689E-04												
1274	1272	MED9	3,9	3,213E-06					1272	NANOS1	2,8	4,030E-02												
1275	1273	C6orf57	3,9	2,559E-04					1273	PPP1R2	2,8	3,539E-05												
1276	1274	TTF2	3,9	3,310E-05					1274	INTS2	2,8	1,034E-03												
1277	1275	NUP153	3,9	2,752E-05					1275	PCYOX1L	2,8	1,333E-05												
1278	1276	TSPAN13	3,9	1,367E-04					1276	MTMR1	2,8	4,174E-04												
1279	1277	SFXN5	3,9	1,897E-04					1277	EPHA2	2,8	1,266E-04												
1280	1278	ASTN2	3,9	1,213E-02					1278	EPS8	2,8	2,315E-03												
1281	1279	TMEM206	3,9	1,232E-04					1279	GABARAPL2	2,8	7,385E-05												
1282	1280	UTY	3,9	1,110E-03					1280	MZT1	2,8	2,787E-04												
1283	1281	TOM1L1	3,9	6,049E-04					1281	TLCD1	2,8	8,909E-04												
1284	1282	SOCS2	3,9	2,431E-03					1282	BCL2L1	2,8	1,851E-05												
1285	1283	ZNF92	3,9	9,906E-05					1283	ETNK1	2,8	3,706E-03												
1286	1284	COCH	3,9	9,375E-04					1284	KAT2B	2,8	1,472E-03												
1287	1285	CCHCR1	3,9	3,683E-04					1285	PRDM2	2,8	1,559E-05												
1288	1286	TRDMT1	3,9	5,970E-03					1286	TAF4B	2,8	1,715E-03												
1289	1287	DENND1A	3,9	2,307E-04					1287	MDK	2,8	6,537E-04												
1290	1288	C4orf21	3,8	4,671E-02					1288	ARMC8	2,8	4,881E-04												
1291	1289	CYB5A	3,8	1,708E-04					1289	TRDMT1	2,8	2,308E-02												
1292	1290	TACC3	3,8	1,313E-03					1290	KIAA2018	2,8	4,758E-03												
1293	1291	TMED4	3,8	1,338E-06					1291	SLC27A1	2,8	1,494E-03												
1294	1292	PFKM	3,8	7,799E-09					1292	MAP3K7	2,8	1,657E-04												
1295	1293	MKS1	3,8	1,042E-04					1293	AKAP1	2,8	2,356E-04												
1296	1294	TFB1M	3,8	6,771E-05					1294	H1F0	2,8	1,768E-05												
1297	1295	GABARAPL2	3,8	2,466E-05					1295	ZNF248	2,8	1,817E-03												
1298	1296	SLC44A1	3,8	9,975E-06					1296	SPICE1	2,8	1,342E-03												
1299	1297	KIAA0556	3,8	3,424E-05					1297	TTC23	2,8	2,070E-03												
1300	1298	ZNF138	3,8	2,023E-03					1298	ISY1	2,8	2,170E-02												
1301	1299	BICD1	3,8	2,391E-03					1299	WASF3	2,8	6,807E-05												
1302	1300	AGTRAP	3,8	5,799E-03					1300	TSHZ2	2,8	6,610E-04												
1303	1301	NAA38	3,8	1,488E-03					1301	PIK3IP1	2,8	3,871E-03												
1304	1302	ATAT1	3,8	1,025E-03					1302	DUSP3	2,8	8,073E-07												
1305	1303	OMA1	3,8	9,135E-07					1303	LEMD3	2,8	4,785E-04												
1306	1304	KHDC1	3,8	3,087E-04					1304	BRD7	2,8	1,303E-04												
1307	1305	SLC39A8	3,8	2,381E-03					1305	HAGH	2,8	4,434E-03												
1308	1306	UPF3B	3,8	2,139E-05					1306	GLI3	2,8	3,637E-04												
1309	1307	PTTG1	3,8	7,277E-05					1307	PHKG2	2,8	2,695E-04												
1310	1308	DHX57	3,8	1,999E-04					1308	HYOU1	2,8	1,020E-03												
1311	1309	AGTPBP1	3,8	9,091E-06					1309	INPP1	2,8	9,406E-05												
1312	1310	SNHG9	3,8	2,378E-04					1310	LNX2	2,8	1,087E-04												
1313	1311	CDC5L	3,8	1,901E-05					1311	HMGB2	2,8	8,289E-03												
1314	1312	ZFP90	3,8	2,134E-03					1312	ZNF718	2,8	3,668E-02												
1315	1313	CCDC104	3,8	3,633E-06					1313	LIG4	2,8	9,735E-07												
1316	1314	APCDD1	3,8	4,239E-04					1314	KCNS3	2,8	9,395E-04												
1317	1315	FAM118A	3,8	2,569E-05					1315	ERMAP	2,8	3,548E-06												
1318	1316	TRAFD1	3,8	1,301E-04					1316	GNS	2,8	2,067E-03												
1319	1317	INTS6-AS1	3,8	1,673E-04					1317	DNAJC16	2,8	5,357E-03												
1320	1318	ARHGAP19	3,8	3,721E-03					1318	PRC1	2,8	2,458E-04												
1321	1319	DARS2	3,8	1,073E-04					1319	MTMR4	2,8	7,677E-06												
1322	1320	TM7SF3	3,8	7,754E-04					1320	PIK3R4	2,8	1,894E-04												
1323	1321	HAUS1	3,8	4,878E-06					1321	SACS	2,8	5,199E-05												
1324	1322	FAXC	3,8	1,292E-02					1322	UXS1	2,8	5,780E-05												
1325	1323	CD44	3,8	1,115E-04					1323	FAM8A1	2,8	4,411E-06												
1326	1324	TMEM8B	3,8	2,644E-03					1324	FICD	2,8	1,818E-04												
1327	1325	GIMAP2	3,8	1,436E-02					1325	COBLL1	2,8	2,211E-02												
1328	1326	C5orf63	3,8	2,947E-04					1326	SLC7A11	2,8	2,513E-05												
1329	1327	RRAGA	3,8	2,150E-06					1327	METTL9	2,8	2,076E-04												
1330	1328	RPGRIP1L	3,8	7,574E-04					1328	SMCHD1	2,8	1,475E-05												
1331	1329	ZNF771	3,8	3,406E-03					1329	STXBP3	2,8	1,415E-04												
1332	1330	RFC3	3,8	1,011E-04					1330	MCCC2	2,8	1,080E-04												
1333	1331	PRIM2	3,8	8,425E-05					1331	DNAJC10	2,8	9,943E-03												
1334	1332	WT1	3,8	1,460E-04					1332	SNX7	2,8	1,318E-05												
1335	1333	NUP37	3,7	1,092E-04					1333	EIF5A2	2,8	1,074E-03												
1336	1334	FAM124A	3,7	1,925E-03					1334	SEC62	2,8	1,269E-02												
1337	1335	TOM1L2	3,7	1,863E-04					1335	SLC2A8	2,8	1,707E-04												
1338	1336	PRPSAP2	3,7	1,161E-03					1336	C10orf10	2,8	5,740E-04												
1339	1337	EXOSC9	3,7	1,849E-05					1337	DHX36	2,8	3,393E-04												
1340	1338	PGRMC1	3,7	4,924E-07					1338	C5orf15	2,8	4,371E-02												
1341	1339	PDRG1	3,7	1,439E-04					1339	FGD6	2,8	7,353E-03												
1342	1340	DOLPP1	3,7	1,582E-05					1340	EIF2AK3	2,8	1,467E-04												
1343	1341	SPATA5	3,7	2,377E-03					1341	STAG2	2,7	1,884E-03												
1344	1342	DDIT4	3,7	3,888E-04					1342	NAP1L5	2,7	5,157E-05												
1345	1343	PPM1D	3,7	7,148E-05					1343	CHST14	2,7	5,127E-05												
1346	1344	SLC12A6	3,7	4,714E-04					1344	CYP2U1	2,7	2,566E-06												
1347	1345	CDC20	3,7	1,274E-06					1345	RB1	2,7	5,034E-07												
1348	1346	CRYGS	3,7	1,222E-02					1346	TSPAN13	2,7	1,566E-06												
1349	1347	GLRB	3,7	4,713E-02					1347	AIFM2	2,7	1,802E-03												
1350	1348	PBXIP1	3,7	4,316E-05					1348	HOXB3	2,7	3,286E-03												
1351	1349	CNOT1	3,7	1,302E-04					1349	CREB3L2	2,7	1,624E-06												
1352	1350	TMEM144	3,7	9,561E-03					1350	SESN1	2,7	6,172E-04												
1353	1351	ORAI2	3,7	7,054E-03					1351	STIM2	2,7	1,532E-04												
1354	1352	TAF1A	3,7	4,795E-04					1352	DISC1	2,7	9,491E-03												
1355	1353	DEPDC1	3,7	2,350E-04					1353	TOPBP1	2,7	7,343E-04												
1356	1354	CHST3	3,7	3,024E-06					1354	C12orf76	2,7	2,551E-05												
1357	1355	SMC1A	3,7	3,019E-04					1355	SFR1	2,7	9,826E-03												
1358	1356	LDHB	3,7	1,829E-05					1356	ARHGAP42	2,7	4,219E-05												
1359	1357	OSGIN1	3,7	8,316E-03					1357	MECOM	2,7	1,910E-03												
1360	1358	PDGFD	3,7	2,103E-03					1358	NDUFB5	2,7	1,999E-02												
1361	1359	SLC2A13	3,7	2,199E-03					1359	PTK7	2,7	6,183E-04												
1362	1360	C17orf103	3,7	3,349E-04					1360	MTMR3	2,7	2,624E-04												
1363	1361	NUDT21	3,7	1,305E-04					1361	SLC11A2	2,7	5,536E-04												
1364	1362	C8orf4	3,7	1,314E-02					1362	GSTZ1	2,7	5,041E-03												
1365	1363	COX15	3,7	3,067E-05					1363	KANSL3	2,7	1,293E-04												
1366	1364	HLTF	3,7	2,276E-04					1364	C9orf64	2,7	4,522E-05												
1367	1365	NUDT5	3,7	7,028E-03					1365	YBX2	2,7	3,529E-02												
1368	1366	SYCE2	3,7	4,256E-03					1366	PPTC7	2,7	3,911E-04												
1369	1367	PDK3	3,7	6,183E-06					1367	BTBD9	2,7	1,107E-03												
1370	1368	ARHGAP39	3,7	2,859E-02					1368	SEC22A	2,7	9,502E-04												
1371	1369	SH2B1	3,7	3,175E-02					1369	TMEM123	2,7	5,588E-07												
1372	1370	PFKFB2	3,7	9,511E-04					1370	RASSF2	2,7	5,102E-03												
1373	1371	RAD54L	3,7	8,600E-06					1371	QSOX2	2,7	7,502E-06												
1374	1372	ANAPC7	3,7	2,296E-04					1372	RBM7	2,7	3,262E-02												
1375	1373	MAP3K13	3,6	2,599E-05					1373	MGAT5B	2,7	1,175E-03												
1376	1374	PPAP2A	3,6	5,364E-05					1374	PHOSPHO2	2,7	1,723E-02												
1377	1375	C4orf33	3,6	4,250E-03					1375	MMP19	2,7	1,993E-05												
1378	1376	DDX28	3,6	3,597E-06					1376	STAT5B	2,7	1,001E-04												
1379	1377	ZNF583	3,6	9,421E-03					1377	FANCI	2,7	1,034E-03												
1380	1378	PUS10	3,6	1,765E-04					1378	KIAA0319L	2,7	2,142E-05												
1381	1379	FAM64A	3,6	1,122E-04					1379	C2	2,7	2,344E-03												
1382	1380	ACAT2	3,6	1,471E-05					1380	ZMYM4	2,7	3,838E-04												
1383	1381	ZNF701	3,6	3,410E-04					1381	TULP3	2,7	4,347E-04												
1384	1382	NCAPG	3,6	1,109E-04					1382	PHF10	2,7	1,191E-05												
1385	1383	MCCC1	3,6	3,728E-03					1383	TBK1	2,7	3,420E-04												
1386	1384	BRD7	3,6	3,367E-03					1384	BTG2	2,7	2,968E-02												
1387	1385	GOLPH3L	3,6	6,745E-05					1385	SH2B3	2,7	1,323E-04												
1388	1386	TSPAN2	3,6	8,715E-05					1386	LRBA	2,7	1,481E-03												
1389	1387	C14orf79	3,6	9,619E-06					1387	CCDC50	2,7	1,596E-04												
1390	1388	C3orf33	3,6	6,248E-04					1388	TMED8	2,7	1,119E-04												
1391	1389	MTX3	3,6	1,813E-04					1389	ARSB	2,7	2,629E-04												
1392	1390	RBM7	3,6	1,594E-02					1390	SOX9	2,7	6,569E-05												
1393	1391	POC5	3,6	1,773E-06					1391	IGF2R	2,7	4,727E-05												
1394	1392	LYRM2	3,6	5,701E-05					1392	GINS4	2,7	5,086E-04												
1395	1393	CCDC28A	3,6	7,360E-04					1393	PCID2	2,7	2,632E-03												
1396	1394	CHPT1	3,6	3,742E-02					1394	HS2ST1	2,7	3,752E-05												
1397	1395	MKL2	3,6	7,274E-04					1395	CDK17	2,7	2,015E-03												
1398	1396	CCNA2	3,6	2,656E-06					1396	SNX29	2,7	1,096E-03												

	A	B	C	D	E	F	G	H	I	J	K	L	M	N	O	P	Q	R	S	T	U	V	W	X
1399	1397	FBXO21	3,6	3,598E-03					1397	LRRC8A	2,7	8,496E-05												
1400	1398	ZNF329	3,6	1,883E-03					1398	PRR5L	2,7	4,934E-02												
1401	1399	POLD3	3,6	4,577E-05					1399	TMPO	2,7	8,287E-04												
1402	1400	OGFRL1	3,6	1,249E-04					1400	DPYD	2,7	5,880E-05												
1403	1401	SPSB2	3,6	2,425E-02					1401	EXO1	2,7	4,190E-03												
1404	1402	CDKL3	3,6	9,844E-03					1402	FYN	2,7	2,761E-04												
1405	1403	AEBP2	3,6	4,562E-03					1403	ABHD13	2,7	6,516E-06												
1406	1404	PROS1	3,6	6,757E-04					1404	RCBTB1	2,7	1,762E-03												
1407	1405	EAF1	3,6	1,109E-04					1405	KIF5C	2,7	2,058E-05												
1408	1406	AFG3L1P	3,6	1,434E-04					1406	C2orf81	2,7	4,663E-02												
1409	1407	ZNF124	3,6	2,635E-02					1407	TXNL4B	2,7	1,213E-03												
1410	1408	PEX12	3,6	1,352E-04					1408	MCM2	2,7	5,909E-05												
1411	1409	SNX11	3,6	1,201E-05					1409	SENP1	2,7	1,263E-02												
1412	1410	TNRC6C	3,6	1,417E-02					1410	SMG8	2,7	3,220E-05												
1413	1411	AGER	3,6	2,002E-03					1411	NR1H3	2,7	1,499E-03												
1414	1412	PYGO2	3,6	1,224E-05					1412	RGL1	2,7	3,125E-03												
1415	1413	FNDC1	3,6	3,651E-06					1413	RAB28	2,7	5,851E-04												
1416	1414	RANGRF	3,6	3,021E-03					1414	FAM118B	2,7	1,843E-03												
1417	1415	AHCYL2	3,6	8,275E-03					1415	ARHGEF25	2,7	8,790E-04												
1418	1416	DNAJA1	3,6	1,632E-07					1416	LIPG	2,7	4,859E-06												
1419	1417	SNAPC3	3,6	5,741E-03					1417	PHF12	2,7	5,482E-04												
1420	1418	NDUFB6	3,6	5,747E-05					1418	KLHL8	2,7	2,967E-04												
1421	1419	C12orf66	3,6	4,860E-04					1419	ZNF513	2,7	1,141E-03												
1422	1420	LSM5	3,6	2,060E-05					1420	B3GALTL	2,7	3,351E-06												
1423	1421	AKAP7	3,6	1,584E-03					1421	SF3B3	2,7	2,344E-06												
1424	1422	BCHE	3,6	1,282E-04					1422	TTC17	2,7	1,094E-05												
1425	1423	ANXA1	3,6	1,054E-04					1423	WDR61	2,7	6,935E-04												
1426	1424	ASPN	3,6	2,385E-02					1424	PLD1	2,7	3,614E-02												
1427	1425	ZNF585A	3,6	4,098E-04					1425	GPR107	2,7	5,758E-08												
1428	1426	SLC25A35	3,5	2,659E-04					1426	ASAP1	2,7	1,431E-04												
1429	1427	DCHS1	3,5	6,719E-06					1427	ACTR6	2,7	2,486E-03												
1430	1428	C8orf31	3,5	5,791E-03					1428	TMEM41A	2,7	1,308E-03												
1431	1429	MCM7	3,5	4,134E-06					1429	DENND3	2,7	9,845E-03												
1432	1430	SMARCE1	3,5	9,817E-03					1430	LGALS3	2,7	4,805E-05												
1433	1431	LRP8	3,5	1,537E-04					1431	CEACAM19	2,7	2,325E-02												
1434	1432	RBBP4	3,5	3,438E-04					1432	RAD51AP1	2,7	9,225E-04												
1435	1433	KIAA1671	3,5	7,795E-05					1433	TFG	2,6	1,306E-05												
1436	1434	PLA2G6	3,5	7,937E-03					1434	MYLIP	2,6	6,096E-04												
1437	1435	GPSM2	3,5	8,937E-06					1435	HEATR5A	2,6	8,710E-04												
1438	1436	MFHAS1	3,5	1,465E-06					1436	DDX60L	2,6	1,560E-02												
1439	1437	ZNF629	3,5	8,490E-05					1437	HAUS6	2,6	2,835E-05												
1440	1438	DLGAP5	3,5	6,982E-06					1438	PJA2	2,6	3,924E-06												
1441	1439	MEIS2	3,5	1,564E-04					1439	DOCK7	2,6	6,754E-07												
1442	1440	RASSF9	3,5	2,174E-04					1440	CDKN3	2,6	1,975E-03												
1443	1441	QKI	3,5	4,477E-02					1441	MASTL	2,6	1,089E-04												
1444	1442	SRGAP1	3,5	1,234E-04					1442	L2HGDH	2,6	1,905E-03												
1445	1443	RHBDD2	3,5	2,207E-03					1443	TRIM62	2,6	5,014E-04												
1446	1444	ESPL1	3,5	2,954E-05					1444	MOAP1	2,6	8,943E-06												
1447	1445	RNF138	3,5	5,145E-04					1445	ABHD10	2,6	9,775E-03												
1448	1446	ALG6	3,5	5,961E-03					1446	SHCBP1	2,6	1,030E-03												
1449	1447	STK36	3,5	3,049E-05					1447	TMEM86B	2,6	7,177E-03												
1450	1448	TMEM186	3,5	2,135E-04					1448	DHX8	2,6	2,663E-04												
1451	1449	DENND4C	3,5	3,472E-06					1449	MTM1	2,6	8,328E-03												
1452	1450	ZNF527	3,5	1,652E-03					1450	HDAC1	2,6	1,048E-04												
1453	1451	PTAR1	3,5	1,300E-04					1451	C1orf56	2,6	2,165E-02												
1454	1452	CREG1	3,5	2,164E-04					1452	CAB39L	2,6	1,049E-03												
1455	1453	C9orf40	3,5	2,499E-05					1453	VAMP1	2,6	6,806E-04												
1456	1454	TSPAN31	3,5	2,232E-03					1454	PGGT1B	2,6	5,107E-05												
1457	1455	BRMS1L	3,5	2,642E-04					1455	PLEKHM3	2,6	1,084E-02												
1458	1456	G2E3	3,5	2,632E-03					1456	ZNF84	2,6	2,508E-02												
1459	1457	POC1B	3,5	9,546E-05					1457	PTGER4	2,6	2,662E-03												
1460	1458	ABCA11P	3,5	8,251E-03					1458	MAPRE2	2,6	6,060E-04												
1461	1459	H1FX	3,5	2,088E-02					1459	ANKRD50	2,6	4,115E-05												
1462	1460	TPX2	3,5	4,299E-05					1460	TMEM17	2,6	2,376E-02												
1463	1461	PHIP	3,5	2,913E-04					1461	MAD2L1BP	2,6	2,284E-05												
1464	1462	NUDT12	3,5	1,265E-04					1462	DCP1B	2,6	1,146E-03												
1465	1463	C7orf61	3,5	1,408E-03					1463	IRS1	2,6	4,478E-05												
1466	1464	SLC46A1	3,5	3,414E-04					1464	TIFA	2,6	4,822E-05												
1467	1465	GUSB	3,5	1,866E-04					1465	TTLL5	2,6	2,078E-04												
1468	1466	IFT80	3,5	3,939E-04					1466	PDXP	2,6	1,919E-05												
1469	1467	NUP107	3,5	1,932E-05					1467	RAD51C	2,6	1,159E-04												
1470	1468	ZNF10	3,5	1,292E-02					1468	POLR2B	2,6	8,115E-06												
1471	1469	GPATCH2	3,5	7,490E-04					1469	NOP14-AS1	2,6	3,888E-05												
1472	1470	AP3B2	3,5	3,364E-03					1470	ZCCHC9	2,6	3,907E-04												
1473	1471	TTLL7	3,5	1,472E-03					1471	ZBTB41	2,6	1,369E-04												
1474	1472	CARHSP1	3,5	2,573E-04					1472	FAM122C	2,6	7,770E-03												
1475	1473	ABR	3,5	4,474E-04					1473	KCTD13	2,6	1,767E-03												
1476	1474	PKIA	3,5	1,394E-03					1474	GJC1	2,6	4,711E-03												
1477	1475	SF3B3	3,5	2,338E-05					1475	IGFBP5	2,6	8,939E-07												
1478	1476	ZNF764	3,5	3,643E-03					1476	C12orf49	2,6	2,884E-04												
1479	1477	EPB41L2	3,5	1,572E-04					1477	KNTC1	2,6	1,448E-04												
1480	1478	H2AFX	3,5	1,273E-02					1478	TNFRSF10B	2,6	5,612E-05												
1481	1479	NUP62	3,5	1,720E-04					1479	LARP1B	2,6	1,857E-03												
1482	1480	GABPA	3,5	4,015E-05					1480	POGLUT1	2,6	3,522E-04												
1483	1481	MED31	3,5	8,361E-05					1481	TNFRSF14	2,6	7,978E-04												
1484	1482	TPK1	3,5	1,686E-02					1482	CENPE	2,6	9,328E-03												
1485	1483	RARRES3	3,5	2,189E-04					1483	CLSTN1	2,6	7,921E-06												
1486	1484	RDH13	3,5	3,585E-03					1484	ZFHX3	2,6	1,797E-03												
1487	1485	SMPDL3A	3,5	3,290E-04					1485	C16orf80	2,6	7,614E-05												
1488	1486	SLC25A40	3,5	2,673E-04					1486	IP6K1	2,6	9,088E-05												
1489	1487	KIAA1161	3,5	1,227E-04					1487	HSDL1	2,6	2,724E-04												
1490	1488	ZNF300	3,5	1,087E-02					1488	RILPL2	2,6	1,064E-04												
1491	1489	CASC5	3,5	1,324E-03					1489	CMAS	2,6	2,055E-05												
1492	1490	PDP2	3,4	4,689E-05					1490	PON2	2,6	2,799E-03												
1493	1491	RBMXL1	3,4	1,602E-04					1491	SLC44A1	2,6	2,639E-05												
1494	1492	CDC25C	3,4	7,982E-04					1492	PRPF4B	2,6	4,280E-04												
1495	1493	CREM	3,4	4,387E-04					1493	TM2D1	2,6	1,483E-03												
1496	1494	HSDL1	3,4	3,530E-04					1494	ELOVL5	2,6	4,658E-03												
1497	1495	PPP1R15B	3,4	5,537E-05					1495	AMFR	2,6	2,424E-04												
1498	1496	GABARAPL1	3,4	2,011E-02					1496	ZNF644	2,6	2,308E-04												
1499	1497	ACBD4	3,4	8,722E-03					1497	SPAG5	2,6	6,536E-05												
1500	1498	FUBP1	3,4	1,293E-05					1498	EFTUD2	2,6	1,057E-03												
1501	1499	EID2B	3,4	6,958E-05					1499	MANEA	2,6	1,498E-03												
1502	1500	COQ9	3,4	1,825E-04					1500	CERK	2,6	1,914E-07												
1503	1501	RNGTT	3,4	4,494E-05					1501	RAP1GAP2	2,6	1,128E-03												
1504	1502	TFDP2	3,4	8,291E-04					1502	LNP1	2,6	1,204E-03												
1505	1503	RARRES1	3,4	1,010E-02					1503	GNL1	2,6	1,680E-05												
1506	1504	MTF1	3,4	4,122E-05					1504	STK38L	2,6	3,972E-03												
1507	1505	GEMIN6	3,4	2,434E-03					1505	DEPDC1B	2,6	2,912E-03												
1508	1506	ID2	3,4	5,565E-03					1506	CCDC125	2,6	7,473E-03												
1509	1507	PARN	3,4	5,834E-06					1507	TM7SF3	2,6	1,442E-04												
1510	1508	BUB1B	3,4	7,235E-04					1508	ATP6V0E2	2,6	1,422E-03												
1511	1509	IFI27L1	3,4	3,089E-04					1509	MLX	2,6	1,448E-04												
1512	1510	PACRGL	3,4	3,862E-05					1510	TEX9	2,6	1,064E-02												
1513	1511	ZFX	3,4	3,242E-06					1511	ANKRD26	2,6	1,955E-03												
1514	1512	ZBTB33	3,4	5,391E-05					1512	HIST1H4H	2,6	6,514E-03												
1515	1513	PRRT3	3,4	1,709E-02					1513	NAA50	2,6	8,869E-03												
1516	1514	TMCO1	3,4	6,731E-05					1514	ANKFY1	2,6	1,787E-05												
1517	1515	ACVR1B	3,4	5,951E-04					1515	PSMC3IP	2,6	8,423E-05												
1518	1516	AURKB	3,4	6,210E-04					1516	PNPO	2,6	3,846E-05												
1519	1517	SUV39H1	3,4	4,941E-05					1517	ST7-AS1	2,6	2,679E-05												
1520	1518	ODF2	3,4	7,927E-08					1518	RAPGEFL1	2,6	1,264E-03												
1521	1519	LRRC1	3,4	2,763E-04					1519	TIAM2	2,6	1,834E-03												
1522	1520	PGAM5	3,4	3,348E-05					1520	METTL21A	2,6	1,750E-03												
1523	1521	CEP290	3,4	3,129E-04					1521	CCBL2	2,6	1,271E-05												
1524	1522	KIF2C	3,4	6,541E-07					1522	KLF3	2,6	1,094E-05												
1525	1523	TMTC1	3,4	1,529E-05					1523	JKAMP	2,6	9,505E-03												
1526	1524	RBM15	3,4	3,503E-05					1524	CDH13	2,6	3,664E-04												
1527	1525	RPA3	3,4	1,335E-05					1525	POMGNT1	2,6	1,883E-04												
1528	1526	GNAZ	3,4	1,047E-05					1526	RSF1	2,6	8,614E-03												
1529	1527	NT5DC3	3,4	5,532E-05					1527	U2SURP	2,5	3,097E-03												
1530	1528	REEP2	3,4	5,104E-03					1528	LMBR1	2,5	2,345E-02												
1531	1529	COG4	3,4	1,097E-03					1529	EPB41	2,5	1,020E-03												
1532	1530	SPRY4	3,4	5,865E-05					1530	FLOT1	2,5	2,626E-06												
1533	1531	HSBP1	3,4	2,064E-07					1531	CHST11	2,5	1,220E-03												
1534	1532	RAD51C	3,4	8,578E-04					1532	RAB7A	2,5	1,335E-05												
1535	1533	TCEANC2	3,4	1,512E-03					1533	DCLRE1A	2,5	1,778E-03												
1536	1534	MIEN1	3,4	1,471E-03					1534	HLA-B	2,5	8,101E-05												
1537	1535	SLC41A1	3,4	4,291E-04					1535	POLK	2,5	5,953E-04												
1538	1536	XPR1	3,4	1,164E-04					1536	ICK	2,5	3,568E-05												
1539	1537	FOPNL	3,4	1,016E-04					1537	POLR2A	2,5	7,998E-06												
1540	1538	PLA2G4A	3,4	2,916E-03					1538	TLK2	2,5	3,822E-04												
1541	1539	ARRDC4	3,4	1,417E-04					1539	MFN1	2,5	6,916E-05												
1542	1540	DNAJC1	3,4	8,340E-06					1540	APEH	2,5	1,683E-03												
1543	1541	C7orf49	3,4	2,748E-05					1541	CIT	2,5	4,586E-03												
1544	1542	CPT2	3,4	1,229E-06					1542	PAM	2,5	9,460E-06												
1545	1543	VLDLR	3,4	4,888E-05					1543	KIAA1324	2,5	1,177E-02												
1546	1544	SFT2D2	3,4	5,232E-05					1544	ZNF280C	2,5	1,736E-03												
1547	1545	APOA1BP	3,4	8,723E-06					1545	MED9	2,5	6,307E-05												
1548	1546	GRPEL2	3,4	7,781E-04					1546	MCFD2	2,5	2,843E-06												
1549	1547	EXOC7	3,4	1,169E-03					1547	COL18A1	2,5	5,669E-03												
1550	1548	ZNF816	3,4	3,486E-03					1548	TGDS	2,5	4,856E-05												
1551	1549	HSD17B11	3,4	9,166E-04					1549	FBXL14	2,5	4,503E-03												
1552	1550	CPEB2	3,3	4,278E-03					1550	AASDH	2,5	2,460E-04												
1553	1551	RFC2	3,3	3,532E-03					1551	PEX7	2,5	1,863E-03												
1554	1552	KLHL15	3,3	5,784E-04					1552	ATP6V1G2	2,5	3,189E-02												
1555	1553	UCHL1	3,3	2,506E-04					1553	ZNF880	2,5	2,582E-05												
1556	1554	EPHA4	3,3	2,770E-03					1554	PBK	2,5	2,671E-03												
1557	1555	SMARCA2	3,3	3,267E-04					1555	JAM2	2,5	4,163E-02												
1558	1556	C17orf53	3,3	8,090E-04					1556	CELSR3	2,5	3,621E-02												
1559	1557	WDR52	3,3	4,286E-03					1557	LRIG2	2,5	5,306E-03												
1560	1558	REC8	3,3	1,504E-03					1558	FAM161A	2,5	3,513E-02												
1561	1559	PMAIP1	3,3	1,503E-04					1559	IFT81	2,5	8,672E-04												
1562	1560	LONRF1	3,3	1,334E-02					1560	PPT2	2,5	7,389E-06												
1563	1561	FYCO1	3,3	7,725E-03					1561	UBXN7	2,5	6,408E-06												
1564	1562	ZNF79	3,3	3,333E-04					1562	UPP1	2,5	2,104E-04												
1565	1563	SLC25A14	3,3	4,881E-04					1563	MSL2	2,5	5,794E-04												
1566	1564	GINS1	3,3	5,854E-04					1564	MARCH9	2,5	3,186E-04												
1567	1565	DDIT3	3,3	7,720E-05					1565	UBE2T	2,5	8,259E-05												
1568	1566	ATAD2	3,3	5,984E-05					1566	MED28	2,5	8,591E-05												
1569	1567	FAM104B	3,3	5,375E-04					1567	NSUN6	2,5	2,080E-03												
1570	1568	RANBP6	3,3	8,041E-05					1568	ISG20	2,5	1,691E-02												
1571	1569	RFC4	3,3	2,123E-03					1569	USP13	2,5	7,941E-04												
1572	1570	ZFP37	3,3	7,402E-04					1570	DTX3	2,5	1,071E-04												
1573	1571	E2F1	3,3	1,612E-04					1571	TESK2	2,5	5,519E-03												
1574	1572	PREPL	3,3	7,015E-05					1572	ZNF107	2,5	2,319E-03												
1575	1573	ANKRD29	3,3	6,535E-03					1573	NUP188	2,5	1,489E-04												
1576	1574	MARC2	3,3	2,585E-03					1574	INTS6-AS1	2,5	1,419E-03												
1577	1575	ZNF493	3,3	1,681E-03					1575	UNG	2,5	5,617E-05												
1578	1576	MAD2L1BP	3,3	1,432E-05					1576	CCNE1	2,5	7,607E-04												
1579	1577	VPS45	3,3	8,254E-04					1577	SRBD1	2,5	6,830E-05												
1580	1578	CENPA	3,3	2,446E-05					1578	ARMC2	2,5	3,278E-02												
1581	1579	GAB1	3,3	2,024E-02					1579	ZNF852	2,5	4,394E-03												
1582	1580	LHFP	3,3	2,403E-07					1580	CDNF	2,5	3,870E-02												
1583	1581	ANLN	3,3	1,790E-04					1581	AGER	2,5	2,345E-02												
1584	1582	CHCHD10	3,3	3,743E-03					1582	MTRF1	2,5	1,439E-03												
1585	1583	C20orf96	3,3	1,133E-04					1583	POMT2	2,5	1,410E-02												
1586	1584	TTF1	3,3	3,553E-05					1584	CMTM6	2,5	8,970E-05												
1587	1585	HIATL1	3,3	3,272E-06					1585	TACC3	2,5	1,846E-04												
1588	1586	POLA2	3,3	7,286E-04					1586	PIGF	2,5	8,667E-05												
1589	1587	DCTN3	3,3	1,084E-04					1587	RDH10	2,5	2,873E-05												
1590	1588	ZNF26	3,3	1,752E-03					1588	TGIF2	2,5	2,725E-02												
1591	1589	FGGY	3,3	1,848E-02					1589	FAM122B	2,5	4,267E-04												
1592	1590	DDX19A	3,3	2,462E-04					1590	C16orf72	2,5	1,964E-04												
1593	1591	CEP55	3,3	8,282E-05					1591	USP33	2,5	1,743E-05												
1594	1592	PEX6	3,3	1,098E-05					1592	FAM63A	2,5	8,805E-04												
1595	1593	CMBL	3,3	5,245E-04					1593	MAP4K4	2,5	6,571E-06												
1596	1594	INTS7	3,3	7,113E-03					1594	DEPDC1	2,5	1,364E-02												
1597	1595	USP1	3,3	5,667E-04					1595	TMEM128	2,5	3,903E-04												
1598	1596	TYMP	3,3	2,196E-02					1596	KPNA5	2,5	2,358E-02												
1599	1597	CSRP2	3,3	3,810E-04					1597	RNF144A	2,5	5,764E-04												
1600	1598	LBR	3,3	1,456E-04					1598	TXNRD2	2,5	3,226E-03												

	A	B	C	D	I	J	K	L
1601	1599	AGPAT5	3,3	4,835E-04	1599	PCDHB14	2,5	1,608E-03
1602	1600	AURKA	3,3	5,034E-05	1600	SNX11	2,5	1,613E-03
1603	1601	SLC25A25	3,3	2,570E-04	1601	HSPH1	2,5	4,842E-06
1604	1602	MCC	3,3	3,315E-03	1602	PTGFRN	2,5	2,742E-05
1605	1603	ATXN7L3B	3,3	4,196E-07	1603	ARAP3	2,5	3,432E-05
1606	1604	TRIM37	3,3	8,158E-05	1604	PGS1	2,5	4,491E-03
1607	1605	CD55	3,3	1,912E-04	1605	TTC5	2,5	9,203E-05
1608	1606	PIGX	3,3	5,126E-04	1606	CNN3	2,5	6,228E-05
1609	1607	CYB5RL	3,3	4,521E-04	1607	SLC25A36	2,5	4,368E-05
1610	1608	CLCN4	3,3	2,792E-05	1608	RANBP10	2,5	9,026E-05
1611	1609	ZNF24	3,3	1,441E-04	1609	DCP1A	2,5	2,695E-03
1612	1610	PAQR3	3,3	1,983E-03	1610	ZNF26	2,5	1,223E-02
1613	1611	NDUFAB1	3,3	6,421E-04	1611	NCAPH	2,5	2,163E-05
1614	1612	CPT1C	3,3	3,627E-03	1612	TNPO3	2,5	6,616E-05
1615	1613	WDR24	3,3	6,765E-03	1613	LYRM5	2,5	2,985E-04
1616	1614	NAE1	3,3	4,667E-04	1614	SFT2D2	2,5	1,398E-04
1617	1615	FAM120B	3,3	1,686E-05	1615	PSPC1	2,5	2,506E-05
1618	1616	NEK8	3,3	2,224E-03	1616	CHD6	2,5	8,095E-05
1619	1617	AGL	3,3	4,789E-04	1617	CENPBD1	2,5	2,602E-04
1620	1618	HINT3	3,3	6,840E-05	1618	TRIM5	2,5	4,094E-03
1621	1619	PHF6	3,3	1,603E-02	1619	TBXAS1	2,5	2,998E-02
1622	1620	DYRK1A	3,3	3,040E-02	1620	TAOK1	2,5	1,131E-04
1623	1621	PTPRD	3,2	1,686E-02	1621	PPP2R2A	2,5	2,555E-02
1624	1622	TRAF3IP1	3,2	4,492E-04	1622	ESCO2	2,5	1,841E-03
1625	1623	CDKL1	3,2	7,159E-03	1623	STAT2	2,5	3,383E-04
1626	1624	SUZ12	3,2	4,252E-04	1624	ACVR1B	2,5	1,212E-03
1627	1625	TADA1	3,2	1,475E-04	1625	CAMK2N2	2,5	3,415E-05
1628	1626	NFATC2IP	3,2	1,727E-04	1626	FAIM3	2,5	1,569E-02
1629	1627	CMTM8	3,2	1,101E-03	1627	CDV3	2,5	4,511E-02
1630	1628	TRIM13	3,2	3,567E-05	1628	BCR	2,5	5,999E-04
1631	1629	CCDC41	3,2	6,511E-03	1629	USP46	2,5	8,644E-04
1632	1630	SPIN1	3,2	8,976E-04	1630	CHST3	2,5	1,813E-04
1633	1631	TWIST1	3,2	6,156E-03	1631	HSPBAP1	2,5	2,571E-05
1634	1632	LNX2	3,2	1,257E-03	1632	SLC33A1	2,5	4,989E-04
1635	1633	PRC1	3,2	1,452E-03	1633	NEDD1	2,5	3,975E-06
1636	1634	ETV5	3,2	6,210E-06	1634	SENP5	2,5	4,436E-04
1637	1635	TCF19	3,2	2,086E-03	1635	HIP1R	2,5	1,304E-03
1638	1636	AP1G1	3,2	1,432E-05	1636	DHRS13	2,5	1,314E-03
1639	1637	CCNB2	3,2	7,397E-05	1637	C12orf66	2,5	8,448E-04
1640	1638	NKRF	3,2	2,255E-04	1638	WDR5B	2,5	6,586E-05
1641	1639	GTF2I	3,2	2,450E-04	1639	SPATA5	2,5	2,655E-02
1642	1640	GOLM1	3,2	2,676E-06	1640	USP22	2,5	1,529E-05
1643	1641	STIM2	3,2	8,171E-05	1641	TRIM24	2,5	1,697E-03
1644	1642	SESN2	3,2	4,457E-05	1642	VEZF1	2,5	2,357E-04
1645	1643	ABHD11	3,2	8,440E-03	1643	TOP2A	2,5	9,893E-05
1646	1644	CCDC34	3,2	7,130E-05	1644	MED13	2,5	1,377E-04
1647	1645	PIBF1	3,2	2,123E-04	1645	CEP85L	2,5	6,191E-04
1648	1646	KIAA2026	3,2	2,773E-02	1646	ATAT1	2,5	3,631E-03
1649	1647	FAM161B	3,2	2,382E-04	1647	GINS2	2,5	1,160E-03
1650	1648	FOXN2	3,2	2,500E-04	1648	NDRG2	2,5	1,332E-02
1651	1649	SMG8	3,2	1,019E-05	1649	TOB1	2,5	5,287E-03
1652	1650	CLUAP1	3,2	6,074E-03	1650	TMCC1	2,5	2,153E-03
1653	1651	PTPRC	3,2	2,925E-02	1651	SLC37A4	2,5	9,916E-04
1654	1652	DNHD1	3,2	3,432E-03	1652	TOPORS	2,5	2,777E-04
1655	1653	C5orf54	3,2	3,051E-04	1653	ATXN10	2,4	4,253E-05
1656	1654	ZNF391	3,2	1,064E-03	1654	LAMB3	2,4	3,008E-03
1657	1655	SMPD2	3,2	7,863E-05	1655	ATF6B	2,4	5,064E-04
1658	1656	CASP2	3,2	2,470E-06	1656	WWOX	2,4	5,216E-03
1659	1657	CRYZ	3,2	3,293E-03	1657	GTF3C1	2,4	1,980E-03
1660	1658	SNRNP25	3,2	4,965E-04	1658	IFT80	2,4	3,066E-03
1661	1659	TMEM130	3,2	4,141E-03	1659	SLCO3A1	2,4	1,194E-05
1662	1660	POLR1C	3,2	7,043E-05	1660	CERS6	2,4	9,940E-06
1663	1661	CLK2	3,2	1,461E-04	1661	TSPYL1	2,4	6,805E-06
1664	1662	DIAPH3	3,2	1,337E-04	1662	TRAF4	2,4	1,618E-04
1665	1663	ZNF397	3,2	1,024E-03	1663	CYB561	2,4	1,449E-02
1666	1664	SAMD9L	3,2	3,645E-04	1664	TOB2	2,4	4,733E-05
1667	1665	P2RX7	3,2	1,039E-04	1665	DGCR2	2,4	3,352E-05
1668	1666	GOT2	3,2	4,598E-06	1666	GATA6	2,4	1,126E-03
1669	1667	NOTCH1	3,2	3,050E-02	1667	KANSL1	2,4	1,201E-05
1670	1668	C7orf55	3,2	3,080E-03	1668	ETS2	2,4	5,512E-06
1671	1669	GJC1	3,2	3,005E-03	1669	TOM1L1	2,4	5,651E-03
1672	1670	CENPBD1	3,2	3,649E-05	1670	ZNF655	2,4	1,122E-02
1673	1671	CHN1	3,2	4,270E-04	1671	PLA2G6	2,4	8,213E-03
1674	1672	MOB1B	3,2	2,064E-03	1672	TSC22D2	2,4	7,981E-04
1675	1673	CDK14	3,2	5,768E-05	1673	SLC35B3	2,4	1,244E-05
1676	1674	H2AFV	3,2	5,115E-05	1674	TOP1	2,4	3,747E-06
1677	1675	QPRT	3,2	1,211E-03	1675	AGPAT5	2,4	2,478E-05
1678	1676	MINPP1	3,2	2,139E-04	1676	FER	2,4	4,449E-03
1679	1677	PTBP2	3,2	1,257E-03	1677	DCUN1D1	2,4	6,609E-03
1680	1678	CCDC138	3,2	1,957E-03	1678	ATAD2	2,4	2,812E-05
1681	1679	MED23	3,2	1,001E-04	1679	SCOC	2,4	1,735E-05
1682	1680	NECAP1	3,2	1,032E-03	1680	TXNDC12	2,4	2,113E-06
1683	1681	UHRF1BP1	3,2	2,455E-06	1681	GOLPH3L	2,4	1,645E-04
1684	1682	ZNF235	3,2	2,965E-03	1682	CARD6	2,4	2,365E-05
1685	1683	ZNRF2	3,2	1,709E-05	1683	NCOA1	2,4	1,113E-04
1686	1684	C6orf136	3,2	1,097E-02	1684	SPC25	2,4	3,724E-04
1687	1685	DNA2	3,2	2,561E-04	1685	LTBP1	2,4	8,882E-04
1688	1686	PRRT2	3,2	7,779E-04	1686	AKAP10	2,4	1,644E-03
1689	1687	ZNF354C	3,2	7,926E-05	1687	LRRC23	2,4	7,848E-03
1690	1688	HOXD11	3,2	2,593E-03	1688	NXT2	2,4	1,483E-05
1691	1689	JMJD4	3,2	1,669E-03	1689	IFIH1	2,4	2,899E-03
1692	1690	FAM212B	3,2	1,502E-05	1690	CTSC	2,4	1,217E-04
1693	1691	NIT1	3,2	5,485E-05	1691	PRDX3	2,4	8,819E-04
1694	1692	JAGN1	3,2	2,861E-05	1692	CEP152	2,4	1,664E-02
1695	1693	RNF149	3,2	4,688E-05	1693	MCM3	2,4	2,877E-04
1696	1694	FIG4	3,2	2,135E-05	1694	DBR1	2,4	9,807E-05
1697	1695	HOXA-AS2	3,2	6,936E-03	1695	PSMD5	2,4	7,726E-05
1698	1696	THNSL1	3,2	2,792E-05	1696	EPC2	2,4	9,053E-05
1699	1697	FBXW9	3,2	2,749E-03	1697	FANCA	2,4	1,784E-03
1700	1698	ZSCAN5A	3,2	7,066E-03	1698	ENDOD1	2,4	6,832E-06
1701	1699	SERPINB6	3,2	2,721E-05	1699	C6orf57	2,4	3,842E-03
1702	1700	CENPN	3,2	2,476E-04	1700	EIF2B5	2,4	2,712E-04
1703	1701	ZNF417	3,2	1,260E-02	1701	KDM2B	2,4	2,014E-03
1704	1702	TRMT11	3,2	1,369E-03	1702	HMGN3	2,4	5,227E-04
1705	1703	LMTK2	3,2	1,898E-06	1703	RMI1	2,4	1,122E-02
1706	1704	SOWAHC	3,2	1,760E-03	1704	CLDN15	2,4	1,338E-04
1707	1705	LRP6	3,2	1,056E-04	1705	CLYBL	2,4	1,214E-02
1708	1706	PLD6	3,2	1,813E-05	1706	GTSE1	2,4	2,655E-04
1709	1707	PHLDB1	3,2	8,676E-03	1707	ZNRF3	2,4	1,295E-04
1710	1708	ATP6V0D1	3,2	2,613E-04	1708	CYTH3	2,4	3,174E-05
1711	1709	AP3M1	3,1	9,517E-06	1709	POLR2H	2,4	1,899E-03
1712	1710	ABHD15	3,1	9,123E-05	1710	DHRS12	2,4	6,596E-03
1713	1711	ITGB3	3,1	5,789E-08	1711	ZNF10	2,4	8,629E-03
1714	1712	INO80C	3,1	1,807E-05	1712	EXOC1	2,4	3,714E-05
1715	1713	ANKRD54	3,1	5,731E-04	1713	TRAFD1	2,4	4,605E-06
1716	1714	VPS33A	3,1	1,352E-05	1714	KIAA1549	2,4	1,674E-03
1717	1715	GBA2	3,1	2,952E-06	1715	CCNYL1	2,4	2,024E-04
1718	1716	ZNF510	3,1	1,219E-03	1716	ADAMTS16	2,4	6,878E-05
1719	1717	MANEA	3,1	9,375E-04	1717	NFE2L1	2,4	6,848E-05
1720	1718	LYRM1	3,1	1,010E-02	1718	SCARB1	2,4	2,792E-04
1721	1719	C19orf55	3,1	5,059E-04	1719	ASB7	2,4	3,550E-05
1722	1720	SIRT5	3,1	1,042E-03	1720	SLC46A1	2,4	9,367E-03
1723	1721	MSL2	3,1	1,466E-04	1721	QTRTD1	2,4	8,456E-03
1724	1722	PRDM5	3,1	1,578E-03	1722	TBCK	2,4	7,367E-04
1725	1723	CMAS	3,1	5,142E-07	1723	WBP4	2,4	1,076E-04
1726	1724	PHKG2	3,1	3,073E-03	1724	CPNE3	2,4	1,882E-04
1727	1725	TCEA3	3,1	1,320E-02	1725	METTL14	2,4	5,708E-04
1728	1726	LIG1	3,1	2,912E-04	1726	NCAPG	2,4	1,015E-04
1729	1727	ECHDC2	3,1	8,951E-05	1727	BST2	2,4	1,366E-03
1730	1728	PHF7	3,1	2,695E-03	1728	MOB3C	2,4	6,636E-03
1731	1729	SCOC	3,1	6,386E-04	1729	PACRGL	2,4	1,278E-02
1732	1730	AMOTL2	3,1	1,120E-02	1730	PIGZ	2,4	5,483E-04
1733	1731	CLDN15	3,1	2,673E-04	1731	ZNF587	2,4	4,612E-03
1734	1732	NAA35	3,1	3,025E-04	1732	HSCB	2,4	1,024E-04
1735	1733	KLHL12	3,1	1,313E-04	1733	ZDHHC14	2,4	2,598E-02
1736	1734	PNISR	3,1	6,993E-03	1734	LCAT	2,4	7,229E-04
1737	1735	ZNF625	3,1	1,483E-03	1735	TXNDC16	2,4	1,227E-04
1738	1736	FEN1	3,1	2,164E-06	1736	VEZT	2,4	4,342E-03
1739	1737	PEX1	3,1	7,558E-04	1737	DHX15	2,4	2,627E-05
1740	1738	VAMP2	3,1	7,187E-05	1738	TUB	2,4	1,783E-02
1741	1739	ZNF641	3,1	4,690E-02	1739	PTHLH	2,4	3,027E-02
1742	1740	ZFP14	3,1	3,200E-02	1740	SLC37A3	2,4	1,062E-03
1743	1741	SESN1	3,1	1,464E-04	1741	CCDC3	2,4	3,811E-03
1744	1742	TBP	3,1	2,524E-04	1742	ARRB2	2,4	5,208E-04
1745	1743	ATP5G1	3,1	1,033E-04	1743	WTAP	2,4	1,059E-04
1746	1744	TYRO3	3,1	5,102E-05	1744	FUCA2	2,4	1,551E-05
1747	1745	WIPF2	3,1	2,443E-03	1745	INTS9	2,4	3,198E-05
1748	1746	MKNK1	3,1	9,353E-05	1746	TRAK1	2,4	1,750E-03
1749	1747	CCDC106	3,1	2,200E-02	1747	TWSG1	2,4	2,629E-04
1750	1748	KIAA1524	3,1	1,561E-04	1748	GMFB	2,4	5,274E-04
1751	1749	MCM4	3,1	2,473E-05	1749	MEF2A	2,4	1,672E-02
1752	1750	ZNF619	3,1	1,364E-02	1750	SHOX2	2,4	2,096E-03
1753	1751	C17orf80	3,1	1,968E-03	1751	KIF13A	2,4	3,819E-03
1754	1752	MRM1	3,1	5,279E-05	1752	APPBP2	2,4	2,880E-06
1755	1753	B3GALNT1	3,1	6,364E-04	1753	ZNF691	2,4	6,538E-04
1756	1754	NAPEPLD	3,1	2,016E-02	1754	KIF13B	2,4	8,377E-04
1757	1755	SKA3	3,1	2,724E-04	1755	TMEM53	2,4	5,566E-03
1758	1756	KPNA2	3,1	3,093E-03	1756	TSPAN15	2,4	2,820E-03
1759	1757	EVI5	3,1	2,285E-04	1757	ATG10	2,4	2,503E-03
1760	1758	C2orf42	3,1	9,111E-05	1758	CPT1A	2,4	3,837E-05
1761	1759	RAD51	3,1	7,280E-04	1759	LMAN2L	2,4	2,117E-05
1762	1760	PCGF5	3,1	2,435E-04	1760	GRK4	2,4	7,588E-03
1763	1761	CALCOCO1	3,1	3,919E-05	1761	TBCCD1	2,4	4,033E-04
1764	1762	MED11	3,1	3,054E-04	1762	ARFGEF1	2,4	6,833E-05
1765	1763	SCD5	3,1	2,470E-05	1763	NCOA7	2,4	7,111E-03
1766	1764	MTM1	3,1	2,824E-03	1764	FAM120B	2,4	9,542E-07
1767	1765	FGF1	3,1	2,799E-03	1765	FN3K	2,3	1,498E-03
1768	1766	POLR3K	3,1	9,677E-05	1766	C2orf44	2,3	1,346E-03
1769	1767	PRKG1	3,1	1,236E-04	1767	FAM200A	2,3	2,457E-03
1770	1768	SNX7	3,1	1,860E-04	1768	XRCC2	2,3	2,960E-03
1771	1769	SCRN2	3,1	2,977E-04	1769	GTF2B	2,3	5,197E-03
1772	1770	SCYL3	3,1	4,631E-03	1770	ZNF318	2,3	3,237E-05
1773	1771	NUCKS1	3,1	5,358E-05	1771	KIF15	2,3	9,839E-04
1774	1772	SV2A	3,1	1,692E-06	1772	IL6ST	2,3	7,337E-03
1775	1773	CEP152	3,1	5,197E-04	1773	SFT2D1	2,3	1,500E-02
1776	1774	PPP2R3B	3,1	3,854E-02	1774	HMGA2	2,3	2,706E-03
1777	1775	FXN	3,1	1,282E-02	1775	TMEM9B	2,3	9,737E-04
1778	1776	ZBTB5	3,1	9,766E-06	1776	RFWD3	2,3	1,196E-04
1779	1777	C12orf4	3,1	2,794E-05	1777	RFX5	2,3	1,039E-02
1780	1778	NCAPH	3,1	1,109E-04	1778	TMED5	2,3	4,410E-04
1781	1779	PAIP2	3,1	1,102E-05	1779	TCTN1	2,3	7,406E-04
1782	1780	FAM105A	3,1	2,428E-03	1780	TRIM37	2,3	2,629E-03
1783	1781	VCP	3,1	7,130E-05	1781	ZNF789	2,3	1,785E-03
1784	1782	DOLK	3,1	3,001E-05	1782	USP6NL	2,3	1,260E-02
1785	1783	DHRS1	3,1	1,007E-03	1783	RNF146	2,3	3,723E-02
1786	1784	TNIK	3,1	2,684E-05	1784	INSIG2	2,3	2,108E-03
1787	1785	ALDH6A1	3,1	2,792E-04	1785	PTPN9	2,3	2,186E-04
1788	1786	FAT3	3,1	1,596E-03	1786	PRDM11	2,3	3,822E-03
1789	1787	KIAA0753	3,1	1,076E-03	1787	PKN3	2,3	1,367E-03
1790	1788	EME1	3,1	3,150E-04	1788	PSKH1	2,3	4,673E-04
1791	1789	KIF17	3,1	1,600E-03	1789	POLR3K	2,3	5,056E-04
1792	1790	ZNF513	3,0	6,263E-03	1790	PHTF1	2,3	4,056E-04
1793	1791	ZEB1-AS1	3,0	7,007E-03	1791	C2orf42	2,3	9,681E-04
1794	1792	EGR1	3,0	1,287E-05	1792	NUPL1	2,3	1,560E-03
1795	1793	PTCD2	3,0	2,074E-02	1793	TMEM87B	2,3	3,657E-05
1796	1794	MRPL50	3,0	1,951E-04	1794	ARHGEF7	2,3	1,319E-04
1797	1795	LMBRD1	3,0	5,474E-04	1795	ACACB	2,3	3,193E-03
1798	1796	PAPLN	3,0	8,128E-03	1796	CBFB	2,3	4,998E-04
1799	1797	UBN2	3,0	1,202E-03	1797	DEK	2,3	1,735E-05
1800	1798	GLO1	3,0	1,516E-05	1798	SMPD1	2,3	1,447E-03
1801	1799	ZNF76	3,0	3,280E-04	1799	FAR2	2,3	1,667E-03
1802	1800	GTF2H4	3,0	1,155E-02	1800	ADAM9	2,3	2,237E-05

	A	B	C	D	E	F	G	H	I	J	K	L	M	N	O	P	Q	R	S	T	U	V	W	X
1803	1801	ZNF571	3,0	1,987E-03					1801	TAF13	2,3	4,367E-04												
1804	1802	DHX16	3,0	6,806E-05					1802	FUCA1	2,3	4,183E-05												
1805	1803	POLR2J4	3,0	3,049E-03					1803	FDXR	2,3	2,157E-02												
1806	1804	BCDIN3D	3,0	2,717E-04					1804	ETNK2	2,3	7,083E-05												
1807	1805	SLC31A2	3,0	4,519E-05					1805	GFM1	2,3	5,742E-04												
1808	1806	NUBP1	3,0	3,683E-05					1806	NKX6-1	2,3	6,487E-03												
1809	1807	ZER1	3,0	1,000E-04					1807	SLC45A1	2,3	3,685E-03												
1810	1808	NDUFA5	3,0	3,136E-02					1808	MCM8	2,3	4,886E-04												
1811	1809	RTN4IP1	3,0	5,303E-04					1809	TAF1A	2,3	2,196E-03												
1812	1810	ATPAF1	3,0	3,497E-04					1810	FANCE	2,3	1,308E-03												
1813	1811	RANGAP1	3,0	8,586E-05					1811	SLC25A19	2,3	8,908E-04												
1814	1812	RNF219	3,0	2,768E-04					1812	AHI1	2,3	1,005E-03												
1815	1813	KCTD5	3,0	6,748E-05					1813	MRPL42	2,3	7,707E-03												
1816	1814	CASP6	3,0	4,189E-06					1814	ISYNA1	2,3	2,738E-02												
1817	1815	ZNF468	3,0	2,282E-06					1815	ATP7A	2,3	1,636E-04												
1818	1816	SLC35B4	3,0	4,750E-05					1816	CEPT1	2,3	1,275E-04												
1819	1817	GJA1	3,0	3,735E-05					1817	RANGAP1	2,3	9,115E-05												
1820	1818	PIGF	3,0	4,133E-04					1818	PHF23	2,3	1,823E-02												
1821	1819	GSTA4	3,0	1,759E-03					1819	ZUFSP	2,3	3,664E-03												
1822	1820	CCNDBP1	3,0	1,586E-03					1820	LINC00467	2,3	1,118E-04												
1823	1821	NUP205	3,0	1,666E-03					1821	HLA-E	2,3	6,546E-04												
1824	1822	TAF11	3,0	1,978E-04					1822	LBR	2,3	1,541E-04												
1825	1823	STX2	3,0	8,289E-06					1823	NASP	2,3	6,555E-06												
1826	1824	EXD3	3,0	1,587E-02					1824	CNBP	2,3	2,135E-03												
1827	1825	PEX19	3,0	3,038E-03					1825	ARL5A	2,3	1,693E-04												
1828	1826	MAP3K1	3,0	2,184E-03					1826	ZNF79	2,3	3,054E-03												
1829	1827	BRWD1	3,0	6,442E-05					1827	ZMYND8	2,3	9,138E-05												
1830	1828	NCEH1	3,0	1,041E-03					1828	DNASE1L1	2,3	1,618E-02												
1831	1829	COPS8	3,0	1,924E-03					1829	C10orf32	2,3	2,816E-04												
1832	1830	DCTN5	3,0	3,894E-02					1830	TSPYL4	2,3	2,027E-05												
1833	1831	CHML	3,0	4,719E-04					1831	TMEM101	2,3	1,160E-05												
1834	1832	DOCK4	3,0	4,173E-02					1832	AKAP13	2,3	4,614E-02												
1835	1833	PLEKHA4	3,0	9,500E-03					1833	AP1B1	2,3	1,145E-04												
1836	1834	TUBB4B	3,0	3,759E-04					1834	PATZ1	2,3	2,667E-05												
1837	1835	EXOSC6	3,0	2,621E-04					1835	LAPTM4B	2,3	4,130E-06												
1838	1836	IPO9	3,0	1,117E-05					1836	UCHL1	2,3	9,157E-03												
1839	1837	ARF3	3,0	3,349E-05					1837	PPAT	2,3	6,868E-04												
1840	1838	RFXAP	3,0	5,680E-05					1838	RAB27B	2,3	1,863E-02												
1841	1839	CBX7	3,0	1,518E-04					1839	C11orf71	2,3	4,579E-03												
1842	1840	OGDH	3,0	1,306E-04					1840	ZNF629	2,3	1,750E-04												
1843	1841	TOR3A	3,0	2,325E-04					1841	FAM64A	2,3	8,884E-04												
1844	1842	CENPO	3,0	5,020E-04					1842	FOXC1	2,3	7,802E-05												
1845	1843	PITPNM3	3,0	1,107E-03					1843	NGFRAP1	2,3	1,836E-03												
1846	1844	ZNF101	3,0	8,635E-03					1844	PARPBP	2,3	3,881E-04												
1847	1845	ZNF319	3,0	1,382E-02					1845	PNRC2	2,3	2,108E-02												
1848	1846	AGBL5	3,0	1,160E-05					1846	HJURP	2,3	1,256E-04												
1849	1847	ARL13B	3,0	4,536E-04					1847	EPS15	2,3	6,119E-04												
1850	1848	DDX47	3,0	9,706E-05					1848	ELK3	2,3	2,069E-03												
1851	1849	LEMD3	3,0	1,069E-03					1849	FBXL4	2,3	1,022E-04												
1852	1850	MIS18A	3,0	1,731E-05					1850	CPT2	2,3	2,556E-06												
1853	1851	DNTTIP2	3,0	2,082E-03					1851	PGM2L1	2,3	2,791E-06												
1854	1852	RNF167	3,0	1,113E-04					1852	JMY	2,3	2,960E-03												
1855	1853	TMEM14B	3,0	8,725E-06					1853	CDC40	2,3	1,196E-04												
1856	1854	MDC1	3,0	7,768E-04					1854	DDX5	2,3	4,531E-04												
1857	1855	BCL7A	3,0	1,391E-03					1855	KIF22	2,3	7,794E-05												
1858	1856	RPA1	3,0	2,981E-04					1856	DCAF12	2,3	7,844E-06												
1859	1857	HELB	3,0	5,200E-03					1857	BARD1	2,3	1,721E-02												
1860	1858	FAM206A	3,0	2,022E-05					1858	ZNF689	2,3	2,932E-03												
1861	1859	SLC7A6OS	3,0	1,734E-05					1859	NBEA	2,3	5,666E-04												
1862	1860	SPG7	3,0	1,763E-04					1860	SLBP	2,3	4,837E-03												
1863	1861	SETD4	3,0	3,138E-03					1861	KIF16B	2,3	2,941E-04												
1864	1862	ATP5G2	3,0	1,632E-03					1862	GGA2	2,3	6,251E-05												
1865	1863	ATP6V1G1	3,0	5,306E-06					1863	S100A4	2,3	1,256E-03												
1866	1864	WTAP	3,0	1,956E-04					1864	GAS2L3	2,3	3,627E-04												
1867	1865	FAM167A	2,9	1,133E-05					1865	ARHGAP19	2,3	5,184E-04												
1868	1866	TUFM	2,9	1,398E-04					1866	ZCCHC7	2,3	1,515E-04												
1869	1867	CEP70	2,9	7,592E-04					1867	APLP2	2,3	5,833E-06												
1870	1868	BIRC5	2,9	8,011E-04					1868	PRR11	2,3	2,755E-04												
1871	1869	SCRN1	2,9	1,016E-03					1869	SUPT6H	2,3	9,782E-05												
1872	1870	MORC4	2,9	3,901E-03					1870	SNAPC3	2,3	2,550E-03												
1873	1871	SLC35D1	2,9	2,799E-05					1871	RWDD2A	2,3	8,411E-04												
1874	1872	CES2	2,9	3,334E-04					1872	ZNF891	2,3	2,324E-02												
1875	1873	AKAP9	2,9	1,432E-03					1873	BACH1	2,3	1,304E-04												
1876	1874	MAP6D1	2,9	1,256E-03					1874	LTBP3	2,3	1,243E-02												
1877	1875	FAM63A	2,9	2,212E-03					1875	CAPN7	2,3	1,032E-02												
1878	1876	RBBP7	2,9	6,134E-05					1876	HMMR	2,3	4,779E-04												
1879	1877	TMED8	2,9	3,569E-04					1877	RAB2B	2,3	3,353E-04												
1880	1878	DCP1B	2,9	7,171E-06					1878	PAFAH1B2	2,3	1,408E-07												
1881	1879	ACADM	2,9	8,889E-05					1879	PDIA4	2,3	3,873E-06												
1882	1880	POMT1	2,9	3,458E-04					1880	LDHB	2,3	1,709E-05												
1883	1881	SART3	2,9	4,802E-06					1881	NAGLU	2,3	2,607E-03												
1884	1882	PRKAR1B	2,9	1,500E-03					1882	SNAP23	2,3	2,902E-02												
1885	1883	NASP	2,9	1,757E-05					1883	KIFC1	2,3	1,810E-06												
1886	1884	C11orf63	2,9	1,989E-05					1884	MDFIC	2,3	3,285E-03												
1887	1885	SEH1L	2,9	2,300E-04					1885	ETV6	2,3	1,753E-04												
1888	1886	TIAM1	2,9	4,385E-05					1886	FEM1B	2,3	4,801E-06												
1889	1887	TLR4	2,9	6,010E-04					1887	CBR4	2,3	1,235E-04												
1890	1888	SLC2A8	2,9	2,216E-03					1888	CC2D2A	2,3	2,649E-04												
1891	1889	HNRNPU-AS1	2,9	2,962E-03					1889	POC5	2,3	3,741E-04												
1892	1890	PPHLN1	2,9	1,609E-04					1890	PHACTR4	2,3	2,047E-05												
1893	1891	C10orf32	2,9	6,551E-05					1891	PIAS1	2,3	3,248E-07												
1894	1892	GPR173	2,9	2,490E-05					1892	ZFYVE16	2,3	3,007E-03												
1895	1893	ANKFY1	2,9	1,855E-05					1893	ZBTB43	2,3	1,953E-02												
1896	1894	SOD1	2,9	2,742E-03					1894	MGAT5	2,3	7,618E-04												
1897	1895	SLX4	2,9	6,537E-04					1895	OMA1	2,3	7,352E-04												
1898	1896	CDCA5	2,9	1,641E-05					1896	IKBKB	2,3	1,621E-04												
1899	1897	KAT2B	2,9	2,638E-04					1897	TP53TG1	2,3	3,301E-03												
1900	1898	IVD	2,9	1,742E-04					1898	KIAA2026	2,3	1,387E-04												
1901	1899	KLHL11	2,9	8,341E-04					1899	RNF38	2,3	1,408E-03												
1902	1900	RASEF	2,9	3,003E-03					1900	PLD6	2,3	3,417E-03												
1903	1901	PCNA	2,9	4,873E-04					1901	PRKCE	2,3	1,872E-03												
1904	1902	FGFR1OP	2,9	1,620E-03					1902	TBX19	2,3	1,964E-02												
1905	1903	NR6A1	2,9	1,893E-03					1903	CIAPIN1	2,3	2,218E-03												
1906	1904	CCNB1	2,9	6,586E-05					1904	IZUMO4	2,3	3,376E-02												
1907	1905	ZNF708	2,9	8,783E-03					1905	ZWINT	2,3	1,091E-03												
1908	1906	UFL1	2,9	1,711E-04					1906	HPS3	2,3	3,041E-04												
1909	1907	CHMP5	2,9	3,144E-05					1907	TMEM186	2,3	5,161E-04												
1910	1908	C18orf8	2,9	1,047E-05					1908	PBX2	2,3	4,013E-04												
1911	1909	SPIN4	2,9	4,435E-04					1909	PAPOLG	2,3	9,169E-03												
1912	1910	UBE2G2	2,9	1,242E-05					1910	LRCH3	2,3	3,049E-03												
1913	1911	UBR7	2,9	3,813E-03					1911	KLHL9	2,3	4,805E-05												
1914	1912	SCCPDH	2,9	1,452E-06					1912	NPHP3	2,3	2,955E-05												
1915	1913	SLC8A1	2,9	7,599E-03					1913	BCL10	2,3	3,707E-04												
1916	1914	VEZT	2,9	1,452E-03					1914	TP53BP2	2,3	2,184E-03												
1917	1915	IFNAR1	2,9	3,344E-04					1915	VPS13A	2,3	9,418E-03												
1918	1916	ERLIN2	2,9	5,379E-05					1916	APOBEC3C	2,3	1,406E-05												
1919	1917	FAM111A	2,9	9,615E-06					1917	TP73-AS1	2,3	3,734E-03												
1920	1918	SCAF4	2,9	3,197E-04					1918	C12orf43	2,3	2,078E-02												
1921	1919	WDR73	2,9	1,373E-05					1919	NDC80	2,3	5,206E-04												
1922	1920	ZFAND4	2,9	3,737E-03					1920	FHL1	2,3	1,281E-04												
1923	1921	CHAF1A	2,9	1,766E-04					1921	C5orf22	2,3	1,749E-04												
1924	1922	KLHDC10	2,9	3,066E-04					1922	N4BP1	2,3	8,086E-05												
1925	1923	ABCB9	2,9	6,740E-03					1923	BAZ2B	2,3	6,715E-03												
1926	1924	TMEM242	2,9	2,150E-05					1924	IMPACT	2,3	2,240E-03												
1927	1925	ERCC4	2,9	5,774E-04					1925	ARHGAP20	2,3	3,093E-04												
1928	1926	HPS3	2,9	6,837E-04					1926	UNK	2,3	3,695E-04												
1929	1927	PYCR2	2,9	4,179E-05					1927	GSG2	2,3	1,359E-02												
1930	1928	ANP32B	2,9	7,453E-06					1928	GGH	2,3	7,880E-05												
1931	1929	NARFL	2,9	3,326E-04					1929	BRCA2	2,3	3,603E-02												
1932	1930	ALDOC	2,9	1,113E-03					1930	SOCS7	2,2	2,446E-03												
1933	1931	LYSMD1	2,9	1,904E-04					1931	GLO1	2,2	3,976E-05												
1934	1932	CNNM3	2,9	1,179E-05					1932	DIRC2	2,2	2,511E-04												
1935	1933	KIRREL3	2,9	6,966E-04					1933	CYB5RL	2,2	2,170E-03												
1936	1934	RGL1	2,9	2,874E-03					1934	PTPRJ	2,2	1,394E-04												
1937	1935	BAG1	2,9	9,763E-04					1935	VAMP5	2,2	3,106E-04												
1938	1936	CCDC3	2,9	3,730E-04					1936	C17orf103	2,2	5,810E-04												
1939	1937	URB2	2,9	1,503E-04					1937	CDKN1A	2,2	3,065E-04												
1940	1938	GLIS2	2,9	1,008E-02					1938	ABCF3	2,2	5,190E-03												
1941	1939	USP25	2,9	8,952E-04					1939	RANBP9	2,2	2,105E-06												
1942	1940	DUSP28	2,9	7,675E-03					1940	OSBPL11	2,2	5,121E-05												
1943	1941	ULBP3	2,9	2,185E-03					1941	SPAG1	2,2	4,848E-02												
1944	1942	ITSN1	2,9	6,293E-04					1942	EXOC2	2,2	1,338E-04												
1945	1943	CARKD	2,9	6,209E-03					1943	PBRM1	2,2	9,475E-04												
1946	1944	WNT3	2,9	4,687E-04					1944	BACE1	2,2	1,680E-05												
1947	1945	RRAGB	2,9	9,530E-06					1945	C2CD3	2,2	8,241E-03												
1948	1946	HIRIP3	2,9	2,665E-04					1946	LUC7L	2,2	4,740E-03												
1949	1947	EPB41	2,9	2,351E-04					1947	DPY19L3	2,2	1,355E-02												
1950	1948	IFIT5	2,9	4,571E-03					1948	SMARCC2	2,2	1,117E-04												
1951	1949	KIF2A	2,9	8,055E-05					1949	C3orf17	2,2	2,712E-03												
1952	1950	ABCD3	2,9	1,280E-02					1950	DNAJB11	2,2	3,176E-03												
1953	1951	ELOVL5	2,9	5,380E-06					1951	ANKRD40	2,2	8,903E-06												
1954	1952	THAP10	2,9	2,082E-03					1952	SSH1	2,2	9,616E-03												
1955	1953	ACADVL	2,9	8,006E-05					1953	SERINC3	2,2	1,050E-03												
1956	1954	GTF3C1	2,9	4,363E-06					1954	TIMELESS	2,2	1,429E-05												
1957	1955	ASB8	2,9	1,322E-05					1955	TMEM242	2,2	1,891E-03												
1958	1956	TP53BP2	2,9	3,079E-04					1956	EXOC7	2,2	8,648E-03												
1959	1957	NEK4	2,9	9,016E-04					1957	EXOSC6	2,2	1,715E-03												
1960	1958	SNRNP35	2,9	2,796E-05					1958	WBSCR27	2,2	4,505E-03												
1961	1959	MCM8	2,9	1,691E-05					1959	DGKA	2,2	2,038E-03												
1962	1960	DDX5	2,8	3,936E-06					1960	OCIAD1	2,2	4,276E-04												
1963	1961	PPP1CC	2,8	1,546E-03					1961	PARN	2,2	5,831E-05												
1964	1962	CREB5	2,8	5,937E-03					1962	P2RX4	2,2	2,285E-04												
1965	1963	MTMR1	2,8	2,592E-04					1963	EEF1E1	2,2	4,759E-04												
1966	1964	AGK	2,8	5,142E-05					1964	CNOT1	2,2	1,116E-04												
1967	1965	WRNIP1	2,8	1,026E-06					1965	CD2BP2	2,2	9,085E-03												
1968	1966	PSMB8	2,8	3,286E-06					1966	UBAC2	2,2	4,209E-02												
1969	1967	CITED4	2,8	1,967E-02					1967	IGF2BP2	2,2	1,626E-03												
1970	1968	SP110	2,8	1,400E-04					1968	TULP4	2,2	4,322E-05												
1971	1969	NFATC1	2,8	1,674E-02					1969	CASP9	2,2	9,695E-04												
1972	1970	DCAF8	2,8	6,607E-05					1970	GNPTAB	2,2	4,490E-05												
1973	1971	PHF2	2,8	1,394E-04					1971	SENP2	2,2	3,857E-02												
1974	1972	ZNF189	2,8	3,816E-03					1972	CASP1	2,2	3,919E-03												
1975	1973	HOXB7	2,8	5,304E-04					1973	FBXW2	2,2	4,711E-02												
1976	1974	NDUFA8	2,8	3,780E-06					1974	NR1D2	2,2	1,547E-06												
1977	1975	CASP8AP2	2,8	4,501E-04					1975	WDR47	2,2	8,367E-05												
1978	1976	TERF2	2,8	3,192E-05					1976	DNAJB14	2,2	6,035E-04												
1979	1977	RAPGEF1	2,8	2,113E-04					1977	ZBTB40	2,2	2,408E-05												
1980	1978	ANK1	2,8	2,937E-03					1978	HIST1H2BK	2,2	1,638E-03												
1981	1979	LMO4	2,8	1,953E-03					1979	MUT	2,2	2,949E-05												
1982	1980	SPICE1	2,8	4,402E-03					1980	SLC10A7	2,2	6,618E-03												
1983	1981	WDR5	2,8	1,597E-04					1981	CCDC106	2,2	1,150E-02												
1984	1982	ZC3H10	2,8	7,263E-03					1982	SENP8	2,2	9,054E-03												
1985	1983	MRPL39	2,8	6,026E-04					1983	GOT2	2,2	4,200E-04												
1986	1984	KITLG	2,8	1,425E-04					1984	TRIM21	2,2	2,833E-03												
1987	1985	WWC3	2,8	5,693E-05					1985	SMARCA2	2,2	3,944E-03												
1988	1986	AGPAT4	2,8	1,134E-04					1986	HOXC6	2,2	1,584E-02												
1989	1987	TTYH2	2,8	1,281E-03					1987	ZNF738	2,2	1,458E-02												
1990	1988	THAP2	2,8	9,002E-04					1988	CHAMP1	2,2	1,120E-03												
1991	1989	ADNP2	2,8	1,501E-05					1989	IKZF4	2,2	2,570E-02												
1992	1990	HMGA1	2,8	4,236E-02					1990	SLC35D2	2,2	1,169E-05												
1993	1991	ZNF480	2,8	1,500E-04					1991	LITAF	2,2	7,018E-04												
1994	1992	CDAN1	2,8	2,136E-03					1992	HOXC11	2,2	3,556E-02												
1995	1993	TMEM19	2,8	2,007E-03					1993	ZNF649	2,2	4,890E-02												
1996	1994	IFT122	2,8	2,624E-06					1994	RAP2A	2,2	1,781E-05												
1997	1995	RSRC2	2,8	1,022E-04					1995	LINC00526	2,2	1,112E-02												
1998	1996	TTLL5	2,8	1,040E-05					1996	HELZ	2,2	2,240E-03												
1999	1997	SMARCC2	2,8	1,955E-04					1997	CRLF3	2,2	1,105E-04												
2000	1998	APEX2	2,8	3,695E-06					1998	SNIP1	2,2	1,434E-03												
2001	1999	PTGR2	2,8	9,852E-03					1999	PRIM1	2,2	3,963E-03												
2002	2000	ZNF768	2,8	2,422E-03					2000	CLCN2	2,2	1,098E-03												
2003	2001	REPS1	2,8	3,939E-04					2001	PI4KB	2,2	6,947E-03												
2004	2002	ABHD10	2,8	7,177E-03					2002	CCDC24	2,2	2,544E-03												

	A	B	C	D	E	F	G	H	I	J	K	L	M	N	O	P	Q	R	S	T	U	V	W	X
2005	2003	GLYR1	2,8	3,384E-07					2003	PRKCI	2,2	3,822E-04												
2006	2004	POLE3	2,8	3,913E-06					2004	MAEA	2,2	8,265E-03												
2007	2005	RBM18	2,8	2,870E-05					2005	SLC25A25	2,2	4,182E-07												
2008	2006	TMEM41A	2,8	3,567E-04					2006	RFC5	2,2	2,531E-03												
2009	2007	ANKS1A	2,8	1,314E-04					2007	NMT1	2,2	1,612E-04												
2010	2008	POLH	2,8	4,341E-06					2008	CSRNP2	2,2	3,337E-04												
2011	2009	HLA-B	2,8	7,464E-04					2009	GSTT1	2,2	1,694E-04												
2012	2010	C4orf27	2,8	2,034E-04					2010	PHIP	2,2	9,232E-04												
2013	2011	ZNF254	2,8	1,827E-03					2011	LYRM2	2,2	1,703E-02												
2014	2012	RUNDC1	2,8	7,952E-06					2012	IFT88	2,2	1,253E-02												
2015	2013	CRY1	2,8	1,402E-04					2013	MIS18BP1	2,2	6,637E-04												
2016	2014	SUSD1	2,8	9,887E-04					2014	RPL22L1	2,2	3,048E-03												
2017	2015	GCLM	2,8	1,267E-03					2015	YIPF4	2,2	1,673E-04												
2018	2016	SLC11A2	2,8	4,050E-05					2016	BCAS3	2,2	5,875E-04												
2019	2017	PON2	2,8	5,698E-04					2017	TMEM192	2,2	2,358E-03												
2020	2018	PI4K2B	2,8	4,780E-04					2018	FASTKD5	2,2	4,860E-04												
2021	2019	INTS2	2,8	1,014E-03					2019	ZFP14	2,2	4,668E-02												
2022	2020	DCAF16	2,8	3,524E-04					2020	SMARCA1	2,2	1,067E-05												
2023	2021	LARP4	2,8	1,134E-03					2021	VGLL4	2,2	1,617E-04												
2024	2022	PIGP	2,8	1,160E-02					2022	APBB1	2,2	7,494E-04												
2025	2023	MPP5	2,8	1,940E-04					2023	VMA21	2,2	6,865E-05												
2026	2024	MAF	2,8	6,825E-04					2024	UBE2N	2,2	5,878E-03												
2027	2025	PPIL1	2,8	9,244E-05					2025	MED1	2,2	2,388E-04												
2028	2026	BVES	2,8	2,095E-06					2026	ADAT1	2,2	4,185E-04												
2029	2027	DYNC2H1	2,8	1,064E-03					2027	FBXO34	2,2	2,081E-04												
2030	2028	ZNF829	2,8	1,124E-04					2028	WDR90	2,2	1,858E-02												
2031	2029	PPOX	2,8	1,638E-04					2029	BCORL1	2,2	1,461E-02												
2032	2030	DHRS7B	2,8	2,731E-03					2030	TMTC3	2,2	8,171E-05												
2033	2031	METTL13	2,8	2,746E-05					2031	CHSY1	2,2	1,932E-05												
2034	2032	CDK2	2,8	3,525E-04					2032	GTF2I	2,2	1,166E-03												
2035	2033	TAGLN2	2,8	6,810E-06					2033	POLDIP3	2,2	1,245E-02												
2036	2034	DHTKD1	2,8	4,040E-04					2034	EIF4G3	2,2	1,372E-04												
2037	2035	TP73-AS1	2,8	1,451E-02					2035	H2AFX	2,2	5,045E-03												
2038	2036	MLEC	2,8	1,941E-02					2036	RANBP6	2,2	4,150E-04												
2039	2037	SMYD4	2,8	2,208E-03					2037	ACADSB	2,2	2,427E-02												
2040	2038	GFOD1	2,8	7,259E-03					2038	SERP2	2,2	4,986E-03												
2041	2039	NQO1	2,8	9,780E-04					2039	ZNF680	2,2	3,802E-04												
2042	2040	HDHD1	2,8	9,094E-04					2040	TMEM184B	2,2	5,036E-04												
2043	2041	TULP3	2,8	8,122E-04					2041	TBC1D14	2,2	1,638E-04												
2044	2042	GAPVD1	2,8	1,339E-05					2042	NUFIP2	2,2	9,641E-05												
2045	2043	BUD13	2,8	1,228E-03					2043	LIPA	2,2	4,352E-06												
2046	2044	LIMK2	2,8	7,936E-05					2044	STOM	2,2	1,323E-04												
2047	2045	WBP11	2,8	1,169E-05					2045	ZNF708	2,2	6,695E-03												
2048	2046	TTC5	2,8	1,173E-04					2046	C1RL	2,2	5,724E-04												
2049	2047	TBK1	2,8	2,866E-04					2047	PREPL	2,2	9,551E-05												
2050	2048	TBCK	2,8	3,871E-04					2048	C11orf54	2,2	6,309E-06												
2051	2049	SLC35A3	2,8	3,961E-03					2049	FAM102B	2,2	2,333E-04												
2052	2050	PCYOX1L	2,8	5,964E-04					2050	ZBTB39	2,2	9,412E-04												
2053	2051	CDC25A	2,8	1,135E-03					2051	TRAF1	2,2	3,384E-04												
2054	2052	GEM	2,8	1,227E-04					2052	RYK	2,2	1,333E-05												
2055	2053	TKT	2,8	7,155E-03					2053	CDKAL1	2,2	4,181E-05												
2056	2054	SCAF8	2,8	6,846E-05					2054	ENTPD4	2,2	6,521E-04												
2057	2055	MID1	2,8	7,224E-04					2055	INCENP	2,2	4,826E-03												
2058	2056	ZNF3	2,8	9,965E-04					2056	CMBL	2,2	1,348E-03												
2059	2057	STIL	2,8	8,066E-04					2057	LSG1	2,2	1,450E-03												
2060	2058	TXNRD2	2,8	2,975E-03					2058	SCYL2	2,2	3,102E-04												
2061	2059	CCNT1	2,8	2,161E-04					2059	PRR3	2,2	1,127E-04												
2062	2060	CCBL2	2,8	2,949E-05					2060	MRPS18B	2,2	6,707E-04												
2063	2061	PARPBP	2,8	1,242E-05					2061	USPL1	2,2	7,187E-04												
2064	2062	C10orf35	2,8	2,696E-04					2062	FAM73A	2,2	1,777E-05												
2065	2063	FBXW2	2,8	2,341E-02					2063	GUF1	2,2	3,948E-03												
2066	2064	C22orf29	2,8	2,216E-05					2064	SLC39A9	2,2	7,322E-03												
2067	2065	HKR1	2,8	3,849E-02					2065	YEATS2	2,2	1,920E-03												
2068	2066	ARNTL2	2,7	7,213E-04					2066	LRP12	2,2	5,209E-04												
2069	2067	SP4	2,7	3,525E-04					2067	IFT74	2,2	1,219E-02												
2070	2068	RNF146	2,7	1,096E-02					2068	RBPJ	2,2	5,833E-05												
2071	2069	PRPF4B	2,7	1,389E-03					2069	RRN3	2,2	7,224E-03												
2072	2070	ATP6V1E2	2,7	7,779E-04					2070	C12orf4	2,2	1,392E-04												
2073	2071	HACE1	2,7	3,179E-04					2071	SRSF2	2,2	2,525E-03												
2074	2072	PCBD1	2,7	7,405E-05					2072	PAPSS1	2,2	4,022E-02												
2075	2073	C12orf43	2,7	3,174E-02					2073	EFNB3	2,2	4,291E-04												
2076	2074	CNIH3	2,7	8,420E-04					2074	SLC25A29	2,2	1,511E-02												
2077	2075	NUDT2	2,7	4,116E-05					2075	C14orf159	2,2	5,410E-04												
2078	2076	FTO	2,7	2,880E-05					2076	PLSCR4	2,2	1,169E-02												
2079	2077	PRPS2	2,7	1,614E-03					2077	PPM1D	2,2	2,111E-03												
2080	2078	KDM5A	2,7	2,098E-04					2078	ZFAND4	2,2	2,768E-02												
2081	2079	MECOM	2,7	6,920E-05					2079	GPCPD1	2,2	2,362E-04												
2082	2080	TATDN3	2,7	2,131E-04					2080	LEMD2	2,2	4,175E-04												
2083	2081	CENPL	2,7	1,569E-04					2081	FDXACB1	2,2	2,086E-02												
2084	2082	TSPY26P	2,7	3,383E-02					2082	POLR2C	2,2	1,038E-04												
2085	2083	FIGNL1	2,7	4,080E-02					2083	SUDS3	2,2	1,042E-04												
2086	2084	PIGO	2,7	5,210E-04					2084	NET1	2,2	1,435E-04												
2087	2085	PRADC1	2,7	6,149E-05					2085	GTF2F2	2,2	2,889E-05												
2088	2086	GLE1	2,7	2,113E-05					2086	NPL	2,2	2,092E-02												
2089	2087	GPR107	2,7	6,854E-06					2087	SASH1	2,2	6,168E-05												
2090	2088	ZDHHC14	2,7	1,021E-03					2088	ATXN1	2,2	7,033E-06												
2091	2089	SEC14L1	2,7	9,839E-05					2089	OSR1	2,2	7,618E-03												
2092	2090	CDK5RAP3	2,7	2,413E-03					2090	EAF1	2,2	5,185E-04												
2093	2091	ACAD10	2,7	1,513E-05					2091	IDH1	2,2	3,526E-03												
2094	2092	OSR2	2,7	4,539E-02					2092	ATM	2,2	1,161E-02												
2095	2093	WDR92	2,7	6,868E-05					2093	PDPK1	2,2	1,919E-03												
2096	2094	ETNK2	2,7	3,689E-06					2094	CCRN4L	2,2	1,941E-02												
2097	2095	ARPC5L	2,7	8,902E-05					2095	LMBRD2	2,2	1,572E-03												
2098	2096	TRNAU1AP	2,7	3,262E-04					2096	ZNF740	2,2	3,893E-04												
2099	2097	CUL9	2,7	6,886E-04					2097	RPA1	2,2	3,558E-04												
2100	2098	SEPP1	2,7	2,238E-03					2098	NOP14	2,2	1,153E-03												
2101	2099	FAM122A	2,7	2,963E-04					2099	TRO	2,2	6,000E-03												
2102	2100	HNRNPA2B1	2,7	1,783E-05					2100	CCDC15	2,2	7,197E-03												
2103	2101	VPS13A	2,7	1,727E-04					2101	IQCB1	2,2	2,953E-05												
2104	2102	RERE	2,7	5,633E-04					2102	ZIK1	2,2	2,762E-03												
2105	2103	ERP29	2,7	2,247E-06					2103	AASDHPPT	2,2	1,537E-04												
2106	2104	NBEAL1	2,7	1,488E-04					2104	KIAA0753	2,2	4,576E-03												
2107	2105	ZC3HAV1	2,7	1,294E-04					2105	FRS2	2,2	2,351E-03												
2108	2106	ZUFSP	2,7	3,684E-04					2106	CEND1	2,2	1,089E-02												
2109	2107	FAM8A1	2,7	2,467E-05					2107	ZNF546	2,2	1,466E-02												
2110	2108	SWI5	2,7	4,071E-05					2108	KBTBD3	2,2	1,084E-02												
2111	2109	NUP93	2,7	1,458E-04					2109	ARF3	2,2	4,559E-04												
2112	2110	POT1	2,7	1,353E-05					2110	L3MBTL1	2,2	4,284E-02												
2113	2111	TYW3	2,7	7,356E-05					2111	GMNN	2,2	1,993E-02												
2114	2112	GTSE1	2,7	2,648E-04					2112	SNRNP35	2,2	3,827E-03												
2115	2113	WDR35	2,7	1,322E-04					2113	MBD4	2,2	9,092E-04												
2116	2114	H2AFZ	2,7	2,681E-05					2114	LGALS8	2,2	1,329E-03												
2117	2115	APAF1	2,7	3,935E-03					2115	CDC5L	2,2	8,835E-06												
2118	2116	MON1B	2,7	1,515E-03					2116	JAK2	2,2	4,609E-06												
2119	2117	NSUN3	2,7	1,502E-03					2117	ZFP36L2	2,2	3,398E-05												
2120	2118	PCBD2	2,7	3,857E-06					2118	NSL1	2,2	2,457E-04												
2121	2119	MED27	2,7	3,691E-06					2119	NUP37	2,2	6,866E-05												
2122	2120	PGM2	2,7	4,115E-03					2120	HMHA1	2,2	2,395E-02												
2123	2121	LRRC37A3	2,7	2,812E-02					2121	KCTD5	2,2	9,602E-06												
2124	2122	MED28	2,7	3,641E-04					2122	LARP4	2,2	1,948E-03												
2125	2123	UBE2I	2,7	1,754E-03					2123	SPPL3	2,2	1,128E-04												
2126	2124	ADORA2B	2,7	3,804E-04					2124	THRA	2,2	1,290E-02												
2127	2125	EFCAB11	2,7	4,839E-04					2125	DDX47	2,2	3,187E-04												
2128	2126	PSENEN	2,7	4,241E-05					2126	ANKRD32	2,2	4,057E-02												
2129	2127	LACTB2	2,7	5,522E-05					2127	ZEB1-AS1	2,2	9,442E-03												
2130	2128	PPIL4	2,7	3,631E-04					2128	RIC8B	2,2	2,852E-02												
2131	2129	OCRL	2,7	6,932E-05					2129	RBL2	2,2	2,298E-04												
2132	2130	MORN4	2,7	4,805E-04					2130	TCF7L1	2,1	2,613E-04												
2133	2131	VPS35	2,7	2,371E-02					2131	PPHLN1	2,1	1,013E-03												
2134	2132	SETD9	2,7	7,628E-06					2132	CSNK1G1	2,1	1,089E-04												
2135	2133	CSTF2	2,7	3,729E-05					2133	CHST10	2,1	2,454E-04												
2136	2134	BRAP	2,7	4,199E-05					2134	ATP5G2	2,1	2,329E-03												
2137	2135	DAB2IP	2,7	8,191E-03					2135	FXR1	2,1	3,176E-05												
2138	2136	CCDC59	2,7	1,382E-04					2136	C7orf55	2,1	1,589E-02												
2139	2137	ZNF652	2,7	4,032E-02					2137	PILRA	2,1	4,929E-02												
2140	2138	INPP4A	2,7	1,003E-03					2138	NPTN	2,1	1,492E-05												
2141	2139	ABLIM1	2,7	6,462E-05					2139	CNNM3	2,1	6,079E-04												
2142	2140	MCAT	2,7	4,138E-04					2140	KIF1B	2,1	3,780E-03												
2143	2141	PPTC7	2,7	1,042E-04					2141	GRB10	2,1	5,932E-05												
2144	2142	ZFY	2,7	6,016E-04					2142	ZNF608	2,1	3,245E-04												
2145	2143	APTX	2,7	1,849E-04					2143	SLC4A7	2,1	1,180E-03												
2146	2144	ZNF747	2,7	2,958E-03					2144	MTX3	2,1	1,881E-03												
2147	2145	SLC9A3R2	2,7	4,370E-02					2145	WDR59	2,1	3,938E-04												
2148	2146	WDR66	2,7	9,414E-03					2146	JAG1	2,1	7,231E-05												
2149	2147	CDCA7L	2,7	1,770E-04					2147	CDK2	2,1	3,158E-02												
2150	2148	CREB3L4	2,7	2,782E-03					2148	PPIG	2,1	5,795E-03												
2151	2149	SAP30BP	2,7	5,898E-05					2149	CCNT1	2,1	1,034E-04												
2152	2150	YBEY	2,7	4,189E-04					2150	SNX9	2,1	1,237E-05												
2153	2151	ZC3H12C	2,7	2,897E-03					2151	MEIS2	2,1	2,242E-03												
2154	2152	FMO4	2,7	6,824E-04					2152	BTBD1	2,1	2,114E-04												
2155	2153	PSPN	2,7	4,326E-04					2153	BIRC3	2,1	2,239E-02												
2156	2154	RC3H1	2,7	2,691E-03					2154	CUEDC1	2,1	4,088E-04												
2157	2155	TUB	2,7	4,114E-03					2155	PPM1A	2,1	9,761E-03												
2158	2156	NDOR1	2,7	3,187E-02					2156	ETAA1	2,1	2,989E-04												
2159	2157	FAM20B	2,7	1,249E-04					2157	ATP13A3	2,1	9,755E-05												
2160	2158	FKBP5	2,7	9,157E-06					2158	RNF6	2,1	1,185E-02												
2161	2159	AMZ2P1	2,7	1,715E-03					2159	KCNMA1	2,1	6,114E-03												
2162	2160	CEBPG	2,7	4,404E-04					2160	DHX38	2,1	3,352E-04												
2163	2161	CYB5D2	2,7	9,909E-03					2161	WDR76	2,1	1,422E-02												
2164	2162	IPPK	2,7	2,399E-03					2162	KDM1A	2,1	2,297E-05												
2165	2163	CCDC167	2,7	8,017E-06					2163	RMND5A	2,1	2,180E-04												
2166	2164	IRS1	2,7	9,211E-04					2164	ARFGAP3	2,1	1,712E-04												
2167	2165	PCBP1-AS1	2,7	2,929E-03					2165	ASF1B	2,1	3,439E-04												
2168	2166	PCM1	2,7	8,387E-03					2166	UTRN	2,1	2,061E-03												
2169	2167	ATXN1L	2,7	8,714E-06					2167	MOB1A	2,1	5,549E-05												
2170	2168	THTPA	2,7	3,827E-05					2168	PIK3CA	2,1	1,731E-03												
2171	2169	LCOR	2,7	4,586E-02					2169	TBC1D23	2,1	6,293E-05												
2172	2170	HIP1	2,7	1,685E-05					2170	GTPBP1	2,1	2,222E-02												
2173	2171	ZNF324B	2,7	4,888E-03					2171	LMNB1	2,1	1,758E-03												
2174	2172	UBAC2-AS1	2,7	6,638E-03					2172	MAPK1	2,1	1,730E-05												
2175	2173	SRRM2	2,7	6,109E-04					2173	FBXO5	2,1	7,190E-04												
2176	2174	C12orf76	2,7	6,836E-04					2174	CTSK	2,1	1,126E-02												
2177	2175	RELL1	2,7	3,450E-04					2175	KIF20B	2,1	1,443E-04												
2178	2176	CNDP2	2,7	1,346E-03					2176	FAM134C	2,1	6,648E-05												
2179	2177	AMFR	2,7	7,118E-03					2177	TMEM30A	2,1	1,094E-05												
2180	2178	C6orf62	2,7	6,585E-05					2178	NUP50	2,1	9,153E-04												
2181	2179	CBFB	2,7	4,723E-05					2179	PTRH2	2,1	1,183E-03												
2182	2180	RAB28	2,7	1,593E-02					2180	EFCAB2	2,1	6,760E-03												
2183	2181	BCKDK	2,7	8,780E-05					2181	WDR73	2,1	9,214E-04												
2184	2182	RHEB	2,7	2,215E-04					2182	ZNF664	2,1	2,145E-04												
2185	2183	DSTYK	2,7	3,043E-04					2183	RAB34	2,1	7,846E-06												
2186	2184	PSPC1	2,7	1,889E-05					2184	OAS3	2,1	5,323E-04												
2187	2185	SLC25A11	2,7	1,218E-04					2185	ZNF274	2,1	4,443E-03												
2188	2186	PIK3R3	2,7	3,109E-04					2186	SSFA2	2,1	3,216E-05												
2189	2187	ATP6V0A2	2,7	1,598E-03					2187	FTSJ3	2,1	7,457E-04												
2190	2188	PLRG1	2,7	1,375E-03					2188	B9D1	2,1	1,470E-03												
2191	2189	TRRAP	2,7	4,681E-04					2189	SLC35B2	2,1	3,606E-03												
2192	2190	MIS18BP1	2,7	2,927E-03					2190	SNX27	2,1	4,894E-03												
2193	2191	TOP3A	2,7	5,120E-05					2191	FKRP	2,1	2,144E-03												
2194	2192	VAMP7	2,7	7,016E-03					2192	CCNG2	2,1	2,475E-03												
2195	2193	KIF1B	2,7	2,161E-04					2193	ATR	2,1	2,678E-03												
2196	2194	KDM4C	2,7	4,107E-03					2194	DCPS	2,1	3,569E-04												
2197	2195	C1orf27	2,7	9,184E-03					2195	GPC2	2,1	9,620E-03												
2198	2196	C16orf52	2,7	7,764E-04					2196	PCNP	2,1	5,056E-05												
2199	2197	PRRC2B	2,7	5,482E-05					2197	DNAJC3	2,1	1,542E-05												
2200	2198	TSR2	2,7	1,053E-05					2198	PLK1	2,1	8,141E-03												
2201	2199	CHIC1	2,7	6,100E-03					2199	LPAR1	2,1	3,437E-02												
2202	2200	ZBTB49	2,7	5,572E-04					2200	BRCA1	2,1	1,464E-02												
2203	2201	TMEM60	2,7	5,120E-04					2201	IFI27	2,1	4,660E-03												
2204	2202	ZNRF3	2,7	2,636E-04					2202	ADIPOR2	2,1	2,053E-05												
2205	2203	TLK2	2,7	3,217E-05					2203	FBXO45	2,1	9,804E-04												
2206	2204	FMR1	2,7	1,394E-02					2204	COQ7	2,1	1,900E-03												

	A	B	C	D	E	F	G	H	I	J	K	L	M	N	O	P	Q	R	S	T	U	V	W	X
2207	2205	FIGN	2,7	2,133E-03					2205	PACSIN3	2,1	4,382E-04												
2208	2206	HDAC2	2,7	4,964E-02					2206	RACGAP1	2,1	9,944E-04												
2209	2207	METTL4	2,7	5,503E-04					2207	SNX25	2,1	8,522E-04												
2210	2208	SLC16A1	2,6	4,232E-03					2208	RASA2	2,1	1,685E-03												
2211	2209	HOXC11	2,6	8,504E-03					2209	PRIM2	2,1	2,530E-04												
2212	2210	IPO8	2,6	1,234E-04					2210	ARL6IP6	2,1	7,834E-03												
2213	2211	ZSWIM6	2,6	8,859E-05					2211	COX7A2	2,1	9,389E-03												
2214	2212	MIDN	2,6	8,517E-03					2212	TTC26	2,1	9,618E-05												
2215	2213	FAM102B	2,6	3,378E-04					2213	DYNLL2	2,1	8,961E-05												
2216	2214	C2CD2L	2,6	3,189E-05					2214	LYAR	2,1	3,321E-06												
2217	2215	PITPNM1	2,6	4,903E-02					2215	ZC3H7B	2,1	1,457E-04												
2218	2216	FDPS	2,6	8,943E-04					2216	SLC29A1	2,1	2,197E-04												
2219	2217	SNRPD1	2,6	9,134E-04					2217	CCDC112	2,1	1,336E-03												
2220	2218	PNKD	2,6	8,258E-05					2218	CHMP5	2,1	1,215E-04												
2221	2219	ANKLE1	2,6	2,012E-02					2219	ANKS1A	2,1	1,002E-04												
2222	2220	MRPS18B	2,6	1,875E-04					2220	UTP20	2,1	3,116E-03												
2223	2221	PTCD3	2,6	2,080E-04					2221	C11orf49	2,1	7,172E-04												
2224	2222	DSG2	2,6	4,557E-05					2222	MYO10	2,1	1,482E-06												
2225	2223	PTCH1	2,6	3,551E-04					2223	GPD1L	2,1	2,509E-02												
2226	2224	RBL1	2,6	1,487E-03					2224	CDC14A	2,1	9,959E-03												
2227	2225	DNAJC14	2,6	1,687E-02					2225	METTL23	2,1	1,930E-03												
2228	2226	COQ5	2,6	1,669E-06					2226	BRSK1	2,1	1,348E-02												
2229	2227	N4BP2	2,6	1,075E-02					2227	AHCYL1	2,1	9,599E-04												
2230	2228	GUCY1B3	2,6	4,826E-05					2228	FOXL2	2,1	4,288E-03												
2231	2229	IL18BP	2,6	3,245E-02					2229	UBLCP1	2,1	6,729E-05												
2232	2230	ABCF3	2,6	4,301E-04					2230	CCDC23	2,1	7,199E-04												
2233	2231	ACTR3B	2,6	8,309E-04					2231	KIAA0556	2,1	2,304E-03												
2234	2232	PLAA	2,6	1,489E-04					2232	FKBP4	2,1	1,115E-04												
2235	2233	ST7L	2,6	8,604E-04					2233	DYNC1LI2	2,1	2,096E-03												
2236	2234	ING3	2,6	6,066E-03					2234	C14orf1	2,1	3,953E-05												
2237	2235	AKIRIN1	2,6	1,609E-04					2235	CCDC18	2,1	1,622E-02												
2238	2236	GINS4	2,6	9,099E-04					2236	TMEM161B	2,1	2,153E-02												
2239	2237	INCENP	2,6	3,546E-04					2237	TPRA1	2,1	2,479E-03												
2240	2238	GPR156	2,6	1,775E-03					2238	FNIP2	2,1	1,067E-03												
2241	2239	ZNF506	2,6	1,502E-03					2239	GAK	2,1	2,253E-03												
2242	2240	CBR3	2,6	6,782E-07					2240	CHD8	2,1	8,638E-03												
2243	2241	PDE4D	2,6	1,937E-03					2241	DDX3Y	2,1	4,768E-04												
2244	2242	TADA2A	2,6	3,554E-03					2242	ALG6	2,1	9,400E-06												
2245	2243	SDF2	2,6	2,824E-03					2243	UPRT	2,1	6,910E-03												
2246	2244	TRIM26	2,6	2,522E-04					2244	FAM214A	2,1	4,608E-03												
2247	2245	ERMAP	2,6	8,756E-05					2245	B3GNT1	2,1	3,708E-04												
2248	2246	E2F3	2,6	6,374E-04					2246	DLG1	2,1	1,081E-04												
2249	2247	SERPINB8	2,6	3,765E-03					2247	THUMPD1	2,1	1,301E-03												
2250	2248	ZBTB9	2,6	2,301E-05					2248	RBM48	2,1	4,050E-04												
2251	2249	ZNF248	2,6	5,007E-04					2249	EDEM1	2,1	1,844E-05												
2252	2250	USP53	2,6	1,201E-03					2250	SGOL1	2,1	5,072E-03												
2253	2251	AHCTF1	2,6	1,623E-02					2251	HACL1	2,1	3,636E-05												
2254	2252	ZNF425	2,6	9,465E-05					2252	MTF1	2,1	8,564E-04												
2255	2253	ZBTB41	2,6	5,847E-04					2253	CCNL1	2,1	4,457E-02												
2256	2254	MTO1	2,6	2,926E-02					2254	RTKN	2,1	7,665E-05												
2257	2255	PARD3	2,6	5,169E-04					2255	BTBD7	2,1	5,208E-05												
2258	2256	KIAA0101	2,6	3,240E-04					2256	PIGV	2,1	5,819E-03												
2259	2257	PRKAB1	2,6	1,580E-04					2257	SMARCA5	2,1	2,254E-05												
2260	2258	GIGYF2	2,6	1,317E-04					2258	C6orf211	2,1	1,820E-02												
2261	2259	NUP133	2,6	5,767E-06					2259	CDK5RAP2	2,1	2,049E-03												
2262	2260	C9orf96	2,6	2,672E-03					2260	PDCD6	2,1	3,752E-04												
2263	2261	TMEM128	2,6	1,288E-06					2261	IMPA1	2,1	5,918E-03												
2264	2262	EXOC8	2,6	9,232E-06					2262	HMGB3	2,1	6,404E-04												
2265	2263	ADCY9	2,6	2,497E-05					2263	ZNF480	2,1	1,050E-03												
2266	2264	NFATC3	2,6	3,148E-05					2264	TBPL1	2,1	4,136E-03												
2267	2265	SUV39H2	2,6	4,503E-02					2265	PIGX	2,1	1,952E-03												
2268	2266	GSTK1	2,6	1,211E-06					2266	GABPA	2,1	7,762E-05												
2269	2267	ASTE1	2,6	9,647E-04					2267	ZMYM1	2,1	8,005E-03												
2270	2268	RBBP6	2,6	4,461E-05					2268	IVD	2,1	8,727E-05												
2271	2269	RANBP9	2,6	7,402E-05					2269	GLYR1	2,1	1,997E-02												
2272	2270	NCALD	2,6	7,972E-03					2270	TBL1XR1	2,1	1,165E-03												
2273	2271	CTSO	2,6	3,738E-04					2271	NHLRC3	2,1	3,570E-04												
2274	2272	CTTNBP2NL	2,6	3,943E-04					2272	ZNF420	2,1	5,569E-03												
2275	2273	NUDC	2,6	4,412E-05					2273	WRNIP1	2,1	4,346E-06												
2276	2274	RBPMS2	2,6	9,809E-04					2274	SYNJ2BP	2,1	5,977E-04												
2277	2275	GPN1	2,6	3,054E-04					2275	C11orf74	2,1	1,127E-04												
2278	2276	TRIM23	2,6	4,089E-04					2276	LGALS9	2,1	1,829E-02												
2279	2277	LACE1	2,6	4,632E-03					2277	ACOT7	2,1	4,524E-03												
2280	2278	NUFIP2	2,6	1,141E-04					2278	PKP4	2,1	3,364E-05												
2281	2279	MCFD2	2,6	8,368E-04					2279	HMGXB4	2,1	4,032E-04												
2282	2280	ZNF83	2,6	2,667E-04					2280	TFDP1	2,1	1,170E-03												
2283	2281	CHD9	2,6	4,281E-04					2281	INPP4A	2,1	3,913E-03												
2284	2282	BMP4	2,6	2,386E-03					2282	KAT6B	2,1	4,272E-05												
2285	2283	DUS4L	2,6	1,305E-03					2283	RIPK1	2,1	3,804E-06												
2286	2284	SP2	2,6	1,926E-03					2284	BAZ2A	2,1	8,020E-03												
2287	2285	KLHL8	2,6	1,935E-04					2285	UBIAD1	2,1	5,690E-05												
2288	2286	CMTM4	2,6	4,321E-03					2286	JAGN1	2,1	1,472E-04												
2289	2287	NRF1	2,6	3,445E-04					2287	DOLPP1	2,1	6,517E-05												
2290	2288	NDUFS2	2,6	3,312E-04					2288	WDR35	2,1	6,813E-04												
2291	2289	CIAPIN1	2,6	2,436E-04					2289	ASH2L	2,1	6,413E-04												
2292	2290	NR1H3	2,6	4,086E-05					2290	ABHD15	2,1	1,958E-04												
2293	2291	C4orf46	2,6	6,681E-05					2291	HSBP1	2,1	2,842E-05												
2294	2292	SETD1A	2,6	3,135E-03					2292	TP53BP1	2,1	2,575E-05												
2295	2293	MFSD5	2,6	3,667E-06					2293	PIKFYVE	2,1	1,165E-06												
2296	2294	DHX9	2,6	4,102E-04					2294	WBP11	2,1	6,939E-05												
2297	2295	PARP11	2,6	2,498E-04					2295	MRPL47	2,1	1,086E-02												
2298	2296	TARS2	2,6	6,455E-05					2296	TCP11L2	2,1	5,819E-03												
2299	2297	WDR5B	2,6	1,383E-03					2297	PTCD3	2,1	2,001E-03												
2300	2298	KLF11	2,6	2,818E-05					2298	STARD7	2,1	9,701E-06												
2301	2299	PNPO	2,6	5,178E-03					2299	MAPK3	2,1	5,658E-04												
2302	2300	RABIF	2,6	2,423E-06					2300	MAML2	2,1	4,821E-06												
2303	2301	PLEKHA5	2,6	2,653E-04					2301	MAD2L1	2,1	8,403E-03												
2304	2302	TXLNG2P	2,6	1,237E-03					2302	PNISR	2,1	3,382E-02												
2305	2303	ASAP3	2,6	4,459E-03					2303	RAB3A	2,1	1,581E-02												
2306	2304	CSTF2T	2,6	1,857E-04					2304	DBF4B	2,1	2,686E-06												
2307	2305	NPR2	2,6	4,618E-04					2305	ERI2	2,1	1,987E-02												
2308	2306	MOSPD1	2,6	1,014E-04					2306	GLT8D1	2,1	4,413E-04												
2309	2307	C18orf54	2,6	2,168E-02					2307	TCEANC2	2,1	1,403E-02												
2310	2308	LPCAT2	2,6	3,080E-04					2308	PBX1	2,1	9,355E-04												
2311	2309	TFAM	2,6	8,759E-04					2309	SMARCD1	2,1	1,438E-03												
2312	2310	ETAA1	2,6	1,271E-03					2310	TBC1D22B	2,1	4,164E-04												
2313	2311	WDHD1	2,6	1,480E-03					2311	NCL	2,1	7,284E-04												
2314	2312	SLC35D2	2,6	3,404E-04					2312	VRK1	2,1	1,271E-03												
2315	2313	GPI	2,6	3,581E-04					2313	ERLIN2	2,1	1,098E-04												
2316	2314	IRF2	2,6	1,523E-03					2314	HMG20A	2,1	1,195E-02												
2317	2315	TAF15	2,6	7,542E-04					2315	CWF19L2	2,1	1,470E-03												
2318	2316	SPR	2,6	5,129E-04					2316	TSPAN31	2,1	1,081E-02												
2319	2317	BLOC1S2	2,6	1,870E-06					2317	FAM96B	2,1	7,759E-04												
2320	2318	CDK2AP1	2,6	1,159E-02					2318	FBXO10	2,1	1,129E-03												
2321	2319	GRIPAP1	2,6	1,996E-05					2319	KATNB1	2,1	2,641E-03												
2322	2320	ME2	2,6	2,795E-05					2320	BRPF3	2,1	3,784E-03												
2323	2321	PITHD1	2,6	1,803E-05					2321	VAPB	2,0	3,647E-03												
2324	2322	TSPAN6	2,6	6,167E-05					2322	CENPM	2,0	1,078E-02												
2325	2323	TMEM161B	2,6	5,810E-03					2323	RAB5A	2,0	8,234E-03												
2326	2324	HAUS4	2,6	1,758E-04					2324	TMEM184C	2,0	4,327E-04												
2327	2325	WDPCP	2,6	5,779E-03					2325	APLP1	2,0	9,357E-04												
2328	2326	PEX7	2,6	1,110E-04					2326	KDM5A	2,0	6,575E-04												
2329	2327	CHST11	2,6	8,371E-04					2327	INADL	2,0	1,441E-04												
2330	2328	FH	2,6	8,510E-06					2328	CASP7	2,0	4,238E-04												
2331	2329	FAM200A	2,6	1,553E-03					2329	PCGF3	2,0	4,845E-02												
2332	2330	ELMOD2	2,6	2,690E-03					2330	ZNF430	2,0	6,647E-04												
2333	2331	C12orf49	2,6	6,923E-04					2331	BAZ1A	2,0	1,774E-04												
2334	2332	MCRS1	2,6	2,925E-03					2332	C10orf2	2,0	4,447E-05												
2335	2333	SFT2D1	2,6	4,409E-03					2333	RFC4	2,0	4,382E-02												
2336	2334	NMRAL1	2,6	2,291E-03					2334	C17orf75	2,0	5,865E-03												
2337	2335	STXBP3	2,6	4,642E-06					2335	TAF11	2,0	3,934E-04												
2338	2336	MAPK1IP1L	2,6	7,199E-03					2336	PTCD2	2,0	2,032E-02												
2339	2337	MOAP1	2,6	1,760E-04					2337	SLC25A30	2,0	2,184E-02												
2340	2338	CASP1	2,6	1,329E-03					2338	IKBIP	2,0	1,248E-04												
2341	2339	POLR3GL	2,6	8,456E-03					2339	ATP6V1E1	2,0	2,806E-04												
2342	2340	AACS	2,6	1,595E-04					2340	PTPN11	2,0	1,136E-05												
2343	2341	DHODH	2,6	2,325E-04					2341	CLCN3	2,0	6,181E-04												
2344	2342	C11orf49	2,6	1,236E-05					2342	KDM1B	2,0	1,322E-05												
2345	2343	FASTKD5	2,6	1,093E-04					2343	C1orf85	2,0	4,916E-04												
2346	2344	ZNF670	2,6	2,934E-03					2344	CLEC16A	2,0	3,449E-05												
2347	2345	TIPIN	2,6	2,615E-03					2345	GNL3	2,0	9,953E-04												
2348	2346	TBC1D22B	2,6	2,801E-04					2346	MTERFD3	2,0	3,203E-03												
2349	2347	ZNF430	2,6	4,986E-04					2347	SMARCD2	2,0	1,967E-03												
2350	2348	ABCC3	2,6	4,544E-04					2348	TMEM33	2,0	4,995E-02												
2351	2349	COX7C	2,6	8,814E-03					2349	CCDC96	2,0	2,967E-02												
2352	2350	ARV1	2,6	3,895E-05					2350	KIAA0947	2,0	2,170E-05												
2353	2351	DCAF7	2,5	3,143E-04					2351	RSRC2	2,0	1,149E-04												
2354	2352	MCCC2	2,5	7,538E-05					2352	CWC22	2,0	2,330E-03												
2355	2353	CELF2	2,5	2,898E-04					2353	METAP2	2,0	2,488E-04												
2356	2354	DCK	2,5	3,961E-05					2354	SERPINB6	2,0	2,706E-05												
2357	2355	OGFOD2	2,5	3,811E-03					2355	PDE4B	2,0	8,746E-03												
2358	2356	CYP2U1	2,5	5,131E-04					2356	CCDC82	2,0	6,062E-04												
2359	2357	NFYB	2,5	3,407E-03					2357	CEP192	2,0	1,435E-02												
2360	2358	IPP	2,5	6,036E-04					2358	HPS5	2,0	2,433E-04												
2361	2359	KIAA0586	2,5	3,128E-04					2359	CLK1	2,0	3,796E-03												
2362	2360	NDFIP2	2,5	1,930E-03					2360	PLSCR1	2,0	2,870E-03												
2363	2361	DTX3	2,5	3,667E-04					2361	IL7	2,0	3,175E-02												
2364	2362	TRIM25	2,5	1,132E-03					2362	CEP76	2,0	1,413E-02												
2365	2363	LOH12CR1	2,5	1,819E-02					2363	MFSD8	2,0	5,235E-03												
2366	2364	KIF5C	2,5	7,638E-05					2364	SLC26A11	2,0	5,736E-03												
2367	2365	ARRDC1	2,5	1,689E-03					2365	POLR3GL	2,0	3,318E-03												
2368	2366	MRI1	2,5	1,820E-04					2366	RNF219	2,0	1,200E-04												
2369	2367	MECP2	2,5	3,392E-05					2367	SLC7A6OS	2,0	3,136E-04												
2370	2368	RCL1	2,5	2,250E-02					2368	OPA1	2,0	6,358E-04												
2371	2369	DNMT1	2,5	2,494E-03					2369	NPEPPS	2,0	6,355E-03												
2372	2370	SCYL2	2,5	5,244E-04					2370	TMEM181	2,0	4,650E-06												
2373	2371	FAM96B	2,5	9,590E-04					2371	ALPK1	2,0	8,838E-04												
2374	2372	IQCG	2,5	2,692E-04					2372	SMYD4	2,0	1,352E-04												
2375	2373	ALPK1	2,5	6,960E-04					2373	ZBTB49	2,0	1,080E-02												
2376	2374	INADL	2,5	1,314E-02					2374	NMD3	2,0	2,827E-03												
2377	2375	GTF3C5	2,5	1,157E-03					2375	PEX12	2,0	8,337E-03												
2378	2376	AKIRIN2	2,5	3,855E-05					2376	HES6	2,0	1,670E-03												
2379	2377	ANKRA2	2,5	1,851E-02					2377	LETMD1	2,0	9,928E-03												
2380	2378	KIAA1147	2,5	1,645E-06					2378	LLPH	2,0	2,565E-04												
2381	2379	ADAT1	2,5	2,589E-06					2379	TTC30B	2,0	4,893E-02												
2382	2380	FUNDC1	2,5	4,033E-02					2380	CCDC25	2,0	3,635E-02												
2383	2381	ZKSCAN4	2,5	3,008E-04					2381	SOCS3	2,0	4,872E-05												
2384	2382	POLG2	2,5	8,755E-04					2382	COMMD2	2,0	2,446E-02												
2385	2383	RRM1	2,5	1,135E-04					2383	MBTPS1	2,0	5,721E-04												
2386	2384	EXOSC2	2,5	3,258E-04					2384	C9orf116	2,0	2,908E-02												
2387	2385	CEACAM19	2,5	1,851E-02					2385	MAP6D1	2,0	1,670E-02												
2388	2386	MBTPS2	2,5	1,698E-03					2386	CCNDBP1	2,0	3,769E-04												
2389	2387	ERVK13-1	2,5	8,548E-03					2387	TRAF3IP1	2,0	3,311E-03												
2390	2388	TCF4	2,5	1,453E-02					2388	GIPR	2,0	3,884E-02												
2391	2389	KLHL5	2,5	1,121E-05					2389	TPX2	2,0	4,975E-04												
2392	2390	YJEFN3	2,5	8,546E-04					2390	IRAK1BP1	2,0	3,356E-02												
2393	2391	YOD1	2,5	5,628E-04					2391	TGFBR2	2,0	1,552E-06												
2394	2392	MAGI3	2,5	2,527E-03					2392	TMEM50B	2,0	1,731E-02												
2395	2393	MYLIP	2,5	7,185E-04					2393	RNF103	2,0	7,153E-03												
2396	2394	ETFDH	2,5	2,823E-03					2394	ACAD10	2,0	1,334E-02												
2397	2395	MYBL2	2,5	2,655E-06					2395	RAB15	2,0	2,156E-04												
2398	2396	DGCR11	2,5	4,361E-03					2396	FNDC3B	2,0	1,019E-02												
2399	2397	ZKSCAN5	2,5	1,127E-04					2397	SP3	2,0	1,103E-02												
2400	2398	WHSC1	2,5	2,380E-03					2398	SLC25A20	2,0	2,172E-02												
2401	2399	MRPL44	2,5	3,415E-05					2399	PNMAL1	2,0	1,750E-04												
2402	2400	FAM73A	2,5	5,710E-04					2400	ZBTB38	2,0	1,437E-03												
2403	2401	TMBIM6	2,5	3,693E-02					2401	PKN2	2,0	8,428E-04												
2404	2402	PAPOLG	2,5	3,121E-03					2402	DLGAP5	2,0	2,349E-06												
2405	2403	NCAPG2	2,5	1,967E-05					2403	TGFB3	2,0	4,491E-04												
2406	2404	APOBEC3F	2,5	2,464E-03					2404	AURKB	2,0	2,697E-03												
2407	2405	TLCD2	2,5	3,946E-02					2405	CDT1	2,0	2,665E-02												
2408	2406	MDH1	2,5	1,924E-03					2406	ZNF334	2,0	2,362E-03												

	A	B	C	D	E	F	G	H	I	J	K	L	M	N	O	P	Q	R	S	T	U	V	W	X
2409	2407	AFAP1L2	2,5	4,841E-02					2407	EZH2	2,0	5,210E-04												
2410	2408	RBM12B	2,5	3,304E-03					2408	VPS33A	2,0	9,860E-04												
2411	2409	SNX4	2,5	7,966E-05					2409	ZKSCAN5	2,0	3,523E-04												
2412	2410	POLR1E	2,5	1,144E-05					2410	NUP107	2,0	1,108E-03												
2413	2411	DSN1	2,5	1,278E-04					2411	PSMD2	2,0	8,079E-05												
2414	2412	TSPYL4	2,5	1,308E-06					2412	TKT	2,0	3,344E-03												
2415	2413	MIF4GD	2,5	1,260E-02					2413	TXNDC11	2,0	1,416E-04												
2416	2414	DCTPP1	2,5	2,358E-03					2414	TNRC6A	2,0	5,213E-04												
2417	2415	TP53TG1	2,5	1,283E-03					2415	RPRD1B	2,0	2,178E-04												
2418	2416	DDX59	2,5	5,916E-03					2416	LIAS	2,0	2,529E-04												
2419	2417	UBE2C	2,5	6,430E-04					2417	CRKL	2,0	1,816E-05												
2420	2418	PRDX6	2,5	3,797E-06					2418	PTGS1	2,0	5,695E-03												
2421	2419	PGBD4	2,5	2,843E-03					2419	CTSO	2,0	8,183E-06												
2422	2420	PDZD4	2,5	2,688E-02					2420	SCARF2	2,0	3,613E-02												
2423	2421	SNRPF	2,5	1,240E-06					2421	SPATA6	2,0	1,328E-02												
2424	2422	CLN5	2,5	2,252E-04					2422	VPS53	2,0	1,456E-03												
2425	2423	TBPL1	2,5	8,098E-04					2423	ZSCAN12	2,0	1,350E-02												
2426	2424	C1orf131	2,5	4,147E-05					2424	NR2C1	2,0	3,175E-04												
2427	2425	SARNP	2,5	1,356E-02					2425	NBR1	2,0	1,189E-04												
2428	2426	LLGL2	2,5	1,930E-02					2426	TOMM70A	2,0	6,249E-06												
2429	2427	NCBP2	2,5	9,611E-03					2427	SCYL3	2,0	9,976E-03												
2430	2428	RPAP3	2,5	7,538E-05					2428	PPIL1	2,0	2,295E-05												
2431	2429	PPP4R4	2,5	6,264E-03					2429	ZBED3	2,0	1,579E-03												
2432	2430	RPP30	2,5	7,038E-04					2430	SENP7	2,0	2,254E-02												
2433	2431	HBS1L	2,5	3,037E-02					2431	POLG2	2,0	1,347E-02												
2434	2432	TAF7	2,5	1,455E-04					2432	RRAGA	2,0	1,417E-04												
2435	2433	COASY	2,5	5,178E-03					2433	BDH2	2,0	1,568E-02												
2436	2434	AJUBA	2,5	1,061E-03					2434	CTBS	2,0	2,796E-02												
2437	2435	SEC11C	2,5	1,271E-03					2435	ERN1	2,0	7,202E-03												
2438	2436	TRMT2B	2,5	6,838E-03					2436	SGCB	2,0	1,477E-02												
2439	2437	ZBTB39	2,5	2,030E-04					2437	WSB2	2,0	6,452E-03												
2440	2438	RBM48	2,5	5,462E-05					2438	AMPD2	2,0	7,285E-04												
2441	2439	EXOG	2,5	1,711E-03					2439	DLAT	2,0	5,676E-04												
2442	2440	KIAA1586	2,5	2,978E-04					2440	ZNF605	2,0	2,331E-02												
2443	2441	ZBTB26	2,5	3,747E-04					2441	CENPW	2,0	3,560E-05												
2444	2442	CNKSR3	2,5	6,664E-04					2442	ATN1	2,0	7,510E-04												
2445	2443	TRAF2	2,5	1,672E-04					2443	PGAP3	2,0	2,453E-03												
2446	2444	IPMK	2,5	2,415E-03					2444	ABHD16A	2,0	1,787E-04												
2447	2445	GDI1	2,5	8,523E-04																				
2448	2446	ILF2	2,5	6,182E-04																				
2449	2447	RCBTB1	2,5	2,095E-04																				
2450	2448	SURF1	2,5	4,866E-05																				
2451	2449	CDKN2B	2,5	2,273E-02																				
2452	2450	S100A4	2,5	1,727E-04																				
2453	2451	STAG2	2,5	1,010E-03																				
2454	2452	PARP12	2,5	5,058E-04																				
2455	2453	RPS4Y1	2,5	7,743E-05																				
2456	2454	CHTF8	2,5	1,458E-03																				
2457	2455	C17orf75	2,5	4,760E-05																				
2458	2456	RAB14	2,5	1,612E-04																				
2459	2457	PTPRU	2,5	2,970E-02																				
2460	2458	ANKRD46	2,5	3,383E-03																				
2461	2459	CBY1	2,5	1,984E-03																				
2462	2460	EBF4	2,5	4,433E-02																				
2463	2461	AARS2	2,5	1,188E-03																				
2464	2462	BROX	2,5	1,165E-03																				
2465	2463	ZNF256	2,5	1,287E-04																				
2466	2464	MFSD8	2,5	2,484E-03																				
2467	2465	DCP1A	2,5	1,115E-02																				
2468	2466	SLC25A19	2,5	6,394E-03																				
2469	2467	AKAP10	2,5	2,106E-04																				
2470	2468	HECA	2,5	2,226E-04																				
2471	2469	ZNF195	2,5	4,600E-03																				
2472	2470	AUTS2	2,5	1,133E-03																				
2473	2471	ACADSB	2,5	1,120E-02																				
2474	2472	POLE	2,5	1,949E-02																				
2475	2473	TRIM39	2,5	4,515E-04																				
2476	2474	KIF3A	2,5	2,704E-04																				
2477	2475	KDM3B	2,5	7,817E-05																				
2478	2476	RBM8A	2,5	3,126E-04																				
2479	2477	SGOL2	2,5	3,602E-03																				
2480	2478	MEA1	2,5	1,591E-06																				
2481	2479	TUFT1	2,5	1,622E-02																				
2482	2480	RHOBTB3	2,5	1,951E-04																				
2483	2481	POLR3F	2,5	9,698E-04																				
2484	2482	ZNF567	2,5	2,446E-02																				
2485	2483	SETDB1	2,5	1,917E-03																				
2486	2484	KIAA0195	2,5	1,162E-02																				
2487	2485	ORC5	2,5	1,939E-04																				
2488	2486	ORC3	2,5	5,178E-05																				
2489	2487	MAD2L1	2,5	1,392E-03																				
2490	2488	GNA13	2,5	9,257E-05																				
2491	2489	EDC4	2,5	1,266E-03																				
2492	2490	ZNF687	2,5	3,032E-04																				
2493	2491	PPT1	2,5	5,674E-04																				
2494	2492	BBS10	2,5	3,179E-03																				
2495	2493	PRRC2A	2,5	1,569E-04																				
2496	2494	PI4KB	2,5	6,428E-03																				
2497	2495	VRK1	2,5	6,061E-04																				
2498	2496	KIAA0232	2,5	1,902E-05																				
2499	2497	RBBP5	2,5	7,966E-05																				
2500	2498	COG2	2,5	1,597E-05																				
2501	2499	SMCHD1	2,4	6,185E-04																				
2502	2500	LMLN	2,4	4,758E-06																				
2503	2501	TXNIP	2,4	7,595E-05																				
2504	2502	LYPLA2	2,4	7,675E-03																				
2505	2503	RWDD2A	2,4	2,999E-04																				
2506	2504	PBLD	2,4	9,999E-03																				
2507	2505	ZNF568	2,4	2,813E-05																				
2508	2506	RABL3	2,4	8,001E-03																				
2509	2507	MDK	2,4	3,395E-03																				
2510	2508	PLK3	2,4	1,750E-02																				
2511	2509	TSNAX	2,4	1,223E-02																				
2512	2510	TMSB15B	2,4	4,429E-04																				
2513	2511	WDR3	2,4	3,148E-03																				
2514	2512	AK1	2,4	3,469E-03																				
2515	2513	ULK2	2,4	5,245E-04																				
2516	2514	ZFP1	2,4	1,045E-03																				
2517	2515	USP5	2,4	1,470E-04																				
2518	2516	UTP20	2,4	1,044E-04																				
2519	2517	TREX1	2,4	2,251E-02																				
2520	2518	SLC35E3	2,4	3,991E-04																				
2521	2519	SPATA7	2,4	3,859E-02																				
2522	2520	GSPT2	2,4	1,296E-05																				
2523	2521	PET112	2,4	7,807E-04																				
2524	2522	TRIM33	2,4	4,587E-04																				
2525	2523	APPBP2	2,4	2,035E-04																				
2526	2524	C21orf33	2,4	3,950E-03																				
2527	2525	ADCK2	2,4	3,066E-04																				
2528	2526	ZMYM3	2,4	3,475E-05																				
2529	2527	NDRG2	2,4	3,847E-03																				
2530	2528	CHFR	2,4	1,001E-03																				
2531	2529	KRR1	2,4	1,599E-03																				
2532	2530	RPRD2	2,4	2,137E-05																				
2533	2531	CXorf23	2,4	3,123E-02																				
2534	2532	RABGAP1	2,4	2,446E-04																				
2535	2533	DOPEY1	2,4	1,918E-03																				
2536	2534	STOML2	2,4	7,603E-07																				
2537	2535	ARL3	2,4	1,391E-05																				
2538	2536	SETX	2,4	1,104E-03																				
2539	2537	RAPGEF2	2,4	2,002E-03																				
2540	2538	OGG1	2,4	6,849E-04																				
2541	2539	PKN2	2,4	6,775E-03																				
2542	2540	CDC40	2,4	2,670E-03																				
2543	2541	PEX5	2,4	5,085E-03																				
2544	2542	WRAP53	2,4	7,114E-05																				
2545	2543	EEF2K	2,4	2,032E-03																				
2546	2544	ZBTB6	2,4	1,328E-03																				
2547	2545	SMC3	2,4	6,498E-04																				
2548	2546	NAP1L5	2,4	8,280E-04																				
2549	2547	CCDC109B	2,4	2,253E-04																				
2550	2548	PEBP1	2,4	2,479E-07																				
2551	2549	CGGBP1	2,4	7,740E-05																				
2552	2550	ZNF404	2,4	6,285E-04																				
2553	2551	KIAA0226	2,4	2,086E-03																				
2554	2552	DHRSX	2,4	3,731E-04																				
2555	2553	PAQR4	2,4	8,515E-03																				
2556	2554	HAUS8	2,4	1,927E-02																				
2557	2555	POLR2C	2,4	3,785E-05																				
2558	2556	MAP3K7	2,4	2,104E-03																				
2559	2557	STRA13	2,4	1,619E-02																				
2560	2558	PRR3	2,4	1,436E-04																				
2561	2559	HEXIM2	2,4	7,328E-04																				
2562	2560	C3orf52	2,4	1,444E-02																				
2563	2561	MTHFSD	2,4	1,200E-03																				
2564	2562	HSPA13	2,4	3,800E-04																				
2565	2563	ENO3	2,4	1,578E-02																				
2566	2564	FAM98B	2,4	1,447E-04																				
2567	2565	FRMD5	2,4	1,094E-04																				
2568	2566	TMX4	2,4	1,430E-04																				
2569	2567	STMN1	2,4	1,163E-03																				
2570	2568	NCS1	2,4	5,110E-04																				
2571	2569	TMEM64	2,4	1,840E-04																				
2572	2570	CAB39	2,4	7,104E-04																				
2573	2571	KLHDC8B	2,4	2,724E-04																				
2574	2572	EPB41L5	2,4	3,160E-03																				
2575	2573	SOCS7	2,4	2,820E-03																				
2576	2574	CCDC24	2,4	7,316E-05																				
2577	2575	ECHDC1	2,4	3,296E-03																				
2578	2576	HSPA1B	2,4	1,776E-02																				
2579	2577	UQCRC2	2,4	1,858E-04																				
2580	2578	CHD7	2,4	1,294E-03																				
2581	2579	MRPS33	2,4	8,988E-05																				
2582	2580	PEX13	2,4	1,467E-03																				
2583	2581	MUT	2,4	2,272E-04																				
2584	2582	ZBED1	2,4	2,440E-02																				
2585	2583	MCM5	2,4	2,230E-03																				
2586	2584	RRP1B	2,4	1,270E-04																				
2587	2585	SNTB1	2,4	1,752E-05																				
2588	2586	ATE1	2,4	4,159E-03																				
2589	2587	ATXN7L1	2,4	2,475E-04																				
2590	2588	HNRNPC	2,4	1,949E-03																				
2591	2589	SNX3	2,4	8,073E-05																				
2592	2590	RQCD1	2,4	1,710E-04																				
2593	2591	FAM120AOS	2,4	3,198E-03																				
2594	2592	LRRC8A	2,4	2,096E-03																				
2595	2593	SYNJ1	2,4	1,129E-04																				
2596	2594	FMN2	2,4	3,618E-04																				
2597	2595	LYPLAL1	2,4	3,548E-03																				
2598	2596	RNF8	2,4	8,719E-03																				
2599	2597	VTA1	2,4	5,804E-04																				
2600	2598	MIER3	2,4	6,303E-05																				
2601	2599	TDP2	2,4	5,387E-05																				
2602	2600	MAFG	2,4	4,628E-03																				
2603	2601	NUP54	2,4	9,860E-04																				
2604	2602	RHBDD1	2,4	3,208E-04																				
2605	2603	SLC7A11	2,4	3,234E-03																				
2606	2604	HSPA14	2,4	2,260E-04																				
2607	2605	ZNF182	2,4	6,864E-04																				
2608	2606	ZC3HC1	2,4	4,537E-04																				
2609	2607	GOT1	2,4	4,152E-04																				
2610	2608	BPNT1	2,4	3,909E-03																				

	A	B	C	D	E	F	G	H	I	J	K	L	M	N	O	P	Q	R	S	T	U	V	W	X
2611	2609	STK38	2,4	4,842E-05																				
2612	2610	SCRN3	2,4	5,385E-03																				
2613	2611	TBCCD1	2,4	5,574E-04																				
2614	2612	C1orf35	2,4	1,404E-02																				
2615	2613	EIF4A3	2,4	6,850E-05																				
2616	2614	SECISBP2	2,4	1,111E-04																				
2617	2615	TNFAIP2	2,4	4,685E-02																				
2618	2616	ZNF853	2,4	1,629E-02																				
2619	2617	CUL1	2,4	5,837E-05																				
2620	2618	PDZD8	2,4	2,250E-04																				
2621	2619	LTN1	2,4	1,520E-04																				
2622	2620	UBXN2B	2,4	1,732E-04																				
2623	2621	MZT1	2,4	6,830E-04																				
2624	2622	TMEM203	2,4	6,314E-06																				
2625	2623	EIF4E3	2,4	4,559E-03																				
2626	2624	CNP	2,4	2,028E-02																				
2627	2625	CCNJ	2,4	1,269E-03																				
2628	2626	CYB5B	2,4	7,982E-05																				
2629	2627	D2HGDH	2,4	3,337E-03																				
2630	2628	PDE12	2,4	5,987E-05																				
2631	2629	SYNRG	2,4	1,856E-02																				
2632	2630	MBIP	2,4	9,079E-03																				
2633	2631	TP53INP2	2,4	7,086E-05																				
2634	2632	TTBK2	2,4	1,090E-03																				
2635	2633	ZNF44	2,4	1,886E-05																				
2636	2634	ZC3H18	2,4	3,580E-03																				
2637	2635	SASS6	2,4	2,819E-03																				
2638	2636	BCAS3	2,4	2,428E-04																				
2639	2637	LARP7	2,4	3,108E-03																				
2640	2638	CROT	2,4	4,529E-04																				
2641	2639	MSRB1	2,4	9,228E-07																				
2642	2640	LIG4	2,4	1,185E-04																				
2643	2641	ACYP2	2,4	2,286E-04																				
2644	2642	PAFAH2	2,4	9,234E-03																				
2645	2643	USP16	2,4	1,899E-02																				
2646	2644	PIGH	2,4	3,827E-02																				
2647	2645	FAM208A	2,4	5,874E-04																				
2648	2646	CHM	2,4	6,711E-05																				
2649	2647	ITFG2	2,4	6,896E-03																				
2650	2648	ZNF207	2,4	5,081E-06																				
2651	2649	YTHDC1	2,4	2,878E-03																				
2652	2650	CHUK	2,4	1,458E-03																				
2653	2651	AGGF1	2,4	2,535E-04																				
2654	2652	CASKIN2	2,4	9,049E-03																				
2655	2653	ZBTB12	2,4	3,617E-02																				
2656	2654	IL10RB	2,4	4,410E-03																				
2657	2655	TAB2	2,4	6,653E-04																				
2658	2656	KAT8	2,4	1,309E-04																				
2659	2657	MLST8	2,4	2,068E-02																				
2660	2658	ARRB2	2,4	9,159E-04																				
2661	2659	MOV10	2,4	1,857E-05																				
2662	2660	C2CD3	2,4	3,770E-04																				
2663	2661	RUFY3	2,4	2,591E-03																				
2664	2662	EFEMP1	2,4	3,061E-04																				
2665	2663	ZDHHC13	2,4	1,480E-05																				
2666	2664	METTL14	2,4	9,617E-05																				
2667	2665	CCDC120	2,3	5,142E-03																				
2668	2666	GATC	2,3	3,487E-05																				
2669	2667	PTPLAD2	2,3	9,673E-04																				
2670	2668	BDH2	2,3	5,178E-03																				
2671	2669	UHRF1BP1L	2,3	1,079E-05																				
2672	2670	THUMPD1	2,3	1,538E-03																				
2673	2671	BPGM	2,3	1,006E-03																				
2674	2672	RRNAD1	2,3	8,771E-04																				
2675	2673	COX18	2,3	2,810E-02																				
2676	2674	SPRYD4	2,3	1,079E-03																				
2677	2675	DPM2	2,3	3,251E-03																				
2678	2676	DDX23	2,3	2,366E-06																				
2679	2677	CYB561	2,3	6,417E-04																				
2680	2678	RGL2	2,3	7,180E-04																				
2681	2679	NOP14-AS1	2,3	6,567E-03																				
2682	2680	TSC1	2,3	6,885E-05																				
2683	2681	LYPD5	2,3	4,773E-02																				
2684	2682	CRELD1	2,3	3,608E-04																				
2685	2683	CPM	2,3	1,614E-03																				
2686	2684	SPATS2L	2,3	5,759E-04																				
2687	2685	SETD6	2,3	4,961E-04																				
2688	2686	ADIPOR1	2,3	5,020E-04																				
2689	2687	DIRC2	2,3	1,432E-06																				
2690	2688	L3MBTL3	2,3	1,249E-02																				
2691	2689	ZNF655	2,3	1,306E-03																				
2692	2690	SMU1	2,3	5,120E-05																				
2693	2691	TBC1D13	2,3	1,321E-05																				
2694	2692	EIF2D	2,3	2,545E-03																				
2695	2693	CCDC134	2,3	8,645E-03																				
2696	2694	S100A10	2,3	1,047E-06																				
2697	2695	NCAPD3	2,3	1,027E-06																				
2698	2696	QSOX2	2,3	1,821E-05																				
2699	2697	GLYCTK	2,3	4,888E-04																				
2700	2698	XPO5	2,3	6,724E-04																				
2701	2699	DDX11	2,3	3,193E-03																				
2702	2700	LCMT2	2,3	6,310E-05																				
2703	2701	LIN54	2,3	1,486E-02																				
2704	2702	PSMD11	2,3	3,711E-05																				
2705	2703	TMEM2	2,3	1,404E-04																				
2706	2704	SMARCD1	2,3	3,536E-03																				
2707	2705	PCMTD2	2,3	4,296E-04																				
2708	2706	AKAP8L	2,3	4,912E-04																				
2709	2707	TIFA	2,3	1,087E-02																				
2710	2708	SPPL3	2,3	1,117E-04																				
2711	2709	KANSL3	2,3	6,575E-04																				
2712	2710	TIGD6	2,3	4,981E-04																				
2713	2711	MTHFD1	2,3	2,038E-05																				
2714	2712	DHX8	2,3	1,977E-03																				
2715	2713	TTC19	2,3	2,604E-02																				
2716	2714	RPRD1A	2,3	4,523E-04																				
2717	2715	ACBD5	2,3	1,183E-02																				
2718	2716	KBTBD2	2,3	2,655E-05																				
2719	2717	RNASEH2A	2,3	9,687E-05																				
2720	2718	FAM76A	2,3	7,031E-04																				
2721	2719	LITAF	2,3	1,365E-03																				
2722	2720	TMCC1	2,3	1,233E-03																				
2723	2721	VEZF1	2,3	4,251E-05																				
2724	2722	ZNF174	2,3	4,282E-04																				
2725	2723	H3F3B	2,3	4,989E-02																				
2726	2724	PNPT1	2,3	3,777E-04																				
2727	2725	ERAL1	2,3	6,695E-03																				
2728	2726	DCAKD	2,3	2,555E-03																				
2729	2727	DYNLL2	2,3	3,511E-05																				
2730	2728	FAM13B	2,3	2,092E-04																				
2731	2729	TPGS2	2,3	1,052E-02																				
2732	2730	SRR	2,3	1,129E-05																				
2733	2731	PGS1	2,3	4,057E-04																				
2734	2732	CDK19	2,3	7,038E-03																				
2735	2733	LGALS3	2,3	5,680E-04																				
2736	2734	TRIM56	2,3	1,319E-03																				
2737	2735	CAV2	2,3	1,086E-04																				
2738	2736	PLSCR1	2,3	1,741E-04																				
2739	2737	GPD2	2,3	5,147E-03																				
2740	2738	TOMM20	2,3	2,984E-04																				
2741	2739	ZNF200	2,3	8,547E-03																				
2742	2740	ITM2C	2,3	6,206E-05																				
2743	2741	C21orf67	2,3	5,349E-03																				
2744	2742	DNAJA2	2,3	1,346E-05																				
2745	2743	STRADA	2,3	2,052E-04																				
2746	2744	NCBP1	2,3	8,048E-05																				
2747	2745	PPP1R7	2,3	3,322E-04																				
2748	2746	RIPK1	2,3	1,422E-05																				
2749	2747	C14orf142	2,3	2,865E-04																				
2750	2748	EED	2,3	5,994E-04																				
2751	2749	SLC27A5	2,3	3,926E-03																				
2752	2750	TYW1	2,3	2,065E-05																				
2753	2751	FAM179B	2,3	2,934E-04																				
2754	2752	PHLPP2	2,3	3,392E-03																				
2755	2753	TMEM68	2,3	1,800E-05																				
2756	2754	SREK1	2,3	1,508E-02																				
2757	2755	RRP12	2,3	3,513E-03																				
2758	2756	NAV2	2,3	7,763E-03																				
2759	2757	GFOD2	2,3	4,352E-05																				
2760	2758	GART	2,3	4,095E-05																				
2761	2759	MOB1A	2,3	1,215E-04																				
2762	2760	WDTC1	2,3	7,048E-05																				
2763	2761	WDR77	2,3	1,238E-03																				
2764	2762	CD97	2,3	3,397E-05																				
2765	2763	SET	2,3	1,063E-02																				
2766	2764	PRPF4	2,3	2,328E-04																				
2767	2765	PARS2	2,3	3,899E-04																				
2768	2766	PHF13	2,3	7,036E-05																				
2769	2767	SEC23B	2,3	2,043E-07																				
2770	2768	COIL	2,3	9,272E-05																				
2771	2769	MMAB	2,3	1,853E-02																				
2772	2770	LPIN2	2,3	1,077E-05																				
2773	2771	TOE1	2,3	6,223E-04																				
2774	2772	NCL	2,3	2,926E-02																				
2775	2773	NDUFB1	2,3	5,628E-04																				
2776	2774	FKBP4	2,3	1,870E-06																				
2777	2775	LAMTOR3	2,3	1,242E-02																				
2778	2776	PNPLA8	2,3	4,202E-03																				
2779	2777	FAM118B	2,3	2,660E-03																				
2780	2778	CDKN3	2,3	7,187E-05																				
2781	2779	ILF3	2,3	1,507E-04																				
2782	2780	PPP1R21	2,3	1,698E-03																				
2783	2781	ZNF407	2,3	8,830E-04																				
2784	2782	POU2F1	2,3	2,611E-03																				
2785	2783	ZNF830	2,3	1,442E-04																				
2786	2784	SRCAP	2,3	2,226E-03																				
2787	2785	TSC22D1	2,3	1,004E-05																				
2788	2786	RBM47	2,3	1,803E-02																				
2789	2787	KLHDC4	2,3	8,778E-03																				
2790	2788	CLCN3	2,3	7,853E-05																				
2791	2789	NAPG	2,3	6,499E-04																				
2792	2790	ARHGEF9	2,3	6,686E-05																				
2793	2791	ZSCAN12	2,3	1,059E-02																				
2794	2792	PMPCA	2,3	4,330E-04																				
2795	2793	WDR62	2,3	5,744E-03																				
2796	2794	TSFM	2,3	1,088E-03																				
2797	2795	ARHGAP29	2,3	3,662E-04																				
2798	2796	ASCC3	2,3	1,291E-04																				
2799	2797	ARHGAP18	2,3	2,117E-05																				
2800	2798	GCFC2	2,3	9,792E-03																				
2801	2799	TEX2	2,3	3,803E-05																				
2802	2800	CHTOP	2,3	1,023E-04																				
2803	2801	RAD21	2,3	2,086E-03																				
2804	2802	NFYA	2,3	7,273E-07																				
2805	2803	ZCCHC8	2,3	1,619E-02																				
2806	2804	STK32B	2,3	5,589E-03																				
2807	2805	QRSL1	2,3	7,935E-04																				
2808	2806	ATF6	2,3	3,643E-04																				
2809	2807	YWHAE	2,3	1,164E-03																				
2810	2808	AGPAT1	2,3	1,913E-04																				
2811	2809	SLC9A6	2,3	1,582E-06																				
2812	2810	ATAD1	2,3	8,765E-06																				

	A	B	C	D	E	F	G	H	I	J	K	L	M	N	O	P	Q	R	S	T	U	V	W	X
2813	2811	CDK10	2,3	1,241E-02																				
2814	2812	BAIAP2	2,3	1,090E-02																				
2815	2813	SMG7	2,3	1,437E-03																				
2816	2814	SLC38A7	2,3	3,066E-03																				
2817	2815	CTNNAL1	2,3	5,512E-04																				
2818	2816	NET1	2,3	1,086E-03																				
2819	2817	HTR7P1	2,3	1,809E-02																				
2820	2818	SEC16A	2,3	8,040E-05																				
2821	2819	LAP3	2,3	2,595E-05																				
2822	2820	ZNF552	2,3	1,931E-02																				
2823	2821	MEPCE	2,3	3,342E-04																				
2824	2822	PHF3	2,3	2,588E-03																				
2825	2823	EPN2	2,3	4,170E-02																				
2826	2824	C17orf58	2,3	1,779E-04																				
2827	2825	DDX60	2,3	1,731E-03																				
2828	2826	CHST10	2,3	1,378E-04																				
2829	2827	ARHGAP20	2,3	6,637E-03																				
2830	2828	CBR4	2,3	2,515E-03																				
2831	2829	EIF2B1	2,3	3,543E-04																				
2832	2830	INPP5E	2,3	1,223E-02																				
2833	2831	NUP214	2,3	4,100E-04																				
2834	2832	PPP1R3F	2,3	1,148E-02																				
2835	2833	PRR11	2,3	9,207E-04																				
2836	2834	C3orf38	2,3	2,694E-03																				
2837	2835	MDFIC	2,3	7,042E-03																				
2838	2836	TAOK2	2,3	2,967E-03																				
2839	2837	UNC93B1	2,3	4,524E-03																				
2840	2838	MRPS18A	2,3	2,974E-04																				
2841	2839	FANCF	2,3	2,576E-04																				
2842	2840	DYNC1LI2	2,3	1,707E-08																				
2843	2841	TAF3	2,3	5,540E-05																				
2844	2842	B3GALNT2	2,3	3,061E-05																				
2845	2843	LLPH	2,3	3,798E-04																				
2846	2844	DHX32	2,3	9,845E-05																				
2847	2845	PSMD7	2,3	2,399E-05																				
2848	2846	ZNF142	2,3	2,130E-03																				
2849	2847	DNMT3A	2,3	2,518E-03																				
2850	2848	VAMP5	2,3	5,422E-05																				
2851	2849	BBIP1	2,3	4,726E-02																				
2852	2850	XRCC5	2,3	5,706E-03																				
2853	2851	ZNF271	2,3	1,086E-03																				
2854	2852	RBM34	2,3	8,187E-03																				
2855	2853	TUBB2B	2,3	5,273E-03																				
2856	2854	PSMD1	2,3	9,892E-05																				
2857	2855	CHST14	2,3	1,101E-03																				
2858	2856	ZNF740	2,3	5,133E-05																				
2859	2857	PRPF38A	2,3	1,410E-05																				
2860	2858	SMAP2	2,3	8,212E-05																				
2861	2859	OTUD6B	2,3	7,713E-03																				
2862	2860	CMTM3	2,3	4,499E-02																				
2863	2861	DOCK10	2,3	2,123E-03																				
2864	2862	DTNBP1	2,3	1,919E-04																				
2865	2863	SHB	2,3	1,065E-03																				
2866	2864	MUTYH	2,3	1,137E-02																				
2867	2865	FAM127B	2,3	2,127E-03																				
2868	2866	PTX3	2,3	6,955E-05																				
2869	2867	NME7	2,3	1,306E-04																				
2870	2868	USP9Y	2,3	4,218E-03																				
2871	2869	UFC1	2,3	2,068E-04																				
2872	2870	CPSF4	2,3	9,747E-03																				
2873	2871	SS18	2,3	1,587E-02																				
2874	2872	NDUFA12	2,3	2,336E-02																				
2875	2873	HDHD2	2,3	1,078E-03																				
2876	2874	NCSTN	2,3	1,278E-03																				
2877	2875	RAPGEFL1	2,3	2,611E-03																				
2878	2876	MRPL10	2,3	7,043E-03																				
2879	2877	ZC3H7A	2,3	2,142E-04																				
2880	2878	WRAP73	2,3	5,733E-03																				
2881	2879	P2RX4	2,3	4,609E-05																				
2882	2880	MIPEP	2,3	3,498E-04																				
2883	2881	PPP4C	2,3	4,004E-05																				
2884	2882	EHMT2	2,2	1,598E-02																				
2885	2883	TBC1D10B	2,2	1,566E-03																				
2886	2884	TRAIP	2,2	8,465E-04																				
2887	2885	NACA	2,2	4,219E-02																				
2888	2886	ATXN2L	2,2	3,775E-03																				
2889	2887	FBXO28	2,2	2,998E-02																				
2890	2888	MYBL1	2,2	1,832E-04																				
2891	2889	ALAD	2,2	1,300E-04																				
2892	2890	FKBPL	2,2	4,703E-03																				
2893	2891	A1BG-AS1	2,2	3,925E-02																				
2894	2892	HLCS	2,2	3,381E-04																				
2895	2893	KIAA0391	2,2	1,962E-03																				
2896	2894	NTPCR	2,2	2,289E-04																				
2897	2895	KANSL2	2,2	4,506E-03																				
2898	2896	CHMP1A	2,2	3,894E-02																				
2899	2897	SSH2	2,2	1,059E-03																				
2900	2898	TBX15	2,2	2,809E-05																				
2901	2899	ZFAT	2,2	9,193E-03																				
2902	2900	ADIPOR2	2,2	1,129E-04																				
2903	2901	ANK3	2,2	2,934E-02																				
2904	2902	UBQLN1	2,2	1,290E-04																				
2905	2903	PNMAL1	2,2	3,928E-04																				
2906	2904	TEFM	2,2	2,309E-03																				
2907	2905	IRS2	2,2	3,426E-05																				
2908	2906	OSTM1	2,2	2,399E-04																				
2909	2907	SLC35B2	2,2	9,186E-03																				
2910	2908	SYS1	2,2	4,742E-05																				
2911	2909	CCT2	2,2	3,120E-03																				
2912	2910	MFSD11	2,2	4,658E-04																				
2913	2911	KIAA1432	2,2	4,872E-03																				
2914	2912	RAB27B	2,2	1,466E-02																				
2915	2913	RHOB	2,2	1,563E-02																				
2916	2914	ZNF646	2,2	2,264E-04																				
2917	2915	FAM178A	2,2	1,392E-03																				
2918	2916	CTCF	2,2	3,034E-04																				
2919	2917	TOR1A	2,2	1,576E-05																				
2920	2918	AUH	2,2	6,985E-05																				
2921	2919	TXNDC16	2,2	4,535E-04																				
2922	2920	UTP18	2,2	6,170E-05																				
2923	2921	RAD54B	2,2	1,214E-04																				
2924	2922	RCC2	2,2	1,724E-04																				
2925	2923	C2orf44	2,2	2,256E-04																				
2926	2924	TMX1	2,2	2,647E-04																				
2927	2925	RLIM	2,2	1,120E-03																				
2928	2926	PCMT1	2,2	2,363E-04																				
2929	2927	UBE2N	2,2	1,688E-03																				
2930	2928	CXorf56	2,2	6,861E-04																				
2931	2929	KRAS	2,2	1,211E-04																				
2932	2930	GNE	2,2	2,655E-02																				
2933	2931	THAP6	2,2	2,012E-02																				
2934	2932	ELAC1	2,2	7,348E-05																				
2935	2933	TTLL4	2,2	2,610E-03																				
2936	2934	MLLT4	2,2	4,729E-03																				
2937	2935	UBE3B	2,2	1,908E-04																				
2938	2936	ZNF81	2,2	2,808E-02																				
2939	2937	TTC32	2,2	3,479E-03																				
2940	2938	URM1	2,2	3,107E-04																				
2941	2939	REXO4	2,2	3,882E-05																				
2942	2940	ZNF599	2,2	2,992E-03																				
2943	2941	YIPF6	2,2	5,179E-04																				
2944	2942	ZNF569	2,2	2,637E-03																				
2945	2943	DDX52	2,2	1,459E-02																				
2946	2944	LINC00526	2,2	3,548E-04																				
2947	2945	CDCA4	2,2	4,091E-03																				
2948	2946	MRPL28	2,2	5,829E-03																				
2949	2947	SOCS4	2,2	1,269E-02																				
2950	2948	OSGIN2	2,2	4,189E-04																				
2951	2949	RFX5	2,2	4,216E-05																				
2952	2950	C19orf54	2,2	6,373E-04																				
2953	2951	ZKSCAN1	2,2	1,214E-03																				
2954	2952	LRBA	2,2	5,719E-03																				
2955	2953	PRMT7	2,2	1,331E-02																				
2956	2954	DHCR24	2,2	1,095E-04																				
2957	2955	NSUN6	2,2	3,149E-03																				
2958	2956	FAM134A	2,2	1,686E-05																				
2959	2957	ZNF638	2,2	8,585E-03																				
2960	2958	CDV3	2,2	1,201E-05																				
2961	2959	PDE1C	2,2	4,144E-04																				
2962	2960	ADAM15	2,2	8,897E-04																				
2963	2961	DDA1	2,2	2,957E-03																				
2964	2962	BAZ1B	2,2	1,772E-04																				
2965	2963	SNAPC4	2,2	1,567E-02																				
2966	2964	NDUFB5	2,2	1,795E-03																				
2967	2965	WWTR1	2,2	1,419E-05																				
2968	2966	PBX2	2,2	1,886E-03																				
2969	2967	LIFR	2,2	6,754E-04																				
2970	2968	YARS2	2,2	1,208E-04																				
2971	2969	COL11A1	2,2	1,779E-04																				
2972	2970	NF1	2,2	6,954E-05																				
2973	2971	CWC25	2,2	2,427E-05																				
2974	2972	TMEM201	2,2	9,390E-03																				
2975	2973	HNRNPU	2,2	9,889E-07																				
2976	2974	HDX	2,2	2,262E-02																				
2977	2975	SENP2	2,2	3,834E-02																				
2978	2976	ZNF239	2,2	1,043E-02																				
2979	2977	TMEM192	2,2	3,780E-02																				
2980	2978	DHX38	2,2	6,860E-04																				
2981	2979	FOXM1	2,2	2,673E-02																				
2982	2980	TNRC6A	2,2	1,337E-03																				
2983	2981	PLA2G12A	2,2	1,577E-04																				
2984	2982	CMC2	2,2	3,980E-04																				
2985	2983	NUPL2	2,2	2,920E-03																				
2986	2984	ATP5S	2,2	2,138E-02																				
2987	2985	PTTG1IP	2,2	9,365E-06																				
2988	2986	TBC1D15	2,2	2,033E-03																				
2989	2987	LCLAT1	2,2	2,182E-03																				
2990	2988	CROCCP2	2,2	2,692E-02																				
2991	2989	EXOSC3	2,2	1,446E-02																				
2992	2990	BANP	2,2	6,934E-03																				
2993	2991	JKAMP	2,2	7,791E-04																				
2994	2992	CNOT10	2,2	4,981E-05																				
2995	2993	SIRT1	2,2	5,178E-05																				
2996	2994	TRMT12	2,2	5,836E-04																				
2997	2995	ZNF611	2,2	3,834E-04																				
2998	2996	UST	2,2	3,993E-04																				
2999	2997	KCTD7	2,2	3,018E-02																				
3000	2998	PLCXD1	2,2	3,493E-03																				
3001	2999	RSBN1L	2,2	2,841E-03																				
3002	3000	PRUNE	2,2	2,261E-02																				
3003	3001	MRPS25	2,2	5,038E-04																				
3004	3002	PDCL	2,2	3,755E-04																				
3005	3003	MACF1	2,2	8,858E-03																				
3006	3004	THOC1	2,2	1,583E-02																				
3007	3005	ADAL	2,2	4,126E-03																				
3008	3006	STAP2	2,2	2,370E-02																				
3009	3007	ARID2	2,2	3,200E-03																				
3010	3008	SRSF9	2,2	1,411E-03																				
3011	3009	NFE2L2	2,2	8,633E-04																				
3012	3010	DBNDD1	2,2	1,172E-02																				
3013	3011	STARD3NL	2,2	7,547E-04																				
3014	3012	ADAR	2,2	7,943E-05																				

	A	B	C	D
3015	3013	ALMS1	2,2	9,429E-04
3016	3014	CNOT6	2,2	1,579E-04
3017	3015	SKP2	2,2	1,727E-04
3018	3016	PSMB1	2,2	1,642E-05
3019	3017	VPS25	2,2	3,239E-05
3020	3018	NAA40	2,2	8,454E-03
3021	3019	IMPA1	2,2	1,197E-02
3022	3020	SPTSSA	2,2	1,437E-05
3023	3021	PAXIP1	2,2	1,964E-03
3024	3022	CEP76	2,2	1,878E-04
3025	3023	TBC1D24	2,2	1,791E-02
3026	3024	ZNF318	2,2	9,459E-03
3027	3025	PRDX3	2,2	1,832E-03
3028	3026	ATPIF1	2,2	2,452E-02
3029	3027	FAM214A	2,2	4,844E-03
3030	3028	PAFAH1B2	2,2	1,984E-05
3031	3029	SLC24A1	2,2	1,127E-02
3032	3030	ZFYVE27	2,2	4,895E-03
3033	3031	GOLGA1	2,2	1,086E-04
3034	3032	KBTBD7	2,2	3,886E-04
3035	3033	UNC13B	2,2	5,390E-05
3036	3034	KIAA0907	2,2	7,066E-04
3037	3035	DDX3Y	2,2	5,554E-05
3038	3036	HSD17B10	2,2	8,903E-04
3039	3037	TCTA	2,2	4,140E-05
3040	3038	SCN4B	2,2	2,784E-02
3041	3039	MEF2BNB	2,2	2,635E-03
3042	3040	ARL16	2,2	2,239E-03
3043	3041	PHLDB3	2,2	4,133E-02
3044	3042	BLOC1S1	2,2	1,556E-03
3045	3043	COG7	2,2	9,323E-06
3046	3044	THOP1	2,2	2,347E-02
3047	3045	FAM129A	2,2	8,370E-05
3048	3046	BRPF3	2,2	1,062E-03
3049	3047	HS2ST1	2,2	3,684E-04
3050	3048	PLEKHF2	2,2	9,621E-03
3051	3049	SMC4	2,2	4,898E-05
3052	3050	DNAL1	2,2	2,731E-03
3053	3051	KCTD6	2,2	3,036E-03
3054	3052	ZNHIT3	2,2	1,105E-04
3055	3053	SNHG10	2,2	3,195E-03
3056	3054	ATG5	2,2	6,997E-05
3057	3055	KIAA1549	2,2	3,801E-04
3058	3056	XRN1	2,2	1,875E-03
3059	3057	SMCR8	2,2	2,386E-05
3060	3058	EIF2AK2	2,2	4,079E-03
3061	3059	ATL2	2,2	6,610E-04
3062	3060	MRS2	2,2	2,248E-06
3063	3061	BEND3	2,2	1,183E-02
3064	3062	MRPL42	2,2	5,134E-04
3065	3063	ABCB10	2,2	3,202E-03
3066	3064	RNF40	2,2	9,381E-03
3067	3065	BTBD3	2,2	4,893E-03
3068	3066	ZSCAN30	2,2	2,847E-02
3069	3067	TMEM184C	2,2	2,962E-04
3070	3068	MTBP	2,2	1,064E-03
3071	3069	ZNF691	2,2	1,028E-03
3072	3070	DYNLT1	2,2	2,950E-05
3073	3071	UBXN7	2,2	1,412E-04
3074	3072	SLC15A4	2,2	1,129E-02
3075	3073	GAL3ST4	2,2	7,108E-04
3076	3074	AFG3L2	2,2	1,733E-04
3077	3075	GPBP1	2,2	2,771E-04
3078	3076	CRADD	2,2	2,069E-04
3079	3077	NOL11	2,2	5,569E-03
3080	3078	ZMYM1	2,2	1,418E-02
3081	3079	USF1	2,2	3,846E-04
3082	3080	CCDC47	2,2	1,062E-02
3083	3081	ZNF644	2,2	3,940E-04
3084	3082	ZFAND3	2,2	8,766E-03
3085	3083	IFT88	2,2	1,393E-03
3086	3084	CSRNP2	2,2	4,178E-04
3087	3085	SEPT7P2	2,2	1,116E-02
3088	3086	ZCWPW1	2,2	2,489E-02
3089	3087	EWSR1	2,1	4,555E-04
3090	3088	IK	2,1	5,978E-06
3091	3089	PDLIM3	2,1	4,696E-03
3092	3090	POLD1	2,1	2,114E-02
3093	3091	RSBN1	2,1	4,082E-04
3094	3092	ZXDB	2,1	7,912E-04
3095	3093	ZMYM4	2,1	9,063E-04
3096	3094	HEATR3	2,1	8,681E-05
3097	3095	MRRF	2,1	3,822E-04
3098	3096	ZNF786	2,1	1,390E-02
3099	3097	FAM73B	2,1	1,225E-02
3100	3098	TSC22D2	2,1	6,445E-04
3101	3099	BTRC	2,1	5,311E-04
3102	3100	PABPC1L	2,1	1,042E-02
3103	3101	RFC1	2,1	2,413E-04
3104	3102	ZNF879	2,1	5,910E-03
3105	3103	SLC35A1	2,1	2,249E-03
3106	3104	FUBP3	2,1	7,381E-06
3107	3105	TRERF1	2,1	1,255E-04
3108	3106	ZNF446	2,1	1,098E-02
3109	3107	RDX	2,1	1,115E-03
3110	3108	MRPL18	2,1	1,240E-04
3111	3109	ING5	2,1	7,952E-03
3112	3110	KIFAP3	2,1	1,408E-04
3113	3111	SUPV3L1	2,1	2,984E-03
3114	3112	ATXN2	2,1	3,199E-03
3115	3113	TEX30	2,1	4,098E-04
3116	3114	RNF2	2,1	4,663E-04
3117	3115	SRP9	2,1	7,545E-04
3118	3116	SURF6	2,1	6,462E-03
3119	3117	MDM4	2,1	1,221E-03
3120	3118	IKBKB	2,1	5,250E-04
3121	3119	IPO11	2,1	5,755E-06
3122	3120	VBP1	2,1	7,633E-04
3123	3121	ADCY6	2,1	2,108E-02
3124	3122	SPATA2	2,1	1,942E-03
3125	3123	C22orf46	2,1	1,021E-03
3126	3124	SRSF10	2,1	1,510E-04
3127	3125	RAB21	2,1	1,578E-03
3128	3126	COX7B	2,1	1,593E-03
3129	3127	C16orf62	2,1	3,234E-03
3130	3128	DBF4B	2,1	2,365E-05
3131	3129	FBLN7	2,1	6,415E-07
3132	3130	USP14	2,1	4,728E-02
3133	3131	DNAJB14	2,1	3,042E-04
3134	3132	MAT2A	2,1	7,729E-05
3135	3133	COX7A2	2,1	8,980E-05
3136	3134	MED13	2,1	1,200E-03
3137	3135	TRUB2	2,1	5,977E-04
3138	3136	TRAPPC2	2,1	3,493E-03
3139	3137	DNAJC8	2,1	5,340E-05
3140	3138	QRICH2	2,1	1,603E-04
3141	3139	RIT1	2,1	2,888E-05
3142	3140	EMG1	2,1	1,064E-04
3143	3141	C19orf44	2,1	7,981E-04
3144	3142	CRLF3	2,1	1,543E-03
3145	3143	AGFG2	2,1	1,416E-02
3146	3144	UBAP2L	2,1	3,653E-03
3147	3145	ADAMTS13	2,1	2,268E-02
3148	3146	KCTD13	2,1	2,553E-02
3149	3147	DISP1	2,1	1,381E-03
3150	3148	CASD1	2,1	1,746E-03
3151	3149	FBXO8	2,1	3,788E-04
3152	3150	MPPE1	2,1	4,936E-04
3153	3151	SNRPC	2,1	9,240E-06
3154	3152	ATMIN	2,1	2,624E-05
3155	3153	CASK	2,1	8,425E-03
3156	3154	MAGEF1	2,1	5,939E-04
3157	3155	CHMP1B	2,1	7,492E-06
3158	3156	MOXD1	2,1	1,540E-06
3159	3157	SOCS6	2,1	1,594E-05
3160	3158	ATP6V0B	2,1	5,939E-05
3161	3159	C12orf5	2,1	1,514E-03
3162	3160	HNRNPR	2,1	2,081E-02
3163	3161	USP22	2,1	1,337E-04
3164	3162	MARCKSL1	2,1	6,558E-04
3165	3163	KANSL1	2,1	1,917E-05
3166	3164	RNPS1	2,1	9,591E-04
3167	3165	ZNF263	2,1	1,463E-02
3168	3166	PIK3R4	2,1	6,901E-04
3169	3167	CHEK1	2,1	1,812E-03
3170	3168	STX17	2,1	4,114E-03
3171	3169	KIAA0020	2,1	3,859E-04
3172	3170	C8orf33	2,1	2,517E-05
3173	3171	DGKD	2,1	9,233E-04
3174	3172	RPF1	2,1	6,057E-05
3175	3173	PPP6C	2,1	6,800E-05
3176	3174	WDR53	2,1	1,480E-05
3177	3175	LSG1	2,1	1,591E-03
3178	3176	ZZZ3	2,1	4,438E-02
3179	3177	HSP90AA1	2,1	1,781E-05
3180	3178	METTL10	2,1	5,444E-03
3181	3179	TPMT	2,1	7,695E-04
3182	3180	KPNB1	2,1	1,146E-05
3183	3181	SLC31A1	2,1	7,317E-04
3184	3182	RMND1	2,1	4,586E-04
3185	3183	TIMM21	2,1	1,510E-02
3186	3184	CYSTM1	2,1	7,672E-06
3187	3185	PRMT2	2,1	7,855E-04
3188	3186	ATP5O	2,1	1,573E-02
3189	3187	MED12	2,1	2,727E-04
3190	3188	SDC4	2,1	2,808E-05
3191	3189	GPATCH8	2,1	4,452E-05
3192	3190	SYPL1	2,1	3,389E-04
3193	3191	PSKH1	2,1	4,454E-05
3194	3192	DBT	2,1	8,045E-04
3195	3193	SHPRH	2,1	3,907E-04
3196	3194	VPS29	2,1	9,609E-04
3197	3195	THOC6	2,1	8,901E-04
3198	3196	CCDC43	2,1	2,327E-04
3199	3197	BRD8	2,1	3,891E-04
3200	3198	TEX10	2,1	7,406E-05
3201	3199	C2orf43	2,1	2,386E-03
3202	3200	TUBG1	2,1	7,776E-04
3203	3201	VPS52	2,1	3,024E-04
3204	3202	SAP130	2,1	1,158E-03
3205	3203	SELT	2,1	3,718E-04
3206	3204	THBS3	2,1	5,870E-04
3207	3205	MTMR11	2,1	1,048E-03
3208	3206	ODF2L	2,1	1,385E-03
3209	3207	TMEM33	2,1	3,387E-02
3210	3208	C12orf10	2,1	1,608E-03
3211	3209	MRPS10	2,1	1,552E-05
3212	3210	C6orf106	2,1	5,513E-05
3213	3211	CLEC16A	2,1	8,738E-04
3214	3212	C5orf15	2,1	5,724E-04
3215	3213	FAM53B	2,1	3,041E-03
3216	3214	MRPS14	2,1	6,233E-03

	A	B	C	D
3217	3215	DCLRE1C	2,1	3,983E-02
3218	3216	MED20	2,1	1,552E-03
3219	3217	C11orf30	2,1	5,454E-04
3220	3218	C6orf211	2,1	4,395E-03
3221	3219	CD2BP2	2,1	7,093E-04
3222	3220	COPZ1	2,1	8,562E-03
3223	3221	EPHA2	2,1	8,979E-03
3224	3222	HCFC1	2,1	8,062E-03
3225	3223	HAUS5	2,1	9,835E-03
3226	3224	MAP1B	2,1	1,488E-03
3227	3225	PIP4K2C	2,1	2,699E-04
3228	3226	SWT1	2,1	4,670E-02
3229	3227	NEK1	2,1	2,996E-03
3230	3228	ZXDA	2,1	4,765E-04
3231	3229	LTA4H	2,1	3,026E-06
3232	3230	ZNF594	2,1	4,477E-03
3233	3231	PLCD4	2,1	1,731E-03
3234	3232	ATP5B	2,1	3,424E-03
3235	3233	EBP	2,1	1,319E-03
3236	3234	GGA2	2,1	1,916E-03
3237	3235	DTYMK	2,1	3,010E-04
3238	3236	CCDC85C	2,1	9,559E-04
3239	3237	ITFG3	2,1	1,272E-02
3240	3238	DBR1	2,1	4,471E-04
3241	3239	EFTUD2	2,1	2,113E-03
3242	3240	GPN3	2,1	3,743E-04
3243	3241	GATAD1	2,1	3,512E-04
3244	3242	TRUB1	2,1	4,866E-04
3245	3243	EXOC2	2,1	1,487E-04
3246	3244	PHF12	2,1	1,109E-03
3247	3245	WSB1	2,1	3,938E-02
3248	3246	ZRANB2	2,1	1,573E-03
3249	3247	DBF4	2,1	2,720E-04
3250	3248	IFNGR2	2,1	2,919E-06
3251	3249	FLVCR2	2,1	1,898E-02
3252	3250	GCC1	2,1	1,318E-04
3253	3251	MED21	2,1	1,732E-04
3254	3252	WIPI2	2,1	1,839E-03
3255	3253	CAPN5	2,1	1,952E-04
3256	3254	MTRF1	2,1	2,836E-03
3257	3255	RAD18	2,1	1,535E-04
3258	3256	MYL5	2,1	2,965E-02
3259	3257	INVS	2,1	6,880E-04
3260	3258	WHSC1L1	2,1	1,433E-03
3261	3259	PMPCB	2,1	2,142E-04
3262	3260	C2orf76	2,1	4,013E-03
3263	3261	PPIH	2,1	3,238E-04
3264	3262	CENPW	2,1	8,936E-04
3265	3263	GDE1	2,1	2,090E-03
3266	3264	BLM	2,1	1,018E-02
3267	3265	ATP6V0C	2,1	4,076E-02
3268	3266	SLC38A9	2,1	1,581E-04
3269	3267	BCAS2	2,1	5,729E-03
3270	3268	FTSJ3	2,1	1,109E-03
3271	3269	CDK4	2,1	2,113E-04
3272	3270	RBM14	2,1	7,177E-03
3273	3271	RAB11A	2,1	1,814E-02
3274	3272	LGALS8	2,1	1,892E-03
3275	3273	TXNDC12	2,1	3,275E-04
3276	3274	GIT2	2,1	5,473E-05
3277	3275	PPA2	2,1	2,209E-03
3278	3276	PPP4R1	2,1	4,384E-04
3279	3277	SHMT2	2,1	4,523E-05
3280	3278	NCAM1	2,1	6,617E-03
3281	3279	EPM2A	2,1	1,979E-03
3282	3280	TMTC4	2,1	3,471E-03
3283	3281	PAN2	2,1	3,267E-03
3284	3282	ZNF266	2,1	2,749E-02
3285	3283	ATG4A	2,1	5,614E-04
3286	3284	HPRT1	2,1	2,377E-04
3287	3285	SF3B5	2,1	1,793E-05
3288	3286	LUC7L3	2,1	1,549E-04
3289	3287	ANTXR1	2,1	3,012E-04
3290	3288	PARG	2,1	4,502E-04
3291	3289	ZNF37BP	2,1	3,294E-04
3292	3290	YY1AP1	2,1	5,493E-03
3293	3291	IRAK4	2,1	6,741E-03
3294	3292	ALKBH1	2,1	1,496E-02
3295	3293	C9orf114	2,1	1,877E-04
3296	3294	CCDC102A	2,1	1,661E-03
3297	3295	ZNF627	2,1	2,648E-02
3298	3296	DNM1	2,1	1,523E-03
3299	3297	SMO	2,0	3,853E-04
3300	3298	MRPS23	2,0	3,186E-04
3301	3299	ITFG1	2,0	6,940E-05
3302	3300	VPS39	2,0	2,374E-05
3303	3301	COMMD4	2,0	4,998E-03
3304	3302	CDK16	2,0	2,134E-02
3305	3303	SNX29	2,0	1,095E-03
3306	3304	PELP1	2,0	1,363E-02
3307	3305	CPSF6	2,0	7,098E-04
3308	3306	FAM13C	2,0	8,391E-03
3309	3307	HNRNPD	2,0	2,176E-02
3310	3308	PSMB4	2,0	9,370E-06
3311	3309	NFE2L1	2,0	5,620E-06
3312	3310	CNOT4	2,0	9,268E-05
3313	3311	OPA1	2,0	2,728E-03
3314	3312	SNAP47	2,0	2,454E-04
3315	3313	MED30	2,0	6,491E-05
3316	3314	ZNF451	2,0	1,766E-04
3317	3315	ARL5B	2,0	2,955E-03
3318	3316	THAP5	2,0	1,110E-04
3319	3317	ELF2	2,0	4,127E-04
3320	3318	FBXO30	2,0	2,028E-03
3321	3319	MTIF2	2,0	5,286E-04
3322	3320	NDUFA9	2,0	1,725E-04
3323	3321	SPOP	2,0	3,031E-03
3324	3322	RBM19	2,0	1,834E-03
3325	3323	STK16	2,0	2,907E-03
3326	3324	SMAD4	2,0	8,689E-04
3327	3325	ATP6V1B2	2,0	2,949E-05
3328	3326	HELZ	2,0	9,723E-03
3329	3327	CTSH	2,0	3,070E-04
3330	3328	CTNS	2,0	2,293E-04
3331	3329	POLR3B	2,0	7,810E-04
3332	3330	KHDRBS1	2,0	1,748E-04
3333	3331	TGFB2	2,0	1,398E-06
3334	3332	ZCCHC11	2,0	4,949E-04
3335	3333	IFT172	2,0	4,172E-04
3336	3334	C5orf34	2,0	8,143E-03
3337	3335	PYROXD1	2,0	4,871E-03
3338	3336	ATP5A1	2,0	2,388E-03
3339	3337	PPID	2,0	2,170E-04
3340	3338	EIF4EBP2	2,0	3,551E-05
3341	3339	MLF2	2,0	5,385E-03
3342	3340	NUP88	2,0	5,518E-05
3343	3341	DDAH1	2,0	8,241E-06
3344	3342	CCDC84	2,0	1,239E-03
3345	3343	POLR3E	2,0	4,770E-03
3346	3344	HAX1	2,0	6,007E-03
3347	3345	ABCB6	2,0	4,617E-03
3348	3346	HMBOX1	2,0	4,934E-04
3349	3347	ESCO1	2,0	1,410E-04
3350	3348	PBX3	2,0	2,842E-03
3351	3349	SNN	2,0	2,118E-05
3352	3350	MIB1	2,0	6,148E-05
3353	3351	TM2D1	2,0	8,403E-05
3354	3352	HIAT1	2,0	9,205E-05
3355	3353	POLR2A	2,0	9,986E-05
3356	3354	ARPC1A	2,0	6,794E-05
3357	3355	WDR60	2,0	6,144E-04
3358	3356	DDX24	2,0	1,196E-03
3359	3357	RBM23	2,0	2,538E-03
3360	3358	PRMT10	2,0	1,623E-03
3361	3359	DENR	2,0	4,053E-04
3362	3360	MFF	2,0	2,521E-04
3363	3361	FAIM	2,0	3,207E-04
3364	3362	ALG1	2,0	8,688E-03
3365	3363	TACO1	2,0	1,462E-02
3366	3364	TSEN2	2,0	2,806E-02
3367	3365	RFK	2,0	2,781E-04
3368	3366	PPP1R10	2,0	1,625E-03
3369	3367	B4GALT4	2,0	5,241E-03
3370	3368	TRIAP1	2,0	7,080E-04
3371	3369	CCT8	2,0	5,257E-06
3372	3370	ASXL1	2,0	7,833E-05
3373	3371	DTX2	2,0	9,346E-03
3374	3372	NVL	2,0	1,744E-03
3375	3373	ARMCX5	2,0	5,727E-03
3376	3374	VPS41	2,0	1,502E-04
3377	3375	RNF168	2,0	5,785E-04
3378	3376	SMARCA5	2,0	1,141E-04
3379	3377	RWDD3	2,0	8,507E-04
3380	3378	ARHGAP5-AS1	2,0	7,278E-03
3381	3379	TRAP1	2,0	5,976E-03
3382	3380	C10orf2	2,0	7,399E-04
3383	3381	WDR44	2,0	1,647E-03
3384	3382	ZNF264	2,0	3,328E-03
3385	3383	AKAP6	2,0	2,592E-02
3386	3384	ZBTB40	2,0	5,932E-05
3387	3385	ERP44	2,0	9,210E-05
3388	3386	CDK12	2,0	4,501E-03
3389	3387	ZDHHC17	2,0	2,235E-02
3390	3388	ZNF275	2,0	1,308E-02
3391	3389	CYCS	2,0	8,644E-03
3392	3390	SPRY2	2,0	3,621E-03
3393	3391	UBAP1	2,0	6,815E-05
3394	3392	DNAJC16	2,0	2,665E-02
3395	3393	RECQL5	2,0	2,362E-03
3396	3394	PHF8	2,0	4,587E-03
3397	3395	TRPC1	2,0	2,265E-03
3398	3396	FGFRL1	2,0	2,391E-02
3399	3397	ANGEL2	2,0	1,318E-04
3400	3398	TAPT1	2,0	4,599E-04
3401	3399	SDR39U1	2,0	1,259E-02
3402	3400	FUCA2	2,0	3,118E-05
3403	3401	HINFP	2,0	7,539E-04
3404	3402	ATG14	2,0	3,447E-04
3405	3403	ACAD9	2,0	7,640E-04
3406	3404	ZNF669	2,0	1,828E-03
3407	3405	TP53BP1	2,0	1,023E-04
3408	3406	RTF1	2,0	6,441E-04
3409	3407	XRCC4	2,0	3,605E-04
3410	3408	KBTBD6	2,0	2,611E-04
3411	3409	CERS5	2,0	3,623E-03
3412	3410	KATNA1	2,0	3,228E-03
3413	3411	MRP63	2,0	4,357E-03
3414	3412	UBB	2,0	6,707E-04

Supplemental Table 4. Identification of altered phosphopeptide levels by PamGene analysis. PamGene data was retrieved from the PamGene station. The first column indicates the spotted peptides for corresponding phospho-sites in the denominated proteins. The value in columns 2 to 10 show normalized relative peptide phosphorylation levels for triplicate measurements of cell lysates derived from U-2982-control, U-2982-SOX2 and U-2987-control. High value indicates detection of high levels of phosphorylated tyrosine residue for the corresponding peptides.

Description	U2982-ctrl-1	U2982-ctrl-2	U2982-ctrl-3	U2982-SOX2-1	U2982-SOX2-2	U2982-SOX2-3	U2987-ctrl-1	U2987-ctrl-2	U2987-ctrl-3
CD3Z_146_158	5,28	5,34	4,95	7,36	7,68	7,62	7,90	8,00	7,05
STAT4_714_726	5,40	5,48	5,54	7,50	7,46	7,54	7,84	7,70	7,65
CD3Z_116_128	6,98	8,43	5,87	9,60	9,51	9,54	9,54	9,60	9,32
SRC8_CHICK_470_482	4,75	4,70	4,80	6,32	6,55	6,27	6,24	6,53	6,09
DYR1A_212_224	4,92	4,59	4,46	6,25	6,17	6,33	5,96	5,77	6,54
EGFR_862_874	4,75	5,15	4,77	6,42	6,84	6,24	6,30	6,29	5,85
ANXA2_17_29	6,68	6,39	6,21	7,88	8,25	8,07	8,06	7,93	7,90
VGFR1_1040_1052	6,25	6,02	6,20	7,26	7,38	7,13	7,46	7,26	8,19
P85A_600_612	11,42	11,63	11,53	13,32	13,40	13,38	13,14	13,24	12,91
VGFR1_1320_1332_C1320S/C1	5,40	5,54	5,81	6,43	6,42	6,46	6,36	6,29	7,28
LCK_387_399	8,80	9,28	9,11	10,46	10,43	10,41	10,33	10,30	10,44
RBL2_99_111	5,56	5,53	5,37	6,11	6,31	6,33	6,42	6,43	6,24
EPHA1_774_786	10,89	10,84	10,97	12,50	12,41	12,32	12,10	12,16	12,01
ERBB2_870_882	7,82	7,92	7,77	9,02	8,86	8,80	8,82	8,75	8,41
EGFR_1103_1115	8,16	8,20	7,80	9,12	9,09	9,17	9,01	8,98	8,66
EPHB1_771_783	10,09	10,33	10,36	11,59	11,58	11,49	11,55	11,48	11,39
CTNB1_79_91	7,64	8,25	8,09	8,97	8,94	8,93	9,16	9,30	8,94
EPHA7_607_619	10,18	10,20	10,26	11,50	11,44	11,34	11,53	11,48	11,34
EPOR_419_431	9,83	9,60	9,55	10,84	10,71	10,78	10,54	10,52	10,56
EPHA2_765_777	11,23	11,37	11,51	12,65	12,68	12,64	12,47	12,46	12,25
FES_706_718	10,89	11,19	11,08	12,32	12,30	12,27	12,16	12,23	12,17
ERBB4_1181_1193	5,51	5,62	5,69	6,33	6,22	6,10	6,53	6,25	6,07
PAXI_111_123	12,08	12,21	12,07	13,40	13,48	13,46	13,16	13,24	13,14
PDPK1_369_381	8,64	8,98	9,00	9,81	9,83	9,88	9,62	9,65	9,83
PECA1_706_718	11,17	11,21	10,97	12,25	12,34	12,41	12,14	12,08	12,29
PAXI_24_36	11,25	11,35	11,32	12,57	12,50	12,54	12,41	12,37	12,54
FER_707_719	9,94	10,07	10,06	11,06	11,17	11,10	11,07	11,06	11,00
PGFRB_1002_1014	8,72	8,90	8,70	9,69	9,74	9,73	9,90	9,88	9,64
EPOR_361_373	10,19	10,29	10,29	11,37	11,37	11,31	11,18	11,21	11,08
RET_1022_1034	11,03	11,34	11,24	12,39	12,38	12,30	12,27	12,30	12,18
PGFRB_572_584	11,72	11,67	11,77	12,87	12,89	12,92	12,81	12,90	12,42
B3AT_39_51	5,89	6,41	6,15	6,61	6,79	6,80	6,90	6,90	6,21
VGFR2_989_1001	11,44	11,45	11,54	12,53	12,58	12,55	12,32	12,25	12,42
JAK1_1015_1027	9,57	9,48	9,73	10,52	10,53	10,41	10,49	10,58	10,27
ACHD_383_395	6,68	5,56	6,30	6,88	6,65	6,64	6,58	6,59	6,98
RASA1_453_465	10,35	10,35	10,27	11,18	11,23	11,26	11,22	11,19	10,84
CDK2_8_20	11,83	11,75	11,81	12,79	12,76	12,75	12,54	12,65	12,92
FRK_380_392	12,53	12,54	12,54	13,52	13,57	13,57	13,20	13,24	13,33
PLCG1_764_776	12,77	12,96	12,95	13,85	13,91	13,98	13,69	13,69	13,67
ZAP70_485_497	9,12	9,34	9,32	10,06	9,94	9,96	10,15	10,15	10,52
PGFRB_771_783	8,59	8,79	8,54	9,35	9,25	9,32	9,16	9,15	9,33
CBL_693_705	7,72	8,21	8,01	8,58	8,59	8,60	9,16	9,25	8,74
ANXA1_14_26	9,23	9,41	9,12	10,04	9,95	9,87	10,14	10,18	9,75
K2C8_425_437	7,48	7,23	7,27	7,77	7,97	7,82	8,04	7,92	7,98
EGFR_1118_1130	5,77	5,71	5,64	6,06	6,21	5,97	6,36	6,39	6,59
JAK2_563_577	9,19	9,33	9,03	9,84	9,73	9,71	9,76	9,79	9,41
PRGR_786_798	8,19	8,36	8,48	8,82	8,91	8,87	8,68	8,52	8,73
ODPAT_291_303	5,72	5,85	6,02	6,18	6,22	6,13	5,85	5,91	5,66
41_654_666	10,74	10,85	10,91	11,36	11,44	11,40	11,34	11,31	11,13
ODBA_340_352	7,54	7,04	7,37	7,72	7,70	7,66	8,01	8,02	8,30
PDPK1_2_14	10,14	10,22	9,94	10,56	10,52	10,64	10,72	10,71	10,72
LAT_249_261	10,32	10,46	10,60	10,92	10,93	10,87	10,81	10,79	11,29
ERBB2_1241_1253	8,59	9,02	8,92	9,26	9,19	9,20	9,26	9,23	9,10
PGFRB_768_780	8,68	8,56	8,77	8,94	9,01	8,99	8,89	8,99	8,95
PGFRB_1014_1028	9,66	9,71	10,20	10,09	10,07	10,02	10,11	10,17	10,06
VGFR2_1046_1058	7,01	7,53	7,37	7,42	7,48	7,39	7,67	7,81	7,64
TEC_512_524	8,78	8,96	8,89	9,07	8,94	8,99	8,97	8,96	8,87
CALM_95_107	6,66	7,46	7,23	7,08	7,18	7,24	7,11	7,24	6,81
VGFR2_1052_1064	7,63	7,73	8,32	8,07	7,92	7,84	8,38	8,46	7,99
MET_1227_1239	10,69	10,42	10,46	10,64	10,41	10,59	10,56	10,51	10,38
VGFR1_1326_1338	9,30	9,07	9,25	9,17	9,14	9,11	9,41	9,43	9,31
VGFR3_1061_1073	7,58	7,00	7,09	7,16	7,30	7,04	7,39	7,23	7,32
PGFRB_709_721	9,22	9,31	9,62	9,25	9,27	9,29	9,51	9,48	9,02
FAK2_572_584	10,72	10,69	10,82	10,60	10,60	10,58	10,65	10,68	10,65
C1R_199_211	5,96	6,61	6,23	6,16	6,19	6,13	6,30	6,18	5,88
NTRK2_696_708	10,05	9,93	9,72	9,74	9,62	9,76	9,53	9,51	9,07
RON_1346_1358	8,85	9,01	9,06	8,70	8,87	8,65	9,13	9,01	9,10
MK07_211_223	8,41	8,47	7,88	8,07	7,57	8,16	7,77	7,88	7,52
STAT3_698_710	6,81	6,08	6,71	6,39	6,06	6,39	6,30	6,31	6,17
PRRX2_202_214	10,77	10,63	10,60	10,16	10,25	10,30	10,14	10,07	10,16

RAF1_332_344	11,00	10,80	10,74	10,32	10,36	10,44	10,18	10,23	10,41
EPHA4_589_601	7,95	6,76	6,53	6,75	6,73	6,79	6,52	6,72	6,64
FAK1_569_581	9,22	9,13	9,24	8,73	8,67	8,76	8,61	8,63	9,07
RON_1353_1365	8,73	8,60	9,33	8,43	8,44	8,34	8,48	8,32	8,19
MK14_173_185	7,35	6,66	7,02	6,72	6,57	6,54	6,72	6,78	6,54
VGFR1_1162_1174	6,05	6,78	5,97	5,78	5,87	6,02	6,07	6,19	6,10
CRK_214_226	7,74	7,99	7,45	7,16	7,35	7,20	7,84	7,87	7,18
FGFR1_761_773	7,94	8,34	8,06	7,53	7,54	7,70	7,77	7,77	7,96
K2C6B_53_65	10,16	9,78	9,79	9,23	9,13	9,31	9,09	9,15	8,98
DCX_109_121	9,43	9,04	9,10	8,67	8,38	8,62	8,62	8,49	8,39
ART_004_EAIYAAPFAKKKXC	13,11	13,12	13,15	12,18	12,22	12,22	12,39	12,54	12,27
NTRK1_489_501	5,78	6,68	6,71	5,99	6,05	5,77	5,97	6,00	5,71
EPHB4_583_595	6,90	7,03	6,39	6,40	6,32	6,13	6,38	6,36	6,77
EPHB1_921_933	7,80	7,18	8,05	7,19	6,95	7,19	6,50	6,62	7,04
DYR1A_312_324	9,20	8,73	9,08	8,40	8,31	8,20	8,11	8,07	8,85
MK01_180_192	8,93	7,80	8,38	7,69	7,57	7,83	7,69	7,71	7,82
RB_804_816	9,19	8,34	9,15	8,22	8,01	8,26	8,07	7,93	8,45
FGFR3_753_765	9,73	9,76	10,10	9,02	9,02	9,01	9,07	9,14	9,09
MK10_216_228	10,04	9,97	9,86	9,05	9,00	9,19	8,88	8,87	8,62
EGFR_1190_1202	7,45	7,53	7,09	6,62	6,65	6,79	6,99	7,10	6,86
MBP_263_275	7,74	6,05	6,84	6,19	6,08	6,26	5,92	5,72	5,57
MK12_178_190	8,48	8,14	8,58	7,41	7,64	7,52	7,51	7,56	7,52
NCF1_313_325	11,02	10,95	10,70	9,72	9,72	9,80	9,48	9,46	9,77
VGFR1_1206_1218	7,31	6,74	7,00	6,40	6,30	6,13	6,11	6,18	5,88
CDK7_157_169	8,99	8,81	8,70	7,90	7,76	7,84	7,46	7,47	7,81
MBP_198_210	8,34	8,88	8,42	7,53	7,62	7,56	7,80	7,57	7,28
LAT_194_206	8,93	8,71	8,59	7,71	7,70	7,76	7,63	7,79	7,90
TYRO3_679_691	11,04	10,76	11,17	9,66	9,69	9,76	9,36	9,26	9,78
FGFR2_762_774	10,04	10,21	10,18	8,90	9,03	8,88	9,05	9,04	9,18
ERBB4_1277_1289	8,46	8,84	8,98	7,60	7,67	7,63	7,59	7,49	7,40
VGFR2_1168_1180	8,50	8,48	8,47	7,35	7,44	7,31	7,60	7,67	8,05
EGFR_1165_1177	9,12	9,20	9,33	7,99	7,98	7,93	8,00	7,99	8,31
INSR_992_1004	6,77	8,03	7,59	6,53	6,17	6,48	6,60	6,55	6,55
VINC_815_827	8,59	8,45	8,47	7,21	7,27	7,37	6,89	7,09	7,72
KSYK_518_530	8,27	8,92	8,59	7,24	7,44	7,12	7,46	7,44	7,45
PP2AB_297_309	8,73	8,41	8,51	6,99	7,29	7,32	6,87	6,72	6,91
MBP_259_271	7,67	8,11	7,77	6,53	6,22	6,92	6,25	6,50	6,65
NPT2A_501_513	9,56	9,05	8,95	7,56	7,64	7,77	7,10	6,83	7,64
ZBT16_621_633	9,72	9,51	9,95	7,95	8,13	8,07	7,63	7,58	7,72
PTN11_539_551	8,61	8,79	8,35	7,11	7,05	7,13	6,79	6,73	7,04
VGFR2_944_956	10,00	9,82	9,85	7,84	8,01	7,89	7,68	7,56	7,97
ART_003_EAI(pY)AAPFAKKK	14,52	14,40	14,46	11,39	11,54	11,40	11,25	11,38	11,48

Supplemental Table 5. Proteins identified by mass spectrometry analysis of SFRP2 co-immunoprecipitated proteins. *SFRP2 is highlighted in orange.*

**Protein accession**	Accession number from Uniprot
**Description**	Description including protein name and Gene name
**Coverage**	Percent of protein covered by the identified peptides. Mass spectrometry analysis does not detect all peptides, hence is not a sequencing tool. High abundant proteins generally results in a high coverage.
**# Proteins**	Number of other proteins that also contain some of the identified peptides
**# Unique peptides**	The number of unique peptides mapping to the protein. One or several unique peptide detected for one protein decrease the probability of it being a "false discovery".
**# Peptides**	Total Number of peptides mapping to the protein.
**# PSMs**	Number of Peptide Spectral Matches. The total number of peptide spectrums matched to the protein. A high PSM number often corresponds to a high abundant protein.
**MS1 precursor are**	The average peak area of the three most intense peptides including unique and non unique peptides. A large area and a high number of PSM´s indicate that the protein is high abundant. However, peptides are different, hence ionize and "fly" differently depending on physical properties. Therefor a poor ionized peptide can be high abundant and vice versa.
**Q-value**	False Discovery Rate. Some matches will be false discoveries but the data analysis algoritm will use a decoy database to exclude most FDR´s. The false discovery rate is often set to 1% at peptide level during data analysis.

Accession	Description	ΣCoverage	Σ# Proteins	Unique Pepti	Σ# Peptides	Σ# PSMs	A5: Area	B5: Area	Score A2	Coverage A2	# Peptides A2	# PSM A2	Score B2	Coverage B2	# Peptides B2	# PSM B2	# AAs	MW [kDa]	calc. pI
P35579	Myosin-9 OS=Homo sapiens OX=9606 GN=MYH9 PE=1 SV=4 - [MYH9_HUMAN]	54,74	9	89	106	1292	2,339E9	2,613E10	559,74	40,61	78	269	2629,65	53,98	100	1023	1960	226,4	5,60
P35580	Myosin-10 OS=Homo sapiens OX=9606 GN=MYH10 PE=1 SV=3 - [MYH10_HUMAN]	48,08	10	70	84	291	4,704E8	1,442E10	152,75	28,19	48	77	555,98	44,84	78	214	1976	228,9	5,54
P60709	Actin, cytoplasmic 1 OS=Homo sapiens OX=9606 GN=ACTB PE=1 SV=1 - [ACTB_HUMAN]	66,40	7	9	17	225	2,196E9	2,796E10	92,26	54,67	15	56	392,69	60,27	16	169	375	41,7	5,48
Q7Z406	Myosin-14 OS=Homo sapiens OX=9606 GN=MYH14 PE=1 SV=2 - [MYH14_HUMAN]	20,50	9	26	35	181	6,723E8	1,538E10	81,77	8,57	18	41	297,69	18,70	31	140	1995	227,7	5,60
P08670	Vimentin OS=Homo sapiens OX=9606 GN=VIM PE=1 SV=4 - [VIME_HUMAN]	54,72	7	22	22	158	5,601E8	5,704E9	76,38	47,21	19	42	252,40	54,72	22	116	466	53,6	5,12
P68032	Actin, alpha cardiac muscle 1 OS=Homo sapiens OX=9606 GN=ACTC1 PE=1 SV=1 - [ACTC_HUMAN]	44,30	4	5	13	176	1,364E9	1,869E10	51,53	36,07	11	43	242,67	44,30	13	133	377	42,0	5,39
Q63HN8	E3 ubiquitin-protein ligase RNF213 OS=Homo sapiens OX=9606 GN=RNF213 PE=1 SV=3 - [RN213_HUM	16,71	3	76	76	83	0,000E0	9,967E7		0,00			182,48	16,71	76	83	5207	591,0	6,48
P04264	Keratin, type II cytoskeletal 1 OS=Homo sapiens OX=9606 GN=KRT1 PE=1 SV=6 - [K2C1_HUMAN]	53,11	2	26	32	275	7,436E9	2,018E9	463,29	50,93	31	222	166,36	42,08	20	53	644	66,0	8,12
P21333	Filamin-A OS=Homo sapiens OX=9606 GN=FLNA PE=1 SV=4 - [FLNA_HUMAN]	29,05	1	53	55	72	7,955E6	2,164E8	32,27	10,54	19	19	140,94	24,03	46	53	2647	280,6	6,06
P49792	E3 SUMO-protein ligase RanBP2 OS=Homo sapiens OX=9606 GN=RANBP2 PE=1 SV=2 - [RBP2_HUMA	18,98	7	47	47	53	0,000E0	2,373E8		0,00			128,47	18,98	47	53	3224	358,0	6,20
P09493	Tropomyosin alpha-1 chain OS=Homo sapiens OX=9606 GN=TPM1 PE=1 SV=2 - [TPM1_HUMAN]	38,38	1	7	12	70	1,992E8	4,165E9	36,32	25,00	7	18	123,53	35,21	11	52	284	32,7	4,74
P19105	Myosin regulatory light chain 12A OS=Homo sapiens OX=9606 GN=MYL12A PE=1 SV=2 - [ML12A_HUI	59,06	2	4	9	68	3,373E8	8,414E9	12,08	29,82	5	11	118,62	59,06	9	57	171	19,8	4,81
Q15149	Plectin OS=Homo sapiens OX=9606 GN=PLEC PE=1 SV=3 - [PLEC_HUMAN]	15,52	1	56	60	72	5,041E7	2,274E7	25,02	5,06	23	23	110,29	12,19	45	49	4684	531,5	5,96
Q52LW3	Rho GTPase-activating protein 29 OS=Homo sapiens OX=9606 GN=ARHGAP29 PE=1 SV=2 - [RHG29_H	28,47	1	29	29	44	0,000E0	2,322E8		0,00			110,14	28,47	29	44	1261	142,0	6,74
Q6WCQ1	Myosin phosphatase Rho-interacting protein OS=Homo sapiens OX=9606 GN=MPRIP PE=1 SV=3 - [MPR	42,93	1	32	33	46	1,043E7	1,506E8	12,65	11,41	9	9	108,96	42,93	33	37	1025	116,5	6,21
P35527	Keratin, type I cytoskeletal 9 OS=Homo sapiens OX=9606 GN=KRT9 PE=1 SV=3 - [K1C9_HUMAN]	64,37	2	23	24	103	2,021E9	9,773E8	135,21	55,06	21	68	104,01	44,78	17	35	623	62,0	5,24
P24928	DNA-directed RNA polymerase II subunit RPB1 OS=Homo sapiens OX=9606 GN=POLR2A PE=1 SV=2 -	17,26	1	29	29	41	0,000E0	4,519E8		0,00			102,60	17,26	29	41	1970	217,0	7,37
Q9UQ35	Serine/arginine repetitive matrix protein 2 OS=Homo sapiens OX=9606 GN=SRRM2 PE=1 SV=2 - [SRRM	15,26	1	27	27	32	0,000E0	2,307E8		0,00			101,90	15,26	27	32	2752	299,4	12,06
O00159	Unconventional myosin-Ic OS=Homo sapiens OX=9606 GN=MYO1C PE=1 SV=4 - [MYO1C_HUMAN]	35,28	1	33	33	60	3,154E7	3,600E8	24,05	17,22	17	18	99,36	30,86	28	42	1063	121,6	9,41
P24844	Myosin regulatory light polypeptide 9 OS=Homo sapiens OX=9606 GN=MYL9 PE=1 SV=4 - [MYL9_HUM	58,72	1	3	8	58	3,091E8	8,414E9	7,20	29,65	5	9	97,71	58,72	8	49	172	19,8	4,92
Q13263	Transcription intermediary factor 1-beta OS=Homo sapiens OX=9606 GN=TRIM28 PE=1 SV=5 - [TIF1B_H	35,09	1	18	18	42	2,808E7	1,287E9	6,74	4,31	2	3	97,30	35,09	18	39	835	88,5	5,77
P06753	Tropomyosin alpha-3 chain OS=Homo sapiens OX=9606 GN=TPM3 PE=1 SV=2 - [TPM3_HUMAN]	32,63	1	4	13	60	1,509E8	2,597E9	30,32	18,25	6	16	96,98	29,12	11	44	285	32,9	4,72
P60660	Myosin light polypeptide 6 OS=Homo sapiens OX=9606 GN=MYL6 PE=1 SV=2 - [MYL6_HUMAN]	54,30	3	5	7	49	3,947E8	9,476E9	21,36	36,42	5	10	96,69	54,30	7	39	151	16,9	4,65
O43795	Unconventional myosin-Ib OS=Homo sapiens OX=9606 GN=MYO1B PE=1 SV=3 - [MYO1B_HUMAN]	29,14	3	29	29	56	2,782E7	3,852E8	18,15	13,20	14	14	92,00	28,52	28	42	1136	131,9	9,38
P11021	Endoplasmic reticulum chaperone BiP OS=Homo sapiens OX=9606 GN=HSPA5 PE=1 SV=2 - [BIP_HUMA	42,81	2	19	21	49	6,822E7	6,010E8	25,46	34,40	17	19	89,98	37,16	17	30	654	72,3	5,16
Q69YQ0	Cytospin-A OS=Homo sapiens OX=9606 GN=SPECC1L PE=1 SV=2 - [CYTSA_HUMAN]	26,23	1	25	25	39	9,771E6	1,472E8	6,95	6,27	6	6	81,97	26,23	25	33	1117	124,5	5,72
P11142	Heat shock cognate 71 kDa protein OS=Homo sapiens OX=9606 GN=HSPA8 PE=1 SV=1 - [HSP7C_HUMA	38,70	2	17	20	46	7,110E7	4,939E8	38,49	27,71	14	20	73,75	28,64	14	26	646	70,9	5,52
Q8WWI1	LIM domain only protein 7 OS=Homo sapiens OX=9606 GN=LMO7 PE=1 SV=3 - [LMO7_HUMAN]	24,42	1	32	32	39	4,368E6	8,307E7	13,25	4,99	7	7	73,42	21,57	28	32	1683	192,6	8,09
P35908	Keratin, type II cytoskeletal 2 epidermal OS=Homo sapiens OX=9606 GN=KRT2 PE=1 SV=2 - [K22E_HUM	43,04	3	20	26	115	3,683E9	1,466E9	187,14	41,63	25	87	65,37	25,82	15	28	639	65,4	8,00
P13645	Keratin, type I cytoskeletal 10 OS=Homo sapiens OX=9606 GN=KRT10 PE=1 SV=6 - [K1C10_HUMAN]	42,81	15	19	25	123	4,590E9	9,767E8	219,53	41,78	24	99	64,21	27,91	16	24	584	58,8	5,21
P07437	Tubulin beta chain OS=Homo sapiens OX=9606 GN=TUBB PE=1 SV=2 - [TBB5_HUMAN]	43,02	4	3	14	45	1,703E8	4,600E8	52,70	28,38	9	22	63,63	42,79	13	23	444	49,6	4,89
P68371	Tubulin beta-4B chain OS=Homo sapiens OX=9606 GN=TUBB4B PE=1 SV=1 - [TBB4B_HUMAN]	42,92	5	4	14	38	9,199E7	4,472E8	35,25	22,25	7	15	63,30	42,70	13	23	445	49,8	4,89
O43707	Alpha-actinin-4 OS=Homo sapiens OX=9606 GN=ACTN4 PE=1 SV=2 - [ACTN4_HUMAN]	37,87	1	18	28	40	1,312E7	7,681E7	21,07	20,53	15	17	61,93	27,55	21	23	911	104,8	5,44
P30876	DNA-directed RNA polymerase II subunit RPB2 OS=Homo sapiens OX=9606 GN=POLR2B PE=1 SV=1 -	22,74	1	21	21	25	0,000E0	2,963E8		0,00			61,91	22,74	21	25	1174	133,8	6,87
Q96N67	Dedicator of cytokinesis protein 7 OS=Homo sapiens OX=9606 GN=DOCK7 PE=1 SV=4 - [DOCK7_HUMA	15,56	2	23	26	27	5,106E5	5,299E7	0,00	0,65	1	1	59,62	15,56	26	26	2140	242,4	6,80
Q16643	Drebrin OS=Homo sapiens OX=9606 GN=DBN1 PE=1 SV=4 - [DREB_HUMAN]	29,12	1	14	14	28	7,820E7	6,382E8	15,05	17,72	8	10	56,57	25,58	11	18	649	71,4	4,45
P12814	Alpha-actinin-1 OS=Homo sapiens OX=9606 GN=ACTN1 PE=1 SV=2 - [ACTN1_HUMAN]	31,84	2	13	23	32	5,375E6	8,008E7	12,55	14,91	9	10	56,45	26,01	21	22	892	103,0	5,41
Q0ZGT2	Nexilin OS=Homo sapiens OX=9606 GN=NEXN PE=1 SV=1 - [NEXN_HUMAN]	26,37	1	18	18	31	3,406E7	1,944E8	8,25	8,15	6	6	55,85	26,37	18	25	675	80,6	5,33
Q9UM54	Unconventional myosin-VI OS=Homo sapiens OX=9606 GN=MYO6 PE=1 SV=4 - [MYO6_HUMAN]	21,02	1	25	25	33	5,877E6	9,676E7	7,10	8,35	10	10	55,27	18,01	21	23	1294	149,6	8,53
Q13885	Tubulin beta-2A chain OS=Homo sapiens OX=9606 GN=TUBB2A PE=1 SV=1 - [TBB2A_HUMAN]	28,76	4	1	10	30	9,203E7	4,472E8	26,49	16,18	6	12	50,90	28,54	9	18	445	49,9	4,89
Q00610	Clathrin heavy chain 1 OS=Homo sapiens OX=9606 GN=CLTC PE=1 SV=5 - [CLH1_HUMAN]	26,21	2	32	32	41	1,558E7	1,007E8	34,14	16,36	19	20	50,80	15,82	21	21	1675	191,5	5,69
P67936	Tropomyosin alpha-4 chain OS=Homo sapiens OX=9606 GN=TPM4 PE=1 SV=3 - [TPM4_HUMAN]	35,08	1	5	11	28	5,370E7	1,895E9	12,28	17,34	4	5	50,49	27,42	9	23	248	28,5	4,69
Q9Y4I1	Unconventional myosin-Va OS=Homo sapiens OX=9606 GN=MYO5A PE=1 SV=2 - [MYO5A_HUMAN]	16,28	3	27	27	28	8,200E5	3,437E7	0,00	0,54	1	1	50,45	15,74	26	27	1855	215,3	8,48
Q96T37	RNA-binding protein 15 OS=Homo sapiens OX=9606 GN=RBM15 PE=1 SV=2 - [RBM15_HUMAN]	19,24	1	16	16	21	0,000E0	6,128E7		0,00			49,11	19,24	16	21	977	107,1	10,08
Q9BQS8	FYVE and coiled-coil domain-containing protein 1 OS=Homo sapiens OX=9606 GN=FYCO1 PE=1 SV=3 -	15,76	6	17	18	19	0,000E0	3,563E7		0,00			49,10	15,76	18	19	1478	166,9	4,92
P02751	Fibronectin OS=Homo sapiens OX=9606 GN=FN1 PE=1 SV=4 - [FINC_HUMAN]	12,57	1	22	22	23	1,007E6	7,212E7	0,00	1,51	2	2	48,89	11,90	20	21	2386	262,5	5,71
P02545	Prelamin-A/C OS=Homo sapiens OX=9606 GN=LMNA PE=1 SV=1 - [LMNA_HUMAN]	35,54	1	22	22	31	3,958E7	4,702E7	21,57	18,52	12	13	48,57	28,01	17	18	664	74,1	7,02
Q32P44	Echinoderm microtubule-associated protein-like 3 OS=Homo sapiens OX=9606 GN=EML3 PE=1 SV=1 - [E	19,64	1	14	14	18	0,000E0	1,438E8		0,00			47,63	19,64	14	18	896	95,1	7,12
Q9P0K7	Ankycorbin OS=Homo sapiens OX=9606 GN=RAI14 PE=1 SV=2 - [RAI14_HUMAN]	27,86	1	23	23	28	5,447E6	7,157E7	5,62	3,98	4	4	45,86	25,82	21	24	980	110,0	6,21
P11216	Glycogen phosphorylase, brain form OS=Homo sapiens OX=9606 GN=PYGB PE=1 SV=5 - [PYGB_HUMA	29,06	1	17	19	19	2,637E6	7,367E7	2,39	1,90	1	1	44,57	27,16	18	18	843	96,6	6,86
Q09666	Neuroblast differentiation-associated protein AHNAK OS=Homo sapiens OX=9606 GN=AHNAK PE=1 SV=	20,90	2	32	32	41	1,109E7	3,923E7	10,53	9,41	10	12	44,26	16,59	27	29	5890	628,7	6,15
P63010	AP-2 complex subunit beta OS=Homo sapiens OX=9606 GN=AP2B1 PE=1 SV=1 - [AP2B1_HUMAN]	22,63	1	11	18	21	2,293E6	6,329E7	0,00	1,60	1	1	44,16	22,63	18	20	937	104,5	5,38
P46940	Ras GTPase-activating-like protein IQGAP1 OS=Homo sapiens OX=9606 GN=IQGAP1 PE=1 SV=1 - [IQG	15,39	3	21	21	22	4,647E6	1,460E7	5,76	4,04	4	4	43,36	12,07	18	18	1657	189,1	6,48
Q9UHB6	LIM domain and actin-binding protein 1 OS=Homo sapiens OX=9606 GN=LIMA1 PE=1 SV=1 - [LIMA1_H	27,54	1	15	15	24	1,279E7	2,569E8	3,05	10,14	6	6	43,33	26,09	14	18	759	85,2	6,84
Q9ULT8	E3 ubiquitin-protein ligase HECTD1 OS=Homo sapiens OX=9606 GN=HECTD1 PE=1 SV=3 - [HECD1_H	7,89	1	19	19	19	0,000E0	4,595E7		0,00			42,61	7,89	19	19	2610	289,2	5,35
O75369	Filamin-B OS=Homo sapiens OX=9606 GN=FLNB PE=1 SV=2 - [FLNB_HUMAN]	13,30	1	23	26	29	3,241E6	3,620E7	5,16	2,96	7	7	42,29	11,41	22	22	2602	278,0	5,73
O15027	Protein transport protein Sec16A OS=Homo sapiens OX=9606 GN=SEC16A PE=1 SV=3 - [SC16A_HUMA	11,06	1	17	17	19	0,000E0	5,555E7		0,00			42,21	11,06	17	19	2179	233,4	5,63
P07355	Annexin A2 OS=Homo sapiens OX=9606 GN=ANXA2 PE=1 SV=2 - [ANXA2_HUMAN]	43,95	2	15	15	22	1,780E7	1,084E8	10,80	25,66	8	8	41,72	38,64	13	14	339	38,6	7,75
Q96HC4	PDZ and LIM domain protein 5 OS=Homo sapiens OX=9606 GN=PDLIM5 PE=1 SV=5 - [PDLI5_HUMAN	23,83	1	11	11	16	0,000E0	5,492E8		0,00			40,97	23,83	11	16	596	63,9	8,21
P06737	Glycogen phosphorylase, liver form OS=Homo sapiens OX=9606 GN=PYGL PE=1 SV=4 - [PYGL_HUMA	24,68	1	17	19	19	0,000E0	5,104E7		0,00			40,09	24,68	19	19	847	97,1	7,17
Q9UHD2	Serine/threonine-protein kinase TBK1 OS=Homo sapiens OX=9606 GN=TBK1 PE=1 SV=1 - [TBK1_HUMA	25,79	1	15	15	15	0,000E0	4,400E7		0,00			40,00	25,79	15	15	729	83,6	6,79
Q9UPQ0	LIM and calponin homology domains-containing protein 1 OS=Homo sapiens OX=9606 GN=LIMCH1 PE=	17,45	1	14	14	16	3,741E6	2,630E7	0,00	1,20	1	1	39,36	17,45	14	15	1083	121,8	6,47
P02533	Keratin, type I cytoskeletal 14 OS=Homo sapiens OX=9606 GN=KRT14 PE=1 SV=4 - [K1C14_HUMAN]	41,74	13	6	22	73	8,987E8	6,585E7	117,31	41,74	22	58	39,14	24,79	12	15	472	51,5	5,16
Q96HF1	Secreted frizzled-related protein 2 OS=Homo sapiens OX=9606 GN=SFRP2 PE=1 SV=2 - [SFRP2_HUMAN	21,36	1	5	5	15	0,000E0	1,260E9		0,00			39,07	21,36	5	15	295	33,5	7,42
O14497	AT-rich interactive domain-containing protein 1A OS=Homo sapiens OX=9606 GN=ARID1A PE=1 SV=3 -	11,60	1	16	18	20	5,555E5	2,212E7	0,00	0,83	1	1	38,56	10,77	17	19	2285	241,9	6,70
P20592	Interferon-induced GTP-binding protein Mx2 OS=Homo sapiens OX=9606 GN=MX2 PE=1 SV=1 - [MX2_H	26,85	1	14	16	16	0,000E0	8,465E7		0,00			38,25	26,85	16	16	715	82,0	8,76
P05787	Keratin, type II cytoskeletal 8 OS=Homo sapiens OX=9606 GN=KRT8 PE=1 SV=7 - [K2C8_HUMAN]	45,96	4	18	22	74	5,725E8	1,348E8	90,67	36,65	18	59	37,62	36,44	15	15	483	53,7	5,59
Q5SW79	Centrosomal protein of 170 kDa OS=Homo sapiens OX=9606 GN=CEP170 PE=1 SV=1 - [CE170_HUMAN	13,19	2	15	15	15	0,000E0	3,333E7		0,00			37,59	13,19	15	15	1584	175,2	7,11
P24821	Tenascin OS=Homo sapiens OX=9606 GN=TNC PE=1 SV=3 - [TENA_HUMAN]	10,86	1	17	17	17	1,299E6	3,170E7	1,82	0,45	1	1	37,59	10,40	16	16	2201	240,7	4,89
Q12802	A-kinase anchor protein 13 OS=Homo sapiens OX=9606 GN=AKAP13 PE=1 SV=2 - [AKP13_HUMAN]	9,53	1	17	17	17	0,000E0	2,116E7		0,00			37,34	9,53	17	17	2813	307,4	5,24
Q15678	Tyrosine-protein phosphatase non-receptor type 14 OS=Homo sapiens OX=9606 GN=PTPN14 PE=1 SV=2 -	12,72	2	12	12	16	0,000E0	2,231E7		0,00			36,46	12,72	12	16	1187	135,2	8,31
Q16630	Cleavage and polyadenylation specificity factor subunit 6 OS=Homo sapiens OX=9606 GN=CPSF6 PE=1 SV	23,59	1	9	9	15	4,793E6	7,492E7	5,46	4,54	1	1	35,75	23,59	9	14	551	59,2	7,15
Q8TAQ2	SWI/SNF complex subunit SMARCC2 OS=Homo sapiens OX=9606 GN=SMARCC2 PE=1 SV=1 - [SMR	12,60	1	9	12	14	0,000E0	2,585E7		0,00			35,70	12,60	12	14	1214	132,8	5,69
P13647	Keratin, type II cytoskeletal 5 OS=Homo sapiens OX=9606 GN=KRT5 PE=1 SV=3 - [K2C5_HUMAN]	41,02	2	17	26	69	7,621E8	1,922E8	96,54	41,02	26	53	35,32	21,36	12	16	590	62,3	7,74
Q12923	Tyrosine-protein phosphatase non-receptor type 13 OS=Homo sapiens OX=9606 GN=PTPN13 PE=1 SV=2 -	7,48	1	16	16	16	0,000E0	8,283E6		0,00			35,10	7,48	16	16	2485	276,7	6,42
Q9BQE3	Tubulin alpha-1C chain OS=Homo sapiens OX=9606 GN=TUBA1C PE=1 SV=1 - [TBA1C_HUMAN]	42,76	7	4	14	39	1,786E8	5,039E8	20,31	34,08	11	23	34,77	30,29	10	16	449	49,9	5,10
O95782	AP-2 complex subunit alpha-1 OS=Homo sapiens OX=9606 GN=AP2A1 PE=1 SV=3 - [AP2A1_HUMAN]	18,01	1	12	14	15	0,000E0	4,519E7		0,00			34,04	18,01	14	15	977	107,5	7,03
Q14315	Filamin-C OS=Homo sapiens OX=9606 GN=FLNC PE=1 SV=3 - [FLNC_HUMAN]	8,95	1	14	17	17	0,000E0	3,176E7		0,00			33,88	8,95	17	17	2725	290,8	5,97
Q9Y4F5	Centrosomal protein of 170 kDa protein B OS=Homo sapiens OX=9606 GN=CEP170B PE=1 SV=4 - [C170	12,40	1	13	13	14	0,000E0	3,105E7		0,00			33,28	12,40	13	14	1589	171,6	6,84
P23396	40S ribosomal protein S3 OS=Homo sapiens OX=9606 GN=RPS3 PE=1 SV=2 - [RS3_HUMAN]	52,26	1	11	11	18	6,337E6	2,308E8	6,62	21,40	4	5	32,55	39,92	9	13	243	26,7	9,66
P62701	40S ribosomal protein S4, X isoform OS=Homo sapiens OX=9606 GN=RPS4X PE=1 SV=2 - [RS4X_HUM	30,80	1	6	9	13	3,545E6	1,215E8	2,46	10,65	2	2	32,24	30,04	8	11	263	29,6	10,15
Q5T5Y3	Calmodulin-regulated spectrin-associated protein 1 OS=Homo sapiens OX=9606 GN=CAMSAP1 PE=1 SV=	11,42	1	14	14	16	0,000E0	2,125E7		0,00			31,87	11,42	14	16	1602	177,9	6,73
Q15075	Early endosome antigen 1 OS=Homo sapiens OX=9606 GN=EEA1 PE=1 SV=2 - [EEA1_HUMAN]	10,42	1	12	12	12	0,000E0	1,964E7		0,00			31,43	10,42	12	12	1411	162,4	5,68
P02452	Collagen alpha-1(I) chain OS=Homo sapiens OX=9606 GN=COL1A1 PE=1 SV=5 - [CO1A1_HUMAN]	12,84	1	14	14	18	3,597E6	1,115E8	6,71	6,22	6	6	31,31	10,11	11	12	1464	138,9	5,80
Q9NYL9	Tropomodulin-3 OS=Homo sapiens OX=9606 GN=TMOD3 PE=1 SV=1 - [TMOD3_HUMAN]	32,10	2	9	10	13	0,000E0	1,909E8		0,00			30,68	32,10	10	13	352	39,6	5,19
P04259	Keratin, type II cytoskeletal 6B OS=Homo sapiens OX=9606 GN=KRT6B PE=1 SV=5 - [K2C6B_HUMAN]	29,96	2	2	17	61	1,849E9	1,999E8	77,00	25,35	15	48	30,56	18,97	10	13	564	60,0	8,00
P08779	Keratin, type I cytoskeletal 16 OS=Homo sapiens OX=9606 GN=KRT16 PE=1 SV=4 - [K1C16_HUMAN]	38,05	13	7	18	62	7,591E8	6,585E7	92,81	36,15	17	49	30,15	21,56	10	13	473	51,2	5,05
Q9BYX2	TBC1 domain family member 2A OS=Homo sapiens OX=9606 GN=TBC1D2 PE=1 SV=3 - [TBD2A_HUM	15,52	1	12	12	12	0,000E0	2,687E7		0,00			29,46	15,52	12	12	928	105,3	6,58
P0CG48	Polyubiquitin-C OS=Homo sapiens OX=9606 GN=UBC PE=1 SV=3 - [UBC_HUMAN]	64,38	4	5	5	21	1,397E8	5,040E8	10,54	64,38	5	8	29,37	56,50	4	13	685	77,0	7,66
P19474	E3 ubiquitin-protein ligase TRIM21 OS=Homo sapiens OX=9606 GN=TRIM21 PE=1 SV=1 - [RO52_HUM	27,16	1	11	11	13	6,917E6	2,230E8	0,00	4,00	2	2	28,54	27,16	11	11	475	54,1	6,38
P17844	Probable ATP-dependent RNA helicase DDX5 OS=Homo sapiens OX=9606 GN=DDX5 PE=1 SV=1 - [DDX	27,69	1	13	17	18	3,819E6	7,281E7	4,62	3,75	2	2	28,25	25,90	16	16	614	69,1	8,92
P31943	Heterogeneous nuclear ribonucleoprotein H OS=Homo sapiens OX=9606 GN=HNRNPH1 PE=1 SV=4 - [HN	23,39	1	2	6	14	6,937E6	7,923E7	9,91	15,37	4	6	28,00	23,39	6	8	449	49,2	6,30
Q96LD4	Tripartite motif-containing protein 47 OS=Homo sapiens OX=9606 GN=TRIM47 PE=1 SV=2 - [TRI47_HU	16,14	1	8	8	13	0,000E0	5,052E7		0,00			27,77	16,14	8	13	638	69,5	6,44
Q92841	Probable ATP-dependent RNA helicase DDX17 OS=Homo sapiens OX=9606 GN=DDX17 PE=1 SV=2 - [DI	23,73	1	12	16	17	5,218E6	7,113E7	2,92	3,29	2	2	27,67	22,09	15	15	729	80,2	8,27
P11940	Polyadenylate-binding protein 1 OS=Homo sapiens OX=9606 GN=PABPC1 PE=1 SV=2 - [PABP1_HUMA	26,10	4	9	14	17	8,749E6	4,676E7	4,15	8,02	4	4	27,60	21,07	12	13	636	70,6	9,50
P38646	Stress-70 protein, mitochondrial OS=Homo sapiens OX=9606 GN=HSPA9 PE=1 SV=2 - [GRP75_HUMAN	26,66	1	13	13	20	1,225E7	3,477E7	10,95	18,41	9	10	27,29	20,47	10	10	679	73,6	6,16
P62269	40S ribosomal protein S18 OS=Homo sapiens OX=9606 GN=RPS18 PE=1 SV=3 - [RS18_HUMAN]	38,82	1	7	7	13	6,372E6	3,144E8	5,69	19,08	3	3	26,74	38,82	7	10	152	17,7	10,99
P02538	Keratin, type II cytoskeletal 6A OS=Homo sapiens OX=9606 GN=KRT6A PE=1 SV=3 - [K2C6A_HUMAN]	31,74	3	3	19	40	7,526E8	1,869E8	51,55	31,74	19	29	26,72	17,38	9	11	564	60,0	8,00
Q14247	Src substrate cortactin OS=Homo sapiens OX=9606 GN=CTTN PE=1 SV=2 - [SRC8_HUMAN]	20,18	1	10	10	15	1,871E6	3,880E7	0,00	7,82	3	3	26,55	18,00	9	12	550	61,5	5,40
P68366	Tubulin alpha-4A chain OS=Homo sapiens OX=9606 GN=TUBA4A PE=1 SV=1 - [TBA4A_HUMAN]	35,04	3	2	12	31	7,664E7	3,973E8	12,08	22,10	7	17	26,39	25,89	9	14	448	49,9	5,06
Q9UEG4	Zinc finger protein 629 OS=Homo sapiens OX=9606 GN=ZNF629 PE=1 SV=2 - [ZN629_HUMAN]	9,55	1	7	7	9	0,000E0	4,098E7		0,00			26,35	9,55	7	9	869	96,6	7,93
P52907	F-actin-capping protein subunit alpha-1 OS=Homo sapiens OX=9606 GN=CAPZA1 PE=1 SV=3 - [CAZA1_	39,51	1	6	7	14	1,133E7	2,392E8	6,05	28,67	5	6	26,24	32,87	6	8	286	32,9	5,69
Q8TF68	Zinc finger protein 384 OS=Homo sapiens OX=9606 GN=ZNF384 PE=1 SV=2 - [ZN384_HUMAN]	12,65	2	5	5	9	0,000E0	1,744E8		0,00			25,83	12,65	5	9	577	63,2	8,95
Q13464	Rho-associated protein kinase 1 OS=Homo sapiens OX=9606 GN=ROCK1 PE=1 SV=1 - [ROCK1_HUMAN	9,68	1	11	12	12	0,000E0	1,322E7		0,00			25,79	9,68	12	12	1354	158,1	5,90
P13667	Protein disulfide-isomerase A4 OS=Homo sapiens OX=9606 GN=PDIA4 PE=1 SV=2 - [PDIA4_HUMAN]	22,17	1	10	10	13	3,056E6	1,757E7	0,00	3,10	1	1	25,53	19,07	9	12	645	72,9	5,07
O95425	Supervillin OS=Homo sapiens OX=9606 GN=SVIL PE=1 SV=2 - [SVIL_HUMAN]	7,54	1	12	12	12	1,060E6	1,929E7	0,00	0,77	1	1	25,32	6,78	11	11	2214	247,6	6,98
Q07065	Cytoskeleton-associated protein 4 OS=Homo sapiens OX=9606 GN=CKAP4 PE=1 SV=2 - [CKAP4_HUMA	20,93	1	11	11	12	5,539E5	4,313E7	2,01	2,33	2	2	25,26	20,60	10	10	602	66,0	5,92
Q6ZSR9	Uncharacterized protein FLJ45252 OS=Homo sapiens OX=9606 PE=2 SV=2 - [YJ005_HUMAN]	36,62	1	8	8	10	0,000E0	3,813E7		0,00			25,15	36,62	8	10	355	38,0	5,26
Q8IXW5	Putative RNA polymerase II subunit B1 CTD phosphatase RPAP2 OS=Homo sapiens OX=9606 GN=RPAP	19,28	1	10	10	11	0,000E0	1,290E8		0,00			24,60	19,28	10	11	612	69,5	7,78
P08238	Heat shock protein HSP 90-beta OS=Homo sapiens OX=9606 GN=HSP90AB1 PE=1 SV=4 - [HS90B_HUM	33,84	4	9	18	25	2,303E7	4,734E7	29,09	25,00	12	14	24,33	16,71	10	11	724	83,2	5,03
P13646	Keratin, type I cytoskeletal 13 OS=Homo sapiens OX=9606 GN=KRT13 PE=1 SV=4 - [K1C13_HUMAN]	21,18	10	4	11	34	8,226E8	2,293E8	56,79	21,18	11	25	24,05	9,17	6	9	458	49,6	4,96
Q9NS26	Sperm protein associated with the nucleus on the X chromosome A OS=Homo sapiens OX=9606 GN=SPAN	24,74	2	2	3	7	0,000E0	1,356E8		0,00			24,02	24,74	3	7	97	11,0	5,10
Q14152	Eukaryotic translation initiation factor 3 subunit A OS=Homo sapiens OX=9606 GN=EIF3A PE=1 SV=1 - [	12,16	1	14	14	14	2,229E6	6,705E6	8,48	3,33	3	3	23,90	8,83	11	11	1382	166,5	6,79
Q13428	Treacle protein OS=Homo sapiens OX=9606 GN=TCOF1 PE=1 SV=3 - [TCOF_HUMAN]	7,12	1	7	7	8	0,000E0	7,543E7		0,00			23,51	7,12	7	8	1488	152,0	9,04
P0DMV8	Heat shock 70 kDa protein 1A OS=Homo sapiens OX=9606 GN=HSPA1A PE=1 SV=1 - [HS71A_HUMAN	33,07	2	7	13	23	6,072E7	1,546E8	15,80	26,68	10	13	23,41	18,88	9	10	641	70,0	5,66
Q9Y608	Leucine-rich repeat flightless-interacting protein 2 OS=Homo sapiens OX=9606 GN=LRRFIP2 PE=1 SV=1 -	15,53	2	9	9	10	5,116E6	6,478E7	5,04	3,47	2	2	23,35	13,87	8	8	721	82,1	6,95
P12236	ADP/ATP translocase 3 OS=Homo sapiens OX=9606 GN=SLC25A6 PE=1 SV=4 - [ADT3_HUMAN]	20,47	3	1	6	14	6,463E6	2,264E8	1,75	6,38	2	2	23,10	20,47	6	12	298	32,8	9,74
Q10567	AP-1 complex subunit beta-1 OS=Homo sapiens OX=9606 GN=AP1B1 PE=1 SV=2 - [AP1B1_HUMAN]	15,60	1	2	9	11	3,138E6	4,694E7	5,37	9,17	3	3	22,41	8,01	7	8	949	104,6	5,06
Q9Y2X9	Zinc finger protein 281 OS=Homo sapiens OX=9606 GN=ZNF281 PE=1 SV=1 - [ZN281_HUMAN]	19,44	1	9	9	9	0,000E0	4,816E7		0,00			22,28	19,44	9	9	895	96,9	8,48
P07900	Heat shock protein HSP 90-alpha OS=Homo sapiens OX=9606 GN=HSP90AA1 PE=1 SV=5 - [HS90A_HU	31,01	4	10	18	24	1,925E7	4,090E7	19,20	21,99	12	13	22,26	19,54	11	11	732	84,6	5,02
P61978	Heterogeneous nuclear ribonucleoprotein K OS=Homo sapiens OX=9606 GN=HNRNPK PE=1 SV=1 - [HNR	32,18	1	10	10	14	6,615E6	4,854E7	6,09	16,41	5	5	22,15	22,89	8	9	463	50,9	5,54
Q2M2I8	AP2-associated protein kinase 1 OS=Homo sapiens OX=9606 GN=AAK1 PE=1 SV=3 - [AAK1_HUMAN]	12,70	1	8	8	8	0,000E0	3,180E7		0,00			22,14	12,70	8	8	961	103,8	6,60
Q14974	Importin subunit beta-1 OS=Homo sapiens OX=9606 GN=KPNB1 PE=1 SV=2 - [IMB1_HUMAN]	12,10	1	8	8	12	8,396E6	8,913E7	1,94	3,65	2	3	21,80	12,10	8	9	876	97,1	4,78
Q92844	TRAF family member-associated NF-kappa-B activator OS=Homo sapiens OX=9606 GN=TANK PE=1 SV=	15,76	1	7	7	8	0,000E0	6,868E7		0,00			21,67	15,76	7	8	425	47,8	5,68
O15144	Actin-related protein 2/3 complex subunit 2 OS=Homo sapiens OX=9606 GN=ARPC2 PE=1 SV=1 - [ARPC	33,67	1	9	9	14	4,229E6	5,083E7	7,00	13,00	3	3	21,65	27,00	8	11	300	34,3	7,36
Q9ULV4	Coronin-1C OS=Homo sapiens OX=9606 GN=CORO1C PE=1 SV=1 - [COR1C_HUMAN]	24,26	1	9	9	12	1,224E7	1,632E8	3,74	11,18	4	4	21,63	15,19	6	8	474	53,2	7,08
Q9BUF5	Tubulin beta-6 chain OS=Homo sapiens OX=9606 GN=TUBB6 PE=1 SV=1 - [TBB6_HUMAN]	19,73	3	4	7	10	7,720E7	2,374E8	1,73	3,14	1	1	21,63	19,73	7	9	446	49,8	4,88
Q96K83	Zinc finger protein 521 OS=Homo sapiens OX=9606 GN=ZNF521 PE=1 SV=1 - [ZN521_HUMAN]	7,78	1	8	8	9	0,000E0	2,225E7		0,00			21,42	7,78	8	9	1311	147,8	7,01
Q9BXF6	Rab11 family-interacting protein 5 OS=Homo sapiens OX=9606 GN=RAB11FIP5 PE=1 SV=1 - [RFIP5_HU	22,66	1	9	9	11	0,000E0	2,848E7		0,00			21,33	22,66	9	11	653	70,4	9,23
P46060	Ran GTPase-activating protein 1 OS=Homo sapiens OX=9606 GN=RANGAP1 PE=1 SV=1 - [RAGP1_HUM	16,18	1	8	8	9	0,000E0	8,635E7		0,00			21,17	16,18	8	9	587	63,5	4,68
P67809	Nuclease-sensitive element-binding protein 1 OS=Homo sapiens OX=9606 GN=YBX1 PE=1 SV=3 - [YBOX	32,41	2	4	7	9	4,735E6	1,632E8	3,96	9,26	2	2	21,16	26,54	6	7	324	35,9	9,88
O00571	ATP-dependent RNA helicase DDX3X OS=Homo sapiens OX=9606 GN=DDX3X PE=1 SV=3 - [DDX3X_H	22,21	3	12	13	14	4,467E6	6,531E7	5,43	7,40	4	4	21,15	14,80	9	10	662	73,2	7,18
Q5VV52	Zinc finger protein 691 OS=Homo sapiens OX=9606 GN=ZNF691 PE=1 SV=1 - [ZN691_HUMAN]	12,82	1	3	3	7	0,000E0	6,117E7		0,00			21,08	12,82	3	7	312	35,7	7,90
P47756	F-actin-capping protein subunit beta OS=Homo sapiens OX=9606 GN=CAPZB PE=1 SV=4 - [CAPZB_HUM	25,27	1	6	6	14	1,959E7	2,278E8	6,04	13,72	3	4	21,08	25,27	6	10	277	31,3	5,59
Q7Z794	Keratin, type II cytoskeletal 1b OS=Homo sapiens OX=9606 GN=KRT77 PE=2 SV=3 - [K2C1B_HUMAN]	17,65	2	8	11	44	5,076E8	7,102E8	66,72	17,65	11	36	20,60	5,19	3	8	578	61,9	5,99
P14649	Myosin light chain 6B OS=Homo sapiens OX=9606 GN=MYL6B PE=1 SV=1 - [MYL6B_HUMAN]	36,06	1	5	7	11	5,062E8	3,699E9	2,73	6,25	1	1	20,39	36,06	7	10	208	22,7	5,73
P05141	ADP/ATP translocase 2 OS=Homo sapiens OX=9606 GN=SLC25A5 PE=1 SV=7 - [ADT2_HUMAN]	24,16	3	2	7	14	5,932E6	2,264E8	3,65	10,74	3	3	20,24	24,16	7	11	298	32,8	9,69
P46781	40S ribosomal protein S9 OS=Homo sapiens OX=9606 GN=RPS9 PE=1 SV=3 - [RS9_HUMAN]	29,90	1	7	7	11	4,234E6	1,715E8	1,62	8,25	2	2	20,20	29,90	7	9	194	22,6	10,65
Q9H792	Inactive tyrosine-protein kinase PEAK1 OS=Homo sapiens OX=9606 GN=PEAK1 PE=1 SV=4 - [PEAK1_H	6,64	1	10	10	10	0,000E0	2,035E7		0,00			20,19	6,64	10	10	1746	193,0	6,89
Q15746	Myosin light chain kinase, smooth muscle OS=Homo sapiens OX=9606 GN=MYLK PE=1 SV=4 - [MYLK_	6,22	11	10	10	10	0,000E0	1,543E7		0,00			20,11	6,22	10	10	1914	210,6	6,15
P49411	Elongation factor Tu, mitochondrial OS=Homo sapiens OX=9606 GN=TUFM PE=1 SV=2 - [EFTU_HUMA	28,32	1	11	11	11	1,264E6	3,166E7	3,23	8,19	2	2	20,06	20,13	9	9	452	49,5	7,61
Q07021	Complement component 1 Q subcomponent-binding protein, mitochondrial OS=Homo sapiens OX=9606 GN	35,46	1	5	5	9	4,394E7	2,366E7	18,92	30,85	4	4	20,03	35,46	5	5	282	31,3	4,84
P51114	Fragile X mental retardation syndrome-related protein 1 OS=Homo sapiens OX=9606 GN=FXR1 PE=1 SV=	14,65	1	6	7	9	2,107E6	3,024E7	4,02	3,54	2	2	19,90	12,40	6	7	621	69,7	6,15
P0DP24	Calmodulin-2 OS=Homo sapiens OX=9606 GN=CALM2 PE=1 SV=1 - [CALM2_HUMAN]	60,40	1	4	5	14	5,741E7	5,030E8	11,25	38,26	3	5	19,82	35,57	4	9	149	16,8	4,22
Q12906	Interleukin enhancer-binding factor 3 OS=Homo sapiens OX=9606 GN=ILF3 PE=1 SV=3 - [ILF3_HUMAN]	15,32	2	10	10	12	5,345E6	1,241E7	4,61	4,70	3	3	19,46	11,86	8	9	894	95,3	8,76
P55795	Heterogeneous nuclear ribonucleoprotein H2 OS=Homo sapiens OX=9606 GN=HNRNPH2 PE=1 SV=1 - [HI	19,15	1	2	5	10	3,275E6	7,155E7	6,65	11,58	3	4	19,44	13,36	4	6	449	49,2	6,30
Q8N8S7	Protein enabled homolog OS=Homo sapiens OX=9606 GN=ENAH PE=1 SV=2 - [ENAH_HUMAN]	19,29	2	8	8	8	0,000E0	3,243E7		0,00			19,36	19,29	8	8	591	66,5	6,93
P60842	Eukaryotic initiation factor 4A-I OS=Homo sapiens OX=9606 GN=EIF4A1 PE=1 SV=1 - [IF4A1_HUMAN]	29,80	2	10	10	15	1,798E7	2,142E7	11,51	21,18	6	7	19,24	19,46	8	8	406	46,1	5,48
Q04695	Keratin, type I cytoskeletal 17 OS=Homo sapiens OX=9606 GN=KRT17 PE=1 SV=2 - [K1C17_HUMAN]	34,72	11	6	19	39	3,690E8	6,585E7	56,13	34,72	19	32	19,20	12,27	6	7	432	48,1	5,02
Q12933	TNF receptor-associated factor 2 OS=Homo sapiens OX=9606 GN=TRAF2 PE=1 SV=2 - [TRAF2_HUMAN	8,78	1	4	4	7	0,000E0	1,698E8		0,00			19,11	8,78	4	7	501	55,8	7,53
P68104	Elongation factor 1-alpha 1 OS=Homo sapiens OX=9606 GN=EEF1A1 PE=1 SV=1 - [EF1A1_HUMAN]	26,41	2	4	10	21	1,348E8	1,931E8	27,71	20,13	7	13	18,83	12,99	6	8	462	50,1	9,01

O75116	Rho-associated protein kinase 2 OS=Homo sapiens OX=9606 GN=ROCK2 PE=1 SV=4 - [ROCK2_HUMAN	9,22	1	10	11	11	0,000E0	1,603E7		0,00			18,49	9,22	11	11	1388	160,8	6,02
Q12792	Twinfilin-1 OS=Homo sapiens OX=9606 GN=TWF1 PE=1 SV=3 - [TWF1_HUMAN]	18,29	1	6	6	8	2,024E6	2,417E7	0,00	3,71	1	1	18,48	18,29	6	7	350	40,3	6,96
Q96JM3	Chromosome alignment-maintaining phosphoprotein 1 OS=Homo sapiens OX=9606 GN=CHAMP1 PE=1 S	12,19	1	9	9	10	0,000E0	6,326E7		0,00			18,29	12,19	9	10	812	89,0	8,44
P16989	Y-box-binding protein 3 OS=Homo sapiens OX=9606 GN=YBX3 PE=1 SV=4 - [YBOX3_HUMAN]	21,51	2	3	6	8	7,867E6	1,140E8	2,25	2,96	1	1	18,19	21,51	6	7	372	40,1	9,77
O60315	Zinc finger E-box-binding homeobox 2 OS=Homo sapiens OX=9606 GN=ZEB2 PE=1 SV=1 - [ZEB2_HUM	10,71	1	8	9	9	0,000E0	1,662E7		0,00			18,15	10,71	9	9	1214	136,4	6,32
Q9NVI7	ATPase family AAA domain-containing protein 3A OS=Homo sapiens OX=9606 GN=ATAD3A PE=1 SV=2	19,72	3	9	9	9	1,398E6	1,621E7	3,23	2,21	1	1	18,00	17,51	8	8	634	71,3	8,98
P46821	Microtubule-associated protein 1B OS=Homo sapiens OX=9606 GN=MAP1B PE=1 SV=2 - [MAP1B_HUM	5,06	1	8	8	8	0,000E0	7,720E6		0,00			17,91	5,06	8	8	2468	270,5	4,81
Q92922	SWI/SNF complex subunit SMARCC1 OS=Homo sapiens OX=9606 GN=SMARCC1 PE=1 SV=3 - [SMR	8,69	1	6	9	9	0,000E0	2,732E7		0,00			17,89	8,69	9	9	1105	122,8	5,76
Q9NR12	PDZ and LIM domain protein 7 OS=Homo sapiens OX=9606 GN=PDLIM7 PE=1 SV=1 - [PDLI7_HUMAN	20,79	1	6	6	6	0,000E0	2,409E7		0,00			17,88	20,79	6	6	457	49,8	8,41
Q16891	MICOS complex subunit MIC60 OS=Homo sapiens OX=9606 GN=IMMT PE=1 SV=1 - [MIC60_HUMAN	10,82	1	7	7	8	0,000E0	3,923E7		0,00			17,85	10,82	7	8	758	83,6	6,48
Q9P2E9	Ribosome-binding protein 1 OS=Homo sapiens OX=9606 GN=RRBP1 PE=1 SV=5 - [RRBP1_HUMAN]	9,86	1	9	9	9	0,000E0	1,206E7		0,00			17,73	9,86	9	9	1410	152,4	8,60
O14974	Protein phosphatase 1 regulatory subunit 12A OS=Homo sapiens OX=9606 GN=PPP1R12A PE=1 SV=1 - [	11,46	1	11	11	12	3,388E6	4,793E7	1,60	1,94	2	2	17,65	9,51	9	10	1030	115,2	5,40
Q6NZI2	Caveolae-associated protein 1 OS=Homo sapiens OX=9606 GN=CAVIN1 PE=1 SV=1 - [CAVN1_HUMAN]	19,74	1	6	6	6	0,000E0	3,080E7		0,00			17,50	19,74	6	6	390	43,4	5,60
Q8N720	Zinc finger protein 655 OS=Homo sapiens OX=9606 GN=ZNF655 PE=1 SV=3 - [ZN655_HUMAN]	15,27	1	7	7	7	0,000E0	2,935E7		0,00			17,46	15,27	7	7	491	57,4	7,14
P34931	Heat shock 70 kDa protein 1-like OS=Homo sapiens OX=9606 GN=HSPA1L PE=1 SV=2 - [HS71L_HUMA	18,88	2	1	8	14	5,860E7	3,379E8	10,24	14,04	6	8	17,43	13,42	6	6	641	70,3	6,02
P10809	60 kDa heat shock protein, mitochondrial OS=Homo sapiens OX=9606 GN=HSPD1 PE=1 SV=2 - [CH60_H	29,84	1	11	11	18	3,520E7	3,689E6	33,34	28,27	10	13	17,30	13,09	5	5	573	61,0	5,87
O00488	Zinc finger protein 593 OS=Homo sapiens OX=9606 GN=ZNF593 PE=1 SV=2 - [ZN593_HUMAN]	67,91	1	6	6	6	0,000E0	6,800E7		0,00			17,23	67,91	6	6	134	15,2	9,82
Q13310	Polyadenylate-binding protein 4 OS=Homo sapiens OX=9606 GN=PABPC4 PE=1 SV=1 - [PABP4_HUMA	17,08	2	4	9	10	8,562E6	3,398E7	2,54	6,68	3	3	17,18	12,11	7	7	644	70,7	9,26
Q9NS91	E3 ubiquitin-protein ligase RAD18 OS=Homo sapiens OX=9606 GN=RAD18 PE=1 SV=2 - [RAD18_HUM	15,35	1	8	8	9	0,000E0	6,109E7		0,00			17,07	15,35	8	9	495	56,2	7,58
P13639	Elongation factor 2 OS=Homo sapiens OX=9606 GN=EEF2 PE=1 SV=4 - [EF2_HUMAN]	25,64	1	14	14	20	2,932E7	1,032E7	23,21	19,58	10	11	17,03	14,69	9	9	858	95,3	6,83
P47755	F-actin-capping protein subunit alpha-2 OS=Homo sapiens OX=9606 GN=CAPZA2 PE=1 SV=3 - [CAZA2_	28,67	1	4	5	7	1,161E7	7,808E7	2,22	3,50	1	1	16,91	28,67	5	6	286	32,9	5,85
Q12965	Unconventional myosin-Ie OS=Homo sapiens OX=9606 GN=MYO1E PE=1 SV=2 - [MYO1E_HUMAN]	12,36	2	10	10	12	1,944E6	1,067E7	2,54	2,53	3	3	16,91	11,64	9	9	1108	127,0	8,92
O95816	BAG family molecular chaperone regulator 2 OS=Homo sapiens OX=9606 GN=BAG2 PE=1 SV=1 - [BAG2	29,86	1	7	7	7	0,000E0	3,058E7		0,00			16,82	29,86	7	7	211	23,8	6,70
P12956	X-ray repair cross-complementing protein 6 OS=Homo sapiens OX=9606 GN=XRCC6 PE=1 SV=2 - [XRCC	30,05	1	14	14	15	1,599E7	1,145E7	8,03	15,93	7	7	16,72	16,42	8	8	609	69,8	6,64
P27348	14-3-3 protein theta OS=Homo sapiens OX=9606 GN=YWHAQ PE=1 SV=1 - [1433T_HUMAN]	25,71	2	4	6	9	2,235E7	4,076E7	12,31	22,45	4	4	16,66	20,82	5	5	245	27,7	4,78
Q969J2	Zinc finger protein with KRAB and SCAN domains 4 OS=Homo sapiens OX=9606 GN=ZKSCAN4 PE=1 S	15,41	1	6	6	6	0,000E0	6,571E7		0,00			16,55	15,41	6	6	545	61,5	7,61
Q96HS1	Serine/threonine-protein phosphatase PGAM5, mitochondrial OS=Homo sapiens OX=9606 GN=PGAM5 PE	28,37	1	8	8	9	0,000E0	9,778E7		0,00			16,43	28,37	8	9	289	32,0	8,68
P17028	Zinc finger protein 24 OS=Homo sapiens OX=9606 GN=ZNF24 PE=1 SV=4 - [ZNF24_HUMAN]	22,28	1	6	6	7	0,000E0	1,227E8		0,00			16,37	22,28	6	7	368	42,1	6,21
Q13490	Baculoviral IAP repeat-containing protein 2 OS=Homo sapiens OX=9606 GN=BIRC2 PE=1 SV=2 - [BIRC2	15,05	2	7	7	7	0,000E0	4,604E8		0,00			16,28	15,05	7	7	618	69,9	6,70
Q9C037	E3 ubiquitin-protein ligase TRIM4 OS=Homo sapiens OX=9606 GN=TRIM4 PE=1 SV=2 - [TRIM4_HUMA	20,00	1	9	9	9	0,000E0	4,640E7		0,00			16,12	20,00	9	9	500	57,4	8,10
P20591	Interferon-induced GTP-binding protein Mx1 OS=Homo sapiens OX=9606 GN=MX1 PE=1 SV=4 - [MX1_H	15,11	1	5	7	7	0,000E0	1,226E7		0,00			16,07	15,11	7	7	662	75,5	5,83
P17029	Zinc finger protein with KRAB and SCAN domains 1 OS=Homo sapiens OX=9606 GN=ZKSCAN1 PE=1 S	12,79	1	5	5	6	0,000E0	1,015E8		0,00			16,05	12,79	5	6	563	63,6	7,05
P62136	Serine/threonine-protein phosphatase PP1-alpha catalytic subunit OS=Homo sapiens OX=9606 GN=PPP1CA	23,33	1	3	6	8	2,066E7	2,758E7	3,54	5,15	1	1	15,94	18,18	5	7	330	37,5	6,33
Q8NAF0	Zinc finger protein 579 OS=Homo sapiens OX=9606 GN=ZNF579 PE=1 SV=2 - [ZN579_HUMAN]	14,59	1	7	7	8	0,000E0	9,359E7		0,00			15,92	14,59	7	8	562	60,5	8,69
Q86V48	Leucine zipper protein 1 OS=Homo sapiens OX=9606 GN=LUZP1 PE=1 SV=2 - [LUZP1_HUMAN]	9,85	1	7	7	8	0,000E0	1,578E7		0,00			15,91	9,85	7	8	1076	120,2	8,50
P19338	Nucleolin OS=Homo sapiens OX=9606 GN=NCL PE=1 SV=3 - [NUCL_HUMAN]	16,06	1	10	10	14	1,660E7	4,606E7	13,71	11,69	6	8	15,86	7,61	5	6	710	76,6	4,70
Q6ZN55	Zinc finger protein 574 OS=Homo sapiens OX=9606 GN=ZNF574 PE=1 SV=2 - [ZN574_HUMAN]	8,04	1	4	4	5	0,000E0	1,274E7		0,00			15,63	8,04	4	5	896	98,8	8,07
Q15084	Protein disulfide-isomerase A6 OS=Homo sapiens OX=9606 GN=PDIA6 PE=1 SV=1 - [PDIA6_HUMAN]	24,55	1	8	8	9	9,902E6	3,755E7	9,59	11,36	4	4	15,43	16,36	5	5	440	48,1	5,08
P55884	Eukaryotic translation initiation factor 3 subunit B OS=Homo sapiens OX=9606 GN=EIF3B PE=1 SV=3 - [	19,04	1	11	11	12	9,074E6	7,270E6	7,92	9,95	4	4	15,37	10,20	8	8	814	92,4	5,00
Q8TAA9	Vang-like protein 1 OS=Homo sapiens OX=9606 GN=VANGL1 PE=1 SV=1 - [VANG1_HUMAN]	13,74	1	5	5	5	0,000E0	4,918E7		0,00			15,21	13,74	5	5	524	59,9	8,81
P08727	Keratin, type I cytoskeletal 19 OS=Homo sapiens OX=9606 GN=KRT19 PE=1 SV=4 - [K1C19_HUMAN]	30,50	10	2	11	26	2,168E8	6,585E7	45,16	30,50	11	20	15,18	14,75	5	6	400	44,1	5,14
Q5VUA4	Zinc finger protein 318 OS=Homo sapiens OX=9606 GN=ZNF318 PE=1 SV=2 - [ZN318_HUMAN]	4,04	1	8	8	8	0,000E0	5,048E6		0,00			15,16	4,04	8	8	2279	251,0	7,20
Q9Y2F5	Little elongation complex subunit 1 OS=Homo sapiens OX=9606 GN=ICE1 PE=1 SV=5 - [ICE1_HUMAN]	6,80	1	11	11	11	0,000E0	1,169E7		0,00			15,14	6,80	11	11	2266	247,7	5,48
P51991	Heterogeneous nuclear ribonucleoprotein A3 OS=Homo sapiens OX=9606 GN=HNRNPA3 PE=1 SV=2 - [R	21,16	1	5	7	13	1,029E7	5,809E7	5,46	21,16	7	8	15,01	18,52	5	5	378	39,6	9,01
P0CAP2	DNA-directed RNA polymerase II subunit GRINL1A OS=Homo sapiens OX=9606 GN=POLR2M PE=1 SV	25,54	2	6	6	6	0,000E0	3,105E7		0,00			15,01	25,54	6	6	368	41,7	6,39
Q68CZ2	Tensin-3 OS=Homo sapiens OX=9606 GN=TNS3 PE=1 SV=2 - [TENS3_HUMAN]	7,54	1	6	6	6	0,000E0	1,388E7		0,00			14,92	7,54	6	6	1445	155,2	6,81
O75362	Zinc finger protein 217 OS=Homo sapiens OX=9606 GN=ZNF217 PE=1 SV=1 - [ZN217_HUMAN]	5,73	1	4	4	5	0,000E0	6,606E6		0,00			14,85	5,73	4	5	1048	115,2	8,48
P18124	60S ribosomal protein L7 OS=Homo sapiens OX=9606 GN=RPL7 PE=1 SV=1 - [RL7_HUMAN]	26,61	2	6	6	9	5,805E6	6,831E7	6,36	16,13	3	3	14,75	22,18	5	6	248	29,2	10,65
Q9UPN3	Microtubule-actin cross-linking factor 1, isoforms 1/2/3/5 OS=Homo sapiens OX=9606 GN=MACF1 PE=1 S	1,42	1	8	8	8	0,000E0	6,775E6		0,00			14,73	1,42	8	8	7388	837,8	5,39
Q05682	Caldesmon OS=Homo sapiens OX=9606 GN=CALD1 PE=1 SV=3 - [CALD1_HUMAN]	13,37	1	8	8	12	1,100E7	1,987E8	11,06	7,94	5	6	14,59	9,33	5	6	793	93,2	5,66
Q9NYF8	Bcl-2-associated transcription factor 1 OS=Homo sapiens OX=9606 GN=BCLAF1 PE=1 SV=2 - [BCLF1_H	5,98	1	4	4	6	0,000E0	4,264E7		0,00			14,58	5,98	4	6	920	106,1	9,98
Q7Z3K3	Pogo transposable element with ZNF domain OS=Homo sapiens OX=9606 GN=POGZ PE=1 SV=2 - [POGZ	5,74	1	6	6	6	0,000E0	1,216E7		0,00			14,56	5,74	6	6	1410	155,2	7,40
P62241	40S ribosomal protein S8 OS=Homo sapiens OX=9606 GN=RPS8 PE=1 SV=2 - [RS8_HUMAN]	33,65	1	6	6	8	6,954E6	1,227E8	6,19	16,83	3	3	14,44	22,12	4	5	208	24,2	10,32
Q96GM5	SWI/SNF-related matrix-associated actin-dependent regulator of chromatin subfamily D member 1 OS=Homo	16,70	1	3	6	6	1,109E6	2,346E7	0,00	3,30	1	1	14,44	13,40	5	5	515	58,2	9,25
Q08211	ATP-dependent RNA helicase A OS=Homo sapiens OX=9606 GN=DHX9 PE=1 SV=4 - [DHX9_HUMAN]	14,57	1	11	11	13	9,098E6	4,603E6	17,43	10,31	7	7	14,43	8,27	6	6	1270	140,9	6,84
O94966	Ubiquitin carboxyl-terminal hydrolase 19 OS=Homo sapiens OX=9606 GN=USP19 PE=1 SV=2 - [UBP19_I	9,41	1	7	7	7	0,000E0	8,385E6		0,00			14,37	9,41	7	7	1318	145,6	6,28
Q15776	Zinc finger protein with KRAB and SCAN domains 8 OS=Homo sapiens OX=9606 GN=ZKSCAN8 PE=1 S	14,01	2	7	7	7	0,000E0	3,750E7		0,00			14,35	14,01	7	7	578	65,8	7,39
P61247	40S ribosomal protein S3a OS=Homo sapiens OX=9606 GN=RPS3A PE=1 SV=2 - [RS3A_HUMAN]	26,52	1	7	7	10	3,376E6	4,576E7	3,24	14,77	3	3	14,25	19,70	6	7	264	29,9	9,73
Q9BZF9	Uveal autoantigen with coiled-coil domains and ankyrin repeats OS=Homo sapiens OX=9606 GN=UACA PE	4,80	1	6	6	7	0,000E0	7,677E6		0,00			14,22	4,80	6	7	1416	162,4	7,03
A1A4S6	Rho GTPase-activating protein 10 OS=Homo sapiens OX=9606 GN=ARHGAP10 PE=1 SV=1 - [RHG10_H	11,32	1	8	8	8	0,000E0	1,258E7		0,00			14,12	11,32	8	8	786	89,3	7,18
P14618	Pyruvate kinase PKM OS=Homo sapiens OX=9606 GN=PKM PE=1 SV=4 - [KPYM_HUMAN]	40,68	2	17	17	30	4,542E7	4,578E7	25,41	35,78	15	23	14,10	16,20	6	7	531	57,9	7,84
Q9Y2W1	Thyroid hormone receptor-associated protein 3 OS=Homo sapiens OX=9606 GN=THRAP3 PE=1 SV=2 - [TI	8,06	1	7	7	8	0,000E0	1,656E7		0,00			13,96	8,06	7	8	955	108,6	10,15
Q7Z5H3	Rho GTPase-activating protein 22 OS=Homo sapiens OX=9606 GN=ARHGAP22 PE=1 SV=1 - [RHG22_H	10,03	1	6	6	6	0,000E0	1,704E7		0,00			13,85	10,03	6	6	698	76,7	8,15
P51532	Transcription activator BRG1 OS=Homo sapiens OX=9606 GN=SMARCA4 PE=1 SV=2 - [SMCA4_HUMA	9,65	2	6	14	14	1,453E6	1,546E7	5,15	2,00	2	2	13,85	7,65	12	12	1647	184,5	7,88
P36578	60S ribosomal protein L4 OS=Homo sapiens OX=9606 GN=RPL4 PE=1 SV=5 - [RL4_HUMAN]	13,82	1	6	6	7	2,148E6	7,362E7	1,61	1,87	1	1	13,76	13,82	6	6	427	47,7	11,06
Q6DD87	Zinc finger protein 787 OS=Homo sapiens OX=9606 GN=ZNF787 PE=1 SV=4 - [ZN787_HUMAN]	13,09	1	4	4	5	0,000E0	7,527E7		0,00			13,73	13,09	4	5	382	40,4	7,96
P62244	40S ribosomal protein S15a OS=Homo sapiens OX=9606 GN=RPS15A PE=1 SV=2 - [RS15A_HUMAN]	35,38	1	5	5	10	3,529E6	6,027E7	2,74	23,85	3	4	13,70	35,38	5	6	130	14,8	10,13
P61160	Actin-related protein 2 OS=Homo sapiens OX=9606 GN=ACTR2 PE=1 SV=1 - [ARP2_HUMAN]	27,66	1	7	7	9	3,312E6	4,232E7	7,24	13,96	3	3	13,68	16,75	5	6	394	44,7	6,74
P62263	40S ribosomal protein S14 OS=Homo sapiens OX=9606 GN=RPS14 PE=1 SV=3 - [RS14_HUMAN]	36,42	1	4	4	7	1,381E7	3,321E8	5,51	15,89	2	2	13,64	29,14	3	5	151	16,3	10,05
Q96NJ6	Zinc finger protein 3 homolog OS=Homo sapiens OX=9606 GN=ZFP3 PE=1 SV=1 - [ZFP3_HUMAN]	12,95	1	5	5	7	0,000E0	2,271E7		0,00			13,62	12,95	5	7	502	57,6	7,34
P62851	40S ribosomal protein S25 OS=Homo sapiens OX=9606 GN=RPS25 PE=1 SV=1 - [RS25_HUMAN]	22,40	1	3	3	7	5,300E6	9,780E7	3,85	8,00	1	2	13,55	22,40	3	5	125	13,7	10,11
O15143	Actin-related protein 2/3 complex subunit 1B OS=Homo sapiens OX=9606 GN=ARPC1B PE=1 SV=3 - [AI	18,82	1	5	5	8	2,447E6	5,662E7	5,15	12,90	3	3	13,42	14,25	4	5	372	40,9	8,35
Q05639	Elongation factor 1-alpha 2 OS=Homo sapiens OX=9606 GN=EEF1A2 PE=1 SV=1 - [EF1A2_HUMAN]	26,35	1	3	9	12	6,269E7	1,887E8	12,26	20,09	6	6	13,40	10,37	5	6	463	50,4	9,03
Q14444	Caprin-1 OS=Homo sapiens OX=9606 GN=CAPRIN1 PE=1 SV=2 - [CAPR1_HUMAN]	13,68	1	6	6	6	0,000E0	2,973E7		0,00			13,37	13,68	6	6	709	78,3	5,25
Q14980	Nuclear mitotic apparatus protein 1 OS=Homo sapiens OX=9606 GN=NUMA1 PE=1 SV=2 - [NUMA1_HUI	6,90	1	10	10	11	3,998E5	9,296E6	0,00	0,99	1	1	13,35	5,91	9	10	2115	238,1	5,78
Q5T200	Zinc finger CCCH domain-containing protein 13 OS=Homo sapiens OX=9606 GN=ZC3H13 PE=1 SV=1 - [	5,10	1	6	6	6	0,000E0	1,292E7		0,00			13,27	5,10	6	6	1668	196,5	9,42
Q16527	Cysteine and glycine-rich protein 2 OS=Homo sapiens OX=9606 GN=CSRP2 PE=1 SV=3 - [CSRP2_HUM.	26,42	1	3	3	4	0,000E0	1,167E8		0,00			13,22	26,42	3	4	193	20,9	8,62
P26373	60S ribosomal protein L13 OS=Homo sapiens OX=9606 GN=RPL13 PE=1 SV=4 - [RL13_HUMAN]	18,01	1	4	4	7	2,687E6	5,378E7	3,45	9,95	2	2	13,07	18,01	4	5	211	24,2	11,65
P53814	Smoothelin OS=Homo sapiens OX=9606 GN=SMTN PE=1 SV=7 - [SMTN_HUMAN]	8,62	1	7	7	8	1,216E6	2,200E7	0,00	1,53	1	1	13,02	8,62	7	7	917	99,0	9,07
P52943	Cysteine-rich protein 2 OS=Homo sapiens OX=9606 GN=CRIP2 PE=1 SV=1 - [CRIP2_HUMAN]	9,13	1	2	2	4	0,000E0	8,470E8		0,00			12,94	9,13	2	4	208	22,5	8,72
P22626	Heterogeneous nuclear ribonucleoproteins A2/B1 OS=Homo sapiens OX=9606 GN=HNRNPA2B1 PE=1 SV	25,21	1	9	11	16	9,275E7	5,657E7	15,58	17,56	7	9	12,93	16,71	6	7	353	37,4	8,95
O75165	DnaJ homolog subfamily C member 13 OS=Homo sapiens OX=9606 GN=DNAJC13 PE=1 SV=5 - [DJC13	5,31	1	9	9	9	0,000E0	6,768E6		0,00			12,71	5,31	9	9	2243	254,3	6,74
P07237	Protein disulfide-isomerase OS=Homo sapiens OX=9606 GN=P4HB PE=1 SV=3 - [PDIA1_HUMAN]	35,43	1	13	13	17	7,819E6	8,100E6	14,47	25,98	8	11	12,54	12,60	6	6	508	57,1	4,87
P39023	60S ribosomal protein L3 OS=Homo sapiens OX=9606 GN=RPL3 PE=1 SV=2 - [RL3_HUMAN]	23,08	1	7	7	10	1,799E7	6,271E7	7,97	14,89	3	3	12,44	20,10	6	7	403	46,1	10,18
P62249	40S ribosomal protein S16 OS=Homo sapiens OX=9606 GN=RPS16 PE=1 SV=2 - [RS16_HUMAN]	36,30	1	5	5	7	3,310E6	1,527E8	0,00	13,70	2	2	12,43	36,30	5	5	146	16,4	10,21
O43809	Cleavage and polyadenylation specificity factor subunit 5 OS=Homo sapiens OX=9606 GN=NUDT21 PE=1	20,26	1	4	4	6	0,000E0	1,963E8		0,00			12,38	20,26	4	6	227	26,2	8,82
P61981	14-3-3 protein gamma OS=Homo sapiens OX=9606 GN=YWHAG PE=1 SV=2 - [1433G_HUMAN]	28,34	2	4	6	8	1,055E7	2,201E7	13,47	25,10	3	3	12,37	20,65	5	5	247	28,3	4,89
P61158	Actin-related protein 3 OS=Homo sapiens OX=9606 GN=ACTR3 PE=1 SV=3 - [ARP3_HUMAN]	23,21	3	7	7	7	1,088E7	3,633E7	0,00	5,26	1	1	12,22	17,94	6	6	418	47,3	5,88
Q7Z5L9	Interferon regulatory factor 2-binding protein 2 OS=Homo sapiens OX=9606 GN=IRF2BP2 PE=1 SV=2 - [I2	10,90	1	4	4	4	0,000E0	1,044E7		0,00			12,21	10,90	4	4	587	61,0	8,69
Q8N1G0	Zinc finger protein 687 OS=Homo sapiens OX=9606 GN=ZNF687 PE=1 SV=1 - [ZN687_HUMAN]	5,01	1	4	4	4	0,000E0	6,702E6		0,00			12,20	5,01	4	4	1237	129,4	8,19
Q15154	Pericentriolar material 1 protein OS=Homo sapiens OX=9606 GN=PCM1 PE=1 SV=5 - [PCM1_HUMAN]	4,74	1	8	8	8	0,000E0	1,025E7		0,00			12,03	4,74	8	8	2024	228,4	5,02
Q8NCA5	Protein FAM98A OS=Homo sapiens OX=9606 GN=FAM98A PE=1 SV=1 - [FA98A_HUMAN]	14,45	1	4	4	4	0,000E0	1,108E7		0,00			12,03	14,45	4	4	519	55,4	9,03
O95817	BAG family molecular chaperone regulator 3 OS=Homo sapiens OX=9606 GN=BAG3 PE=1 SV=3 - [BAG3	9,74	1	4	4	4	0,000E0	5,666E6		0,00			11,98	9,74	4	4	575	61,6	6,95
P49756	RNA-binding protein 25 OS=Homo sapiens OX=9606 GN=RBM25 PE=1 SV=3 - [RBM25_HUMAN]	13,88	1	7	7	7	3,073E6	1,113E7	5,01	3,32	1	1	11,96	10,56	6	6	843	100,1	6,32
P16403	Histone H1.2 OS=Homo sapiens OX=9606 GN=HIST1H1C PE=1 SV=2 - [H12_HUMAN]	19,25	5	3	4	9	4,314E7	4,406E8	6,21	19,25	4	4	11,82	19,25	4	5	213	21,4	10,93
P19388	DNA-directed RNA polymerases I, II, and III subunit RPABC1 OS=Homo sapiens OX=9606 GN=POLR2E	19,05	1	3	3	4	0,000E0	3,350E7		0,00			11,75	19,05	3	4	210	24,5	5,95
P46013	Proliferation marker protein Ki-67 OS=Homo sapiens OX=9606 GN=MKI67 PE=1 SV=2 - [KI67_HUMAN]	3,53	1	6	6	7	2,361E6	6,895E6	0,00	1,04	2	2	11,75	3,16	5	5	3256	358,5	9,45
P23588	Eukaryotic translation initiation factor 4B OS=Homo sapiens OX=9606 GN=EIF4B PE=1 SV=2 - [IF4B_HU	14,57	1	7	7	8	4,814E6	1,167E7	9,90	4,58	2	2	11,61	9,98	5	6	611	69,1	5,73
Q16670	Zinc finger and SCAN domain-containing protein 26 OS=Homo sapiens OX=9606 GN=ZSCAN26 PE=1 SV	10,25	1	4	4	4	0,000E0	3,590E7		0,00			11,58	10,25	4	4	478	55,2	8,06
P28290	Sperm-specific antigen 2 OS=Homo sapiens OX=9606 GN=SSFA2 PE=1 SV=3 - [SSFA2_HUMAN]	4,77	1	4	4	4	0,000E0	7,608E6		0,00			11,58	4,77	4	4	1259	138,3	5,19
Q99613	Eukaryotic translation initiation factor 3 subunit C OS=Homo sapiens OX=9606 GN=EIF3C PE=1 SV=1 - [	6,90	2	6	6	6	4,472E5	8,518E6	1,76	1,10	1	1	11,53	5,81	5	5	913	105,3	5,68
P46776	60S ribosomal protein L27a OS=Homo sapiens OX=9606 GN=RPL27A PE=1 SV=2 - [RL27A_HUMAN]	22,30	1	3	3	6	5,153E6	2,467E8	1,81	14,19	2	2	11,48	22,30	3	4	148	16,6	11,00
Q86X10	Ral GTPase-activating protein subunit beta OS=Homo sapiens OX=9606 GN=RALGAPB PE=1 SV=1 - [RL	6,02	1	5	5	5	0,000E0	1,016E7		0,00			11,44	6,02	5	5	1494	166,7	6,79
P05161	Ubiquitin-like protein ISG15 OS=Homo sapiens OX=9606 GN=ISG15 PE=1 SV=5 - [ISG15_HUMAN]	25,45	1	3	3	5	0,000E0	3,833E7		0,00			11,39	25,45	3	5	165	17,9	7,44
Q15643	Thyroid receptor-interacting protein 11 OS=Homo sapiens OX=9606 GN=TRIP11 PE=1 SV=3 - [TRIPB_HU	3,39	1	6	6	6	0,000E0	4,820E6		0,00			11,38	3,39	6	6	1979	227,4	5,26
P51531	Probable global transcription activator SNF2L2 OS=Homo sapiens OX=9606 GN=SMARCA2 PE=1 SV=2 -	6,92	2	2	10	10	2,232E6	1,485E7	2,21	0,82	1	1	11,35	6,10	9	9	1590	181,2	7,20
P19387	DNA-directed RNA polymerase II subunit RPB3 OS=Homo sapiens OX=9606 GN=POLR2C PE=1 SV=2 -	26,18	1	5	5	7	0,000E0	8,688E7		0,00			11,26	26,18	5	7	275	31,4	4,92
Q07020	60S ribosomal protein L18 OS=Homo sapiens OX=9606 GN=RPL18 PE=1 SV=2 - [RL18_HUMAN]	25,00	1	4	4	7	3,079E6	6,697E7	5,11	18,09	3	3	11,23	18,09	3	4	188	21,6	11,72
Q13105	Zinc finger and BTB domain-containing protein 17 OS=Homo sapiens OX=9606 GN=ZBTB17 PE=1 SV=3	6,72	1	5	5	5	0,000E0	9,805E6		0,00			11,22	6,72	5	5	803	87,9	6,44
Q969G3	SWI/SNF-related matrix-associated actin-dependent regulator of chromatin subfamily E member 1 OS=Homo	12,17	1	4	4	4	0,000E0	1,565E7		0,00			11,20	12,17	4	4	411	46,6	4,88
P52597	Heterogeneous nuclear ribonucleoprotein F OS=Homo sapiens OX=9606 GN=HNRNPF PE=1 SV=3 - [HNR	16,63	1	2	4	8	5,878E6	4,262E7	3,72	12,77	3	4	11,20	10,36	3	4	415	45,6	5,58
P50402	Emerin OS=Homo sapiens OX=9606 GN=EMD PE=1 SV=1 - [EMD_HUMAN]	25,20	1	5	5	6	0,000E0	8,505E7		0,00			11,14	25,20	5	6	254	29,0	5,50
Q9P258	Protein RCC2 OS=Homo sapiens OX=9606 GN=RCC2 PE=1 SV=2 - [RCC2_HUMAN]	9,96	1	4	4	5	6,754E5	6,942E6	0,00	1,34	1	1	11,14	8,62	3	4	522	56,0	8,78
P36873	Serine/threonine-protein phosphatase PP1-gamma catalytic subunit OS=Homo sapiens OX=9606 GN=PPP1C	18,58	1	2	5	6	2,066E7	3,197E7	3,54	5,26	1	1	11,14	13,31	4	5	323	37,0	6,54
Q8TD47	40S ribosomal protein S4, Y isoform 2 OS=Homo sapiens OX=9606 GN=RPS4Y2 PE=2 SV=3 - [RS4Y2_I	15,21	1	1	4	5	0,000E0	1,039E8		0,00			11,03	15,21	4	5	263	29,3	10,08
Q9Y262	Eukaryotic translation initiation factor 3 subunit L OS=Homo sapiens OX=9606 GN=EIF3L PE=1 SV=1 - [E	11,17	1	6	6	6	0,000E0	5,138E6		0,00			11,01	11,17	6	6	564	66,7	6,34
Q14677	Clathrin interactor 1 OS=Homo sapiens OX=9606 GN=CLINT1 PE=1 SV=1 - [EPN4_HUMAN]	9,92	1	5	5	5	0,000E0	8,504E6		0,00			10,96	9,92	5	5	625	68,2	6,42
Q16666	Gamma-interferon-inducible protein 16 OS=Homo sapiens OX=9606 GN=IFI16 PE=1 SV=3 - [IF16_HUMA	8,41	2	6	6	7	3,703E6	1,627E7	0,00	3,69	2	2	10,95	8,15	5	5	785	88,2	9,28
Q9BXN6	Sperm protein associated with the nucleus on the X chromosome D OS=Homo sapiens OX=9606 GN=SPAN	23,71	2	1	2	3	0,000E0	1,391E7		0,00			10,90	23,71	2	3	97	11,0	6,11
Q00341	Vigilin OS=Homo sapiens OX=9606 GN=HDLBP PE=1 SV=2 - [VIGLN_HUMAN]	7,73	1	7	7	8	9,876E5	5,310E6	2,22	2,21	2	2	10,78	5,52	5	6	1268	141,4	6,87
P62258	14-3-3 protein epsilon OS=Homo sapiens OX=9606 GN=YWHAE PE=1 SV=1 - [1433E_HUMAN]	14,51	2	2	4	5	6,255E7	1,846E7	7,39	11,37	1	1	10,77	14,51	4	4	255	29,2	4,74
P08123	Collagen alpha-2(I) chain OS=Homo sapiens OX=9606 GN=COL1A2 PE=1 SV=7 - [CO1A2_HUMAN]	5,27	1	4	4	4	0,000E0	3,008E6		0,00			10,71	5,27	4	4	1366	129,2	8,95
Q8NFD5	AT-rich interactive domain-containing protein 1B OS=Homo sapiens OX=9606 GN=ARID1B PE=1 SV=2 -	4,47	1	5	7	7	5,555E5	6,841E6	0,00	0,85	1	1	10,71	3,62	6	6	2236	236,0	6,73
Q13347	Eukaryotic translation initiation factor 3 subunit I OS=Homo sapiens OX=9606 GN=EIF3I PE=1 SV=1 - [EI	19,08	1	4	4	4	0,000E0	5,613E6		0,00			10,61	19,08	4	4	325	36,5	5,64
Q9NX58	Cell growth-regulating nucleolar protein OS=Homo sapiens OX=9606 GN=LYAR PE=1 SV=2 - [LYAR_HU	22,16	1	5	5	6	3,379E6	2,099E7	0,00	9,50	2	2	10,61	15,57	4	4	379	43,6	9,54
P37275	Zinc finger E-box-binding homeobox 1 OS=Homo sapiens OX=9606 GN=ZEB1 PE=1 SV=2 - [ZEB1_HUM	5,34	1	4	5	5	0,000E0	9,028E6		0,00			10,59	5,34	5	5	1124	124,0	4,94
Q9Y3I0	tRNA-splicing ligase RtcB homolog OS=Homo sapiens OX=9606 GN=RTCB PE=1 SV=1 - [RTCB_HUMA	10,10	1	4	4	5	8,784E5	7,869E6	0,00	2,38	1	1	10,59	10,10	4	4	505	55,2	7,23
P06748	Nucleophosmin OS=Homo sapiens OX=9606 GN=NPM1 PE=1 SV=2 - [NPM_HUMAN]	31,29	2	8	8	17	5,560E7	5,781E7	19,01	26,87	6	12	10,54	15,31	4	5	294	32,6	4,78
Q674X7	Kazrin OS=Homo sapiens OX=9606 GN=KAZN PE=1 SV=2 - [KAZRN_HUMAN]	8,13	1	5	5	5	0,000E0	6,578E6		0,00			10,44	8,13	5	5	775	86,3	7,03
Q5M775	Cytospin-B OS=Homo sapiens OX=9606 GN=SPECC1 PE=1 SV=1 - [CYTSB_HUMAN]	3,56	1	4	4	4	0,000E0	3,423E6		0,00			10,43	3,56	4	5	1068	118,5	6,70
Q9Y2D5	A-kinase anchor protein 2 OS=Homo sapiens OX=9606 GN=AKAP2 PE=1 SV=3 - [AKAP2_HUMAN]	8,85	1	4	4	4	0,000E0	8,804E6		0,00			10,37	8,85	4	4	859	94,6	5,11
Q8NC51	Plasminogen activator inhibitor 1 RNA-binding protein OS=Homo sapiens OX=9606 GN=SERBP1 PE=1 S	14,71	1	5	5	5	0,000E0	1,595E7		0,00			10,17	14,71	5	5	408	44,9	8,65
P62750	60S ribosomal protein L23a OS=Homo sapiens OX=9606 GN=RPL23A PE=1 SV=1 - [RL23A_HUMAN]	32,05	1	5	5	9	6,255E6	6,307E7	3,85	20,51	3	3	9,96	32,05	5	6	156	17,7	10,45
P62917	60S ribosomal protein L8 OS=Homo sapiens OX=9606 GN=RPL8 PE=1 SV=2 - [RL8_HUMAN]	10,51	1	2	2	6	4,234E6	3,994E7	2,42	10,51	2	3	9,82	10,51	2	3	257	28,0	11,03
P22061	Protein-L-isoaspartate(D-aspartate) O-methyltransferase OS=Homo sapiens OX=9606 GN=PCMT1 PE=1 SV	22,91	1	4	4	4	1,984E6	1,849E7	1,83	3,96	1	1	9,81	18,94	3	3	227	24,6	7,21
Q9Y276	Mitochondrial chaperone BCS1 OS=Homo sapiens OX=9606 GN=BCS1L PE=1 SV=1 - [BCS1_HUMAN]	11,46	1	4	4	4	0,000E0	1,524E7		0,00			9,80	11,46	4	4	419	47,5	8,50
Q9NZR1	Tropomodulin-2 OS=Homo sapiens OX=9606 GN=TMOD2 PE=1 SV=1 - [TMOD2_HUMAN]	11,68	1	4	4	4	0,000E0	1,728E7		0,00			9,77	11,68	4	4	351	39,6	5,27
Q06787	Synaptic functional regulator FMR1 OS=Homo sapiens OX=9606 GN=FMR1 PE=1 SV=1 - [FMR1_HUMA	6,49	1	3	4	5	7,144E5	2,112E7	0,00	1,27	1	1	9,75	6,49	4	4	632	71,1	7,42
O60814	Histone H2B type 1-K OS=Homo sapiens OX=9606 GN=HIST1H2BK PE=1 SV=3 - [H2B1K_HUMAN]	26,98	10	2	4	14	2,227E8	9,154E7	20,68	26,98	4	9	9,70	26,98	4	5	126	13,9	10,32
Q14160	Protein scribble homolog OS=Homo sapiens OX=9606 GN=SCRIB PE=1 SV=4 - [SCRIB_HUMAN]	3,25	2	5	5	5	0,000E0	8,505E6		0,00			9,64	3,25	5	5	1630	174,8	5,07
P06899	Histone H2B type 1-J OS=Homo sapiens OX=9606 GN=HIST1H2BJ PE=1 SV=3 - [H2B1J_HUMAN]	26,19	8	1	3	11	2,119E8	6,848E7	16,01	26,19	3	7	9,57	26,19	3	4	126	13,9	10,32
Q9UJS0	Calcium-binding mitochondrial carrier protein Aralar2 OS=Homo sapiens OX=9606 GN=SLC25A13 PE=1 S	8,30	2	5	5	5	0,000E0	6,216E6		0,00			9,55	8,30	5	5	675	74,1	8,62
Q7Z2W4	Zinc finger CCCH-type antiviral protein 1 OS=Homo sapiens OX=9606 GN=ZC3HAV1 PE=1 SV=3 - [ZCC	6,43	1	4	4	4	0,000E0	5,549E6		0,00			9,53	6,43	4	4	902	101,4	8,40
P09651	Heterogeneous nuclear ribonucleoprotein A1 OS=Homo sapiens OX=9606 GN=HNRNPA1 PE=1 SV=5 - [R	22,85	2	5	7	15	9,040E7	2,359E7	19,98	18,82	6	10	9,47	12,90	4	5	372	38,7	9,13
P63104	14-3-3 protein zeta/delta OS=Homo sapiens OX=9606 GN=YWHAZ PE=1 SV=1 - [1433Z_HUMAN]	33,06	2	4	6	8	3,486E7	2,359E7	7,56	20,82	3	3	9,46	25,31	5	5	245	27,7	4,79
P62424	60S ribosomal protein L7a OS=Homo sapiens OX=9606 GN=RPL7A PE=1 SV=2 - [RL7A_HUMAN]	16,54	1	4	4	7	5,388E6	6,807E7	0,00	12,41	3	3	9,28	13,53	3	4	266	30,0	10,61
P62753	40S ribosomal protein S6 OS=Homo sapiens OX=9606 GN=RPS6 PE=1 SV=1 - [RS6_HUMAN]	16,87	1	4	4	7	5,290E6	1,610E8	1,76	8,03	2	2	9,26	16,87	4	5	249	28,7	10,84
Q9UKN8	General transcription factor 3C polypeptide 4 OS=Homo sapiens OX=9606 GN=GTF3C4 PE=1 SV=2 - [TF3	7,54	1	4	4	4	0,000E0	8,059E6		0,00			9,24	7,54	4	4	822	91,9	6,65
Q9Y230	RuvB-like 2 OS=Homo sapiens OX=9606 GN=RUVBL2 PE=1 SV=3 - [RUVB2_HUMAN]	21,60	1	7	7	8	3,604E6	9,103E6	4,70	9,07	2	2	9,21	15,98	6	6	463	51,1	5,64
O94973	AP-2 complex subunit alpha-2 OS=Homo sapiens OX=9606 GN=AP2A2 PE=1 SV=2 - [AP2A2_HUMAN]	7,24	1	3	5	5	0,000E0	7,532E6		0,00			9,17	7,24	5	5	939	103,9	6,96
P61353	60S ribosomal protein L27 OS=Homo sapiens OX=9606 GN=RPL27 PE=1 SV=2 - [RL27_HUMAN]	25,74	1	4	4	5	5,933E5	5,037E7	0,00	5,88	1	1	9,13	25,74	4	4	136	15,8	10,56
P62140	Serine/threonine-protein phosphatase PP1-beta catalytic subunit OS=Homo sapiens OX=9606 GN=PPP1CB I	15,29	1	1	4	5	2,066E7	2,428E7	3,54	5,20	1	1	9,12	10,09	3	4	327	37,2	6,19
Q9UNF1	Melanoma-associated antigen D2 OS=Homo sapiens OX=9606 GN=MAGED2 PE=1 SV=2 - [MAGD2_HUM	11,72	2	5	6	6	0,000E0	1,684E7		0,00			9,07	11,72	6	6	606	64,9	9,32
P15880	40S ribosomal protein S2 OS=Homo sapiens OX=9606 GN=RPS2 PE=1 SV=2 - [RS2_HUMAN]	17,75	1	5	5	7	3,684E6	2,420E8	0,00	7,51	2	2	9,07	10,24	3	5	293	31,3	10,24
Q02878	60S ribosomal protein L6 OS=Homo sapiens OX=9606 GN=RPL6 PE=1 SV=3 - [RL6_HUMAN]	27,43	1	8	8	9	1,647E7	3,771E7	7,27	14,58	4	4	9,05	16,67	5	5	288	32,7	10,58
P23528	Cofilin-1 OS=Homo sapiens OX=9606 GN=CFL1 PE=1 SV=3 - [COF1_HUMAN]	33,73	2	6	6	13	1,014E8	3,271E7	25,80	33,73	6	10	9,01	22,29	2	3	166	18,5	8,09
Q96HP0	Dedicator of cytokinesis protein 6 OS=Homo sapiens OX=9606 GN=DOCK6 PE=1 SV=3 - [DOCK6_HUM.	2,15	1	1	4	4	0,000E0	1,821E7		0,00			8,99	2,15	4	4	2047	229,4	6,74
Q9BU76	Multiple myeloma tumor-associated protein 2 OS=Homo sapiens OX=9606 GN=MMTAG2 PE=1 SV=1 - [M	15,59	1	3	3	3	0,000E0	8,070E6		0,00			8,92	15,59	3	3	263	29,4	10,02
Q659A1	Little elongation complex subunit 2 OS=Homo sapiens OX=9606 GN=ICE2 PE=1 SV=2 - [ICE2_HUMAN]	7,94	1	7	7	8	0,000E0	9,081E6		0,00			8,86	7,94	7	8	982	109,9	7,09
P02768	Serum albumin OS=Homo sapiens OX=9606 GN=ALB PE=1 SV=2 - [ALBU_HUMAN]	7,88	1	5	5	7	1,583E7	3,659E8	1,89	2,46	2	2	8,82	6,57	4	5	609	69,3	6,28
P62913	60S ribosomal protein L11 OS=Homo sapiens OX=9606 GN=RPL11 PE=1 SV=2 - [RL11_HUMAN]	16,85	1	3	3	5	8,551E6	1,883E8	0,00	7,87	1	1	8,82	16,85	3	4	178	20,2	9,60
P63173	60S ribosomal protein L38 OS=Homo sapiens OX=9606 GN=RPL38 PE=1 SV=2 - [RL38_HUMAN]	28,57	1	2	2	5	5,848E6	7,816E6	1,70	14,29	1	1	8,76	28,57	2	4	70	8,2	10,10
P60866	40S ribosomal protein S20 OS=Homo sapiens OX=9606 GN=RPS20 PE=1 SV=1 - [RS20_HUMAN]	19,33	1	2	2	4	6,717E6	1,497E8	2,13	9,24	1	1	8,76	19,33	2	3	119	13,4	9,94
Q9HBE1	POZ-, AT hook-, and zinc finger-containing protein 1 OS=Homo sapiens OX=9606 GN=PATZ1 PE=1 SV=1	6,11	1	3	3	3	0,000E0	6,592E6		0,00			8,75	6,11	3	3	687	74,0	8,35
Q6NUN9	Zinc finger protein 746 OS=Homo sapiens OX=9606 GN=ZNF746 PE=1 SV=1 - [ZN746_HUMAN]	12,27	1	5	5	6	0,000E0	7,516E6		0,00			8,73	12,27	5	6	644	69,1	6,86
P36957	Dihydrolipoyllysine-residue succinyltransferase component of 2-oxoglutarate dehydrogenase complex, mitoch	7,28	1	3	3	3	0,000E0	8,531E6		0,00			8,65	7,28	3	3	453	48,7	8,95
P59998	Actin-related protein 2/3 complex subunit 4 OS=Homo sapiens OX=9606 GN=ARPC4 PE=1 SV=3 - [ARPC	27,98	1	5	5	6	3,698E6	2,036E7	2,87	11,90	2	2	8,57	22,62	4	4	168	19,7	8,43
P27635	60S ribosomal protein L10 OS=Homo sapiens OX=9606 GN=RPL10 PE=1 SV=4 - [RL10_HUMAN]	17,76	2	4	4	5	0,000E0	8,835E7		0,00			8,56	17,76	4	5	214	24,6	10,08

P40429	60S ribosomal protein L13a OS=Homo sapiens OX=9606 GN=RPL13A PE=1 SV=2 - [RL13A_HUMAN]	17,73	2	4	4	7	2,132E6	9,095E6	0,00	10,84	2	2	8,54	17,73	4	5	203	23,6	10,93
Q9NQW6	Anillin OS=Homo sapiens OX=9606 GN=ANLN PE=1 SV=2 - [ANLN_HUMAN]	5,87	1	5	5	6	0,000E0	8,736E6		0,00			8,54	5,87	5	6	1124	124,1	8,07
Q9UK33	Zinc finger protein 580 OS=Homo sapiens OX=9606 GN=ZNF580 PE=1 SV=1 - [ZN580_HUMAN]	21,51	1	3	3	3	0,000E0	3,430E7		0,00			8,52	21,51	3	3	172	18,7	10,08
P08865	40S ribosomal protein SA OS=Homo sapiens OX=9606 GN=RPSA PE=1 SV=4 - [RSSA_HUMAN]	16,27	1	4	4	5	4,578E6	5,258E6	1,85	3,39	1	1	8,49	16,27	4	4	295	32,8	4,87
P17036	Zinc finger protein 3 OS=Homo sapiens OX=9606 GN=ZNF3 PE=1 SV=3 - [ZNF3_HUMAN]	8,74	1	3	3	4	0,000E0	1,805E7		0,00			8,48	8,74	3	4	446	50,9	7,52
P35637	RNA-binding protein FUS OS=Homo sapiens OX=9606 GN=FUS PE=1 SV=1 - [FUS_HUMAN]	11,22	2	4	4	5	1,930E7	7,619E6	4,66	7,22	2	2	8,43	8,56	3	3	526	53,4	9,36
P30466	HLA class I histocompatibility antigen, B-18 alpha chain OS=Homo sapiens OX=9606 GN=HLA-B PE=1 S	15,75	15	3	4	4	4,075E5	1,891E7	0,00	3,59	1	1	8,42	12,15	3	3	362	40,3	6,76
Q15773	Myeloid leukemia factor 2 OS=Homo sapiens OX=9606 GN=MLF2 PE=1 SV=1 - [MLF2_HUMAN]	11,29	1	2	2	3	0,000E0	2,547E7		0,00			8,34	11,29	2	3	248	28,1	6,90
Q99700	Ataxin-2 OS=Homo sapiens OX=9606 GN=ATXN2 PE=1 SV=2 - [ATX2_HUMAN]	3,05	1	2	3	3	0,000E0	5,990E6		0,00			8,30	3,05	3	3	1313	140,2	9,57
O60506	Heterogeneous nuclear ribonucleoprotein Q OS=Homo sapiens OX=9606 GN=SYNCRIP PE=1 SV=2 - [HNI	21,35	1	7	8	10	1,302E7	4,102E6	5,03	9,47	4	5	8,29	14,77	5	5	623	69,6	8,59
Q9UKL0	REST corepressor 1 OS=Homo sapiens OX=9606 GN=RCOR1 PE=1 SV=2 - [RCOR1_HUMAN]	13,81	1	5	5	5	0,000E0	8,942E6		0,00			8,25	13,81	5	5	485	53,3	7,03
O75083	WD repeat-containing protein 1 OS=Homo sapiens OX=9606 GN=WDR1 PE=1 SV=4 - [WDR1_HUMAN]	13,37	1	6	6	6	2,141E6	1,198E7	0,00	2,81	2	2	8,22	10,56	4	4	606	66,2	6,65
P31946	14-3-3 protein beta/alpha OS=Homo sapiens OX=9606 GN=YWHAB PE=1 SV=3 - [1433B_HUMAN]	27,64	2	4	6	6	4,821E6	1,469E7	8,90	12,60	3	3	8,22	15,04	3	3	246	28,1	4,83
P42677	40S ribosomal protein S27 OS=Homo sapiens OX=9606 GN=RPS27 PE=1 SV=3 - [RS27_HUMAN]	28,57	1	1	2	5	4,679E6	2,552E8	1,63	13,10	1	1	8,22	28,57	2	4	84	9,5	9,45
Q96CW1	AP-2 complex subunit mu OS=Homo sapiens OX=9606 GN=AP2M1 PE=1 SV=2 - [AP2M1_HUMAN]	18,85	1	7	7	7	0,000E0	3,087E7		0,00			8,21	18,85	7	7	435	49,6	9,54
Q00839	Heterogeneous nuclear ribonucleoprotein U OS=Homo sapiens OX=9606 GN=HNRNPU PE=1 SV=6 - [HNR	16,85	1	10	10	10	2,504E7	1,349E7	7,30	12,48	6	6	8,21	4,36	4	4	825	90,5	6,00
P05121	Plasminogen activator inhibitor 1 OS=Homo sapiens OX=9606 GN=SERPINE1 PE=1 SV=1 - [PAI1_HUM/	19,40	1	6	6	6	0,000E0	2,046E7		0,00			8,19	19,40	6	6	402	45,0	7,20
Q9Y3U8	60S ribosomal protein L36 OS=Homo sapiens OX=9606 GN=RPL36 PE=1 SV=3 - [RL36_HUMAN]	20,00	1	3	3	3	0,000E0	1,791E7		0,00			8,15	20,00	3	3	105	12,2	11,59
Q15007	Pre-mRNA-splicing regulator WTAP OS=Homo sapiens OX=9606 GN=WTAP PE=1 SV=2 - [FL2D_HUM/	11,36	1	3	3	3	0,000E0	1,125E7		0,00			8,12	11,36	3	3	396	44,2	5,19
Q71UM5	40S ribosomal protein S27-like OS=Homo sapiens OX=9606 GN=RPS27L PE=1 SV=3 - [RS27L_HUMAN	28,57	1	1	2	3	0,000E0	1,116E8		0,00			8,11	28,57	2	3	84	9,5	9,45
Q13045	Protein flightless-1 homolog OS=Homo sapiens OX=9606 GN=FLII PE=1 SV=2 - [FLII_HUMAN]	5,28	1	6	6	6	0,000E0	1,963E7		0,00			8,07	5,28	6	6	1269	144,7	6,05
O75553	Disabled homolog 1 OS=Homo sapiens OX=9606 GN=DAB1 PE=1 SV=3 - [DAB1_HUMAN]	6,46	1	3	3	3	0,000E0	1,375E7		0,00			8,05	6,46	3	3	588	63,7	5,01
Q13501	Sequestosome-1 OS=Homo sapiens OX=9606 GN=SQSTM1 PE=1 SV=1 - [SQSTM_HUMAN]	18,18	1	5	5	6	0,000E0	1,191E8		0,00			8,04	18,18	5	6	440	47,7	5,22
P21291	Cysteine and glycine-rich protein 1 OS=Homo sapiens OX=9606 GN=CSRP1 PE=1 SV=3 - [CSRP1_HUM/	20,73	1	3	3	3	0,000E0	9,421E7		0,00			8,03	20,73	3	3	193	20,6	8,57
O15514	DNA-directed RNA polymerase II subunit RPB4 OS=Homo sapiens OX=9606 GN=POLR2D PE=1 SV=1 -	40,85	1	4	4	4	0,000E0	4,478E7		0,00			7,86	40,85	4	4	142	16,3	4,79
P83731	60S ribosomal protein L24 OS=Homo sapiens OX=9606 GN=RPL24 PE=1 SV=1 - [RL24_HUMAN]	13,38	1	2	2	3	0,000E0	5,454E7		0,00			7,78	13,38	2	3	157	17,8	11,25
P31689	DnaJ homolog subfamily A member 1 OS=Homo sapiens OX=9606 GN=DNAJA1 PE=1 SV=2 - [DNJA1_H	20,91	3	6	6	7	2,240E6	4,706E7	4,47	10,83	2	2	7,71	10,08	4	5	397	44,8	7,08
P04792	Heat shock protein beta-1 OS=Homo sapiens OX=9606 GN=HSPB1 PE=1 SV=2 - [HSPB1_HUMAN]	35,61	1	6	6	7	4,132E7	4,506E7	8,57	19,51	3	3	7,67	24,39	4	4	205	22,8	6,40
P17040	Zinc finger and SCAN domain-containing protein 20 OS=Homo sapiens OX=9606 GN=ZSCAN20 PE=1 SV	3,16	1	3	3	3	0,000E0	8,766E6		0,00			7,67	3,16	3	3	1043	117,5	6,43
P46782	40S ribosomal protein S5 OS=Homo sapiens OX=9606 GN=RPS5 PE=1 SV=4 - [RS5_HUMAN]	5,39	1	3	3	3	0,000E0	5,524E7		0,00			7,64	5,39	3	3	204	22,9	9,72
P04406	Glyceraldehyde-3-phosphate dehydrogenase OS=Homo sapiens OX=9606 GN=GAPDH PE=1 SV=3 - [G3P_I	69,55	2	15	15	37	2,335E8	7,154E6	62,99	65,37	14	33	7,64	21,19	4	4	335	36,0	8,46
P69905	Hemoglobin subunit alpha OS=Homo sapiens OX=9606 GN=HBA1 PE=1 SV=2 - [HBA_HUMAN]	23,24	1	3	3	4	0,000E0	2,517E7		0,00			7,58	23,24	3	4	142	15,2	8,68
P17098	Zinc finger protein 8 OS=Homo sapiens OX=9606 GN=ZNF8 PE=1 SV=2 - [ZNF8_HUMAN]	6,09	1	3	3	3	0,000E0	7,011E6		0,00			7,54	6,09	3	3	575	64,9	7,39
P18621	60S ribosomal protein L17 OS=Homo sapiens OX=9606 GN=RPL17 PE=1 SV=3 - [RL17_HUMAN]	30,98	1	5	5	6	3,195E6	7,343E7	0,00	7,61	1	1	7,48	30,98	5	5	184	21,4	10,17
P46783	40S ribosomal protein S10 OS=Homo sapiens OX=9606 GN=RPS10 PE=1 SV=1 - [RS10_HUMAN]	28,48	2	4	4	6	0,000E0	2,056E7		0,00			7,46	28,48	4	6	165	18,9	10,15
O43829	Zinc finger and BTB domain-containing protein 14 OS=Homo sapiens OX=9606 GN=ZBTB14 PE=1 SV=2	9,35	1	3	3	3	0,000E0	5,881E6		0,00			7,38	9,35	3	3	449	50,9	6,04
P27816	Microtubule-associated protein 4 OS=Homo sapiens OX=9606 GN=MAP4 PE=1 SV=3 - [MAP4_HUMAN]	6,34	1	5	5	5	1,618E6	1,388E7	4,44	1,82	1	1	7,37	4,51	4	4	1152	120,9	5,43
P13010	X-ray repair cross-complementing protein 5 OS=Homo sapiens OX=9606 GN=XRCC5 PE=1 SV=3 - [XRCC	17,35	1	8	8	13	3,452E7	5,102E6	20,75	14,62	6	10	7,37	6,28	3	3	732	82,7	5,81
O43143	Pre-mRNA-splicing factor ATP-dependent RNA helicase DHX15 OS=Homo sapiens OX=9606 GN=DHX15	8,18	1	5	5	5	6,842E6	6,519E6	3,02	2,77	1	1	7,29	5,41	4	4	795	90,9	7,46
A6NHL2	Tubulin alpha chain-like 3 OS=Homo sapiens OX=9606 GN=TUBAL3 PE=1 SV=2 - [TBAL3_HUMAN]	7,40	1	1	3	5	9,520E6	7,625E7	0,00	1,57	1	1	7,27	7,40	3	4	446	49,9	6,05
P05997	Collagen alpha-2(V) chain OS=Homo sapiens OX=9606 GN=COL5A2 PE=1 SV=3 - [CO5A2_HUMAN]	3,47	1	3	3	3	0,000E0	4,429E6		0,00			7,19	3,47	3	3	1499	144,8	6,46
Q01804	OTU domain-containing protein 4 OS=Homo sapiens OX=9606 GN=OTUD4 PE=1 SV=4 - [OTUD4_HUM/	2,87	1	2	2	2	0,000E0	5,120E6		0,00			7,17	2,87	2	2	1114	124,0	6,71
P68871	Hemoglobin subunit beta OS=Homo sapiens OX=9606 GN=HBB PE=1 SV=2 - [HBB_HUMAN]	21,77	5	3	3	5	1,659E6	5,363E7	3,91	15,65	2	2	7,16	12,93	2	3	147	16,0	7,28
O94979	Protein transport protein Sec31A OS=Homo sapiens OX=9606 GN=SEC31A PE=1 SV=3 - [SC31A_HUMA	2,46	1	3	3	3	0,000E0	7,409E6		0,00			7,14	2,46	3	3	1220	132,9	6,89
P52272	Heterogeneous nuclear ribonucleoprotein M OS=Homo sapiens OX=9606 GN=HNRNPM PE=1 SV=3 - [HN	7,95	1	5	5	7	1,022E7	6,663E6	3,37	4,38	3	4	7,11	4,79	3	3	730	77,5	8,70
Q9Y512	Sorting and assembly machinery component 50 homolog OS=Homo sapiens OX=9606 GN=SAMM50 PE=1	9,81	1	4	4	4	0,000E0	9,725E6		0,00			7,10	9,81	4	4	469	51,9	6,90
P50990	T-complex protein 1 subunit theta OS=Homo sapiens OX=9606 GN=CCT8 PE=1 SV=4 - [TCPQ_HUMAN]	8,94	1	4	4	4	2,562E6	4,417E6	0,00	2,55	1	1	7,06	6,39	3	3	548	59,6	5,60
Q00325	Phosphate carrier protein, mitochondrial OS=Homo sapiens OX=9606 GN=SLC25A3 PE=1 SV=2 - [MPCP_	12,15	1	4	4	4	0,000E0	2,745E7		0,00			7,04	12,15	4	4	362	40,1	9,38
P53621	Coatomer subunit alpha OS=Homo sapiens OX=9606 GN=COPA PE=1 SV=2 - [COPA_HUMAN]	4,49	1	5	5	5	0,000E0	4,015E6		0,00			7,01	4,49	5	5	1224	138,3	7,66
Q6STE5	SWI/SNF-related matrix-associated actin-dependent regulator of chromatin subfamily D member 3 OS=Homo	13,46	1	2	5	5	1,109E6	1,045E7	0,00	3,52	1	1	6,99	9,94	4	4	483	55,0	9,35
Q96LW9	Zinc finger and SCAN domain-containing protein 31 OS=Homo sapiens OX=9606 GN=ZSCAN31 PE=1 SV	6,65	1	2	2	3	0,000E0	4,804E7		0,00			6,97	6,65	2	3	406	47,3	6,89
O00139	Kinesin-like protein KIF2A OS=Homo sapiens OX=9606 GN=KIF2A PE=1 SV=3 - [KIF2A_HUMAN]	5,24	1	4	4	4	0,000E0	1,100E7		0,00			6,97	5,24	4	4	706	79,9	6,68
P0C0S5	Histone H2A.Z OS=Homo sapiens OX=9606 GN=H2AFZ PE=1 SV=2 - [H2AZ_HUMAN]	42,97	15	4	4	8	7,960E7	1,385E8	13,72	42,97	4	5	6,87	20,31	3	3	128	13,5	10,58
P68400	Casein kinase II subunit alpha OS=Homo sapiens OX=9606 GN=CSNK2A1 PE=1 SV=1 - [CSK21_HUMAI	6,65	4	2	3	3	0,000E0	2,778E6		0,00			6,79	6,65	3	3	391	45,1	7,74
P62826	GTP-binding nuclear protein Ran OS=Homo sapiens OX=9606 GN=RAN PE=1 SV=3 - [RAN_HUMAN]	25,00	1	5	5	7	3,693E6	8,689E7	2,45	19,91	4	4	6,77	14,81	3	3	216	24,4	7,49
P16401	Histone H1.5 OS=Homo sapiens OX=9606 GN=HIST1H1B PE=1 SV=3 - [H15_HUMAN]	18,14	1	3	4	6	1,699E7	4,921E7	2,02	9,29	2	3	6,76	12,83	3	3	226	22,6	10,92
Q9UJV9	Probable ATP-dependent RNA helicase DDX41 OS=Homo sapiens OX=9606 GN=DDX41 PE=1 SV=2 - [DI	5,31	1	3	3	3	0,000E0	4,804E6		0,00			6,71	5,31	3	3	622	69,8	6,84
P39019	40S ribosomal protein S19 OS=Homo sapiens OX=9606 GN=RPS19 PE=1 SV=2 - [RS19_HUMAN]	22,07	1	3	3	3	0,000E0	3,223E6		0,00			6,68	22,07	3	3	145	16,1	10,32
P18085	ADP-ribosylation factor 4 OS=Homo sapiens OX=9606 GN=ARF4 PE=1 SV=3 - [ARF4_HUMAN]	37,22	2	4	6	7	1,060E7	2,633E7	0,00	21,11	3	3	6,66	20,00	4	4	180	20,5	7,14
O94964	Protein SOGA1 OS=Homo sapiens OX=9606 GN=SOGA1 PE=1 SV=2 - [SOGA1_HUMAN]	5,20	1	5	5	5	0,000E0	2,336E6		0,00			6,65	5,20	5	5	1423	159,7	6,46
Q9H706	GRB2-associated and regulator of MAPK protein 1 OS=Homo sapiens OX=9606 GN=GAREM1 PE=1 SV=2	6,39	1	5	5	5	0,000E0	5,160E6		0,00			6,65	6,39	5	5	876	97,1	6,77
Q14432	cGMP-inhibited 3',5'-cyclic phosphodiesterase A OS=Homo sapiens OX=9606 GN=PDE3A PE=1 SV=3 - [P	5,26	1	4	4	4	0,000E0	4,002E7		0,00			6,54	5,26	4	4	1141	124,9	6,00
Q06830	Peroxiredoxin-1 OS=Homo sapiens OX=9606 GN=PRDX1 PE=1 SV=1 - [PRDX1_HUMAN]	23,62	1	4	5	6	3,492E7	1,424E7	5,12	14,07	3	3	6,51	14,57	3	3	199	22,1	8,13
Q9BQE4	Selenoprotein S OS=Homo sapiens OX=9606 GN=SELENOS PE=1 SV=3 - [SELS_HUMAN]	15,34	1	2	2	2	0,000E0	2,958E7		0,00			6,49	15,34	2	2	189	21,2	9,70
Q69YN4	Protein virilizer homolog OS=Homo sapiens OX=9606 GN=VIRMA PE=1 SV=2 - [VIR_HUMAN]	1,93	1	3	3	3	0,000E0	6,978E6		0,00			6,46	1,93	3	3	1812	201,9	5,01
Q9Y618	Nuclear receptor corepressor 2 OS=Homo sapiens OX=9606 GN=NCOR2 PE=1 SV=2 - [NCOR2_HUMAN]	2,10	1	4	4	4	0,000E0	5,366E6		0,00			6,40	2,10	4	4	2525	274,6	7,59
P61204	ADP-ribosylation factor 3 OS=Homo sapiens OX=9606 GN=ARF3 PE=1 SV=2 - [ARF3_HUMAN]	43,65	3	4	6	6	1,550E7	1,244E7	0,00	21,55	3	3	6,37	22,10	3	3	181	20,6	7,43
Q8N684	Cleavage and polyadenylation specificity factor subunit 7 OS=Homo sapiens OX=9606 GN=CPSF7 PE=1 SV	6,37	1	2	2	2	0,000E0	1,280E7		0,00			6,34	6,37	2	2	471	52,0	8,00
P20700	Lamin-B1 OS=Homo sapiens OX=9606 GN=LMNB1 PE=1 SV=2 - [LMNB1_HUMAN]	8,36	1	4	4	6	2,967E7	2,751E6	4,18	5,97	3	3	6,32	5,97	3	3	586	66,4	5,16
P13682	Zinc finger protein 35 OS=Homo sapiens OX=9606 GN=ZNF35 PE=1 SV=4 - [ZNF35_HUMAN]	8,54	1	3	3	4	0,000E0	1,263E6		0,00			6,27	8,54	3	4	527	59,1	8,09
Q86YZ3	Hornerin OS=Homo sapiens OX=9606 GN=HRNR PE=1 SV=2 - [HORN_HUMAN]	7,93	1	6	6	10	1,265E8	4,912E6	37,80	7,61	5	8	6,24	2,32	2	2	2850	282,2	10,04
P55789	FAD-linked sulfhydryl oxidase ALR OS=Homo sapiens OX=9606 GN=GFER PE=1 SV=2 - [ALR_HUMAN	18,05	1	2	2	2	0,000E0	2,248E7		0,00			6,23	18,05	2	2	205	23,4	7,62
O15397	Importin-8 OS=Homo sapiens OX=9606 GN=IPO8 PE=1 SV=2 - [IPO8_HUMAN]	4,05	1	3	3	3	0,000E0	4,255E6		0,00			6,23	4,05	3	3	1037	119,9	5,16
Q9NYW8	RB-associated KRAB zinc finger protein OS=Homo sapiens OX=9606 GN=RBAK PE=1 SV=1 - [RBAK_H	6,86	1	4	4	4	0,000E0	3,727E6		0,00			6,18	6,86	4	4	714	82,9	7,33
Q6GYQ0	Ral GTPase-activating protein subunit alpha-1 OS=Homo sapiens OX=9606 GN=RALGAPA1 PE=1 SV=1 -	1,52	1	3	3	3	0,000E0	3,822E6		0,00			6,14	1,52	3	3	2036	229,7	6,19
P62277	40S ribosomal protein S13 OS=Homo sapiens OX=9606 GN=RPS13 PE=1 SV=2 - [RS13_HUMAN]	27,15	1	4	4	5	4,502E6	2,088E7	0,00	4,64	1	1	6,12	27,15	4	4	151	17,2	10,54
O75400	Pre-mRNA-processing factor 40 homolog A OS=Homo sapiens OX=9606 GN=PRPF40A PE=1 SV=2 - [PR	3,66	1	3	3	3	0,000E0	1,193E7		0,00			6,10	3,66	3	3	957	108,7	7,56
Q02413	Desmoglein-1 OS=Homo sapiens OX=9606 GN=DSG1 PE=1 SV=2 - [DSG1_HUMAN]	14,59	1	11	11	16	6,483E7	1,440E7	29,53	13,06	10	14	6,04	3,34	2	2	1049	113,7	5,03
P56270	Myc-associated zinc finger protein OS=Homo sapiens OX=9606 GN=MAZ PE=1 SV=1 - [MAZ_HUMAN]	5,24	1	2	2	3	0,000E0	1,747E8		0,00			6,03	5,24	2	3	477	48,6	8,95
P55735	Protein SEC13 homolog OS=Homo sapiens OX=9606 GN=SEC13 PE=1 SV=3 - [SEC13_HUMAN]	6,83	1	2	2	2	0,000E0	5,075E6		0,00			6,00	6,83	2	2	322	35,5	5,48
Q8WWM7	Ataxin-2-like protein OS=Homo sapiens OX=9606 GN=ATXN2L PE=1 SV=2 - [ATX2L_HUMAN]	3,26	1	2	3	3	0,000E0	7,912E6		0,00			5,99	3,26	3	3	1075	113,3	8,59
Q63ZY3	KN motif and ankyrin repeat domain-containing protein 2 OS=Homo sapiens OX=9606 GN=KANK2 PE=1 S	4,70	1	2	2	2	0,000E0	4,478E6		0,00			5,99	4,70	2	2	851	91,1	5,63
P30504	HLA class I histocompatibility antigen, Cw-4 alpha chain OS=Homo sapiens OX=9606 GN=HLA-C PE=1 S	13,93	23	1	4	4	4,075E5	1,885E7	0,00	7,38	2	2	5,97	6,56	2	2	366	41,0	6,47
P20908	Collagen alpha-1(V) chain OS=Homo sapiens OX=9606 GN=COL5A1 PE=1 SV=3 - [CO5A1_HUMAN]	2,29	2	2	4	4	0,000E0	1,147E7		0,00			5,93	2,29	4	4	1838	183,4	5,06
Q13185	Chromobox protein homolog 3 OS=Homo sapiens OX=9606 GN=CBX3 PE=1 SV=4 - [CBX3_HUMAN]	13,11	1	2	2	2	0,000E0	5,183E7		0,00			5,92	13,11	2	2	183	20,8	5,33
P14625	Endoplasmin OS=Homo sapiens OX=9606 GN=HSP90B1 PE=1 SV=1 - [ENPL_HUMAN]	12,58	1	5	6	7	3,720E6	2,512E6	5,10	10,96	5	5	5,89	3,61	2	2	803	92,4	4,84
P35268	60S ribosomal protein L22 OS=Homo sapiens OX=9606 GN=RPL22 PE=1 SV=2 - [RL22_HUMAN]	18,75	1	2	2	2	0,000E0	5,452E7		0,00			5,88	18,75	2	2	128	14,8	9,19
Q02241	Kinesin-like protein KIF23 OS=Homo sapiens OX=9606 GN=KIF23 PE=1 SV=3 - [KIF23_HUMAN]	5,00	1	4	4	4	0,000E0	4,948E6		0,00			5,84	5,00	4	4	960	110,0	8,51
P16070	CD44 antigen OS=Homo sapiens OX=9606 GN=CD44 PE=1 SV=3 - [CD44_HUMAN]	3,91	1	2	2	2	0,000E0	1,116E7		0,00			5,80	3,91	2	2	742	81,5	5,33
P05783	Keratin, type I cytoskeletal 18 OS=Homo sapiens OX=9606 GN=KRT18 PE=1 SV=2 - [K1C18_HUMAN]	26,98	8	8	10	22	2,968E8	5,188E7	36,00	26,98	10	18	5,71	11,16	3	4	430	48,0	5,45
Q14119	Vascular endothelial zinc finger 1 OS=Homo sapiens OX=9606 GN=VEZF1 PE=1 SV=2 - [VEZF1_HUMAN	7,29	1	2	2	2	0,000E0	5,746E7		0,00			5,70	7,29	2	2	521	56,9	9,52
P50914	60S ribosomal protein L14 OS=Homo sapiens OX=9606 GN=RPL14 PE=1 SV=4 - [RL14_HUMAN]	11,16	1	2	2	4	3,383E7	2,313E7	5,56	5,58	1	2	5,69	11,16	2	2	215	23,4	10,93
Q03135	Caveolin-1 OS=Homo sapiens OX=9606 GN=CAV1 PE=1 SV=4 - [CAV1_HUMAN]	19,66	1	2	2	2	0,000E0	2,408E7		0,00			5,65	19,66	2	2	178	20,5	6,02
Q13813	Spectrin alpha chain, non-erythrocytic 1 OS=Homo sapiens OX=9606 GN=SPTAN1 PE=1 SV=3 - [SPTN1_	4,65	1	7	7	7	3,668E6	4,520E6	5,02	3,07	4	4	5,64	1,58	3	3	2472	284,4	5,35
Q96SL8	Flt3-interacting zinc finger protein 1 OS=Homo sapiens OX=9606 GN=FIZ1 PE=1 SV=2 - [FIZ1_HUMAN]	4,03	1	2	2	2	0,000E0	1,609E7		0,00			5,63	4,03	2	2	496	52,0	8,19
Q5VUJ6	Leucine-rich repeat and calponin homology domain-containing protein 2 OS=Homo sapiens OX=9606 GN=L	7,84	1	5	5	5	0,000E0	3,028E6		0,00			5,63	7,84	5	5	765	84,5	6,55
Q99661	Kinesin-like protein KIF2C OS=Homo sapiens OX=9606 GN=KIF2C PE=1 SV=2 - [KIF2C_HUMAN]	6,21	2	3	3	3	0,000E0	5,775E6		0,00			5,59	6,21	3	3	725	81,3	7,83
Q92928	Putative Ras-related protein Rab-1C OS=Homo sapiens OX=9606 GN=RAB1C PE=5 SV=2 - [RAB1C_HU	22,89	22	2	4	5	5,713E6	4,565E6	1,68	9,45	2	2	5,57	17,41	3	3	201	22,0	5,43
P62266	40S ribosomal protein S23 OS=Homo sapiens OX=9606 GN=RPS23 PE=1 SV=3 - [RS23_HUMAN]	15,38	1	2	2	2	0,000E0	8,864E7		0,00			5,55	15,38	2	2	143	15,8	10,49
Q86YT6	E3 ubiquitin-protein ligase MIB1 OS=Homo sapiens OX=9606 GN=MIB1 PE=1 SV=1 - [MIB1_HUMAN]	2,58	1	2	2	2	0,000E0	2,776E6		0,00			5,52	2,58	2	2	1006	110,1	6,92
P28300	Protein-lysine 6-oxidase OS=Homo sapiens OX=9606 GN=LOX PE=1 SV=2 - [LYOX_HUMAN]	9,83	1	3	3	3	0,000E0	5,907E6		0,00			5,51	9,83	3	3	417	46,9	8,09
O60832	H/ACA ribonucleoprotein complex subunit DKC1 OS=Homo sapiens OX=9606 GN=DKC1 PE=1 SV=3 - [I	8,17	1	3	3	3	0,000E0	3,532E6		0,00			5,49	8,17	3	3	514	57,6	9,42
P08651	Nuclear factor 1 C-type OS=Homo sapiens OX=9606 GN=NFIC PE=1 SV=2 - [NFIC_HUMAN]	5,71	4	3	3	3	0,000E0	2,198E7		0,00			5,48	5,71	3	3	508	55,6	8,38
O95793	Double-stranded RNA-binding protein Staufen homolog 1 OS=Homo sapiens OX=9606 GN=STAU1 PE=1 S	4,68	1	2	2	2	0,000E0	1,432E6		0,00			5,47	4,68	2	2	577	63,1	9,44
P25705	ATP synthase subunit alpha, mitochondrial OS=Homo sapiens OX=9606 GN=ATP5F1A PE=1 SV=1 - [ATI	15,73	1	7	7	7	3,315E6	3,524E6	7,94	11,57	5	5	5,45	4,16	2	2	553	59,7	9,13
Q15417	Calponin-3 OS=Homo sapiens OX=9606 GN=CNN3 PE=1 SV=1 - [CNN3_HUMAN]	7,60	2	2	2	2	0,000E0	5,531E6		0,00			5,42	7,60	2	2	329	36,4	6,05
Q8TCY9	Up-regulator of cell proliferation OS=Homo sapiens OX=9606 GN=URGCP PE=1 SV=2 - [URGCP_HUMA	4,19	1	3	3	3	0,000E0	5,542E6		0,00			5,42	4,19	3	3	931	104,9	6,42
P09543	2',3'-cyclic-nucleotide 3'-phosphodiesterase OS=Homo sapiens OX=9606 GN=CNP PE=1 SV=2 - [CN37_HU	7,84	1	3	3	3	0,000E0	3,762E6		0,00			5,42	7,84	3	3	421	47,5	9,07
Q12789	General transcription factor 3C polypeptide 1 OS=Homo sapiens OX=9606 GN=GTF3C1 PE=1 SV=4 - [TF3	2,18	1	4	4	4	0,000E0	4,233E6		0,00			5,41	2,18	4	4	2109	238,7	7,30
Q9Y5Q9	General transcription factor 3C polypeptide 3 OS=Homo sapiens OX=9606 GN=GTF3C3 PE=1 SV=1 - [TF3	3,27	1	3	3	3	0,000E0	4,364E6		0,00			5,41	3,27	3	3	886	101,2	5,07
Q6NYC8	Phostensin OS=Homo sapiens OX=9606 GN=PPP1R18 PE=1 SV=1 - [PPR18_HUMAN]	8,32	1	3	3	3	0,000E0	1,283E7		0,00			5,41	8,32	3	3	613	67,9	5,40
O94905	Erlin-2 OS=Homo sapiens OX=9606 GN=ERLIN2 PE=1 SV=1 - [ERLN2_HUMAN]	7,08	2	2	2	2	0,000E0	6,069E6		0,00			5,37	7,08	2	2	339	37,8	5,62
O95613	Pericentrin OS=Homo sapiens OX=9606 GN=PCNT PE=1 SV=4 - [PCNT_HUMAN]	0,90	2	3	3	3	0,000E0	3,511E6		0,00			5,36	0,90	3	3	3336	377,8	5,55
Q13547	Histone deacetylase 1 OS=Homo sapiens OX=9606 GN=HDAC1 PE=1 SV=1 - [HDAC1_HUMAN]	4,98	2	2	2	2	0,000E0	1,285E7		0,00			5,33	4,98	2	2	482	55,1	5,48
P58107	Epiplakin OS=Homo sapiens OX=9606 GN=EPPK1 PE=1 SV=3 - [EPIPL_HUMAN]	13,60	1	14	18	19	4,046E7	6,809E6	15,39	12,85	17	17	5,32	0,92	2	2	5088	555,3	5,62
Q8N5F7	NF-kappa-B-activating protein OS=Homo sapiens OX=9606 GN=NKAP PE=1 SV=1 - [NKAP_HUMAN]	6,99	1	2	2	2	0,000E0	9,371E6		0,00			5,31	6,99	2	2	415	47,1	10,11
O60292	Signal-induced proliferation-associated 1-like protein 3 OS=Homo sapiens OX=9606 GN=SIPA1L3 PE=1 SV	1,57	1	2	2	2	0,000E0	3,707E6		0,00			5,27	1,57	2	2	1781	194,5	8,32
Q86YV5	Inactive tyrosine-protein kinase PRAG1 OS=Homo sapiens OX=9606 GN=PRAG1 PE=1 SV=4 - [PRAG1_I	2,70	1	3	3	4	0,000E0	9,457E6		0,00			5,27	2,70	3	4	1406	149,5	7,20
P27694	Replication protein A 70 kDa DNA-binding subunit OS=Homo sapiens OX=9606 GN=RPA1 PE=1 SV=2 -	7,47	1	4	4	4	3,311E6	3,535E6	0,00	2,27	1	1	5,20	5,19	3	3	616	68,1	7,21
P41182	B-cell lymphoma 6 protein OS=Homo sapiens OX=9606 GN=BCL6 PE=1 SV=1 - [BCL6_HUMAN]	4,96	2	3	3	3	0,000E0	1,195E7		0,00			5,19	4,96	3	3	706	78,8	7,94
Q9NTW7	Zinc finger protein 64 homolog, isoforms 3 and 4 OS=Homo sapiens OX=9606 GN=ZFP64 PE=1 SV=3 - [Z	4,81	2	2	2	2	0,000E0	1,368E7		0,00			5,19	4,81	2	2	645	72,2	8,43
Q9P2F9	Zinc finger protein 319 OS=Homo sapiens OX=9606 GN=ZNF319 PE=1 SV=2 - [ZN319_HUMAN]	3,61	1	2	2	2	0,000E0	1,844E7		0,00			5,18	3,61	2	2	582	65,5	8,37
P55209	Nucleosome assembly protein 1-like 1 OS=Homo sapiens OX=9606 GN=NAP1L1 PE=1 SV=1 - [NP1L1_H	17,65	1	4	4	4	2,592E6	1,368E7	0,00	10,23	2	2	5,16	7,42	2	2	391	45,3	4,46
P11717	Cation-independent mannose-6-phosphate receptor OS=Homo sapiens OX=9606 GN=IGF2R PE=1 SV=3 - [M	1,32	1	2	2	2	0,000E0	5,887E6		0,00			5,14	1,32	2	2	2491	274,2	5,94
P84098	60S ribosomal protein L19 OS=Homo sapiens OX=9606 GN=RPL19 PE=1 SV=1 - [RL19_HUMAN]	13,78	1	4	4	4	1,268E6	7,580E6	1,89	4,59	1	1	5,13	9,18	3	3	196	23,5	11,47
P26599	Polypyrimidine tract-binding protein 1 OS=Homo sapiens OX=9606 GN=PTBP1 PE=1 SV=1 - [PTBP1_HU	23,54	1	8	8	8	1,903E7	5,247E6	10,36	15,44	5	5	5,13	8,10	3	3	531	57,2	9,17
Q8WYQ9	Zinc finger CCHC domain-containing protein 14 OS=Homo sapiens OX=9606 GN=ZCCHC14 PE=2 SV=1	2,11	1	1	1	2	0,000E0	5,477E6		0,00			5,12	2,11	1	2	949	100,0	8,18
O43422	52 kDa repressor of the inhibitor of the protein kinase OS=Homo sapiens OX=9606 GN=THAP12 PE=1 SV=	3,02	1	2	2	2	0,000E0	5,523E6		0,00			5,11	3,02	2	2	761	87,6	5,86
P42167	Lamina-associated polypeptide 2, isoforms beta/gamma OS=Homo sapiens OX=9606 GN=TMPO PE=1 SV=	11,01	2	3	3	3	1,690E6	6,299E6	2,31	6,17	1	1	5,09	4,85	2	2	454	50,6	9,38
P06576	ATP synthase subunit beta, mitochondrial OS=Homo sapiens OX=9606 GN=ATP5F1B PE=1 SV=3 - [ATP	23,44	1	9	9	10	1,944E7	3,468E6	23,07	23,44	9	9	5,06	3,02	1	1	529	56,5	5,40
Q9UL63	Muskelin OS=Homo sapiens OX=9606 GN=MKLN1 PE=1 SV=2 - [MKLN1_HUMAN]	8,98	1	5	5	5	0,000E0	3,601E6		0,00			5,05	8,98	5	5	735	84,7	6,34
Q12824	SWI/SNF-related matrix-associated actin-dependent regulator of chromatin subfamily B member 1 OS=Homo	11,17	1	3	3	3	0,000E0	1,995E7	0,00	5,45	1	1	5,05	5,71	2	2	385	44,1	6,23
Q92785	Zinc finger protein ubi-d4 OS=Homo sapiens OX=9606 GN=DPF2 PE=1 SV=2 - [REQU_HUMAN]	6,14	1	2	2	2	0,000E0	2,417E7		0,00			5,04	6,14	2	2	391	44,1	6,33
Q9Y5V3	Melanoma-associated antigen D1 OS=Homo sapiens OX=9606 GN=MAGED1 PE=1 SV=3 - [MAGD1_HUN	3,47	1	1	2	2	0,000E0	1,822E7		0,00			5,03	3,47	2	2	778	86,1	5,83
Q8NCA9	Zinc finger protein 784 OS=Homo sapiens OX=9606 GN=ZNF784 PE=1 SV=1 - [ZN784_HUMAN]	5,88	1	1	1	2	0,000E0	6,993E6		0,00			5,03	5,88	1	2	323	34,2	7,80
Q14241	Elongin-A OS=Homo sapiens OX=9606 GN=ELOA PE=1 SV=2 - [ELOA1_HUMAN]	2,88	1	2	2	2	0,000E0	5,140E6		0,00			5,02	2,88	2	2	798	89,9	9,57
P62805	Histone H4 OS=Homo sapiens OX=9606 GN=HIST1H4A PE=1 SV=2 - [H4_HUMAN]	31,07	1	5	5	15	1,154E8	4,393E7	24,37	31,07	5	13	5,01	21,36	2	2	103	11,4	11,36
P62854	40S ribosomal protein S26 OS=Homo sapiens OX=9606 GN=RPS26 PE=1 SV=3 - [RS26_HUMAN]	20,87	2	2	2	4	1,131E7	3,972E8	2,26	13,04	1	1	5,00	20,87	2	3	115	13,0	11,00
A2RRD8	Zinc finger protein 320 OS=Homo sapiens OX=9606 GN=ZNF320 PE=1 SV=1 - [ZN320_HUMAN]	4,32	13	2	2	2	0,000E0	2,697E6		0,00			4,97	4,32	2	2	509	59,3	8,91
O43293	Death-associated protein kinase 3 OS=Homo sapiens OX=9606 GN=DAPK3 PE=1 SV=1 - [DAPK3_HUMA	7,27	1	3	3	3	0,000E0	2,902E6		0,00			4,96	7,27	3	3	454	52,5	6,89
P50454	Serpin H1 OS=Homo sapiens OX=9606 GN=SERPINH1 PE=1 SV=2 - [SERPH_HUMAN]	11,00	1	3	3	3	2,715E6	6,221E6	5,49	6,22	1	1	4,92	4,78	2	2	418	46,4	8,69
Q58A45	PAN2-PAN3 deadenylation complex subunit PAN3 OS=Homo sapiens OX=9606 GN=PAN3 PE=1 SV=3 -	2,25	1	2	2	2	0,000E0	5,257E6		0,00			4,92	2,25	2	2	887	95,6	8,57
Q96SB3	Neurabin-2 OS=Homo sapiens OX=9606 GN=PPP1R9B PE=1 SV=2 - [NEB2_HUMAN]	4,17	2	3	3	3	0,000E0	3,851E6		0,00			4,91	4,17	3	3	815	89,1	4,97
O95365	Zinc finger and BTB domain-containing protein 7A OS=Homo sapiens OX=9606 GN=ZBTB7A PE=1 SV=1	6,85	1	2	2	2	0,000E0	2,553E7		0,00			4,89	6,85	2	2	584	61,4	5,19
P58215	Lysyl oxidase homolog 3 OS=Homo sapiens OX=9606 GN=LOXL3 PE=1 SV=1 - [LOXL3_HUMAN]	4,78	1	3	3	3	0,000E0	6,680E6		0,00			4,87	4,78	3	3	753	83,1	6,86
P62847	40S ribosomal protein S24 OS=Homo sapiens OX=9606 GN=RPS24 PE=1 SV=1 - [RS24_HUMAN]	19,55	1	2	2	3	0,000E0	8,857E7		0,00			4,85	19,55	2	3	133	15,4	10,78
P26640	Valine--tRNA ligase OS=Homo sapiens OX=9606 GN=VARS PE=1 SV=4 - [SYVC_HUMAN]	4,98	1	3	3	3	6,648E6	0,000E0	2,58	2,85	1	1	4,84	2,14	2	2	1264	140,4	7,59
Q15233	Non-POU domain-containing octamer-binding protein OS=Homo sapiens OX=9606 GN=NONO PE=1 SV=4	10,40	1	3	4	4	1,254E7	5,534E6	6,98	6,58	2	2	4,78	3,82	2	2	471	54,2	8,95
P62906	60S ribosomal protein L10a OS=Homo sapiens OX=9606 GN=RPL10A PE=1 SV=2 - [RL10A_HUMAN]	21,66	1	4	4	5	0,000E0	2,920E7		0,00			4,77	21,66	4	5	217	24,8	9,94
Q9UBG3	Cornulin OS=Homo sapiens OX=9606 GN=CRNN PE=1 SV=1 - [CRNN_HUMAN]	6,67	1	1	1	1	0,000E0	9,472E6		0,00			4,77	6,67	1	1	495	53,5	6,10
Q9UPN4	Centrosomal protein of 131 kDa OS=Homo sapiens OX=9606 GN=CEP131 PE=1 SV=3 - [CP131_HUMAN	3,23	1	3	3	3	0,000E0	4,379E6		0,00			4,76	3,23	3	3	1083	122,1	8,69
P43243	Matrin-3 OS=Homo sapiens OX=9606 GN=MATR3 PE=1 SV=2 - [MATR3_HUMAN]	4,13	1	3	3	3	0,000E0	2,989E6		0,00			4,76	4,13	3	3	847	94,6	6,25
Q92598	Heat shock protein 105 kDa OS=Homo sapiens OX=9606 GN=HSPH1 PE=1 SV=1 - [HS105_HUMAN]	2,91	1	2	2	2	0,000E0	2,914E6		0,00			4,72	2,91	2	2	858	96,8	5,39
Q96II8	Leucine-rich repeat and calponin homology domain-containing protein 3 OS=Homo sapiens OX=9606 GN=L	5,79	1	4	4	4	0,000E0	5,075E6		0,00			4,71	5,79	4	4	777	86,0	6,71
P62841	40S ribosomal protein S15 OS=Homo sapiens OX=9606 GN=RPS15 PE=1 SV=2 - [RS15_HUMAN]	28,28	1	2	2	4	1,593E7	1,985E7	10,46	28,28	2	2	4,70	13,10	1	2	145	17,0	10,39
Q9NZN4	EH domain-containing protein 2 OS=Homo sapiens OX=9606 GN=EHD2 PE=1 SV=2 - [EHD2_HUMAN]	6,81	1	2	3	3	0,000E0	3,796E6		0,00			4,70	6,81	3	3	543	61,1	6,46
P60228	Eukaryotic translation initiation factor 3 subunit E OS=Homo sapiens OX=9606 GN=EIF3E PE=1 SV=1 - [I	10,79	1	3	3	3	4,923E6	3,159E6	1,82	4,04	1	1	4,69	6,74	2	2	445	52,2	6,04
Q08380	Galectin-3-binding protein OS=Homo sapiens OX=9606 GN=LGALS3BP PE=1 SV=1 - [LG3BP_HUMAN]	4,27	1	2	2	2	0,000E0	2,113E7		0,00			4,69	4,27	2	2	585	65,3	5,27
Q13425	Beta-2-syntrophin OS=Homo sapiens OX=9606 GN=SNTB2 PE=1 SV=1 - [SNTB2_HUMAN]	6,48	1	3	3	3	0,000E0	3,152E6		0,00			4,68	6,48	3	3	540	57,9	8,82
P63165	Small ubiquitin-related modifier 1 OS=Homo sapiens OX=9606 GN=SUMO1 PE=1 SV=1 - [SUMO1_HUM	20,79	1	2	2	2	0,000E0	5,575E7		0,00			4,67	20,79	2	2	101	11,5	5,52
P53803	DNA-directed RNA polymerases I, II, and III subunit RPABC4 OS=Homo sapiens OX=9606 GN=POLR2K	13,79	1	2	2	2	0,000E0	3,970E7		0,00			4,67	13,79	2	2	58	7,0	9,06
Q8IYB3	Serine/arginine repetitive matrix protein 1 OS=Homo sapiens OX=9606 GN=SRRM1 PE=1 SV=2 - [SRRM	4,42	1	2	2	2	0,000E0	1,777E7		0,00			4,65	4,42	2	2	904	102,3	11,84
P61313	60S ribosomal protein L15 OS=Homo sapiens OX=9606 GN=RPL15 PE=1 SV=2 - [RL15_HUMAN]	13,73	1	3	3	4	2,948E6	3,269E7	0,00	10,29	2	2	4,64	10,29	2	2	204	24,1	11,62
Q15468	SCL-interrupting locus protein OS=Homo sapiens OX=9606 GN=STIL PE=1 SV=2 - [STIL_HUMAN]	2,87	1	2	2	2	0,000E0	3,985E6		0,00			4,64	2,87	2	2	1287	142,9	6,46
P56134	ATP synthase subunit f, mitochondrial OS=Homo sapiens OX=9606 GN=ATP5J2 PE=1 SV=3 - [ATPK_HU	25,53	1	2	2	2	0,000E0	6,557E6					4,61	25,53	2	2	94	10,9	9,67
Q8TDX7	Serine/threonine-protein kinase Nek7 OS=Homo sapiens OX=9606 GN=NEK7 PE=1 SV=1 - [NEK7_HUMA	7,62	2	2	2	2	0,000E0	5,285E6		0,00			4,61	7,62	2	2	302	34,5	8,25
P31944	Caspase-14 OS=Homo sapiens OX=9606 GN=CASP14 PE=1 SV=2 - [CASPE_HUMAN]	19,42	1	6	6	12	1,192E8	4,702E6	26,93	19,42	6	10	4,60	8,68	2	2	242	27,7	5,58
Q13627	Dual specificity tyrosine-phosphorylation-regulated kinase 1A OS=Homo sapiens OX=9606 GN=DYRK1A P	2,88	2	2	2	2	0,000E0	2,835E6		0,00			4,60	2,88	2	2	763	85,5	8,75

Q9Y3Z3	Deoxynucleoside triphosphate triphosphohydrolase SAMHD1 OS=Homo sapiens OX=9606 GN=SAMHD1 P	4,47	1	3	3	3	0,000E0	1,051E6		0,00			4,60	4,47	3	3	626	72,2	7,14
B9A064	Immunoglobulin lambda-like polypeptide 5 OS=Homo sapiens OX=9606 GN=IGLL5 PE=2 SV=2 - [IGLL5_	3,74	2	1	1	3	5,999E7	3,017E8	2,14	3,74	1	1	4,60	3,74	1	2	214	23,0	8,84
P36542	ATP synthase subunit gamma, mitochondrial OS=Homo sapiens OX=9606 GN=ATP5F1C PE=1 SV=1 - [A	7,72	1	2	2	2	0,000E0	3,534E6		0,00			4,58	7,72	2	2	298	33,0	9,22
P17980	26S proteasome regulatory subunit 6A OS=Homo sapiens OX=9606 GN=PSMC3 PE=1 SV=3 - [PRS6A_H	15,72	1	3	3	3	4,840E6	7,634E6	3,93	7,52	1	1	4,58	8,20	2	2	439	49,2	5,24
O75821	Eukaryotic translation initiation factor 3 subunit G OS=Homo sapiens OX=9606 GN=EIF3G PE=1 SV=2 - [	9,38	1	2	2	2	0,000E0	1,076E7		0,00			4,58	9,38	2	2	320	35,6	6,13
Q8TCB0	Interferon-induced protein 44 OS=Homo sapiens OX=9606 GN=IFI44 PE=2 SV=2 - [IFI44_HUMAN]	5,63	1	2	2	2	0,000E0	1,054E7		0,00			4,58	5,63	2	2	444	50,5	6,89
P62937	Peptidyl-prolyl cis-trans isomerase A OS=Homo sapiens OX=9606 GN=PPIA PE=1 SV=2 - [PPIA_HUMAN	32,12	1	5	5	8	5,790E7	3,460E7	4,72	32,12	5	6	4,58	12,12	2	2	165	18,0	7,81
O00622	Protein CYR61 OS=Homo sapiens OX=9606 GN=CYR61 PE=1 SV=1 - [CYR61_HUMAN]	6,56	1	2	2	2	0,000E0	1,758E7		0,00			4,58	6,56	2	2	381	42,0	8,21
Q3KQU3	MAP7 domain-containing protein 1 OS=Homo sapiens OX=9606 GN=MAP7D1 PE=1 SV=1 - [MA7D1_HU	3,57	1	2	2	2	0,000E0	2,929E6		0,00			4,58	3,57	2	2	841	92,8	10,11
P56545	C-terminal-binding protein 2 OS=Homo sapiens OX=9606 GN=CTBP2 PE=1 SV=1 - [CTBP2_HUMAN]	4,27	1	2	2	2	0,000E0	9,878E6		0,00			4,57	4,27	2	2	445	48,9	6,95
O60568	Procollagen-lysine,2-oxoglutarate 5-dioxygenase 3 OS=Homo sapiens OX=9606 GN=PLOD3 PE=1 SV=1 - [	2,44	1	2	2	2	0,000E0	6,145E6		0,00			4,56	2,44	2	2	738	84,7	6,05
P04899	Guanine nucleotide-binding protein G(i) subunit alpha-2 OS=Homo sapiens OX=9606 GN=GNAI2 PE=1 SV	7,32	10	1	2	2	0,000E0	2,596E6		0,00			4,55	7,32	2	2	355	40,4	5,54
Q96S59	Ran-binding protein 9 OS=Homo sapiens OX=9606 GN=RANBP9 PE=1 SV=1 - [RANB9_HUMAN]	3,84	1	2	2	2	0,000E0	5,498E6		0,00			4,54	3,84	2	2	729	77,8	6,79
Q9UH62	Armadillo repeat-containing X-linked protein 3 OS=Homo sapiens OX=9606 GN=ARMCX3 PE=1 SV=1 - [	7,92	1	2	2	2	0,000E0	7,596E6		0,00			4,53	7,92	2	2	379	42,5	8,37
P28288	ATP-binding cassette sub-family D member 3 OS=Homo sapiens OX=9606 GN=ABCD3 PE=1 SV=1 - [AB	9,71	1	5	5	5	1,748E6	5,002E6	2,93	3,79	1	1	4,53	5,92	4	4	659	75,4	9,36
P35244	Replication protein A 14 kDa subunit OS=Homo sapiens OX=9606 GN=RPA3 PE=1 SV=1 - [RFA3_HUM	54,55	1	4	4	4	2,618E6	3,238E6	3,99	19,01	1	1	4,51	35,54	3	3	121	13,6	5,08
Q92522	Histone H1x OS=Homo sapiens OX=9606 GN=H1FX PE=1 SV=1 - [H1X_HUMAN]	5,63	1	1	1	2	0,000E0	8,261E6		0,00			4,51	5,63	1	2	213	22,5	10,76
P63244	Receptor of activated protein C kinase 1 OS=Homo sapiens OX=9606 GN=RACK1 PE=1 SV=3 - [RACK1_	15,14	1	5	5	6	2,534E6	1,008E7	3,59	12,93	4	4	4,49	5,36	2	2	317	35,1	7,69
P62316	Small nuclear ribonucleoprotein Sm D2 OS=Homo sapiens OX=9606 GN=SNRPD2 PE=1 SV=1 - [SMD2_	16,10	1	2	2	2	0,000E0	1,689E6		0,00			4,48	16,10	2	2	118	13,5	9,91
Q9Y4K0	Lysyl oxidase homolog 2 OS=Homo sapiens OX=9606 GN=LOXL2 PE=1 SV=1 - [LOXL2_HUMAN]	2,33	1	2	2	2	0,000E0	4,675E6		0,00			4,46	2,33	2	2	774	86,7	6,38
Q6ZVM7	TOM1-like protein 2 OS=Homo sapiens OX=9606 GN=TOM1L2 PE=1 SV=1 - [TM1L2_HUMAN]	5,92	1	2	2	2	0,000E0	3,004E6		0,00			4,44	5,92	2	2	507	55,5	4,79
P62899	60S ribosomal protein L31 OS=Homo sapiens OX=9606 GN=RPL31 PE=1 SV=1 - [RL31_HUMAN]	24,80	1	3	3	4	8,473E6	4,371E7	4,58	18,40	2	2	4,44	13,60	2	2	125	14,5	10,54
P63167	Dynein light chain 1, cytoplasmic OS=Homo sapiens OX=9606 GN=DYNLL1 PE=1 SV=1 - [DYL1_HUMA	12,36	2	1	1	3	7,445E6	6,982E7	1,76	12,36	1	1	4,42	12,36	1	2	89	10,4	7,40
Q92614	Unconventional myosin-XVIIIa OS=Homo sapiens OX=9606 GN=MYO18A PE=1 SV=3 - [MY18A_HUMA	0,97	1	2	2	2	0,000E0	3,836E6		0,00			4,41	0,97	2	2	2054	233,0	6,30
Q9UBP9	PTB domain-containing engulfment adapter protein 1 OS=Homo sapiens OX=9606 GN=GULP1 PE=1 SV=1	7,89	1	2	2	2	0,000E0	8,056E6		0,00			4,40	7,89	2	2	304	34,5	7,90
Q9Y4P3	Transducin beta-like protein 2 OS=Homo sapiens OX=9606 GN=TBL2 PE=1 SV=1 - [TBL2_HUMAN]	5,37	1	2	2	2	0,000E0	4,737E6		0,00			4,39	5,37	2	2	447	49,8	9,44
Q63HR2	Tensin-2 OS=Homo sapiens OX=9606 GN=TNS2 PE=1 SV=2 - [TNS2_HUMAN]	1,42	1	2	2	2	0,000E0	4,829E6		0,00			4,39	1,42	2	2	1409	152,5	8,35
Q9Y5Q8	General transcription factor 3C polypeptide 5 OS=Homo sapiens OX=9606 GN=GTF3C5 PE=1 SV=2 - [TF3	5,39	1	2	2	2	0,000E0	3,363E6		0,00			4,39	5,39	2	2	519	59,5	6,90
Q9NZN5	Rho guanine nucleotide exchange factor 12 OS=Homo sapiens OX=9606 GN=ARHGEF12 PE=1 SV=1 - [AR	1,94	1	2	2	2	0,000E0	3,233E6		0,00			4,39	1,94	2	2	1544	173,1	5,74
O94763	Unconventional prefoldin RPB5 interactor 1 OS=Homo sapiens OX=9606 GN=URI1 PE=1 SV=3 - [RMP_H	8,04	2	3	4	4	0,000E0	6,866E6		0,00			4,39	8,04	4	4	535	59,8	5,05
Q04837	Single-stranded DNA-binding protein, mitochondrial OS=Homo sapiens OX=9606 GN=SSBP1 PE=1 SV=1	28,38	1	3	3	4	8,245E6	3,937E6	0,00	22,30	2	2	4,38	16,22	2	2	148	17,2	9,60
Q01082	Spectrin beta chain, non-erythrocytic 1 OS=Homo sapiens OX=9606 GN=SPTBN1 PE=1 SV=2 - [SPTB2_H	3,30	1	6	6	6	1,801E6	7,934E5	7,64	2,24	3	3	4,36	1,06	3	3	2364	274,4	5,57
P62487	DNA-directed RNA polymerase II subunit RPB7 OS=Homo sapiens OX=9606 GN=POLR2G PE=1 SV=1 -	16,86	1	3	3	3	0,000E0	1,761E7		0,00			4,32	16,86	3	3	172	19,3	5,54
P09874	Poly [ADP-ribose] polymerase 1 OS=Homo sapiens OX=9606 GN=PARP1 PE=1 SV=4 - [PARP1_HUMAN	5,82	1	4	4	4	1,367E6	6,486E6	3,81	1,78	1	1	4,30	4,04	3	3	1014	113,0	8,88
Q9P035	Very-long-chain (3R)-3-hydroxyacyl-CoA dehydratase 3 OS=Homo sapiens OX=9606 GN=HACD3 PE=1 SV	4,97	1	2	2	2	0,000E0	1,363E6		0,00			4,28	4,97	2	2	362	43,1	8,94
P62081	40S ribosomal protein S7 OS=Homo sapiens OX=9606 GN=RPS7 PE=1 SV=1 - [RS7_HUMAN]	36,08	1	5	5	7	2,832E5	1,514E7	2,33	27,32	3	4	4,28	20,10	3	3	194	22,1	10,10
P04439	HLA class I histocompatibility antigen, A-3 alpha chain OS=Homo sapiens OX=9606 GN=HLA-A PE=1 SV	13,70	20	1	4	4	4,075E5	1,228E7	0,00	7,40	2	2	4,26	6,30	2	2	365	40,8	6,00
O96019	Actin-like protein 6A OS=Homo sapiens OX=9606 GN=ACTL6A PE=1 SV=1 - [ACL6A_HUMAN]	10,72	1	3	3	3	0,000E0	1,871E7		0,00			4,26	10,72	3	3	429	47,4	5,60
Q9H2D6	TRIO and F-actin-binding protein OS=Homo sapiens OX=9606 GN=TRIOBP PE=1 SV=3 - [TARA_HUMA	0,97	1	1	2	2	0,000E0	9,765E6		0,00			4,25	0,97	2	2	2365	261,2	8,48
Q9BUP0	EF-hand domain-containing protein D1 OS=Homo sapiens OX=9606 GN=EFHD1 PE=1 SV=1 - [EFHD1_H	8,79	2	2	2	2	0,000E0	2,095E7		0,00			4,24	8,79	2	2	239	26,9	5,39
Q9Y5M8	Signal recognition particle receptor subunit beta OS=Homo sapiens OX=9606 GN=SRPRB PE=1 SV=3 - [S	20,30	1	3	3	3	0,000E0	9,936E6	0,00	6,27	1	1	4,24	14,02	2	2	271	29,7	9,04
Q07666	KH domain-containing, RNA-binding, signal transduction-associated protein 1 OS=Homo sapiens OX=9606	11,96	3	5	5	5	2,130E7	6,688E6	2,28	6,55	2	2	4,24	5,42	3	3	443	48,2	8,66
P62829	60S ribosomal protein L23 OS=Homo sapiens OX=9606 GN=RPL23 PE=1 SV=1 - [RL23_HUMAN]	11,43	1	2	2	2	0,000E0	1,069E7		0,00			4,21	11,43	2	2	140	14,9	10,51
P39060	Collagen alpha-1(XVIII) chain OS=Homo sapiens OX=9606 GN=COL18A1 PE=1 SV=5 - [COIA1_HUMAN	1,77	1	2	2	2	0,000E0	1,026E6		0,00			4,21	1,77	2	2	1754	178,1	6,01
P46777	60S ribosomal protein L5 OS=Homo sapiens OX=9606 GN=RPL5 PE=1 SV=3 - [RL5_HUMAN]	8,08	1	2	2	2	0,000E0	7,066E6		0,00			4,17	8,08	2	2	297	34,3	9,72
P14373	Zinc finger protein RFP OS=Homo sapiens OX=9606 GN=TRIM27 PE=1 SV=1 - [TRI27_HUMAN]	4,48	1	2	2	2	0,000E0	2,228E6		0,00			4,17	4,48	2	2	513	58,5	6,21
Q8N0Y2	Zinc finger protein 444 OS=Homo sapiens OX=9606 GN=ZNF444 PE=1 SV=1 - [ZN444_HUMAN]	8,26	1	2	2	2	0,000E0	8,782E6		0,00			4,16	8,26	2	2	327	35,2	8,56
Q13162	Peroxiredoxin-4 OS=Homo sapiens OX=9606 GN=PRDX4 PE=1 SV=1 - [PRDX4_HUMAN]	7,38	1	1	2	2	0,000E0	9,878E6		0,00			4,15	7,38	2	2	271	30,5	6,29
P06312	Immunoglobulin kappa variable 4-1 OS=Homo sapiens OX=9606 GN=IGKV4-1 PE=1 SV=1 - [KV401_HU	13,22	1	2	2	3	1,456E7	1,224E8	2,32	7,44	1	1	4,12	13,22	2	2	121	13,4	5,25
P84243	Histone H3.3 OS=Homo sapiens OX=9606 GN=H3F3A PE=1 SV=2 - [H33_HUMAN]	35,29	3	1	3	5	2,718E7	9,181E6	9,65	30,15	2	3	4,12	28,68	2	2	136	15,3	11,27
Q9Y4F1	FERM, ARHGEF and pleckstrin domain-containing protein 1 OS=Homo sapiens OX=9606 GN=FARP1 PE	2,87	1	2	2	2	0,000E0	3,355E6		0,00			4,10	2,87	2	2	1045	118,6	8,15
P17987	T-complex protein 1 subunit alpha OS=Homo sapiens OX=9606 GN=TCP1 PE=1 SV=1 - [TCPA_HUMAN	18,71	1	6	6	7	7,579E6	2,835E6	12,83	16,91	5	5	4,08	3,60	2	2	556	60,3	6,11
P08708	40S ribosomal protein S17 OS=Homo sapiens OX=9606 GN=RPS17 PE=1 SV=2 - [RS17_HUMAN]	24,44	1	3	3	5	8,476E6	2,488E7	0,00	17,04	2	2	4,08	23,70	2	3	135	15,5	9,85
P17020	Zinc finger protein 16 OS=Homo sapiens OX=9606 GN=ZNF16 PE=1 SV=3 - [ZNF16_HUMAN]	3,37	1	2	2	2	0,000E0	2,370E6		0,00			4,05	3,37	2	2	682	76,4	7,97
Q99715	Collagen alpha-1(XII) chain OS=Homo sapiens OX=9606 GN=COL12A1 PE=1 SV=2 - [COCA1_HUMAN]	1,89	1	4	4	4	0,000E0	4,103E6		0,00			4,04	1,89	4	4	3063	332,9	5,53
P05388	60S acidic ribosomal protein P0 OS=Homo sapiens OX=9606 GN=RPLP0 PE=1 SV=1 - [RLA0_HUMAN]	17,67	2	5	5	7	4,102E6	1,857E7	5,30	17,67	5	5	4,00	6,62	2	2	317	34,3	5,97
P49790	Nuclear pore complex protein Nup153 OS=Homo sapiens OX=9606 GN=NUP153 PE=1 SV=2 - [NU153_HU	2,31	1	2	2	2	0,000E0	6,118E6		0,00			4,00	2,31	2	2	1475	153,8	8,73
Q9UMS4	Pre-mRNA-processing factor 19 OS=Homo sapiens OX=9606 GN=PRPF19 PE=1 SV=1 - [PRP19_HUMAN	1,98	1	1	1	2	0,000E0	3,179E6		0,00			3,97	1,98	1	2	504	55,1	6,61
P18754	Regulator of chromosome condensation OS=Homo sapiens OX=9606 GN=RCC1 PE=1 SV=1 - [RCC1_HU	5,46	1	2	2	2	0,000E0	7,281E6		0,00			3,97	5,46	2	2	421	44,9	7,52
A5PL33	Protein KRBA1 OS=Homo sapiens OX=9606 GN=KRBA1 PE=1 SV=3 - [KRBA1_HUMAN]	2,14	1	2	2	2	0,000E0	5,561E6		0,00			3,97	2,14	2	2	1030	107,4	7,83
Q7Z7K6	Centromere protein V OS=Homo sapiens OX=9606 GN=CENPV PE=1 SV=1 - [CENPV_HUMAN]	14,91	1	3	3	3	0,000E0	8,736E6		0,00			3,94	14,91	3	3	275	29,9	9,73
P63208	S-phase kinase-associated protein 1 OS=Homo sapiens OX=9606 GN=SKP1 PE=1 SV=2 - [SKP1_HUMAN	11,66	1	1	1	2	6,283E6	8,929E5	5,25	11,66	1	1	3,91	11,66	1	1	163	18,6	4,54
Q96JM7	Lethal(3)malignant brain tumor-like protein 3 OS=Homo sapiens OX=9606 GN=L3MBTL3 PE=1 SV=2 - [L	3,59	1	2	2	2	0,000E0	3,564E6		0,00			3,90	3,59	2	2	780	88,3	6,46
Q15582	Transforming growth factor-beta-induced protein ig-h3 OS=Homo sapiens OX=9606 GN=TGFBI PE=1 SV=1	5,56	1	3	3	3	0,000E0	4,224E6		0,00			3,89	5,56	3	3	683	74,6	7,71
P38919	Eukaryotic initiation factor 4A-III OS=Homo sapiens OX=9606 GN=EIF4A3 PE=1 SV=4 - [IF4A3_HUMAN	9,98	1	3	3	3	1,013E7	1,721E6	3,29	4,62	1	1	3,89	5,35	2	2	411	46,8	6,73
Q02809	Procollagen-lysine,2-oxoglutarate 5-dioxygenase 1 OS=Homo sapiens OX=9606 GN=PLOD1 PE=1 SV=2 - [	3,44	1	2	2	2	0,000E0	5,275E6		0,00			3,86	3,44	2	2	727	83,5	6,95
P35998	26S proteasome regulatory subunit 7 OS=Homo sapiens OX=9606 GN=PSMC2 PE=1 SV=3 - [PRS7_HUM	14,78	1	6	6	6	2,359E6	3,816E6	0,00	1,85	1	1	3,84	12,93	5	5	433	48,6	5,95
Q07955	Serine/arginine-rich splicing factor 1 OS=Homo sapiens OX=9606 GN=SRSF1 PE=1 SV=2 - [SRSF1_HUM	16,53	1	3	3	3	4,353E7	7,784E6	3,13	9,27	1	1	3,82	7,26	2	2	248	27,7	10,36
O60884	DnaJ homolog subfamily A member 2 OS=Homo sapiens OX=9606 GN=DNAJA2 PE=1 SV=1 - [DNJA2_H	21,12	1	5	5	5	3,726E6	1,058E7	4,97	13,35	2	2	3,81	7,77	3	3	412	45,7	6,48
P84103	Serine/arginine-rich splicing factor 3 OS=Homo sapiens OX=9606 GN=SRSF3 PE=1 SV=1 - [SRSF3_HUM	25,61	1	3	4	5	3,000E7	4,285E7	2,71	20,12	2	2	3,79	14,02	2	3	164	19,3	11,65
Q15365	Poly(rC)-binding protein 1 OS=Homo sapiens OX=9606 GN=PCBP1 PE=1 SV=2 - [PCBP1_HUMAN]	19,38	2	3	4	5	2,515E6	7,098E6	2,80	13,76	3	3	3,78	12,08	2	2	356	37,5	7,09
Q53G44	Interferon-induced protein 44-like OS=Homo sapiens OX=9606 GN=IFI44L PE=2 SV=3 - [IF44L_HUMAN]	5,09	1	2	2	3	0,000E0	5,460E6		0,00			3,77	5,09	2	3	452	51,3	6,79
Q99832	T-complex protein 1 subunit eta OS=Homo sapiens OX=9606 GN=CCT7 PE=1 SV=2 - [TCPH_HUMAN]	9,21	1	3	3	3	6,212E6	2,058E6	4,53	2,95	1	1	3,77	6,26	2	2	543	59,3	7,65
P27482	Calmodulin-like protein 3 OS=Homo sapiens OX=9606 GN=CALML3 PE=1 SV=2 - [CALL3_HUMAN]	40,27	1	4	5	9	5,316E7	8,303E8	11,88	40,27	5	6	3,71	5,37	1	3	149	16,9	4,42
Q12931	Heat shock protein 75 kDa, mitochondrial OS=Homo sapiens OX=9606 GN=TRAP1 PE=1 SV=3 - [TRAP1	3,41	1	1	2	3	9,524E6	3,309E7	2,95	3,41	2	2	3,70	1,99	1	1	704	80,1	8,21
P12107	Collagen alpha-1(XI) chain OS=Homo sapiens OX=9606 GN=COL11A1 PE=1 SV=4 - [COBA1_HUMAN]	1,61	2	1	3	3	0,000E0	3,427E6		0,00			3,70	1,61	3	3	1806	181,0	5,17
Q8IXQ6	Poly [ADP-ribose] polymerase 9 OS=Homo sapiens OX=9606 GN=PARP9 PE=1 SV=2 - [PARP9_HUMAN	1,87	1	1	1	1	0,000E0	3,490E6		0,00			3,69	1,87	1	1	854	96,3	7,91
O15156	Zinc finger and BTB domain-containing protein 7B OS=Homo sapiens OX=9606 GN=ZBTB7B PE=1 SV=2	4,82	1	1	1	2	0,000E0	7,270E7		0,00			3,69	4,82	1	2	539	58,0	5,86
L0R819	ASNSD1 upstream open reading frame protein OS=Homo sapiens OX=9606 GN=ASDURF PE=1 SV=1 - [A	23,96	1	2	2	2	0,000E0	7,151E6		0,00			3,68	23,96	2	2	96	11,2	8,88
P39687	Acidic leucine-rich nuclear phosphoprotein 32 family member A OS=Homo sapiens OX=9606 GN=ANP32A	26,51	2	3	3	5	3,286E7	1,287E7	9,19	26,51	3	4	3,65	6,43	1	1	249	28,6	4,09
P10599	Thioredoxin OS=Homo sapiens OX=9606 GN=TXN PE=1 SV=3 - [THIO_HUMAN]	12,38	1	1	1	2	8,873E7	3,330E7	3,37	12,38	1	1	3,63	12,38	1	1	105	11,7	4,92
P42704	Leucine-rich PPR motif-containing protein, mitochondrial OS=Homo sapiens OX=9606 GN=LRPPRC PE=1	5,09	1	5	5	6	3,918E6	1,007E6	2,97	5,09	4	4	3,61	1,65	2	2	1394	157,8	6,13
Q92925	SWI/SNF-related matrix-associated actin-dependent regulator of chromatin subfamily D member 2 OS=Homo	7,34	1	1	3	3	1,109E6	5,679E6	0,00	3,20	1	1	3,58	4,14	2	2	531	58,9	9,64
P47914	60S ribosomal protein L29 OS=Homo sapiens OX=9606 GN=RPL29 PE=1 SV=2 - [RL29_HUMAN]	14,47	1	2	2	2	3,524E6	2,257E8	2,24	5,03	1	1	3,57	9,43	1	1	159	17,7	11,66
Q8WYP5	Protein ELYS OS=Homo sapiens OX=9606 GN=AHCTF1 PE=1 SV=3 - [ELYS_HUMAN]	1,77	1	2	2	2	0,000E0	1,770E6		0,00			3,56	1,77	2	2	2266	252,3	6,60
Q9H7X3	Zinc finger protein 696 OS=Homo sapiens OX=9606 GN=ZNF696 PE=2 SV=2 - [ZN696_HUMAN]	6,68	1	1	1	1	0,000E0	2,037E7		0,00			3,53	6,68	1	1	374	40,5	8,92
A6NF01	Putative nuclear envelope pore membrane protein POM 121B OS=Homo sapiens OX=9606 GN=POM121B I	2,40	3	2	2	2	0,000E0	3,648E6		0,00			3,53	2,40	2	2	834	83,0	9,95
Q96CX3	Zinc finger protein 501 OS=Homo sapiens OX=9606 GN=ZNF501 PE=2 SV=2 - [ZN501_HUMAN]	4,80	1	1	1	1	0,000E0	1,294E7		0,00			3,42	4,80	1	1	271	31,2	9,19
Q8TF47	Zinc finger protein 90 homolog OS=Homo sapiens OX=9606 GN=ZFP90 PE=1 SV=2 - [ZFP90_HUMAN]	2,52	1	1	1	1	0,000E0	3,733E6		0,00			3,37	2,52	1	1	636	73,0	8,06
Q9NW07	Zinc finger protein 358 OS=Homo sapiens OX=9606 GN=ZNF358 PE=1 SV=2 - [ZN358_HUMAN]	4,05	1	2	2	2	0,000E0	1,060E7		0,00			3,34	4,05	2	2	568	59,2	6,60
P62318	Small nuclear ribonucleoprotein Sm D3 OS=Homo sapiens OX=9606 GN=SNRPD3 PE=1 SV=1 - [SMD3_I	7,94	1	1	1	1	0,000E0	1,097E7		0,00			3,28	7,94	1	1	126	13,9	10,32
O60304	Zinc finger protein 500 OS=Homo sapiens OX=9606 GN=ZNF500 PE=2 SV=2 - [ZN500_HUMAN]	3,33	1	1	1	1	0,000E0	3,880E6		0,00			3,24	3,33	1	1	480	53,6	7,24
P62891	60S ribosomal protein L39 OS=Homo sapiens OX=9606 GN=RPL39 PE=1 SV=2 - [RL39_HUMAN]	19,61	2	1	1	1	0,000E0	1,059E8					3,24	19,61	1	1	51	6,4	12,56
P63279	SUMO-conjugating enzyme UBC9 OS=Homo sapiens OX=9606 GN=UBE2I PE=1 SV=1 - [UBC9_HUMAN	7,59	1	1	1	1	0,000E0	6,767E7		0,00			3,21	7,59	1	1	158	18,0	8,66
Q86V81	THO complex subunit 4 OS=Homo sapiens OX=9606 GN=ALYREF PE=1 SV=3 - [THOC4_HUMAN]	14,79	1	3	3	3	7,831E6	9,527E6	8,82	10,51	2	2	3,20	4,28	1	1	257	26,9	11,15
Q5EB52	Mesoderm-specific transcript homolog protein OS=Homo sapiens OX=9606 GN=MEST PE=2 SV=2 - [MES	5,37	1	1	1	1	0,000E0	1,768E6		0,00			3,19	5,37	1	1	335	38,8	9,74
P48634	Protein PRRC2A OS=Homo sapiens OX=9606 GN=PRRC2A PE=1 SV=3 - [PRC2A_HUMAN]	0,60	1	1	1	1	0,000E0	1,959E6		0,00			3,18	0,60	1	1	2157	228,7	9,45
Q15366	Poly(rC)-binding protein 2 OS=Homo sapiens OX=9606 GN=PCBP2 PE=1 SV=1 - [PCBP2_HUMAN]	11,78	2	1	2	3	1,318E7	1,206E7	0,00	6,30	1	1	3,18	11,78	2	2	365	38,6	6,79
Q86X55	Histone-arginine methyltransferase CARM1 OS=Homo sapiens OX=9606 GN=CARM1 PE=1 SV=3 - [CAR	3,78	1	2	2	2	0,000E0	9,672E6		0,00			3,17	3,78	2	2	608	65,8	6,73
Q8N5C8	TGF-beta-activated kinase 1 and MAP3K7-binding protein 3 OS=Homo sapiens OX=9606 GN=TAB3 PE=1	2,39	1	1	1	1	0,000E0	4,026E6		0,00			3,15	2,39	1	1	712	78,6	8,54
Q15942	Zyxin OS=Homo sapiens OX=9606 GN=ZYX PE=1 SV=1 - [ZYX_HUMAN]	3,32	1	1	1	1	0,000E0	0,000E0		0,00			3,14	3,32	1	1	572	61,2	6,67
O15371	Eukaryotic translation initiation factor 3 subunit D OS=Homo sapiens OX=9606 GN=EIF3D PE=1 SV=1 - [	3,65	1	2	2	2	1,408E6	4,030E6	0,00	1,82	1	1	3,13	1,82	1	1	548	63,9	6,05
Q12962	Transcription initiation factor TFIID subunit 10 OS=Homo sapiens OX=9606 GN=TAF10 PE=1 SV=1 - [TA	11,47	1	1	1	1	0,000E0	1,310E6		0,00			3,13	11,47	1	1	218	21,7	6,57
Q9NS69	Mitochondrial import receptor subunit TOM22 homolog OS=Homo sapiens OX=9606 GN=TOMM22 PE=1	8,45	1	1	1	1	0,000E0	5,618E6					3,13	8,45	1	1	142	15,5	4,34
P41091	Eukaryotic translation initiation factor 2 subunit 3 OS=Homo sapiens OX=9606 GN=EIF2S3 PE=1 SV=3 - [	2,33	2	1	1	1	0,000E0	1,271E7		0,00			3,12	2,33	1	1	472	51,1	8,40
Q16600	Zinc finger protein 239 OS=Homo sapiens OX=9606 GN=ZNF239 PE=1 SV=3 - [ZN239_HUMAN]	5,46	1	2	2	2	0,000E0	6,127E6		0,00			3,11	5,46	2	2	458	51,6	7,64
Q9BPX5	Actin-related protein 2/3 complex subunit 5-like protein OS=Homo sapiens OX=9606 GN=ARPC5L PE=1 S	8,50	1	1	1	2	6,220E5	5,622E6	1,87	8,50	1	1	3,07	8,50	1	1	153	16,9	6,60
P53985	Monocarboxylate transporter 1 OS=Homo sapiens OX=9606 GN=SLC16A1 PE=1 SV=3 - [MOT1_HUMAN	2,40	1	1	1	1	0,000E0	1,065E7		0,00			3,03	2,40	1	1	500	53,9	8,66
Q8IZ26	Zinc finger protein 34 OS=Homo sapiens OX=9606 GN=ZNF34 PE=1 SV=3 - [ZNF34_HUMAN]	4,64	1	2	2	2	0,000E0	2,283E6		0,00			2,99	4,64	2	2	560	64,0	8,78
O75190	DnaJ homolog subfamily B member 6 OS=Homo sapiens OX=9606 GN=DNAJB6 PE=1 SV=2 - [DNJB6_H	5,21	1	1	1	1	0,000E0	1,781E6		0,00			2,99	5,21	1	1	326	36,1	9,16
P49757	Protein numb homolog OS=Homo sapiens OX=9606 GN=NUMB PE=1 SV=2 - [NUMB_HUMAN]	2,30	1	1	1	1	0,000E0	7,008E6		0,00			2,99	2,30	1	1	651	70,8	8,51
Q9BRJ6	Uncharacterized protein C7orf50 OS=Homo sapiens OX=9606 GN=C7orf50 PE=1 SV=1 - [CG050_HUMAN	11,34	1	1	1	1	0,000E0	0,000E0		0,00			2,98	11,34	1	1	194	22,1	9,64
P05198	Eukaryotic translation initiation factor 2 subunit 1 OS=Homo sapiens OX=9606 GN=EIF2S1 PE=1 SV=3 - [	9,52	1	3	3	3	1,356E7	7,997E6	2,07	3,17	1	1	2,97	6,35	2	2	315	36,1	5,08
P40227	T-complex protein 1 subunit zeta OS=Homo sapiens OX=9606 GN=CCT6A PE=1 SV=3 - [TCPZ_HUMAN	12,99	1	5	5	6	9,837E6	8,152E5	10,32	11,86	4	4	2,97	5,08	2	2	531	58,0	6,68
P51784	Ubiquitin carboxyl-terminal hydrolase 11 OS=Homo sapiens OX=9606 GN=USP11 PE=1 SV=3 - [UBP11_I	1,66	1	1	1	1	0,000E0	2,304E6		0,00			2,96	1,66	1	1	963	109,7	5,45
Q8TBX8	Phosphatidylinositol 5-phosphate 4-kinase type-2 gamma OS=Homo sapiens OX=9606 GN=PIP4K2C PE=1	3,33	1	1	1	1	0,000E0	4,875E6		0,00			2,95	3,33	1	1	421	47,3	6,84
Q13077	TNF receptor-associated factor 1 OS=Homo sapiens OX=9606 GN=TRAF1 PE=1 SV=1 - [TRAF1_HUMAN	9,62	1	4	4	4	0,000E0	6,851E6		0,00			2,94	9,62	4	4	416	46,1	6,11
Q8IWY8	Zinc finger and SCAN domain-containing protein 29 OS=Homo sapiens OX=9606 GN=ZSCAN29 PE=1 SV	1,29	1	1	1	1	0,000E0	2,832E6		0,00			2,93	1,29	1	1	852	96,7	6,99
Q15072	Zinc finger protein OZF OS=Homo sapiens OX=9606 GN=ZNF146 PE=1 SV=2 - [OZF_HUMAN]	4,11	1	1	1	1	0,000E0	3,870E7		0,00			2,92	4,11	1	1	292	33,3	8,88
P17302	Gap junction alpha-1 protein OS=Homo sapiens OX=9606 GN=GJA1 PE=1 SV=2 - [CXA1_HUMAN]	3,93	1	1	1	1	0,000E0	4,166E6		0,00			2,91	3,93	1	1	382	43,0	8,76
Q9NX63	MICOS complex subunit MIC19 OS=Homo sapiens OX=9606 GN=CHCHD3 PE=1 SV=1 - [MIC19_HUM	3,52	1	1	1	1	0,000E0	4,013E7		0,00			2,90	3,52	1	1	227	26,1	8,28
P62191	26S proteasome regulatory subunit 4 OS=Homo sapiens OX=9606 GN=PSMC1 PE=1 SV=1 - [PRS4_HUM	2,73	1	1	1	1	0,000E0	2,580E6		0,00			2,90	2,73	1	1	440	49,2	6,21
P67870	Casein kinase II subunit beta OS=Homo sapiens OX=9606 GN=CSNK2B PE=1 SV=1 - [CSK2B_HUMAN]	6,51	1	1	1	2	2,182E6	7,832E6	0,00	6,51	1	1	2,87	6,51	1	1	215	24,9	5,55
P06733	Alpha-enolase OS=Homo sapiens OX=9606 GN=ENO1 PE=1 SV=2 - [ENOA_HUMAN]	46,08	2	11	13	29	9,711E7	6,531E6	45,25	46,08	13	27	2,86	5,30	2	2	434	47,1	7,39
P01040	Cystatin-A OS=Homo sapiens OX=9606 GN=CSTA PE=1 SV=1 - [CYTA_HUMAN]	37,76	1	3	3	6	8,057E7	3,954E7	9,86	37,76	3	5	2,85	18,37	1	1	98	11,0	5,50
Q16204	Coiled-coil domain-containing protein 6 OS=Homo sapiens OX=9606 GN=CCDC6 PE=1 SV=2 - [CCDC6_	3,59	1	1	1	1	0,000E0	2,782E6		0,00			2,84	3,59	1	1	474	53,3	7,34
P17096	High mobility group protein HMG-I/HMG-Y OS=Homo sapiens OX=9606 GN=HMGA1 PE=1 SV=3 - [HM	14,02	1	1	1	1	0,000E0	2,293E7		0,00			2,84	14,02	1	1	107	11,7	10,32
P55769	NHP2-like protein 1 OS=Homo sapiens OX=9606 GN=SNU13 PE=1 SV=3 - [NH2L1_HUMAN]	9,38	1	1	1	2	5,030E5	2,219E6	0,00	9,38	1	1	2,82	9,38	1	1	128	14,2	8,46
P63220	40S ribosomal protein S21 OS=Homo sapiens OX=9606 GN=RPS21 PE=1 SV=1 - [RS21_HUMAN]	12,05	1	1	1	2	4,923E5	0,000E0	0,00	12,05	1	1	2,82	12,05	1	1	83	9,1	8,50
Q92766	Ras-responsive element-binding protein 1 OS=Homo sapiens OX=9606 GN=RREB1 PE=1 SV=3 - [RREB1	1,19	1	1	1	1	0,000E0	2,783E6		0,00			2,79	1,19	1	1	1687	181,3	6,98
Q8TF39	Zinc finger protein 483 OS=Homo sapiens OX=9606 GN=ZNF483 PE=1 SV=3 - [ZN483_HUMAN]	2,15	1	1	1	1	0,000E0	7,797E6		0,00			2,79	2,15	1	1	744	85,0	8,57
Q15717	ELAV-like protein 1 OS=Homo sapiens OX=9606 GN=ELAVL1 PE=1 SV=2 - [ELAV1_HUMAN]	3,99	1	1	1	1	0,000E0	0,000E0		0,00			2,79	3,99	1	1	326	36,1	9,17
Q96E35	Zinc finger MYND domain-containing protein 19 OS=Homo sapiens OX=9606 GN=ZMYND19 PE=1 SV=1	4,85	1	1	1	1	0,000E0	2,102E6		0,00			2,78	4,85	1	1	227	26,4	6,86
O60573	Eukaryotic translation initiation factor 4E type 2 OS=Homo sapiens OX=9606 GN=EIF4E2 PE=1 SV=1 - [IF	6,53	1	1	1	1	0,000E0	3,368E6		0,00			2,78	6,53	1	1	245	28,3	8,88
Q92499	ATP-dependent RNA helicase DDX1 OS=Homo sapiens OX=9606 GN=DDX1 PE=1 SV=2 - [DDX1_HUM	12,97	1	6	6	6	4,442E6	4,728E6	5,35	10,27	4	4	2,78	2,70	2	2	740	82,4	7,23
Q9NQC3	Reticulon-4 OS=Homo sapiens OX=9606 GN=RTN4 PE=1 SV=2 - [RTN4_HUMAN]	1,09	1	1	1	2	1,809E6	5,668E6	0,00	1,09	1	1	2,77	1,09	1	1	1192	129,9	4,50
Q04637	Eukaryotic translation initiation factor 4 gamma 1 OS=Homo sapiens OX=9606 GN=EIF4G1 PE=1 SV=4 - [	4,25	2	6	6	6	6,746E5	3,692E6	1,68	2,94	4	4	2,75	1,31	2	2	1599	175,4	5,33
Q7L099	Protein RUFY3 OS=Homo sapiens OX=9606 GN=RUFY3 PE=1 SV=1 - [RUFY3_HUMAN]	2,35	1	1	1	1	0,000E0	0,000E0		0,00			2,74	2,35	1	1	469	52,9	5,49
Q9NY12	H/ACA ribonucleoprotein complex subunit 1 OS=Homo sapiens OX=9606 GN=GAR1 PE=1 SV=1 - [GAR1	4,15	1	1	1	1	0,000E0	5,350E6		0,00			2,74	4,15	1	1	217	22,3	10,92
Q99497	Protein/nucleic acid deglycase DJ-1 OS=Homo sapiens OX=9606 GN=PARK7 PE=1 SV=2 - [PARK7_HUM	14,81	1	2	2	3	6,537E6	1,112E6	7,77	14,81	2	2	2,73	10,05	1	1	189	19,9	6,79
Q9Y2H2	Phosphatidylinositide phosphatase SAC2 OS=Homo sapiens OX=9606 GN=INPP5F PE=1 SV=3 - [SAC2_I	3,00	1	2	2	2	0,000E0	7,201E6		0,00			2,73	3,00	2	2	1132	128,3	7,02
P62857	40S ribosomal protein S28 OS=Homo sapiens OX=9606 GN=RPS28 PE=1 SV=1 - [RS28_HUMAN]	17,39	1	1	1	1	0,000E0	9,473E7		0,00			2,73	17,39	1	1	69	7,8	10,70
Q9GZM3	DNA-directed RNA polymerase II subunit RPB11-b1 OS=Homo sapiens OX=9606 GN=POLR2J2 PE=1 SV	8,70	3	1	1	1	0,000E0	2,097E7		0,00			2,69	8,70	1	1	115	13,1	6,77
Q9H6F5	Coiled-coil domain-containing protein 86 OS=Homo sapiens OX=9606 GN=CCDC86 PE=1 SV=1 - [CCD8	3,61	1	1	1	1	0,000E0	1,836E6		0,00			2,69	3,61	1	1	360	40,2	10,33
P46778	60S ribosomal protein L21 OS=Homo sapiens OX=9606 GN=RPL21 PE=1 SV=2 - [RL21_HUMAN]	9,38	1	1	1	1	0,000E0	7,155E6		0,00			2,68	9,38	1	1	160	18,6	10,49
O00303	Eukaryotic translation initiation factor 3 subunit F OS=Homo sapiens OX=9606 GN=EIF3F PE=1 SV=1 - [E	10,08	1	2	2	2	7,842E6	0,000E0	5,53	5,32	1	1	2,67	4,76	1	1	357	37,5	5,45
P17676	CCAAT/enhancer-binding protein beta OS=Homo sapiens OX=9606 GN=CEBPB PE=1 SV=2 - [CEBPB_H	2,90	1	1	1	1	0,000E0	1,976E7		0,00			2,66	2,90	1	1	345	36,1	8,31
Q9UDY2	Tight junction protein ZO-2 OS=Homo sapiens OX=9606 GN=TJP2 PE=1 SV=2 - [ZO2_HUMAN]	0,92	1	1	1	1	0,000E0	1,738E6		0,00			2,66	0,92	1	1	1190	133,9	7,40
Q7Z6J0	E3 ubiquitin-protein ligase SH3RF1 OS=Homo sapiens OX=9606 GN=SH3RF1 PE=1 SV=2 - [SH3R1_HU	1,80	1	1	1	1	0,000E0	5,050E6		0,00			2,65	1,80	1	1	888	93,1	8,54
P04843	Dolichyl-diphosphooligosaccharide--protein glycosyltransferase subunit 1 OS=Homo sapiens OX=9606 GN=I	6,26	1	3	3	4	4,953E6	5,868E6	0,00	4,45	2	2	2,65	3,79	2	2	607	68,5	6,38
O15550	Lysine-specific demethylase 6A OS=Homo sapiens OX=9606 GN=KDM6A PE=1 SV=2 - [KDM6A_HUMA	0,79	1	1	1	1	0,000E0	9,721E5		0,00			2,64	0,79	1	1	1401	154,1	7,44
Q9BQE9	B-cell CLL/lymphoma 7 protein family member B OS=Homo sapiens OX=9606 GN=BCL7B PE=1 SV=1 -	4,95	3	1	1	1	0,000E0	4,104E6					2,64	4,95	1	1	202	22,2	4,75
Q14257	Reticulocalbin-2 OS=Homo sapiens OX=9606 GN=RCN2 PE=1 SV=1 - [RCN2_HUMAN]	3,47	1	1	1	1	0,000E0	1,331E6					2,64	3,47	1	1	317	36,9	4,40
P62873	Guanine nucleotide-binding protein G(I)/G(S)/G(T) subunit beta-1 OS=Homo sapiens OX=9606 GN=GNB1 I	3,24	3	1	1	1	0,000E0	0,000E0		0,00			2,63	3,24	1	1	340	37,4	6,00
Q8WUM0	Nuclear pore complex protein Nup133 OS=Homo sapiens OX=9606 GN=NUP133 PE=1 SV=2 - [NU133_HU	1,04	1	1	1	1	0,000E0	2,748E6		0,00			2,63	1,04	1	1	1156	128,9	5,10
Q96JF6	Zinc finger protein 594 OS=Homo sapiens OX=9606 GN=ZNF594 PE=2 SV=3 - [ZN594_HUMAN]	1,61	1	1	1	1	0,000E0	3,787E6		0,00			2,62	1,61	1	1	807	93,8	8,70
Q3KNS6	Zinc finger protein 829 OS=Homo sapiens OX=9606 GN=ZNF829 PE=2 SV=1 - [ZN829_HUMAN]	7,64	1	3	3	3	0,000E0	7,754E6		0,00			2,62	7,64	3	3	432	50,1	7,93
P29728	2'-5'-oligoadenylate synthase 2 OS=Homo sapiens OX=9606 GN=OAS2 PE=1 SV=3 - [OAS2_HUMAN]	1,39	1	1	1	1	0,000E0	5,378E6		0,00			2,62	1,39	1	1	719	82,4	8,25
Q9NVM9	Integrator complex subunit 13 OS=Homo sapiens OX=9606 GN=INTS13 PE=1 SV=2 - [INT13_HUMAN]	1,56	1	1	1	1	0,000E0	6,129E6		0,00			2,62	1,56	1	1	706	80,2	6,70
P25311	Zinc-alpha-2-glycoprotein OS=Homo sapiens OX=9606 GN=AZGP1 PE=1 SV=2 - [ZA2G_HUMAN]	12,08	1	3	3	5	2,971E7	4,921E6	4,57	12,08	3	4	2,62	5,70	1	1	298	34,2	6,05
Q9H307	Pinin OS=Homo sapiens OX=9606 GN=PNN PE=1 SV=5 - [PININ_HUMAN]	2,09	1	1	1	1	0,000E0	2,870E6		0,00			2,61	2,09	1	1	717	81,6	7,14
Q96H40	Zinc finger protein 486 OS=Homo sapiens OX=9606 GN=ZNF486 PE=2 SV=4 - [ZN486_HUMAN]	2,16	2	1	1	1	0,000E0	6,077E6		0,00			2,61	2,16	1	1	463	53,6	9,10
O95292	Vesicle-associated membrane protein-associated protein B/C OS=Homo sapiens OX=9606 GN=VAPB PE=1	4,94	1	1	1	1	0,000E0	3,467E6		0,00			2,60	4,94	1	1	243	27,2	7,30
O15511	Actin-related protein 2/3 complex subunit 5 OS=Homo sapiens OX=9606 GN=ARPC5 PE=1 SV=3 - [ARPC	16,56	1	3	3	4	8,430E5	3,604E6	1,82	7,95	1	1	2,59	16,56	3	3	151	16,3	5,67
A6NP11	Zinc finger protein 716 OS=Homo sapiens OX=9606 GN=ZNF716 PE=2 SV=4 - [ZN716_HUMAN]	4,24	1	2	2	2	0,000E0	3,103E6		0,00			2,58	4,24	2	2	495	57,0	9,17
Q9BWE0	Replication initiator 1 OS=Homo sapiens OX=9606 GN=REPIN1 PE=1 SV=1 - [REPI1_HUMAN]	4,76	1	2	2	2	0,000E0	2,848E6		0,00			2,58	4,76	2	2	567	63,5	9,98
P06702	Protein S100-A9 OS=Homo sapiens OX=9606 GN=S100A9 PE=1 SV=1 - [S10A9_HUMAN]	37,72	1	3	3	11	1,356E8	1,633E7	20,69	37,72	3	10	2,58	11,40	1	1	114	13,2	6,13
Q4KMQ1	Taperin OS=Homo sapiens OX=9606 GN=TPRN PE=1 SV=2 - [TPRN_HUMAN]	2,53	1	1	1	1	0,000E0	2,606E6		0,00			2,57	2,53	1	1	711	75,5	7,25

Q7Z417	Nuclear fragile X mental retardation-interacting protein 2 OS=Homo sapiens OX=9606 GN=NUFIP2 PE=1 S	3,88	1	2	2	2	0,000E0	1,302E7		0,00			2,57	3,88	2	2	695	76,1	8,70
Q96T51	RUN and FYVE domain-containing protein 1 OS=Homo sapiens OX=9606 GN=RUFY1 PE=1 SV=2 - [RUI	3,11	1	2	2	2	0,000E0	4,625E6		0,00			2,56	3,11	2	2	708	79,8	5,74
P46976	Glycogenin-1 OS=Homo sapiens OX=9606 GN=GYG1 PE=1 SV=4 - [GLYG_HUMAN]	5,14	1	1	1	1	0,000E0	0,000E0		0,00			2,55	5,14	1	1	350	39,4	5,53
Q9Y383	Putative RNA-binding protein Luc7-like 2 OS=Homo sapiens OX=9606 GN=LUC7L2 PE=1 SV=2 - [LC7L2	3,83	1	1	1	1	0,000E0	1,042E7		0,00			2,55	3,83	1	1	392	46,5	10,01
Q9P021	Cysteine-rich PDZ-binding protein OS=Homo sapiens OX=9606 GN=CRIPT PE=1 SV=1 - [CRIPT_HUMA	10,89	1	1	1	1	0,000E0	1,521E7		0,00			2,54	10,89	1	1	101	11,2	9,47
P51572	B-cell receptor-associated protein 31 OS=Homo sapiens OX=9606 GN=BCAP31 PE=1 SV=3 - [BAP31_HU	4,47	1	1	1	1	0,000E0	1,081E7		0,00			2,53	4,47	1	1	246	28,0	8,44
P40926	Malate dehydrogenase, mitochondrial OS=Homo sapiens OX=9606 GN=MDH2 PE=1 SV=3 - [MDHM_HUM	7,69	1	2	2	3	1,707E6	4,352E6	3,89	7,69	2	2	2,53	3,55	1	1	338	35,5	8,68
Q9H6T3	RNA polymerase II-associated protein 3 OS=Homo sapiens OX=9606 GN=RPAP3 PE=1 SV=2 - [RPAP3_H	4,51	1	2	2	2	0,000E0	6,840E6		0,00			2,52	4,51	2	2	665	75,7	6,84
Q9Y265	RuvB-like 1 OS=Homo sapiens OX=9606 GN=RUVBL1 PE=1 SV=1 - [RUVB1_HUMAN]	3,07	1	1	1	1	0,000E0	5,042E6		0,00			2,52	3,07	1	1	456	50,2	6,42
P49848	Transcription initiation factor TFIID subunit 6 OS=Homo sapiens OX=9606 GN=TAF6 PE=1 SV=1 - [TAF6	2,07	1	1	1	1	0,000E0	1,970E6		0,00			2,51	2,07	1	1	677	72,6	8,60
O95376	E3 ubiquitin-protein ligase ARIH2 OS=Homo sapiens OX=9606 GN=ARIH2 PE=1 SV=1 - [ARI2_HUMAN	3,04	1	1	1	1	0,000E0	4,189E6		0,00			2,51	3,04	1	1	493	57,8	5,63
P26196	Probable ATP-dependent RNA helicase DDX6 OS=Homo sapiens OX=9606 GN=DDX6 PE=1 SV=2 - [DDX	2,28	1	1	1	1	0,000E0	6,729E6		0,00			2,50	2,28	1	1	483	54,4	8,66
Q9H0M5	Zinc finger protein 700 OS=Homo sapiens OX=9606 GN=ZNF700 PE=2 SV=1 - [ZN700_HUMAN]	1,89	1	1	1	1	0,000E0	2,264E6		0,00			2,50	1,89	1	1	742	86,2	8,90
O14771	Zinc finger protein 213 OS=Homo sapiens OX=9606 GN=ZNF213 PE=2 SV=2 - [ZN213_HUMAN]	9,37	1	3	3	3	0,000E0	3,190E6		0,00			2,50	9,37	3	3	459	51,2	6,95
Q9HCX3	Zinc finger protein 304 OS=Homo sapiens OX=9606 GN=ZNF304 PE=1 SV=2 - [ZN304_HUMAN]	1,37	1	1	1	1	0,000E0	8,417E6		0,00			2,48	1,37	1	1	659	75,0	8,35
P11387	DNA topoisomerase 1 OS=Homo sapiens OX=9606 GN=TOP1 PE=1 SV=2 - [TOP1_HUMAN]	1,31	1	1	1	1	0,000E0	6,452E6		0,00			2,48	1,31	1	1	765	90,7	9,31
P00734	Prothrombin OS=Homo sapiens OX=9606 GN=F2 PE=1 SV=2 - [THRB_HUMAN]	1,45	1	1	1	1	0,000E0	7,427E6		0,00			2,48	1,45	1	1	622	70,0	5,90
Q15345	Leucine-rich repeat-containing protein 41 OS=Homo sapiens OX=9606 GN=LRRC41 PE=1 SV=3 - [LRC41	1,72	1	1	1	1	0,000E0	0,000E0		0,00			2,48	1,72	1	1	812	88,6	8,38
Q8NBJ5	Procollagen galactosyltransferase 1 OS=Homo sapiens OX=9606 GN=COLGALT1 PE=1 SV=1 - [GT251_HU	3,70	1	2	2	2	0,000E0	1,369E6		0,00			2,46	3,70	2	2	622	71,6	7,31
P50991	T-complex protein 1 subunit delta OS=Homo sapiens OX=9606 GN=CCT4 PE=1 SV=4 - [TCPD_HUMAN]	12,43	1	4	5	5	1,155E6	7,002E6	3,86	8,72	3	3	2,46	3,71	2	2	539	57,9	7,83
Q15291	Retinoblastoma-binding protein 5 OS=Homo sapiens OX=9606 GN=RBBP5 PE=1 SV=2 - [RBBP5_HUMA	3,90	1	1	1	1	0,000E0	8,541E6		0,00			2,46	3,90	1	1	538	59,1	5,10
Q9H0D6	5'-3' exoribonuclease 2 OS=Homo sapiens OX=9606 GN=XRN2 PE=1 SV=1 - [XRN2_HUMAN]	4,32	1	3	3	3	3,703E6	1,543E6	3,14	1,58	1	1	2,46	2,74	2	2	950	108,5	7,47
P06396	Gelsolin OS=Homo sapiens OX=9606 GN=GSN PE=1 SV=1 - [GELS_HUMAN]	2,43	1	2	2	2	2,157E6	1,084E7	2,27	1,02	1	1	2,46	1,41	1	1	782	85,6	6,28
O14908	PDZ domain-containing protein GIPC1 OS=Homo sapiens OX=9606 GN=GIPC1 PE=1 SV=2 - [GIPC1_HU	9,91	1	1	1	1	0,000E0	7,160E6		0,00			2,45	9,91	1	1	333	36,0	6,28
Q6P2D0	Zinc finger protein 1 homolog OS=Homo sapiens OX=9606 GN=ZFP1 PE=1 SV=2 - [ZFP1_HUMAN]	2,95	1	1	1	1	0,000E0	6,182E5		0,00			2,44	2,95	1	1	407	47,5	8,57
Q9H992	E3 ubiquitin-protein ligase MARCH7 OS=Homo sapiens OX=9606 GN=MARCH7 PE=1 SV=1 - [MARH7_	1,85	1	1	1	1	0,000E0	5,086E6		0,00			2,44	1,85	1	1	704	78,0	6,77
P30050	60S ribosomal protein L12 OS=Homo sapiens OX=9606 GN=RPL12 PE=1 SV=1 - [RL12_HUMAN]	18,79	1	2	2	4	1,967E6	9,800E6	1,80	9,09	1	3	2,44	9,70	1	1	165	17,8	9,42
P61962	DDB1- and CUL4-associated factor 7 OS=Homo sapiens OX=9606 GN=DCAF7 PE=1 SV=1 - [DCAF7_HU	6,73	1	2	2	2	0,000E0	4,149E6		0,00			2,43	6,73	2	2	342	38,9	5,52
P04004	Vitronectin OS=Homo sapiens OX=9606 GN=VTN PE=1 SV=1 - [VTNC_HUMAN]	3,14	1	1	1	2	1,770E6	6,565E7	0,00	3,14	1	1	2,43	3,14	1	1	478	54,3	5,80
Q96C55	Zinc finger protein 524 OS=Homo sapiens OX=9606 GN=ZNF524 PE=1 SV=1 - [ZN524_HUMAN]	3,79	1	1	1	1	0,000E0	1,614E7					2,42	3,79	1	1	264	28,7	8,75
Q9H9B4	Sideroflexin-1 OS=Homo sapiens OX=9606 GN=SFXN1 PE=1 SV=4 - [SFXN1_HUMAN]	3,73	1	1	1	1	0,000E0	1,017E7		0,00			2,42	3,73	1	1	322	35,6	9,07
Q9H0H5	Rac GTPase-activating protein 1 OS=Homo sapiens OX=9606 GN=RACGAP1 PE=1 SV=1 - [RGAP1_HUM	4,11	1	3	3	3	0,000E0	4,715E6		0,00			2,41	4,11	3	3	632	71,0	8,88
P41743	Protein kinase C iota type OS=Homo sapiens OX=9606 GN=PRKCI PE=1 SV=2 - [KPCI_HUMAN]	2,35	1	1	1	1	0,000E0	0,000E0		0,00			2,41	2,35	1	1	596	68,2	5,85
Q68DY9	Zinc finger protein 772 OS=Homo sapiens OX=9606 GN=ZNF772 PE=1 SV=2 - [ZN772_HUMAN]	1,84	1	1	1	1	0,000E0	2,074E7		0,00			2,40	1,84	1	1	489	55,3	7,71
O15164	Transcription intermediary factor 1-alpha OS=Homo sapiens OX=9606 GN=TRIM24 PE=1 SV=3 - [TIF1A_I	0,95	1	1	1	1	0,000E0	1,243E6		0,00			2,40	0,95	1	1	1050	116,8	7,11
Q8NBQ5	Estradiol 17-beta-dehydrogenase 11 OS=Homo sapiens OX=9606 GN=HSD17B11 PE=1 SV=3 - [DHB11_H	6,00	1	1	1	1	0,000E0	0,000E0		0,00			2,38	6,00	1	1	300	32,9	9,07
O14880	Microsomal glutathione S-transferase 3 OS=Homo sapiens OX=9606 GN=MGST3 PE=1 SV=1 - [MGST3_I	9,21	1	1	1	1	0,000E0	0,000E0		0,00			2,38	9,21	1	1	152	16,5	9,38
P52292	Importin subunit alpha-1 OS=Homo sapiens OX=9606 GN=KPNA2 PE=1 SV=1 - [IMA1_HUMAN]	9,07	1	3	3	3	3,233E6	5,083E6	0,00	4,16	1	1	2,38	4,91	2	2	529	57,8	5,40
P62861	40S ribosomal protein S30 OS=Homo sapiens OX=9606 GN=FAU PE=1 SV=1 - [RS30_HUMAN]	16,95	1	1	1	2	0,000E0	7,799E7		0,00			2,38	16,95	1	2	59	6,6	12,15
P19532	Transcription factor E3 OS=Homo sapiens OX=9606 GN=TFE3 PE=1 SV=4 - [TFE3_HUMAN]	1,74	1	1	1	1	0,000E0	1,989E7					2,37	1,74	1	1	575	61,5	5,58
Q9ULU4	Protein kinase C-binding protein 1 OS=Homo sapiens OX=9606 GN=ZMYND8 PE=1 SV=2 - [PKCB1_HU	1,18	1	1	1	1	0,000E0	3,408E6		0,00			2,35	1,18	1	1	1186	131,6	7,20
Q06033	Inter-alpha-trypsin inhibitor heavy chain H3 OS=Homo sapiens OX=9606 GN=ITIH3 PE=1 SV=2 - [ITIH3_I	1,12	1	1	1	1	0,000E0	7,748E6		0,00			2,34	1,12	1	1	890	99,8	5,74
P29401	Transketolase OS=Homo sapiens OX=9606 GN=TKT PE=1 SV=3 - [TKT_HUMAN]	6,58	1	3	3	5	1,314E7	2,634E6	9,32	6,58	3	4	2,34	1,93	1	1	623	67,8	7,66
P01008	Antithrombin-III OS=Homo sapiens OX=9606 GN=SERPINC1 PE=1 SV=1 - [ANT3_HUMAN]	1,94	1	1	1	1	0,000E0	4,920E6		0,00			2,33	1,94	1	1	464	52,6	6,71
P22087	rRNA 2'-O-methyltransferase fibrillarin OS=Homo sapiens OX=9606 GN=FBL PE=1 SV=2 - [FBRL_HUMA	3,43	2	1	1	1	0,000E0	4,035E6		0,00			2,32	3,43	1	1	321	33,8	10,18
Q9BS26	Endoplasmic reticulum resident protein 44 OS=Homo sapiens OX=9606 GN=ERP44 PE=1 SV=1 - [ERP44_	9,61	1	3	4	5	6,161E5	1,524E6	2,67	7,39	3	3	2,32	4,68	2	2	406	46,9	5,26
P18077	60S ribosomal protein L35a OS=Homo sapiens OX=9606 GN=RPL35A PE=1 SV=2 - [RL35A_HUMAN]	8,18	1	1	1	1	0,000E0	6,382E7		0,00			2,32	8,18	1	1	110	12,5	11,06
Q9UQR1	Zinc finger protein 148 OS=Homo sapiens OX=9606 GN=ZNF148 PE=1 SV=2 - [ZN148_HUMAN]	2,14	1	1	1	1	0,000E0	4,449E6		0,00			2,32	2,14	1	1	794	88,9	6,48
P55199	RNA polymerase II elongation factor ELL OS=Homo sapiens OX=9606 GN=ELL PE=1 SV=1 - [ELL_HUM	2,90	1	1	1	1	0,000E0	7,774E6		0,00			2,29	2,90	1	1	621	68,2	9,33
P27824	Calnexin OS=Homo sapiens OX=9606 GN=CANX PE=1 SV=2 - [CALX_HUMAN]	11,99	1	7	7	7	1,505E7	1,244E7	12,39	5,91	4	4	2,29	6,08	3	3	592	67,5	4,60
Q13642	Four and a half LIM domains protein 1 OS=Homo sapiens OX=9606 GN=FHL1 PE=1 SV=4 - [FHL1_HUM	3,41	1	1	1	1	0,000E0	1,716E7		0,00			2,29	3,41	1	1	323	36,2	8,97
P30101	Protein disulfide-isomerase A3 OS=Homo sapiens OX=9606 GN=PDIA3 PE=1 SV=4 - [PDIA3_HUMAN]	18,42	1	6	6	6	3,611E6	2,665E6	0,00	10,50	3	3	2,28	7,92	3	3	505	56,7	6,35
Q5VWQ0	Lysine-specific demethylase 9 OS=Homo sapiens OX=9606 GN=RSBN1 PE=1 SV=2 - [RSBN1_HUMAN]	2,00	1	1	1	1	0,000E0	2,237E6		0,00			2,28	2,00	1	1	802	90,0	8,60
P98082	Disabled homolog 2 OS=Homo sapiens OX=9606 GN=DAB2 PE=1 SV=3 - [DAB2_HUMAN]	2,34	1	1	1	1	0,000E0	2,607E5		0,00			2,27	2,34	1	1	770	82,4	5,53
Q92974	Rho guanine nucleotide exchange factor 2 OS=Homo sapiens OX=9606 GN=ARHGEF2 PE=1 SV=4 - [ARH	1,12	1	1	1	1	0,000E0	2,014E6		0,00			2,27	1,12	1	1	986	111,5	7,27
Q9H3U1	Protein unc-45 homolog A OS=Homo sapiens OX=9606 GN=UNC45A PE=1 SV=1 - [UN45A_HUMAN]	2,65	1	2	2	2	0,000E0	1,869E6		0,00			2,27	2,65	2	2	944	103,0	6,07
P61254	60S ribosomal protein L26 OS=Homo sapiens OX=9606 GN=RPL26 PE=1 SV=1 - [RL26_HUMAN]	17,24	2	3	3	4	7,358E6	1,549E8	7,07	17,24	3	3	2,26	6,21	1	1	145	17,2	10,55
P51911	Calponin-1 OS=Homo sapiens OX=9606 GN=CNN1 PE=1 SV=2 - [CNN1_HUMAN]	4,38	1	1	1	1	0,000E0	3,542E6		0,00			2,26	4,38	1	1	297	33,1	9,07
Q9NRE2	Teashirt homolog 2 OS=Homo sapiens OX=9606 GN=TSHZ2 PE=1 SV=3 - [TSH2_HUMAN]	1,35	1	1	1	1	0,000E0	0,000E0		0,00			2,26	1,35	1	1	1034	114,9	7,83
Q9Y3Y2	Chromatin target of PRMT1 protein OS=Homo sapiens OX=9606 GN=CHTOP PE=1 SV=2 - [CHTOP_HU	5,24	1	1	1	1	0,000E0	1,060E7		0,00			2,26	5,24	1	1	248	26,4	12,23
O14639	Actin-binding LIM protein 1 OS=Homo sapiens OX=9606 GN=ABLIM1 PE=1 SV=3 - [ABLM1_HUMAN]	1,80	1	1	1	1	0,000E0	0,000E0		0,00			2,25	1,80	1	1	778	87,6	8,59
P40939	Trifunctional enzyme subunit alpha, mitochondrial OS=Homo sapiens OX=9606 GN=HADHA PE=1 SV=2 -	7,34	1	4	4	4	9,656E6	2,864E6	3,78	6,16	3	3	2,25	1,18	1	1	763	82,9	9,04
P51571	Translocon-associated protein subunit delta OS=Homo sapiens OX=9606 GN=SSR4 PE=1 SV=1 - [SSRD_I	17,34	1	2	2	2	5,738E6	1,463E6	2,06	10,98	1	1	2,24	6,36	1	1	173	19,0	6,15
Q9H9B1	Histone-lysine N-methyltransferase EHMT1 OS=Homo sapiens OX=9606 GN=EHMT1 PE=1 SV=4 - [EHM	1,16	1	1	1	1	0,000E0	2,041E7		0,00			2,24	1,16	1	1	1298	141,4	5,76
P08621	U1 small nuclear ribonucleoprotein 70 kDa OS=Homo sapiens OX=9606 GN=SNRNP70 PE=1 SV=2 - [RU	3,66	1	1	1	1	0,000E0	6,273E6		0,00			2,23	3,66	1	1	437	51,5	9,94
O14628	Zinc finger protein 195 OS=Homo sapiens OX=9606 GN=ZNF195 PE=1 SV=2 - [ZN195_HUMAN]	3,82	1	1	1	1	0,000E0	1,861E7		0,00			2,23	3,82	1	1	629	72,3	8,09
P05023	Sodium/potassium-transporting ATPase subunit alpha-1 OS=Homo sapiens OX=9606 GN=ATP1A1 PE=1 S	4,79	4	3	3	3	1,950E6	4,686E6	2,45	2,25	1	1	2,22	2,54	2	2	1023	112,8	5,49
O15357	Phosphatidylinositol 3,4,5-trisphosphate 5-phosphatase 2 OS=Homo sapiens OX=9606 GN=INPPL1 PE=1 S	1,27	1	1	1	1	0,000E0	5,800E6		0,00			2,22	1,27	1	1	1258	138,5	6,54
Q16531	DNA damage-binding protein 1 OS=Homo sapiens OX=9606 GN=DDB1 PE=1 SV=1 - [DDB1_HUMAN]	5,35	1	4	4	4	3,042E6	1,088E6	2,26	2,11	2	2	2,22	3,25	2	2	1140	126,9	5,26
P61619	Protein transport protein Sec61 subunit alpha isoform 1 OS=Homo sapiens OX=9606 GN=SEC61A1 PE=1 S	1,89	2	1	1	1	0,000E0	1,270E7		0,00			2,21	1,89	1	1	476	52,2	8,06
Q9UBS4	DnaJ homolog subfamily B member 11 OS=Homo sapiens OX=9606 GN=DNAJB11 PE=1 SV=1 - [DJB11_	2,51	1	1	1	1	0,000E0	3,189E6		0,00			2,21	2,51	1	1	358	40,5	6,18
Q9UM00	Calcium load-activated calcium channel OS=Homo sapiens OX=9606 GN=TMCO1 PE=1 SV=1 - [TMCO1_	15,43	1	2	2	2	0,000E0	3,678E6		0,00			2,21	15,43	2	2	188	21,2	9,74
O43852	Calumenin OS=Homo sapiens OX=9606 GN=CALU PE=1 SV=2 - [CALU_HUMAN]	13,65	1	3	3	3	1,932E6	5,174E6	2,63	5,08	1	1	2,21	8,57	2	2	315	37,1	4,64
Q8NFW8	N-acylneuraminate cytidylyltransferase OS=Homo sapiens OX=9606 GN=CMAS PE=1 SV=2 - [NEUA_HUM	1,84	1	1	1	1	0,000E0	1,019E6		0,00			2,20	1,84	1	1	434	48,3	7,93
P01624	Immunoglobulin kappa variable 3-15 OS=Homo sapiens OX=9606 GN=IGKV3-15 PE=1 SV=2 - [KV315_H	7,83	2	1	1	2	9,191E7	1,199E8	2,27	7,83	1	1	2,20	7,83	1	1	115	12,5	5,19
Q12904	Aminoacyl tRNA synthase complex-interacting multifunctional protein 1 OS=Homo sapiens OX=9606 GN=A	3,85	1	1	1	2	3,403E5	1,137E6	0,00	3,85	1	1	2,20	3,85	1	1	312	34,3	8,43
P20042	Eukaryotic translation initiation factor 2 subunit 2 OS=Homo sapiens OX=9606 GN=EIF2S2 PE=1 SV=2 - [	10,21	1	2	2	2	0,000E0	5,636E6	0,00	5,71	1	1	2,20	4,50	1	1	333	38,4	5,80
P62993	Growth factor receptor-bound protein 2 OS=Homo sapiens OX=9606 GN=GRB2 PE=1 SV=1 - [GRB2_HUM	5,53	1	1	1	1	0,000E0	6,301E5		0,00			2,20	5,53	1	1	217	25,2	6,32
Q1KMD3	Heterogeneous nuclear ribonucleoprotein U-like protein 2 OS=Homo sapiens OX=9606 GN=HNRNPUL2 PE	7,23	1	3	3	4	2,752E7	7,196E6	0,00	6,16	2	2	2,19	4,02	2	2	747	85,1	4,91
Q9HC07	Transmembrane protein 165 OS=Homo sapiens OX=9606 GN=TMEM165 PE=1 SV=1 - [TM165_HUMAN]	5,56	1	1	1	1	0,000E0	2,779E6		0,00			2,19	5,56	1	1	324	34,9	7,02
P17027	Zinc finger protein 23 OS=Homo sapiens OX=9606 GN=ZNF23 PE=1 SV=3 - [ZNF23_HUMAN]	1,87	1	1	1	1	0,000E0	5,367E6		0,00			2,19	1,87	1	1	643	73,0	8,12
P53007	Tricarboxylate transport protein, mitochondrial OS=Homo sapiens OX=9606 GN=SLC25A1 PE=1 SV=2 - [T	2,89	1	1	1	1	0,000E0	5,095E6		0,00			2,19	2,89	1	1	311	34,0	9,89
P01859	Immunoglobulin heavy constant gamma 2 OS=Homo sapiens OX=9606 GN=IGHG2 PE=1 SV=2 - [IGHG2_	4,91	2	1	2	4	1,319E6	5,288E8	3,89	4,91	2	3	2,19	2,76	1	1	326	35,9	7,59
Q8NE71	ATP-binding cassette sub-family F member 1 OS=Homo sapiens OX=9606 GN=ABCF1 PE=1 SV=2 - [ABC	2,60	1	2	2	2	6,107E5	5,604E6	1,68	1,30	1	1	2,17	1,30	1	1	845	95,9	6,80
O75390	Citrate synthase, mitochondrial OS=Homo sapiens OX=9606 GN=CS PE=1 SV=2 - [CISY_HUMAN]	6,44	1	1	1	1	0,000E0	1,614E6		0,00			2,17	6,44	1	1	466	51,7	8,32
P55197	Protein AF-10 OS=Homo sapiens OX=9606 GN=MLLT10 PE=1 SV=2 - [AF10_HUMAN]	1,40	1	1	1	1	0,000E0	2,563E6		0,00			2,17	1,40	1	1	1068	113,3	8,21
P63241	Eukaryotic translation initiation factor 5A-1 OS=Homo sapiens OX=9606 GN=EIF5A PE=1 SV=2 - [IF5A1_	25,97	3	4	4	4	1,085E7	2,155E6	4,71	25,97	3	3	2,17	14,94	1	1	154	16,8	5,24
Q9C026	E3 ubiquitin-protein ligase TRIM9 OS=Homo sapiens OX=9606 GN=TRIM9 PE=1 SV=1 - [TRIM9_HUMA	2,25	1	1	1	1	0,000E0	0,000E0		0,00			2,17	2,25	1	1	710	79,1	6,84
P61513	60S ribosomal protein L37a OS=Homo sapiens OX=9606 GN=RPL37A PE=1 SV=2 - [RL37A_HUMAN]	19,57	1	1	1	1	0,000E0	7,906E6		0,00			2,17	19,57	1	1	92	10,3	10,43
Q9Y5A6	Zinc finger and SCAN domain-containing protein 21 OS=Homo sapiens OX=9606 GN=ZSCAN21 PE=1 SV	3,38	1	1	1	1	0,000E0	7,492E6		0,00			2,16	3,38	1	1	473	53,6	7,72
Q6P1L8	39S ribosomal protein L14, mitochondrial OS=Homo sapiens OX=9606 GN=MRPL14 PE=1 SV=1 - [RM14	9,66	1	1	1	1	0,000E0	5,039E6		0,00			2,16	9,66	1	1	145	15,9	10,24
P53350	Serine/threonine-protein kinase PLK1 OS=Homo sapiens OX=9606 GN=PLK1 PE=1 SV=1 - [PLK1_HUMA	3,15	1	1	1	1	0,000E0	6,024E6		0,00			2,15	3,15	1	1	603	68,2	8,91
P57088	Transmembrane protein 33 OS=Homo sapiens OX=9606 GN=TMEM33 PE=1 SV=2 - [TMM33_HUMAN]	4,86	1	1	1	1	0,000E0	1,371E6		0,00			2,15	4,86	1	1	247	28,0	9,70
Q07157	Tight junction protein ZO-1 OS=Homo sapiens OX=9606 GN=TJP1 PE=1 SV=3 - [ZO1_HUMAN]	1,43	1	2	2	2	9,882E5	0,000E0	0,00	0,69	1	1	2,15	0,74	1	1	1748	195,3	6,70
Q8WX93	Palladin OS=Homo sapiens OX=9606 GN=PALLD PE=1 SV=3 - [PALLD_HUMAN]	0,80	1	1	1	1	0,000E0	1,005E7		0,00			2,15	0,80	1	1	1383	150,5	7,09
O75427	Leucine-rich repeat and calponin homology domain-containing protein 4 OS=Homo sapiens OX=9606 GN=L	2,49	1	1	1	1	0,000E0	3,078E6		0,00			2,14	2,49	1	1	683	73,4	8,22
P05114	Non-histone chromosomal protein HMG-14 OS=Homo sapiens OX=9606 GN=HMGN1 PE=1 SV=3 - [HMG	13,00	1	1	1	1	0,000E0	1,136E6		0,00			2,14	13,00	1	1	100	10,7	9,60
P29317	Ephrin type-A receptor 2 OS=Homo sapiens OX=9606 GN=EPHA2 PE=1 SV=2 - [EPHA2_HUMAN]	2,05	1	2	2	2	0,000E0	1,694E6		0,00			2,13	2,05	2	2	976	108,2	6,23
O43390	Heterogeneous nuclear ribonucleoprotein R OS=Homo sapiens OX=9606 GN=HNRNPR PE=1 SV=1 - [HNR	4,58	1	1	2	2	3,768E6	3,511E6	0,00	2,69	1	1	2,13	1,90	1	1	633	70,9	8,13
Q14697	Neutral alpha-glucosidase AB OS=Homo sapiens OX=9606 GN=GANAB PE=1 SV=3 - [GANAB_HUMAN]	8,37	1	4	4	5	1,433E6	1,629E6	2,64	6,57	3	4	2,13	1,80	1	1	944	106,8	6,14
Q9NSI2	Protein FAM207A OS=Homo sapiens OX=9606 GN=FAM207A PE=1 SV=2 - [F207A_HUMAN]	6,09	1	1	1	1	0,000E0	2,086E6		0,00			2,13	6,09	1	1	230	25,4	11,08
P32119	Peroxiredoxin-2 OS=Homo sapiens OX=9606 GN=PRDX2 PE=1 SV=5 - [PRDX2_HUMAN]	18,18	1	3	3	3	3,664E7	1,690E6	1,90	5,05	1	1	2,12	13,13	2	2	198	21,9	5,97
Q92900	Regulator of nonsense transcripts 1 OS=Homo sapiens OX=9606 GN=UPF1 PE=1 SV=2 - [RENT1_HUMA	1,59	1	1	1	1	0,000E0	0,000E0		0,00			2,12	1,59	1	1	1129	124,3	6,61
Q96IZ0	PRKC apoptosis WT1 regulator protein OS=Homo sapiens OX=9606 GN=PAWR PE=1 SV=1 - [PAWR_HU	2,35	1	1	1	1	0,000E0	6,218E6		0,00			2,12	2,35	1	1	340	36,5	5,41
Q8WUA4	General transcription factor 3C polypeptide 2 OS=Homo sapiens OX=9606 GN=GTF3C2 PE=1 SV=2 - [TF3	1,21	1	1	1	1	0,000E0	4,929E6		0,00			2,12	1,21	1	1	911	100,6	7,31
P42566	Epidermal growth factor receptor substrate 15 OS=Homo sapiens OX=9606 GN=EPS15 PE=1 SV=2 - [EPS1	1,45	1	1	1	1	0,000E0	0,000E0		0,00			2,12	1,45	1	1	896	98,6	4,64
P63151	Serine/threonine-protein phosphatase 2A 55 kDa regulatory subunit B alpha isoform OS=Homo sapiens OX=9	5,82	3	2	2	2	1,615E6	4,341E6	0,00	3,13	1	1	2,12	2,68	1	1	447	51,7	6,20
P39656	Dolichyl-diphosphooligosaccharide--protein glycosyltransferase 48 kDa subunit OS=Homo sapiens OX=9606	8,33	1	2	2	2	1,639E6	6,748E6	0,00	6,36	1	1	2,12	1,97	1	1	456	50,8	6,55
Q96SY0	Integrator complex subunit 14 OS=Homo sapiens OX=9606 GN=INTS14 PE=1 SV=2 - [INT14_HUMAN]	2,12	1	1	1	1	0,000E0	2,267E6					2,11	2,12	1	1	518	57,4	5,12
O94762	ATP-dependent DNA helicase Q5 OS=Homo sapiens OX=9606 GN=RECQL5 PE=1 SV=2 - [RECQ5_HUM	2,12	1	2	2	2	0,000E0	4,439E6		0,00			2,10	2,12	2	2	991	108,8	8,56
O15260	Surfeit locus protein 4 OS=Homo sapiens OX=9606 GN=SURF4 PE=1 SV=3 - [SURF4_HUMAN]	3,72	1	1	1	1	0,000E0	5,443E6		0,00			2,09	3,72	1	1	269	30,4	7,78
Q99460	26S proteasome non-ATPase regulatory subunit 1 OS=Homo sapiens OX=9606 GN=PSMD1 PE=1 SV=2 - [	1,99	1	1	1	1	0,000E0	0,000E0		0,00			2,09	1,99	1	1	953	105,8	5,39
Q15029	116 kDa U5 small nuclear ribonucleoprotein component OS=Homo sapiens OX=9606 GN=EFTUD2 PE=1 S	3,50	1	2	2	2	1,105E6	1,474E6	2,50	2,16	1	1	2,09	1,34	1	1	972	109,4	5,00
Q9BWT7	Caspase recruitment domain-containing protein 10 OS=Homo sapiens OX=9606 GN=CARD10 PE=1 SV=2 -	1,94	1	1	1	1	0,000E0	1,638E6		0,00			2,09	1,94	1	1	1032	115,9	5,95
Q9UHW5	GPN-loop GTPase 3 OS=Homo sapiens OX=9606 GN=GPN3 PE=1 SV=2 - [GPN3_HUMAN]	4,58	1	1	1	1	0,000E0	1,598E6		0,00			2,09	4,58	1	1	284	32,7	4,50
Q9UFB7	Zinc finger and BTB domain-containing protein 47 OS=Homo sapiens OX=9606 GN=ZBTB47 PE=1 SV=3	3,21	1	1	1	1	0,000E0	1,744E6		0,00			2,08	3,21	1	1	747	82,7	5,76
Q14123	Calcium/calmodulin-dependent 3',5'-cyclic nucleotide phosphodiesterase 1C OS=Homo sapiens OX=9606 GN	1,83	1	1	1	1	0,000E0	2,576E6		0,00			2,08	1,83	1	1	709	80,7	8,82
P68431	Histone H3.1 OS=Homo sapiens OX=9606 GN=HIST1H3A PE=1 SV=2 - [H31_HUMAN]	35,29	3	1	3	4	9,501E6	1,356E7	7,89	30,15	2	3	2,08	5,15	1	1	136	15,4	11,12
Q71DI3	Histone H3.2 OS=Homo sapiens OX=9606 GN=HIST2H3A PE=1 SV=3 - [H32_HUMAN]	35,29	3	1	3	6	5,279E7	1,356E7	15,65	30,15	2	5	2,08	5,15	1	1	136	15,4	11,27
P02787	Serotransferrin OS=Homo sapiens OX=9606 GN=TF PE=1 SV=3 - [TRFE_HUMAN]	3,01	1	2	2	2	3,922E6	7,183E6	0,00	1,58	1	1	2,07	1,43	1	1	698	77,0	7,12
P10074	Telomere zinc finger-associated protein OS=Homo sapiens OX=9606 GN=ZBTB48 PE=1 SV=2 - [TZAP_HU	1,74	1	1	1	1	0,000E0	2,010E7		0,00			2,07	1,74	1	1	688	77,0	8,18
P26368	Splicing factor U2AF 65 kDa subunit OS=Homo sapiens OX=9606 GN=U2AF2 PE=1 SV=4 - [U2AF2_HU	13,26	1	4	4	4	3,922E6	1,762E6	3,78	9,47	2	2	2,06	3,79	2	2	475	53,5	9,09
P48681	Nestin OS=Homo sapiens OX=9606 GN=NES PE=1 SV=2 - [NEST_HUMAN]	1,60	1	2	2	2	0,000E0	1,007E7		0,00			2,05	1,60	2	2	1621	177,3	4,36
P61964	WD repeat-containing protein 5 OS=Homo sapiens OX=9606 GN=WDR5 PE=1 SV=1 - [WDR5_HUMAN]	4,19	1	1	1	1	0,000E0	1,096E6					2,05	4,19	1	1	334	36,6	8,27
Q09161	Nuclear cap-binding protein subunit 1 OS=Homo sapiens OX=9606 GN=NCBP1 PE=1 SV=1 - [NCBP1_HU	1,39	1	1	1	1	0,000E0	3,452E6		0,00			2,04	1,39	1	1	790	91,8	6,43
Q75N03	E3 ubiquitin-protein ligase Hakai OS=Homo sapiens OX=9606 GN=CBLL1 PE=1 SV=1 - [HAKAI_HUMA	4,07	1	1	1	1	0,000E0	1,408E7		0,00			2,04	4,07	1	1	491	54,5	8,29
Q7KZF4	Staphylococcal nuclease domain-containing protein 1 OS=Homo sapiens OX=9606 GN=SND1 PE=1 SV=1 -	2,75	1	2	2	2	0,000E0	1,500E6	0,00	1,43	1	1	2,03	1,32	1	1	910	101,9	7,17
P34932	Heat shock 70 kDa protein 4 OS=Homo sapiens OX=9606 GN=HSPA4 PE=1 SV=4 - [HSP74_HUMAN]	3,93	1	2	2	2	0,000E0	1,633E6		0,00			2,03	3,93	2	2	840	94,3	5,19
P13797	Plastin-3 OS=Homo sapiens OX=9606 GN=PLS3 PE=1 SV=4 - [PLST_HUMAN]	8,73	2	3	5	6	4,412E6	2,542E6	5,29	7,30	4	4	2,03	3,65	2	2	630	70,8	5,60
O95232	Luc7-like protein 3 OS=Homo sapiens OX=9606 GN=LUC7L3 PE=1 SV=2 - [LC7L3_HUMAN]	4,17	1	1	1	1	0,000E0	3,625E6		0,00			2,03	4,17	1	1	432	51,4	9,79
Q9H4M9	EH domain-containing protein 1 OS=Homo sapiens OX=9606 GN=EHD1 PE=1 SV=2 - [EHD1_HUMAN]	4,31	2	1	2	2	7,302E5	6,326E6	2,39	2,62	1	1	2,03	1,69	1	1	534	60,6	6,83
O95248	Myotubularin-related protein 5 OS=Homo sapiens OX=9606 GN=SBF1 PE=1 SV=4 - [MTMR5_HUMAN]	1,12	1	1	1	1	0,000E0	2,239E6		0,00			2,03	1,12	1	1	1868	208,3	6,90
Q8TBC3	SH3KBP1-binding protein 1 OS=Homo sapiens OX=9606 GN=SHKBP1 PE=1 SV=2 - [SHKB1_HUMAN]	2,26	1	1	1	1	0,000E0	0,000E0		0,00			2,03	2,26	1	1	707	76,3	8,28
P63092	Guanine nucleotide-binding protein G(s) subunit alpha isoforms short OS=Homo sapiens OX=9606 GN=GNA	15,74	11	2	3	3	1,224E6	3,284E6	2,57	12,94	2	2	2,02	2,79	1	1	394	45,6	5,82
P13674	Prolyl 4-hydroxylase subunit alpha-1 OS=Homo sapiens OX=9606 GN=P4HA1 PE=1 SV=2 - [P4HA1_HUM	1,87	1	1	1	1	0,000E0	0,000E0		0,00			2,01	1,87	1	1	534	61,0	6,01
P49207	60S ribosomal protein L34 OS=Homo sapiens OX=9606 GN=RPL34 PE=1 SV=3 - [RL34_HUMAN]	5,98	1	1	1	1	0,000E0	2,185E7		0,00			2,01	5,98	1	1	117	13,3	11,47
Q7RTS7	Keratin, type II cytoskeletal 74 OS=Homo sapiens OX=9606 GN=KRT74 PE=1 SV=2 - [K2C74_HUMAN]	6,43	4	2	4	11	5,024E8	3,148E8	13,15	6,43	4	10	2,00	1,32	1	1	529	57,8	7,71
P85037	Forkhead box protein K1 OS=Homo sapiens OX=9606 GN=FOXK1 PE=1 SV=1 - [FOXK1_HUMAN]	3,41	1	3	3	3	0,000E0	1,348E6		0,00			1,99	3,41	3	3	733	75,4	9,32
Q86XJ1	GAS2-like protein 3 OS=Homo sapiens OX=9606 GN=GAS2L3 PE=1 SV=1 - [GA2L3_HUMAN]	1,87	1	1	1	1	0,000E0	2,160E6		0,00			1,98	1,87	1	1	694	75,2	9,63
Q6PCB5	Round spermatid basic protein 1-like protein OS=Homo sapiens OX=9606 GN=RSBN1L PE=1 SV=2 - [RSI	3,19	1	3	3	3	0,000E0	7,380E6		0,00			1,98	3,19	3	3	846	94,8	8,78
Q8N2K0	Monoacylglycerol lipase ABHD12 OS=Homo sapiens OX=9606 GN=ABHD12 PE=1 SV=2 - [ABD12_HUM	2,26	1	1	1	1	0,000E0	0,000E0		0,00			1,97	2,26	1	1	398	45,1	8,65
Q9NZB2	Constitutive coactivator of PPAR-gamma-like protein 1 OS=Homo sapiens OX=9606 GN=FAM120A PE=1	1,16	1	1	1	1	0,000E0	3,912E6		0,00			1,97	1,16	1	1	1118	121,8	8,88
P41212	Transcription factor ETV6 OS=Homo sapiens OX=9606 GN=ETV6 PE=1 SV=1 - [ETV6_HUMAN]	3,76	1	2	2	2	0,000E0	1,334E7		0,00			1,97	3,76	2	2	452	53,0	7,43
Q15286	Ras-related protein Rab-35 OS=Homo sapiens OX=9606 GN=RAB35 PE=1 SV=1 - [RAB35_HUMAN]	19,90	20	1	3	3	9,924E6	8,056E6	4,18	14,43	2	2	1,97	5,47	1	1	201	23,0	8,29
P61026	Ras-related protein Rab-10 OS=Homo sapiens OX=9606 GN=RAB10 PE=1 SV=1 - [RAB10_HUMAN]	16,50	20	1	3	3	5,497E6	8,056E6	1,68	11,00	2	2	1,97	5,50	1	1	200	22,5	8,38
P51153	Ras-related protein Rab-13 OS=Homo sapiens OX=9606 GN=RAB13 PE=1 SV=1 - [RAB13_HUMAN]	10,34	1	1	2	2	0,000E0	4,810E6		0,00			1,97	10,34	2	2	203	22,8	9,19
P01023	Alpha-2-macroglobulin OS=Homo sapiens OX=9606 GN=A2M PE=1 SV=3 - [A2MG_HUMAN]	1,29	2	2	2	2	0,000E0	1,118E8		0,00			1,97	1,29	2	2	1474	163,2	6,46
Q96ST8	Centrosomal protein of 89 kDa OS=Homo sapiens OX=9606 GN=CEP89 PE=1 SV=3 - [CEP89_HUMAN]	2,81	1	2	2	2	0,000E0	3,661E6		0,00			1,97	2,81	2	2	783	89,5	6,80
P60903	Protein S100-A10 OS=Homo sapiens OX=9606 GN=S100A10 PE=1 SV=2 - [S10AA_HUMAN]	10,31	1	1	1	1	0,000E0	1,002E8					1,97	10,31	1	1	97	11,2	7,37
O43175	D-3-phosphoglycerate dehydrogenase OS=Homo sapiens OX=9606 GN=PHGDH PE=1 SV=4 - [SERA_HUM	11,07	1	4	4	4	1,468E6	5,963E6	3,41	9,01	3	3	1,97	2,06	1	1	533	56,6	6,71
Q9BQ67	Glutamate-rich WD repeat-containing protein 1 OS=Homo sapiens OX=9606 GN=GRWD1 PE=1 SV=1 - [GI	2,02	1	1	1	1	0,000E0	0,000E0		0,00			1,96	2,02	1	1	446	49,4	4,92
Q9NW64	Pre-mRNA-splicing factor RBM22 OS=Homo sapiens OX=9606 GN=RBM22 PE=1 SV=1 - [RBM22_HUM	6,43	1	2	2	2	0,000E0	1,638E7		0,00			1,96	6,43	2	2	420	46,9	8,54
Q92621	Nuclear pore complex protein Nup205 OS=Homo sapiens OX=9606 GN=NUP205 PE=1 SV=3 - [NU205_HU	0,45	1	1	1	1	0,000E0	3,056E6		0,00			1,96	0,45	1	1	2012	227,8	6,19
O43296	Zinc finger protein 264 OS=Homo sapiens OX=9606 GN=ZNF264 PE=1 SV=1 - [ZN264_HUMAN]	1,75	1	1	1	1	0,000E0	2,772E6		0,00			1,95	1,75	1	1	627	70,5	7,49
Q9UG63	ATP-binding cassette sub-family F member 2 OS=Homo sapiens OX=9606 GN=ABCF2 PE=1 SV=2 - [ABC	1,44	1	1	1	1	0,000E0	3,292E6		0,00			1,95	1,44	1	1	623	71,2	7,37
Q8NC56	LEM domain-containing protein 2 OS=Homo sapiens OX=9606 GN=LEMD2 PE=1 SV=1 - [LEMD2_HUM	5,96	1	2	2	2	0,000E0	2,842E6		0,00			1,95	5,96	2	2	503	56,9	9,00
P02647	Apolipoprotein A-I OS=Homo sapiens OX=9606 GN=APOA1 PE=1 SV=1 - [APOA1_HUMAN]	5,99	1	1	1	1	0,000E0	1,463E7		0,00			1,95	5,99	1	1	267	30,8	5,76
P00558	Phosphoglycerate kinase 1 OS=Homo sapiens OX=9606 GN=PGK1 PE=1 SV=3 - [PGK1_HUMAN]	28,54	1	9	9	10	1,995E7	3,757E6	12,92	26,14	8	9	1,94	2,40	1	1	417	44,6	8,10
Q9BYK8	Helicase with zinc finger domain 2 OS=Homo sapiens OX=9606 GN=HELZ2 PE=1 SV=6 - [HELZ2_HUMA	0,38	1	1	1	1	0,000E0	0,000E0		0,00			1,94	0,38	1	1	2649	294,5	7,49
O60341	Lysine-specific histone demethylase 1A OS=Homo sapiens OX=9606 GN=KDM1A PE=1 SV=2 - [KDM1A_	1,17	1	1	1	1	0,000E0	1,678E6		0,00			1,94	1,17	1	1	852	92,8	6,52
Q96D71	RalBP1-associated Eps domain-containing protein 1 OS=Homo sapiens OX=9606 GN=REPS1 PE=1 SV=3 -	2,26	2	2	2	2	0,000E0	4,451E6		0,00			1,94	2,26	2	2	796	86,6	5,69
Q96D46	60S ribosomal export protein NMD3 OS=Homo sapiens OX=9606 GN=NMD3 PE=1 SV=1 - [NMD3_HUM	1,59	1	1	1	1	0,000E0	1,604E6		0,00			1,93	1,59	1	1	503	57,6	7,14
Q9H8G1	Zinc finger protein 430 OS=Homo sapiens OX=9606 GN=ZNF430 PE=1 SV=3 - [ZN430_HUMAN]	1,58	2	1	1	1	0,000E0	4,893E6		0,00			1,93	1,58	1	1	570	66,3	8,65
Q6KB66	Keratin, type II cytoskeletal 80 OS=Homo sapiens OX=9606 GN=KRT80 PE=1 SV=2 - [K2C80_HUMAN]	8,41	2	3	4	24	1,104E7	2,493E5	36,13	8,41	4	23	1,93	2,43	1	1	452	50,5	5,67
P46939	Utrophin OS=Homo sapiens OX=9606 GN=UTRN PE=1 SV=2 - [UTRO_HUMAN]	0,73	1	2	2	2	0,000E0	2,830E6		0,00			1,91	0,73	2	2	3433	394,2	5,33
Q9BXP5	Serrate RNA effector molecule homolog OS=Homo sapiens OX=9606 GN=SRRT PE=1 SV=1 - [SRRT_HU	1,48	1	1	1	1	0,000E0	2,080E6		0,00			1,91	1,48	1	1	876	100,6	5,96
Q16576	Histone-binding protein RBBP7 OS=Homo sapiens OX=9606 GN=RBBP7 PE=1 SV=1 - [RBBP7_HUMAN]	11,53	1	2	3	4	2,551E6	9,139E5	2,72	11,53	3	3	1,90	2,82	1	1	425	47,8	5,05
O43166	Signal-induced proliferation-associated 1-like protein 1 OS=Homo sapiens OX=9606 GN=SIPA1L1 PE=1 SV	0,72	1	1	1	1	0,000E0	7,525E5		0,00			1,90	0,72	1	1	1804	199,9	8,19

Q8N1N4	Keratin, type II cytoskeletal 78 OS=Homo sapiens OX=9606 GN=KRT78 PE=1 SV=2 - [K2C78_HUMAN]	18,85	1	8	9	13	4,209E7	1,753E6	11,86	16,54	8	11	1,90	5,19	2	2	520	56,8	6,02
Q10471	Polypeptide N-acetylgalactosaminyltransferase 2 OS=Homo sapiens OX=9606 GN=GALNT2 PE=1 SV=1 - [	1,75	1	1	1	1	0,000E0	2,003E6		0,00			1,89	1,75	1	1	571	64,7	8,35
O95168	NADH dehydrogenase [ubiquinone] 1 beta subcomplex subunit 4 OS=Homo sapiens OX=9606 GN=NDUFB	7,75	1	1	1	1	0,000E0	2,188E6					1,89	7,75	1	1	129	15,2	9,85
P17812	CTP synthase 1 OS=Homo sapiens OX=9606 GN=CTPS1 PE=1 SV=2 - [PYRG1_HUMAN]	5,75	1	3	3	3	3,472E6	8,514E6	1,73	2,71	1	1	1,89	3,05	2	2	591	66,6	6,46
P31947	14-3-3 protein sigma OS=Homo sapiens OX=9606 GN=SFN PE=1 SV=1 - [1433S_HUMAN]	11,69	2	1	3	4	3,722E7	1,852E7	4,31	4,44	1	2	1,89	7,26	2	2	248	27,8	4,74
P49750	YLP motif-containing protein 1 OS=Homo sapiens OX=9606 GN=YLPM1 PE=1 SV=4 - [YLPM1_HUMAN	0,47	1	1	1	1	0,000E0	3,483E6		0,00			1,88	0,47	1	1	2146	241,5	6,55
O60762	Dolichol-phosphate mannosyltransferase subunit 1 OS=Homo sapiens OX=9606 GN=DPM1 PE=1 SV=1 - [	11,54	1	3	3	3	0,000E0	6,827E6		0,00			1,88	11,54	3	3	260	29,6	9,57
Q14669	E3 ubiquitin-protein ligase TRIP12 OS=Homo sapiens OX=9606 GN=TRIP12 PE=1 SV=1 - [TRIPC_HUM	0,85	1	1	1	1	0,000E0	1,325E6		0,00			1,88	0,85	1	1	1992	220,3	8,48
Q9HCJ0	Trinucleotide repeat-containing gene 6C protein OS=Homo sapiens OX=9606 GN=TNRC6C PE=1 SV=3 - [	0,71	2	1	1	1	0,000E0	0,000E0		0,00			1,88	0,71	1	1	1690	175,9	6,96
P06732	Creatine kinase M-type OS=Homo sapiens OX=9606 GN=CKM PE=1 SV=2 - [KCRM_HUMAN]	1,57	1	1	1	1	0,000E0	1,057E7		0,00			1,88	1,57	1	1	381	43,1	7,25
Q12905	Interleukin enhancer-binding factor 2 OS=Homo sapiens OX=9606 GN=ILF2 PE=1 SV=2 - [ILF2_HUMAN]	13,59	1	3	3	4	0,000E0	6,226E6	0,00	5,90	1	1	1,88	13,59	3	3	390	43,0	5,26
Q76M96	Coiled-coil domain-containing protein 80 OS=Homo sapiens OX=9606 GN=CCDC80 PE=1 SV=1 - [CCD8	1,26	1	1	1	1	0,000E0	1,224E6		0,00			1,87	1,26	1	1	950	108,1	9,72
Q9BRR0	Zinc finger protein with KRAB and SCAN domains 3 OS=Homo sapiens OX=9606 GN=ZKSCAN3 PE=1 S	4,46	1	2	2	2	0,000E0	4,353E6		0,00			1,86	4,46	2	2	538	60,6	6,40
P42766	60S ribosomal protein L35 OS=Homo sapiens OX=9606 GN=RPL35 PE=1 SV=2 - [RL35_HUMAN]	13,82	1	2	2	2	0,000E0	1,493E8		0,00			1,86	13,82	2	2	123	14,5	11,05
P25205	DNA replication licensing factor MCM3 OS=Homo sapiens OX=9606 GN=MCM3 PE=1 SV=3 - [MCM3_H	9,90	1	6	6	6	3,597E6	1,208E6	9,84	7,30	4	4	1,86	2,60	2	2	808	90,9	5,77
Q9NYU1	UDP-glucose:glycoprotein glucosyltransferase 2 OS=Homo sapiens OX=9606 GN=UGGT2 PE=1 SV=4 - [U	0,79	1	1	1	1	0,000E0	0,000E0		0,00			1,86	0,79	1	1	1516	174,6	6,89
P09496	Clathrin light chain A OS=Homo sapiens OX=9606 GN=CLTA PE=1 SV=1 - [CLCA_HUMAN]	3,23	1	1	1	1	0,000E0	1,499E6		0,00			1,85	3,23	1	1	248	27,1	4,51
P13073	Cytochrome c oxidase subunit 4 isoform 1, mitochondrial OS=Homo sapiens OX=9606 GN=COX4I1 PE=1	5,92	1	1	1	2	4,451E5	1,275E6	0,00	5,92	1	1	1,85	5,92	1	1	169	19,6	9,51
Q13573	SNW domain-containing protein 1 OS=Homo sapiens OX=9606 GN=SNW1 PE=1 SV=1 - [SNW1_HUMAN	1,87	1	1	1	1	0,000E0	1,429E6		0,00			1,85	1,87	1	1	536	61,5	9,52
Q9H5H4	Zinc finger protein 768 OS=Homo sapiens OX=9606 GN=ZNF768 PE=1 SV=2 - [ZN768_HUMAN]	3,70	1	2	2	2	0,000E0	2,477E6		0,00			1,84	3,70	2	2	540	60,2	7,97
P02771	Alpha-fetoprotein OS=Homo sapiens OX=9606 GN=AFP PE=1 SV=1 - [FETA_HUMAN]	1,64	1	1	1	1	0,000E0	5,286E6		0,00			1,84	1,64	1	1	609	68,6	5,68
Q14684	Ribosomal RNA processing protein 1 homolog B OS=Homo sapiens OX=9606 GN=RRP1B PE=1 SV=3 - [	1,45	1	1	1	1	0,000E0	0,000E0		0,00			1,84	1,45	1	1	758	84,4	9,76
Q08J23	tRNA (cytosine(34)-C(5))-methyltransferase OS=Homo sapiens OX=9606 GN=NSUN2 PE=1 SV=2 - [NSUN	4,56	1	3	3	3	2,559E6	1,975E6	0,00	4,56	2	2	1,83	2,48	1	1	767	86,4	6,77
Q9UBK9	Protein UXT OS=Homo sapiens OX=9606 GN=UXT PE=1 SV=1 - [UXT_HUMAN]	8,28	1	1	1	1	0,000E0	2,198E6		0,00			1,82	8,28	1	1	157	18,2	7,59
P00403	Cytochrome c oxidase subunit 2 OS=Homo sapiens OX=9606 GN=MT-CO2 PE=1 SV=1 - [COX2_HUMAN	4,41	1	1	1	1	0,000E0	1,669E6		0,00			1,82	4,41	1	1	227	25,5	4,82
P07910	Heterogeneous nuclear ribonucleoproteins C1/C2 OS=Homo sapiens OX=9606 GN=HNRNPC PE=1 SV=4 -	6,54	4	2	2	3	6,769E6	7,241E6	2,79	6,54	2	2	1,82	2,94	1	1	306	33,6	5,08
O75290	Zinc finger protein 780A OS=Homo sapiens OX=9606 GN=ZNF780A PE=2 SV=3 - [Z780A_HUMAN]	2,18	1	1	1	1	0,000E0	2,743E6		0,00			1,82	2,18	1	1	641	74,5	8,65
Q96I27	Zinc finger protein 625 OS=Homo sapiens OX=9606 GN=ZNF625 PE=1 SV=1 - [ZN625_HUMAN]	2,94	1	1	1	1	0,000E0	3,272E6		0,00			1,81	2,94	1	1	306	34,7	8,98
Q14686	Nuclear receptor coactivator 6 OS=Homo sapiens OX=9606 GN=NCOA6 PE=1 SV=3 - [NCOA6_HUMAN]	1,21	1	2	2	2	0,000E0	1,348E6		0,00			1,81	1,21	2	2	2063	219,0	9,36
Q71RC2	La-related protein 4 OS=Homo sapiens OX=9606 GN=LARP4 PE=1 SV=3 - [LARP4_HUMAN]	1,80	1	1	1	1	0,000E0	1,990E6		0,00			1,81	1,80	1	1	724	80,5	6,61
Q9NT68	Teneurin-2 OS=Homo sapiens OX=9606 GN=TENM2 PE=1 SV=3 - [TEN2_HUMAN]	0,61	1	1	1	1	0,000E0	1,390E6		0,00			1,80	0,61	1	1	2774	307,6	6,68
P22314	Ubiquitin-like modifier-activating enzyme 1 OS=Homo sapiens OX=9606 GN=UBA1 PE=1 SV=3 - [UBA1_	9,36	1	4	4	4	1,203E7	2,407E6	8,42	7,94	3	3	1,80	1,42	1	1	1058	117,8	5,76
O43663	Protein regulator of cytokinesis 1 OS=Homo sapiens OX=9606 GN=PRC1 PE=1 SV=2 - [PRC1_HUMAN]	1,29	1	1	1	1	0,000E0	1,793E6		0,00			1,80	1,29	1	1	620	71,6	6,68
Q16629	Serine/arginine-rich splicing factor 7 OS=Homo sapiens OX=9606 GN=SRSF7 PE=1 SV=1 - [SRSF7_HUM	17,65	1	3	4	4	1,035E7	4,697E7	3,11	13,87	2	2	1,79	9,66	2	2	238	27,4	11,82
Q9NZJ7	Mitochondrial carrier homolog 1 OS=Homo sapiens OX=9606 GN=MTCH1 PE=1 SV=1 - [MTCH1_HUMA	5,40	1	2	2	2	0,000E0	0,000E0		0,00			1,79	5,40	2	2	389	41,5	9,32
P61326	Protein mago nashi homolog OS=Homo sapiens OX=9606 GN=MAGOH PE=1 SV=1 - [MGN_HUMAN]	6,16	2	1	1	1	0,000E0	1,037E7		0,00			1,79	6,16	1	1	146	17,2	6,11
P15622	Zinc finger protein 250 OS=Homo sapiens OX=9606 GN=ZNF250 PE=1 SV=3 - [ZN250_HUMAN]	1,61	1	1	1	1	0,000E0	3,525E6		0,00			1,79	1,61	1	1	560	63,4	8,28
Q5VZL5	Zinc finger MYM-type protein 4 OS=Homo sapiens OX=9606 GN=ZMYM4 PE=1 SV=1 - [ZMYM4_HUM	0,84	1	1	1	1	0,000E0	2,625E6		0,00			1,79	0,84	1	1	1548	172,7	6,84
Q96L58	Beta-1,3-galactosyltransferase 6 OS=Homo sapiens OX=9606 GN=B3GALT6 PE=1 SV=2 - [B3GT6_HUMA	2,74	1	1	1	1	0,000E0	7,145E5		0,00			1,78	2,74	1	1	329	37,1	9,66
P83111	Serine beta-lactamase-like protein LACTB, mitochondrial OS=Homo sapiens OX=9606 GN=LACTB PE=1 S	1,28	1	1	1	1	0,000E0	3,690E6		0,00			1,78	1,28	1	1	547	60,7	8,53
P22695	Cytochrome b-c1 complex subunit 2, mitochondrial OS=Homo sapiens OX=9606 GN=UQCRC2 PE=1 SV=	11,04	1	3	3	4	1,664E6	5,128E6	4,26	4,42	1	1	1,76	6,62	2	3	453	48,4	8,63
P62491	Ras-related protein Rab-11A OS=Homo sapiens OX=9606 GN=RAB11A PE=1 SV=3 - [RB11A_HUMAN]	11,11	2	2	2	2	3,012E6	2,637E6	0,00	6,02	1	1	1,76	5,09	1	1	216	24,4	6,57
Q15758	Neutral amino acid transporter B(0) OS=Homo sapiens OX=9606 GN=SLC1A5 PE=1 SV=2 - [AAAT_HUM	3,70	1	1	1	1	0,000E0	3,554E6		0,00			1,76	3,70	1	1	541	56,6	5,48
Q13724	Mannosyl-oligosaccharide glucosidase OS=Homo sapiens OX=9606 GN=MOGS PE=1 SV=5 - [MOGS_HU	1,19	1	1	1	1	0,000E0	4,240E6		0,00			1,76	1,19	1	1	837	91,9	8,90
P09382	Galectin-1 OS=Homo sapiens OX=9606 GN=LGALS1 PE=1 SV=2 - [LEG1_HUMAN]	11,85	1	2	2	2	0,000E0	5,877E6					1,76	11,85	2	2	135	14,7	5,50
Q9GZS1	DNA-directed RNA polymerase I subunit RPA49 OS=Homo sapiens OX=9606 GN=POLR1E PE=1 SV=2 -	4,37	1	1	1	1	0,000E0	2,367E6		0,00			1,75	4,37	1	1	481	53,9	8,56
Q93008	Probable ubiquitin carboxyl-terminal hydrolase FAF-X OS=Homo sapiens OX=9606 GN=USP9X PE=1 SV=	1,75	7	3	4	4	1,752E6	3,151E6	4,45	0,82	1	1	1,75	0,93	3	3	2570	292,1	5,80
Q9NP64	Nucleolar protein of 40 kDa OS=Homo sapiens OX=9606 GN=ZCCHC17 PE=1 SV=1 - [NO40_HUMAN]	4,56	1	1	1	1	0,000E0	2,447E7		0,00			1,75	4,56	1	1	241	27,6	9,70
P12532	Creatine kinase U-type, mitochondrial OS=Homo sapiens OX=9606 GN=CKMT1A PE=1 SV=1 - [KCRU_H	4,32	3	1	2	2	3,499E6	3,109E6	0,00	2,88	1	1	1,74	1,44	1	1	417	47,0	8,34
O15061	Synemin OS=Homo sapiens OX=9606 GN=SYNM PE=1 SV=2 - [SYNEM_HUMAN]	1,41	1	2	2	2	0,000E0	2,547E6		0,00			1,74	1,41	2	2	1565	172,7	5,16
P19525	Interferon-induced, double-stranded RNA-activated protein kinase OS=Homo sapiens OX=9606 GN=EIF2AK	1,81	1	1	1	1	0,000E0	1,506E6		0,00			1,73	1,81	1	1	551	62,1	8,40
P21589	5'-nucleotidase OS=Homo sapiens OX=9606 GN=NT5E PE=1 SV=1 - [5NTD_HUMAN]	4,53	1	2	2	2	0,000E0	1,588E6		0,00			1,73	4,53	2	2	574	63,3	7,03
P49747	Cartilage oligomeric matrix protein OS=Homo sapiens OX=9606 GN=COMP PE=1 SV=2 - [COMP_HUMA	1,32	2	1	1	1	0,000E0	5,317E6		0,00			1,72	1,32	1	1	757	82,8	4,60
P52736	Zinc finger protein 133 OS=Homo sapiens OX=9606 GN=ZNF133 PE=1 SV=2 - [ZN133_HUMAN]	1,22	1	1	1	1	0,000E0	1,746E6		0,00			1,72	1,22	1	1	654	73,3	8,97
Q6UXN9	WD repeat-containing protein 82 OS=Homo sapiens OX=9606 GN=WDR82 PE=1 SV=1 - [WDR82_HUMA	2,24	1	1	1	1	0,000E0	2,325E6		0,00			1,72	2,24	1	1	313	35,1	7,69
O14602	Eukaryotic translation initiation factor 1A, Y-chromosomal OS=Homo sapiens OX=9606 GN=EIF1AY PE=1	7,64	2	1	1	1	0,000E0	1,448E6		0,00			1,72	7,64	1	1	144	16,4	5,24
A0FGR8	Extended synaptotagmin-2 OS=Homo sapiens OX=9606 GN=ESYT2 PE=1 SV=1 - [ESYT2_HUMAN]	1,41	1	1	1	1	0,000E0	2,647E6		0,00			1,70	1,41	1	1	921	102,3	9,26
Q13151	Heterogeneous nuclear ribonucleoprotein A0 OS=Homo sapiens OX=9606 GN=HNRNPA0 PE=1 SV=1 - [R	9,18	1	1	3	4	6,096E6	3,538E7	3,47	9,18	3	3	1,70	2,30	1	1	305	30,8	9,29
O60264	SWI/SNF-related matrix-associated actin-dependent regulator of chromatin subfamily A member 5 OS=Homo	2,76	2	3	3	3	0,000E0	3,257E6		0,00			1,68	2,76	3	3	1052	121,8	8,09
Q9Y2R9	28S ribosomal protein S7, mitochondrial OS=Homo sapiens OX=9606 GN=MRPS7 PE=1 SV=2 - [RT07_H	4,96	1	1	1	1	0,000E0	1,915E6		0,00			1,68	4,96	1	1	242	28,1	9,99
Q9Y3F4	Serine-threonine kinase receptor-associated protein OS=Homo sapiens OX=9606 GN=STRAP PE=1 SV=1 - [	5,14	1	1	1	1	0,000E0	0,000E0		0,00			1,68	5,14	1	1	350	38,4	5,12
Q5SSJ5	Heterochromatin protein 1-binding protein 3 OS=Homo sapiens OX=9606 GN=HP1BP3 PE=1 SV=1 - [HP1	6,87	1	2	2	2	5,811E6	2,496E6	4,26	4,88	1	1	1,68	1,99	1	1	553	61,2	9,67
Q14847	LIM and SH3 domain protein 1 OS=Homo sapiens OX=9606 GN=LASP1 PE=1 SV=2 - [LASP1_HUMAN]	5,36	1	1	1	1	0,000E0	2,715E6		0,00			1,68	5,36	1	1	261	29,7	7,05
O15145	Actin-related protein 2/3 complex subunit 3 OS=Homo sapiens OX=9606 GN=ARPC3 PE=1 SV=3 - [ARPC	13,48	1	2	2	2	8,714E5	2,267E7	0,00	6,18	1	1	1,67	7,30	1	1	178	20,5	8,59
Q9Y6C9	Mitochondrial carrier homolog 2 OS=Homo sapiens OX=9606 GN=MTCH2 PE=1 SV=1 - [MTCH2_HUMA	2,31	1	1	1	1	0,000E0	2,193E6		0,00			1,67	2,31	1	1	303	33,3	7,97
P62910	60S ribosomal protein L32 OS=Homo sapiens OX=9606 GN=RPL32 PE=1 SV=2 - [RL32_HUMAN]	13,33	1	2	2	2	8,030E5	5,654E6	2,08	7,41	1	1	1,67	5,93	1	1	135	15,8	11,33
O75475	PC4 and SFRS1-interacting protein OS=Homo sapiens OX=9606 GN=PSIP1 PE=1 SV=1 - [PSIP1_HUMA	3,40	1	1	1	1	0,000E0	1,507E6		0,00			1,67	3,40	1	1	530	60,1	9,13
O00483	Cytochrome c oxidase subunit NDUFA4 OS=Homo sapiens OX=9606 GN=NDUFA4 PE=1 SV=1 - [NDUA	12,35	1	1	1	1	0,000E0	9,487E6		0,00			1,66	12,35	1	1	81	9,4	9,38
Q8TED1	Probable glutathione peroxidase 8 OS=Homo sapiens OX=9606 GN=GPX8 PE=1 SV=2 - [GPX8_HUMAN]	10,53	1	2	2	2	0,000E0	5,264E6		0,00			1,66	10,53	2	2	209	23,9	9,35
O75147	Obscurin-like protein 1 OS=Homo sapiens OX=9606 GN=OBSL1 PE=1 SV=4 - [OBSL1_HUMAN]	0,74	1	1	1	1	0,000E0	9,240E7		0,00			1,65	0,74	1	1	1896	206,8	5,63
O60701	UDP-glucose 6-dehydrogenase OS=Homo sapiens OX=9606 GN=UGDH PE=1 SV=1 - [UGDH_HUMAN]	11,94	1	4	4	6	2,247E7	8,297E5	11,11	11,94	4	5	1,65	2,63	1	1	494	55,0	7,12
Q6ZNH5	Zinc finger protein 497 OS=Homo sapiens OX=9606 GN=ZNF497 PE=1 SV=2 - [ZN497_HUMAN]	2,61	1	1	1	1	0,000E0	1,274E7		0,00			1,65	2,61	1	1	498	54,7	8,94
Q16514	Transcription initiation factor TFIID subunit 12 OS=Homo sapiens OX=9606 GN=TAF12 PE=1 SV=1 - [TA	6,83	1	1	1	1	0,000E0	7,460E6					1,64	6,83	1	1	161	17,9	7,99
P49368	T-complex protein 1 subunit gamma OS=Homo sapiens OX=9606 GN=CCT3 PE=1 SV=4 - [TCPG_HUMA	11,56	1	3	3	3	6,991E6	3,622E6	6,68	9,54	2	2	1,64	2,02	1	1	545	60,5	6,49
P41250	Glycine--tRNA ligase OS=Homo sapiens OX=9606 GN=GARS PE=1 SV=3 - [GARS_HUMAN]	1,35	1	1	1	1	0,000E0	0,000E0		0,00			1,64	1,35	1	1	739	83,1	7,03
P00973	2'-5'-oligoadenylate synthase 1 OS=Homo sapiens OX=9606 GN=OAS1 PE=1 SV=4 - [OAS1_HUMAN]	1,75	1	1	1	1	0,000E0	1,133E6		0,00			1,64	1,75	1	1	400	46,0	8,22
P48047	ATP synthase subunit O, mitochondrial OS=Homo sapiens OX=9606 GN=ATP5O PE=1 SV=1 - [ATPO_HU	19,25	1	3	3	3	8,417E5	8,055E5	1,82	7,98	1	1	1,63	11,27	2	2	213	23,3	9,96
P61218	DNA-directed RNA polymerases I, II, and III subunit RPABC2 OS=Homo sapiens OX=9606 GN=POLR2F	6,30	1	1	1	1	0,000E0	1,323E7					1,63	6,30	1	1	127	14,5	4,22
P32969	60S ribosomal protein L9 OS=Homo sapiens OX=9606 GN=RPL9 PE=1 SV=1 - [RL9_HUMAN]	29,17	1	3	3	4	3,671E6	3,255E7	5,24	29,17	3	3	1,63	5,21	1	1	192	21,8	9,95
P15924	Desmoplakin OS=Homo sapiens OX=9606 GN=DSP PE=1 SV=3 - [DESP_HUMAN]	17,14	1	50	51	56	5,317E7	2,471E6	80,35	17,14	51	54	1,62	0,98	2	2	2871	331,6	6,81
Q9NS86	LanC-like protein 2 OS=Homo sapiens OX=9606 GN=LANCL2 PE=1 SV=1 - [LANC2_HUMAN]	2,22	1	1	1	1	0,000E0	1,680E6		0,00			1,62	2,22	1	1	450	50,8	7,43
P53680	AP-2 complex subunit sigma OS=Homo sapiens OX=9606 GN=AP2S1 PE=1 SV=2 - [AP2S1_HUMAN]	4,93	1	1	1	1	0,000E0	1,436E7		0,00			1,62	4,93	1	1	142	17,0	6,18
O95684	FGFR1 oncogene partner OS=Homo sapiens OX=9606 GN=FGFR1OP PE=1 SV=1 - [FR1OP_HUMAN]	4,51	1	1	1	1	0,000E0	3,272E6		0,00			1,61	4,51	1	1	399	43,0	4,81
Q15646	2'-5'-oligoadenylate synthase-like protein OS=Homo sapiens OX=9606 GN=OASL PE=1 SV=2 - [OASL_HU	1,95	1	1	1	1	0,000E0	0,000E0		0,00			1,61	1,95	1	1	514	59,2	7,87
O75044	SLIT-ROBO Rho GTPase-activating protein 2 OS=Homo sapiens OX=9606 GN=SRGAP2 PE=1 SV=3 - [S	1,12	2	1	1	1	0,000E0	1,273E6		0,00			1,61	1,12	1	1	1071	120,8	6,70
P84090	Enhancer of rudimentary homolog OS=Homo sapiens OX=9606 GN=ERH PE=1 SV=1 - [ERH_HUMAN]	5,77	1	1	1	1	0,000E0	3,050E6		0,00			1,60	5,77	1	1	104	12,3	5,92
Q9UJF2	Ras GTPase-activating protein nGAP OS=Homo sapiens OX=9606 GN=RASAL2 PE=1 SV=2 - [NGAP_HU	0,88	1	1	1	1	0,000E0	0,000E0		0,00			1,60	0,88	1	1	1139	128,5	8,24
Q9NX00	Transmembrane protein 160 OS=Homo sapiens OX=9606 GN=TMEM160 PE=1 SV=1 - [TM160_HUMAN]	3,72	1	1	1	1	0,000E0	1,457E8					0,00	3,72	1	1	188	19,6	8,03
P62280	40S ribosomal protein S11 OS=Homo sapiens OX=9606 GN=RPS11 PE=1 SV=3 - [RS11_HUMAN]	10,76	1	2	2	2	0,000E0	9,164E7		0,00			0,00	10,76	2	2	158	18,4	10,30
Q9NRM1	Enamelin OS=Homo sapiens OX=9606 GN=ENAM PE=1 SV=3 - [ENAM_HUMAN]	0,96	1	1	1	1	0,000E0	7,851E7		0,00			0,00	0,96	1	1	1142	128,7	6,83
Q02543	60S ribosomal protein L18a OS=Homo sapiens OX=9606 GN=RPL18A PE=1 SV=2 - [RL18A_HUMAN]	5,68	1	1	1	1	0,000E0	3,120E7		0,00			0,00	5,68	1	1	176	20,7	10,71
Q96AG4	Leucine-rich repeat-containing protein 59 OS=Homo sapiens OX=9606 GN=LRRC59 PE=1 SV=1 - [LRC59	10,75	1	3	3	3	6,144E5	2,974E7	0,00	3,26	1	1	0,00	7,49	2	2	307	34,9	9,57
Q96EG3	Zinc finger protein 837 OS=Homo sapiens OX=9606 GN=ZNF837 PE=1 SV=2 - [ZN837_HUMAN]	2,82	1	1	1	1	0,000E0	2,247E7		0,00			0,00	2,82	1	1	531	58,0	9,25
P19823	Inter-alpha-trypsin inhibitor heavy chain H2 OS=Homo sapiens OX=9606 GN=ITIH2 PE=1 SV=2 - [ITIH2_H	0,74	1	1	1	1	0,000E0	1,666E7		0,00			0,00	0,74	1	1	946	106,4	6,86
P26641	Elongation factor 1-gamma OS=Homo sapiens OX=9606 GN=EEF1G PE=1 SV=3 - [EF1G_HUMAN]	8,47	1	3	3	3	1,584E7	1,591E7	1,65	6,41	2	2	0,00	2,06	1	1	437	50,1	6,67
P25398	40S ribosomal protein S12 OS=Homo sapiens OX=9606 GN=RPS12 PE=1 SV=3 - [RS12_HUMAN]	7,58	1	1	1	1	0,000E0	1,262E7		0,00			0,00	7,58	1	1	132	14,5	7,21
Q8IXB1	DnaJ homolog subfamily C member 10 OS=Homo sapiens OX=9606 GN=DNAJC10 PE=1 SV=2 - [DJC10	4,67	1	2	2	2	0,000E0	1,239E7		0,00			0,00	4,67	2	2	793	91,0	7,18
P62888	60S ribosomal protein L30 OS=Homo sapiens OX=9606 GN=RPL30 PE=1 SV=2 - [RL30_HUMAN]	5,22	2	1	1	1	0,000E0	8,761E6		0,00			0,00	5,22	1	1	115	12,8	9,63
P37802	Transgelin-2 OS=Homo sapiens OX=9606 GN=TAGLN2 PE=1 SV=3 - [TAGL2_HUMAN]	24,12	1	3	3	3	2,351E6	7,874E6	4,37	15,08	2	2	0,00	9,05	1	1	199	22,4	8,25
Q86Y25	Zinc finger protein 354C OS=Homo sapiens OX=9606 GN=ZNF354C PE=1 SV=1 - [Z354C_HUMAN]	4,51	1	2	2	2	0,000E0	7,849E6		0,00			0,00	4,51	2	2	554	64,8	8,05
P52434	DNA-directed RNA polymerases I, II, and III subunit RPABC3 OS=Homo sapiens OX=9606 GN=POLR2H	4,00	1	1	1	1	0,000E0	6,377E6		0,00			0,00	4,00	1	1	150	17,1	4,68
P43307	Translocon-associated protein subunit alpha OS=Homo sapiens OX=9606 GN=SSR1 PE=1 SV=3 - [SSRA_	13,29	1	2	2	3	9,366E6	6,373E6	2,27	13,29	2	2	0,00	5,24	1	1	286	32,2	4,49
Q9NQZ8	Endothelial zinc finger protein induced by tumor necrosis factor alpha OS=Homo sapiens OX=9606 GN=ZNF	2,66	1	1	1	1	0,000E0	6,111E6		0,00			0,00	2,66	1	1	489	54,5	8,68
Q9C0F3	Zinc finger protein 436 OS=Homo sapiens OX=9606 GN=ZNF436 PE=1 SV=2 - [ZN436_HUMAN]	2,34	1	1	1	1	0,000E0	5,821E6		0,00			0,00	2,34	1	1	470	54,2	7,18
Q53H12	Acylglycerol kinase, mitochondrial OS=Homo sapiens OX=9606 GN=AGK PE=1 SV=2 - [AGK_HUMAN]	3,08	1	1	1	1	0,000E0	5,779E6		0,00			0,00	3,08	1	1	422	47,1	8,09
P19484	Transcription factor EB OS=Homo sapiens OX=9606 GN=TFEB PE=1 SV=3 - [TFEB_HUMAN]	2,10	1	1	1	1	0,000E0	5,777E6		0,00			0,00	2,10	1	1	476	52,8	6,29
O00505	Importin subunit alpha-4 OS=Homo sapiens OX=9606 GN=KPNA3 PE=1 SV=2 - [IMA4_HUMAN]	5,18	1	2	2	2	8,188E5	5,745E6	0,00	2,50	1	1	0,00	2,69	1	1	521	57,8	4,94
P0C0L4	Complement C4-A OS=Homo sapiens OX=9606 GN=C4A PE=1 SV=2 - [CO4A_HUMAN]	1,61	2	2	2	2	0,000E0	5,723E6		0,00			0,00	1,61	2	2	1744	192,7	7,08
Q9NPL8	Complex I assembly factor TIMMDC1, mitochondrial OS=Homo sapiens OX=9606 GN=TIMMDC1 PE=1 S	3,51	1	1	1	1	0,000E0	5,664E6		0,00			0,00	3,51	1	1	285	32,2	8,50
O94813	Slit homolog 2 protein OS=Homo sapiens OX=9606 GN=SLIT2 PE=1 SV=1 - [SLIT2_HUMAN]	0,85	1	1	1	1	0,000E0	5,357E6		0,00			0,00	0,85	1	1	1529	169,8	7,06
Q00587	Cdc42 effector protein 1 OS=Homo sapiens OX=9606 GN=CDC42EP1 PE=1 SV=1 - [BORG5_HUMAN]	3,58	1	1	1	1	0,000E0	4,750E6		0,00			0,00	3,58	1	1	391	40,3	7,15
P28482	Mitogen-activated protein kinase 1 OS=Homo sapiens OX=9606 GN=MAPK1 PE=1 SV=3 - [MK01_HUMA	2,50	1	1	1	1	0,000E0	4,610E6		0,00			0,00	2,50	1	1	360	41,4	6,98
Q53EZ4	Centrosomal protein of 55 kDa OS=Homo sapiens OX=9606 GN=CEP55 PE=1 SV=3 - [CEP55_HUMAN]	1,29	1	1	1	1	0,000E0	4,535E6		0,00			0,00	1,29	1	1	464	54,1	7,01
Q12901	Zinc finger protein 155 OS=Homo sapiens OX=9606 GN=ZNF155 PE=1 SV=4 - [ZN155_HUMAN]	2,42	3	1	1	1	0,000E0	4,251E6		0,00			0,00	2,42	1	1	538	62,0	8,46
P07996	Thrombospondin-1 OS=Homo sapiens OX=9606 GN=THBS1 PE=1 SV=2 - [TSP1_HUMAN]	1,20	2	1	1	1	0,000E0	4,239E6		0,00			0,00	1,20	1	1	1170	129,3	4,94
Q9Y3C6	Peptidyl-prolyl cis-trans isomerase-like 1 OS=Homo sapiens OX=9606 GN=PPIL1 PE=1 SV=1 - [PPIL1_HU	10,24	1	1	1	1	0,000E0	4,165E6					0,00	10,24	1	1	166	18,2	7,99
P04844	Dolichyl-diphosphooligosaccharide--protein glycosyltransferase subunit 2 OS=Homo sapiens OX=9606 GN=R	10,46	1	4	4	4	2,925E6	4,012E6	7,27	9,19	3	3	0,00	1,27	1	1	631	69,2	5,69
P26006	Integrin alpha-3 OS=Homo sapiens OX=9606 GN=ITGA3 PE=1 SV=5 - [ITA3_HUMAN]	0,95	1	1	1	1	0,000E0	3,991E6		0,00			0,00	0,95	1	1	1051	116,5	6,77
Q9NSV4	Protein diaphanous homolog 3 OS=Homo sapiens OX=9606 GN=DIAPH3 PE=1 SV=4 - [DIAP3_HUMAN]	1,17	1	1	1	1	0,000E0	3,834E6		0,00			0,00	1,17	1	1	1193	136,8	7,03
Q96JX3	Protein SERAC1 OS=Homo sapiens OX=9606 GN=SERAC1 PE=1 SV=1 - [SRAC1_HUMAN]	3,82	1	1	1	1	0,000E0	3,827E6		0,00			0,00	3,82	1	1	654	74,1	7,68
Q8N1F7	Nuclear pore complex protein Nup93 OS=Homo sapiens OX=9606 GN=NUP93 PE=1 SV=2 - [NUP93_HUM	1,10	1	1	1	1	0,000E0	3,804E6		0,00			0,00	1,10	1	1	819	93,4	5,72
Q9H4G4	Golgi-associated plant pathogenesis-related protein 1 OS=Homo sapiens OX=9606 GN=GLIPR2 PE=1 SV=3	8,44	1	1	1	1	0,000E0	3,697E6		0,00			0,00	8,44	1	1	154	17,2	9,41
P50552	Vasodilator-stimulated phosphoprotein OS=Homo sapiens OX=9606 GN=VASP PE=1 SV=3 - [VASP_HUM	3,42	1	1	1	1	0,000E0	3,652E6		0,00			0,00	3,42	1	1	380	39,8	8,94
Q8N9F8	Zinc finger protein 454 OS=Homo sapiens OX=9606 GN=ZNF454 PE=2 SV=2 - [ZN454_HUMAN]	2,49	1	1	1	1	0,000E0	3,636E6		0,00			0,00	2,49	1	1	522	60,0	8,65
P36954	DNA-directed RNA polymerase II subunit RPB9 OS=Homo sapiens OX=9606 GN=POLR2I PE=1 SV=1 - [	18,40	1	1	1	1	0,000E0	3,483E6		0,00			0,00	18,40	1	1	125	14,5	5,14
O43823	A-kinase anchor protein 8 OS=Homo sapiens OX=9606 GN=AKAP8 PE=1 SV=1 - [AKAP8_HUMAN]	5,92	1	2	2	2	2,741E6	3,453E6	4,57	3,03	1	1	0,00	2,89	1	1	692	76,1	5,15
Q96RN5	Mediator of RNA polymerase II transcription subunit 15 OS=Homo sapiens OX=9606 GN=MED15 PE=1 SV	2,28	1	1	1	1	0,000E0	3,449E6					0,00	2,28	1	1	788	86,7	9,42
P05109	Protein S100-A8 OS=Homo sapiens OX=9606 GN=S100A8 PE=1 SV=1 - [S10A8_HUMAN]	27,96	1	3	3	6	1,354E8	3,404E6	9,31	27,96	3	5	0,00	11,83	1	1	93	10,8	7,03
Q6AW86	Zinc finger protein 324B OS=Homo sapiens OX=9606 GN=ZNF324B PE=1 SV=1 - [Z324B_HUMAN]	4,04	2	1	1	1	0,000E0	3,373E6		0,00			0,00	4,04	1	1	544	60,6	9,69
Q9Y5S2	Serine/threonine-protein kinase MRCK beta OS=Homo sapiens OX=9606 GN=CDC42BPB PE=1 SV=2 - [M	0,58	2	1	1	1	0,000E0	3,346E6		0,00			0,00	0,58	1	1	1711	194,2	6,37
P20930	Filaggrin OS=Homo sapiens OX=9606 GN=FLG PE=1 SV=3 - [FILA_HUMAN]	2,54	1	8	8	9	9,509E7	3,251E6	20,94	2,54	8	8	0,00	0,39	1	1	4061	434,9	9,25
Q66PJ3	ADP-ribosylation factor-like protein 6-interacting protein 4 OS=Homo sapiens OX=9606 GN=ARL6IP4 PE=	3,80	1	1	1	1	0,000E0	3,194E6		0,00			0,00	3,80	1	1	421	44,9	10,93
P11233	Ras-related protein Ral-A OS=Homo sapiens OX=9606 GN=RALA PE=1 SV=1 - [RALA_HUMAN]	6,31	1	1	1	1	0,000E0	3,084E6		0,00			0,00	6,31	1	1	206	23,6	7,11
Q14145	Kelch-like ECH-associated protein 1 OS=Homo sapiens OX=9606 GN=KEAP1 PE=1 SV=2 - [KEAP1_HUM	2,08	1	1	1	1	0,000E0	3,022E6		0,00			0,00	2,08	1	1	624	69,6	6,44
O75533	Splicing factor 3B subunit 1 OS=Homo sapiens OX=9606 GN=SF3B1 PE=1 SV=3 - [SF3B1_HUMAN]	4,29	1	3	3	3	3,299E6	3,015E6	6,42	3,22	2	2	0,00	1,07	1	1	1304	145,7	7,09
Q8NEZ4	Histone-lysine N-methyltransferase 2C OS=Homo sapiens OX=9606 GN=KMT2C PE=1 SV=3 - [KMT2C_H	0,65	1	2	2	2	0,000E0	2,915E6		0,00			0,00	0,65	2	2	4911	541,0	6,49
O75808	Calpain-15 OS=Homo sapiens OX=9606 GN=CAPN15 PE=1 SV=1 - [CAN15_HUMAN]	1,29	1	1	1	1	0,000E0	2,877E6		0,00			0,00	1,29	1	1	1086	117,2	6,71
Q8NA42	Zinc finger protein 383 OS=Homo sapiens OX=9606 GN=ZNF383 PE=2 SV=1 - [ZN383_HUMAN]	5,47	1	2	2	2	0,000E0	2,815E6		0,00			0,00	5,47	2	2	475	54,6	7,90
Q9BZK7	F-box-like/WD repeat-containing protein TBL1XR1 OS=Homo sapiens OX=9606 GN=TBL1XR1 PE=1 SV=	2,53	3	1	1	1	0,000E0	2,762E6		0,00			0,00	2,53	1	1	514	55,6	5,55
O60716	Catenin delta-1 OS=Homo sapiens OX=9606 GN=CTNND1 PE=1 SV=1 - [CTND1_HUMAN]	1,34	1	1	1	1	0,000E0	2,686E6		0,00			0,00	1,34	1	1	968	108,1	6,23
Q15648	Mediator of RNA polymerase II transcription subunit 1 OS=Homo sapiens OX=9606 GN=MED1 PE=1 SV=	0,76	1	1	1	1	0,000E0	2,662E6		0,00			0,00	0,76	1	1	1581	168,4	8,73
Q9NZ01	Very-long-chain enoyl-CoA reductase OS=Homo sapiens OX=9606 GN=TECR PE=1 SV=1 - [TECR_HUMA	5,84	1	2	2	2	0,000E0	2,658E6		0,00			0,00	5,84	2	2	308	36,0	9,45
Q9NP72	Ras-related protein Rab-18 OS=Homo sapiens OX=9606 GN=RAB18 PE=1 SV=1 - [RAB18_HUMAN]	7,28	1	1	1	1	0,000E0	2,634E6		0,00			0,00	7,28	1	1	206	23,0	5,24
Q9NP81	Serine--tRNA ligase, mitochondrial OS=Homo sapiens OX=9606 GN=SARS2 PE=1 SV=1 - [SYSM_HUMA	3,09	1	1	1	1	0,000E0	2,626E6		0,00			0,00	3,09	1	1	518	58,2	8,13
P13796	Plastin-2 OS=Homo sapiens OX=9606 GN=LCP1 PE=1 SV=6 - [PLSL_HUMAN]	12,12	1	2	4	4	5,591E6	2,542E6	6,49	10,69	3	3	0,00	1,44	1	1	627	70,2	5,43
Q9H0U3	Magnesium transporter protein 1 OS=Homo sapiens OX=9606 GN=MAGT1 PE=1 SV=1 - [MAGT1_HUMA	2,99	1	1	1	1	0,000E0	2,516E6		0,00			0,00	2,99	1	1	335	38,0	9,63
P14866	Heterogeneous nuclear ribonucleoprotein L OS=Homo sapiens OX=9606 GN=HNRNPL PE=1 SV=2 - [HNR	7,64	1	2	2	4	2,130E7	2,375E6	10,24	7,64	2	3	0,00	1,70	1	1	589	64,1	8,22
O15042	U2 snRNP-associated SURP motif-containing protein OS=Homo sapiens OX=9606 GN=U2SURP PE=1 SV	2,62	1	2	2	2	2,762E6	2,330E6	2,93	1,46	1	1	0,00	1,17	1	1	1029	118,2	8,47
Q08554	Desmocollin-1 OS=Homo sapiens OX=9606 GN=DSC1 PE=1 SV=2 - [DSC1_HUMAN]	6,94	1	5	5	7	4,724E7	2,212E6	12,49	5,82	4	5	0,00	1,12	1	2	894	99,9	5,43
Q9Y467	Sal-like protein 2 OS=Homo sapiens OX=9606 GN=SALL2 PE=1 SV=4 - [SALL2_HUMAN]	1,19	1	1	1	1	0,000E0	2,171E6		0,00			0,00	1,19	1	1	1007	105,2	6,30
Q8TBZ8	Zinc finger protein 564 OS=Homo sapiens OX=9606 GN=ZNF564 PE=1 SV=1 - [ZN564_HUMAN]	5,61	1	2	2	2	0,000E0	2,166E6		0,00			0,00	5,61	2	2	553	63,7	8,68
Q96I25	Splicing factor 45 OS=Homo sapiens OX=9606 GN=RBM17 PE=1 SV=1 - [SPF45_HUMAN]	2,74	1	1	1	1	0,000E0	2,160E6		0,00			0,00	2,74	1	1	401	44,9	5,97
Q499Z4	Zinc finger protein 672 OS=Homo sapiens OX=9606 GN=ZNF672 PE=2 SV=2 - [ZN672_HUMAN]	5,53	1	2	2	2	0,000E0	2,159E6		0,00			0,00	5,53	2	2	452	50,2	9,89
Q8IWU5	Extracellular sulfatase Sulf-2 OS=Homo sapiens OX=9606 GN=SULF2 PE=1 SV=1 - [SULF2_HUMAN]	1,49	1	1	1	1	0,000E0	2,158E6		0,00			0,00	1,49	1	1	870	100,4	9,17
O43854	EGF-like repeat and discoidin I-like domain-containing protein 3 OS=Homo sapiens OX=9606 GN=EDIL3 P	2,50	1	1	1	1	0,000E0	2,152E6		0,00			0,00	2,50	1	1	480	53,7	7,28
Q8IUR7	Armadillo repeat-containing protein 8 OS=Homo sapiens OX=9606 GN=ARMC8 PE=1 SV=2 - [ARMC8_H	1,63	1	1	1	1	0,000E0	2,149E6		0,00			0,00	1,63	1	1	673	75,5	6,73
O95985	DNA topoisomerase 3-beta-1 OS=Homo sapiens OX=9606 GN=TOP3B PE=1 SV=1 - [TOP3B_HUMAN]	1,16	1	1	1	1	0,000E0	2,139E6		0,00			0,00	1,16	1	1	862	96,6	8,12
P04075	Fructose-bisphosphate aldolase A OS=Homo sapiens OX=9606 GN=ALDOA PE=1 SV=2 - [ALDOA_HUMA	25,27	2	9	10	17	4,797E7	2,111E6	28,37	21,98	9	15	0,00	6,87	2	2	364	39,4	8,09
Q969W8	Zinc finger protein 566 OS=Homo sapiens OX=9606 GN=ZNF566 PE=1 SV=1 - [ZN566_HUMAN]	1,67	1	1	1	1	0,000E0	2,056E6		0,00			0,00	1,67	1	1	418	49,2	8,06
P50750	Cyclin-dependent kinase 9 OS=Homo sapiens OX=9606 GN=CDK9 PE=1 SV=3 - [CDK9_HUMAN]	2,42	3	1	1	1	0,000E0	2,012E6		0,00			0,00	2,42	1	1	372	42,8	8,79
Q9UBL3	Set1/Ash2 histone methyltransferase complex subunit ASH2 OS=Homo sapiens OX=9606 GN=ASH2L PE=	4,14	1	2	2	2	0,000E0	1,998E6		0,00			0,00	4,14	2	2	628	68,7	5,69
P16615	Sarcoplasmic/endoplasmic reticulum calcium ATPase 2 OS=Homo sapiens OX=9606 GN=ATP2A2 PE=1 SV	4,13	1	3	3	3	1,794E6	1,994E6	0,00	3,26	2	2	0,00	0,86	1	1	1042	114,7	5,34
Q7KZ85	Transcription elongation factor SPT6 OS=Homo sapiens OX=9606 GN=SUPT6H PE=1 SV=2 - [SPT6H_HU	0,70	1	1	1	1	0,000E0	1,969E6		0,00			0,00	0,70	1	1	1726	198,9	4,91
Q14103	Heterogeneous nuclear ribonucleoprotein D0 OS=Homo sapiens OX=9606 GN=HNRNPD PE=1 SV=1 - [HN	5,92	1	2	2	3	1,486E7	1,946E6	5,32	5,92	1	2	0,00	5,35	1	1	355	38,4	7,81
Q9UKV3	Apoptotic chromatin condensation inducer in the nucleus OS=Homo sapiens OX=9606 GN=ACIN1 PE=1 SV	0,52	1	1	1	1	0,000E0	1,919E6		0,00			0,00	0,52	1	1	1341	151,8	6,43
P33176	Kinesin-1 heavy chain OS=Homo sapiens OX=9606 GN=KIF5B PE=1 SV=1 - [KINH_HUMAN]	1,04	1	1	1	1	0,000E0	1,896E6		0,00			0,00	1,04	1	1	963	109,6	6,51
P58317	Zinc finger protein 121 OS=Homo sapiens OX=9606 GN=ZNF121 PE=3 SV=2 - [ZN121_HUMAN]	3,33	1	1	1	1	0,000E0	1,873E6		0,00			0,00	3,33	1	1	390	44,7	8,05
Q9BX40	Protein LSM14 homolog B OS=Homo sapiens OX=9606 GN=LSM14B PE=1 SV=1 - [LS14B_HUMAN]	2,86	1	1	1	1	0,000E0	1,839E6		0,00			0,00	2,86	1	1	385	42,0	9,69

Q8IYB9	Zinc finger protein 595 OS=Homo sapiens OX=9606 GN=ZNF595 PE=2 SV=2 - [ZN595_HUMAN]	1,85	1	1	1	1	0,000E0	1,812E6		0,00			0,00	1,85	1	1	648	74,3	9,11
Q9P0S9	Transmembrane protein 14C OS=Homo sapiens OX=9606 GN=TMEM14C PE=1 SV=1 - [TM14C_HUMAN	8,93	1	1	1	1	0,000E0	1,783E6					0,00	8,93	1	1	112	11,6	9,88
Q15654	Thyroid receptor-interacting protein 6 OS=Homo sapiens OX=9606 GN=TRIP6 PE=1 SV=3 - [TRIP6_HUM	4,41	1	1	1	1	0,000E0	1,754E6		0,00			0,00	4,41	1	1	476	50,3	7,37
Q99735	Microsomal glutathione S-transferase 2 OS=Homo sapiens OX=9606 GN=MGST2 PE=1 SV=1 - [MGST2_H	9,52	1	1	1	1	0,000E0	1,715E6		0,00			0,00	9,52	1	1	147	16,6	9,55
Q9NXW2	DnaJ homolog subfamily B member 12 OS=Homo sapiens OX=9606 GN=DNAJB12 PE=1 SV=4 - [DJB12_	2,67	1	1	1	1	0,000E0	1,699E6		0,00			0,00	2,67	1	1	375	41,8	8,53
Q9NRA8	Eukaryotic translation initiation factor 4E transporter OS=Homo sapiens OX=9606 GN=EIF4ENIF1 PE=1 SV	1,12	1	1	1	1	0,000E0	1,665E6		0,00			0,00	1,12	1	1	985	108,1	8,32
Q8TF32	Zinc finger protein 431 OS=Homo sapiens OX=9606 GN=ZNF431 PE=2 SV=2 - [ZN431_HUMAN]	2,08	1	1	1	1	0,000E0	1,607E6		0,00			0,00	2,08	1	1	576	67,2	8,76
O43309	Zinc finger and SCAN domain-containing protein 12 OS=Homo sapiens OX=9606 GN=ZSCAN12 PE=1 SV	1,99	1	1	1	1	0,000E0	1,583E6		0,00			0,00	1,99	1	1	604	70,2	6,74
Q9Y4L1	Hypoxia up-regulated protein 1 OS=Homo sapiens OX=9606 GN=HYOU1 PE=1 SV=1 - [HYOU1_HUMAN	3,10	1	2	2	2	3,999E6	1,560E6	1,85	1,50	1	1	0,00	1,60	1	1	999	111,3	5,22
O00165	HCLS1-associated protein X-1 OS=Homo sapiens OX=9606 GN=HAX1 PE=1 SV=2 - [HAX1_HUMAN]	4,30	1	1	1	1	0,000E0	1,486E6		0,00			0,00	4,30	1	1	279	31,6	4,92
Q8TCJ2	Dolichyl-diphosphooligosaccharide--protein glycosyltransferase subunit STT3B OS=Homo sapiens OX=9606	0,85	1	1	1	1	0,000E0	1,480E6		0,00			0,00	0,85	1	1	826	93,6	8,91
P30414	NK-tumor recognition protein OS=Homo sapiens OX=9606 GN=NKTR PE=1 SV=2 - [NKTR_HUMAN]	0,75	1	1	1	1	0,000E0	1,458E6		0,00			0,00	0,75	1	1	1462	165,6	9,99
Q13200	26S proteasome non-ATPase regulatory subunit 2 OS=Homo sapiens OX=9606 GN=PSMD2 PE=1 SV=3 - [	6,94	1	4	4	4	3,458E6	1,324E6	11,37	5,84	3	3	0,00	1,10	1	1	908	100,1	5,20
Q504Q3	PAN2-PAN3 deadenylation complex catalytic subunit PAN2 OS=Homo sapiens OX=9606 GN=PAN2 PE=1	0,75	1	1	1	1	0,000E0	1,284E6		0,00			0,00	0,75	1	1	1202	135,3	5,99
Q9UQ88	Cyclin-dependent kinase 11A OS=Homo sapiens OX=9606 GN=CDK11A PE=1 SV=4 - [CD11A_HUMAN]	2,17	2	1	1	1	0,000E0	1,253E6		0,00			0,00	2,17	1	1	783	91,3	5,36
Q02978	Mitochondrial 2-oxoglutarate/malate carrier protein OS=Homo sapiens OX=9606 GN=SLC25A11 PE=1 SV=	2,55	1	1	1	1	0,000E0	1,234E6		0,00			0,00	2,55	1	1	314	34,0	9,91
P11413	Glucose-6-phosphate 1-dehydrogenase OS=Homo sapiens OX=9606 GN=G6PD PE=1 SV=4 - [G6PD_HUM	6,99	1	3	3	3	2,648E6	1,209E6	1,99	5,05	2	2	0,00	1,94	1	1	515	59,2	6,84
Q9BV73	Centrosome-associated protein CEP250 OS=Homo sapiens OX=9606 GN=CEP250 PE=1 SV=2 - [CP250_H	0,74	1	1	1	1	0,000E0	1,182E6		0,00			0,00	0,74	1	1	2442	281,0	5,02
P07814	Bifunctional glutamate/proline--tRNA ligase OS=Homo sapiens OX=9606 GN=EPRS PE=1 SV=5 - [SYEP_	3,17	1	4	4	4	2,270E6	1,152E6	4,63	2,65	3	3	0,00	0,53	1	1	1512	170,5	7,33
O00231	26S proteasome non-ATPase regulatory subunit 11 OS=Homo sapiens OX=9606 GN=PSMD11 PE=1 SV=3	3,08	1	1	1	1	0,000E0	1,100E6		0,00			0,00	3,08	1	1	422	47,4	6,48
Q8NF37	Lysophosphatidylcholine acyltransferase 1 OS=Homo sapiens OX=9606 GN=LPCAT1 PE=1 SV=2 - [PCAT	1,50	1	1	1	1	0,000E0	1,082E6		0,00			0,00	1,50	1	1	534	59,1	6,02
Q3SXZ3	Zinc finger protein 718 OS=Homo sapiens OX=9606 GN=ZNF718 PE=2 SV=2 - [ZN718_HUMAN]	2,51	1	1	1	1	0,000E0	1,069E6		0,00			0,00	2,51	1	1	478	55,3	9,42
Q96BR9	Zinc finger and BTB domain-containing protein 8A OS=Homo sapiens OX=9606 GN=ZBTB8A PE=1 SV=2	3,40	1	1	1	1	0,000E0	1,043E6		0,00			0,00	3,40	1	1	441	50,1	7,24
Q12797	Aspartyl/asparaginyl beta-hydroxylase OS=Homo sapiens OX=9606 GN=ASPH PE=1 SV=3 - [ASPH_HUM	2,77	1	1	1	1	0,000E0	9,869E5		0,00			0,00	2,77	1	1	758	85,8	5,01
P61019	Ras-related protein Rab-2A OS=Homo sapiens OX=9606 GN=RAB2A PE=1 SV=1 - [RAB2A_HUMAN]	6,60	1	1	1	1	0,000E0	9,837E5		0,00			0,00	6,60	1	1	212	23,5	6,54
P47897	Glutamine--tRNA ligase OS=Homo sapiens OX=9606 GN=QARS PE=1 SV=1 - [SYQ_HUMAN]	4,00	1	2	2	2	1,082E6	9,431E5	0,00	1,94	1	1	0,00	2,06	1	1	775	87,7	7,15
O75534	Cold shock domain-containing protein E1 OS=Homo sapiens OX=9606 GN=CSDE1 PE=1 SV=2 - [CSDE1	4,14	1	3	3	3	2,206E6	9,009E5	1,77	2,88	2	2	0,00	1,25	1	1	798	88,8	6,25
Q96JG9	Zinc finger protein 469 OS=Homo sapiens OX=9606 GN=ZNF469 PE=2 SV=3 - [ZN469_HUMAN]	0,43	1	1	1	1	0,000E0	8,785E5		0,00			0,00	0,43	1	1	3925	409,9	7,72
Q8NGX8	Olfactory receptor 6Y1 OS=Homo sapiens OX=9606 GN=OR6Y1 PE=3 SV=1 - [OR6Y1_HUMAN]	9,85	1	1	1	1	0,000E0	8,781E5		0,00			0,00	9,85	1	1	325	36,6	8,06
Q9NX65	Zinc finger and SCAN domain-containing protein 32 OS=Homo sapiens OX=9606 GN=ZSCAN32 PE=1 SV	2,73	1	1	1	1	0,000E0	8,268E5		0,00			0,00	2,73	1	1	697	78,7	8,06
Q8TD17	Zinc finger protein 398 OS=Homo sapiens OX=9606 GN=ZNF398 PE=1 SV=1 - [ZN398_HUMAN]	1,40	1	1	1	1	0,000E0	8,265E5		0,00			0,00	1,40	1	1	642	71,3	6,67
Q9NX78	Transmembrane protein 260 OS=Homo sapiens OX=9606 GN=TMEM260 PE=1 SV=3 - [TM260_HUMAN]	3,96	1	1	1	2	0,000E0	8,173E5		0,00			0,00	3,96	1	2	707	79,5	8,40
Q32P28	Prolyl 3-hydroxylase 1 OS=Homo sapiens OX=9606 GN=P3H1 PE=1 SV=2 - [P3H1_HUMAN]	1,36	1	1	1	1	0,000E0	7,841E5		0,00			0,00	1,36	1	1	736	83,3	5,14
Q9H089	Large subunit GTPase 1 homolog OS=Homo sapiens OX=9606 GN=LSG1 PE=1 SV=2 - [LSG1_HUMAN]	1,22	1	1	1	1	0,000E0	7,518E5		0,00			0,00	1,22	1	1	658	75,2	6,38
Q9NXV6	CDKN2A-interacting protein OS=Homo sapiens OX=9606 GN=CDKN2AIP PE=1 SV=3 - [CARF_HUMAN	3,62	1	1	1	1	0,000E0	7,438E5		0,00			0,00	3,62	1	1	580	61,1	9,01
P49916	DNA ligase 3 OS=Homo sapiens OX=9606 GN=LIG3 PE=1 SV=2 - [DNLI3_HUMAN]	0,99	1	1	1	1	0,000E0	7,013E5		0,00			0,00	0,99	1	1	1009	112,8	9,01
Q92747	Actin-related protein 2/3 complex subunit 1A OS=Homo sapiens OX=9606 GN=ARPC1A PE=2 SV=2 - [AI	4,59	1	1	1	1	0,000E0	6,682E5		0,00			0,00	4,59	1	1	370	41,5	8,18
P31151	Protein S100-A7 OS=Homo sapiens OX=9606 GN=S100A7 PE=1 SV=4 - [S10A7_HUMAN]	21,78	2	2	2	16	4,673E8	5,415E5	14,93	21,78	2	15	0,00	10,89	1	1	101	11,5	6,77
Q9UBM7	7-dehydrocholesterol reductase OS=Homo sapiens OX=9606 GN=DHCR7 PE=1 SV=1 - [DHCR7_HUMAN]	1,68	1	1	1	1	0,000E0	4,400E5		0,00			0,00	1,68	1	1	475	54,5	8,70
O00268	Transcription initiation factor TFIID subunit 4 OS=Homo sapiens OX=9606 GN=TAF4 PE=1 SV=2 - [TAF4	0,92	1	1	1	1	0,000E0	4,313E5		0,00			0,00	0,92	1	1	1085	110,0	9,94
P35658	Nuclear pore complex protein Nup214 OS=Homo sapiens OX=9606 GN=NUP214 PE=1 SV=2 - [NU214_HU	0,48	1	1	1	1	0,000E0	3,403E5		0,00			0,00	0,48	1	1	2090	213,5	7,47
P17480	Nucleolar transcription factor 1 OS=Homo sapiens OX=9606 GN=UBTF PE=1 SV=1 - [UBF1_HUMAN]	1,44	1	1	1	1	0,000E0	3,298E5		0,00			0,00	1,44	1	1	764	89,4	5,81
Q96MX6	WD repeat-containing protein 92 OS=Homo sapiens OX=9606 GN=WDR92 PE=1 SV=1 - [WDR92_HUMA	9,24	1	2	2	2	0,000E0	2,926E5		0,00			0,00	9,24	2	2	357	39,7	8,09
Q14498	RNA-binding protein 39 OS=Homo sapiens OX=9606 GN=RBM39 PE=1 SV=2 - [RBM39_HUMAN]	2,83	1	1	1	1	0,000E0	1,901E5		0,00			0,00	2,83	1	1	530	59,3	10,10
O60488	Long-chain-fatty-acid--CoA ligase 4 OS=Homo sapiens OX=9606 GN=ACSL4 PE=1 SV=2 - [ACSL4_HUM	2,53	2	1	1	1	0,000E0	0,000E0		0,00			0,00	2,53	1	1	711	79,1	8,38
P59510	A disintegrin and metalloproteinase with thrombospondin motifs 20 OS=Homo sapiens OX=9606 GN=ADA	0,89	1	1	1	1	0,000E0	0,000E0		0,00			0,00	0,89	1	1	1910	214,6	7,21
P35613	Basigin OS=Homo sapiens OX=9606 GN=BSG PE=1 SV=2 - [BASI_HUMAN]	2,34	1	1	1	1	0,000E0	0,000E0		0,00			0,00	2,34	1	1	385	42,2	5,66
Q16543	Hsp90 co-chaperone Cdc37 OS=Homo sapiens OX=9606 GN=CDC37 PE=1 SV=1 - [CDC37_HUMAN]	2,91	1	1	1	1	0,000E0	0,000E0		0,00			0,00	2,91	1	1	378	44,4	5,25
Q9Y2G8	DnaJ homolog subfamily C member 16 OS=Homo sapiens OX=9606 GN=DNAJC16 PE=2 SV=3 - [DJC16	1,02	1	1	1	1	0,000E0	0,000E0		0,00			0,00	1,02	1	1	782	90,5	7,12
Q8TCX1	Cytoplasmic dynein 2 light intermediate chain 1 OS=Homo sapiens OX=9606 GN=DYNC2LI1 PE=1 SV=1	2,56	1	1	1	1	0,000E0	0,000E0		0,00			0,00	2,56	1	1	351	39,6	7,53
P55084	Trifunctional enzyme subunit beta, mitochondrial OS=Homo sapiens OX=9606 GN=HADHB PE=1 SV=3 -	6,33	1	2	2	2	2,018E6	0,000E0	0,00	3,38	1	1	0,00	2,95	1	1	474	51,3	9,41
P14314	Glucosidase 2 subunit beta OS=Homo sapiens OX=9606 GN=PRKCSH PE=1 SV=2 - [GLU2B_HUMAN]	2,46	1	1	1	2	1,621E6	0,000E0	2,38	2,46	1	1	0,00	2,46	1	1	528	59,4	4,41
P01861	Immunoglobulin heavy constant gamma 4 OS=Homo sapiens OX=9606 GN=IGHG4 PE=1 SV=1 - [IGHG4_	9,79	2	1	2	2	1,908E6	0,000E0	0,00	2,14	1	1	0,00	7,65	1	1	327	35,9	7,36
Q14573	Inositol 1,4,5-trisphosphate receptor type 3 OS=Homo sapiens OX=9606 GN=ITPR3 PE=1 SV=2 - [ITPR3_	0,34	1	1	1	1	0,000E0	0,000E0		0,00			0,00	0,34	1	1	2671	303,9	6,48
Q9Y468	Lethal(3)malignant brain tumor-like protein 1 OS=Homo sapiens OX=9606 GN=L3MBTL1 PE=1 SV=4 - [L	3,69	1	1	1	1	0,000E0	0,000E0		0,00			0,00	3,69	1	1	840	92,2	6,04
O00567	Nucleolar protein 56 OS=Homo sapiens OX=9606 GN=NOP56 PE=1 SV=4 - [NOP56_HUMAN]	5,05	1	2	2	2	6,559E6	0,000E0	4,02	2,86	1	1	0,00	2,19	1	1	594	66,0	9,19
Q9BZF1	Oxysterol-binding protein-related protein 8 OS=Homo sapiens OX=9606 GN=OSBPL8 PE=1 SV=3 - [OSBI	2,14	1	1	1	1	0,000E0	0,000E0		0,00			0,00	2,14	1	1	889	101,1	6,96
O00541	Pescadillo homolog OS=Homo sapiens OX=9606 GN=PES1 PE=1 SV=1 - [PESC_HUMAN]	1,02	2	1	1	1	0,000E0	0,000E0		0,00			0,00	1,02	1	1	588	68,0	7,33
P20339	Ras-related protein Rab-5A OS=Homo sapiens OX=9606 GN=RAB5A PE=1 SV=2 - [RAB5A_HUMAN]	10,23	3	2	2	2	2,969E6	0,000E0	0,00	5,12	1	1	0,00	5,12	1	1	215	23,6	8,15
P14678	Small nuclear ribonucleoprotein-associated proteins B and B' OS=Homo sapiens OX=9606 GN=SNRPB PE=	3,33	2	1	1	1	0,000E0	0,000E0		0,00			0,00	3,33	1	1	240	24,6	11,19
Q9Y5B9	FACT complex subunit SPT16 OS=Homo sapiens OX=9606 GN=SUPT16H PE=1 SV=1 - [SP16H_HUMA	6,02	1	3	3	3	1,896E6	0,000E0	3,45	4,39	2	2	0,00	1,62	1	1	1047	119,8	5,66
P42224	Signal transducer and activator of transcription 1-alpha/beta OS=Homo sapiens OX=9606 GN=STAT1 PE=1	1,73	1	1	1	1	0,000E0	0,000E0		0,00			0,00	1,73	1	1	750	87,3	6,05
Q9UPQ9	Trinucleotide repeat-containing gene 6B protein OS=Homo sapiens OX=9606 GN=TNRC6B PE=1 SV=4 - [	1,20	1	1	1	1	0,000E0	0,000E0		0,00			0,00	1,20	1	1	1833	193,9	6,76
P48643	T-complex protein 1 subunit epsilon OS=Homo sapiens OX=9606 GN=CCT5 PE=1 SV=1 - [TCPE_HUMA	18,30	1	6	6	6	9,353E6	0,000E0	5,61	17,01	5	5	0,00	1,29	1	1	541	59,6	5,66
A0A1B0GVZ9	Transmembrane protein 269 OS=Homo sapiens OX=9606 GN=TMEM269 PE=3 SV=1 - [TM269_HUMAN]	9,80	1	1	1	4	0,000E0	0,000E0		0,00			0,00	9,80	1	4	245	26,9	7,12
Q96QK1	Vacuolar protein sorting-associated protein 35 OS=Homo sapiens OX=9606 GN=VPS35 PE=1 SV=2 - [VPS	2,39	1	1	1	1	0,000E0	0,000E0		0,00			0,00	2,39	1	1	796	91,6	5,49
Q99873	Protein arginine N-methyltransferase 1 OS=Homo sapiens OX=9606 GN=PRMT1 PE=1 SV=3 - [ANM1_HU	5,66	2	2	2	2	6,259E6	0,000E0	0,00	2,96	1	1	0,00	2,70	1	1	371	42,4	5,35
Q9H0P0	Cytosolic 5'-nucleotidase 3A OS=Homo sapiens OX=9606 GN=NT5C3A PE=1 SV=3 - [5NT3A_HUMAN]	4,76	1	1	1	1	1,999E6	0,000E0	0,00	4,76	1	1		0,00			336	37,9	7,12
P52209	6-phosphogluconate dehydrogenase, decarboxylating OS=Homo sapiens OX=9606 GN=PGD PE=1 SV=3 - [	19,46	1	5	5	5	4,191E6	0,000E0	12,29	19,46	5	5		0,00			483	53,1	7,23
P00813	Adenosine deaminase OS=Homo sapiens OX=9606 GN=ADA PE=1 SV=3 - [ADA_HUMAN]	8,26	1	1	1	1	1,758E7	0,000E0	3,49	8,26	1	1		0,00			363	40,7	5,95
P55263	Adenosine kinase OS=Homo sapiens OX=9606 GN=ADK PE=1 SV=2 - [ADK_HUMAN]	5,52	1	1	1	1	2,858E6	0,000E0	3,76	5,52	1	1		0,00			362	40,5	6,70
P17174	Aspartate aminotransferase, cytoplasmic OS=Homo sapiens OX=9606 GN=GOT1 PE=1 SV=3 - [AATC_HU	8,47	1	2	2	2	7,573E5	0,000E0	2,04	8,47	2	2		0,00			413	46,2	7,01
P61221	ATP-binding cassette sub-family E member 1 OS=Homo sapiens OX=9606 GN=ABCE1 PE=1 SV=1 - [ABC	2,50	1	1	1	1	1,282E6	0,000E0	1,69	2,50	1	1		0,00			599	67,3	8,34
Q15027	Arf-GAP with coiled-coil, ANK repeat and PH domain-containing protein 1 OS=Homo sapiens OX=9606 GN	3,24	1	1	1	1	9,632E5	0,000E0	0,00	3,24	1	1		0,00			740	81,5	7,66
Q99798	Aconitate hydratase, mitochondrial OS=Homo sapiens OX=9606 GN=ACO2 PE=1 SV=2 - [ACON_HUMAN	1,41	1	1	1	1	2,220E6	0,000E0	0,00	1,41	1	1		0,00			780	85,4	7,61
P30153	Serine/threonine-protein phosphatase 2A 65 kDa regulatory subunit A alpha isoform OS=Homo sapiens OX=	5,60	2	2	2	3	4,046E6	0,000E0	7,87	5,60	2	3		0,00			589	65,3	5,11
P00505	Aspartate aminotransferase, mitochondrial OS=Homo sapiens OX=9606 GN=GOT2 PE=1 SV=3 - [AATM_H	4,65	1	1	1	1	1,684E6	0,000E0	2,55	4,65	1	1		0,00			430	47,5	9,01
P49748	Very long-chain specific acyl-CoA dehydrogenase, mitochondrial OS=Homo sapiens OX=9606 GN=ACADV	1,22	1	1	1	1	1,156E6	0,000E0	0,00	1,22	1	1		0,00			655	70,3	8,75
P53396	ATP-citrate synthase OS=Homo sapiens OX=9606 GN=ACLY PE=1 SV=3 - [ACLY_HUMAN]	2,45	1	2	2	2	1,364E6	0,000E0	3,43	2,45	2	2		0,00			1101	120,8	7,33
A8K2U0	Alpha-2-macroglobulin-like protein 1 OS=Homo sapiens OX=9606 GN=A2ML1 PE=1 SV=3 - [A2ML1_HU	1,38	1	2	2	2	4,309E6	0,000E0	0,00	1,38	2	2		0,00			1454	161,0	5,73
P11766	Alcohol dehydrogenase class-3 OS=Homo sapiens OX=9606 GN=ADH5 PE=1 SV=4 - [ADHX_HUMAN]	5,88	1	1	1	1	1,398E6	0,000E0	0,00	5,88	1	1		0,00			374	39,7	7,49
P49189	4-trimethylaminobutyraldehyde dehydrogenase OS=Homo sapiens OX=9606 GN=ALDH9A1 PE=1 SV=3 - [	8,50	1	3	3	3	3,739E6	0,000E0	7,18	8,50	3	3		0,00			494	53,8	5,87
P00352	Retinal dehydrogenase 1 OS=Homo sapiens OX=9606 GN=ALDH1A1 PE=1 SV=2 - [AL1A1_HUMAN]	1,40	2	1	1	1	5,784E6	0,000E0	2,08	1,40	1	1		0,00			501	54,8	6,73
P51648	Fatty aldehyde dehydrogenase OS=Homo sapiens OX=9606 GN=ALDH3A2 PE=1 SV=1 - [AL3A2_HUMAN	4,95	1	2	2	2	1,384E6	0,000E0	1,95	4,95	2	2		0,00			485	54,8	7,88
Q9NUJ1	Mycophenolic acid acyl-glucuronide esterase, mitochondrial OS=Homo sapiens OX=9606 GN=ABHD10 PE=	5,23	1	1	1	1	7,389E5	0,000E0	2,51	5,23	1	1		0,00			306	33,9	8,57
P07108	Acyl-CoA-binding protein OS=Homo sapiens OX=9606 GN=DBI PE=1 SV=2 - [ACBP_HUMAN]	41,38	1	2	2	2	5,120E6	0,000E0	2,11	41,38	2	2		0,00			87	10,0	6,57
P36405	ADP-ribosylation factor-like protein 3 OS=Homo sapiens OX=9606 GN=ARL3 PE=1 SV=2 - [ARL3_HUM	9,89	1	1	1	1	2,615E6	0,000E0	2,38	9,89	1	1					182	20,4	7,24
Q9ULZ3	Apoptosis-associated speck-like protein containing a CARD OS=Homo sapiens OX=9606 GN=PYCARD PE	7,18	1	1	1	1	7,615E5	0,000E0	0,00	7,18	1	1		0,00			195	21,6	6,34
O95994	Anterior gradient protein 2 homolog OS=Homo sapiens OX=9606 GN=AGR2 PE=1 SV=1 - [AGR2_HUMA	26,29	1	3	3	3	4,629E7	0,000E0	4,50	26,29	3	3		0,00			175	20,0	9,00
O43747	AP-1 complex subunit gamma-1 OS=Homo sapiens OX=9606 GN=AP1G1 PE=1 SV=5 - [AP1G1_HUMAN	5,11	1	2	2	3	3,291E6	0,000E0	7,95	5,11	2	3		0,00			822	91,3	6,80
P05089	Arginase-1 OS=Homo sapiens OX=9606 GN=ARG1 PE=1 SV=2 - [ARGI1_HUMAN]	16,77	1	6	6	7	1,870E7	0,000E0	6,39	16,77	6	7		0,00			322	34,7	7,21
Q9Y2T2	AP-3 complex subunit mu-1 OS=Homo sapiens OX=9606 GN=AP3M1 PE=1 SV=1 - [AP3M1_HUMAN]	4,78	1	1	1	1	1,289E6	0,000E0	0,00	4,78	1	1		0,00			418	46,9	6,93
Q9HDC9	Adipocyte plasma membrane-associated protein OS=Homo sapiens OX=9606 GN=APMAP PE=1 SV=2 - [A	5,53	1	1	1	1	1,438E6	0,000E0	3,40	5,53	1	1		0,00			416	46,5	6,16
P40616	ADP-ribosylation factor-like protein 1 OS=Homo sapiens OX=9606 GN=ARL1 PE=1 SV=1 - [ARL1_HUM	8,84	1	1	1	1	4,721E6	0,000E0	3,55	8,84	1	1		0,00			181	20,4	5,72
P08758	Annexin A5 OS=Homo sapiens OX=9606 GN=ANXA5 PE=1 SV=2 - [ANXA5_HUMAN]	10,00	1	2	3	3	3,117E6	0,000E0	2,00	10,00	3	3		0,00			320	35,9	5,05
Q7Z6R9	Transcription factor AP-2-delta OS=Homo sapiens OX=9606 GN=TFAP2D PE=2 SV=1 - [AP2D_HUMAN]	1,55	1	1	1	1	5,201E6	0,000E0	0,00	1,55	1	1		0,00			452	49,5	8,16
Q9BZZ5	Apoptosis inhibitor 5 OS=Homo sapiens OX=9606 GN=API5 PE=1 SV=3 - [API5_HUMAN]	3,44	1	1	1	1	1,339E6	0,000E0	2,09	3,44	1	1		0,00			524	59,0	7,34
O14983	Sarcoplasmic/endoplasmic reticulum calcium ATPase 1 OS=Homo sapiens OX=9606 GN=ATP2A1 PE=1 SV	2,10	2	1	1	1	1,180E6	0,000E0	0,00	2,10	1	1		0,00			1001	110,2	5,16
P12429	Annexin A3 OS=Homo sapiens OX=9606 GN=ANXA3 PE=1 SV=3 - [ANXA3_HUMAN]	4,95	1	1	1	1	4,384E5	0,000E0	0,00	4,95	1	1		0,00			323	36,4	5,92
P09525	Annexin A4 OS=Homo sapiens OX=9606 GN=ANXA4 PE=1 SV=4 - [ANXA4_HUMAN]	18,18	1	4	5	5	2,939E6	0,000E0	6,54	18,18	5	5		0,00			319	35,9	6,13
P27695	DNA-(apurinic or apyrimidinic site) lyase OS=Homo sapiens OX=9606 GN=APEX1 PE=1 SV=2 - [APEX1_	3,46	1	1	1	1	1,490E6	0,000E0	1,62	3,46	1	1		0,00			318	35,5	8,12
P09972	Fructose-bisphosphate aldolase C OS=Homo sapiens OX=9606 GN=ALDOC PE=1 SV=2 - [ALDOC_HUM	8,24	2	1	2	2	1,060E7	0,000E0	1,85	8,24	2	2		0,00			364	39,4	6,87
Q9BTT0	Acidic leucine-rich nuclear phosphoprotein 32 family member E OS=Homo sapiens OX=9606 GN=ANP32E	13,43	1	2	2	2	7,967E6	0,000E0	4,55	13,43	2	2		0,00			268	30,7	3,85
P55957	BH3-interacting domain death agonist OS=Homo sapiens OX=9606 GN=BID PE=1 SV=1 - [BID_HUMAN]	7,69	1	1	1	1	2,976E6	0,000E0	5,03	7,69	1	1		0,00			195	22,0	5,44
P53004	Biliverdin reductase A OS=Homo sapiens OX=9606 GN=BLVRA PE=1 SV=2 - [BIEA_HUMAN]	8,45	1	1	1	1	2,620E6	0,000E0	4,51	8,45	1	1		0,00			296	33,4	6,44
P24539	ATP synthase F(0) complex subunit B1, mitochondrial OS=Homo sapiens OX=9606 GN=ATP5F1 PE=1 SV	5,86	1	1	1	1	1,056E6	0,000E0	0,00	5,86	1	1		0,00			256	28,9	9,36
Q13510	Acid ceramidase OS=Homo sapiens OX=9606 GN=ASAH1 PE=1 SV=5 - [ASAH1_HUMAN]	2,78	1	1	1	1	1,395E6	0,000E0	0,00	2,78	1	1		0,00			395	44,6	7,62
P00966	Argininosuccinate synthase OS=Homo sapiens OX=9606 GN=ASS1 PE=1 SV=2 - [ASSY_HUMAN]	1,70	1	1	1	1	0,000E0	0,000E0	0,00	1,70	1	1		0,00			412	46,5	8,02
Q6PL18	ATPase family AAA domain-containing protein 2 OS=Homo sapiens OX=9606 GN=ATAD2 PE=1 SV=1 - [	1,58	1	1	1	1	7,721E5	0,000E0	2,29	1,58	1	1		0,00			1390	158,5	6,32
P07339	Cathepsin D OS=Homo sapiens OX=9606 GN=CTSD PE=1 SV=1 - [CATD_HUMAN]	10,19	1	4	4	5	3,641E7	0,000E0	5,85	10,19	4	5		0,00			412	44,5	6,54
Q9UI42	Carboxypeptidase A4 OS=Homo sapiens OX=9606 GN=CPA4 PE=1 SV=2 - [CBPA4_HUMAN]	6,89	1	3	3	3	1,044E7	0,000E0	1,65	6,89	3	3		0,00			421	47,3	6,70
Q14137	Ribosome biogenesis protein BOP1 OS=Homo sapiens OX=9606 GN=BOP1 PE=1 SV=2 - [BOP1_HUMAN	3,49	1	1	1	1	2,312E6	0,000E0	3,63	3,49	1	1		0,00			746	83,6	6,19
Q8TDN6	Ribosome biogenesis protein BRX1 homolog OS=Homo sapiens OX=9606 GN=BRIX1 PE=1 SV=2 - [BRX	3,12	1	1	1	1	5,992E5	0,000E0	1,80	3,12	1	1		0,00			353	41,4	9,92
P11586	C-1-tetrahydrofolate synthase, cytoplasmic OS=Homo sapiens OX=9606 GN=MTHFD1 PE=1 SV=3 - [C1TC	4,39	1	2	2	2	1,051E6	0,000E0	2,58	4,39	2	2		0,00			935	101,5	7,30
Q9Y6E2	Basic leucine zipper and W2 domain-containing protein 2 OS=Homo sapiens OX=9606 GN=BZW2 PE=1 SV	7,40	2	2	2	2	9,157E5	0,000E0	3,83	7,40	2	2		0,00			419	48,1	6,68
Q96K17	Transcription factor BTF3 homolog 4 OS=Homo sapiens OX=9606 GN=BTF3L4 PE=1 SV=1 - [BT3L4_HU	7,59	1	1	1	1	5,101E6	0,000E0	1,72	7,59	1	1		0,00			158	17,3	6,35
Q9BRK5	45 kDa calcium-binding protein OS=Homo sapiens OX=9606 GN=SDF4 PE=1 SV=1 - [CAB45_HUMAN]	7,73	1	1	1	1	2,817E6	0,000E0	0,00	7,73	1	1		0,00			362	41,8	4,86
P27797	Calreticulin OS=Homo sapiens OX=9606 GN=CALR PE=1 SV=1 - [CALR_HUMAN]	11,99	1	2	2	2	3,173E7	0,000E0	4,61	11,99	2	2		0,00			417	48,1	4,44
Q01518	Adenylyl cyclase-associated protein 1 OS=Homo sapiens OX=9606 GN=CAP1 PE=1 SV=5 - [CAP1_HUMA	9,26	2	3	3	3	2,076E6	0,000E0	3,79	9,26	3	3		0,00			475	51,9	8,06
P40121	Macrophage-capping protein OS=Homo sapiens OX=9606 GN=CAPG PE=1 SV=2 - [CAPG_HUMAN]	3,45	1	1	1	1	2,411E6	0,000E0	2,25	3,45	1	1		0,00			348	38,5	6,19
O43684	Mitotic checkpoint protein BUB3 OS=Homo sapiens OX=9606 GN=BUB3 PE=1 SV=1 - [BUB3_HUMAN]	5,79	1	1	1	1	6,693E5	0,000E0	0,00	5,79	1	1		0,00			328	37,1	6,84
Q13867	Bleomycin hydrolase OS=Homo sapiens OX=9606 GN=BLMH PE=1 SV=1 - [BLMH_HUMAN]	8,13	1	3	3	3	1,933E7	0,000E0	3,88	8,13	3	3		0,00			455	52,5	6,27
Q99622	Protein C10 OS=Homo sapiens OX=9606 GN=C12orf57 PE=1 SV=1 - [C10_HUMAN]	14,29	1	1	1	1	1,031E6	0,000E0	0,00	14,29	1	1					126	13,2	5,14
P30043	Flavin reductase (NADPH) OS=Homo sapiens OX=9606 GN=BLVRB PE=1 SV=3 - [BLVRB_HUMAN]	12,14	1	2	2	2	4,804E6	0,000E0	1,64	12,14	2	2		0,00			206	22,1	7,65
Q9NZT1	Calmodulin-like protein 5 OS=Homo sapiens OX=9606 GN=CALML5 PE=1 SV=2 - [CALL5_HUMAN]	64,38	1	7	7	9	2,100E8	0,000E0	19,14	64,38	7	9		0,00			146	15,9	4,44
P04040	Catalase OS=Homo sapiens OX=9606 GN=CAT PE=1 SV=3 - [CATA_HUMAN]	6,64	1	4	4	4	5,938E6	0,000E0	3,82	6,64	4	4		0,00			527	59,7	7,39
P09668	Pro-cathepsin H OS=Homo sapiens OX=9606 GN=CTSH PE=1 SV=4 - [CATH_HUMAN]	3,88	1	1	1	1	3,344E6	0,000E0	0,00	3,88	1	1		0,00			335	37,4	8,07
O60911	Cathepsin L2 OS=Homo sapiens OX=9606 GN=CTSV PE=1 SV=2 - [CATL2_HUMAN]	4,19	1	1	1	1	4,831E5	0,000E0	0,00	4,19	1	1		0,00			334	37,3	8,76
O00299	Chloride intracellular channel protein 1 OS=Homo sapiens OX=9606 GN=CLIC1 PE=1 SV=4 - [CLIC1_HU	4,98	1	1	1	1	1,083E7	0,000E0	2,10	4,98	1	1		0,00			241	26,9	5,17
Q9Y2V2	Calcium-regulated heat-stable protein 1 OS=Homo sapiens OX=9606 GN=CARHSP1 PE=1 SV=2 - [CHSP1	24,49	1	1	1	1	9,377E6	0,000E0	2,65	24,49	1	1		0,00			147	15,9	8,21
P00450	Ceruloplasmin OS=Homo sapiens OX=9606 GN=CP PE=1 SV=1 - [CERU_HUMAN]	2,07	1	2	2	2	3,580E6	0,000E0	0,00	2,07	2	2		0,00			1065	122,1	5,72
Q9Y6A4	Cilia- and flagella-associated protein 20 OS=Homo sapiens OX=9606 GN=CFAP20 PE=1 SV=1 - [CFA20_I	8,81	1	1	1	1	2,202E6	0,000E0	1,75	8,81	1	1		0,00			193	22,8	9,76
O14519	Cyclin-dependent kinase 2-associated protein 1 OS=Homo sapiens OX=9606 GN=CDK2AP1 PE=1 SV=1 - [	11,30	1	1	1	1	1,026E6	0,000E0	2,04	11,30	1	1					115	12,4	9,38
P60953	Cell division control protein 42 homolog OS=Homo sapiens OX=9606 GN=CDC42 PE=1 SV=2 - [CDC42_	8,90	1	1	1	1	8,135E6	0,000E0	0,00	8,90	1	1					191	21,2	6,55
Q8N163	Cell cycle and apoptosis regulator protein 2 OS=Homo sapiens OX=9606 GN=CCAR2 PE=1 SV=2 - [CCAI	0,98	1	1	1	1	2,212E6	0,000E0	0,00	0,98	1	1		0,00			923	102,8	5,22
P01730	T-cell surface glycoprotein CD4 OS=Homo sapiens OX=9606 GN=CD4 PE=1 SV=1 - [CD4_HUMAN]	3,71	1	1	1	1	5,608E5	0,000E0	0,00	3,71	1	1		0,00			458	51,1	9,54
Q15517	Corneodesmosin OS=Homo sapiens OX=9606 GN=CDSN PE=1 SV=3 - [CDSN_HUMAN]	5,10	1	2	2	3	3,816E7	0,000E0	2,07	5,10	2	3		0,00			529	51,5	8,35
Q9H444	Charged multivesicular body protein 4b OS=Homo sapiens OX=9606 GN=CHMP4B PE=1 SV=1 - [CHM4E	12,05	1	1	1	1	2,903E6	0,000E0	2,07	12,05	1	1		0,00			224	24,9	4,82
P61604	10 kDa heat shock protein, mitochondrial OS=Homo sapiens OX=9606 GN=HSPE1 PE=1 SV=2 - [CH10_H	5,88	1	1	1	1	4,292E6	0,000E0	0,00	5,88	1	1					102	10,9	8,92
Q14011	Cold-inducible RNA-binding protein OS=Homo sapiens OX=9606 GN=CIRBP PE=1 SV=1 - [CIRBP_HUN	8,14	1	1	1	1	5,841E6	0,000E0	2,41	8,14	1	1		0,00			172	18,6	9,51
Q14839	Chromodomain-helicase-DNA-binding protein 4 OS=Homo sapiens OX=9606 GN=CHD4 PE=1 SV=2 - [CH	1,31	1	2	2	3	7,873E6	0,000E0	8,48	1,31	2	3		0,00			1912	217,9	5,86
Q96JB5	CDK5 regulatory subunit-associated protein 3 OS=Homo sapiens OX=9606 GN=CDK5RAP3 PE=1 SV=2 -	2,77	1	1	1	1	9,876E5	0,000E0	2,49	2,77	1	1		0,00			506	56,9	4,75
Q9BR76	Coronin-1B OS=Homo sapiens OX=9606 GN=CORO1B PE=1 SV=1 - [COR1B_HUMAN]	5,93	1	1	1	1	4,566E6	0,000E0	0,00	5,93	1	1		0,00			489	54,2	5,88
O96005	Cleft lip and palate transmembrane protein 1 OS=Homo sapiens OX=9606 GN=CLPTM1 PE=1 SV=1 - [CLI	3,29	1	1	1	1	1,099E6	0,000E0	0,00	3,29	1	1		0,00			669	76,0	6,30
Q99829	Copine-1 OS=Homo sapiens OX=9606 GN=CPNE1 PE=1 SV=1 - [CPNE1_HUMAN]	3,72	1	1	1	1	1,637E6	0,000E0	2,59	3,72	1	1		0,00			537	59,0	5,83
P50416	Carnitine O-palmitoyltransferase 1, liver isoform OS=Homo sapiens OX=9606 GN=CPT1A PE=1 SV=2 - [C	2,20	1	1	1	1	4,990E5	0,000E0	1,92	2,20	1	1		0,00			773	88,3	8,65
P35606	Coatomer subunit beta' OS=Homo sapiens OX=9606 GN=COPB2 PE=1 SV=2 - [COPB2_HUMAN]	3,42	1	1	1	1	5,868E6	0,000E0	3,20	3,42	1	1		0,00			906	102,4	5,27
Q9Y678	Coatomer subunit gamma-1 OS=Homo sapiens OX=9606 GN=COPG1 PE=1 SV=1 - [COPG1_HUMAN]	1,95	1	1	1	1	3,234E6	0,000E0	0,00	1,95	1	1		0,00			874	97,7	5,47
P61923	Coatomer subunit zeta-1 OS=Homo sapiens OX=9606 GN=COPZ1 PE=1 SV=1 - [COPZ1_HUMAN]	18,64	1	1	1	1	5,238E6	0,000E0	5,33	18,64	1	1					177	20,2	4,81
P31146	Coronin-1A OS=Homo sapiens OX=9606 GN=CORO1A PE=1 SV=4 - [COR1A_HUMAN]	4,34	1	1	1	1	4,025E6	0,000E0	0,00	4,34	1	1		0,00			461	51,0	6,68
O95833	Chloride intracellular channel protein 3 OS=Homo sapiens OX=9606 GN=CLIC3 PE=1 SV=2 - [CLIC3_HU	13,14	2	2	2	2	8,458E5	0,000E0	3,00	13,14	2	2		0,00			236	26,6	6,43
Q15021	Condensin complex subunit 1 OS=Homo sapiens OX=9606 GN=NCAPD2 PE=1 SV=3 - [CND1_HUMAN]	1,14	1	1	1	1	5,989E5	0,000E0	0,00	1,14	1	1		0,00			1401	157,1	6,61
Q9Y2B0	Protein canopy homolog 2 OS=Homo sapiens OX=9606 GN=CNPY2 PE=1 SV=1 - [CNPY2_HUMAN]	8,79	1	1	1	1	1,313E6	0,000E0	0,00	8,79	1	1		0,00			182	20,6	4,92
P83436	Conserved oligomeric Golgi complex subunit 7 OS=Homo sapiens OX=9606 GN=COG7 PE=1 SV=1 - [CO	2,21	1	1	1	1	1,061E6	0,000E0	2,98	2,21	1	1		0,00			770	86,3	5,47
Q5TZA2	Rootletin OS=Homo sapiens OX=9606 GN=CROCC PE=1 SV=1 - [CROCC_HUMAN]	0,55	1	1	1	1	2,670E7	0,000E0	2,13	0,55	1	1		0,00			2017	228,4	5,50
P04080	Cystatin-B OS=Homo sapiens OX=9606 GN=CSTB PE=1 SV=2 - [CYTB_HUMAN]	12,24	1	1	1	1	5,187E6	0,000E0	0,00	12,24	1	1					98	11,1	7,56
P04632	Calpain small subunit 1 OS=Homo sapiens OX=9606 GN=CAPNS1 PE=1 SV=1 - [CPNS1_HUMAN]	8,58	1	1	1	1	1,942E6	0,000E0	2,14	8,58	1	1		0,00			268	28,3	5,20
Q96L46	Calpain small subunit 2 OS=Homo sapiens OX=9606 GN=CAPNS2 PE=2 SV=1 - [CPNS2_HUMAN]	5,24	1	1	1	1	8,303E5	0,000E0	0,00	5,24	1	1		0,00			248	27,6	5,73
P61201	COP9 signalosome complex subunit 2 OS=Homo sapiens OX=9606 GN=COPS2 PE=1 SV=1 - [CSN2_HU	3,84	1	1	1	1	1,108E6	0,000E0	2,69	3,84	1	1		0,00			443	51,6	5,53
Q9BT78	COP9 signalosome complex subunit 4 OS=Homo sapiens OX=9606 GN=COPS4 PE=1 SV=1 - [CSN4_HU	4,93	1	1	1	1	2,505E6	0,000E0	2,69	4,93	1	1		0,00			406	46,2	5,83
P99999	Cytochrome c OS=Homo sapiens OX=9606 GN=CYCS PE=1 SV=2 - [CYC_HUMAN]	16,19	1	1	1	1	2,322E6	0,000E0	1,87	16,19	1	1		0,00			105	11,7	9,57
P41240	Tyrosine-protein kinase CSK OS=Homo sapiens OX=9606 GN=CSK PE=1 SV=1 - [CSK_HUMAN]	4,22	1	1	1	1	4,568E5	0,000E0	0,00	4,22	1	1		0,00			450	50,7	7,06
Q9H9Q2	COP9 signalosome complex subunit 7b OS=Homo sapiens OX=9606 GN=COPS7B PE=1 SV=1 - [CSN7B	6,82	1	1	1	1	1,948E6	0,000E0	3,23	6,82	1	1		0,00			264	29,6	6,15
P33240	Cleavage stimulation factor subunit 2 OS=Homo sapiens OX=9606 GN=CSTF2 PE=1 SV=1 - [CSTF2_HU	6,24	1	1	2	2	9,113E5	0,000E0	2,85	6,24	2	2		0,00			577	60,9	6,83
Q15828	Cystatin-M OS=Homo sapiens OX=9606 GN=CST6 PE=1 SV=1 - [CYTM_HUMAN]	7,38	1	1	1	1	1,769E7	0,000E0	1,91	7,38	1	1		0,00			149	16,5	8,09
Q9H0L4	Cleavage stimulation factor subunit 2 tau variant OS=Homo sapiens OX=9606 GN=CSTF2T PE=1 SV=1 - [	5,84	1	1	2	2	6,891E5	0,000E0	2,85	5,84	2	2		0,00			616	64,4	7,25
P08574	Cytochrome c1, heme protein, mitochondrial OS=Homo sapiens OX=9606 GN=CYC1 PE=1 SV=3 - [CY1_	12,31	1	1	1	1	1,088E6	0,000E0	2,88	12,31	1	1		0,00			325	35,4	9,00
O43169	Cytochrome b5 type B OS=Homo sapiens OX=9606 GN=CYB5B PE=1 SV=2 - [CYB5B_HUMAN]	23,29	1	1	1	1	0,000E0	0,000E0	3,63	23,29	1	1					146	16,3	4,97

P81605	Dermcidin OS=Homo sapiens OX=9606 GN=DCD PE=1 SV=2 - [DCD_HUMAN]	10,00	1	1	1	2	1,365E8	0,000E0	2,06	10,00	1	2					110	11,3	6,54
Q9UJU6	Drebrin-like protein OS=Homo sapiens OX=9606 GN=DBNL PE=1 SV=1 - [DBNL_HUMAN]	4,19	1	1	1	1	8,651E5	0,000E0	0,00	4,19	1	1		0,00			430	48,2	5,05
P60981	Destrin OS=Homo sapiens OX=9606 GN=DSTN PE=1 SV=3 - [DEST_HUMAN]	9,70	1	1	1	1	3,990E5	0,000E0	0,00	9,70	1	1		0,00			165	18,5	7,85
Q13363	C-terminal-binding protein 1 OS=Homo sapiens OX=9606 GN=CTBP1 PE=1 SV=2 - [CTBP1_HUMAN]	4,32	1	1	1	1	2,115E6	0,000E0	0,00	4,32	1	1		0,00			440	47,5	6,77
P35221	Catenin alpha-1 OS=Homo sapiens OX=9606 GN=CTNNA1 PE=1 SV=1 - [CTNA1_HUMAN]	2,76	1	1	1	1	1,366E6	0,000E0	2,63	2,76	1	1		0,00			906	100,0	6,29
Q13618	Cullin-3 OS=Homo sapiens OX=9606 GN=CUL3 PE=1 SV=2 - [CUL3_HUMAN]	1,56	1	1	1	1	5,533E5	0,000E0	0,00	1,56	1	1		0,00			768	88,9	8,48
Q96EP5	DAZ-associated protein 1 OS=Homo sapiens OX=9606 GN=DAZAP1 PE=1 SV=1 - [DAZP1_HUMAN]	3,93	1	1	1	1	6,796E5	0,000E0	0,00	3,93	1	1		0,00			407	43,4	8,56
P27707	Deoxycytidine kinase OS=Homo sapiens OX=9606 GN=DCK PE=1 SV=1 - [DCK_HUMAN]	8,08	1	1	1	1	1,154E6	0,000E0	2,73	8,08	1	1					260	30,5	5,21
P00367	Glutamate dehydrogenase 1, mitochondrial OS=Homo sapiens OX=9606 GN=GLUD1 PE=1 SV=2 - [DHE3_	2,87	1	1	1	1	0,000E0	0,000E0	0,00	2,87	1	1		0,00			558	61,4	7,80
Q9NVM6	DnaJ homolog subfamily C member 17 OS=Homo sapiens OX=9606 GN=DNAJC17 PE=1 SV=1 - [DJC17_	6,91	1	1	1	1	4,696E5	0,000E0	2,01	6,91	1	1		0,00			304	34,7	8,53
P09622	Dihydrolipoyl dehydrogenase, mitochondrial OS=Homo sapiens OX=9606 GN=DLD PE=1 SV=2 - [DLDH_	2,75	1	1	1	1	5,494E6	0,000E0	0,00	2,75	1	1		0,00			509	54,1	7,85
P06132	Uroporphyrinogen decarboxylase OS=Homo sapiens OX=9606 GN=UROD PE=1 SV=2 - [DCUP_HUMAN]	5,99	1	1	1	1	6,351E5	0,000E0	0,00	5,99	1	1		0,00			367	40,8	6,14
Q86YN1	Dolichyldiphosphatase 1 OS=Homo sapiens OX=9606 GN=DOLPP1 PE=2 SV=1 - [DOPP1_HUMAN]	7,14	1	1	1	1	4,911E5	0,000E0	0,00	7,14	1	1		0,00			238	27,0	9,44
Q13409	Cytoplasmic dynein 1 intermediate chain 2 OS=Homo sapiens OX=9606 GN=DYNC1I2 PE=1 SV=3 - [DC1	3,76	1	1	1	1	0,000E0	0,000E0	0,00	3,76	1	1		0,00			638	71,4	5,20
Q14203	Dynactin subunit 1 OS=Homo sapiens OX=9606 GN=DCTN1 PE=1 SV=3 - [DCTN1_HUMAN]	1,49	1	1	1	1	1,160E6	0,000E0	0,00	1,49	1	1		0,00			1278	141,6	5,81
Q9NR30	Nucleolar RNA helicase 2 OS=Homo sapiens OX=9606 GN=DDX21 PE=1 SV=5 - [DDX21_HUMAN]	4,60	1	2	2	2	2,103E6	0,000E0	6,07	4,60	2	2		0,00			783	87,3	9,28
Q9H773	dCTP pyrophosphatase 1 OS=Homo sapiens OX=9606 GN=DCTPP1 PE=1 SV=1 - [DCTP1_HUMAN]	11,76	1	1	1	1	0,000E0	0,000E0	0,00	11,76	1	1		0,00			170	18,7	5,03
Q9BQ39	ATP-dependent RNA helicase DDX50 OS=Homo sapiens OX=9606 GN=DDX50 PE=1 SV=1 - [DDX50_HU	3,39	1	1	1	1	2,702E6	0,000E0	0,00	3,39	1	1		0,00			737	82,5	9,17
P51659	Peroxisomal multifunctional enzyme type 2 OS=Homo sapiens OX=9606 GN=HSD17B4 PE=1 SV=3 - [DH	1,22	1	1	1	1	5,629E5	0,000E0	0,00	1,22	1	1		0,00			736	79,6	8,84
P18858	DNA ligase 1 OS=Homo sapiens OX=9606 GN=LIG1 PE=1 SV=1 - [DNLI1_HUMAN]	1,52	1	1	1	1	1,045E6	0,000E0	2,32	1,52	1	1		0,00			919	101,7	5,62
P13762	HLA class II histocompatibility antigen, DR beta 4 chain OS=Homo sapiens OX=9606 GN=HLA-DRB4 PE=	10,15	1	1	1	1	0,000E0	0,000E0	0,00	10,15	1	1		0,00			266	29,9	7,33
Q9NY33	Dipeptidyl peptidase 3 OS=Homo sapiens OX=9606 GN=DPP3 PE=1 SV=2 - [DPP3_HUMAN]	1,22	1	1	1	1	2,683E6	0,000E0	0,00	1,22	1	1		0,00			737	82,5	5,10
O00148	ATP-dependent RNA helicase DDX39A OS=Homo sapiens OX=9606 GN=DDX39A PE=1 SV=2 - [DX39A_	6,32	1	1	2	2	1,018E7	0,000E0	1,95	6,32	2	2		0,00			427	49,1	5,68
Q13838	Spliceosome RNA helicase DDX39B OS=Homo sapiens OX=9606 GN=DDX39B PE=1 SV=1 - [DX39B_H	7,01	1	1	2	2	6,167E6	0,000E0	4,61	7,01	2	2		0,00			428	49,0	5,67
Q14204	Cytoplasmic dynein 1 heavy chain 1 OS=Homo sapiens OX=9606 GN=DYNC1H1 PE=1 SV=5 - [DYHC1_	2,13	1	6	6	6	4,115E6	0,000E0	6,19	2,13	6	6		0,00			4646	532,1	6,40
P29692	Elongation factor 1-delta OS=Homo sapiens OX=9606 GN=EEF1D PE=1 SV=5 - [EF1D_HUMAN]	4,27	1	1	1	1	3,223E6	0,000E0	2,04	4,27	1	1		0,00			281	31,1	5,01
Q14574	Desmocollin-3 OS=Homo sapiens OX=9606 GN=DSC3 PE=1 SV=3 - [DSC3_HUMAN]	2,12	1	2	2	2	3,959E6	0,000E0	3,47	2,12	2	2		0,00			896	99,9	6,10
Q16610	Extracellular matrix protein 1 OS=Homo sapiens OX=9606 GN=ECM1 PE=1 SV=2 - [ECM1_HUMAN]	7,78	1	3	3	3	9,802E6	0,000E0	8,98	7,78	3	3		0,00			540	60,6	6,71
Q5VYK3	Proteasome adapter and scaffold protein ECM29 OS=Homo sapiens OX=9606 GN=ECPAS PE=1 SV=2 - [E	1,68	1	2	2	2	1,514E6	0,000E0	5,36	1,68	2	2		0,00			1845	204,2	7,12
Q6NXG1	Epithelial splicing regulatory protein 1 OS=Homo sapiens OX=9606 GN=ESRP1 PE=1 SV=2 - [ESRP1_HU	3,52	1	1	1	1	1,648E6	0,000E0	3,14	3,52	1	1		0,00			681	75,5	6,68
P15170	Eukaryotic peptide chain release factor GTP-binding subunit ERF3A OS=Homo sapiens OX=9606 GN=GSPT	5,01	1	1	1	1	1,958E6	0,000E0	0,00	5,01	1	1		0,00			499	55,7	5,62
Q8TE68	Epidermal growth factor receptor kinase substrate 8-like protein 1 OS=Homo sapiens OX=9606 GN=EPS8L1	0,97	1	1	1	1	1,105E6	0,000E0	0,00	0,97	1	1		0,00			723	80,2	6,04
Q9UHY7	Enolase-phosphatase E1 OS=Homo sapiens OX=9606 GN=ENOPH1 PE=1 SV=1 - [ENOPH_HUMAN]	4,60	1	1	1	1	3,854E5	0,000E0	0,00	4,60	1	1		0,00			261	28,9	4,78
P10768	S-formylglutathione hydrolase OS=Homo sapiens OX=9606 GN=ESD PE=1 SV=2 - [ESTD_HUMAN]	7,80	1	1	1	1	1,603E6	0,000E0	2,48	7,80	1	1		0,00			282	31,4	7,02
Q92817	Envoplakin OS=Homo sapiens OX=9606 GN=EVPL PE=1 SV=3 - [EVPL_HUMAN]	2,21	2	4	4	4	2,786E6	0,000E0	5,44	2,21	4	4		0,00			2033	231,5	6,96
O75844	CAAX prenyl protease 1 homolog OS=Homo sapiens OX=9606 GN=ZMPSTE24 PE=1 SV=2 - [FACE1_HU	3,37	1	1	1	1	3,033E6	0,000E0	3,03	3,37	1	1		0,00			475	54,8	7,49
Q14318	Peptidyl-prolyl cis-trans isomerase FKBP8 OS=Homo sapiens OX=9606 GN=FKBP8 PE=1 SV=2 - [FKBP8	3,64	1	1	1	1	1,105E6	0,000E0	0,00	3,64	1	1					412	44,5	4,84
P09104	Gamma-enolase OS=Homo sapiens OX=9606 GN=ENO2 PE=1 SV=3 - [ENOG_HUMAN]	18,89	2	2	4	5	2,852E7	0,000E0	10,29	18,89	4	5		0,00			434	47,2	5,03
P15311	Ezrin OS=Homo sapiens OX=9606 GN=EZR PE=1 SV=4 - [EZRI_HUMAN]	2,90	1	1	1	1	8,586E6	0,000E0	2,48	2,90	1	1		0,00			586	69,4	6,27
P09467	Fructose-1,6-bisphosphatase 1 OS=Homo sapiens OX=9606 GN=FBP1 PE=1 SV=5 - [F16P1_HUMAN]	2,96	1	1	1	1	0,000E0	0,000E0	2,61	2,96	1	1		0,00			338	36,8	6,99
P49327	Fatty acid synthase OS=Homo sapiens OX=9606 GN=FASN PE=1 SV=3 - [FAS_HUMAN]	6,01	1	9	9	9	1,359E7	0,000E0	25,83	6,01	9	9		0,00			2511	273,3	6,44
Q9BSJ8	Extended synaptotagmin-1 OS=Homo sapiens OX=9606 GN=ESYT1 PE=1 SV=1 - [ESYT1_HUMAN]	3,44	1	2	2	2	1,915E6	0,000E0	5,88	3,44	2	2		0,00			1104	122,8	5,83
P13804	Electron transfer flavoprotein subunit alpha, mitochondrial OS=Homo sapiens OX=9606 GN=ETFA PE=1 SV	6,01	1	1	1	1	1,576E6	0,000E0	3,05	6,01	1	1		0,00			333	35,1	8,38
Q52LJ0	Protein FAM98B OS=Homo sapiens OX=9606 GN=FAM98B PE=1 SV=1 - [FA98B_HUMAN]	5,76	1	1	1	1	1,596E6	0,000E0	0,00	5,76	1	1		0,00			330	37,2	6,29
P39748	Flap endonuclease 1 OS=Homo sapiens OX=9606 GN=FEN1 PE=1 SV=1 - [FEN1_HUMAN]	5,26	1	1	1	1	0,000E0	0,000E0	0,00	5,26	1	1		0,00			380	42,6	8,62
Q96I24	Far upstream element-binding protein 3 OS=Homo sapiens OX=9606 GN=FUBP3 PE=1 SV=2 - [FUBP3_H	1,40	2	1	1	2	1,727E6	0,000E0	0,00	1,40	1	2		0,00			572	61,6	8,38
Q5D862	Filaggrin-2 OS=Homo sapiens OX=9606 GN=FLG2 PE=1 SV=1 - [FILA2_HUMAN]	2,59	1	3	3	4	6,182E7	0,000E0	20,57	2,59	3	4		0,00			2391	247,9	8,31
P49354	Protein farnesyltransferase/geranylgeranyltransferase type-1 subunit alpha OS=Homo sapiens OX=9606 GN=F	4,75	1	1	1	1	9,535E5	0,000E0	3,71	4,75	1	1		0,00			379	44,4	5,08
Q16658	Fascin OS=Homo sapiens OX=9606 GN=FSCN1 PE=1 SV=3 - [FSCN1_HUMAN]	3,65	1	1	1	1	4,749E5	0,000E0	2,53	3,65	1	1		0,00			493	54,5	7,24
Q9UN86	Ras GTPase-activating protein-binding protein 2 OS=Homo sapiens OX=9606 GN=G3BP2 PE=1 SV=2 - [G	3,53	1	1	1	1	4,772E6	0,000E0	1,63	3,53	1	1		0,00			482	54,1	5,55
P06744	Glucose-6-phosphate isomerase OS=Homo sapiens OX=9606 GN=GPI PE=1 SV=4 - [G6PI_HUMAN]	12,01	1	3	3	3	1,940E7	0,000E0	9,14	12,01	3	3		0,00			558	63,1	8,32
P51570	Galactokinase OS=Homo sapiens OX=9606 GN=GALK1 PE=1 SV=1 - [GALK1_HUMAN]	7,91	1	1	1	1	1,486E6	0,000E0	0,00	7,91	1	1		0,00			392	42,2	6,46
Q96RT7	Gamma-tubulin complex component 6 OS=Homo sapiens OX=9606 GN=TUBGCP6 PE=1 SV=3 - [GCP6_	0,38	1	1	1	1	2,297E6	0,000E0	0,00	0,38	1	1		0,00			1819	200,4	6,32
O75223	Gamma-glutamylcyclotransferase OS=Homo sapiens OX=9606 GN=GGCT PE=1 SV=1 - [GGCT_HUMAN]	39,36	1	5	5	5	3,083E7	0,000E0	11,16	39,36	5	5		0,00			188	21,0	5,14
Q92947	Glutaryl-CoA dehydrogenase, mitochondrial OS=Homo sapiens OX=9606 GN=GCDH PE=1 SV=1 - [GCDH	5,94	1	1	1	1	9,435E5	0,000E0	0,00	5,94	1	1		0,00			438	48,1	8,06
Q92616	eIF-2-alpha kinase activator GCN1 OS=Homo sapiens OX=9606 GN=GCN1 PE=1 SV=6 - [GCN1_HUMAN	1,31	1	2	2	2	1,079E6	0,000E0	3,57	1,31	2	2		0,00			2671	292,6	7,47
O94925	Glutaminase kidney isoform, mitochondrial OS=Homo sapiens OX=9606 GN=GLS PE=1 SV=1 - [GLSK_H	3,14	1	1	1	1	6,559E5	0,000E0	1,68	3,14	1	1		0,00			669	73,4	7,77
Q16775	Hydroxyacylglutathione hydrolase, mitochondrial OS=Homo sapiens OX=9606 GN=HAGH PE=1 SV=2 - [C	3,90	1	1	1	1	1,506E5	0,000E0	0,00	3,90	1	1		0,00			308	33,8	8,12
O76003	Glutaredoxin-3 OS=Homo sapiens OX=9606 GN=GLRX3 PE=1 SV=2 - [GLRX3_HUMAN]	3,58	1	1	1	1	3,273E5	0,000E0	0,00	3,58	1	1		0,00			335	37,4	5,39
O95249	Golgi SNAP receptor complex member 1 OS=Homo sapiens OX=9606 GN=GOSR1 PE=1 SV=1 - [GOSR1_	6,00	1	1	1	1	4,130E5	0,000E0	2,29	6,00	1	1		0,00			250	28,6	9,42
P43304	Glycerol-3-phosphate dehydrogenase, mitochondrial OS=Homo sapiens OX=9606 GN=GPD2 PE=1 SV=3 - [	5,91	1	2	2	2	6,709E5	0,000E0	1,88	5,91	2	2		0,00			727	80,8	7,69
O75367	Core histone macro-H2A.1 OS=Homo sapiens OX=9606 GN=H2AFY PE=1 SV=4 - [H2AY_HUMAN]	4,57	2	1	1	1	1,681E6	0,000E0	0,00	4,57	1	1		0,00			372	39,6	9,79
Q9H4A5	Golgi phosphoprotein 3-like OS=Homo sapiens OX=9606 GN=GOLPH3L PE=1 SV=1 - [GLP3L_HUMAN]	4,56	2	1	1	1	6,737E5	0,000E0	2,15	4,56	1	1		0,00			285	32,7	5,83
P04062	Glucosylceramidase OS=Homo sapiens OX=9606 GN=GBA PE=1 SV=3 - [GLCM_HUMAN]	3,36	1	2	2	2	2,134E6	0,000E0	0,00	3,36	2	2		0,00			536	59,7	7,61
Q96QA5	Gasdermin-A OS=Homo sapiens OX=9606 GN=GSDMA PE=1 SV=4 - [GSDMA_HUMAN]	2,02	1	1	1	1	1,635E7	0,000E0	1,72	2,02	1	1		0,00			445	49,3	5,29
P78347	General transcription factor II-I OS=Homo sapiens OX=9606 GN=GTF2I PE=1 SV=2 - [GTF2I_HUMAN]	2,51	1	1	1	1	0,000E0	0,000E0	3,21	2,51	1	1		0,00			998	112,3	6,39
P50395	Rab GDP dissociation inhibitor beta OS=Homo sapiens OX=9606 GN=GDI2 PE=1 SV=2 - [GDIB_HUMAN	22,25	2	7	7	7	1,502E7	0,000E0	8,14	22,25	7	7		0,00			445	50,6	6,47
P34897	Serine hydroxymethyltransferase, mitochondrial OS=Homo sapiens OX=9606 GN=SHMT2 PE=1 SV=3 - [G	2,18	1	1	1	1	6,003E5	0,000E0	1,74	2,18	1	1		0,00			504	56,0	8,53
P52566	Rho GDP-dissociation inhibitor 2 OS=Homo sapiens OX=9606 GN=ARHGDIB PE=1 SV=3 - [GDIR2_HU	16,92	1	2	2	2	1,608E7	0,000E0	2,30	16,92	2	2		0,00			201	23,0	5,21
P52306	Rap1 GTPase-GDP dissociation stimulator 1 OS=Homo sapiens OX=9606 GN=RAP1GDS1 PE=1 SV=3 - [	2,97	1	1	1	1	1,001E6	0,000E0	3,29	2,97	1	1		0,00			607	66,3	5,31
Q92820	Gamma-glutamyl hydrolase OS=Homo sapiens OX=9606 GN=GGH PE=1 SV=2 - [GGH_HUMAN]	7,23	1	2	2	2	2,858E6	0,000E0	4,16	7,23	2	2		0,00			318	35,9	7,11
P35754	Glutaredoxin-1 OS=Homo sapiens OX=9606 GN=GLRX PE=1 SV=2 - [GLRX1_HUMAN]	10,38	1	1	1	1	2,579E6	0,000E0	1,77	10,38	1	1					106	11,8	8,09
O60234	Glia maturation factor gamma OS=Homo sapiens OX=9606 GN=GMFG PE=1 SV=1 - [GMFG_HUMAN]	12,68	1	1	1	1	5,118E6	0,000E0	0,00	12,68	1	1		0,00			142	16,8	5,26
P49915	GMP synthase [glutamine-hydrolyzing] OS=Homo sapiens OX=9606 GN=GMPS PE=1 SV=1 - [GUAA_HU	4,62	1	2	2	3	5,216E6	0,000E0	3,86	4,62	2	3		0,00			693	76,7	6,87
Q08623	Pseudouridine-5'-phosphatase OS=Homo sapiens OX=9606 GN=PUDP PE=1 SV=3 - [HDHD1_HUMAN]	7,89	1	1	1	1	1,513E6	0,000E0	4,05	7,89	1	1		0,00			228	25,2	5,31
P51858	Hepatoma-derived growth factor OS=Homo sapiens OX=9606 GN=HDGF PE=1 SV=1 - [HDGF_HUMAN]	10,42	1	2	2	2	2,124E6	0,000E0	0,00	10,42	2	2		0,00			240	26,8	4,73
P30519	Heme oxygenase 2 OS=Homo sapiens OX=9606 GN=HMOX2 PE=1 SV=2 - [HMOX2_HUMAN]	6,01	1	1	1	1	3,419E5	0,000E0	0,00	6,01	1	1		0,00			316	36,0	5,41
P07305	Histone H1.0 OS=Homo sapiens OX=9606 GN=H1F0 PE=1 SV=3 - [H10_HUMAN]	6,70	1	1	1	1	3,304E6	0,000E0	2,31	6,70	1	1		0,00			194	20,9	10,84
Q6AI08	HEAT repeat-containing protein 6 OS=Homo sapiens OX=9606 GN=HEATR6 PE=1 SV=1 - [HEAT6_HUM	1,02	1	1	1	1	0,000E0	0,000E0	0,00	1,02	1	1		0,00			1181	128,7	7,03
P02790	Hemopexin OS=Homo sapiens OX=9606 GN=HPX PE=1 SV=2 - [HEMO_HUMAN]	2,38	1	1	1	1	1,886E6	0,000E0	0,00	2,38	1	1					462	51,6	7,02
P78417	Glutathione S-transferase omega-1 OS=Homo sapiens OX=9606 GN=GSTO1 PE=1 SV=2 - [GSTO1_HUMA	5,39	1	1	1	1	6,899E6	0,000E0	2,54	5,39	1	1		0,00			241	27,5	6,60
P09211	Glutathione S-transferase P OS=Homo sapiens OX=9606 GN=GSTP1 PE=1 SV=2 - [GSTP1_HUMAN]	8,57	1	1	1	1	2,641E6	0,000E0	0,00	8,57	1	1		0,00			210	23,3	5,64
Q9Y450	HBS1-like protein OS=Homo sapiens OX=9606 GN=HBS1L PE=1 SV=1 - [HBS1L_HUMAN]	2,63	1	1	1	1	1,838E6	0,000E0	0,00	2,63	1	1		0,00			684	75,4	6,61
Q16836	Hydroxyacyl-coenzyme A dehydrogenase, mitochondrial OS=Homo sapiens OX=9606 GN=HADH PE=1 SV	9,55	1	1	1	1	2,692E7	0,000E0	5,20	9,55	1	1		0,00			314	34,3	8,85
P09601	Heme oxygenase 1 OS=Homo sapiens OX=9606 GN=HMOX1 PE=1 SV=1 - [HMOX1_HUMAN]	8,33	1	2	2	2	4,558E6	0,000E0	4,40	8,33	2	2		0,00			288	32,8	8,25
P00492	Hypoxanthine-guanine phosphoribosyltransferase OS=Homo sapiens OX=9606 GN=HPRT1 PE=1 SV=2 - [H	5,96	1	1	1	1	9,976E6	0,000E0	2,13	5,96	1	1		0,00			218	24,6	6,68
O75874	Isocitrate dehydrogenase [NADP] cytoplasmic OS=Homo sapiens OX=9606 GN=IDH1 PE=1 SV=2 - [IDHC_	5,80	1	1	1	1	0,000E0	0,000E0	2,54	5,80	1	1		0,00			414	46,6	7,01
P01834	Immunoglobulin kappa constant OS=Homo sapiens OX=9606 GN=IGKC PE=1 SV=2 - [IGKC_HUMAN]	18,69	2	1	1	1	3,193E7	0,000E0	1,61	18,69	1	1		0,00			107	11,8	6,52
P26583	High mobility group protein B2 OS=Homo sapiens OX=9606 GN=HMGB2 PE=1 SV=2 - [HMGB2_HUMA	5,26	1	1	1	1	6,398E5	0,000E0	0,00	5,26	1	1		0,00			209	24,0	7,81
O14979	Heterogeneous nuclear ribonucleoprotein D-like OS=Homo sapiens OX=9606 GN=HNRNPDL PE=1 SV=3 -	4,52	1	1	1	1	2,240E7	0,000E0	2,15	4,52	1	1		0,00			420	46,4	9,57
Q9BUJ2	Heterogeneous nuclear ribonucleoprotein U-like protein 1 OS=Homo sapiens OX=9606 GN=HNRNPUL1 PE	3,15	1	1	1	1	1,332E6	0,000E0	3,06	3,15	1	1		0,00			856	95,7	6,92
P14735	Insulin-degrading enzyme OS=Homo sapiens OX=9606 GN=IDE PE=1 SV=4 - [IDE_HUMAN]	15,11	1	15	15	17	3,711E7	0,000E0	17,77	15,11	15	17		0,00			1019	117,9	6,61
P48735	Isocitrate dehydrogenase [NADP], mitochondrial OS=Homo sapiens OX=9606 GN=IDH2 PE=1 SV=2 - [IDH	9,29	1	2	2	2	0,000E0	0,000E0	2,09	9,29	2	2		0,00			452	50,9	8,69
P42357	Histidine ammonia-lyase OS=Homo sapiens OX=9606 GN=HAL PE=1 SV=1 - [HUTH_HUMAN]	6,39	1	4	4	4	9,350E6	0,000E0	4,71	6,39	4	4		0,00			657	72,7	6,95
A0A0B4J1U7	Immunoglobulin heavy variable 6-1 OS=Homo sapiens OX=9606 GN=IGHV6-1 PE=3 SV=1 - [HV601_HUM	5,79	1	1	1	1	8,802E5	0,000E0	0,00	5,79	1	1					121	13,5	9,20
Q15056	Eukaryotic translation initiation factor 4H OS=Homo sapiens OX=9606 GN=EIF4H PE=1 SV=5 - [IF4H_HU	5,24	1	1	1	1	1,346E7	0,000E0	1,84	5,24	1	1		0,00			248	27,4	7,23
P56537	Eukaryotic translation initiation factor 6 OS=Homo sapiens OX=9606 GN=EIF6 PE=1 SV=1 - [IF6_HUMAN	4,08	1	1	1	1	7,044E6	0,000E0	2,21	4,08	1	1					245	26,6	4,68
P01877	Immunoglobulin heavy constant alpha 2 OS=Homo sapiens OX=9606 GN=IGHA2 PE=1 SV=4 - [IGHA2_H	2,94	3	1	1	1	4,238E5	0,000E0	0,00	2,94	1	1		0,00			340	36,6	6,27
P01857	Immunoglobulin heavy constant gamma 1 OS=Homo sapiens OX=9606 GN=IGHG1 PE=1 SV=1 - [IGHG1_	5,76	3	1	2	2	2,502E6	0,000E0	1,64	5,76	2	2		0,00			330	36,1	8,19
Q15181	Inorganic pyrophosphatase OS=Homo sapiens OX=9606 GN=PPA1 PE=1 SV=2 - [IPYR_HUMAN]	16,96	1	4	4	5	5,161E6	0,000E0	0,00	16,96	4	5		0,00			289	32,6	5,86
Q9C075	Keratin, type I cytoskeletal 23 OS=Homo sapiens OX=9606 GN=KRT23 PE=1 SV=2 - [K1C23_HUMAN]	12,09	1	4	4	4	5,057E6	0,000E0	7,42	12,09	4	4		0,00			422	48,1	6,54
O14732	Inositol monophosphatase 2 OS=Homo sapiens OX=9606 GN=IMPA2 PE=1 SV=1 - [IMPA2_HUMAN]	3,47	1	1	1	1	2,623E6	0,000E0	0,00	3,47	1	1		0,00			288	31,3	6,61
Q27J81	Inverted formin-2 OS=Homo sapiens OX=9606 GN=INF2 PE=1 SV=2 - [INF2_HUMAN]	1,12	1	1	1	1	6,008E5	0,000E0	2,61	1,12	1	1		0,00			1249	135,5	5,38
P54819	Adenylate kinase 2, mitochondrial OS=Homo sapiens OX=9606 GN=AK2 PE=1 SV=2 - [KAD2_HUMAN]	15,90	1	2	2	2	4,636E6	0,000E0	5,24	15,90	2	2		0,00			239	26,5	7,81
P10644	cAMP-dependent protein kinase type I-alpha regulatory subunit OS=Homo sapiens OX=9606 GN=PRKAR1A	6,30	1	1	1	1	1,077E6	0,000E0	2,87	6,30	1	1		0,00			381	43,0	5,35
P30085	UMP-CMP kinase OS=Homo sapiens OX=9606 GN=CMPK1 PE=1 SV=3 - [KCY_HUMAN]	8,67	1	2	2	2	4,967E6	0,000E0	7,38	8,67	2	2		0,00			196	22,2	5,57
Q9Y337	Kallikrein-5 OS=Homo sapiens OX=9606 GN=KLK5 PE=1 SV=3 - [KLK5_HUMAN]	2,73	1	1	1	1	5,152E6	0,000E0	0,00	2,73	1	1		0,00			293	32,0	8,27
O00410	Importin-5 OS=Homo sapiens OX=9606 GN=IPO5 PE=1 SV=4 - [IPO5_HUMAN]	4,65	1	2	2	2	2,404E6	0,000E0	1,80	4,65	2	2		0,00			1097	123,5	4,94
Q96P70	Importin-9 OS=Homo sapiens OX=9606 GN=IPO9 PE=1 SV=3 - [IPO9_HUMAN]	1,73	1	1	1	1	4,753E5	0,000E0	0,00	1,73	1	1		0,00			1041	115,9	4,81
Q9H2U2	Inorganic pyrophosphatase 2, mitochondrial OS=Homo sapiens OX=9606 GN=PPA2 PE=1 SV=2 - [IPYR2_	4,79	1	1	1	1	2,443E6	0,000E0	3,10	4,79	1	1		0,00			334	37,9	7,39
Q7Z6Z7	E3 ubiquitin-protein ligase HUWE1 OS=Homo sapiens OX=9606 GN=HUWE1 PE=1 SV=3 - [HUWE1_HU	0,66	1	1	1	1	3,638E6	0,000E0	3,47	0,66	1	1		0,00			4374	481,6	5,22
A0A0A0MS15	Immunoglobulin heavy variable 3-49 OS=Homo sapiens OX=9606 GN=IGHV3-49 PE=3 SV=1 - [HV349_H	9,24	4	1	1	1	1,505E6	0,000E0	0,00	9,24	1	1		0,00			119	13,0	8,62
A0A0J9YX35	Immunoglobulin heavy variable 3-64D OS=Homo sapiens OX=9606 GN=IGHV3-64D PE=3 SV=1 - [HV64D	9,40	1	1	1	2	0,000E0	0,000E0	4,69	9,40	1	2					117	12,8	7,85
O00629	Importin subunit alpha-3 OS=Homo sapiens OX=9606 GN=KPNA4 PE=1 SV=1 - [IMA3_HUMAN]	3,26	1	1	1	1	2,906E6	0,000E0	5,13	3,26	1	1		0,00			521	57,9	4,96
P07476	Involucrin OS=Homo sapiens OX=9606 GN=IVL PE=1 SV=2 - [INVO_HUMAN]	9,91	1	4	4	4	9,823E6	0,000E0	2,55	9,91	4	4		0,00			585	68,4	4,61
Q96CN7	Isochorismatase domain-containing protein 1 OS=Homo sapiens OX=9606 GN=ISOC1 PE=1 SV=3 - [ISOC	12,42	1	2	2	2	2,430E6	0,000E0	3,41	12,42	2	2		0,00			298	32,2	7,39
P47929	Galectin-7 OS=Homo sapiens OX=9606 GN=LGALS7 PE=1 SV=2 - [LEG7_HUMAN]	39,71	1	5	5	10	2,234E8	0,000E0	27,25	39,71	5	10		0,00			136	15,1	7,62
Q9H008	Phospholysine phosphohistidine inorganic pyrophosphate phosphatase OS=Homo sapiens OX=9606 GN=LH	7,78	1	1	1	1	5,555E5	0,000E0	0,00	7,78	1	1		0,00			270	29,1	6,15
Q12907	Vesicular integral-membrane protein VIP36 OS=Homo sapiens OX=9606 GN=LMAN2 PE=1 SV=1 - [LMA	11,24	1	2	2	2	2,052E7	0,000E0	0,00	11,24	2	2					356	40,2	6,95
P36776	Lon protease homolog, mitochondrial OS=Homo sapiens OX=9606 GN=LONP1 PE=1 SV=2 - [LONM_HU	3,65	1	1	1	1	7,267E6	0,000E0	4,36	3,65	1	1		0,00			959	106,4	6,39
P07195	L-lactate dehydrogenase B chain OS=Homo sapiens OX=9606 GN=LDHB PE=1 SV=2 - [LDHB_HUMAN]	11,08	1	3	3	3	1,753E7	0,000E0	2,97	11,08	3	3					334	36,6	6,05
P43034	Platelet-activating factor acetylhydrolase IB subunit alpha OS=Homo sapiens OX=9606 GN=PAFAH1B1 PE	4,63	1	1	2	2	9,184E5	0,000E0	1,68	4,63	2	2		0,00			410	46,6	7,37
Q9NSB4	Keratin, type II cuticular Hb2 OS=Homo sapiens OX=9606 GN=KRT82 PE=3 SV=3 - [KRT82_HUMAN]	1,75	1	1	1	1	1,788E6	0,000E0	1,71	1,75	1	1		0,00			513	56,6	6,74
A0A0A0MT36	Immunoglobulin kappa variable 6D-21 OS=Homo sapiens OX=9606 GN=IGKV6D-21 PE=3 SV=1 - [KVD2	10,53	1	1	1	1	5,769E6	0,000E0	0,00	10,53	1	1		0,00			114	12,3	7,28
Q6PKG0	La-related protein 1 OS=Homo sapiens OX=9606 GN=LARP1 PE=1 SV=2 - [LARP1_HUMAN]	3,65	1	2	2	2	1,215E6	0,000E0	0,00	3,65	2	2		0,00			1096	123,4	8,82
Q14739	Lamin-B receptor OS=Homo sapiens OX=9606 GN=LBR PE=1 SV=2 - [LBR_HUMAN]	1,79	1	1	1	1	0,000E0	0,000E0	0,00	1,79	1	1		0,00			615	70,7	9,36
P00338	L-lactate dehydrogenase A chain OS=Homo sapiens OX=9606 GN=LDHA PE=1 SV=2 - [LDHA_HUMAN]	33,13	2	8	8	8	2,909E7	0,000E0	17,92	33,13	8	8		0,00			332	36,7	8,27
P17931	Galectin-3 OS=Homo sapiens OX=9606 GN=LGALS3 PE=1 SV=5 - [LEG3_HUMAN]	7,20	1	2	2	2	2,707E6	0,000E0	2,08	7,20	2	2					250	26,1	8,56
Q3ZCW2	Galectin-related protein OS=Homo sapiens OX=9606 GN=LGALSL PE=1 SV=2 - [LEGL_HUMAN]	5,23	1	1	1	1	3,777E6	0,000E0	1,67	5,23	1	1					172	19,0	5,35
P50851	Lipopolysaccharide-responsive and beige-like anchor protein OS=Homo sapiens OX=9606 GN=LRBA PE=1	0,49	1	1	1	1	2,156E5	0,000E0	0,00	0,49	1	1		0,00			2863	318,9	5,60
Q5VSP4	Putative lipocalin 1-like protein 1 OS=Homo sapiens OX=9606 GN=LCN1P1 PE=5 SV=1 - [LC1L1_HUMA	6,79	2	1	1	1	1,040E6	0,000E0	0,00	6,79	1	1					162	17,9	5,00
P05455	Lupus La protein OS=Homo sapiens OX=9606 GN=SSB PE=1 SV=2 - [LA_HUMAN]	2,94	1	1	1	1	0,000E0	0,000E0	2,18	2,94	1	1		0,00			408	46,8	7,12
Q5T749	Keratinocyte proline-rich protein OS=Homo sapiens OX=9606 GN=KPRP PE=1 SV=1 - [KPRP_HUMAN]	1,04	1	1	1	1	1,286E7	0,000E0	1,62	1,04	1	1		0,00			579	64,1	8,27
A0A075B6P5	Immunoglobulin kappa variable 2-28 OS=Homo sapiens OX=9606 GN=IGKV2-28 PE=3 SV=1 - [KV228_H	10,83	7	1	1	12	5,223E9	0,000E0	35,34	10,83	1	12		0,00			120	12,9	5,94
A0A0C4DH68	Immunoglobulin kappa variable 2-24 OS=Homo sapiens OX=9606 GN=IGKV2-24 PE=3 SV=1 - [KV224_H	10,83	1	1	1	1	7,759E6	0,000E0	2,21	10,83	1	1		0,00			120	13,1	8,53
Q04760	Lactoylglutathione lyase OS=Homo sapiens OX=9606 GN=GLO1 PE=1 SV=4 - [LGUL_HUMAN]	22,28	1	3	3	3	8,445E6	0,000E0	5,90	22,28	3	3		0,00			184	20,8	5,31
Q9Y4Y9	U6 snRNA-associated Sm-like protein LSm5 OS=Homo sapiens OX=9606 GN=LSM5 PE=1 SV=3 - [LSM5	25,27	1	1	1	1	8,571E5	0,000E0	0,00	25,27	1	1					91	9,9	4,54
Q6UWN5	Ly6/PLAUR domain-containing protein 5 OS=Homo sapiens OX=9606 GN=LYPD5 PE=1 SV=2 - [LYPD5	2,79	1	1	1	1	3,371E6	0,000E0	0,00	2,79	1	1		0,00			251	26,9	7,24
Q14566	DNA replication licensing factor MCM6 OS=Homo sapiens OX=9606 GN=MCM6 PE=1 SV=1 - [MCM6_H	3,53	1	2	2	2	8,996E6	0,000E0	5,81	3,53	2	2		0,00			821	92,8	5,41
P48163	NADP-dependent malic enzyme OS=Homo sapiens OX=9606 GN=ME1 PE=1 SV=1 - [MAOX_HUMAN]	6,29	1	4	4	4	3,790E6	0,000E0	1,74	6,29	4	4		0,00			572	64,1	6,13
P33991	DNA replication licensing factor MCM4 OS=Homo sapiens OX=9606 GN=MCM4 PE=1 SV=5 - [MCM4_H	2,20	1	1	1	1	1,024E7	0,000E0	3,42	2,20	1	1		0,00			863	96,5	6,74
P33993	DNA replication licensing factor MCM7 OS=Homo sapiens OX=9606 GN=MCM7 PE=1 SV=4 - [MCM7_H	5,56	1	3	3	3	8,252E5	0,000E0	1,63	5,56	3	3		0,00			719	81,3	6,46
P40925	Malate dehydrogenase, cytoplasmic OS=Homo sapiens OX=9606 GN=MDH1 PE=1 SV=4 - [MDHC_HUMA	11,38	1	1	1	1	0,000E0	0,000E0	3,09	11,38	1	1					334	36,4	7,36
P61626	Lysozyme C OS=Homo sapiens OX=9606 GN=LYZ PE=1 SV=1 - [LYSC_HUMAN]	8,11	1	1	1	1	4,087E6	0,000E0	2,23	8,11	1	1		0,00			148	16,5	9,16
Q9BQG0	Myb-binding protein 1A OS=Homo sapiens OX=9606 GN=MYBBP1A PE=1 SV=2 - [MBB1A_HUMAN]	1,28	1	1	1	1	2,710E6	0,000E0	0,00	1,28	1	1		0,00			1328	148,8	9,28
O15116	U6 snRNA-associated Sm-like protein LSm1 OS=Homo sapiens OX=9606 GN=LSM1 PE=1 SV=1 - [LSM1	14,29	1	1	1	1	9,554E5	0,000E0	0,00	14,29	1	1					133	15,2	5,22
Q9UK45	U6 snRNA-associated Sm-like protein LSm7 OS=Homo sapiens OX=9606 GN=LSM7 PE=1 SV=1 - [LSM7	24,27	1	1	1	1	1,470E7	0,000E0	3,96	24,27	1	1		0,00			103	11,6	5,27
O95777	U6 snRNA-associated Sm-like protein LSm8 OS=Homo sapiens OX=9606 GN=LSM8 PE=1 SV=3 - [LSM8	16,67	1	1	1	1	4,066E6	0,000E0	0,00	16,67	1	1					96	10,4	4,48
P49736	DNA replication licensing factor MCM2 OS=Homo sapiens OX=9606 GN=MCM2 PE=1 SV=4 - [MCM2_H	4,98	1	3	3	3	3,455E6	0,000E0	0,00	4,98	3	3		0,00			904	101,8	5,52
Q10713	Mitochondrial-processing peptidase subunit alpha OS=Homo sapiens OX=9606 GN=PMPCA PE=1 SV=2 -	3,24	1	1	1	1	7,003E5	0,000E0	3,70	3,24	1	1		0,00			525	58,2	6,92
P26038	Moesin OS=Homo sapiens OX=9606 GN=MSN PE=1 SV=3 - [MOES_HUMAN]	5,55	1	2	2	2	1,979E6	0,000E0	2,53	5,55	2	2		0,00			577	67,8	6,40
Q7L9L4	MOB kinase activator 1B OS=Homo sapiens OX=9606 GN=MOB1B PE=1 SV=3 - [MOB1B_HUMAN]	10,19	2	1	1	1	0,000E0	0,000E0	3,10	10,19	1	1		0,00			216	25,1	6,73
P43246	DNA mismatch repair protein Msh2 OS=Homo sapiens OX=9606 GN=MSH2 PE=1 SV=1 - [MSH2_HUMA	2,68	1	2	2	2	1,567E6	0,000E0	2,22	2,68	2	2		0,00			934	104,7	5,77
O75439	Mitochondrial-processing peptidase subunit beta OS=Homo sapiens OX=9606 GN=PMPCB PE=1 SV=2 - [M	4,29	1	1	1	1	7,659E5	0,000E0	0,00	4,29	1	1		0,00			489	54,3	6,83
Q9BYG3	MKI67 FHA domain-interacting nucleolar phosphoprotein OS=Homo sapiens OX=9606 GN=NIFK PE=1 SV	7,85	1	1	1	1	0,000E0	0,000E0	0,00	7,85	1	1		0,00			293	34,2	9,88
Q9BXJ9	N-alpha-acetyltransferase 15, NatA auxiliary subunit OS=Homo sapiens OX=9606 GN=NAA15 PE=1 SV=1 -	1,96	1	1	1	1	7,219E5	0,000E0	0,00	1,96	1	1		0,00			866	101,2	7,42
P49321	Nuclear autoantigenic sperm protein OS=Homo sapiens OX=9606 GN=NASP PE=1 SV=2 - [NASP_HUMA	1,65	1	1	1	1	1,334E6	0,000E0	0,00	1,65	1	1		0,00			788	85,2	4,30
Q13126	S-methyl-5'-thioadenosine phosphorylase OS=Homo sapiens OX=9606 GN=MTAP PE=1 SV=2 - [MTAP_H	6,01	1	1	1	1	1,888E6	0,000E0	0,00	6,01	1	1		0,00			283	31,2	7,18
Q13765	Nascent polypeptide-associated complex subunit alpha OS=Homo sapiens OX=9606 GN=NACA PE=1 SV=1	13,49	2	2	2	2	9,055E6	0,000E0	2,71	13,49	2	2		0,00			215	23,4	4,56
Q8WU39	Marginal zone B- and B1-cell-specific protein OS=Homo sapiens OX=9606 GN=MZB1 PE=1 SV=1 - [MZB	6,88	1	1	1	1	2,665E6	0,000E0	2,05	6,88	1	1		0,00			189	20,7	5,57
Q9H0A0	RNA cytidine acetyltransferase OS=Homo sapiens OX=9606 GN=NAT10 PE=1 SV=2 - [NAT10_HUMAN]	3,51	1	2	2	2	2,553E6	0,000E0	2,06	3,51	2	2		0,00			1025	115,7	8,27
P16435	NADPH--cytochrome P450 reductase OS=Homo sapiens OX=9606 GN=POR PE=1 SV=2 - [NCPR_HUMA	3,84	1	1	1	1	9,204E6	0,000E0	4,02	3,84	1	1		0,00			677	76,6	5,58
Q01658	Protein Dr1 OS=Homo sapiens OX=9606 GN=DR1 PE=1 SV=1 - [NC2B_HUMAN]	13,64	1	1	1	1	2,079E6	0,000E0	2,12	13,64	1	1		0,00			176	19,4	4,75
P13995	Bifunctional methylenetetrahydrofolate dehydrogenase/cyclohydrolase, mitochondrial OS=Homo sapiens OX=	6,57	1	1	1	1	1,541E6	0,000E0	0,00	6,57	1	1					350	37,9	8,73
Q9UJ70	N-acetyl-D-glucosamine kinase OS=Homo sapiens OX=9606 GN=NAGK PE=1 SV=4 - [NAGK_HUMAN]	2,91	1	1	1	1	2,414E6	0,000E0	0,00	2,91	1	1		0,00			344	37,4	6,24
P43490	Nicotinamide phosphoribosyltransferase OS=Homo sapiens OX=9606 GN=NAMPT PE=1 SV=1 - [NAMPT_	2,04	1	1	1	1	1,624E6	0,000E0	0,00	2,04	1	1		0,00			491	55,5	7,15
P00387	NADH-cytochrome b5 reductase 3 OS=Homo sapiens OX=9606 GN=CYB5R3 PE=1 SV=3 - [NB5R3_HUM	3,99	1	1	1	2	1,100E6	0,000E0	0,00	3,99	1	2		0,00			301	34,2	7,59
P15531	Nucleoside diphosphate kinase A OS=Homo sapiens OX=9606 GN=NME1 PE=1 SV=1 - [NDKA_HUMAN]	38,16	1	2	4	4	3,302E6	0,000E0	4,41	38,16	4	4		0,00			152	17,1	6,19

P22392	Nucleoside diphosphate kinase B OS=Homo sapiens OX=9606 GN=NME2 PE=1 SV=1 - [NDKB_HUMAN]	38,82	2	2	4	5	4,124E7	0,000E0	7,30	38,82	4	5		0,00			152	17,3	8,41
Q15843	NEDD8 OS=Homo sapiens OX=9606 GN=NEDD8 PE=1 SV=1 - [NEDD8_HUMAN]	13,58	1	1	1	1	3,299E6	0,000E0	1,77	13,58	1	1					81	9,1	8,43
Q8WX92	Negative elongation factor B OS=Homo sapiens OX=9606 GN=NELFB PE=1 SV=1 - [NELFB_HUMAN]	5,17	1	1	1	1	1,338E6	0,000E0	0,00	5,17	1	1		0,00			580	65,7	6,13
Q8TCD5	5'(3')-deoxyribonucleotidase, cytosolic type OS=Homo sapiens OX=9606 GN=NT5C PE=1 SV=2 - [NT5C_H	8,96	1	1	1	1	4,666E5	0,000E0	3,05	8,96	1	1		0,00			201	23,4	6,64
Q13952	Nuclear transcription factor Y subunit gamma OS=Homo sapiens OX=9606 GN=NFYC PE=1 SV=3 - [NFY(	2,84	1	1	1	1	1,019E6	0,000E0	2,84	2,84	1	1		0,00			458	50,3	6,10
Q96TA1	Niban-like protein 1 OS=Homo sapiens OX=9606 GN=FAM129B PE=1 SV=3 - [NIBL1_HUMAN]	2,95	1	2	2	2	1,176E6	0,000E0	3,67	2,95	2	2		0,00			746	84,1	6,19
P69849	Nodal modulator 3 OS=Homo sapiens OX=9606 GN=NOMO3 PE=3 SV=2 - [NOMO3_HUMAN]	1,47	3	1	1	1	1,159E6	0,000E0	0,00	1,47	1	1		0,00			1222	134,0	5,67
P80188	Neutrophil gelatinase-associated lipocalin OS=Homo sapiens OX=9606 GN=LCN2 PE=1 SV=2 - [NGAL_HU	7,58	1	1	1	1	1,621E7	0,000E0	2,93	7,58	1	1					198	22,6	8,91
Q9BZE4	Nucleolar GTP-binding protein 1 OS=Homo sapiens OX=9606 GN=GTPBP4 PE=1 SV=3 - [NOG1_HUMA	2,84	1	1	1	1	4,846E6	0,000E0	0,00	2,84	1	1		0,00			634	73,9	9,50
O60287	Nucleolar pre-ribosomal-associated protein 1 OS=Homo sapiens OX=9606 GN=URB1 PE=1 SV=4 - [NPA1H	0,75	1	1	1	1	5,141E5	0,000E0	1,68	0,75	1	1		0,00			2271	254,2	6,47
P15559	NAD(P)H dehydrogenase [quinone] 1 OS=Homo sapiens OX=9606 GN=NQO1 PE=1 SV=1 - [NQO1_HUMA	7,30	1	1	1	1	2,204E6	0,000E0	2,50	7,30	1	1		0,00			274	30,8	8,88
P16083	Ribosyldihydronicotinamide dehydrogenase [quinone] OS=Homo sapiens OX=9606 GN=NQO2 PE=1 SV=5	7,79	1	1	1	1	3,334E5	0,000E0	0,00	7,79	1	1		0,00			231	25,9	6,29
Q9UQ80	Proliferation-associated protein 2G4 OS=Homo sapiens OX=9606 GN=PA2G4 PE=1 SV=3 - [PA2G4_HUM	5,58	1	2	2	2	9,397E5	0,000E0	0,00	5,58	2	2		0,00			394	43,8	6,55
Q9BV86	N-terminal Xaa-Pro-Lys N-methyltransferase 1 OS=Homo sapiens OX=9606 GN=NTMT1 PE=1 SV=3 - [NT	7,17	1	1	1	1	2,437E6	0,000E0	0,00	7,17	1	1		0,00			223	25,4	5,52
Q9Y266	Nuclear migration protein nudC OS=Homo sapiens OX=9606 GN=NUDC PE=1 SV=1 - [NUDC_HUMAN]	4,53	1	1	1	1	1,440E6	0,000E0	0,00	4,53	1	1		0,00			331	38,2	5,38
P04181	Ornithine aminotransferase, mitochondrial OS=Homo sapiens OX=9606 GN=OAT PE=1 SV=1 - [OAT_HUM	4,10	1	1	1	1	1,061E6	0,000E0	0,00	4,10	1	1		0,00			439	48,5	7,03
Q02818	Nucleobindin-1 OS=Homo sapiens OX=9606 GN=NUCB1 PE=1 SV=4 - [NUCB1_HUMAN]	3,47	1	1	1	1	1,352E6	0,000E0	0,00	3,47	1	1		0,00			461	53,8	5,25
Q9UKK9	ADP-sugar pyrophosphatase OS=Homo sapiens OX=9606 GN=NUDT5 PE=1 SV=1 - [NUDT5_HUMAN]	12,79	1	2	2	2	4,047E6	0,000E0	3,48	12,79	2	2					219	24,3	4,94
P37198	Nuclear pore glycoprotein p62 OS=Homo sapiens OX=9606 GN=NUP62 PE=1 SV=3 - [NUP62_HUMAN]	2,68	1	1	1	1	7,564E5	0,000E0	1,67	2,68	1	1		0,00			522	53,2	5,31
Q9Y3B8	Oligoribonuclease, mitochondrial OS=Homo sapiens OX=9606 GN=REXO2 PE=1 SV=3 - [ORN_HUMAN]	7,59	1	1	1	1	0,000E0	0,000E0	2,07	7,59	1	1		0,00			237	26,8	6,87
P0C869	Cytosolic phospholipase A2 beta OS=Homo sapiens OX=9606 GN=PLA2G4B PE=1 SV=2 - [PA24B_HUM	1,66	1	1	1	1	1,466E6	0,000E0	0,00	1,66	1	1		0,00			781	87,9	5,96
Q8N7H5	RNA polymerase II-associated factor 1 homolog OS=Homo sapiens OX=9606 GN=PAF1 PE=1 SV=2 - [PAI	4,14	1	1	1	1	4,378E5	0,000E0	0,00	4,14	1	1		0,00			531	59,9	4,63
Q9NTK5	Obg-like ATPase 1 OS=Homo sapiens OX=9606 GN=OLA1 PE=1 SV=2 - [OLA1_HUMAN]	15,15	1	3	3	3	4,853E6	0,000E0	1,66	15,15	3	3		0,00			396	44,7	7,81
O60313	Dynamin-like 120 kDa protein, mitochondrial OS=Homo sapiens OX=9606 GN=OPA1 PE=1 SV=3 - [OPA1	2,92	1	1	1	1	3,370E6	0,000E0	4,31	2,92	1	1		0,00			960	111,6	7,87
Q96FW1	Ubiquitin thioesterase OTUB1 OS=Homo sapiens OX=9606 GN=OTUB1 PE=1 SV=2 - [OTUB1_HUMAN]	10,70	1	1	1	1	2,512E6	0,000E0	3,06	10,70	1	1		0,00			271	31,3	4,94
P11182	Lipoamide acyltransferase component of branched-chain alpha-keto acid dehydrogenase complex, mitochondria	1,87	1	1	1	1	6,596E5	0,000E0	0,00	1,87	1	1		0,00			482	53,5	8,51
P54886	Delta-1-pyrroline-5-carboxylate synthase OS=Homo sapiens OX=9606 GN=ALDH18A1 PE=1 SV=2 - [P5CS	3,02	1	1	1	1	3,320E6	0,000E0	4,28	3,02	1	1		0,00			795	87,2	7,12
P05120	Plasminogen activator inhibitor 2 OS=Homo sapiens OX=9606 GN=SERPINB2 PE=1 SV=2 - [PAI2_HUM	1,93	1	1	1	1	3,017E5	0,000E0	0,00	1,93	1	1		0,00			415	46,6	5,63
O96013	Serine/threonine-protein kinase PAK 4 OS=Homo sapiens OX=9606 GN=PAK4 PE=1 SV=1 - [PAK4_HUM	3,05	1	1	1	1	1,443E6	0,000E0	3,64	3,05	1	1		0,00			591	64,0	9,73
O95340	Bifunctional 3'-phosphoadenosine 5'-phosphosulfate synthase 2 OS=Homo sapiens OX=9606 GN=PAPSS2 P	2,93	1	1	1	1	1,153E6	0,000E0	2,81	2,93	1	1		0,00			614	69,5	8,03
O00151	PDZ and LIM domain protein 1 OS=Homo sapiens OX=9606 GN=PDLIM1 PE=1 SV=4 - [PDLI1_HUMAN	5,17	1	1	1	1	1,143E6	0,000E0	0,00	5,17	1	1		0,00			329	36,0	7,02
O00764	Pyridoxal kinase OS=Homo sapiens OX=9606 GN=PDXK PE=1 SV=1 - [PDXK_HUMAN]	4,49	1	1	1	1	8,008E5	0,000E0	2,81	4,49	1	1		0,00			312	35,1	6,13
Q15645	Pachytene checkpoint protein 2 homolog OS=Homo sapiens OX=9606 GN=TRIP13 PE=1 SV=2 - [PCH2_H	4,17	1	1	1	1	7,859E5	0,000E0	4,21	4,17	1	1		0,00			432	48,5	6,09
P12004	Proliferating cell nuclear antigen OS=Homo sapiens OX=9606 GN=PCNA PE=1 SV=1 - [PCNA_HUMAN]	7,28	1	1	1	1	0,000E0	0,000E0	3,85	7,28	1	1		0,00			261	28,8	4,69
Q9UHG3	Prenylcysteine oxidase 1 OS=Homo sapiens OX=9606 GN=PCYOX1 PE=1 SV=3 - [PCYOX_HUMAN]	2,97	1	1	1	1	2,653E6	0,000E0	1,83	2,97	1	1		0,00			505	56,6	6,18
P30086	Phosphatidylethanolamine-binding protein 1 OS=Homo sapiens OX=9606 GN=PEBP1 PE=1 SV=3 - [PEBP	25,67	1	3	3	3	5,825E6	0,000E0	5,36	25,67	3	3		0,00			187	21,0	7,53
O15212	Prefoldin subunit 6 OS=Homo sapiens OX=9606 GN=PFDN6 PE=1 SV=1 - [PFD6_HUMAN]	10,85	1	1	1	1	8,288E6	0,000E0	1,97	10,85	1	1		0,00			129	14,6	8,88
O60437	Periplakin OS=Homo sapiens OX=9606 GN=PPL PE=1 SV=4 - [PEPL_HUMAN]	7,29	1	13	13	14	1,123E7	0,000E0	14,34	7,29	13	14		0,00			1756	204,6	5,60
P61758	Prefoldin subunit 3 OS=Homo sapiens OX=9606 GN=VBP1 PE=1 SV=3 - [PFD3_HUMAN]	9,64	1	1	1	1	2,709E5	0,000E0	0,00	9,64	1	1		0,00			197	22,6	7,11
Q92696	Geranylgeranyl transferase type-2 subunit alpha OS=Homo sapiens OX=9606 GN=RABGGTA PE=1 SV=2 -	4,06	1	1	1	1	0,000E0	0,000E0	0,00	4,06	1	1		0,00			567	65,0	5,67
Q8WVV4	Protein POF1B OS=Homo sapiens OX=9606 GN=POF1B PE=1 SV=3 - [POF1B_HUMAN]	10,36	1	6	6	6	2,754E7	0,000E0	9,67	10,36	6	6		0,00			589	68,0	6,32
O60828	Polyglutamine-binding protein 1 OS=Homo sapiens OX=9606 GN=PQBP1 PE=1 SV=1 - [PQBP1_HUMAN	9,81	1	1	1	1	2,174E5	0,000E0	0,00	9,81	1	1		0,00			265	30,5	6,33
P43686	26S proteasome regulatory subunit 6B OS=Homo sapiens OX=9606 GN=PSMC4 PE=1 SV=2 - [PRS6B_H	4,55	1	2	2	2	6,701E6	0,000E0	3,53	4,55	2	2		0,00			418	47,3	5,21
Q15435	Protein phosphatase 1 regulatory subunit 7 OS=Homo sapiens OX=9606 GN=PPP1R7 PE=1 SV=1 - [PP1R	11,11	1	2	2	2	9,976E5	0,000E0	2,03	11,11	2	2		0,00			360	41,5	4,91
P23284	Peptidyl-prolyl cis-trans isomerase B OS=Homo sapiens OX=9606 GN=PPIB PE=1 SV=2 - [PPIB_HUMAN	11,11	1	2	2	3	3,470E6	0,000E0	0,00	11,11	2	3		0,00			216	23,7	9,41
Q13835	Plakophilin-1 OS=Homo sapiens OX=9606 GN=PKP1 PE=1 SV=2 - [PKP1_HUMAN]	3,88	1	2	2	2	5,173E6	0,000E0	3,87	3,88	2	2		0,00			747	82,8	9,13
P14923	Junction plakoglobin OS=Homo sapiens OX=9606 GN=JUP PE=1 SV=3 - [PLAK_HUMAN]	13,42	1	8	8	11	3,488E7	0,000E0	24,16	13,42	8	11		0,00			745	81,7	6,14
O60664	Perilipin-3 OS=Homo sapiens OX=9606 GN=PLIN3 PE=1 SV=3 - [PLIN3_HUMAN]	6,91	1	1	1	1	7,553E6	0,000E0	3,57	6,91	1	1					434	47,0	5,44
Q99623	Prohibitin-2 OS=Homo sapiens OX=9606 GN=PHB2 PE=1 SV=2 - [PHB2_HUMAN]	12,04	1	2	2	2	6,140E6	0,000E0	1,92	12,04	2	2		0,00			299	33,3	9,83
Q13526	Peptidyl-prolyl cis-trans isomerase NIMA-interacting 1 OS=Homo sapiens OX=9606 GN=PIN1 PE=1 SV=1	11,66	1	1	1	1	4,600E5	0,000E0	0,00	11,66	1	1					163	18,2	8,82
Q9NRX1	RNA-binding protein PNO1 OS=Homo sapiens OX=9606 GN=PNO1 PE=1 SV=1 - [PNO1_HUMAN]	7,14	1	1	1	1	3,021E5	0,000E0	0,00	7,14	1	1		0,00			252	27,9	9,73
Q9NVS9	Pyridoxine-5'-phosphate oxidase OS=Homo sapiens OX=9606 GN=PNPO PE=1 SV=1 - [PNPO_HUMAN]	7,28	1	1	1	1	1,821E6	0,000E0	2,83	7,28	1	1		0,00			261	30,0	7,06
Q96C90	Protein phosphatase 1 regulatory subunit 14B OS=Homo sapiens OX=9606 GN=PPP1R14B PE=1 SV=3 - [	8,84	1	1	1	1	2,269E6	0,000E0	3,07	8,84	1	1					147	15,9	4,86
P18669	Phosphoglycerate mutase 1 OS=Homo sapiens OX=9606 GN=PGAM1 PE=1 SV=2 - [PGAM1_HUMAN]	4,33	1	1	1	1	2,243E6	0,000E0	0,00	4,33	1	1		0,00			254	28,8	7,18
Q96G03	Phosphoglucomutase-2 OS=Homo sapiens OX=9606 GN=PGM2 PE=1 SV=4 - [PGM2_HUMAN]	5,56	1	3	3	3	8,191E6	0,000E0	0,00	5,56	3	3		0,00			612	68,2	6,73
Q13492	Phosphatidylinositol-binding clathrin assembly protein OS=Homo sapiens OX=9606 GN=PICALM PE=1 SV	5,52	1	1	1	1	2,622E5	0,000E0	3,13	5,52	1	1		0,00			652	70,7	7,90
P08567	Pleckstrin OS=Homo sapiens OX=9606 GN=PLEK PE=1 SV=3 - [PLEK_HUMAN]	11,43	1	2	2	2	2,996E6	0,000E0	5,51	11,43	2	2		0,00			350	40,1	8,28
P00491	Purine nucleoside phosphorylase OS=Homo sapiens OX=9606 GN=PNP PE=1 SV=2 - [PNPH_HUMAN]	9,34	1	2	2	2	1,877E6	0,000E0	3,10	9,34	2	2		0,00			289	32,1	6,95
Q5H9R7	Serine/threonine-protein phosphatase 6 regulatory subunit 3 OS=Homo sapiens OX=9606 GN=PPP6R3 PE=	1,60	1	1	1	1	4,203E6	0,000E0	1,98	1,60	1	1		0,00			873	97,6	4,60
P15309	Prostatic acid phosphatase OS=Homo sapiens OX=9606 GN=ACPP PE=1 SV=3 - [PPAP_HUMAN]	3,11	1	1	1	1	6,910E6	0,000E0	2,88	3,11	1	1		0,00			386	44,5	6,24
P30044	Peroxiredoxin-5, mitochondrial OS=Homo sapiens OX=9606 GN=PRDX5 PE=1 SV=4 - [PRDX5_HUMAN	7,94	1	1	1	1	1,233E7	0,000E0	3,75	7,94	1	1		0,00			214	22,1	8,70
P30041	Peroxiredoxin-6 OS=Homo sapiens OX=9606 GN=PRDX6 PE=1 SV=3 - [PRDX6_HUMAN]	14,29	1	3	3	3	9,061E6	0,000E0	0,00	14,29	3	3					224	25,0	6,38
Q9UNP9	Peptidyl-prolyl cis-trans isomerase E OS=Homo sapiens OX=9606 GN=PPIE PE=1 SV=1 - [PPIE_HUMAN	8,31	1	1	1	1	3,635E5	0,000E0	0,00	8,31	1	1		0,00			301	33,4	5,60
O94906	Pre-mRNA-processing factor 6 OS=Homo sapiens OX=9606 GN=PRPF6 PE=1 SV=1 - [PRP6_HUMAN]	2,02	1	1	1	1	4,611E5	0,000E0	0,00	2,02	1	1		0,00			941	106,9	8,25
P11908	Ribose-phosphate pyrophosphokinase 2 OS=Homo sapiens OX=9606 GN=PRPS2 PE=1 SV=2 - [PRPS2_H	4,72	2	1	1	1	1,280E6	0,000E0	3,74	4,72	1	1		0,00			318	34,7	6,61
P25786	Proteasome subunit alpha type-1 OS=Homo sapiens OX=9606 GN=PSMA1 PE=1 SV=1 - [PSA1_HUMAN]	19,39	1	3	3	3	1,551E7	0,000E0	4,57	19,39	3	3		0,00			263	29,5	6,61
P25787	Proteasome subunit alpha type-2 OS=Homo sapiens OX=9606 GN=PSMA2 PE=1 SV=2 - [PSA2_HUMAN]	10,68	1	2	2	2	2,150E6	0,000E0	1,90	10,68	2	2		0,00			234	25,9	7,43
P55786	Puromycin-sensitive aminopeptidase OS=Homo sapiens OX=9606 GN=NPEPPS PE=1 SV=2 - [PSA_HUM	3,26	2	3	3	3	1,384E6	0,000E0	0,00	3,26	3	3		0,00			919	103,2	5,72
P55036	26S proteasome non-ATPase regulatory subunit 4 OS=Homo sapiens OX=9606 GN=PSMD4 PE=1 SV=1 - [	8,49	2	1	1	1	9,061E6	0,000E0	5,08	8,49	1	1		0,00			377	40,7	4,79
Q15257	Serine/threonine-protein phosphatase 2A activator OS=Homo sapiens OX=9606 GN=PTPA PE=1 SV=3 - [P	8,10	1	1	1	1	1,015E7	0,000E0	2,85	8,10	1	1		0,00			358	40,6	5,94
Q15397	Pumilio homolog 3 OS=Homo sapiens OX=9606 GN=PUM3 PE=1 SV=3 - [PUM3_HUMAN]	2,78	1	1	1	1	1,200E6	0,000E0	2,19	2,78	1	1		0,00			648	73,5	9,64
P22234	Multifunctional protein ADE2 OS=Homo sapiens OX=9606 GN=PAICS PE=1 SV=3 - [PUR6_HUMAN]	7,53	1	2	2	2	1,186E7	0,000E0	2,14	7,53	2	2					425	47,0	7,23
P51665	26S proteasome non-ATPase regulatory subunit 7 OS=Homo sapiens OX=9606 GN=PSMD7 PE=1 SV=2 - [	10,80	1	2	2	2	3,410E6	0,000E0	1,70	10,80	2	2		0,00			324	37,0	6,77
Q06323	Proteasome activator complex subunit 1 OS=Homo sapiens OX=9606 GN=PSME1 PE=1 SV=1 - [PSME1_	5,22	1	1	1	1	1,999E6	0,000E0	0,00	5,22	1	1		0,00			249	28,7	6,02
Q96BW5	Phosphotriesterase-related protein OS=Homo sapiens OX=9606 GN=PTER PE=1 SV=1 - [PTER_HUMAN]	7,16	1	1	1	1	2,082E6	0,000E0	3,07	7,16	1	1		0,00			349	39,0	6,52
P29350	Tyrosine-protein phosphatase non-receptor type 6 OS=Homo sapiens OX=9606 GN=PTPN6 PE=1 SV=1 - [P	3,53	1	1	1	1	4,604E5	0,000E0	0,00	3,53	1	1		0,00			595	67,5	7,78
P22102	Trifunctional purine biosynthetic protein adenosine-3 OS=Homo sapiens OX=9606 GN=GART PE=1 SV=1 -	4,46	1	2	2	2	1,176E6	0,000E0	0,00	4,46	2	2		0,00			1010	107,7	6,70
P30520	Adenylosuccinate synthetase isozyme 2 OS=Homo sapiens OX=9606 GN=ADSS PE=1 SV=3 - [PURA2_HU	2,41	1	1	1	1	1,303E6	0,000E0	1,65	2,41	1	1		0,00			456	50,1	6,55
O00487	26S proteasome non-ATPase regulatory subunit 14 OS=Homo sapiens OX=9606 GN=PSMD14 PE=1 SV=1	11,29	1	2	2	2	2,586E6	0,000E0	5,95	11,29	2	2		0,00			310	34,6	6,52
Q16401	26S proteasome non-ATPase regulatory subunit 5 OS=Homo sapiens OX=9606 GN=PSMD5 PE=1 SV=3 - [	4,17	1	1	1	1	9,567E5	0,000E0	3,47	4,17	1	1		0,00			504	56,2	5,48
P78527	DNA-dependent protein kinase catalytic subunit OS=Homo sapiens OX=9606 GN=PRKDC PE=1 SV=3 - [P	4,63	1	11	11	11	5,745E6	0,000E0	24,52	4,63	11	11		0,00			4128	468,8	7,12
P25788	Proteasome subunit alpha type-3 OS=Homo sapiens OX=9606 GN=PSMA3 PE=1 SV=2 - [PSA3_HUMAN]	4,71	1	1	1	1	6,893E6	0,000E0	2,61	4,71	1	1		0,00			255	28,4	5,33
P49721	Proteasome subunit beta type-2 OS=Homo sapiens OX=9606 GN=PSMB2 PE=1 SV=1 - [PSB2_HUMAN]	7,46	1	1	1	1	2,254E6	0,000E0	0,00	7,46	1	1		0,00			201	22,8	7,02
Q9UL46	Proteasome activator complex subunit 2 OS=Homo sapiens OX=9606 GN=PSME2 PE=1 SV=4 - [PSME2_	14,64	1	2	2	2	7,343E6	0,000E0	4,26	14,64	2	2		0,00			239	27,4	5,73
P61289	Proteasome activator complex subunit 3 OS=Homo sapiens OX=9606 GN=PSME3 PE=1 SV=1 - [PSME3_	4,33	1	1	1	1	1,095E6	0,000E0	0,00	4,33	1	1		0,00			254	29,5	5,95
P28066	Proteasome subunit alpha type-5 OS=Homo sapiens OX=9606 GN=PSMA5 PE=1 SV=3 - [PSA5_HUMAN]	7,88	1	1	1	1	2,710E6	0,000E0	0,00	7,88	1	1		0,00			241	26,4	4,79
O14818	Proteasome subunit alpha type-7 OS=Homo sapiens OX=9606 GN=PSMA7 PE=1 SV=1 - [PSA7_HUMAN]	13,71	2	3	3	3	4,793E6	0,000E0	5,98	13,71	3	3		0,00			248	27,9	8,46
P20618	Proteasome subunit beta type-1 OS=Homo sapiens OX=9606 GN=PSMB1 PE=1 SV=2 - [PSB1_HUMAN]	5,81	1	1	1	1	0,000E0	0,000E0	0,00	5,81	1	1		0,00			241	26,5	8,13
P49720	Proteasome subunit beta type-3 OS=Homo sapiens OX=9606 GN=PSMB3 PE=1 SV=2 - [PSB3_HUMAN]	8,78	1	1	1	1	2,243E6	0,000E0	1,66	8,78	1	1		0,00			205	22,9	6,55
P28074	Proteasome subunit beta type-5 OS=Homo sapiens OX=9606 GN=PSMB5 PE=1 SV=3 - [PSB5_HUMAN]	9,89	1	2	2	2	5,048E6	0,000E0	5,13	9,89	2	2		0,00			263	28,5	6,92
P28072	Proteasome subunit beta type-6 OS=Homo sapiens OX=9606 GN=PSMB6 PE=1 SV=4 - [PSB6_HUMAN]	4,60	1	1	1	1	3,670E6	0,000E0	2,25	4,60	1	1		0,00			239	25,3	4,92
P07737	Profilin-1 OS=Homo sapiens OX=9606 GN=PFN1 PE=1 SV=2 - [PROF1_HUMAN]	31,43	1	3	3	3	2,874E7	0,000E0	4,81	31,43	3	3					140	15,0	8,27
P62195	26S proteasome regulatory subunit 8 OS=Homo sapiens OX=9606 GN=PSMC5 PE=1 SV=1 - [PRS8_HUM	7,39	1	2	2	2	1,602E6	0,000E0	2,64	7,39	2	2		0,00			406	45,6	7,55
P60900	Proteasome subunit alpha type-6 OS=Homo sapiens OX=9606 GN=PSMA6 PE=1 SV=1 - [PSA6_HUMAN]	4,07	1	1	1	1	5,265E5	0,000E0	0,00	4,07	1	1		0,00			246	27,4	6,76
O15355	Protein phosphatase 1G OS=Homo sapiens OX=9606 GN=PPM1G PE=1 SV=1 - [PPM1G_HUMAN]	3,11	1	1	1	1	2,440E7	0,000E0	3,45	3,11	1	1		0,00			546	59,2	4,36
Q6P2Q9	Pre-mRNA-processing-splicing factor 8 OS=Homo sapiens OX=9606 GN=PRPF8 PE=1 SV=2 - [PRP8_HU	3,34	1	4	4	4	2,273E6	0,000E0	8,38	3,34	4	4		0,00			2335	273,4	8,84
O00232	26S proteasome non-ATPase regulatory subunit 12 OS=Homo sapiens OX=9606 GN=PSMD12 PE=1 SV=3	5,04	1	1	1	1	2,165E6	0,000E0	4,11	5,04	1	1		0,00			456	52,9	7,65
Q9UNM6	26S proteasome non-ATPase regulatory subunit 13 OS=Homo sapiens OX=9606 GN=PSMD13 PE=1 SV=2	4,52	1	1	1	1	1,104E6	0,000E0	0,00	4,52	1	1		0,00			376	42,9	5,81
Q15008	26S proteasome non-ATPase regulatory subunit 6 OS=Homo sapiens OX=9606 GN=PSMD6 PE=1 SV=1 - [	6,17	1	1	1	1	7,871E5	0,000E0	2,74	6,17	1	1		0,00			389	45,5	5,62
P31939	Bifunctional purine biosynthesis protein PURH OS=Homo sapiens OX=9606 GN=ATIC PE=1 SV=3 - [PUR	2,53	1	1	1	1	8,223E6	0,000E0	1,94	2,53	1	1		0,00			592	64,6	6,71
P51149	Ras-related protein Rab-7a OS=Homo sapiens OX=9606 GN=RAB7A PE=1 SV=1 - [RAB7A_HUMAN]	20,29	1	3	3	3	2,388E6	0,000E0	2,66	20,29	3	3		0,00			207	23,5	6,70
P29373	Cellular retinoic acid-binding protein 2 OS=Homo sapiens OX=9606 GN=CRABP2 PE=1 SV=2 - [RABP2_	6,52	1	1	1	1	9,539E6	0,000E0	1,62	6,52	1	1					138	15,7	5,40
O60216	Double-strand-break repair protein rad21 homolog OS=Homo sapiens OX=9606 GN=RAD21 PE=1 SV=2 - [	8,56	1	2	2	2	4,435E6	0,000E0	0,00	8,56	2	2		0,00			631	71,6	4,65
Q9Y3L5	Ras-related protein Rap-2c OS=Homo sapiens OX=9606 GN=RAP2C PE=1 SV=1 - [RAP2C_HUMAN]	6,56	1	1	1	1	1,265E6	0,000E0	0,00	6,56	1	1		0,00			183	20,7	4,94
P40937	Replication factor C subunit 5 OS=Homo sapiens OX=9606 GN=RFC5 PE=1 SV=1 - [RFC5_HUMAN]	5,88	1	1	1	1	1,015E6	0,000E0	3,45	5,88	1	1		0,00			340	38,5	7,20
Q07960	Rho GTPase-activating protein 1 OS=Homo sapiens OX=9606 GN=ARHGAP1 PE=1 SV=1 - [RHG01_HUM	8,66	1	2	2	2	3,513E6	0,000E0	4,91	8,66	2	2		0,00			439	50,4	6,29
P61586	Transforming protein RhoA OS=Homo sapiens OX=9606 GN=RHOA PE=1 SV=1 - [RHOA_HUMAN]	12,44	1	1	1	1	7,352E6	0,000E0	3,40	12,44	1	1		0,00			193	21,8	6,10
P08134	Rho-related GTP-binding protein RhoC OS=Homo sapiens OX=9606 GN=RHOC PE=1 SV=1 - [RHOC_HU	12,44	1	1	1	1	2,718E6	0,000E0	4,55	12,44	1	1		0,00			193	22,0	6,58
P13489	Ribonuclease inhibitor OS=Homo sapiens OX=9606 GN=RNH1 PE=1 SV=2 - [RINI_HUMAN]	3,25	1	1	1	1	1,790E6	0,000E0	2,12	3,25	1	1		0,00			461	49,9	4,82
P54727	UV excision repair protein RAD23 homolog B OS=Homo sapiens OX=9606 GN=RAD23B PE=1 SV=1 - [R	5,38	1	2	2	2	2,996E6	0,000E0	0,00	5,38	2	2		0,00			409	43,1	4,84
P35241	Radixin OS=Homo sapiens OX=9606 GN=RDX PE=1 SV=1 - [RADI_HUMAN]	5,15	1	2	2	2	3,580E6	0,000E0	2,34	5,15	2	2		0,00			583	68,5	6,37
P61225	Ras-related protein Rap-2b OS=Homo sapiens OX=9606 GN=RAP2B PE=1 SV=1 - [RAP2B_HUMAN]	6,56	1	1	1	1	7,569E5	0,000E0	1,64	6,56	1	1		0,00			183	20,5	4,81
Q09028	Histone-binding protein RBBP4 OS=Homo sapiens OX=9606 GN=RBBP4 PE=1 SV=3 - [RBBP4_HUMAN	5,88	1	1	2	2	1,438E7	0,000E0	2,72	5,88	2	2		0,00			425	47,6	4,89
P98179	RNA-binding protein 3 OS=Homo sapiens OX=9606 GN=RBM3 PE=1 SV=1 - [RBM3_HUMAN]	11,46	1	1	1	2	8,060E5	0,000E0	0,00	11,46	1	2		0,00			157	17,2	8,91
P06400	Retinoblastoma-associated protein OS=Homo sapiens OX=9606 GN=RB1 PE=1 SV=2 - [RB_HUMAN]	2,59	1	1	1	1	1,473E6	0,000E0	3,54	2,59	1	1		0,00			928	106,1	7,94
Q5UIP0	Telomere-associated protein RIF1 OS=Homo sapiens OX=9606 GN=RIF1 PE=1 SV=2 - [RIF1_HUMAN]	0,69	1	1	1	1	7,933E5	0,000E0	2,37	0,69	1	1		0,00			2472	274,3	5,52
O76021	Ribosomal L1 domain-containing protein 1 OS=Homo sapiens OX=9606 GN=RSL1D1 PE=1 SV=3 - [RL1I	2,65	1	1	1	1	2,760E6	0,000E0	2,34	2,65	1	1		0,00			490	54,9	10,13
P46779	60S ribosomal protein L28 OS=Homo sapiens OX=9606 GN=RPL28 PE=1 SV=3 - [RL28_HUMAN]	8,03	1	1	1	1	5,958E5	0,000E0	1,93	8,03	1	1					137	15,7	12,02
P05386	60S acidic ribosomal protein P1 OS=Homo sapiens OX=9606 GN=RPLP1 PE=1 SV=1 - [RLA1_HUMAN]	14,04	1	1	1	1	3,834E6	0,000E0	0,00	14,04	1	1					114	11,5	4,32
Q99729	Heterogeneous nuclear ribonucleoprotein A/B OS=Homo sapiens OX=9606 GN=HNRNPAB PE=1 SV=2 - [I	5,72	1	1	1	1	2,728E7	0,000E0	0,00	5,72	1	1		0,00			332	36,2	8,21
P05387	60S acidic ribosomal protein P2 OS=Homo sapiens OX=9606 GN=RPLP2 PE=1 SV=1 - [RLA2_HUMAN]	10,43	1	1	1	1	1,036E7	0,000E0	2,67	10,43	1	1		0,00			115	11,7	4,54
Q96E39	RNA binding motif protein, X-linked-like-1 OS=Homo sapiens OX=9606 GN=RBMXL1 PE=1 SV=1 - [RM	3,33	2	1	1	1	3,231E6	0,000E0	1,67	3,33	1	1		0,00			390	42,1	9,89
Q9H1E1	Ribonuclease 7 OS=Homo sapiens OX=9606 GN=RNASE7 PE=1 SV=2 - [RNAS7_HUMAN]	8,97	1	1	1	1	1,958E6	0,000E0	0,00	8,97	1	1					156	17,4	9,69
Q9BYD2	39S ribosomal protein L9, mitochondrial OS=Homo sapiens OX=9606 GN=MRPL9 PE=1 SV=2 - [RM09_I	6,37	1	1	1	1	1,087E6	0,000E0	1,63	6,37	1	1		0,00			267	30,2	10,08
Q9H7B2	Ribosome production factor 2 homolog OS=Homo sapiens OX=9606 GN=RPF2 PE=1 SV=2 - [RPF2_HUM	4,90	1	1	1	1	5,057E5	0,000E0	0,00	4,90	1	1		0,00			306	35,6	9,99
Q9Y508	E3 ubiquitin-protein ligase RNF114 OS=Homo sapiens OX=9606 GN=RNF114 PE=1 SV=1 - [RN114_HUM	10,09	1	1	1	1	0,000E0	0,000E0	0,00	10,09	1	1		0,00			228	25,7	7,25
A6NIZ1	Ras-related protein Rap-1b-like protein OS=Homo sapiens OX=9606 PE=2 SV=1 - [RP1BL_HUMAN]	6,52	3	1	1	1	8,906E6	0,000E0	1,68	6,52	1	1		0,00			184	20,9	5,48
Q96E11	Ribosome-recycling factor, mitochondrial OS=Homo sapiens OX=9606 GN=MRRF PE=1 SV=1 - [RRFM_I	8,40	1	1	1	1	1,283E6	0,000E0	2,85	8,40	1	1					262	29,3	9,79
Q9HCY8	Protein S100-A14 OS=Homo sapiens OX=9606 GN=S100A14 PE=1 SV=1 - [S10AE_HUMAN]	6,73	1	1	1	1	4,282E6	0,000E0	0,00	6,73	1	1		0,00			104	11,7	5,24
Q96FQ6	Protein S100-A16 OS=Homo sapiens OX=9606 GN=S100A16 PE=1 SV=1 - [S10AG_HUMAN]	11,65	1	1	1	1	3,503E6	0,000E0	0,00	11,65	1	1					103	11,8	6,79
Q9UBT2	SUMO-activating enzyme subunit 2 OS=Homo sapiens OX=9606 GN=UBA2 PE=1 SV=2 - [SAE2_HUMAN	7,66	1	3	3	3	5,691E6	0,000E0	8,64	7,66	3	3		0,00			640	71,2	5,29
Q9NR31	GTP-binding protein SAR1a OS=Homo sapiens OX=9606 GN=SAR1A PE=1 SV=1 - [SAR1A_HUMAN]	11,62	1	1	1	1	5,384E6	0,000E0	3,01	11,62	1	1		0,00			198	22,4	6,68
Q6UWP8	Suprabasin OS=Homo sapiens OX=9606 GN=SBSN PE=1 SV=2 - [SBSN_HUMAN]	32,54	1	9	9	13	6,048E7	0,000E0	42,11	32,54	9	13		0,00			590	60,5	7,01
Q9H190	Syntenin-2 OS=Homo sapiens OX=9606 GN=SDCBP2 PE=1 SV=2 - [SDCB2_HUMAN]	3,77	1	1	1	1	1,040E6	0,000E0	0,00	3,77	1	1		0,00			292	31,6	9,07
Q15393	Splicing factor 3B subunit 3 OS=Homo sapiens OX=9606 GN=SF3B3 PE=1 SV=4 - [SF3B3_HUMAN]	4,52	1	2	2	2	2,220E6	0,000E0	2,43	4,52	2	2		0,00			1217	135,5	5,26
Q99584	Protein S100-A13 OS=Homo sapiens OX=9606 GN=S100A13 PE=1 SV=1 - [S10AD_HUMAN]	17,35	1	2	2	2	6,431E6	0,000E0	1,78	17,35	2	2					98	11,5	6,16
P02743	Serum amyloid P-component OS=Homo sapiens OX=9606 GN=APCS PE=1 SV=2 - [SAMP_HUMAN]	2,69	1	1	1	1	9,313E6	0,000E0	0,00	2,69	1	1					223	25,4	6,54
O75396	Vesicle-trafficking protein SEC22b OS=Homo sapiens OX=9606 GN=SEC22B PE=1 SV=4 - [SC22B_HUM	5,58	1	1	1	1	1,649E6	0,000E0	0,00	5,58	1	1		0,00			215	24,6	6,92
Q8IZC4	Rhotekin-2 OS=Homo sapiens OX=9606 GN=RTKN2 PE=2 SV=1 - [RTKN2_HUMAN]	1,15	1	1	1	1	3,538E6	0,000E0	0,00	1,15	1	1		0,00			609	69,3	7,83
Q9Y224	RNA transcription, translation and transport factor protein OS=Homo sapiens OX=9606 GN=RTRAF PE=1 S	5,33	1	1	1	1	3,336E5	0,000E0	0,00	5,33	1	1		0,00			244	28,1	6,65
Q6NUK1	Calcium-binding mitochondrial carrier protein SCaMC-1 OS=Homo sapiens OX=9606 GN=SLC25A24 PE=	3,35	1	1	1	1	5,957E6	0,000E0	3,99	3,35	1	1		0,00			477	53,3	6,33
P09661	U2 small nuclear ribonucleoprotein A' OS=Homo sapiens OX=9606 GN=SNRPA1 PE=1 SV=2 - [RU2A_HU	9,80	1	1	1	1	3,624E6	0,000E0	3,26	9,80	1	1		0,00			255	28,4	8,62
A8MWD9	Putative small nuclear ribonucleoprotein G-like protein 15 OS=Homo sapiens OX=9606 GN=SNRPGP15 PE	15,79	2	1	1	2	6,693E6	0,000E0	2,96	15,79	1	2					76	8,5	8,84
P23526	Adenosylhomocysteinase OS=Homo sapiens OX=9606 GN=AHCY PE=1 SV=4 - [SAHH_HUMAN]	5,32	1	2	2	2	1,185E6	0,000E0	2,13	5,32	2	2		0,00			432	47,7	6,34
P07602	Prosaposin OS=Homo sapiens OX=9606 GN=PSAP PE=1 SV=2 - [SAP_HUMAN]	5,73	1	3	3	3	4,580E6	0,000E0	3,05	5,73	3	3		0,00			524	58,1	5,17
P25815	Protein S100-P OS=Homo sapiens OX=9606 GN=S100P PE=1 SV=2 - [S100P_HUMAN]	28,42	1	2	2	2	4,554E6	0,000E0	3,42	28,42	2	2					95	10,4	4,88
P29034	Protein S100-A2 OS=Homo sapiens OX=9606 GN=S100A2 PE=1 SV=3 - [S10A2_HUMAN]	9,18	1	1	1	1	4,273E6	0,000E0	1,87	9,18	1	1		0,00			98	11,1	4,78
P31949	Protein S100-A11 OS=Homo sapiens OX=9606 GN=S100A11 PE=1 SV=2 - [S10AB_HUMAN]	23,81	1	2	2	4	5,290E7	0,000E0	0,00	23,81	2	4		0,00			105	11,7	7,12
P17900	Ganglioside GM2 activator OS=Homo sapiens OX=9606 GN=GM2A PE=1 SV=4 - [SAP3_HUMAN]	5,18	1	1	1	1	1,785E7	0,000E0	2,76	5,18	1	1		0,00			193	20,8	5,31
Q9NQZ2	Something about silencing protein 10 OS=Homo sapiens OX=9606 GN=UTP3 PE=1 SV=1 - [SAS10_HUM	3,97	1	1	1	1	1,617E6	0,000E0	1,78	3,97	1	1		0,00			479	54,5	5,62
O00560	Syntenin-1 OS=Homo sapiens OX=9606 GN=SDCBP PE=1 SV=1 - [SDCB1_HUMAN]	6,38	1	1	1	1	7,433E5	0,000E0	0,00	6,38	1	1		0,00			298	32,4	7,53
Q9UGP8	Translocation protein SEC63 homolog OS=Homo sapiens OX=9606 GN=SEC63 PE=1 SV=2 - [SEC63_HU	1,97	1	1	1	1	6,712E5	0,000E0	1,60	1,97	1	1		0,00			760	87,9	5,31
Q14683	Structural maintenance of chromosomes protein 1A OS=Homo sapiens OX=9606 GN=SMC1A PE=1 SV=2 -	1,14	1	1	1	1	2,681E6	0,000E0	2,92	1,14	1	1		0,00			1233	143,1	7,64
Q8IW75	Serpin A12 OS=Homo sapiens OX=9606 GN=SERPINA12 PE=1 SV=1 - [SPA12_HUMAN]	4,11	1	2	2	2	1,774E6	0,000E0	0,00	4,11	2	2		0,00			414	47,1	9,29
Q8IY81	pre-rRNA processing protein FTSJ3 OS=Homo sapiens OX=9606 GN=FTSJ3 PE=1 SV=2 - [SPB1_HUMA	2,24	1	1	1	1	2,942E6	0,000E0	1,68	2,24	1	1		0,00			847	96,5	8,40
P36952	Serpin B5 OS=Homo sapiens OX=9606 GN=SERPINB5 PE=1 SV=2 - [SPB5_HUMAN]	5,87	1	2	2	3	1,552E7	0,000E0	2,06	5,87	2	3		0,00			375	42,1	6,05
Q8NBT2	Kinetochore protein Spc24 OS=Homo sapiens OX=9606 GN=SPC24 PE=1 SV=2 - [SPC24_HUMAN]	8,63	1	1	1	1	6,920E5	0,000E0	2,75	8,63	1	1		0,00			197	22,5	4,70
Q8WYJ6	Septin-1 OS=Homo sapiens OX=9606 GN=SEPT1 PE=1 SV=2 - [SEPT1_HUMAN]	4,36	1	1	1	1	4,166E6	0,000E0	2,14	4,36	1	1		0,00			367	41,9	5,80
Q92599	Septin-8 OS=Homo sapiens OX=9606 GN=SEPT8 PE=1 SV=4 - [SEPT8_HUMAN]	4,97	1	1	1	1	2,141E6	0,000E0	3,37	4,97	1	1		0,00			483	55,7	6,28
Q15637	Splicing factor 1 OS=Homo sapiens OX=9606 GN=SF1 PE=1 SV=4 - [SF01_HUMAN]	7,36	1	2	2	3	4,251E6	0,000E0	3,44	7,36	2	3		0,00			639	68,3	8,98
P62314	Small nuclear ribonucleoprotein Sm D1 OS=Homo sapiens OX=9606 GN=SNRPD1 PE=1 SV=1 - [SMD1_I	27,73	1	2	2	2	1,576E7	0,000E0	3,95	27,73	2	2		0,00			119	13,3	11,56
Q9NTJ3	Structural maintenance of chromosomes protein 4 OS=Homo sapiens OX=9606 GN=SMC4 PE=1 SV=2 - [S	1,16	1	1	1	1	1,904E6	0,000E0	4,21	1,16	1	1		0,00			1288	147,1	6,79
Q96P63	Serpin B12 OS=Homo sapiens OX=9606 GN=SERPINB12 PE=1 SV=1 - [SPB12_HUMAN]	6,67	1	3	3	3	1,418E7	0,000E0	3,81	6,67	3	3		0,00			405	46,2	5,53
Q9UIV8	Serpin B13 OS=Homo sapiens OX=9606 GN=SERPINB13 PE=1 SV=2 - [SPB13_HUMAN]	4,86	1	2	2	2	1,563E6	0,000E0	2,18	4,86	2	2		0,00			391	44,2	5,71
P29508	Serpin B3 OS=Homo sapiens OX=9606 GN=SERPINB3 PE=1 SV=2 - [SPB3_HUMAN]	29,74	1	6	11	17	1,401E8	0,000E0	29,69	29,74	11	17		0,00			390	44,5	6,81
P48594	Serpin B4 OS=Homo sapiens OX=9606 GN=SERPINB4 PE=1 SV=2 - [SPB4_HUMAN]	16,15	1	1	6	11	1,513E8	0,000E0	21,88	16,15	6	11		0,00			390	44,8	6,21
P50452	Serpin B8 OS=Homo sapiens OX=9606 GN=SERPINB8 PE=1 SV=2 - [SPB8_HUMAN]	3,21	1	1	1	1	1,750E6	0,000E0	1,78	3,21	1	1		0,00			374	42,7	5,57
P42285	Exosome RNA helicase MTR4 OS=Homo sapiens OX=9606 GN=MTREX PE=1 SV=3 - [MTREX_HUMAI	2,02	1	1	1	1	2,633E6	0,000E0	3,01	2,02	1	1		0,00			1042	117,7	6,52

O15270	Serine palmitoyltransferase 2 OS=Homo sapiens OX=9606 GN=SPTLC2 PE=1 SV=1 - [SPTC2_HUMAN]	2,67	1	1	1	1	0,000E0	0,000E0	1,97	2,67	1	1		0,00			562	62,9	7,78
P37108	Signal recognition particle 14 kDa protein OS=Homo sapiens OX=9606 GN=SRP14 PE=1 SV=2 - [SRP14_	8,82	1	1	1	1	6,336E5	0,000E0	0,00	8,82	1	1		0,00			136	14,6	10,04
Q9UHB9	Signal recognition particle subunit SRP68 OS=Homo sapiens OX=9606 GN=SRP68 PE=1 SV=2 - [SRP68_	2,39	1	1	1	1	2,121E6	0,000E0	2,26	2,39	1	1		0,00			627	70,7	8,56
O76094	Signal recognition particle subunit SRP72 OS=Homo sapiens OX=9606 GN=SRP72 PE=1 SV=3 - [SRP72_	2,83	1	1	1	1	5,289E5	0,000E0	0,00	2,83	1	1		0,00			671	74,6	9,26
Q13596	Sorting nexin-1 OS=Homo sapiens OX=9606 GN=SNX1 PE=1 SV=3 - [SNX1_HUMAN]	4,79	1	1	1	1	1,577E6	0,000E0	3,65	4,79	1	1		0,00			522	59,0	5,15
Q8TE77	Protein phosphatase Slingshot homolog 3 OS=Homo sapiens OX=9606 GN=SSH3 PE=1 SV=2 - [SSH3_HU	2,12	1	1	1	1	5,244E5	0,000E0	1,87	2,12	1	1		0,00			659	73,0	5,30
Q15019	Septin-2 OS=Homo sapiens OX=9606 GN=SEPT2 PE=1 SV=1 - [SEPT2_HUMAN]	4,43	1	1	1	1	5,301E6	0,000E0	2,76	4,43	1	1					361	41,5	6,60
Q14141	Septin-6 OS=Homo sapiens OX=9606 GN=SEPT6 PE=1 SV=4 - [SEPT6_HUMAN]	5,53	1	1	1	1	0,000E0	0,000E0	0,00	5,53	1	1		0,00			434	49,7	6,67
Q9UHD8	Septin-9 OS=Homo sapiens OX=9606 GN=SEPT9 PE=1 SV=2 - [SEPT9_HUMAN]	3,07	1	1	1	1	6,432E6	0,000E0	3,44	3,07	1	1		0,00			586	65,4	8,97
Q01105	Protein SET OS=Homo sapiens OX=9606 GN=SET PE=1 SV=3 - [SET_HUMAN]	10,00	1	2	2	4	3,893E7	0,000E0	8,34	10,00	2	4		0,00			290	33,5	4,32
Q15459	Splicing factor 3A subunit 1 OS=Homo sapiens OX=9606 GN=SF3A1 PE=1 SV=1 - [SF3A1_HUMAN]	3,03	1	1	1	1	5,070E6	0,000E0	2,14	3,03	1	1		0,00			793	88,8	5,22
O75368	SH3 domain-binding glutamic acid-rich-like protein OS=Homo sapiens OX=9606 GN=SH3BGRL PE=1 SV=	15,79	1	1	1	1	7,506E6	0,000E0	2,00	15,79	1	1		0,00			114	12,8	5,25
Q9NWH9	SAFB-like transcription modulator OS=Homo sapiens OX=9606 GN=SLTM PE=1 SV=2 - [SLTM_HUMAN	1,55	1	1	1	2	4,885E6	0,000E0	0,00	1,55	1	2		0,00			1034	117,1	7,87
O60749	Sorting nexin-2 OS=Homo sapiens OX=9606 GN=SNX2 PE=1 SV=2 - [SNX2_HUMAN]	4,82	1	1	1	1	1,649E6	0,000E0	4,33	4,82	1	1		0,00			519	58,4	5,12
P00441	Superoxide dismutase [Cu-Zn] OS=Homo sapiens OX=9606 GN=SOD1 PE=1 SV=2 - [SODC_HUMAN]	32,47	1	3	3	3	2,143E7	0,000E0	7,01	32,47	3	3		0,00			154	15,9	6,13
Q8N3U4	Cohesin subunit SA-2 OS=Homo sapiens OX=9606 GN=STAG2 PE=1 SV=3 - [STAG2_HUMAN]	4,22	1	2	2	2	3,191E6	0,000E0	0,00	4,22	2	2		0,00			1231	141,2	5,43
Q12874	Splicing factor 3A subunit 3 OS=Homo sapiens OX=9606 GN=SF3A3 PE=1 SV=1 - [SF3A3_HUMAN]	6,59	1	2	2	2	2,034E6	0,000E0	5,70	6,59	2	2		0,00			501	58,8	5,38
Q13435	Splicing factor 3B subunit 2 OS=Homo sapiens OX=9606 GN=SF3B2 PE=1 SV=2 - [SF3B2_HUMAN]	3,24	1	1	1	1	6,680E6	0,000E0	0,00	3,24	1	1		0,00			895	100,2	5,67
P23246	Splicing factor, proline- and glutamine-rich OS=Homo sapiens OX=9606 GN=SFPQ PE=1 SV=2 - [SFPQ_H	6,08	1	2	3	3	5,271E6	0,000E0	5,89	6,08	3	3		0,00			707	76,1	9,44
Q9H299	SH3 domain-binding glutamic acid-rich-like protein 3 OS=Homo sapiens OX=9606 GN=SH3BGRL3 PE=1 S	51,61	1	2	2	2	1,287E6	0,000E0	0,00	51,61	2	2		0,00			93	10,4	4,93
Q15046	Lysine--tRNA ligase OS=Homo sapiens OX=9606 GN=KARS PE=1 SV=3 - [SYK_HUMAN]	1,84	1	1	1	1	1,232E6	0,000E0	1,93	1,84	1	1		0,00			597	68,0	6,35
P31948	Stress-induced-phosphoprotein 1 OS=Homo sapiens OX=9606 GN=STIP1 PE=1 SV=1 - [STIP1_HUMAN]	3,68	1	1	1	1	1,331E7	0,000E0	2,03	3,68	1	1		0,00			543	62,6	6,80
Q9Y6E0	Serine/threonine-protein kinase 24 OS=Homo sapiens OX=9606 GN=STK24 PE=1 SV=1 - [STK24_HUMAN	3,39	1	1	1	1	3,860E5	0,000E0	0,00	3,39	1	1					443	49,3	5,69
Q9UJZ1	Stomatin-like protein 2, mitochondrial OS=Homo sapiens OX=9606 GN=STOML2 PE=1 SV=1 - [STML2_	8,99	1	2	2	2	4,950E6	0,000E0	1,93	8,99	2	2		0,00			356	38,5	7,39
P41252	Isoleucine--tRNA ligase, cytoplasmic OS=Homo sapiens OX=9606 GN=IARS PE=1 SV=2 - [SYIC_HUMAN	2,46	1	2	2	2	3,692E6	0,000E0	1,97	2,46	2	2		0,00			1262	144,4	6,15
O15020	Spectrin beta chain, non-erythrocytic 2 OS=Homo sapiens OX=9606 GN=SPTBN2 PE=1 SV=3 - [SPTN2_H	0,59	1	1	1	1	5,412E5	0,000E0	0,00	0,59	1	1		0,00			2390	271,2	6,11
Q08945	FACT complex subunit SSRP1 OS=Homo sapiens OX=9606 GN=SSRP1 PE=1 SV=1 - [SSRP1_HUMAN]	1,55	1	1	1	1	1,830E5	0,000E0	2,41	1,55	1	1		0,00			709	81,0	6,87
P49588	Alanine--tRNA ligase, cytoplasmic OS=Homo sapiens OX=9606 GN=AARS PE=1 SV=2 - [SYAC_HUMAN	4,34	1	2	2	2	1,943E6	0,000E0	3,43	4,34	2	2		0,00			968	106,7	5,53
P14868	Aspartate--tRNA ligase, cytoplasmic OS=Homo sapiens OX=9606 GN=DARS PE=1 SV=2 - [SYDC_HUMA	2,20	1	1	1	1	3,753E5	0,000E0	1,73	2,20	1	1		0,00			501	57,1	6,55
P49590	Probable histidine--tRNA ligase, mitochondrial OS=Homo sapiens OX=9606 GN=HARS2 PE=1 SV=1 - [SY	1,78	2	1	1	1	7,883E5	0,000E0	0,00	1,78	1	1		0,00			506	56,9	8,24
P23381	Tryptophan--tRNA ligase, cytoplasmic OS=Homo sapiens OX=9606 GN=WARS PE=1 SV=2 - [SYWC_HU	4,03	1	1	1	1	1,423E6	0,000E0	2,47	4,03	1	1		0,00			471	53,1	6,23
O00204	Sulfotransferase family cytosolic 2B member 1 OS=Homo sapiens OX=9606 GN=SULT2B1 PE=1 SV=2 - [	6,58	1	1	1	1	2,175E6	0,000E0	4,26	6,58	1	1		0,00			365	41,3	5,40
Q86TL2	Store-operated calcium entry regulator STIMATE OS=Homo sapiens OX=9606 GN=STIMATE PE=1 SV=1	2,72	1	1	1	1	7,247E6	0,000E0	0,00	2,72	1	1		0,00			294	33,2	8,15
P16949	Stathmin OS=Homo sapiens OX=9606 GN=STMN1 PE=1 SV=3 - [STMN1_HUMAN]	8,72	1	1	1	2	5,837E7	0,000E0	6,21	8,72	1	2		0,00			149	17,3	5,97
P54136	Arginine--tRNA ligase, cytoplasmic OS=Homo sapiens OX=9606 GN=RARS PE=1 SV=2 - [SYRC_HUMA	3,79	1	1	1	1	3,582E6	0,000E0	2,64	3,79	1	1		0,00			660	75,3	6,68
P37837	Transaldolase OS=Homo sapiens OX=9606 GN=TALDO1 PE=1 SV=2 - [TALDO_HUMAN]	6,82	1	2	2	2	9,799E5	0,000E0	0,00	6,82	2	2		0,00			337	37,5	6,81
O14776	Transcription elongation regulator 1 OS=Homo sapiens OX=9606 GN=TCERG1 PE=1 SV=2 - [TCRG1_HU	1,46	1	1	1	1	2,604E6	0,000E0	0,00	1,46	1	1		0,00			1098	123,8	8,65
Q7L0Y3	tRNA methyltransferase 10 homolog C OS=Homo sapiens OX=9606 GN=TRMT10C PE=1 SV=2 - [TM10C	4,47	1	1	1	1	0,000E0	0,000E0	0,00	4,47	1	1		0,00			403	47,3	9,36
Q13148	TAR DNA-binding protein 43 OS=Homo sapiens OX=9606 GN=TARDBP PE=1 SV=1 - [TADBP_HUMAN	2,90	1	1	1	1	3,564E5	0,000E0	0,00	2,90	1	1		0,00			414	44,7	6,19
P55072	Transitional endoplasmic reticulum ATPase OS=Homo sapiens OX=9606 GN=VCP PE=1 SV=4 - [TERA_H	14,14	1	8	8	8	1,790E7	0,000E0	17,16	14,14	8	8		0,00			806	89,3	5,26
P22735	Protein-glutamine gamma-glutamyltransferase K OS=Homo sapiens OX=9606 GN=TGM1 PE=1 SV=4 - [TG	2,69	1	2	2	2	1,282E7	0,000E0	1,85	2,69	2	2		0,00			817	89,7	6,04
Q15185	Prostaglandin E synthase 3 OS=Homo sapiens OX=9606 GN=PTGES3 PE=1 SV=1 - [TEBP_HUMAN]	7,50	1	1	1	1	7,257E6	0,000E0	0,00	7,50	1	1		0,00			160	18,7	4,54
Q15813	Tubulin-specific chaperone E OS=Homo sapiens OX=9606 GN=TBCE PE=1 SV=1 - [TBCE_HUMAN]	4,36	1	1	1	1	9,874E4	0,000E0	0,00	4,36	1	1		0,00			527	59,3	6,76
P53999	Activated RNA polymerase II transcriptional coactivator p15 OS=Homo sapiens OX=9606 GN=SUB1 PE=1	13,39	1	2	2	2	1,841E6	0,000E0	1,80	13,39	2	2		0,00			127	14,4	9,60
Q08188	Protein-glutamine gamma-glutamyltransferase E OS=Homo sapiens OX=9606 GN=TGM3 PE=1 SV=4 - [TG	12,70	1	8	8	10	3,826E7	0,000E0	19,08	12,70	8	10		0,00			693	76,6	5,86
Q96FV9	THO complex subunit 1 OS=Homo sapiens OX=9606 GN=THOC1 PE=1 SV=1 - [THOC1_HUMAN]	4,26	1	1	1	1	1,202E6	0,000E0	0,00	4,26	1	1		0,00			657	75,6	4,98
P11388	DNA topoisomerase 2-alpha OS=Homo sapiens OX=9606 GN=TOP2A PE=1 SV=3 - [TOP2A_HUMAN]	5,49	2	5	5	5	6,447E6	0,000E0	11,74	5,49	5	5		0,00			1531	174,3	8,72
Q16881	Thioredoxin reductase 1, cytoplasmic OS=Homo sapiens OX=9606 GN=TXNRD1 PE=1 SV=3 - [TRXR1_H	5,55	1	2	2	2	3,115E6	0,000E0	2,73	5,55	2	2		0,00			649	70,9	7,39
O43396	Thioredoxin-like protein 1 OS=Homo sapiens OX=9606 GN=TXNL1 PE=1 SV=3 - [TXNL1_HUMAN]	2,77	1	1	1	1	8,867E5	0,000E0	0,00	2,77	1	1		0,00			289	32,2	4,96
Q9UNS1	Protein timeless homolog OS=Homo sapiens OX=9606 GN=TIMELESS PE=1 SV=2 - [TIM_HUMAN]	1,66	1	1	1	1	5,274E5	0,000E0	0,00	1,66	1	1		0,00			1208	138,6	5,40
Q12974	Protein tyrosine phosphatase type IVA 2 OS=Homo sapiens OX=9606 GN=PTP4A2 PE=1 SV=1 - [TP4A2_	17,37	1	1	1	1	3,612E6	0,000E0	4,85	17,37	1	1					167	19,1	8,37
P60174	Triosephosphate isomerase OS=Homo sapiens OX=9606 GN=TPI1 PE=1 SV=3 - [TPIS_HUMAN]	25,17	1	4	4	4	5,602E7	0,000E0	5,28	25,17	4	4		0,00			286	30,8	5,92
P12270	Nucleoprotein TPR OS=Homo sapiens OX=9606 GN=TPR PE=1 SV=3 - [TPR_HUMAN]	0,76	1	1	1	1	3,081E5	0,000E0	0,00	0,76	1	1		0,00			2363	267,1	5,02
P02788	Lactotransferrin OS=Homo sapiens OX=9606 GN=LTF PE=1 SV=6 - [TRFL_HUMAN]	1,41	1	1	1	1	1,791E6	0,000E0	0,00	1,41	1	1		0,00			710	78,1	8,12
Q92734	Protein TFG OS=Homo sapiens OX=9606 GN=TFG PE=1 SV=2 - [TFG_HUMAN]	4,75	1	1	1	1	1,014E7	0,000E0	0,00	4,75	1	1		0,00			400	43,4	5,10
Q9H0E2	Toll-interacting protein OS=Homo sapiens OX=9606 GN=TOLLIP PE=1 SV=1 - [TOLIP_HUMAN]	3,28	1	1	1	1	1,164E6	0,000E0	0,00	3,28	1	1		0,00			274	30,3	5,97
P55327	Tumor protein D52 OS=Homo sapiens OX=9606 GN=TPD52 PE=1 SV=2 - [TPD52_HUMAN]	6,70	1	1	1	1	1,295E6	0,000E0	0,00	6,70	1	1					224	24,3	4,83
Q14258	E3 ubiquitin/ISG15 ligase TRIM25 OS=Homo sapiens OX=9606 GN=TRIM25 PE=1 SV=2 - [TRI25_HUM	3,33	1	2	2	2	6,975E5	0,000E0	0,00	3,33	2	2		0,00			630	70,9	8,09
Q99805	Transmembrane 9 superfamily member 2 OS=Homo sapiens OX=9606 GN=TM9SF2 PE=1 SV=1 - [TM9S2	1,36	1	1	1	1	4,354E5	0,000E0	0,00	1,36	1	1		0,00			663	75,7	7,44
Q15363	Transmembrane emp24 domain-containing protein 2 OS=Homo sapiens OX=9606 GN=TMED2 PE=1 SV=1	6,47	1	1	1	1	7,511E5	0,000E0	0,00	6,47	1	1		0,00			201	22,7	5,17
O43399	Tumor protein D54 OS=Homo sapiens OX=9606 GN=TPD52L2 PE=1 SV=2 - [TPD54_HUMAN]	4,37	1	1	1	1	5,080E6	0,000E0	2,38	4,37	1	1					206	22,2	5,36
A0AVT1	Ubiquitin-like modifier-activating enzyme 6 OS=Homo sapiens OX=9606 GN=UBA6 PE=1 SV=1 - [UBA6_	1,24	1	1	1	1	1,267E6	0,000E0	2,23	1,24	1	1		0,00			1052	117,9	6,14
P61088	Ubiquitin-conjugating enzyme E2 N OS=Homo sapiens OX=9606 GN=UBE2N PE=1 SV=1 - [UBE2N_HUM	23,03	1	2	2	2	2,290E6	0,000E0	0,00	23,03	2	2		0,00			152	17,1	6,57
Q93009	Ubiquitin carboxyl-terminal hydrolase 7 OS=Homo sapiens OX=9606 GN=USP7 PE=1 SV=2 - [UBP7_HUM	4,08	1	3	3	3	1,270E6	0,000E0	1,79	4,08	3	3		0,00			1102	128,2	5,55
Q9BQ50	Three prime repair exonuclease 2 OS=Homo sapiens OX=9606 GN=TREX2 PE=1 SV=1 - [TREX2_HUMAN	16,13	1	3	3	3	9,958E6	0,000E0	7,99	16,13	3	3		0,00			279	30,6	6,83
P62995	Transformer-2 protein homolog beta OS=Homo sapiens OX=9606 GN=TRA2B PE=1 SV=1 - [TRA2B_HUM	4,86	1	1	1	1	6,814E6	0,000E0	1,98	4,86	1	1		0,00			288	33,6	11,25
P19971	Thymidine phosphorylase OS=Homo sapiens OX=9606 GN=TYMP PE=1 SV=2 - [TYPH_HUMAN]	2,70	1	1	1	1	1,285E6	0,000E0	2,05	2,70	1	1		0,00			482	49,9	5,53
Q5T6F2	Ubiquitin-associated protein 2 OS=Homo sapiens OX=9606 GN=UBAP2 PE=1 SV=1 - [UBAP2_HUMAN]	0,71	1	1	1	1	6,033E5	0,000E0	0,00	0,71	1	1		0,00			1119	117,0	7,34
P49755	Transmembrane emp24 domain-containing protein 10 OS=Homo sapiens OX=9606 GN=TMED10 PE=1 SV=	5,02	1	1	1	1	5,134E6	0,000E0	1,91	5,02	1	1		0,00			219	25,0	7,44
Q92973	Transportin-1 OS=Homo sapiens OX=9606 GN=TNPO1 PE=1 SV=2 - [TNPO1_HUMAN]	2,23	1	1	1	1	2,193E6	0,000E0	1,76	2,23	1	1		0,00			898	102,3	4,98
P38606	V-type proton ATPase catalytic subunit A OS=Homo sapiens OX=9606 GN=ATP6V1A PE=1 SV=2 - [VAT	4,86	1	2	2	2	1,446E6	0,000E0	1,80	4,86	2	2		0,00			617	68,3	5,52
Q9UI12	V-type proton ATPase subunit H OS=Homo sapiens OX=9606 GN=ATP6V1H PE=1 SV=1 - [VATH_HUM	4,35	1	1	1	1	1,044E6	0,000E0	2,19	4,35	1	1		0,00			483	55,8	6,48
Q8NEY4	V-type proton ATPase subunit C 2 OS=Homo sapiens OX=9606 GN=ATP6V1C2 PE=1 SV=2 - [VATC2_H	3,98	1	1	1	1	2,859E5	0,000E0	0,00	3,98	1	1		0,00			427	48,7	6,14
O94874	E3 UFM1-protein ligase 1 OS=Homo sapiens OX=9606 GN=UFL1 PE=1 SV=2 - [UFL1_HUMAN]	2,52	1	1	1	1	1,550E6	0,000E0	0,00	2,52	1	1		0,00			794	89,5	6,79
Q16851	UTP--glucose-1-phosphate uridylyltransferase OS=Homo sapiens OX=9606 GN=UGP2 PE=1 SV=5 - [UGPA	3,54	1	1	1	1	4,929E6	0,000E0	2,00	3,54	1	1		0,00			508	56,9	8,15
P47985	Cytochrome b-c1 complex subunit Rieske, mitochondrial OS=Homo sapiens OX=9606 GN=UQCRFS1 PE=	8,76	1	1	1	1	2,909E6	0,000E0	2,12	8,76	1	1		0,00			274	29,6	8,32
P68036	Ubiquitin-conjugating enzyme E2 L3 OS=Homo sapiens OX=9606 GN=UBE2L3 PE=1 SV=1 - [UB2L3_HU	30,52	2	2	2	2	1,108E7	0,000E0	3,99	30,52	2	2					154	17,9	8,51
P54578	Ubiquitin carboxyl-terminal hydrolase 14 OS=Homo sapiens OX=9606 GN=USP14 PE=1 SV=3 - [UBP14_H	2,23	1	1	1	1	2,791E6	0,000E0	2,22	2,23	1	1		0,00			494	56,0	5,30
Q9NYU2	UDP-glucose:glycoprotein glucosyltransferase 1 OS=Homo sapiens OX=9606 GN=UGGT1 PE=1 SV=3 - [U	3,22	1	2	2	2	1,470E6	0,000E0	2,64	3,22	2	2		0,00			1555	177,1	5,63
O75643	U5 small nuclear ribonucleoprotein 200 kDa helicase OS=Homo sapiens OX=9606 GN=SNRNP200 PE=1 SY	2,29	1	3	3	3	1,698E6	0,000E0	4,60	2,29	3	3		0,00			2136	244,4	6,06
P45974	Ubiquitin carboxyl-terminal hydrolase 5 OS=Homo sapiens OX=9606 GN=USP5 PE=1 SV=2 - [UBP5_HUM	8,74	1	4	4	4	5,923E6	0,000E0	0,00	8,74	4	4		0,00			858	95,7	5,03
Q04323	UBX domain-containing protein 1 OS=Homo sapiens OX=9606 GN=UBXN1 PE=1 SV=2 - [UBXN1_HUM	13,47	1	1	1	1	6,189E6	0,000E0	3,33	13,47	1	1		0,00			297	33,3	5,25
P18206	Vinculin OS=Homo sapiens OX=9606 GN=VCL PE=1 SV=4 - [VINC_HUMAN]	4,41	1	4	4	4	4,234E6	0,000E0	0,00	4,41	4	4		0,00			1134	123,7	5,66
Q9UBQ0	Vacuolar protein sorting-associated protein 29 OS=Homo sapiens OX=9606 GN=VPS29 PE=1 SV=1 - [VPS	5,49	1	1	1	1	6,117E5	0,000E0	0,00	5,49	1	1					182	20,5	6,79
P55060	Exportin-2 OS=Homo sapiens OX=9606 GN=CSE1L PE=1 SV=3 - [XPO2_HUMAN]	2,16	1	1	1	1	5,489E6	0,000E0	4,63	2,16	1	1		0,00			971	110,3	5,77
P36543	V-type proton ATPase subunit E 1 OS=Homo sapiens OX=9606 GN=ATP6V1E1 PE=1 SV=1 - [VATE1_HU	3,98	2	1	1	1	0,000E0	0,000E0	0,00	3,98	1	1		0,00			226	26,1	8,00
Q16864	V-type proton ATPase subunit F OS=Homo sapiens OX=9606 GN=ATP6V1F PE=1 SV=2 - [VATF_HUMA	14,29	1	1	1	1	4,280E5	0,000E0	3,58	14,29	1	1		0,00			119	13,4	5,52
P45880	Voltage-dependent anion-selective channel protein 2 OS=Homo sapiens OX=9606 GN=VDAC2 PE=1 SV=2	4,08	1	1	1	1	0,000E0	0,000E0	0,00	4,08	1	1					294	31,5	7,56
Q9GZS3	WD repeat-containing protein 61 OS=Homo sapiens OX=9606 GN=WDR61 PE=1 SV=1 - [WDR61_HUMA	6,56	1	1	1	1	9,830E5	0,000E0	2,69	6,56	1	1					305	33,6	5,47
P21281	V-type proton ATPase subunit B, brain isoform OS=Homo sapiens OX=9606 GN=ATP6V1B2 PE=1 SV=3	11,35	1	2	2	2	1,599E6	0,000E0	0,00	11,35	2	2		0,00			511	56,5	5,81
Q9HAV4	Exportin-5 OS=Homo sapiens OX=9606 GN=XPO5 PE=1 SV=1 - [XPO5_HUMAN]	1,74	1	1	1	1	0,000E0	0,000E0	0,00	1,74	1	1		0,00			1204	136,2	5,80
Q9H267	Vacuolar protein sorting-associated protein 33B OS=Homo sapiens OX=9606 GN=VPS33B PE=1 SV=2 - [V	2,27	1	1	1	1	3,304E5	0,000E0	0,00	2,27	1	1		0,00			617	70,5	6,71
Q9NP79	Vacuolar protein sorting-associated protein VTA1 homolog OS=Homo sapiens OX=9606 GN=VTA1 PE=1 S	2,61	1	1	1	1	8,699E5	0,000E0	0,00	2,61	1	1		0,00			307	33,9	6,29
O14980	Exportin-1 OS=Homo sapiens OX=9606 GN=XPO1 PE=1 SV=1 - [XPO1_HUMAN]	5,14	1	3	3	3	2,654E6	0,000E0	4,54	5,14	3	3		0,00			1071	123,3	6,06
Q8WU90	Zinc finger CCCH domain-containing protein 15 OS=Homo sapiens OX=9606 GN=ZC3H15 PE=1 SV=1 - [	2,82	1	1	1	2	4,618E6	0,000E0	4,10	2,82	1	2		0,00			426	48,6	5,31
Q96KR1	Zinc finger RNA-binding protein OS=Homo sapiens OX=9606 GN=ZFR PE=1 SV=2 - [ZFR_HUMAN]	1,68	1	1	1	1	1,592E6	0,000E0	0,00	1,68	1	1		0,00			1074	116,9	9,04
Q14202	Zinc finger MYM-type protein 3 OS=Homo sapiens OX=9606 GN=ZMYM3 PE=1 SV=2 - [ZMYM3_HUM	1,46	1	1	1	1	4,837E5	0,000E0	1,66	1,46	1	1		0,00			1370	152,3	6,35
P80303	Nucleobindin-2 OS=Homo sapiens OX=9606 GN=NUCB2 PE=1 SV=3 - [NUCB2_HUMAN]	5,00	1	1	1	1	1,547E6	0,000E0	0,00	5,00	1	1		0,00			420	50,2	5,12

Supplemental Table 6. Ontology enrichment analysis of top 50 identified proteins. Enrichment analysis was performed at the msigDB portal among ontology gene sets (www.gsea-msigdb.org/gsea/msigdb).

Overlap Results

Collection(s):	C5
# overlaps shown:	88
# genesets in collections:	14765
# genes in comparison (n):	50
# genes in universe (N):	40071

Gene Set Name	# Genes in Gen	Description	# Genes in Ove	k/K	p-value	FDR q-value
GO_RNA_POLYMERASE_II_CORE_COMPLEX	15	RNA polymera:	4	0,2667	2,90E-09	3,75E-05
GO_NCRNA_TRANSCRIPTION	109	The transcriptio	6	0,055	5,08E-09	3,75E-05
GO_RNA_POLYMERASE_II_HOLOENZYME	79	A nuclear DNA	5	0,0633	5,18E-08	2,55E-04
GO_RNA_POLYMERASE_COMPLEX	108	Any complex tl	5	0,0463	2,49E-07	8,29E-04
GO_RNA_POLYMERASE_ACTIVITY	44	Catalysis of the	4	0,0909	2,81E-07	8,29E-04
**GO_NON_CANONICAL_WNT_SIGNALING_PATHWAY**	**151**	**The series of n**	**5**	**0,0331**	**1,31E-06**	**2,89E-03**
GO_PROTEIN_MODIFICATION_BY_SMALL_PROTEIN_CONJUGATION_OR_REMOVAL	1160	A protein modi	10	0,0086	1,43E-06	2,89E-03
GO_UBIQUITIN_LIKE_PROTEIN_TRANSFERASE_ACTIVITY	457	Catalysis of the	7	0,0153	1,57E-06	2,89E-03
GO_SNRNA_TRANSCRIPTION	74	The synthesis o	4	0,0541	2,31E-06	3,77E-03
GO_PROTEIN_MODIFICATION_BY_SMALL_PROTEIN_CONJUGATION	958	A protein modi	9	0,0094	2,56E-06	3,77E-03
GO_INCLUSION_BODY	78	A discrete intra	4	0,0513	2,86E-06	3,78E-03
GO_SYMBIOTIC_PROCESS	980	A process carric	9	0,0092	3,07E-06	3,78E-03
HP_PROXIMAL_MUSCLE_WEAKNESS	190	Proximal muscl	5	0,0263	4,05E-06	4,47E-03
GO_SUPRAMOLECULAR_COMPLEX	1311	A cellular comp	10	0,0076	4,24E-06	4,47E-03
GO_MYD88_INDEPENDENT_TOLL_LIKE_RECEPTOR_SIGNALING_PATHWAY	34	Any series of m	3	0,0882	1,06E-05	9,82E-03
GO_RNA_CAPPING	34	The sequence o	3	0,0882	1,06E-05	9,82E-03
HP_GENERALIZED_MUSCLE_WEAKNESS	118	Generalized mu	4	0,0339	1,48E-05	1,26E-02
GO_REGULATION_OF_POSTTRANSCRIPTIONAL_GENE_SILENCING	119	Any process tha	4	0,0336	1,53E-05	1,26E-02
GO_TRANSFERASE_COMPLEX_TRANSFERRING_PHOSPHORUS_CONTAINING_GROUPS	253	A transferase co	5	0,0198	1,62E-05	1,26E-02
GO_ESTABLISHMENT_OF_TISSUE_POLARITY	124	Coordinated or	4	0,0323	1,80E-05	1,32E-02
GO_POSITIVE_REGULATION_OF_VIRAL_TRANSCRIPTION	41	Any process tha	3	0,0732	1,88E-05	1,32E-02
GO_NUCLEOTIDYLTRANSFERASE_ACTIVITY	128	Catalysis of the	4	0,0312	2,04E-05	1,37E-02
GO_MICROTUBULE_CYTOSKELETON	1256	The part of the	9	0,0072	2,23E-05	1,40E-02
**GO_NEGATIVE_REGULATION_OF_NON_CANONICAL_WNT_SIGNALING_PATHWAY**	**6**	**Any process th**	**2**	**0,3333**	**2,28E-05**	**1,40E-02**
GO_CLATHRIN_DEPENDENT_ENDOCYTOSIS	45	An endocytosis	3	0,0667	2,50E-05	1,48E-02
GO_NUCLEAR_ENVELOPE	473	The double lipi	6	0,0127	2,68E-05	1,52E-02
GO_REGULATION_OF_GENE_SILENCING	139	Any process tha	4	0,0288	2,82E-05	1,54E-02
GO_SUPRAMOLECULAR_POLYMER	996	A polymeric su	8	0,008	3,02E-05	1,59E-02
GO_MORPHOGENESIS_OF_A_POLARIZED_EPITHELIUM	145	The morphogen	4	0,0276	3,33E-05	1,69E-02
GO_PROGRAMMED_NECROTIC_CELL_DEATH	50	A necrotic cell	3	0,06	3,44E-05	1,69E-02
HP_DISINHIBITION	153	Disinhibition	4	0,0261	4,10E-05	1,92E-02
HP_NEURONAL_LOSS_IN_THE_CEREBRAL_CORTEX	8	Neuronal loss ir	2	0,25	4,25E-05	1,92E-02
GO_WHOLE_MEMBRANE	1721	Any lipid bilay	10	0,0058	4,44E-05	1,92E-02
**GO_CELL_CELL_SIGNALING_BY_WNT**	**521**	**Any process th**	**6**	**0,0115**	**4,59E-05**	**1,92E-02**
GO_TRANSFERASE_COMPLEX	771	A protein comp	7	0,0091	4,62E-05	1,92E-02
GO_UBIQUITIN_LIKE_PROTEIN_LIGASE_BINDING	316	Interacting selec	5	0,0158	4,68E-05	1,92E-02
GO_ENZYME_ACTIVATOR_ACTIVITY	526	Binds to and in	6	0,0114	4,84E-05	1,93E-02
HP_TETRAPLEGIA_TETRAPARESIS	62	Tetraplegia/tetra	3	0,0484	6,56E-05	2,55E-02
GO_NECROTIC_CELL_DEATH	64	A type of cell d	3	0,0469	7,22E-05	2,73E-02
GO_REGULATION_OF_VIRAL_TRANSCRIPTION	65	Any process tha	3	0,0462	7,56E-05	2,79E-02
GO_REGULATION_OF_MORPHOGENESIS_OF_AN_EPITHELIUM	181	Any process tha	4	0,0221	7,88E-05	2,84E-02
GO_SPINDLE	367	The array of mi	5	0,0136	9,47E-05	3,04E-02
GO_CLATHRIN_COATED_PIT	71	A part of the en	3	0,0423	9,84E-05	3,04E-02
HP_HYPERTRIGLYCERIDEMIA	71	Hypertriglyceric	3	0,0423	9,84E-05	3,04E-02
GO_CLATHRIN_COAT_OF_ENDOCYTIC_VESICLE	12	A clathrin coat	2	0,1667	9,99E-05	3,04E-02
GO_NUCLEAR_INCLUSION_BODY	12	An intranuclear	2	0,1667	9,99E-05	3,04E-02
HP_INTRINSIC_HAND_MUSCLE_ATROPHY	12	Intrinsic hand n	2	0,1667	9,99E-05	3,04E-02
HP_PAIN	604	Pain	6	0,0099	1,04E-04	3,04E-02
GO_TRANSFERASE_ACTIVITY_TRANSFERRING_PHOSPHORUS_CONTAINING_GROUPS	879	Catalysis of the	7	0,008	1,05E-04	3,04E-02
GO_VIRAL_GENE_EXPRESSION	195	A process by w	4	0,0205	1,05E-04	3,04E-02
GO_TRANSCRIPTION_COUPLED_NUCLEOTIDE_EXCISION_REPAIR	73	The nucleotide-	3	0,0411	1,07E-04	3,04E-02
HP_ATRIAL_ARRHYTHMIA	73	Atrial arrhythm	3	0,0411	1,07E-04	3,04E-02
GO_SOMATIC_STEM_CELL_POPULATION_MAINTENANCE	75	Any process by	3	0,04	1,16E-04	3,23E-02
GO_ENVELOPE	1219	A multilayered	8	0,0066	1,24E-04	3,36E-02
GO_CELL_SURFACE_RECEPTOR_SIGNALING_PATHWAY_INVOLVED_IN_CELL_CELL_SIGNALING	626	Any cell surface	6	0,0096	1,26E-04	3,36E-02
GO_RNA_SPLICING_VIA_TRANSESTERIFICATION_REACTIONS	391	Splicing of RN	5	0,0128	1,27E-04	3,36E-02
HP_CONSTITUTIONAL_SYMPTOM	918	Constitutional :	7	0,0076	1,37E-04	3,37E-02
GO_ENDOLYSOSOME_MEMBRANE	14	The lipid bilaye	2	0,1429	1,38E-04	3,37E-02
GO_LOW_DENSITY_LIPOPROTEIN_PARTICLE_RECEPTOR_CATABOLIC_PROCESS	14	The chemical re	2	0,1429	1,38E-04	3,37E-02
HP_DISTAL_UPPER_LIMB_MUSCLE_WEAKNESS	14	Distal upper lin	2	0,1429	1,38E-04	3,37E-02
GO_RNA_BINDING	1975	Interacting selec	10	0,0051	1,39E-04	3,37E-02
GO_ORGANELLE_LOCALIZATION	649	Any process in	6	0,0092	1,53E-04	3,59E-02
GO_PROTEIN_CONTAINING_COMPLEX_BINDING	1258	Interacting selec	8	0,0064	1,53E-04	3,59E-02
GO_MEMBRANE_ORGANIZATION	937	A process whicl	7	0,0075	1,56E-04	3,59E-02
HP_ABNORMAL_MUSCLE_FIBER_MORPHOLOGY	220	Abnormal musc	4	0,0182	1,67E-04	3,76E-02
GO_NUCLEAR_PORE	85	Any of the num	3	0,0353	1,68E-04	3,76E-02
HP_GLIOSIS	86	Gliosis	3	0,0349	1,74E-04	3,83E-02
GO_TRANSCRIPTION_ELONGATION_FROM_RNA_POLYMERASE_II_PROMOTER	87	The extension c	3	0,0345	1,80E-04	3,87E-02
GO_CLATHRIN_COAT_OF_COATED_PIT	16	The coat found	2	0,125	1,81E-04	3,87E-02
GO_CYTOSKELETAL_PROTEIN_BINDING	979	Interacting selec	7	0,0072	2,03E-04	4,15E-02
GO_CARGO_ADAPTOR_ACTIVITY	17	Binding directly	2	0,1176	2,05E-04	4,15E-02
HP_ABNORMAL_MITOCHONDRIAL_MORPHOLOGY	17	Abnormal mito	2	0,1176	2,05E-04	4,15E-02
HP_PERSEVERATION	17	Perseveration	2	0,1176	2,05E-04	4,15E-02
GO_ACTIN_BINDING	437	Interacting selec	5	0,0114	2,13E-04	4,26E-02

GO_CONTRACTILE_FIBER	238	Fibers, compos	4	0,0168	2,26E-04	4,36E-02
HP_ABNORMAL_CIRCULATING_PROTEIN_LEVEL	443	Abnormal circu	5	0,0113	2,27E-04	4,36E-02
GO_CADHERIN_BINDING_INVOLVED_IN_CELL_CELL_ADHESION	18	Any cadherin bi	2	0,1111	2,30E-04	4,36E-02
HP_DYSCALCULIA	18	Dyscalculia	2	0,1111	2,30E-04	4,36E-02
GO_DNA_TEMPLATED_TRANSCRIPTION_INITIATION	249	Any process inv	4	0,0161	2,68E-04	4,87E-02
HP_HYPERLIPIDEMIA	100	Hyperlipidemia	3	0,03	2,71E-04	4,87E-02
HP_ABNORMAL_RESPIRATORY_SYSTEM_MORPHOLOGY	1028	Abnormal respi	7	0,0068	2,74E-04	4,87E-02
GO_CATALYTIC_COMPLEX	1372	A protein comp	8	0,0058	2,76E-04	4,87E-02
GO_ENDOLYSOSOME	20	An transient hy	2	0,1	2,86E-04	4,87E-02
HP_ABNORMAL_UPPER_MOTOR_NEURON_MORPHOLOGY	20	Abnormal uppe	2	0,1	2,86E-04	4,87E-02
HP_SUPRAVENTRICULAR_ARRHYTHMIA	102	Supraventricula	3	0,0294	2,88E-04	4,87E-02
GO_REGULATION_OF_ANIMAL_ORGAN_MORPHOGENESIS	254	Any process tha	4	0,0157	2,89E-04	4,87E-02
HP_HYPERTROPHIC_CARDIOMYOPATHY	254	Hypertrophic ca	4	0,0157	2,89E-04	4,87E-02
GO_INTRACELLULAR_TRANSPORT	1758	The directed mo	9	0,0051	2,90E-04	4,87E-02

Supplemental Table 7. Comparison of SFRP2 and SOX2 regulated genes with spatial gene expression profiles from glioblastoma tissue. **A**. The first column lists genes from the spatial gene expression data that positively correlated with SFRP2 expression with r>0.5. The second column describes the correlation to SFRP2 (r value). The third and fourth columns show the fold change value of the respective genes upon SFRP2 overexpression in U-2987 or SOX2 overexpression in U-2982. **B**. The same as for A, but with regard to SOX2. **C.** The first column lists genes from the spatial gene expression data higher expressed in VA areas than CT areas. Other two columns are as in column set A. **D**. The first column lists genes from the spatial gene expression data higher expressed in CT areas than in VA areas. Other two columns are as in column set A.

A. SFRP2 correlated genes in IVY glioblastoma

SFRP2 correlated genes	r	U2987-SFRP2 vs control	U2982-SOX2 vs control
SFRP2	1	1254,898	0,071
COL14A1	0,649	0,260	0,619
FAP	0,62	2,775	0,040
FBLN1	0,603	0,858	17,540
GATA6	0,601	0,581	2,435
COL21A1	0,594	0,550	7,422
CXCL12	0,579	47,102	0,076
ADAMTS2	0,565	325,795	0,043
FNDC1	0,565	0,010	0,022
COL11A1	0,558	0,288	1,979
BNC2	0,556	0,927	0,770
LTBP2	0,552	3,132	0,478
DAB2	0,527	1,569	0,271
SULF1	0,518	3,474	1,269
SPTLC3	0,513	1,216	0,634
ITGB5	0,508	0,889	0,928
COL1A1	0,506	25,666	0,083
COL6A3	0,504	1,570	0,393
GLT8D2	0,503	3,140	3,504
A4GALT	0,501	3,943	9,388

B. SOX2 correlated genes in IVY glioblastoma

SOX2 correlated genes	r	U2987-SFRP2 vs control	U2982-SOX2 vs control
SOX2	1	0,019	204,999
NLGN1	0,796	0,028	9,214
NOVA1	0,787	0,013	
PTPRZ1	0,77	0,001	1590,966
POU3F2	0,731	0,084	1,431
ZNF608	0,722	0,736	2,144
SOX6	0,716	0,005	86,237
MYEF2	0,713	0,477	8,693
DSEL	0,705	0,865	1,598
NCAM1	0,701	0,303	0,991
BCHE	0,695	0,342	1,450
KIF21A	0,684	0,387	161,282
QKI	0,683	0,841	1,248
ARHGEF26	0,681	0,163	157,650
SALL1	0,672	0,016	0,061
CLCC1	0,658	0,524	1,996
ID4	0,654	0,411	3,665
LRRN1	0,654	0,992	0,008
MANEAL	0,645	0,268	0,632
GPM6B	0,643	0,002	132,311
DCLK2	0,638	0,569	0,595
DDR1	0,637	0,762	6,411
SCD5	0,637	0,811	16,298
TRIM9	0,634	0,285	0,139
TUBB2B	0,634	0,531	0,823
SYT11	0,629	0,294	6,508
JAKMIP2	0,624	0,165	103,998
ZNF43	0,624	0,764	5,808
WHSC1	0,623	0,994	1,749
LRIG1	0,618	0,875	0,848
ZNF107	0,615	1,220	2,513
LPHN3	0,611	0,168	21,606
ZNF664	0,611	0,457	2,118
PBX1	0,611	0,551	2,057
ETV1	0,611	1,649	4,193
GKAP1	0,61	0,620	
SOX9	0,609	0,488	2,688
TMEM106B	0,604	0,601	1,447
PCDHB9	0,604	1,158	1,865
RAB3IP	0,598	0,501	1,359
CCDC88A	0,596	0,799	1,910
KIAA1430	0,596	0,936	1,989
BAALC	0,595	0,042	0,916
CHD7	0,594	0,833	5,800
CREB5	0,594	1,987	5,442
PGAP1	0,591	0,330	1,466
CYP2U1	0,591	0,779	2,745
MBOAT2	0,591	0,888	1,112
PEX12	0,589	0,531	2,029
RHOBTB3	0,589	3,305	0,546
ICA1L	0,588	0,237	0,727
IPO9	0,588	0,700	1,750
DBI	0,587	1,347	1,089
CCDC112	0,586	0,935	2,101
ZNF606	0,584	0,460	1,895
AGPAT5	0,583	0,514	2,428
LIFR	0,582	0,542	1,732
HES6	0,58	0,155	2,028
MAGI2	0,58	0,329	1,141
KIDINS220	0,58	0,490	1,506
ZNF738	0,577	1,130	2,212
TAF4	0,576	1,402	0,974
DIP2B	0,575	0,926	3,261
MTMR1	0,575	1,089	2,821
PM20D2	0,575	1,298	7,390
ZNF138	0,574	1,471	1,577
PSAT1	0,573	0,596	0,586
TRAF3IP2-AS1	0,571	0,943	1,887
RND2	0,569	0,113	16,457
ALDH3A2	0,569	0,214	2,865
ENOPH1	0,568	0,825	1,460
TNKS	0,567	0,734	1,552
STK33	0,562	0,199	8,791
SP4	0,562	0,934	1,669
FGF2	0,561	0,553	1,873
KLHL13	0,56	0,198	41,708
USP30	0,558	0,596	2,826
FUBP3	0,558	0,995	1,346
EPB41L5	0,557	0,484	3,546
TRIM2	0,555	0,685	7,233
GAS1	0,555	11,604	0,179
PTCD3	0,554	0,614	2,063
REV3L	0,552	2,799	1,869
PTN	0,551	0,504	9,020
BRD7	0,551	0,728	2,795
ITGB8	0,549	0,057	3,327
FAM161A	0,548	0,377	2,525
BARD1	0,548	0,661	2,295
PRMT3	0,548	0,717	1,429
ZNF462	0,546	0,463	1,769
MAGEF1	0,546	1,083	3,706
C14orf37	0,545	0,418	1,395
CTNND2	0,544	0,002	95,525
IFT81	0,544	0,302	2,524
CEP70	0,542	0,767	3,474
EIF2AK1	0,54	0,952	1,557
ZNF92	0,539	0,755	2,977
SOX11	0,537	0,010	1,445
ZC3H8	0,537	0,707	1,004
SPATA6	0,535	0,193	2,010
SBF2	0,535	0,961	1,087
KCTD3	0,534	0,830	1,012
DCLK1	0,533	0,024	0,535
TCF12	0,533	1,323	1,510
ZFP30	0,532	0,448	0,726
FAM199X	0,531	0,838	1,696
GPR125	0,53	0,631	4,814
FMNL2	0,529	0,409	0,451
FIBIN	0,529	8,504	3,522
TBC1D14	0,526	0,932	2,190
NRCAM	0,525	0,041	254,987
CKB	0,525	0,115	8,858
PSIP1	0,525	0,701	3,469
MNS1	0,524	0,173	1,354
ZNF566	0,524	0,474	0,940
ZSCAN30	0,524	1,037	1,353
ZNF829	0,522	0,711	1,081
GLCCI1	0,522	0,828	2,959
KIAA1211	0,521	0,033	
RSRC1	0,519	1,063	3,131
SNCAIP	0,518	0,008	26,563
ZNF721	0,517	1,129	1,828
GNG4	0,517	3,071	5,553
PHLPP1	0,516	0,320	5,483
CNOT4	0,516	0,965	1,867
GPR56	0,515	0,518	3,094
ZNF117	0,515	0,575	1,096

C. Genes high expressed in VA area compared with CT area in IVY glioblastoma

VA vs CT	fold difference	U2987-SFRP2 vs control	U2982-SOX2 vs control
HTRA3	2,399		0,090
PTGS2	2,358		72,715
SFRP2	2,865	1254,898	0,071
ADAMTS2	2,447	325,795	0,043
COL15A1	5,333	224,545	0,445
BDKRB2	5,997	61,287	0,116
LDB2	4,769	61,241	0,290
CXCL12	5,73	47,102	0,076
PLXDC1	7,774	37,555	0,042
VEGFC	2,181	34,510	0,196
FAM20A	3,873	32,515	0,073
COL1A1	15,531	25,666	0,083
PRR16	6,806	25,199	0,092
CSPG4	3,21	23,834	0,035
TP53I11	3,168	23,608	0,197
ANPEP	2,417	21,046	0,441
HMCN1	2,644	20,974	0,414
COL12A1	4,247	18,385	0,354
TGFBI	6,684	18,201	0,195
COL1A2	12,394	16,815	0,196
UNC5B	2,64	15,067	0,290
PCOLCE	8,191	14,275	0,315
DYSF	2,921	13,633	0,096
GPR85	3,092	12,229	0,160
PLAUR	2,489	12,164	0,479
COL5A1	2,947	11,998	0,195
IFITM2	4,577	11,540	0,262
ACTG2	18,748	11,455	0,393
COL3A1	38,731	10,834	0,059
MATN3	4,303	10,603	0,295
IL4R	3,384	10,347	0,268
GPR124	4,236	10,205	0,087
LOXL2	3,694	9,417	0,067
SNAI2	7,747	9,138	0,320
OLFML3	6,301	8,943	0,070
ITGA5	4,53	8,698	0,351
ENG	4,847	8,201	0,392
GPRC5C	4,499	7,906	0,048
CNN1	2,47	7,789	0,273
PMAIP1	2,691	7,135	0,095
JUNB	2,071	6,890	0,218
NREP	2,364	6,617	0,083
FRMD6	2,612	5,732	0,459
DACT1	2,724	5,499	0,250
COL4A1	16,747	5,420	0,157
BGN	4,818	5,244	0,029
FXYD5	2,769	5,040	0,183
ATP8B1	5,471	5,033	0,383
IL32	3,326	4,703	0,255
TBC1D4	3,053	4,429	0,424
DCN	16,577	4,367	0,026
ECM1	6,695	4,313	0,481
COL4A2	8,065	4,296	0,258
PDGFRB	9,775	4,282	0,194
ZNF503	2,029	4,009	0,152
ADAM12	3,847	3,936	0,227
MFAP2	6,067	3,881	0,391
TPM2	12,089	3,699	0,056
RCN3	2,783	3,680	0,325
TAGLN	14,449	3,664	0,043
NID2	12,78	3,584	0,078
POSTN	3,407	3,469	0,091
CALHM2	2,608	3,398	0,290
LUM	21,666	3,372	0,080
LOX	5,857	3,212	0,185
PRDM1	6,157	3,201	0,325
SERPINH1	6,516	3,163	0,160
FAM198B	2,722	3,149	0,145
LTBP2	2,058	3,132	0,478
P4HA2	2,634	3,000	0,274
DLC1	3,146	2,986	0,478
VGLL3	2,856	2,882	0,146
FAP	3,33	2,775	0,040
STARD8	2,373	2,762	0,088
PPIC	2,922	2,723	0,375
DSE	2,682	2,714	0,409
PLXNA2	2,329	2,705	0,279
MYO1B	12,732	2,591	0,211
ARHGEF17	2,268	2,503	0,309
VASP	4,4	2,413	0,380
ARHGAP24	2,826	2,304	0,329
MYL9	8,73	2,280	0,072
FHL2	4,849	2,234	0,367
NR2F2	5,656	2,109	0,347
TGFB1I1	2,417	2,108	0,384
EDIL3	2,566	2,045	0,417
AXL	2,416	7,456	0,505
MLKL	3,107	11,199	0,525
EGFL7	3,008	6,760	0,527
NOV	2,403	4,195	0,536
SLFN11	2,284	6,056	0,539
THBS1	9,381	2,186	0,548
ADAMTS1	5,918	23,208	0,549
RAMP2	5,509	2,060	0,557
EPS8L1	2,18	2,182	0,559
AFAP1L1	3,33	3,217	0,578
KIAA1462	3,353	2,516	0,579
TGM2	4,61	10,861	0,579
AKR1C3	2,338	10,546	0,580
SMTN	2,922	3,080	0,580
DUSP23	2,039	2,695	0,593
MYLK	5,094	8,331	0,595
PTPRM	2,829	51,413	0,596
B2M	2,143	2,916	0,597
ETS1	7,82	2,755	0,608
PHLDA2	3,26	20,064	0,608
INHBA	2,439	138,153	0,612
PLAC9	10,076	22,923	0,630
ADA	3,273	13,260	0,650
PLAGL1	2,764	2,306	0,669
PTPRB	4,992	4,770	0,680
COL5A2	8,728	3,569	0,694
IFITM1	3,777	70,455	0,699
KDELR3	8,102	2,896	0,706
PLK2	4,062	2,096	0,715
LEPRE1	3,283	2,823	0,720
SLC43A3	2,061	2,985	0,728
FOXP1	2,782	3,501	0,731
FKBP7	3,632	5,994	0,743
SLC38A2	2,128	2,104	0,744
OAF	4,381	31,750	0,758
ITPRIP	2,126	2,007	0,786
SELM	4,716	2,169	0,788
TMSB10	2,007	2,168	0,807
FBLIM1	2,523	3,592	0,815
MFGE8	2,177	2,759	0,856
MARVELD	2,391	6,408	0,873

D. Genes high expressed in CT area compared with VA area in IVY glioblastoma

CT vs VA	fold difference	U2987-SFRP2 vs control	U2982-SOX2 vs control
NOVA1	3,473	0,013	
MAOB	2,458	0,031	
KIAA1211	2,11	0,033	
S100B	3,849	0,055	
PTPRZ1	3,873	0,001	1590,966
NTRK2	4,4	0,016	850,692
GRIA4	2,973	0,004	479,409
TMOD1	2,239	0,022	276,489
NRCAM	2,179	0,041	254,987
GAP43	2,87	0,035	247,930
SOX2	4,154	0,019	204,999
GATM	4,417	0,055	188,793
CXADR	2,822	0,154	188,211
KIF21A	4,225	0,387	161,282
ARHGEF26	2,818	0,163	157,650
GPM6B	3,114	0,002	132,311
AQP4	4,602	0,002	117,154
JAKMIP2	2,679	0,165	103,998
TRIM36	2,093	0,244	99,331
CNR1	2,263	0,019	97,719
CTNND2	3,513	0,002	95,525
BBOX1	3,32	0,288	87,486
SOX6	2,621	0,005	86,237
LONRF2	2,013	0,127	78,940
FGFR3	2,384	0,051	62,464
FAM184A	2,149	0,008	55,055
METTL7A	2,271	0,387	54,258
RHOU	2,877	0,472	41,487
AIF1L	3,026	0,289	37,781
ATP1B2	3,677	0,034	31,046
THBS4	2,814	0,286	29,729
PIPOX	2,808	0,047	27,914
SNCAIP	2,088	0,008	26,563
LPHN3	2,343	0,168	21,606
CLU	4,142	0,112	20,497
DOK5	2,49	0,003	19,946
CDO1	3,352	0,055	19,696
RND2	2,703	0,113	16,457
CTNNA2	3,387	0,022	16,284
MLC1	3,419	0,003	15,826
GLDC	2,062	0,058	15,183
PLEKHB1	4,312	0,296	10,760
DTNA	3,422	0,251	10,321
SALL2	2,091	0,179	9,389
NLGN1	3,183	0,028	9,214
CKB	5,485	0,115	8,858
STK33	2,371	0,199	8,791
SYBU	2,71	0,402	8,774
PCDH9	3,161	0,448	8,759
ANK2	2,938	0,047	8,729
TM7SF2	3,055	0,360	8,213
PLA2G16	2,047	0,335	8,198
ABAT	3,38	0,238	8,154
FEZ1	3,04	0,212	8,124
MAP2	2,122	0,209	7,655
NDRG4	4,015	0,277	6,804
SYT11	2,306	0,294	6,508
PHLPP1	2,438	0,320	5,483
CRYAB	2,498	0,136	5,326
ALDH5A1	2,429	0,269	4,011
TTLL7	2,374	0,301	3,845
ID4	3,426	0,411	3,665
MAPK8IP1	2,629	0,069	3,489
HOXA7	2,241	0,480	3,450
ITGB8	3,163	0,057	3,327
CRYL1	2,257	0,485	3,256
AHCYL2	2,231	0,204	3,074
TMEM108	2,096	0,003	3,061
SV2A	3,136	0,408	2,939
GATS	2,412	0,212	2,893
SOX9	3,504	0,488	2,688
ATP6V0E2	3,713	0,331	2,572
LCAT	2,134	0,451	2,374
GDAP1	2,422	0,484	2,269
CEND1	2,225	0,035	2,160
AHCYL1	2,028	0,473	2,096
NTRK3	2,443	0,149	2,083
APLP1	4,563	0,149	2,048
PDE4B	2,043	0,140	2,038
HES6	3,717	0,155	2,028
SLC4A4	4,474	0,001	1,960
GFAP	2,185	0,004	1,837
SRI	2,254	0,423	1,685
BCHE	3,393	0,342	1,450
SOX11	2,217	0,010	1,445
TMEM59L	4,057	0,388	1,434
POU3F2	3,462	0,084	1,431
REEP2	3,078	0,143	1,422
ID2	2,315	0,482	1,388
ARNT2	3,232	0,294	1,258
CLIP3	3,026	0,321	1,255
MAGI2	2,46	0,329	1,141
ASIC1	2,207	0,232	1,105
APBA2	2,11	0,216	1,061
SLC1A3	3,635	0,347	1,015
SFXN5	2,502	0,431	1,013
NCAM1	4,384	0,303	0,991
GNAZ	2,527	0,345	0,926
BAALC	3,259	0,042	0,916
BEX1	3,033	0,182	0,912
SDC3	2,079	0,444	0,881
APC2	2,092	0,017	0,717
PTPRD	2,053	0,037	0,633
MANEAL	2,726	0,268	0,632
DCLK1	2,469	0,024	0,535
CRISPLD1	2,676	0,000	0,521
RAB6B	2,036	0,109	0,486
FMNL2	2,501	0,409	0,451
METRN	2,214	0,070	0,362
ENHO	3,378	0,098	0,248
EGLN3	3,081	0,328	0,231
ATP1B1	2,568	0,182	0,149
TRIM9	3,661	0,285	0,139
NAP1L3	2,26	0,305	0,087
MAP1A	2,13	0,072	0,082
RAMP1	3,059	0,225	0,072
SALL1	3,126	0,016	0,061
CORO2B	2,608	0,180	0,043
MAP6	2,521	0,005	0,037
RAB3IP	2,321	0,501	1,359
RNF157	2,845	0,502	26,103
CACNG4	2,105	0,502	49,176
GPSM2	2,224	0,504	1,125
PTN	2,098	0,504	9,020
ARL8A	2,299	0,506	1,325
BBS2	2,026	0,508	2,917
PPP2R2B	3,25	0,509	33,188

CHTOP	0,515	0,846	1,127
CRISPLD1	0,513	0,000	0,521
VANGL2	0,512	0,230	73,505
TSPAN3	0,512	0,989	4,169
GLDC	0,511	0,058	15,183
ABAT	0,511	0,238	8,154
GPSM2	0,511	0,504	1,125
PTAR1	0,51	0,547	1,715
TTC32	0,51	0,978	1,623
TNIK	0,509	0,617	1,994
OSGEPL1	0,509	0,826	1,762
TFDP2	0,509	1,051	3,310
RHOU	0,507	0,472	41,487
OSBPL6	0,507	0,525	0,882
FHL1	0,507	0,651	2,256
PRKCA	0,506	1,401	1,795
MKLN1	0,505	1,107	1,618
RMI1	0,503	0,853	2,400
SNRNP200	0,502	0,955	1,245
REPIN1	0,502	1,265	1,436
GRIK2	0,502	1,833	0,243
XPR1	0,5	0,265	1,206
PDP2	0,5	0,996	3,861

IL1R1	3,381	18,923	0,883
RAB32	2,601	3,759	0,887
SERPINE1	3,723	25,611	0,889
PDE7B	2,217	16,479	0,895
IGFBP6	3,217	5,419	0,899
TIMP3	2,072	11,732	0,901
SEC24D	3,491	2,160	0,918
BMP4	3,965	2,685	0,929
RUNX1	2,901	2,387	0,936
HDAC7	2,277	2,502	0,940
ARPC1B	3,728	2,352	0,957
EBF1	8,386	21,324	0,961
MESDC1	2,47	3,342	0,969
LAMB1	9,412	3,261	0,991
FHOD1	3,95	2,279	1,001
C15orf39	2,257	3,503	1,021
MYO1E	2,332	2,012	1,025
ACVR1	2,548	2,431	1,028
SNRK	2,6	2,053	1,043
MCAM	8,069	4,364	1,046
ADAMTS5	4,172	25,880	1,110
TNFAIP8	3,389	3,718	1,117
ZNF618	2,049	2,462	1,137
PLOD1	2,34	2,167	1,153
DUSP5	2,937	10,269	1,171
EHD4	3,541	2,705	1,204
DOCK6	2,405	3,640	1,240
CLIC1	4,073	7,010	1,282
CD2AP	2,246	2,139	1,283
CASP4	3,661	3,305	1,333
CD276	2,044	3,055	1,338
GLIPR1	2,421	2,393	1,341
B4GALT1	2,673	2,453	1,341
DOCK9	3,961	2,052	1,381
RASSF3	4,897	2,778	1,398
PARVB	2,756	3,706	1,434
ACSL5	2,289	10,104	1,437
TRIM38	2,881	11,639	1,453
RBPMS2	2,438	2,363	1,463
RARG	2,264	2,664	1,471
PAPSS2	8,624	3,633	1,494
PTPRK	3,782	2,199	1,546
OSBPL10	2,007	2,455	1,554
CDC42EP3	2,161	3,522	1,592
FILIP1L	7,183	3,019	1,595
STC1	8,513	5,611	1,650
MMP11	5,422	4,736	1,684
MXRA5	8,129	24,174	1,697
PXDN	7,891	5,844	1,714
PHLDB2	5,152	2,579	1,830
PROCR	5,68	4,962	1,831
GSN	2,366	2,858	1,866
RASGRP3	4,134	4,410	1,923
F2R	3,886	10,126	1,999
TGFBR2	5,916	3,378	2,021
EDEM1	2,065	2,066	2,089
SLC29A1	3,511	6,325	2,101
IFI27	5,063	41,437	2,108
JAG1	3,766	3,242	2,143
ELK3	2,72	4,250	2,296
HLA-E	2,026	3,917	2,312
NCOA7	2,628	5,922	2,351
TSPAN15	2,647	11,430	2,355
CTSC	2,197	3,100	2,415
ETS2	3,125	4,824	2,433
FAIM3	2,18	5,399	2,472
ISG20	2,377	27,014	2,517
SH2B3	3,683	4,808	2,693
EPHA2	2,333	2,365	2,821
TFPI	4,623	2,965	2,875
ITPR1	2,216	2,986	3,061
PMEPA1	3,116	2,317	3,132
NT5DC1	2,108	2,006	3,194
GLT8D2	2,902	3,140	3,504
PDE5A	5,929	16,646	3,536
TNFAIP3	2,53	5,196	3,601
DOK4	2,112	5,663	3,810
MICA	2,289	3,376	4,180
RNF149	2,319	2,016	4,223
DCBLD2	2,341	3,115	4,444
EDN1	5,34	6,358	4,600
SPRY4	2,309	2,084	4,972
FZD4	4,336	2,521	4,988
UACA	13,394	2,146	5,005
TNFSF10	3,015	14,225	5,102
ITGA4	8,188	2,028	5,172
F2RL2	2,379	176,582	5,242
PGF	5,838	9,577	5,521
PPM1H	2,977	24,023	5,559
HLA-DPB1	2,473	55,853	5,720
TNFAIP6	4,66	52,171	5,735
ECE1	2,48	2,869	6,100
IL7R	2,427	9,677	6,116
ADORA2B	4,169	2,161	6,803
SEMA3C	3,262	5,006	7,480
IFI44	2,179	3,529	7,826
ARHGAP18	4,913	3,766	7,838
CD55	4,582	3,873	10,215
TNFRSF1B	2,1	5,903	18,937
TBX3	2,83	2,885	19,385
JUP	3,636	12,651	22,254
FKBP11	2,392	2,242	31,240
ACE	4,267	19,494	31,417
AFAP1L2	3,169	6,613	40,570
OLFML2A	8,03	2,642	55,117
EDNRA	14,351	27,469	67,919
MTUS1	3,088	8,587	70,396
LURAP1L	2,652	3,638	78,715
IL8	2,52	69,569	167,086
CD200	2,112	2,168	563,589
FLT1	12,53	573,934	2234,541
HLA-DRA	2,123	107,033	
HLA-DRB1	2,04	66,715	
PLBD1	2,012	1,976	
KLF6	2,551	1,949	1,009
FURIN	2,08	1,942	1,515
STARD13	4,035	1,935	2,957
KLF11	3,113	1,923	0,974
SH3BP5	2,849	1,913	1,122
INPP5A	2,067	1,912	1,488
ARHGAP29	7,214	1,906	3,048
GALNT1	2,052	1,900	10,557
NOTCH3	4,145	1,892	0,940
AVEN	2,284	1,881	0,268
FSTL1	6,728	1,878	1,354
SLFN5	3,485	1,871	0,837
ATP10D	2,243	1,866	1,109
RBMS3	5,302	1,836	0,839
CDH11	4,161	1,832	0,744
FOXC1	6,757	1,831	2,301
KLF2	2,556	1,822	0,348
CYR61	3,913	1,818	0,706
TMED3	2,067	1,810	0,906
CTSS	2,115	1,800	15,800
FBN1	4,444	1,798	0,639
NRP1	4,761	1,798	0,610
SEC23A	2,639	1,796	1,161
FGD6	2,494	1,791	2,753
LGALS1	2,724	1,788	0,459
ANXA11	2,023	1,780	1,047
ITPRIPL2	3,319	1,777	1,412

PNMAL1	2,64	0,516	2,020
GPR56	2,936	0,518	3,094
OSBPL6	2,009	0,525	0,882
APBB1	2,218	0,530	2,198
TUBB2B	3,565	0,531	0,823
PPARGC1A	2,277	0,531	6,858
PLP1	6,722	0,534	1,697
DCXR	2,036	0,535	2,966
SCARA3	2,381	0,540	3,464
LIFR	2,733	0,542	1,732
CNIH3	2,028	0,545	0,770
NFIX	3,048	0,546	0,714
PBX1	2,079	0,551	2,057
FGF2	2,619	0,553	1,873
NIPAL3	2,131	0,557	3,215
HHLA3	2,075	0,558	1,799
IRS2	2,28	0,560	3,378
OLFM2	2,48	0,564	0,930
DCLK2	2,433	0,569	0,595
FAM169A	2,808	0,570	262,804
PLCD3	2,009	0,583	1,609
HSPA4L	2,224	0,587	34,491
GDI1	2,301	0,590	1,523
PSAT1	4,566	0,596	0,586
RUFY3	2,103	0,611	0,971
PHGDH	2,642	0,613	0,797
TNIK	2,331	0,617	1,994
GKAP1	2,53	0,620	
SHISA4	2,429	0,625	0,872
NDRG2	2,112	0,633	2,456
KHDRBS3	2,05	0,660	0,364
SEC14L2	2,105	0,664	1,583
CST3	2,819	0,673	0,498
ARAP2	2,007	0,676	47,058
TRIM2	4,127	0,685	7,233
ATAT1	2,072	0,688	2,457
PDE4DIP	2,25	0,695	0,332
TOX	2,051	0,701	0,253
ALDH6A1	2,555	0,702	4,299
FOXG1	2,909	0,706	0,626
MAP6D1	2,607	0,710	2,024
TRIM47	2,02	0,721	0,772
PTPRS	2,224	0,744	1,933
ACYP2	2,007	0,745	1,626
C12orf76	2,281	0,747	2,730
CADM4	2,071	0,757	7,631
GLUL	2,331	0,760	1,618
DDR1	3,857	0,762	6,411
VAMP2	2,246	0,763	1,511
TUBG2	2,621	0,782	1,158
GPT2	2,705	0,784	1,565
C10orf35	2,043	0,787	1,444
MEIS2	2,04	0,797	2,142
PRDX2	2,261	0,799	0,976
SCD5	3,874	0,811	16,298
ZFHX4	2,379	0,813	0,897
CLIP4	2,059	0,817	1,075
PEBP1	2,009	0,826	1,877
VSNL1	2,036	0,854	1,606
SNN	2,147	0,854	1,676
GLRB	2,819	0,861	3,376
SLC25A4	2,104	0,862	5,120
DSEL	2,373	0,865	1,598
ENO2	3,904	0,871	1,241
LHPP	2,417	0,871	1,264
LRIG1	2,602	0,875	0,848
NUDT11	2,314	0,881	0,069
GAD1	2,509	0,882	0,136
LANCL2	2,334	0,883	0,364
PAFAH1B3	2,138	0,889	0,378
AGT	4,843	0,910	0,033
METTL7B	2,908	0,912	0,025
CERS4	2,068	0,916	0,150
CLDN10	2,652	0,924	
RTN3	2,025	0,924	1,290
SH3BGR	2,321	0,927	4,498
MGST1	3,101	0,937	4,668
HIPK2	2,218	0,949	4,763
RNF182	2,646	0,958	3,105
TUBB2A	2,554	0,958	0,554
RHBDD2	2,192	0,966	1,842
C5orf30	2,108	0,971	0,703
TLCD1	2,707	0,973	2,816
DNAJB2	2,05	0,977	1,302
LRRN1	3,331	0,992	0,008
HSD17B6	2,796	1,003	0,467
SNTA1	2,846	1,008	0,672
MRAS	2,092	1,010	1,193
SLC6A9	2,294	1,024	0,388
DNM3	2,031	1,028	0,563
PTPRF	2,859	1,029	1,253
SLC6A8	3,068	1,046	1,013
DHRS13	2,207	1,060	2,464
WASF3	2,641	1,068	2,801
THRA	2,713	1,069	2,153
GABBR1	2,14	1,069	0,852
FMN2	2,913	1,080	1,740
FBXO44	2,216	1,081	1,085
TUBB4A	3,985	1,085	0,889
RTKN	2,032	1,099	2,087
FGFRL1	2,099	1,109	1,945
ZNF365	2,118	1,156	1,482
FBXL16	2,009	1,160	0,277
C8orf46	3,081	1,166	5,890
DBNDD1	2,519	1,189	6,559
SLC48A1	2,128	1,201	1,212
QDPR	2,578	1,204	1,188
ITPK1	2,109	1,244	1,226
TMEM144	2,874	1,299	4,068
C16orf45	2,118	1,310	0,482
PCDHGC3	3,534	1,311	1,300
NMB	3,243	1,321	0,274
VEGFA	4,202	1,322	0,308
ANK3	2,022	1,326	4,256
CBS	2,207	1,343	1,108
KAT2B	2,373	1,344	2,814
DBI	2,278	1,347	1,089
CNP	2,658	1,366	2,446
BEND6	2,119	1,396	0,791
TSC22D4	2,926	1,397	0,920
PRKCA	2,354	1,401	1,795
HK2	2,149	1,447	0,344
SLC4A3	2,127	1,451	0,431
MPP6	2,464	1,451	0,844
WASF1	2,002	1,463	0,998
DDIT3	2,172	1,473	1,535
BEX2	3,604	1,479	1,268
UCHL1	2,519	1,511	2,305
FSD1	2,23	1,539	1,221
ALDOC	7,114	1,592	34,351
MEIS1	2,76	1,640	1,061
ETV1	2,061	1,649	4,193
GRIK2	2,414	1,833	0,243
SLC22A17	2,825	1,862	1,315
PAX6	2,893	1,895	0,199
PCBP4	2,088	1,932	1,574
FBXO17	2,059	1,938	1,450
SDC4	2,632	1,944	2,966
MT1G	2,415	1,945	11,413
CTH	2,343	1,962	0,525
CREB5	2,274	1,987	5,442

TIMP1	3,085	1,765	0,411
PHACTR2	6,276	1,760	1,637
MYH9	4,805	1,756	0,416
YME1L1	2,093	1,753	1,422
MYL12A	5,27	1,739	1,154
PELO	2,552	1,734	1,274
TSPAN9	2,214	1,734	1,518
TMEM109	2,682	1,711	0,689
PARVA	2,321	1,701	0,344
BTG1	2,443	1,696	1,414
CARD6	2,248	1,685	2,425
RGS3	4,379	1,681	0,544
PFN1	2,055	1,678	0,691
BMPR2	2,032	1,668	1,224
PVRL2	2,876	1,666	1,311
TPM1	6,141	1,665	0,329
GRK5	2,524	1,658	1,676
FN1	16,931	1,647	0,424
CDKN2B	2,635	1,636	3,257
CNN2	4,567	1,636	0,368
GSTO1	2,384	1,630	1,416
PRSS23	10,383	1,621	0,676
PDLIM1	8,368	1,604	1,573
IVNS1ABP	2,365	1,601	0,657
RRAS	3,603	1,600	0,491
PLAU	2,043	1,596	3,692
TGFB3	5,117	1,596	2,018
EPAS1	2,178	1,596	0,451
SHC1	3,192	1,595	1,096
CBLB	2,068	1,590	1,444
LAMA4	6,096	1,589	0,572
POLD4	2,198	1,578	0,698
GMPPB	2,187	1,572	1,251
PLCB4	2,504	1,570	0,103
COL6A3	11,124	1,570	0,393
DAB2	3,831	1,569	0,271
PNP	4,523	1,569	0,865
WDFY1	2,014	1,563	1,387
GPX8	5,734	1,563	1,077
PLXND1	2,756	1,557	1,621
PLS3	3,273	1,557	1,529
TMEM165	2,177	1,556	1,684
RBMS1	2,561	1,554	1,130
SPARC	3,626	1,550	0,140
MYLIP	2,303	1,542	2,650
MCU	2,841	1,539	0,883
MYL12B	2,49	1,538	1,219
ANXA6	2,992	1,527	0,549
MYO1C	3,589	1,527	1,020
LAMA2	3,739	1,526	1198,967
ADAM19	2,088	1,522	0,898
POMP	2,357	1,514	1,619
NEDD4	2,255	1,498	1,688
PDE8A	2,485	1,496	0,771
DUSP1	3,018	1,496	0,988
HYAL2	3,698	1,495	2,894
SLFN12	3,02	1,483	1,767
ZMYM6NB	2,364	1,474	1,718
ILK	2,162	1,472	0,682
TEAD4	2,445	1,468	1,184
FAM114A1	2,673	1,466	1,663
PEAK1	2,251	1,462	1,943
VWA1	3,279	1,460	0,200
ARHGAP26	2,044	1,459	6,864
SLC35B3	2,292	1,438	2,429
PPFIBP1	2,637	1,437	1,607
RHBDL2	3,544	1,436	10,043
ARL15	2,188	1,425	1,787
NID1	9,333	1,420	3,664
IGFBP4	9,159	1,419	1,344
EXT1	2,024	1,410	0,366
TWIST1	4,711	1,409	1,006
AGRN	2,104	1,405	0,717
C6orf211	2,123	1,400	2,085
MCL1	2,004	1,399	1,687
OSTC	2,621	1,397	1,080
SLC46A3	3,835	1,392	1,397
BACE2	3,524	1,388	0,089
CASP7	2,185	1,387	2,046
ACTR3	2,129	1,382	0,721
LATS2	2,946	1,374	1,062
MAGED2	2,335	1,373	0,515
EPS8	4,228	1,372	2,820
SWAP70	2,323	1,371	1,727
CAP1	2,435	1,353	0,823
ADAM10	2,081	1,352	1,875
NAT1	2,744	1,350	1,536
TMEM184B	2	1,349	2,190
IKBIP	3,365	1,348	2,044
GDI2	2,247	1,347	0,846
RALB	2,35	1,339	0,503
THY1	6,808	1,335	0,828
RHOBTB1	2,346	1,335	0,687
HBEGF	2,382	1,332	4,269
TBC1D2B	2,172	1,331	1,507
RUNX2	3,041	1,331	0,394
RAI14	3,09	1,327	1,721
YAP1	2,17	1,325	0,820
ACOT9	2,177	1,313	0,668
GNAI1	2,32	1,310	0,915
CTHRC1	9,279	1,307	0,090
S100A11	2,074	1,306	0,296
ARHGEF12	2,152	1,303	3,303
WIPI1	2,359	1,301	1,084
ICAM3	2,665	1,301	0,959
ARF4	2,127	1,299	0,703
PTBP3	2,572	1,299	1,418
TPM4	3,873	1,293	0,662
NTAN1	2,446	1,291	1,180
SEMA3F	2,951	1,286	0,056
ARPC2	2,903	1,283	0,691
RBM3	2,511	1,279	0,484
NDUFA4L2	8,687	1,279	0,484
TBC1D9	2,174	1,278	3,013
ASB9	2,976	1,273	5,923
OSBPL9	2,02	1,272	1,649
IGFBP3	2,88	1,271	0,081
JAK1	2,155	1,264	0,923
SLC25A24	3,877	1,261	1,711
CMTM6	2,046	1,259	2,504
KLF10	2,66	1,259	0,797
DYNLT3	2,605	1,255	1,556
SRPX2	2,76	1,255	1,103
FKBP1A	2,788	1,250	0,835
ING1	2,326	1,248	1,988
EPSTI1	3,184	1,248	1,511
CMTM8	3,525	1,244	9,396
C1QTNF6	3,134	1,234	0,706
TMEM2	3,813	1,233	6,185
SLC26A2	2,659	1,231	1,064
C10orf118	2,283	1,224	1,408
LAMC1	6,065	1,219	0,537
GLRX3	2,339	1,215	0,946
CALD1	4,429	1,212	0,615
PTPRG	2,734	1,209	0,269
CYTH3	2,63	1,201	2,398
GMDS	2,216	1,197	3,582
TSPAN14	2,275	1,197	3,465
LIMS1	2,53	1,196	0,947
B3GNT2	5,345	1,191	3,409
SVIL	5,881	1,188	26,119

FADS2	2,331	2,025	1,270
SCD	4,787	2,103	0,715
LPAR1	2,336	2,385	2,109
MT1X	3,335	2,467	4,163
SERPINE2	2,675	2,543	1,284
LDOC1	2,157	2,579	0,945
EGFR	3,335	2,620	1,369
BNIP3	2,493	2,717	0,606
ZNF395	2,68	2,739	0,732
SHOX2	2,795	3,507	2,360
FGFR2	2,999	3,613	0,177
NRN1	2,022	3,740	1,093
SPOCK1	3,244	3,953	0,058
MT2A	2,186	3,978	1,791
LPPR4	2,009	5,182	0,969
DDIT4L	2,005	5,313	0,545
MT1F	2,462	5,378	20,919
AK4	4,241	5,842	0,113
MSTN	2,696	6,394	212,426
MBP	6,125	6,672	3,491
STMN3	2,612	6,975	0,119
FIBIN	2,581	8,504	3,522
GMPR	2,463	8,936	19,579
SNAP25	3,704	9,173	0,024
PTGDS	3,235	9,867	0,231
CXCL14	2,569	10,116	27,927
GAS1	2,945	11,604	0,179
MT1M	3,128	13,412	41,161
PCDH10	3,421	17,397	4,951
IGFBP5	2,285	18,654	2,602
LFNG	2,07	18,712	0,367
SCG2	2,29	23,815	25,199
CA12	3,57	61,352	0,516
CYP2J2	2,411		

UTRN	2,615	1,187	2,133
CREG1	2,129	1,183	0,435
HPRT1	2,548	1,181	1,772
UBE2J1	2,601	1,176	1,234
SERPINB1	2,42	1,176	309,633
PLEKHA1	2,861	1,168	0,649
KCTD10	2,283	1,160	1,063
UBE2D1	2,14	1,158	0,863
CREM	2,44	1,153	1,899
ARPC5	2,156	1,151	0,941
FAM160B1	2,346	1,146	1,396
PTRF	2,665	1,143	0,493
CPM	2,493	1,142	0,132
TBC1D8B	3,226	1,130	1,167
CENPW	2,341	1,123	2,002
MYOF	3,643	1,123	0,834
GRB10	2,196	1,114	2,144
COL5A3	2,735	1,110	0,105
SCPEP1	2,851	1,107	2,904
CFH	7,476	1,106	0,220
ARHGAP42	2,718	1,102	2,726
TPT1	2,212	1,100	0,793
PDGFD	4,828	1,096	32,809
OSTF1	3,488	1,096	1,408
LMNA	2,66	1,090	0,822
ABTB1	2,032	1,088	1,721
IGFBP7	6,826	1,087	1,030
RASAL2	2,311	1,087	0,777
SLK	2,151	1,077	0,970
RPL39L	4,366	1,062	1,652
RBPMS	3,459	1,062	0,356
TXNDC9	2,168	1,061	1,129
COMMD8	2,124	1,058	1,317
CDC42EP2	2,213	1,057	0,210
ANXA2	2,127	1,057	0,966
SOX18	3,815	1,056	0,044
NNMT	2,128	1,055	0,508
ACTA2	20,052	1,052	0,078
H2AFJ	2,48	1,052	6,075
EPHA3	4,08	1,050	0,624
SPRY1	4,247	1,043	8,886
FNDC3B	2,41	1,037	2,020
FCHO2	2,194	1,034	0,991
PQLC3	2,138	1,032	0,979
FZD6	3,535	1,029	0,971
EIF4EBP2	2,035	1,025	1,733
PGM2	2,155	1,021	1,829
DIAPH2	2,074	1,019	0,893
KIAA0247	2,124	1,017	1,512
LY96	2,187	1,016	2,992
COBLL1	4,337	1,014	2,768
PPM1F	2,183	1,011	1,319
SERPINI1	2,278	1,009	16,184
COL18A1	6,328	0,999	2,531
RSU1	2,397	0,993	0,530
TMEM173	5,222	0,981	0,752
ATP1B3	2,097	0,978	1,635
RECQL	2,196	0,972	1,844
PCTP	2,241	0,971	3,425
APOBEC3B	2,477	0,969	3,967
CCNG1	2,129	0,962	1,279
TUBB6	3,424	0,954	0,530
REEP3	2,231	0,950	1,760
VMP1	2,106	0,944	1,921
MDFIC	2,903	0,943	2,274
RILPL2	2,482	0,938	2,590
SGMS1	2,298	0,936	0,861
EIF4E2	2,026	0,934	0,910
CTNNB1	2,265	0,930	1,425
CREB3L2	2,207	0,930	2,736
LIMS2	2,143	0,929	0,088
AP2S1	2,354	0,928	0,664
LTBR	2,861	0,925	1,277
MSN	2,295	0,923	0,460
PCDH18	6,329	0,920	0,990
MTMR6	2,072	0,919	1,548
TBC1D1	2,214	0,911	1,921
MMP14	2,158	0,907	0,668
VPS29	2,144	0,905	1,381
METRNL	2,093	0,897	0,362
TM4SF1	6,492	0,894	0,949
TBX15	3,166	0,889	1,813
LASP1	2,15	0,873	1,002
MAST4	2,195	0,866	15,198
TNFAIP1	2,006	0,859	1,580
FBLN1	4,417	0,858	17,540
VANGL1	2,089	0,856	1,320
MGST2	2,821	0,847	7,918
SLC25A5	2,058	0,844	0,997
CTGF	8,976	0,838	9,319
IQGAP1	2,049	0,834	1,430
VAMP5	2,139	0,828	2,248
TMEM133	3,143	0,820	3,578
KITLG	2,805	0,815	0,506
GJC1	4,405	0,813	2,605
COL6A2	2,929	0,810	0,239
BMP1	2,409	0,809	0,378
CTSK	8,951	0,799	2,125
KANK2	2,359	0,797	0,626
IPO11	2,863	0,795	1,167
NT5DC2	2,515	0,794	0,787
NUDT15	2,064	0,793	1,947
EPHX4	3,369	0,789	#DIV/0!
FRMD8	2,466	0,771	1,060
STOM	3,633	0,768	2,189
RECK	2,309	0,768	1,656
MIR22HG	2,484	0,768	0,625
CEP55	2,308	0,766	1,563
CAV1	4,9	0,757	1,023
FCGRT	2,287	0,755	6,974
ATG4A	2,43	0,753	1,614
ENTPD7	2,175	0,752	1,938
LAYN	3,871	0,743	1,392
OLFML2B	7,095	0,737	0,580
PRKAR1A	2,231	0,735	1,606
NRP2	2,272	0,732	0,975
MYL6	2,377	0,721	0,960
CAV2	4,232	0,715	1,544
KIAA1217	2,117	0,715	0,449
VCL	5,01	0,714	0,857
PAWR	3,166	0,713	0,507
ANKRD50	2,12	0,711	2,620
CD46	2,858	0,699	1,709
CCL26	4,703	0,691	11,732
INSR	4,223	0,691	0,496
RGS5	18,316	0,685	4,929
TCN2	3,575	0,685	8,759
MEF2C	3,088	0,681	1,178
NQO1	4,165	0,678	1,225
TPBG	4,283	0,676	0,526
SLC9A3R2	3,189	0,674	1,055
KLHDC8B	3,365	0,673	0,873
TMTC3	2,352	0,659	2,196
APLN	2,943	0,657	0,105
MSRB3	4,695	0,643	1,578
CRIM1	2,2	0,640	1,151
GEM	2,544	0,635	1,094
DUSP6	2,538	0,631	3,417
CERK	2,136	0,630	2,581
RELL1	3,294	0,627	1,002
ISYNA1	2,348	0,621	2,316

CD59	2,749	0,610	0,763
MAP3K8	2,309	0,597	0,073
COL6A1	2,184	0,592	0,240
TAGLN2	2,296	0,588	1,370
SLC12A2	2,382	0,584	41,635
PERP	2,153	0,582	2,976
CCNDBP1	2,422	0,578	2,024
LBH	7,185	0,572	0,503
SLCO3A1	2,097	0,571	2,440
MECOM	5,276	0,564	2,725
AJUBA	2,856	0,561	0,373
HEG1	2,429	0,544	1,008
FAM101B	5,136	0,538	1,006
SMAGP	2,779	0,532	3,578
MYADM	3,383	0,511	0,766
TNS3	2,962	0,506	1,212
DNAJC1	2,493	0,506	0,596
CCDC102B	7,235	0,504	22,505
MAN1A1	3,104	0,500	1,825
ITGAV	2,016	0,492	0,717
SLC39A8	4,844	0,491	1,679
ITGA2	2,069	0,486	8,401
ZIC1	2,277	0,480	0,604
TDO2	9,812	0,478	0,196
GNG11	9,422	0,466	4,008
MAN2A1	2,057	0,466	2,961
FAS	2,326	0,462	0,108
KCNJ8	4,614	0,443	141,934
PTEN	4,337	0,433	0,562
TCIRG1	2,043	0,431	0,415
SDC2	6,027	0,431	0,410
MGP	12,465	0,394	19,882
PRKG1	5,53	0,390	0,077
APOLD1	6,353	0,387	3,369
CHST2	2,18	0,374	8,131
PODXL	4,442	0,350	1,677
TSPAN2	2,745	0,336	1,364
GUCY1B3	7,448	0,320	1,300
SLC7A11	2,266	0,317	2,766
RFTN1	2,549	0,294	0,464
CRIP1	23,102	0,279	0,588
COL14A1	2,342	0,260	0,619
AMOTL1	2,143	0,257	4,399
LHFP	2,624	0,242	1,927
RARB	2,219	0,237	3,006
LRRC8C	3,248	0,234	15,289
MAF	2,621	0,230	1,027
LEF1	5,006	0,209	0,502
PROS1	2,062	0,206	5,451
LZTS1	2,349	0,204	3,285
LPHN2	4,8	0,195	0,567
S1PR3	7,783	0,189	7,917
S100A4	2,684	0,185	2,290
SORBS2	3,037	0,106	5,471
MMP7	3,327	0,103	1,003
EFNB2	2,28	0,089	4,270
SLC40A1	2,284	0,076	128,662
SLCO2A1	4,006	0,071	0,322
PDGFB	5,876	0,065	38,972
SAMD5	2,898	0,062	98,391
ECM2	2,048	0,059	4,494
FAM107B	2,03	0,058	9,046
CD34	5,473	0,053	317,199
COCH	3,081	0,031	39,116
CALCRL	4,121	0,020	84,949
ASPN	4,85	0,013	5,865
CDH6	4,801	0,011	16,770
KCNJ2	5,679	0,004	57,364
ITIH5	3,065	0,003	0,033
A2M	5,234	0,002	0,122
SNTB1	4,458	0,002	0,610

Supplemental Table 8. STR profiling of U-2982, U-2987, and their SOX2 or SFRP2 overexpressing derivatives by AmpFISTR Identifiler PCR amplification Kit (Thermo Fisher). The analysis confirms that U-2982-control and U-2982-SOX2, and U-2987-control and U-2987-SFRP2 are more than 96% related, thus confirming no mixing up of cells has occurred during generation of the cultures. Inconsistencies were found for 3 alleles denoted with * of the 16 analyzed markers in U-2982 and 1 allele denoted with ** for U-2987.

	U-2982-control		U-2982-SOX2		U-2987-control		U-2987-SFRP2	
**Marker name**	**Allele 1**	**Allele 2**	**Allele 1**	**Allele 2**	**Allele 1**	**Allele 2**	**Allele 1**	**Allele 2**
D8S1179	13	13	13	13	13	13	13	13
D21S11*	28	30	30	30	28	30	28	30
D7S820	9	11	9	11	12	12	12	12
CSF1PO	10	13	10	13	10	12	10	12
D3S1358	14	17	14	17	15	15	15	15
TH01	9.3	9.3	9.3	9.3	7	7	7	7
D13S317	11	12	11	12	12	12	12	12
D16S539*	11	11	9	11	11	13	11	13
D2S1338	17	19	17	19	17	22	17	22
D19S433**	13	14	13	14	13	14	13	13
vWA	14	14	14	14	16	16	16	16
TPOX	8	11	8	11	8	8	8	8
D18S51	14	17	14	17	15	17	15	17
AMEL	X	Y	X	Y	X	Y	X	Y
D5S818	12	14	12	14	10	13	10	13
FGA*	20	20	20	21	19	20	19	20
Similarity to control (% match)			0.96025				0.96875	

Supplemental table 9. Table of included cell lines from CCLE. First column denotes full name used in CCLE gene expression data matrix. Second column denotes cell line name according to CCLE. Column 3 denotes if cell line was selected and included in figure 1 and subsequent analyses. The two following columns indicated the order of appearance of the cell lines in figure 1 from left to right and if they were clustered into cluster C1 or C2. The two last columnns indicate the mentioned histological origin of each cell lines and subsequent histological subtype.

**CCLE CENTRAL_NERVOUS_SYSTEM**	**Cell line**	**Included in Figure 1**	**Order of apperance in figure 1**	**Cluster**	**CCLE_Histology**	**CCLE_Hist_Subtype1**
U87MG_CENTRAL_NERVOUS_SYSTEM	U87MG	Included	1	C1	glioma	astrocytoma
AM38_CENTRAL_NERVOUS_SYSTEM	AM38	Included	2	C1	glioma	astrocytoma_Grade_IV
SNU489_CENTRAL_NERVOUS_SYSTEM	SNU489	Included	3	C1	glioma	astrocytoma_Grade_IV
SNU1105_CENTRAL_NERVOUS_SYSTEM	SNU1105	Included	4	C1	glioma	astrocytoma_Grade_IV
YH13_CENTRAL_NERVOUS_SYSTEM	YH13	Included	5	C1	glioma	astrocytoma_Grade_IV
DKMG_CENTRAL_NERVOUS_SYSTEM	DKMG	Included	6	C1	glioma	astrocytoma_Grade_IV
42MGBA_CENTRAL_NERVOUS_SYSTEM	42MGBA	Included	7	C1	glioma	astrocytoma_Grade_IV
LN443_CENTRAL_NERVOUS_SYSTEM	LN443	Included	8	C1	glioma	astrocytoma_Grade_IV
U178_CENTRAL_NERVOUS_SYSTEM	U178	Included	9	C1	glioma	astrocytoma_Grade_IV
U138MG_CENTRAL_NERVOUS_SYSTEM	U138MG	Included	10	C1	glioma	astrocytoma
U118MG_CENTRAL_NERVOUS_SYSTEM	U118MG	Included	11	C1	glioma	astrocytoma
BECKER_CENTRAL_NERVOUS_SYSTEM	BECKER	Included	12	C1	glioma	astrocytoma_Grade_IV
KG1C_CENTRAL_NERVOUS_SYSTEM	KG1C	Included	13	C1	glioma	astrocytoma_Grade_IV
CAS1_CENTRAL_NERVOUS_SYSTEM	CAS1	Included	14	C1	glioma	astrocytoma_Grade_IV
YKG1_CENTRAL_NERVOUS_SYSTEM	YKG1	Included	15	C1	glioma	astrocytoma_Grade_IV
SF295_CENTRAL_NERVOUS_SYSTEM	SF295	Included	16	C1	glioma	astrocytoma_Grade_IV
U343_CENTRAL_NERVOUS_SYSTEM	U343	Included	17	C1	glioma	astrocytoma_Grade_III
LN18_CENTRAL_NERVOUS_SYSTEM	LN18	Included	18	C1	glioma	astrocytoma_Grade_IV
CCFSTTG1_CENTRAL_NERVOUS_SYSTEM	CCFSTTG1	Included	19	C1	glioma	astrocytoma
LN382_CENTRAL_NERVOUS_SYSTEM	LN382	Included	20	C1	glioma	astrocytoma_Grade_IV
T98G_CENTRAL_NERVOUS_SYSTEM	T98G	Included	21	C1	glioma	astrocytoma_Grade_IV
A172_CENTRAL_NERVOUS_SYSTEM	A172	Included	22	C2	glioma	astrocytoma_Grade_IV
LN428_CENTRAL_NERVOUS_SYSTEM	LN428	Included	23	C2	glioma	astrocytoma_Grade_IV
SF172_CENTRAL_NERVOUS_SYSTEM	SF172	Included	24	C2	glioma	astrocytoma_Grade_IV
8MGBA_CENTRAL_NERVOUS_SYSTEM	8MGBA	Included	25	C2	glioma	astrocytoma_Grade_IV
GMS10_CENTRAL_NERVOUS_SYSTEM	GMS10	Included	26	C2	glioma	astrocytoma_Grade_IV
LN215_CENTRAL_NERVOUS_SYSTEM	LN215	Included	27	C2	glioma	astrocytoma_Grade_IV
M059K_CENTRAL_NERVOUS_SYSTEM	M059K	Included	28	C2	glioma	astrocytoma_Grade_IV
KNS60_CENTRAL_NERVOUS_SYSTEM	KNS60	Included	29	C2	glioma	astrocytoma_Grade_IV
LNZ308_CENTRAL_NERVOUS_SYSTEM	LNZ308	Included	30	C2	glioma	astrocytoma_Grade_IV
KS1_CENTRAL_NERVOUS_SYSTEM	KS1	Included	31	C2	glioma	astrocytoma_Grade_IV
LN464_CENTRAL_NERVOUS_SYSTEM	LN464	Included	32	C2	glioma	astrocytoma_Grade_IV
SNU466_CENTRAL_NERVOUS_SYSTEM	SNU466	Included	33	C2	glioma	astrocytoma_Grade_IV
LN229_CENTRAL_NERVOUS_SYSTEM	LN229	Included	34	C2	glioma	astrocytoma_Grade_IV
LN235_CENTRAL_NERVOUS_SYSTEM	LN235	Included	35	C2	glioma	astrocytoma_Grade_IV
LN340_CENTRAL_NERVOUS_SYSTEM	LN340	Included	36	C2	glioma	astrocytoma_Grade_IV
DBTRG05MG_CENTRAL_NERVOUS_SYSTEM	DBTRG05MG	Included	37	C2	glioma	astrocytoma_Grade_IV
GB1_CENTRAL_NERVOUS_SYSTEM	GB1	Included	38	C2	glioma	astrocytoma_Grade_IV
TM31_CENTRAL_NERVOUS_SYSTEM	TM31	Included	39	C2	glioma	astrocytoma
SNU201_CENTRAL_NERVOUS_SYSTEM	SNU201	Included	40	C2	glioma	astrocytoma_Grade_IV
SNB19_CENTRAL_NERVOUS_SYSTEM	SNB19	Included	41	C2	glioma	astrocytoma_Grade_IV
U251MG_CENTRAL_NERVOUS_SYSTEM	U251MG	Included	42	C2	glioma	astrocytoma
KNS81_CENTRAL_NERVOUS_SYSTEM	KNS81	Included	43	C2	glioma	astrocytoma_Grade_IV
SNU626_CENTRAL_NERVOUS_SYSTEM	SNU626	Included	44	C2	glioma	astrocytoma_Grade_IV
KNS42_CENTRAL_NERVOUS_SYSTEM	KNS42	Included	45	C2	glioma	NS
SF767_CENTRAL_NERVOUS_SYSTEM	SF767	Not included	#N/A		#N/A	#N/A
SNU738_CENTRAL_NERVOUS_SYSTEM	SNU738	Not included	#N/A		glioma	oligodendroglioma
GAMG_CENTRAL_NERVOUS_SYSTEM	GAMG	Not included	#N/A		glioma	NS
H4_CENTRAL_NERVOUS_SYSTEM	H4	Not included	#N/A		glioma	NS
D283MED_CENTRAL_NERVOUS_SYSTEM	D283MED	Not included	#N/A	mitive_neuroectodermal_tumour-me		NS
NMCG1_CENTRAL_NERVOUS_SYSTEM	NMCG1	Not included	#N/A		glioma	NS
KALS1_CENTRAL_NERVOUS_SYSTEM	KALS1	Not included	#N/A		glioma	NS
D341MED_CENTRAL_NERVOUS_SYSTEM	D341MED	Not included	#N/A	mitive_neuroectodermal_tumour-me		NS
SW1088_CENTRAL_NERVOUS_SYSTEM	SW1088	Not included	#N/A		glioma	NS
HS683_CENTRAL_NERVOUS_SYSTEM	HS683	Not included	#N/A		glioma	NS
ONS76_CENTRAL_NERVOUS_SYSTEM	ONS76	Not included	#N/A	mitive_neuroectodermal_tumour-me		NS
DAOY_CENTRAL_NERVOUS_SYSTEM	DAOY	Not included	#N/A	mitive_neuroectodermal_tumour-me		NS
F5_CENTRAL_NERVOUS_SYSTEM	F5	Not included	#N/A		meningioma	NS
A1207_CENTRAL_NERVOUS_SYSTEM	A1207	Not included	#N/A		glioma	NS
IOMMLEE_CENTRAL_NERVOUS_SYSTEM	IOMMLEE	Not included	#N/A		meningioma	NS
CH157MN_CENTRAL_NERVOUS_SYSTEM	CH157MN	Not included	#N/A		meningioma	NS
GI1_CENTRAL_NERVOUS_SYSTEM	GI1	Not included	#N/A		glioma	gliosarcoma
SW1783_CENTRAL_NERVOUS_SYSTEM	SW1783	Not included	#N/A		glioma	astrocytoma_Grade_III
LN319_CENTRAL_NERVOUS_SYSTEM	LN319	Not included	#N/A		glioma	astrocytoma_Grade_III
GOS3_CENTRAL_NERVOUS_SYSTEM	GOS3	Not included	#N/A		glioma	astrocytoma
MOGGCCM_CENTRAL_NERVOUS_SYSTEM	MOGGCCM	Not included	#N/A		glioma	astrocytoma
1321N1_CENTRAL_NERVOUS_SYSTEM	1321N1	Not included	#N/A		glioma	astrocytoma
MOGGUVW_CENTRAL_NERVOUS_SYSTEM	MOGGUVW	Not included	#N/A		glioma	astrocytoma
SF126_CENTRAL_NERVOUS_SYSTEM	SF126	Not included	#N/A		glioma	astrocytoma

Supplemental Table 10. Antibodies and qPCR primers.

Antibodies	Catalog number	Comapany
SOX2	AB5603	EDM Millipore
SFRP2	sc-365524	Santa Cruz
FN1	610078	BD bioscience
PDGFRA	sc-338	Santa Cruz
PDGFRB	sc-432	Santa Cruz
CCNB1	sc-245	Santa Cruz
CCND1	sc-8396	Santa Cruz
CCNE1	ab7959-1	Abcam
PARP	ab194586	Abcam
Beta-catenin	sc-7963	Santa Cruz
Non-Phospho (Active) beta-catenin (Ser45) (D2U8Y)	19807p	Cell signaling technology
Brn2/POU3F2	12137s	Cell signaling technology
KLF4	12173	Cell signaling technology
P-AKT(Ser473)	9271	Cell signaling technology
AKT	9272	Cell signaling technology
P-ERK	sc-7383	Santa Cruz
ERK	9102	Cell signaling technology
GAPDH	sc-32233	Santa Cruz
**Primers**	**Forward**	**Reverse**
human SOX2	TGCGAGCGCTGCACAT	GCAGCGTGTACTTATCCTTCTTCA
human SFRP2	ACCGAGGAAGCTCCAAAG	TGGTATCTCGGTTGATGTAGG
human ARG	TGGACAGACTAGGAATTGGCA	CCAGTCCGTCAACATCAAAACT
human MRC1	CTACAAGGGATCGGGTTTATGGA	TTGGCATTGCCTAGTAGCGTA
human CD80	GGCCCGAGTACAAGAACCG	TCGTATGTGCCCTCGTCAGAT
human IL-12	CCTTGCACTTCTGAAGAGATTGA	ACAGGGCCATCATAAAAGAGGT
human IL-10	TCAAGGCGCATGTGAACTCC	GATGTCAAACTCACTCATGGCT
